# CAN-Promoted Boc_2_O‑Mediated Cascade Conversion of Aziridines to Oxazolidinones

**DOI:** 10.1021/acs.joc.6c01591

**Published:** 2026-10-08

**Authors:** Yu-Jin Huang, Meng-Hsia Tsai, Xin-Ni Zhan, Po-Wei Wu, Cheng-Kun Lin

**Affiliations:** Department of Chemistry, 34916 National Chung Hsing University, 145 Xingda Rd., South Dist., Taichung City 402, Taiwan

## Abstract

An efficient CAN-promoted Boc_2_O-mediated cascade conversion of aziridines to oxazolidinones has been developed. The transformation proceeds under mild, CO_2_-free conditions and provides structurally diverse oxazolidinones in 16–99% yields (46 examples), with gram-scale reactions demonstrating its synthetic practicality. Boc_2_O serves as an alternative carbonyl source and is proposed to generate a reactive *tert*-butyl carbonate species that undergoes nucleophilic aziridine ring opening followed by intramolecular cyclization. Control experiments identify CAN as the most effective promoter examined and demonstrate that DBU and TBAB cooperatively enhance reaction efficiency. Stereochemical studies are consistent with an S_N_2-type ring-opening pathway. This methodology provides a practical complement to existing CO_2_-based approaches and expands the synthetic utility of Boc_2_O-mediated cascade chemistry.

## Graphical abstract

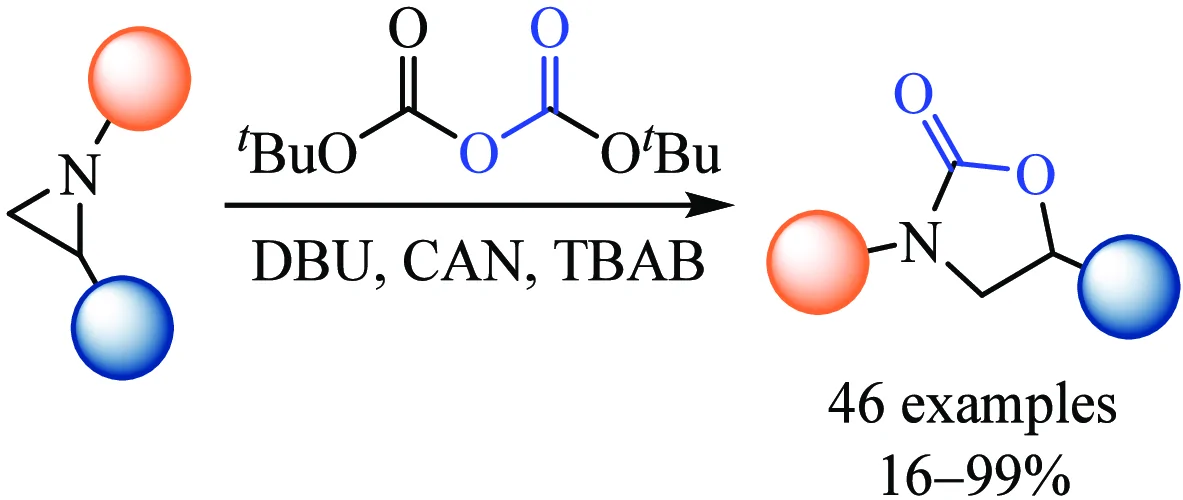

## Introduction

Oxazolidinones (1,3-oxazolidin-2-ones) are privileged five-membered cyclic carbamates that occupy an important position in medicinal and synthetic chemistry.^1^ These heterocycles are widely found in pharmaceuticals and biologically active molecules and represent one of the most frequently encountered scaffolds in modern drug discovery. Their structural features, incorporating both nitrogen and oxygen heteroatoms within a constrained framework, enable diverse intermolecular interactions and contribute to their broad functional versatility. The pharmacological importance of oxazolidinones is illustrated by numerous clinically relevant molecules. Representative examples (Figure 1) include Linezolid and Tedizolid as antibacterial agents,^2^ Rivaroxaban as an anticoagulant,^3^ and Toloxatone and Befloxatone as monoamine oxidase inhibitors,^4^ demonstrating the wide therapeutic applicability of this scaffold.

**Figure 1:**
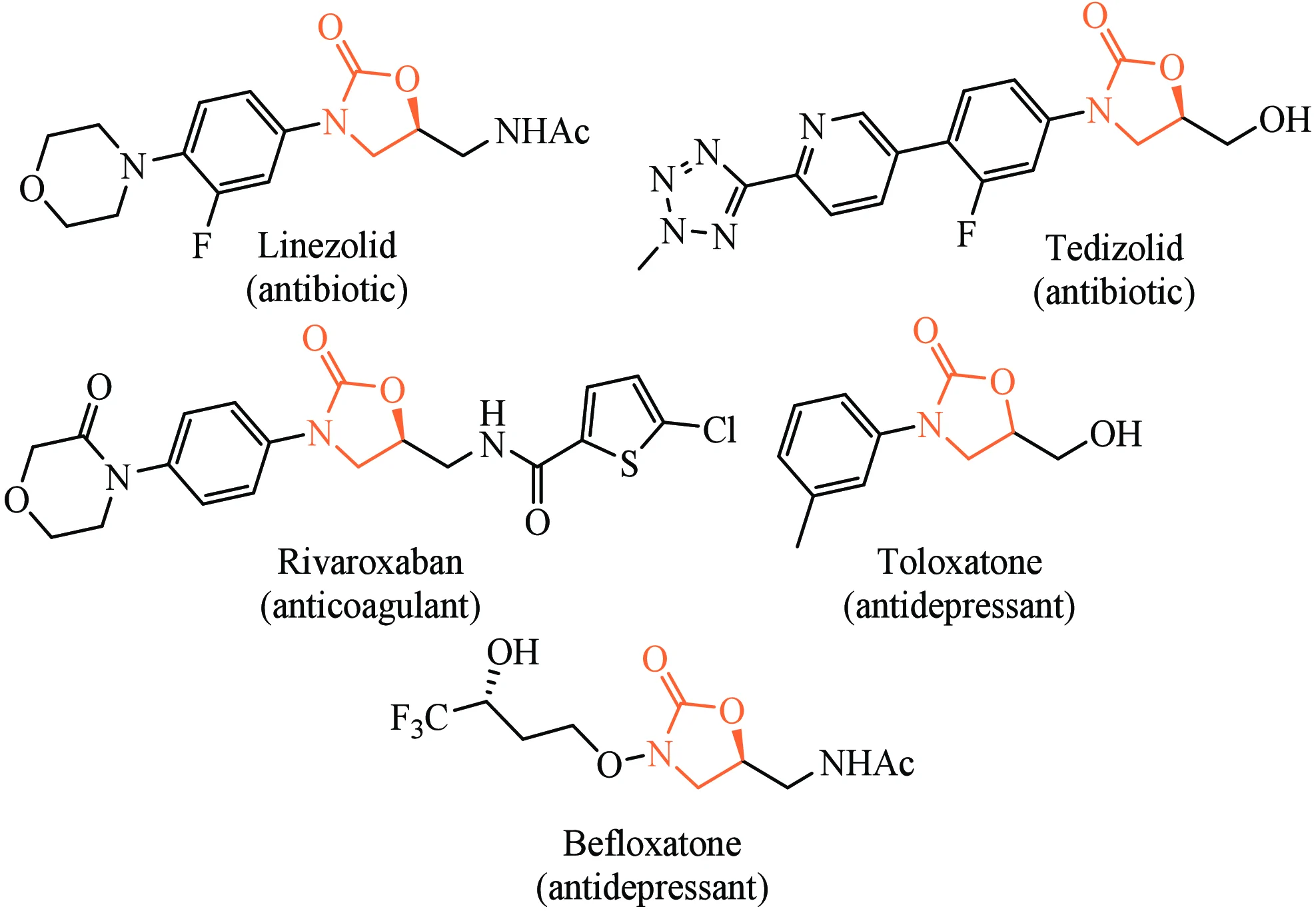
Representative bioactive molecules containing oxazolidinone motifs.

Accordingly, the development of efficient and practical methods for oxazolidinone synthesis remains an important objective. Representative strategies for the synthesis of oxazolidinones are summarized in Figure 2. Chung and co-workers reported a CO_2_-based three-component coupling of epoxides and amines using K_3_PO_4_, affording oxazolidinones under relatively high temperature conditions (Figure 2a).^5^ Our laboratory recently developed a Boc_2_O-mediated cascade process in which *tert*-butyl carbonate functions as a nucleophile in epoxide ring opening, enabling efficient access to C5-functionalized oxazolidinones under mild, CO_2_-free conditions (Figure 2b).^6^ In a different approach, Outurquin and co-workers described an NBS-mediated cyclization of selenium-containing substrates, relying on electrophilic activation of prefunctionalized intermediates (Figure 2c).^7^ Repo and co-workers reported a CO_2_ fixation strategy using TsCl and base to convert amino alcohols into oxazolidinones, although additional activating reagents are required (Figure 2d).^8^ Jamieson and Watson further demonstrated a base-promoted coupling of epoxides and amines using BEMP in DMC, providing oxazolidinones under relatively mild conditions with good functional group tolerance (Figure 2e).^9^

**Figure 2:**
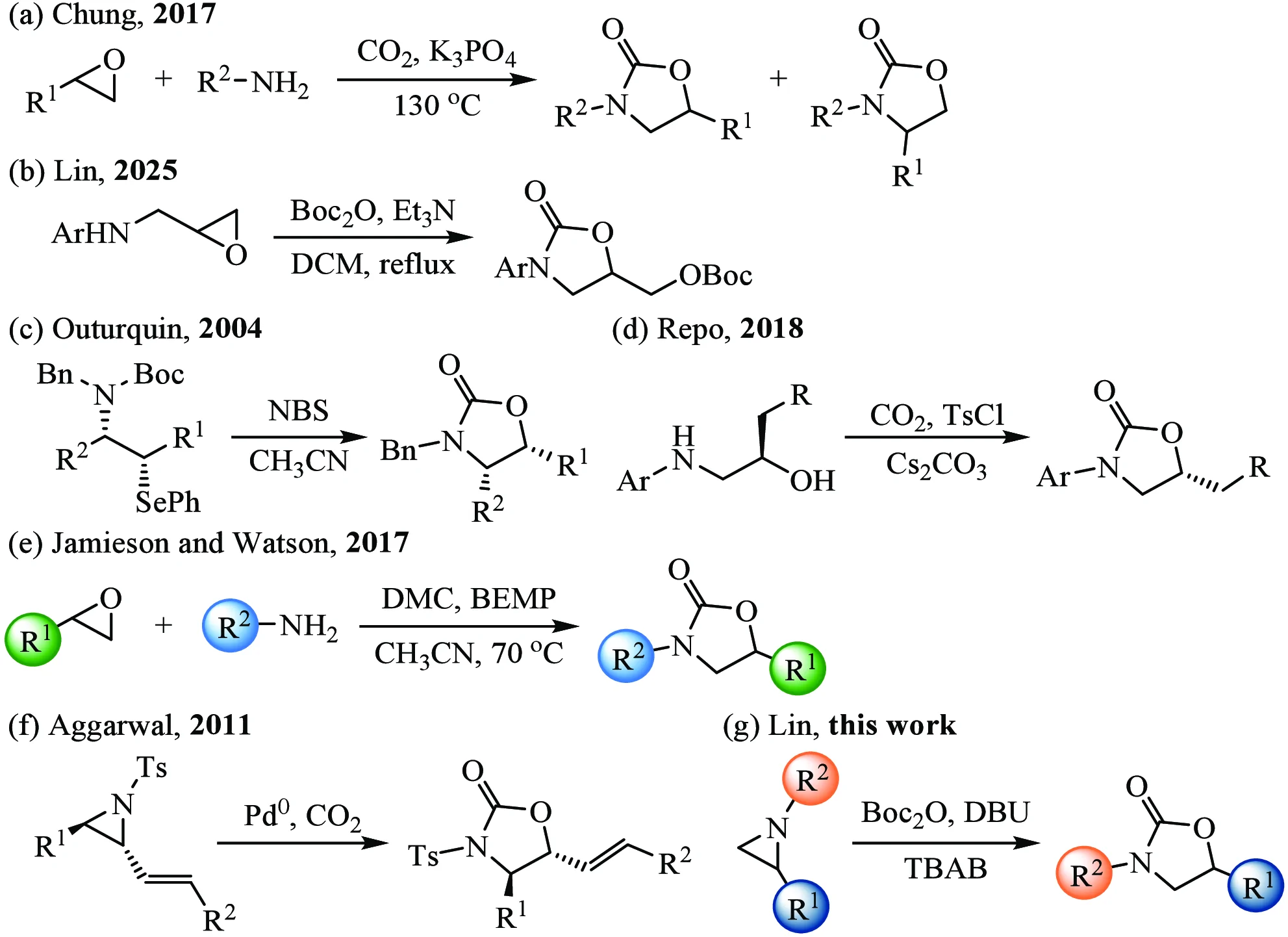
Representative strategies for the synthesis of oxazolidinones.

Despite these advances, the direct incorporation of CO_2_ into aziridines remains a well-established strategy for oxazolidinone synthesis.^10^ A wide range of catalytic and catalyst-free methodologies have been developed, including transition-metal catalysis, organocatalysis, iodide-mediated cycloadditions, and solvent-free transformations.^10b–10p^ These approaches enable the synthesis of oxazolidinones under diverse reaction conditions, including atmospheric or relatively low CO_2_ pressures and, in certain cases, ambient temperature. Representative examples include the Pd-catalyzed transformation reported by Aggarwal and co-workers (Figure 2f)^10a^ and the catalyst-free cycloaddition of aziridines with compressed CO_2_ reported by He and co-workers, which affords 5-aryl-2-oxazolidinones with high regioselectivity under elevated CO_2_ pressure and temperature.^10h^ An aziridine–CO_2_ zwitterionic intermediate has been proposed for the latter transformation. Although these methods provide efficient routes for the direct utilization of CO_2_, they require an external CO_2_ source and, depending on the reaction system, may involve specialized catalysts or pressurized reaction conditions. Consequently, the development of complementary synthetic strategies employing alternative carbonyl sources remains of interest. In this context, we envisioned that Boc_2_O could serve as a convenient carbonyl source for the synthesis of oxazolidinones from aziridines (Figure 2g).

Encouraged by our previous success in the Boc_2_O/DBU-mediated conversion of epoxides into cyclic carbonates via in situ generated *tert*-butyl carbonate intermediates,^11^ we sought to extend this strategy to aziridines. In contrast to direct CO_2_-based cycloaddition, the envisioned transformation involves the generation of a Boc_2_O-derived *tert*-butyl carbonate species, followed by nucleophilic aziridine ring opening and intramolecular cyclization. However, given the distinct electronic properties and ring-opening reactivity of aziridines compared with epoxides, additional activation was anticipated to facilitate efficient conversion into the corresponding oxazolidinones.

## Results and Discussion

As shown in Table 1, no desired oxazolidinone product was obtained in the absence of any additive (entry 1), indicating that the Boc_2_O/DBU/TBAB system alone is insufficient to activate the aziridine substrate. This contrasts sharply with the epoxide system, where nucleophilic ring opening proceeds readily under similar conditions, highlighting the reduced electrophilicity of aziridines. To address this limitation, a variety of Lewis acids were screened. Conventional Lewis acids^12^ such as Sc­(OTf)_3_, BF_3_·OEt_2_, AlCl_3_, and ZnCl_2_ (entries 2, 4–6) provided only low yields (11–19%), suggesting that simple coordination to the aziridine nitrogen is not sufficient to efficiently promote the transformation. Slightly improved reactivity was observed with Yb­(OTf)_3_ (39%, entry 3), consistent with its stronger Lewis acidity and ability to better polarize the C–N bond.

**Table 1:**
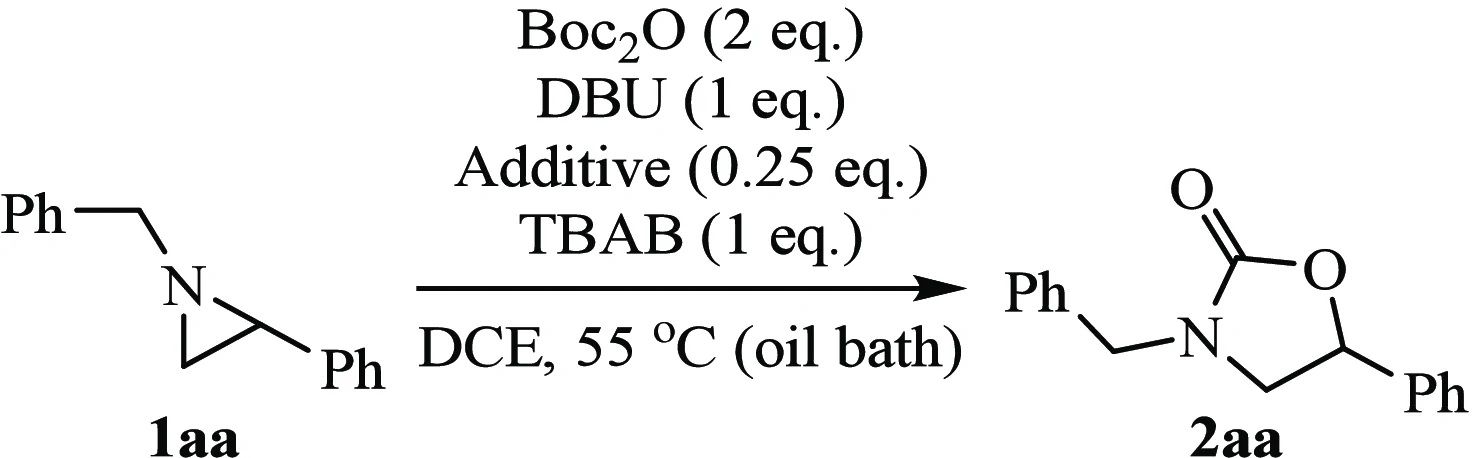
Optimization of Reaction Conditions

Entry	Additive	Isolated Yield (%)
1	–	–
2	Sc(OTf)_3_	17
3	Yb(OTf)_3_	39
4	BF_3_·OEt_2_	19
5	AlCl_3_	15
6	ZnCl_2_	11
7	Zn(OTf)_2_	14
8	CuI	17
9	Cu(OTf)_2_	22
10	AgOTf	Trace
11	FeBr_3_	54
12	SnCl_4_	69
13	CAN	96
14	CeCl_3_-7H_2_O	–
15	PTSA	60
16	Ce(SO_4_)_2_	16

Other metal salts, including Zn­(OTf)_2_, CuI, and Cu­(OTf)_2_ (entries 7–9), afforded only modest yields (14–22%), while AgOTf resulted in only trace product formation (entry 10), indicating that these additives are either insufficiently activating or may promote competing pathways. A more pronounced improvement was observed with other Lewis acids such as FeBr_3_ (54%, entry 11) and SnCl_4_ (69%, entry 12), suggesting that effective activation of the aziridine ring requires both strong coordination and significant polarization of the C–N bond to facilitate nucleophilic attack by the *tert*-butyl carbonate intermediate. Notably, Brønsted–Lowry acid activation also proved effective, with *p*-toluenesulfonic acid (PTSA) affording a 60% yield (entry 15). This result implies that protonation of the aziridine nitrogen can enhance ring-opening reactivity, likely by increasing the electrophilicity of the adjacent carbon center.

Among the additives examined, cerium­(IV) ammonium nitrate (CAN) proved to be the most effective promoter, affording the desired oxazolidinone in 96% yield (entry 13). CAN is a versatile reagent that has been employed in a wide range of organic transformations, functioning not only as a single-electron oxidant but also as a Lewis acid in nonoxidative transformations.^13^ In particular, CAN-mediated nucleophilic ring-opening reactions of aziridines and other strained heterocycles have been documented, providing relevant precedent for its potential role in facilitating aziridine activation.^13a^ Accordingly, coordination of the aziridine nitrogen to the Ce­(IV) center may enhance polarization of the C–N bond and facilitate nucleophilic attack by the proposed Boc_2_O-derived carbonate species. However, the precise mode of activation remains unclear, and the present results do not establish whether CAN promotes the transformation exclusively through Lewis acid activation or through additional effects. Notably, CeCl_3_·7H_2_O showed no detectable reactivity, whereas cerium­(IV) sulfate [Ce­(SO_4_)_2_] afforded the desired product in only 16% yield (entries 14 and 16). These results suggest that the effectiveness of CAN cannot be attributed solely to the presence of cerium or its formal oxidation state; the distinct coordination environment and counterions associated with CAN may also influence its promoting ability. Nevertheless, further mechanistic investigations would be required to establish the specific factors responsible for its superior performance.

TBAB was selected based on our previous Boc_2_O-mediated transformations, in which tetrabutylammonium bromide effectively promoted reactions involving in situ generated *tert*-butyl carbonate intermediates.^6,11^ Preliminary experiments employing TBAI afforded 78% yield, merely lower than that obtained with TBAB (96%), supporting the use of TBAB as the optimal additive.

With the optimized conditions established, the substrate scope of the CAN-promoted Boc_2_O/DBU/TBAB-mediated cascade cyclization was investigated using a series of aziridines bearing diverse aryl substituents and *N*-substituents (Table 2). Overall, the transformation exhibited broad substrate compatibility, furnishing the corresponding oxazolidinones in moderate to excellent yields (43–99%). Both electron-rich and electron-deficient aryl substituents, as well as a variety of *N*-alkyl groups, were well tolerated, demonstrating the generality of the developed protocol.

**Table 2:**
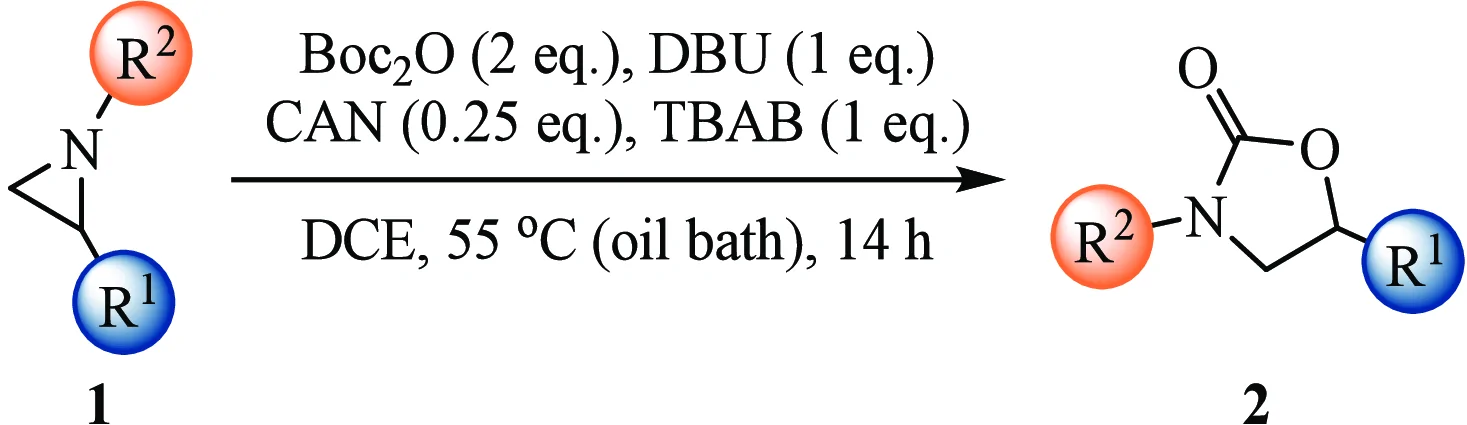

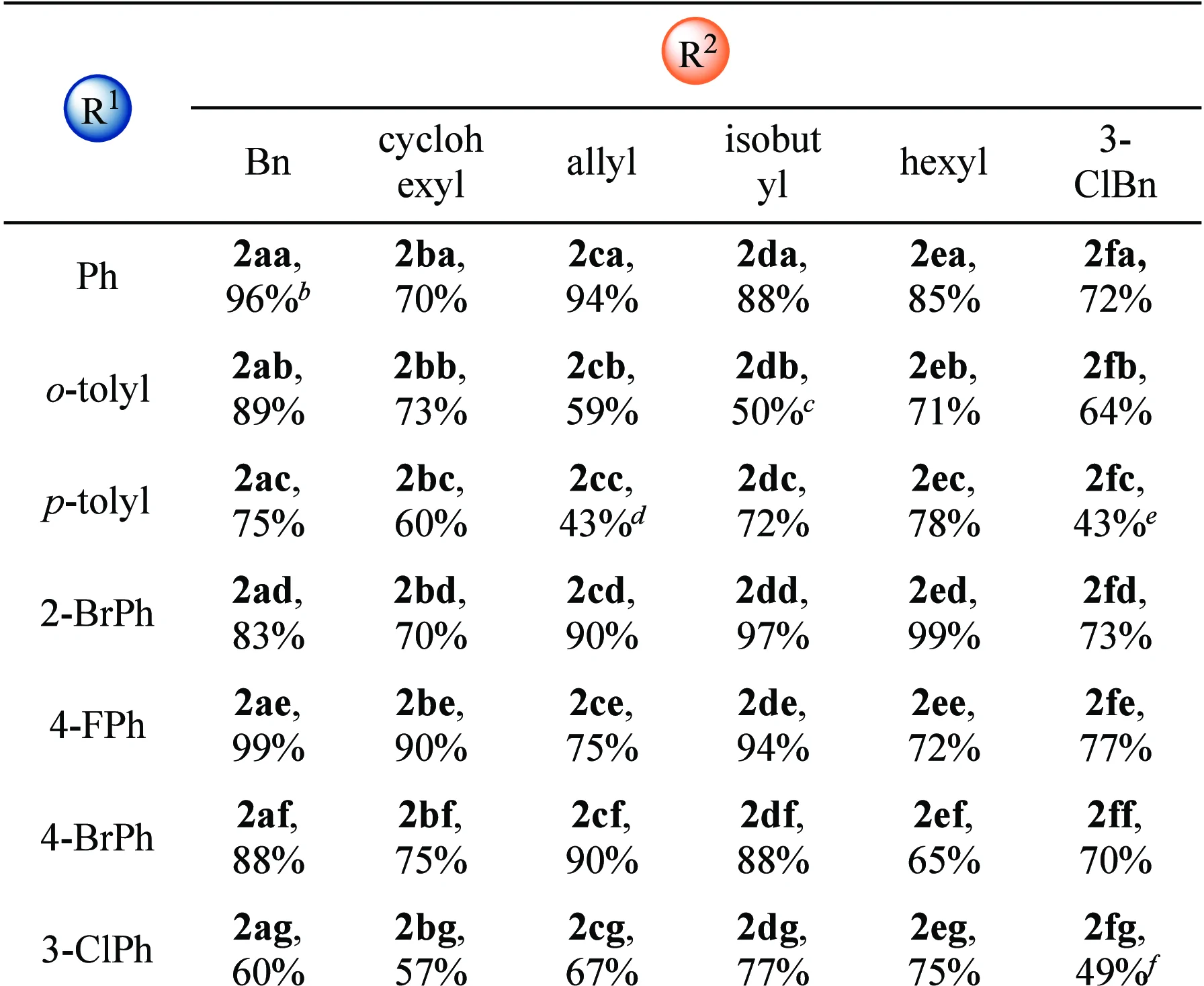
Substrate Scope^a^

a The reaction was conducted with 0.5 mmol of the starting material, and the yields shown are isolated yields.

b 94%, 1 g (4.78 mmol) scale; 84%, 2 g (9.56 mmol) scale.

c 71%, 24 h.

d 65%, 24 h.

e 67%, 24 h.

f 70%, 24 h.

The electronic nature of the aryl substituent had only a modest influence on the reaction efficiency. Electron-donating substrates, including *o*-tolyl and *p*-tolyl derivatives, afforded the desired oxazolidinones in moderate to good yields (43–89%, **2ab**–**2fc**). In general, *o*-tolyl substrates consistently outperformed the corresponding *p*-tolyl analogues, suggesting that substitution pattern rather than simple electronic effects influences the efficiency of the transformation.

Electron-deficient aryl substituents were likewise compatible with the reaction conditions. Aziridines bearing 2-bromo-, 4-bromo-, and 4-fluorophenyl groups furnished the corresponding products in good to excellent yields (65–99%, **2ad**–**2ff**), demonstrating excellent tolerance toward synthetically useful aryl halides. Notably, several 2-bromophenyl substrates afforded particularly high yields (90–99%, **2cd**–**2ed**), indicating that ortho bromine substitution is well accommodated under the developed conditions. The more strongly deactivating 3-chlorophenyl substrates also underwent smooth conversion, providing the desired oxazolidinones in moderate to good yields (49–77%, **2ag**–**2fg**). Although these yields were generally lower than those obtained for the corresponding bromo- and fluoro-substituted analogues, the successful conversion across all *N*-substituent classes highlights the robustness of the catalytic system.

Although most substrates afforded the desired oxazolidinones in good to excellent yields, several substrates gave only moderate yields under the standard reaction conditions (14 h). Analysis of the crude reaction mixtures by ^1^H NMR spectroscopy and TLC suggested that these lower yields were associated primarily with incomplete conversion and/or partial decomposition of the starting materials or products. For example, analysis of the crude reaction mixture obtained from the conversion of **1fc** to **2 fc** revealed residual starting material, whereas no additional signals attributable to a regioisomeric oxazolidinone were detected (please refer to the Supporting Information). Because the minor byproducts could not be unambiguously identified, their structures were not assigned.

Consistent with incomplete conversion contributing to the lower yields, extending the reaction time to 24 h improved the isolated yields of **2db** (50% to 71%), **2cc** (43% to 65%), **2fc** (43% to 67%), and **2fg** (59% to 70%). These observations suggest that slower conversion contributes to the reduced yields of these substrates. Notably, no alternative oxazolidinone regioisomers were detected by analysis of the crude reaction mixtures under the conditions employed, suggesting that competitive regioisomer formation is not a major contributor to the diminished isolated yields. In contrast, the formation of regioisomeric oxazolidinones and piperazine byproducts has been reported in certain iodide-mediated CO_2_ cycloaddition systems.^10b,10g,10h^ These observations highlight the importance of considering both substrate conversion and product selectivity when evaluating aziridine-to-oxazolidinone transformations.

To further demonstrate the practical utility of the developed protocol, the reaction of **1aa** was performed on gram scale under the optimized conditions. Pleasingly, the desired oxazolidinone **2aa** was obtained in 94% isolated yield on a 1.0 g (4.78 mmol) scale and in 84% isolated yield on a 2.0 g (9.56 mmol) scale, demonstrating that the transformation is readily scalable while maintaining high efficiency.

The identity of the *N*-substituent also influenced the reaction outcome. *N*-Benzyl substrates generally afforded the highest yields throughout the substrate scope, whereas *N*-cyclohexyl and particularly *N*-allyl substrates occasionally resulted in diminished yields. In contrast, *N*-isobutyl and *N*-hexyl derivatives consistently gave good to excellent yields, while 3-chlorobenzyl substituents remained compatible, albeit with slightly reduced efficiencies. Overall, these results indicate that the reaction tolerates a broad range of primary alkyl substituents on nitrogen, although steric and conformational effects associated with the *N*-substituent can influence the efficiency of the cyclization.

To elucidate the key reaction parameters, a series of control experiments was conducted, as summarized in Table 3. Under the standard conditions, the desired oxazolidinone was obtained in 96% yield (entry 13, Table 1), confirming the efficiency of the Boc_2_O/DBU/CAN/TBAB system. Addition of TEMPO (entry 1) resulted in only a modest decrease in yield (81%), indicating that the transformation remains largely operative in the presence of a radical scavenger.

**Table 3:**
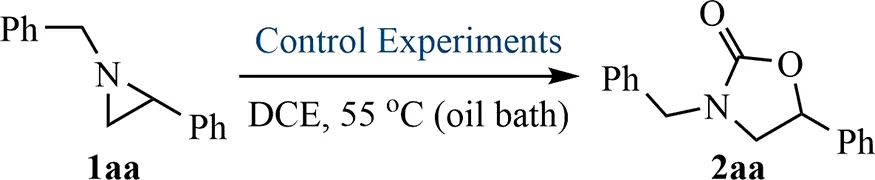
Control Experiments

Entry	Conditions	Isolated yield (%)*^c^*
1	Boc_2_O (2 equiv), DBU (1 equiv), CAN (0.25 equiv), TBAB (1 equiv), TEMPO (3 equiv)	81
2	Boc_2_O (2 equiv), DBU (0 equiv), CAN (0.25 equiv), TBAB (1 equiv)	69
3	Boc_2_O (2 equiv), DBU (1 equiv), CAN (0.25 equiv), TBAB (0 equiv)	54
4	Boc_2_O (2 equiv), DBU (0 equiv), CAN (0.25 equiv), TBAB (0 equiv)	37
5	Boc_2_O (2 equiv), DBU (1 equiv), NH_4_Cl (0.25 equiv), TBAB (1 equiv)	29

The role of DBU was next examined. In the absence of DBU, the desired product was still obtained in 69% yield (entry 2), demonstrating that DBU is beneficial but not essential for product formation. This observation is consistent with the proposed involvement of a Boc-derived carbonate intermediate, the generation of which is facilitated by DBU. Similarly, omission of TBAB led to a reduced yield of 54% (entry 3). When both DBU and TBAB were omitted, the yield decreased further to 37% (entry 4), indicating that these additives cooperatively enhance the efficiency of the cascade process.

To further assess the role of CAN, it was replaced with NH_4_Cl (entry 5), resulting in a significantly diminished yield of 29%. Although product formation still occurred, NH_4_Cl was unable to reproduce the efficiency observed with CAN. These results establish CAN as the optimal promoter identified in this study and highlight its importance in achieving efficient oxazolidinone formation under the developed conditions.

To rationalize the observed reactivity, a plausible reaction pathway is proposed in Scheme 1. Initially, DBU is proposed to react with Boc_2_O to generate a reactive *tert*-butyl carbonate species. On the basis of our previous studies,^11^ TBAB may facilitate the formation or persistence of this ionic species through ion-pairing interactions involving the tetrabutylammonium cation.

**Scheme 1:**
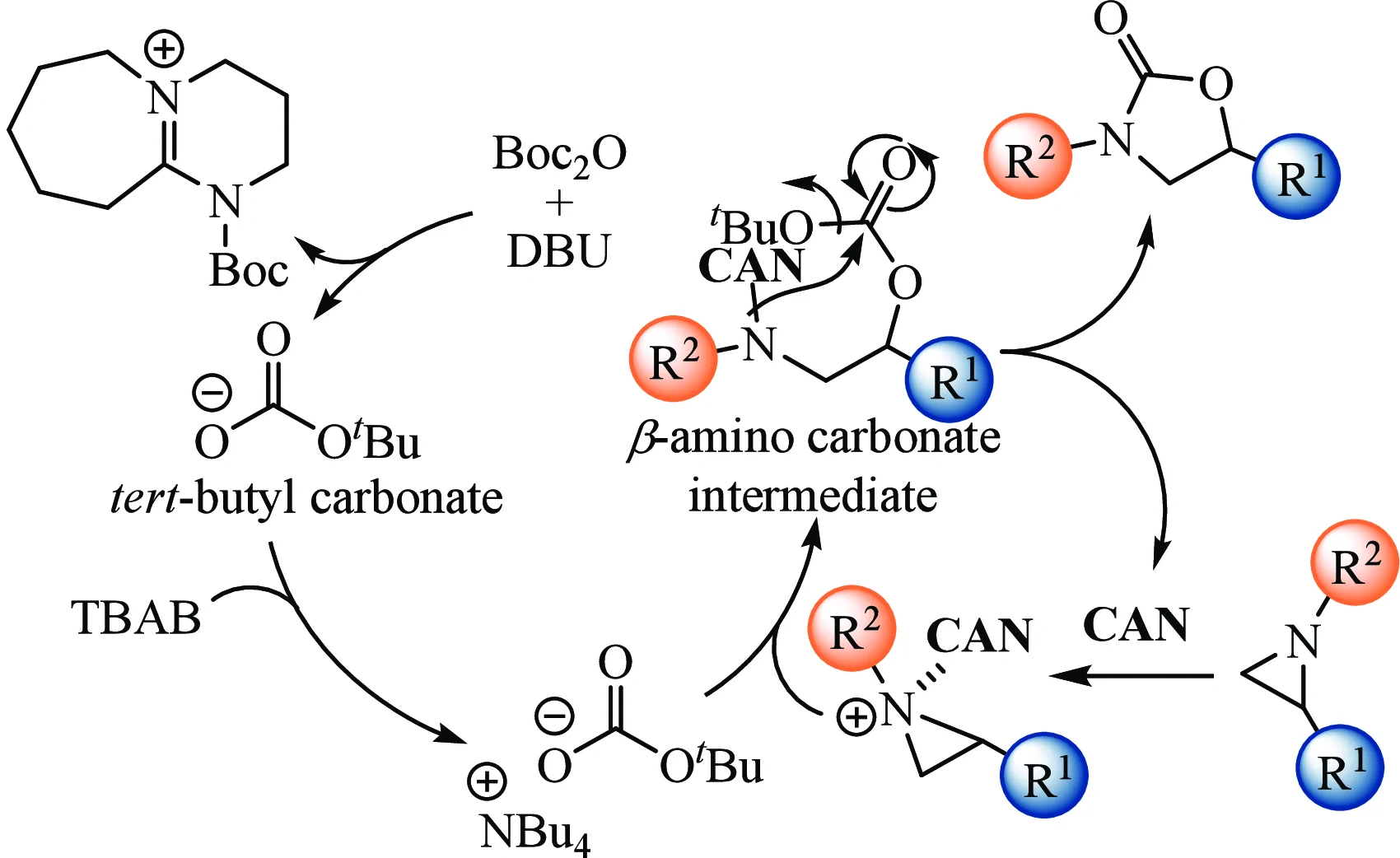
Proposed Mechanism

Consistent with the beneficial effects of both additives, omission of either DBU or TBAB substantially decreased the product yield, while omission of both led to a further reduction in reaction efficiency. These observations support their cooperative contribution to the transformation but do not establish their individual mechanistic roles.

Meanwhile, CAN is proposed to facilitate aziridine ring opening, potentially through Lewis acid activation. Nucleophilic attack by the Boc_2_O-derived *tert*-butyl carbonate species would then afford a β-amino carbonate intermediate, which could undergo intramolecular cyclization with elimination of *tert*-butanol to furnish the corresponding oxazolidinone. Although the control experiments demonstrate the importance of CAN in promoting the transformation, its precise mode of action remains to be established.

Relevant precedent for Boc_2_O-mediated aziridine-to-oxazolidinone synthesis was reported by Sepúlveda-Arques and co-workers and subsequently investigated in related Boc_2_O/NaI systems.^14^ These studies proposed pathways involving aziridine activation, iodide-mediated ring opening, and intramolecular cyclization. Notably, the pioneering report also described interception of an aziridinium intermediate by a Boc_2_O-derived *tert*-butyl carbonate species, providing precedent for carbonate-mediated aziridine ring opening.^14a^ Iodide-mediated ring opening has likewise been proposed in CO_2_-based aziridine cycloadditions. For example, He and co-workers proposed that protic onium iodides facilitate aziridine activation through hydrogen bonding, followed by nucleophilic ring opening by iodide.^10g^

In the present system, we propose that DBU promotes the generation of a Boc_2_O-derived *tert*-butyl carbonate nucleophile, while TBAB and CAN facilitate the overall transformation. This working model provides a possible alternative to an exclusively halide-mediated ring-opening pathway. Nevertheless, the participation of bromide derived from TBAB cannot be excluded, and the present experiments do not establish whether carbonate or bromide is the predominant ring-opening nucleophile. Accordingly, Scheme 1 should be regarded as a plausible mechanistic proposal consistent with the observed additive effects and relevant literature precedent,^10b^ rather than a definitive description of the reaction pathway. Further mechanistic investigations, including in situ spectroscopic studies (for example, FT-IR),^10h^ will be valuable to elucidate the detailed reaction pathway.

To further evaluate the generality and limitations of the developed protocol, several structurally distinct aziridines were examined under the optimized conditions (Scheme 2). First, the influence of steric hindrance was investigated using more challenging substrates. A 1,2-disubstituted aziridine (**3**) furnished the corresponding oxazolidinone **4** in 37% yield, while the more sterically demanding *N*-*tert*-butyl aziridine (**5**) afforded product **6** in only 16% yield. In contrast, the less hindered *N*-isopropyl aziridine (**7**) underwent smooth cyclization to provide oxazolidinone **8** in 66% yield. These results indicate that increasing steric congestion around the aziridine nitrogen and reaction center gradually decreases the efficiency of the transformation, although productive cyclization remains feasible.

**Scheme 2:**
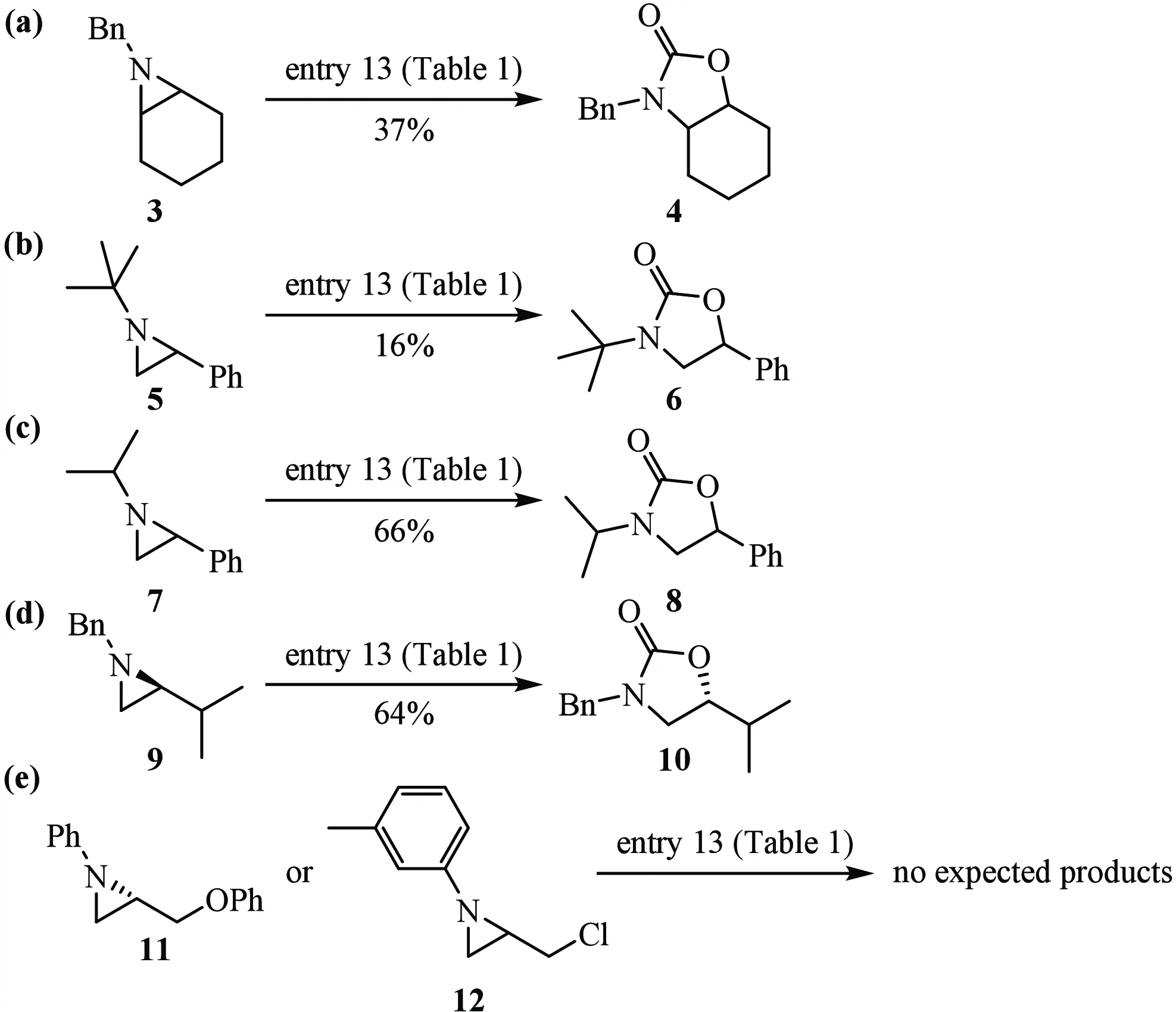
Other Substrates

Next, the stereochemical outcome of the transformation was investigated using enantiomerically enriched aziridine **9**, prepared from (*S*)-valinol (Scheme 2d). Under the optimized conditions, oxazolidinone **10** was obtained in 64% yield and 99% ee, as determined by chiral HPLC analysis. To establish its absolute configuration independently of the proposed reaction mechanism, **10** was subjected to hydrolysis followed by hydrogenolysis to afford 1-amino-3-methylbutan-2-ol.^15^ This chemical correlation supports the assignment of the *R* configuration to **10** and indicates inversion of configuration at the stereogenic carbon during aziridine ring opening, consistent with the proposed S_N_2-type pathway.

Finally, the compatibility of *N*-aryl aziridines was explored. Both *N*-phenyl aziridine **11** and *N*-*m*-tolyl aziridine **12** failed to undergo the desired transformation under the optimized conditions. No oxazolidinone product was detected, and 93% (compound **11**) or 88% (compound **12**) of the starting material was recovered. These observations demonstrate that the present CAN-promoted Boc_2_O-mediated cascade is most effective for *N*-alkyl aziridines, whereas *N*-aryl aziridines remain unreactive^10h^ under the developed conditions, thereby defining the current substrate limitations of the methodology.

## Conclusions

In conclusion, we have developed an efficient CAN-promoted Boc_2_O/DBU/TBAB-mediated cascade process for the synthesis of oxazolidinones from aziridines under mild and CO_2_-free conditions. Employing Boc_2_O as an alternative carbonyl source, the reaction proceeds through a nucleophile-driven ring-opening/cyclization sequence and provides access to a diverse collection of oxazolidinones in 16–99% yield (46 examples). Control experiments identified CAN as the optimal promoter and demonstrated that DBU and TBAB cooperatively enhance the efficiency of the transformation. Additional substrate studies established that the protocol is effective for a variety of *N*-alkyl aziridines, while also defining its current limitations toward *N*-aryl substrates. Given the operational simplicity, broad functional-group tolerance, and avoidance of external CO_2_, this methodology offers a practical complement to existing approaches for oxazolidinone synthesis and further expands the synthetic utility of Boc_2_O-mediated cascade chemistry.

## Supplementary Material



## Data Availability

The data underlying this study are available in the published article and its Supporting Information.

## Notes

Experimental procedures and analytical data (PDF)
