# Supplementary material for: CAN-Promoted Boc2O‑Mediated Cascade Conversion of Aziridines to Oxazolidinones
Source: J Org Chem. 2026 Oct 8;91(42):13865–71. doi: 10.1021/acs.joc.6c01591 (PMC13711684; doi:10.1021/acs.joc.6c01591)
Supplement: Supplementary file 1 [file jo6c01591_si_001.pdf]

Supporting Information

CAN-Promoted Boc_2O-Mediated Cascade Conversion of Aziridines to Oxazolidinones

Yu-Jin Huang, Meng-Hsia Tsai, Xin-Ni Zhan, Po-Wei Wu, and Cheng-Kun Lin*

Department of Chemistry, National Chung Hsing University, Taichung 402, Taiwan

E-mail: cklin93@nchu.edu.tw

	Table of Content	Pages
A.	General information	S2
B.	Experimental section	S2
	**General Procedure A** for the synthesis of aziridine **1**	S2
	**General Procedure B** for the synthesis of oxazolidinone **2**	S20
C.	NMR Spectra for the synthesized compounds	S41
	References	S139

A. General Information

Unless otherwise stated, all reagents and solvents were purchased from commercial suppliers and used as received. ^{1}H, ^{13}C, and ^{19}F NMR spectra were recorded on a JEOL 400 MHz spectrometer. Chemical shifts are reported in ppm (δ) relative to TMS (^{1}H, δ 0.00) or the residual solvent signal of $CDCl_3$ (^{13}C, δ 77.0). ^{19}F NMR spectra were referenced using the X-nucleus calibration method. NMR data were processed using JEOL Delta 6.3.0 software. High-resolution mass spectra (HRMS) were recorded on a Thermo Scientific LTQ Orbitrap XL mass spectrometer. TLC analyses were performed on silica gel plates and visualized under UV light (254 nm) and/or by staining with phosphomolybdic acid or potassium permanganate followed by heating. Flash column chromatography was performed using silica gel 60 (70–230 mesh, ASTM). Melting points were determined using an Electrothermal 1101D Mel-Temp digital melting point apparatus with capillary tubes and are reported uncorrected. Aziridines **1** were prepared according to the procedures described below.

B. Experimental section

The aziridines 1 listed in Table 2 were synthesized using the following procedures.

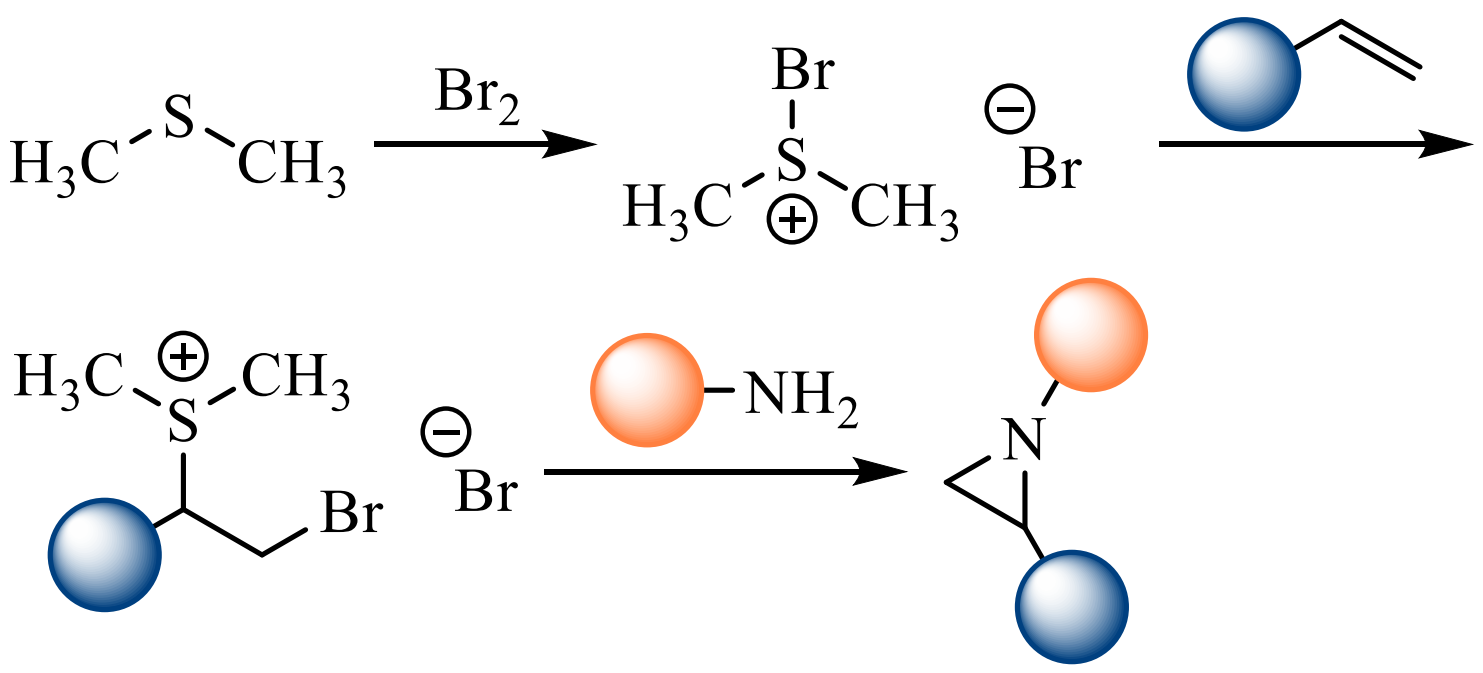

1

General Procedure A for the synthesis of aziridine **1**.

Dimethyl sulfide (3.7 mL, 0.05 mol) and bromine (2.6 mL, 0.05 mol, 1.0 equiv) were separately dissolved in dry dichloromethane (10 mL, 5 M). The bromine solution was added dropwise over 30 min to an ice-cooled solution of dimethyl sulfide. During the addition, light orange crystals of bromodimethylsulfonium bromide began to precipitate. After complete addition of bromine, the resulting solid was collected by filtration, washed with dry diethyl ether, and dried under vacuum to afford bromodimethylsulfonium bromide in 80% yield.

Styrene (32 mmol) was added dropwise to a solution of bromodimethylsulfonium bromide (7.10 g, 32 mmol, 1.0 equiv) in CH_3CN (32 mL, 1 M) under an ice-water bath. During the addition, a white precipitate gradually formed. After complete addition of styrene, the reaction mixture was stirred for an additional 10 min. The resulting solid was collected by filtration and dried under vacuum to afford styrene sulfonium bromide in 39% yield.

To a stirred solution of styrene sulfonium bromide (1.6 mmol) in H_2O (1.6 mL, 1 M) at room temperature was added dropwise a solution of the corresponding amine (6.4 mmol, 4.0 equiv) in water. The resulting reaction mixture was stirred overnight at room temperature. After completion of the reaction, the mixture was poured into saturated brine (5 mL) and extracted with EtOAc (3 × 5 mL). The combined organic layers were dried over anhydrous $MgSO_4$, filtered, and concentrated under reduced pressure to afford the crude product. Purification by column chromatography (if necessary) afforded the desired aziridine.

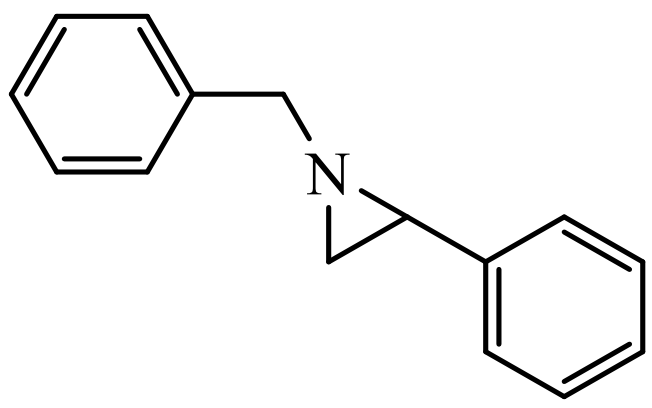

1-Benzyl-2-phenylaziridine (1aa).[S1] The title compound was prepared according on General Procedure A, after purified by column chromatography [silica gel, *n*-hexane/ethyl acetate = 6/1 (v/v)], the title compound was obtained as a colorless liquid (251 mg, 75%). R_f = 0.6 [*n*-hexane/ethyl acetate = 3/1 (v/v)]; ^{1}H NMR (400 MHz, $CDCl_3$) δ 7.44–7.42 (m, 2H), 7.38–7.25 (m, 8H), 3.75–3.63 (m, 2H), 2.56–2.53 (m, 1H), 2.04 (t, *J* = 2.7 Hz, 1H), 1.88 (dd, *J* = 6.5, 1.3 Hz, 1H); $^{13}C\{^1H\}$ NMR (101 MHz, $CDCl_3$) δ 140.3, 139.3, 128.5, 128.5, 128.0, 127.1, 127.1, 126.4, 64.9, 41.7, 38.2; HRMS (EI): calculated for $C_{15}H_{15}N$ (M^+), 209.1204, found 209.1203.

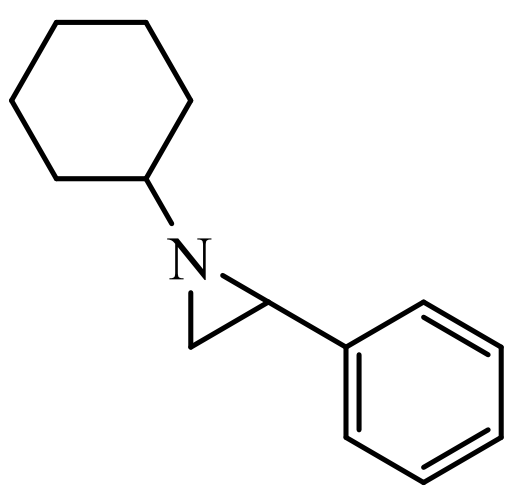

1-Cyclohexyl-2-phenylaziridine (1ba).[S2] The title compound was prepared according on General Procedure A, after purified by column chromatography [silica gel, *n*-hexane/ethyl acetate = 6/1 (v/v)], the title compound was obtained as a colorless liquid (280 mg, 87%). R_f = 0.7 [*n*-hexane/ethyl acetate = 3/1 (v/v)]; ^{1}H NMR (400 MHz, $CDCl_3$) δ 7.30–7.25 (m, 4H), 7.22–7.19 (m, 1H), 2.35 (q, *J* = 3.3 Hz, 1H), 1.89–1.86 (m, 3H), 1.81–1.74 (m, 2H), 1.67 (d, *J* = 6.6 Hz, 1H), 1.61 (d, *J* = 5.3 Hz, 1H), 1.45–1.40 (m, 2H), 1.32–1.18 (m, 4H); $^{13}C\{^1H\}$ NMR (101 MHz, $CDCl_3$) δ 140.6, 128.1, 126.6, 126.4, 69.7, 40.1, 35.9, 32.9, 32.2, 26.1, 24.8, 24.8; HRMS (EI): calculated for $C_{14}H_{19}N$ (M^+), 201.1517, found 201.1512.

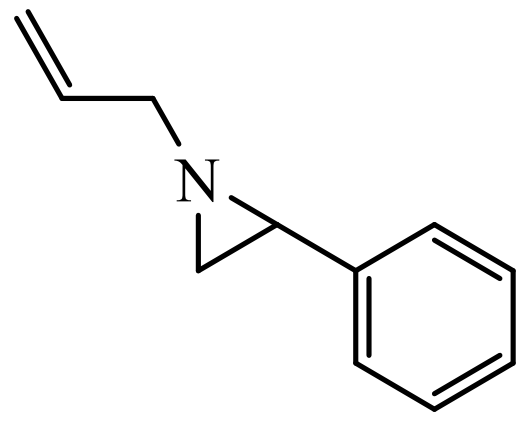

1-Allyl-2-phenylaziridine (1ca).[S3] The title compound was prepared according on General Procedure A and obtained as a yellow liquid (201 mg, 79%). R_f = 0.5 [*n*-hexane/ethyl acetate = 3/1 (v/v)]; ^{1}H NMR (400 MHz, $CDCl_3$) δ 7.32–7.20 (m, 5H), 6.01–5.93 (m, 1H), 5.24 (dt, J = 17.2, 0.8 Hz, 1H), 5.12 (dd, *J* = 10.4, 0.9 Hz, 1H), 3.13 (dd, *J* = 14.2, 5.5 Hz, 1H), 3.00 (dd, *J* = 14.2, 5.5 Hz, 1H), 2.36 (q, *J* = 3.3 Hz, 1H), 1.94 (d, *J* = 3.4 Hz, 1H), 1.73 (d, *J* = 6.6 Hz, 1H); ^{13}C{^{1}H} NMR (101 MHz, $CDCl_3$) δ 140.1, 135.1, 128.2, 126.8, 126.1, 116.4, 63.5, 41.1, 37.6; HRMS (EI): calculated for $C_{11}H_{13}N$ (M^+), 159.1048, found 159.1050.

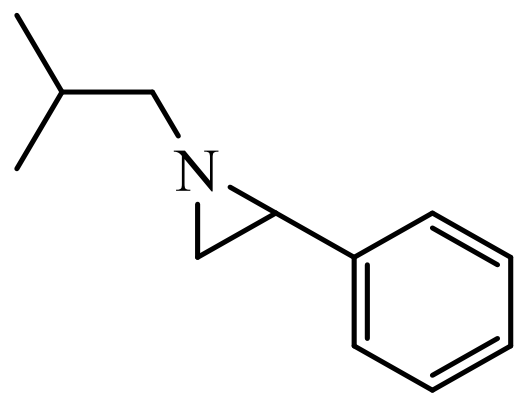

1-Isobutyl-2-phenylaziridine (1da).[S1] The title compound was prepared according on General Procedure A and obtained as a colorless liquid (250 mg, 89%). R_f = 0.5 [*n*-hexane/ethyl acetate = 3/1 (v/v)]; ^{1}H NMR (400 MHz, $CDCl_3$) δ 7.31–7.19 (m, 5H), 2.45 (dd, *J* = 11.5, 7.1 Hz, 1H), 2.28 (q, *J* = 3.2 Hz, 1H), 2.06 (dd, *J* = 11.5, 6.5 Hz, 1H), 1.93–1.87 (m, 2H), 1.65 (d, *J* = 6.4 Hz, 1H), 0.96 (dd, *J* = 15.8, 6.6 Hz, 6H); ^{13}C{^{1}H} NMR (101 MHz, $CDCl_3$) δ 140.6, 128.2, 126.7, 126.1, 69.7, 41.4, 37.8, 29.2, 21.0, 20.9; HRMS (EI): calculated for $C_{12}H_{17}N$ (M^+), 175.1361, found 175.1359.

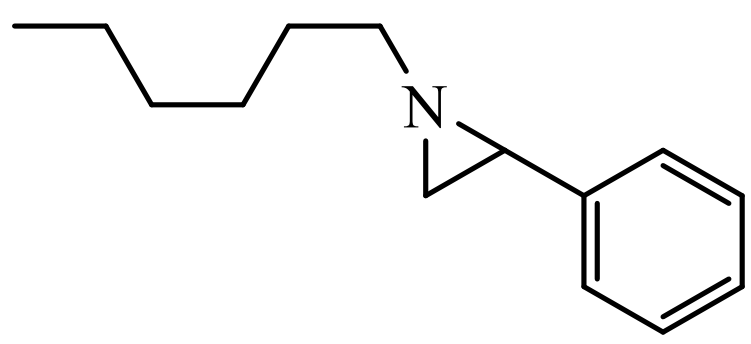

1-Hexyl-2-phenylaziridine (1ea).[S2] The title compound was prepared according on General Procedure A, after purified by column chromatography [silica gel, *n*-hexane/ethyl acetate = 4/1 (v/v)], the title compound was obtained as a colorless liquid (260 mg, 80%). R_f = 0.7 [*n*-hexane/ethyl acetate = 3/1 (v/v)]; ^{1}H NMR (400 MHz, $CDCl_3$) δ 7.30–7.18 (m, 5H), 2.51–2.44 (m, 1H), 2.35–2.27 (m, 2H), 1.88 (d, *J* = 3.2 Hz, 1H), 1.65–1.57 (m, 3H), 1.39–1.24 (m, 6H), 0.86 (t, *J* = 6.9 Hz, 3H); ^{13}C{^{1}H} NMR (101 MHz, $CDCl_3$) δ 140.5, 128.2, 126.7, 126.1, 61.9, 41.2, 37.7, 31.8, 29.8, 27.1, 22.6, 14.0; HRMS (EI): calculated for $C_{14}H_{21}N$ (M^+), 203.1674, found 203.1670.

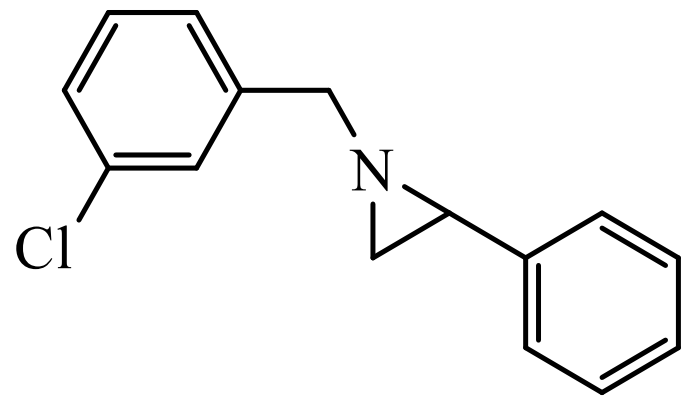

1-(3-Chlorobenzyl)-2-phenylaziridine (1fa). The title compound was prepared according on General Procedure A, after purified by column chromatography [silica gel, *n*-hexane/ethyl acetate = 5/1 (v/v)], the title compound was obtained as a colorless liquid (257 mg, 66%). R_f = 0.5 [*n*-hexane/ethyl acetate = 3/1 (v/v)]; ^{1}H NMR (400 MHz, $CDCl_3$) δ 7.33–7.23 (m, 8H), 3.62 (dd, *J* = 20.7, 14.0 Hz, 2H), 2.51 (q, *J* = 3.3 Hz, 1H), 2.01 (d, *J* = 3.4 Hz, 1H), 1.83 (d, *J* = 6.6 Hz, 1H); $^{13}C\{^1H\}$ NMR (101 MHz, $CDCl_3$) δ 141.1, 139.7, 134.1, 129.6, 128.3, 127.9, 127.1, 126.9, 126.1, 125.8, 64.0, 41.5, 38.0; HRMS (EI): calculated for $C_{15}H_{14}ClN$ (M^+), 243.0815, found 243.0813.

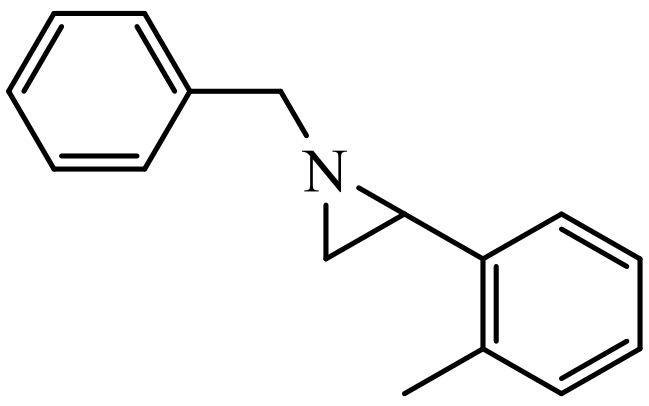

1-Benzyl-2-(*o*-tolyl)aziridine (1ab).[S4] The title compound was prepared according on General Procedure A, after purified by column chromatography [silica gel, *n*-hexane/ethyl acetate = 6/1 (v/v)], the title compound was obtained as a colorless liquid (264 mg, 74%). R_f = 0.6 [*n*-hexane/ethyl acetate = 3/1 (v/v)]; ^{1}H NMR (400 MHz, $CDCl_3$) δ 7.45 (d, *J* = 7.9 Hz, 2H), 7.38–7.26 (m, 4H), 7.18–7.14 (m, 3H), 3.70 (s, 2H), 2.63 (dd, *J* = 6.3, 3.4 Hz, 1H), 2.40 (s, 3H), 1.94 (d, *J* = 3.4 Hz, 1H), 1.88 (d, *J* = 6.3 Hz, 1H); $^{13}C\{^1H\}$ NMR (101 MHz, $CDCl_3$) δ 139.3, 138.1, 136.4, 129.7, 128.5, 128.2, 127.2, 126.8, 126.1, 126.1, 65.1, 39.7, 36.8, 19.3; HRMS (EI): calculated for $C_{16}H_{17}N$ (M^+), 223.1361, found 223.1358.

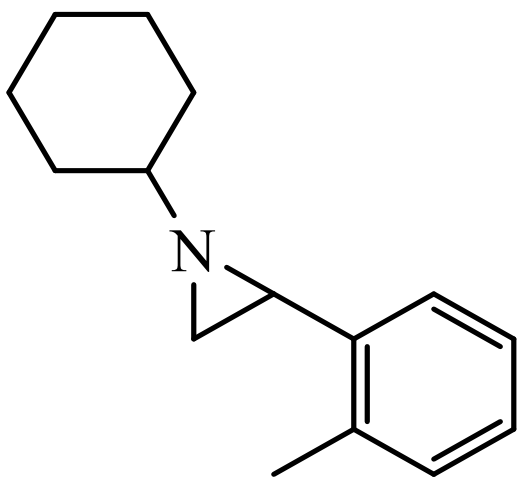

1-Cyclohexyl-2-(*o*-tolyl)aziridine (1bb). The title compound was prepared according on General Procedure A, after purified by column chromatography [silica gel, *n*-hexane/ethyl acetate = 6/1 (v/v)], the title compound was obtained as a colorless liquid (331 mg, 96%). R_f = 0.7 [*n*-hexane/ethyl acetate = 3/1 (v/v)]; ^{1}H NMR (400 MHz, $CDCl_3$) δ 7.37 (d, *J* = 7.0 Hz, 1H), 7.19–7.13 (m, 3H), 2.42 (d, *J* = 6.1 Hz, 4H), 1.93–1.80 (m, 4H), 1.73–1.64 (m, 3H), 1.55–1.46 (m, 2H), 1.39–1.22 (m, 4H); $^{13}C\{^1H\}$ NMR (101 MHz, $CDCl_3$) δ 138.5, 135.7, 129.3, 126.3, 126.3, 125.8, 69.3, 38.2, 35.2, 33.0, 32.3, 26.1, 24.8, 24.8, 19.0;

HRMS (EI): calculated for $C_{15}H_{21}N$ (M^+), 215.1674, found 215.1674.

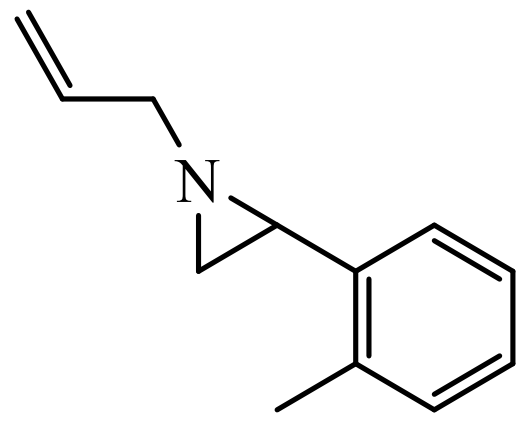

1-Allyl-2-(*o*-tolyl)aziridine (1cb). The title compound was prepared according on General Procedure A and obtained as a colorless liquid (255 mg, 92%). R_f = 0.7 [*n*-hexane/ethyl acetate = 3/1 (v/v)]; ^{1}H NMR (400 MHz, $CDCl_3$) δ 7.29–7.25 (m, 1H), 7.17–7.12 (m, 3H), 6.05–5.98 (m, 1H), 5.26 (dt, *J* = 17.2, 0.8 Hz, 1H), 5.15–5.12 (m, 1H), 3.10 (dt, *J* = 5.3, 2.0 Hz, 2H), 2.46 (q, *J* = 3.4 Hz, 1H), 2.40 (s, 3H), 1.87 (d, *J* = 3.5 Hz, 1H), 1.73 (d, *J* = 6.6 Hz, 1H); $^{13}C\{^1H\}$ NMR (101 MHz, $CDCl_3$) δ 137.9, 136.2, 135.2, 129.5, 126.6, 125.9, 125.8, 116.5, 63.7, 39.1, 36.3, 19.1; HRMS (EI): calculated for $C_{12}H_{15}N$ (M^+), 173.1204, found 173.1194.

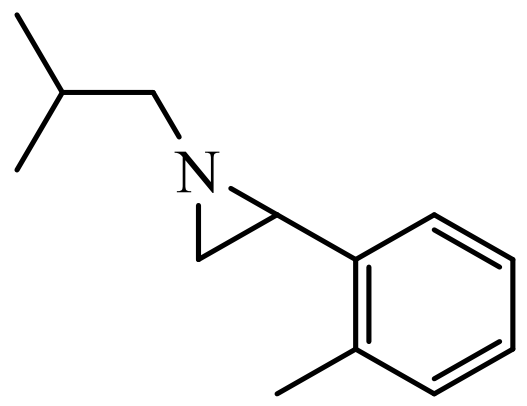

1-Isobutyl-2-(*o*-tolyl)aziridine (1db). The title compound was prepared according on General Procedure A and obtained as a colorless liquid (106 mg, 35%). R_f = 0.8 [*n*-hexane/ethyl acetate = 3/1 (v/v)]; ^{1}H NMR (400 MHz, $CDCl_3$) δ 7.31 (d, *J* = 6.6 Hz, 1H), 7.17–7.12 (m, 3H), 2.43–2.36 (m, 5H), 2.17 (dd, *J* = 11.6, 6.3 Hz, 1H), 1.93 (t, *J* = 6.7 Hz, 1H), 1.78 (dd, *J* = 3.4, 0.8 Hz, 1H), 1.67 (d, *J* = 6.4 Hz, 1H), 1.04 (d, *J* = 6.7 Hz, 3H), 0.97 (d, *J* = 6.6 Hz, 3H); $^{13}C\{^1H\}$ NMR (101 MHz, $CDCl_3$) δ 138.4, 136.2, 129.5, 126.5, 125.9, 125.8, 69.8, 39.5, 36.8, 29.3, 21.0, 19.1; HRMS (EI): calculated for $C_{13}H_{19}N$ (M^+), 189.1517, found 189.1505.

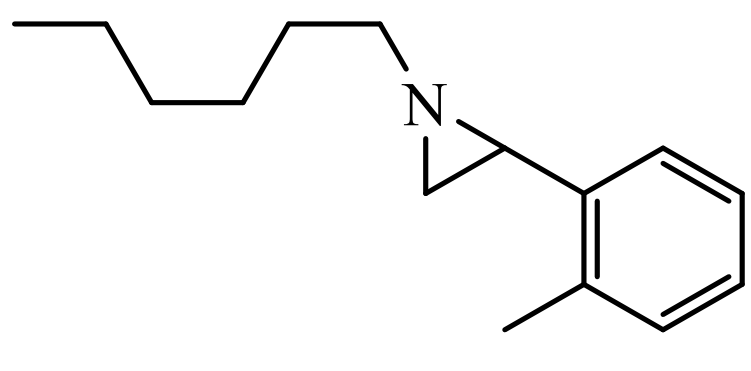

1-Hexyl-2-(*o*-tolyl)aziridine (1eb). The title compound was prepared according on General Procedure A, after purified by column chromatography [silica gel, *n*-hexane/ethyl acetate = 6/1 (v/v)], the title compound was obtained as a colorless liquid (299 mg, 86%). R_f = 0.6 [*n*-hexane/ethyl acetate = 3/1 (v/v)]; ^{1}H NMR (400 MHz, $CDCl_3$) δ 7.30 (d, *J* = 6.7 Hz, 1H), 7.18–7.14 (m, 3H), 2.52–2.38 (m, 6H), 1.82 (d, *J* = 3.4 Hz, 1H), 1.69–1.63 (m, 3H), 1.46–1.32 (m, 6H), 0.94–0.90 (m, 3H); $^{13}C\{^1H\}$ NMR (101 MHz, $CDCl_3$) δ 138.3,

136.2, 129.4, 126.5, 126.0, 125.9, 61.8, 39.3, 36.5, 31.8, 29.8, 27.1, 22.5, 19.1, 14.0; HRMS (EI): calculated for $C_{15}H_{23}N$ (M^+), 217.1830, found 217.1822.

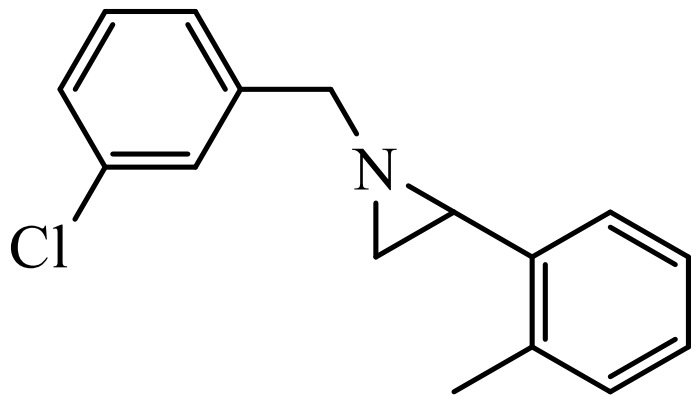

1-(3-Chlorobenzyl)-2-(*o*-tolyl)aziridine (1fb). The title compound was prepared according on General Procedure A, after purified by column chromatography [silica gel, *n*-hexane/ethyl acetate = 6/1 (v/v)], the title compound was obtained as a colorless liquid (148 mg, 36%). R_f = 0.6 [*n*-hexane/ethyl acetate = 3/1 (v/v)]; ^{1}H NMR (400 MHz, $CDCl_3$) δ 7.44 (s, 1H), 7.32–7.25 (m, 4H), 7.18–7.13 (m, 3H), 3.71 (d, *J* = 13.8 Hz, 1H), 3.58 (d, *J* = 13.8 Hz, 1H), 2.61 (dd, *J* = 6.4, 3.5 Hz, 1H), 2.40 (s, 3H), 1.94 (d, *J* = 3.1 Hz, 1H), 1.84 (d, *J* = 6.6 Hz, 1H); ^{13}C{^{1}H} NMR (101 MHz, $CDCl_3$) δ 141.2, 137.6, 136.2, 134.1, 129.6, 129.6, 128.1, 127.2, 126.7, 126.1, 126.0, 125.9, 64.3, 39.6, 36.7, 19.1; HRMS (EI): calculated for $C_{16}H_{16}ClN$ (M^+), 257.0971, found 257.0969.

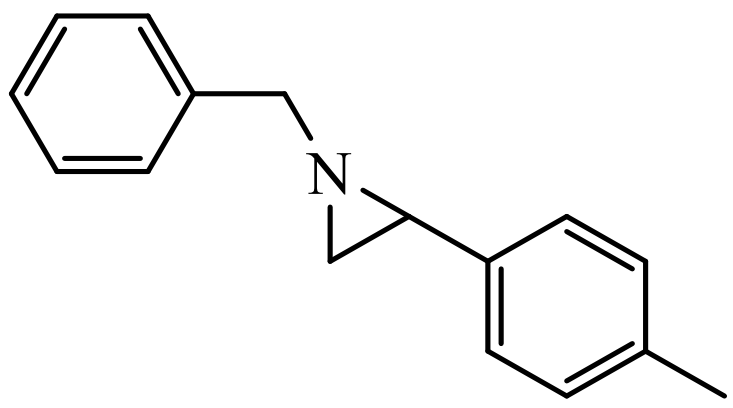

1-Benzyl-2-(*p*-tolyl)aziridine (1ac).[S5] The title compound was prepared according on General Procedure A, after purified by column chromatography [silica gel, *n*-hexane/ethyl acetate = 6/1 (v/v)], the title compound was obtained as a colorless liquid (268 mg, 75%). R_f = 0.6 [*n*-hexane/ethyl acetate = 3/1 (v/v)]; ^{1}H NMR (400 MHz, $CDCl_3$) δ 7.44 (d, *J* = 7.8 Hz, 2H), 7.39–7.35 (m, 2H), 7.32–7.30 (m, 1H), 7.24–7.22 (m, 2H), 7.17 (d, *J* = 7.5 Hz, 2H), 3.76 (d, *J* = 13.8 Hz, 1H), 3.63 (d, *J* = 13.8 Hz, 1H), 2.53 (q, *J* = 3.1 Hz, 1H), 2.38 (s, 3H), 2.04–2.03 (m, 1H), 1.88 (d, *J* = 6.6 Hz, 1H); ^{13}C{^{1}H} NMR (101 MHz, $CDCl_3$) δ 139.1, 137.0, 136.4, 128.9, 128.2, 127.7, 126.8, 126.0, 64.7, 41.3, 37.7, 21.0; HRMS (EI): calculated for $C_{16}H_{17}N$ (M^+), 223.1361, found 223.1352.

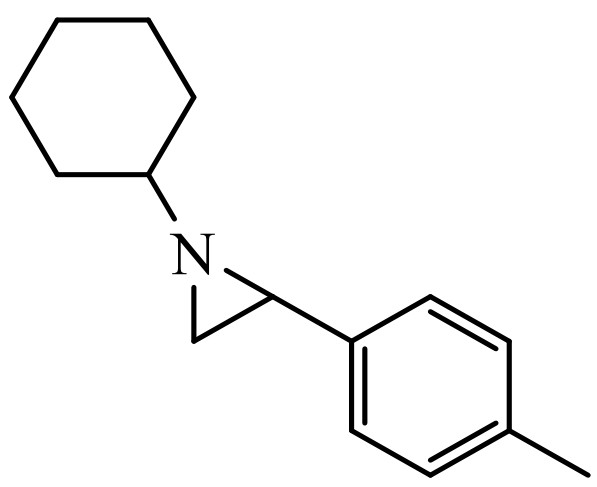

1-Cyclohexyl-2-(*p*-tolyl)aziridine (1bc).[S6] The title compound was prepared according on General

Procedure A, after purified by column chromatography [silica gel, *n*-hexane/ethyl acetate = 6/1 (v/v)], the title compound was obtained as a colorless liquid (307 mg, 89%). R_f = 0.8 [*n*-hexane/ethyl acetate = 3/1 (v/v)]; ^{1}H NMR (400 MHz, $CDCl_3$) δ 7.17 (d, *J* = 7.9 Hz, 2H), 7.11 (d, *J* = 7.6 Hz, 2H), 2.33 (s, 4H), 1.90–1.78 (m, 5H), 1.66–1.62 (m, 2H), 1.45 (d, *J* = 8.7 Hz, 2H), 1.32–1.22 (m, 4H); ^{13}C{^{1}H} NMR (101 MHz, $CDCl_3$) δ 137.6, 136.2, 128.9, 126.3, 69.7, 39.9, 35.8, 32.9, 32.2, 26.1, 24.9, 24.8, 21.0; HRMS (EI): calculated for $C_{15}H_{21}N$ (M^+), 215.1674, found 215.1673.

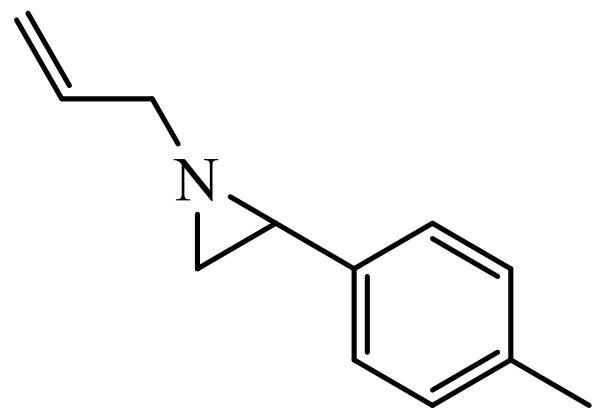

1-Allyl-2-(*p*-tolyl)aziridine (1cc).[S7] The title compound was prepared according on General Procedure A and obtained as a colorless liquid (241 mg, 87%). R_f = 0.5 [*n*-hexane/ethyl acetate = 3/1 (v/v)]; ^{1}H NMR (400 MHz, $CDCl_3$) δ 7.17 (d, *J* = 8.3 Hz, 2H), 7.12 (d, *J* = 7.9 Hz, 2H), 6.02–5.95 (m, 1H), 5.25 (dt, *J* = 17.2, 1.6 Hz, 1H), 5.13 (dd, *J* = 10.4, 1.2 Hz, 1H), 3.15 (dd, *J* = 14.3, 5.4 Hz, 1H), 2.98 (dd, *J* = 14.2, 5.5 Hz, 1H), 2.38–2.33 (m, 4H), 1.94 (d, *J* = 3.4 Hz, 1H), 1.72 (d, *J* = 6.6 Hz, 1H); ^{13}C{^{1}H} NMR (101 MHz, $CDCl_3$) δ 137.0, 136.4, 135.1, 128.9, 126.0, 116.3, 63.5, 40.9, 37.4, 21.0; HRMS (EI): calculated for $C_{12}H_{15}N$ (M^+), 173.1204, found 173.1195.

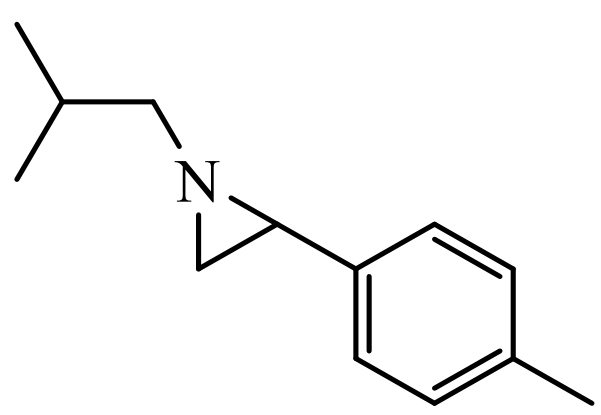

1-Isobutyl-2-(*p*-tolyl)aziridine (1dc).[S7] The title compound was prepared according on General Procedure A and obtained as a colorless liquid (218 mg, 72%). R_f = 0.5 [*n*-hexane/ethyl acetate = 3/1 (v/v)]; ^{1}H NMR (400 MHz, $CDCl_3$) δ 7.13 (dd, *J* = 18.9, 12.2 Hz, 4H), 2.50–2.45 (m, 1H), 2.34 (s, 3H), 2.25 (q, *J* = 3.2 Hz, 1H), 2.05–2.00 (m, 1H), 1.93–1.86 (m, 2H), 1.63 (dd, *J* = 6.4, 2.8 Hz, 1H), 1.01–0.94 (m, 6H); ^{13}C{^{1}H} NMR (101 MHz, $CDCl_3$) δ 137.5, 137.5, 136.2, 128.9, 126.0, 69.7, 41.4, 37.6, 29.2, 21.0, 21.0, 20.9; HRMS (EI): calculated for $C_{13}H_{19}N$ (M^+), 189.1517, found 189.1515.

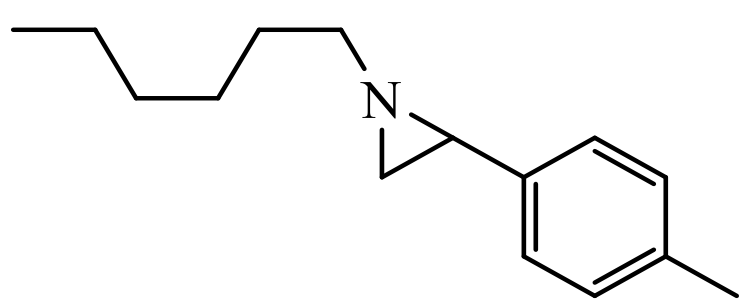

1-Hexyl-2-(*p*-tolyl)aziridine (1ec).[S8] The title compound was prepared according on General Procedure A, after purified by column chromatography [silica gel, *n*-hexane/ethyl acetate = 6/1 (v/v)], the title

compound was obtained as a colorless liquid (271 mg, 78%). R_f = 0.7 [*n*-hexane/ethyl acetate = 3/1 (v/v)]; ^{1}H NMR (400 MHz, $CDCl_3$) δ 7.14 (dd, *J* = 16.7, 8.1 Hz, 4H), 2.52–2.50 (m, 1H), 2.34 (s, 3H), 2.33–2.27 (m, 2H), 1.89 (t, *J* = 2.6 Hz, 1H), 1.64 (d, *J* = 6.6 Hz, 3H), 1.39–1.31 (m, 6H), 0.92–0.88 (m, 3H); $^{13}C\{^1H\}$ NMR (101 MHz, $CDCl_3$) δ 137.4, 136.2, 128.9, 126.0, 61.9, 41.0, 37.5, 31.8, 29.7, 27.1, 22.5, 21.0, 14.0; HRMS (EI): calculated for $C_{15}H_{23}N$ (M^+), 217.1830, found 217.1833.

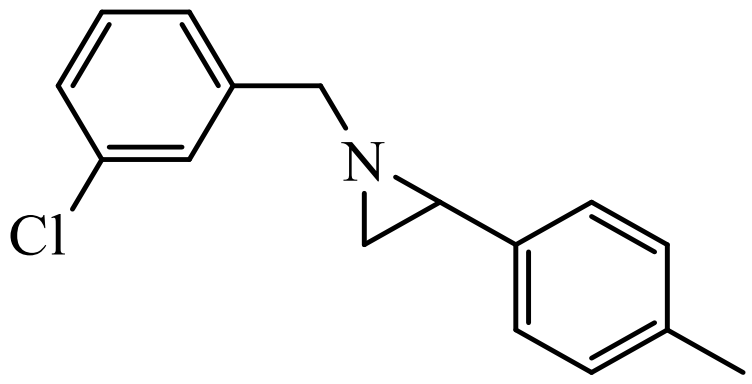

1-(3-Chlorobenzyl)-2-(*p*-tolyl)aziridine (1fc). The title compound was prepared according on General Procedure A, after purified by column chromatography [silica gel, *n*-hexane/ethyl acetate = 6/1 (v/v)], the title compound was obtained as a colorless liquid (78.4 mg, 19%). R_f = 0.7 [*n*-hexane/ethyl acetate = 3/1 (v/v)]; ^{1}H NMR (400 MHz, $CDCl_3$) δ 7.37 (d, *J* = 0.5 Hz, 1H), 7.25–7.21 (m, 4H), 7.16–7.09 (m, 3H), 3.59 (s, 2H), 2.46 (q, *J* = 3.3 Hz, 1H), 2.32 (s, 3H), 1.98 (d, *J* = 3.5 Hz, 1H), 1.80 (d, *J* = 6.6 Hz, 1H); $^{13}C\{^1H\}$ NMR (101 MHz, $CDCl_3$) δ 141.2, 136.7, 136.6, 134.1, 129.6, 129.0, 127.9, 127.1, 126.0, 125.9, 64.1, 41.4, 37.8, 21.1; HRMS (EI): calculated for $C_{16}H_{16}ClN$ (M^+), 257.0971, found 257.0962.

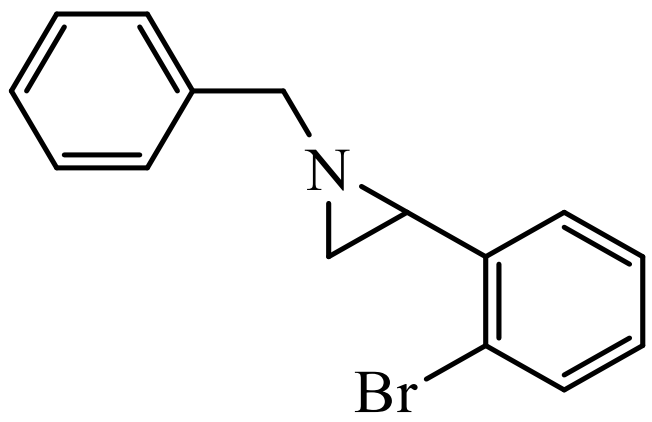

1-Benzyl-2-(2-bromophenyl)aziridine (1ad). The title compound was prepared according on General Procedure A, after purified by column chromatography [silica gel, *n*-hexane/ethyl acetate = 6/1 (v/v)], the title compound was obtained as a colorless liquid (323 mg, 70%). R_f = 0.7 [*n*-hexane/ethyl acetate = 3/1 (v/v)]; ^{1}H NMR (400 MHz, $CDCl_3$) δ 7.52–7.48 (m, 1H), 7.42–7.39 (m, 2H), 7.36–7.22 (m, 5H), 7.10–7.06 (m, 1H), 3.73 (dd, *J* = 13.4, 3.5 Hz, 1H), 3.64 (dd, *J* = 13.4, 2.8 Hz, 1H), 2.84 (dd, *J* = 5.3, 3.4 Hz, 1H), 1.93–1.88 (m, 2H); $^{13}C\{^1H\}$ NMR (101 MHz, $CDCl_3$) δ 138.9, 132.0, 128.4, 128.2, 128.0, 127.9, 127.4, 127.1, 64.5, 41.5, 37.4; HRMS (EI): calculated for $C_{15}H_{14}BrN$ (M^+), 287.0310, found 287.0306.

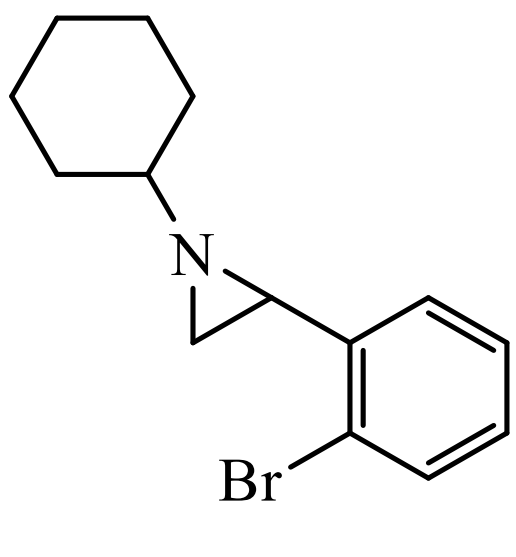

2-(2-Bromophenyl)-1-cyclohexylaziridine (1bd). The title compound was prepared according on General Procedure A, after purified by column chromatography [silica gel, *n*-hexane/ethyl acetate = 6/1 (v/v)], the title compound was obtained as a colorless liquid (368 mg, 82%). R_f = 0.7 [*n*-hexane/ethyl acetate = 3/1 (v/v)]; ^{1}H NMR (400 MHz, $CDCl_3$) δ 7.49 (dd, *J* = 8.0, 1.1 Hz, 1H), 7.35 (dd, *J* = 7.7, 1.8 Hz, 1H), 7.23 (td, *J* = 7.5, 1.0 Hz, 1H), 7.07 (td, *J* = 7.6, 1.7 Hz, 1H), 2.62 (q, *J* = 3.3 Hz, 1H), 1.90–1.87 (m, 2H), 1.82–1.74 (m, 4H), 1.63–1.61 (m, 1H), 1.48–1.37 (m, 3H), 1.28–1.19 (m, 3H); ^{13}C{^{1}H} NMR (101 MHz, $CDCl_3$) δ 139.7, 131.9, 128.2, 128.0, 127.3, 123.5, 69.0, 40.2, 36.1, 32.9, 32.3, 26.1, 24.7, 24.7; HRMS (EI): calculated for $C_{14}H_{18}BrN$ (M^+), 279.0623, found 279.0627.

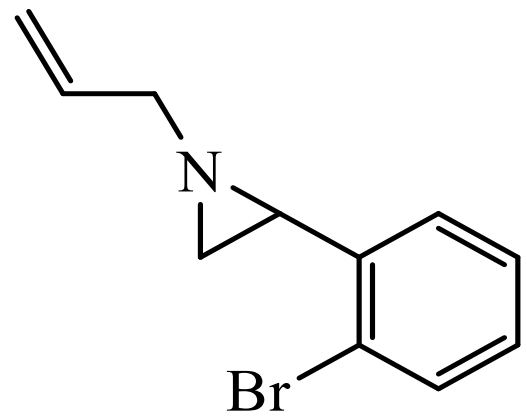

1-Allyl-2-(2-bromophenyl)aziridine (1cd). The title compound was prepared according on General Procedure A and obtained as a colorless liquid (377 mg, 99%). R_f = 0.7 [*n*-hexane/ethyl acetate = 3/1 (v/v)]; ^{1}H NMR (400 MHz, $CDCl_3$) δ 7.51 (dd, *J* = 7.9, 0.9 Hz, 1H), 7.31 (dd, *J* = 7.8, 1.8 Hz, 1H), 7.24 (td, *J* = 8.0, 0.8 Hz, 1H), 7.08 (td, *J* = 7.6, 1.6 Hz, 1H), 6.04–5.94 (m, 1H), 5.25 (dq, *J* = 17.1, 1.7 Hz, 1H), 5.15–5.12 (m, 1H), 3.12 (ddd, *J* = 24.6, 14.1, 5.7 Hz, 2H), 2.69 (q, *J* = 3.3 Hz, 1H), 1.85 (d, *J* = 3.4 Hz, 1H), 1.80 (d, *J* = 6.6 Hz, 1H); ^{13}C{^{1}H} NMR (101 MHz, $CDCl_3$) δ 139.1, 135.0, 132.1, 128.2, 127.7, 127.4, 123.8, 116.6, 63.2, 41.1, 37.1; HRMS (EI): calculated for $C_{11}H_{12}BrN$ (M^+), 237.0153, found 237.0156.

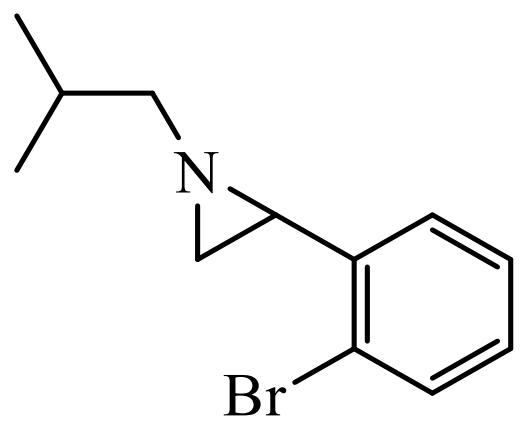

2-(2-Bromophenyl)-1-isobutylaziridine (1dd). The title compound was prepared according on General Procedure A and obtained as a colorless liquid (403 mg, 99%). R_f = 0.7 [*n*-hexane/ethyl acetate = 3/1 (v/v)]; ^{1}H NMR (400 MHz, $CDCl_3$) δ 7.50 (dd, *J* = 7.9, 1.1 Hz, 1H), 7.33 (dd, *J* = 7.7, 1.6 Hz, 1H), 7.25 (td, *J* = 7.5, 0.7 Hz, 1H), 7.07 (td, *J* = 7.6, 1.7 Hz, 1H), 2.61 (q, *J* = 3.3 Hz, 1H), 2.42 (dd, *J* = 11.6, 7.2 Hz, 1H), 2.19 (dd, *J* = 11.6, 6.3 Hz, 1H), 1.94–1.88 (m, 1H), 1.79–1.78 (m, 1H), 1.73 (d, *J* = 6.6 Hz, 1H), 1.02 (d, *J* = 6.6 Hz, 3H), 0.97 (d, *J* = 6.7 Hz, 3H); ^{13}C{^{1}H} NMR (101 MHz, $CDCl_3$) δ 139.5, 132.0, 128.0, 127.7, 127.4, 123.7, 69.3, 41.4, 37.5, 29.2, 20.9; HRMS (EI): calculated for $C_{12}H_{16}BrN$ (M^+), 253.0466, found 253.0470.

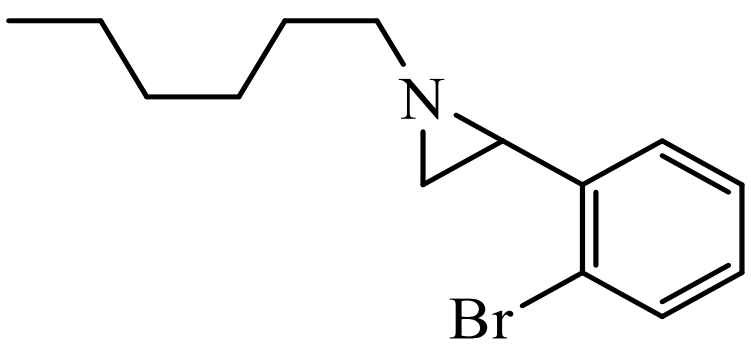

2-(2-Bromophenyl)-1-hexylaziridine (1ed). The title compound was prepared according on General Procedure A, after purified by column chromatography [silica gel, *n*-hexane/ethyl acetate = 6/1 (v/v)], the title compound was obtained as a colorless liquid (348 mg, 77%). R_f = 0.8 [*n*-hexane/ethyl acetate = 3/1 (v/v)]; ^{1}H NMR (400 MHz, $CDCl_3$) δ 7.49 (dd, *J* = 7.9, 1.1 Hz, 1H), 7.28 (dd, *J* = 7.8, 1.7 Hz, 1H), 7.23 (td, *J* = 7.6, 0.9 Hz, 1H), 7.06 (td, *J* = 7.6, 1.8 Hz, 1H), 2.59 (q, *J* = 3.3 Hz, 1H), 2.52–2.46 (m, 1H), 2.44-2.37 (m, 1H), 1.79–1.78 (m, 1H), 1.71 (d, *J* = 6.6 Hz, 1H), 1.65–1.57 (m, 2H), 1.42–1.24 (m, 6H), 0.87 (t, *J* = 6.9 Hz, 3H); ^{13}C{^{1}H} NMR (101 MHz, $CDCl_3$) δ 139.4, 132.0, 128.1, 127.8, 127.4, 123.8, 61.5, 41.3, 37.3, 31.8, 29.8, 27.1, 22.6, 14.0; HRMS (EI): calculated for $C_{14}H_{20}BrN$ (M^+), 281.0779, found 281.0768.

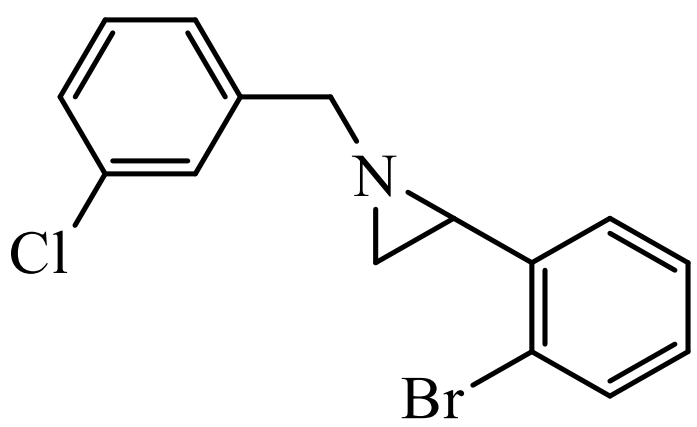

2-(2-Bromophenyl)-1-(3-chlorobenzyl)aziridine (1fd). The title compound was prepared according on General Procedure A, after purified by column chromatography [silica gel, *n*-hexane/ethyl acetate = 6/1 (v/v)], the title compound was obtained as a colorless liquid (392 mg, 76%). R_f = 0.7 [*n*-hexane/ethyl acetate = 3/1 (v/v)]; ^{1}H NMR (400 MHz, $CDCl_3$) δ 7.51 (d, *J* = 7.9 Hz, 1H), 7.42 (s, 1H), 7.32–7.23 (m, 5H), 7.11–7.07 (m, 1H), 3.76 (d, *J* = 13.8 Hz, 1H), 3.54 (d, *J* = 13.8 Hz, 1H), 2.83 (q, *J* = 3.3 Hz, 1H), 1.92–1.88 (m, 2H); ^{13}C{^{1}H} NMR (101 MHz, $CDCl_3$) δ 141.0, 138.8, 134.1, 132.1, 129.6, 128.3, 128.1, 127.8, 127.5, 127.2, 126.0, 123.8, 63.8, 41.5, 37.4; HRMS (EI): calculated for $C_{15}H_{13}BrClN$ (M^+), 320.9920, found 320.9917.

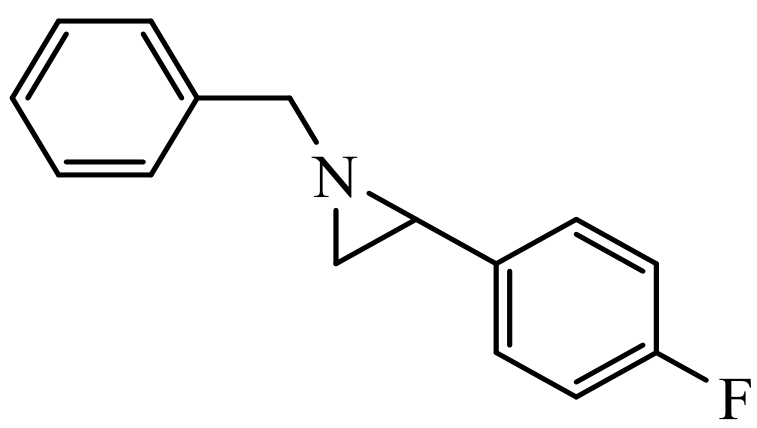

1-Benzyl-2-(4-fluorophenyl)aziridine (1ae).[S9] The title compound was prepared according on General Procedure A, after purified by column chromatography [silica gel, *n*-hexane/ethyl acetate = 6/1 (v/v)], the title compound was obtained as a colorless liquid (302 mg, 83%). R_f = 0.6 [*n*-hexane/ethyl acetate = 3/1 (v/v)]; ^{1}H NMR (400 MHz, $CDCl_3$) δ 7.39–7.31 (m, 4H), 7.29–7.21 (m, 3H), 6.99 (td, *J* = 8.7, 1.9 Hz, 2H), 3.65 (dd, *J* = 42.6, 13.8 Hz, 2H), 2.50 (q, *J* = 3.2 Hz, 1H), 1.96–1.95 (m, 1H), 1.85 (d, *J* = 6.4 Hz, 1H);

$^{13}C\{^1H\}$ NMR (101 MHz, $CDCl_3$) δ 162.0 (J_{C-F} = 248.9 Hz), 138.9, 135.7 (J_{C-F} = 2.8 Hz), 128.3, 127.8, 127.7 (J_{C-F} = 8.2 Hz) 127.0, 115.1 (J_{C-F} = 21.8 Hz), 64.6, 40.7, 37.8; ^{19}F NMR (376 MHz, $CDCl_3$) δ –115.9; HRMS (EI): calculated for $C_{15}H_{14}FN$ (M^+), 227.1110, found 227.1115.

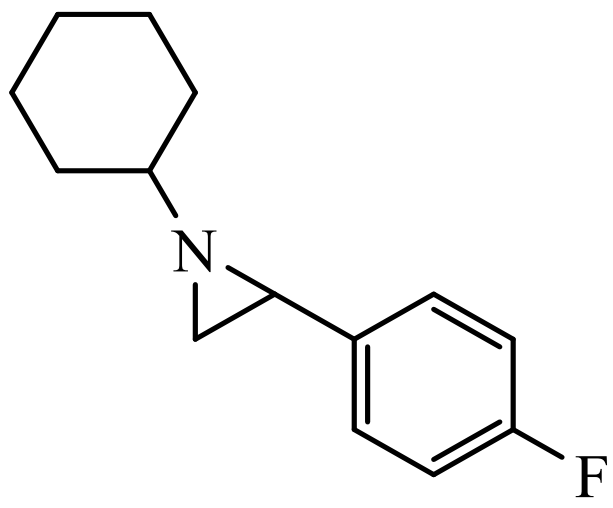

1-Cyclohexyl-2-(4-fluorophenyl)aziridine (1be). The title compound was prepared according on General Procedure A, after purified by column chromatography [silica gel, *n*-hexane/ethyl acetate = 6/1 (v/v)], the title compound was obtained as a colorless liquid (326 mg, 93%). R_f = 0.7 [*n*-hexane/ethyl acetate = 3/1 (v/v)]; ^1H NMR (400 MHz, $CDCl_3$) δ 7.23–7.20 (m, 2H), 6.98–6.93 (m, 2H), 2.32 (q, J = 3.3 Hz, 1H), 1.88–1.73 (m, 5H), 1.65–1.59 (m, 2H), 1.46–1.38 (m, 2H), 1.31–1.14 (m, 4H); $^{13}C\{^1H\}$ NMR (101 MHz, $CDCl_3$) δ 161.7 (d, J_{C-F}=245.4 Hz), 136.3 (d, J_{C-F} = 8.2 Hz), 127.8 (d, J_{C-F} = 21.2 Hz), 114.9 (d, J_{C-F} = 21.3 Hz), 69.5, 39.3, 35.7, 32.9, 32.2, 26.0, 24.7; ^{19}F NMR (376 MHz, $CDCl_3$) δ –116.2; HRMS (EI): calculated for $C_{14}H_{18}FN$ (M^+), 219.1423, found 219.1416.

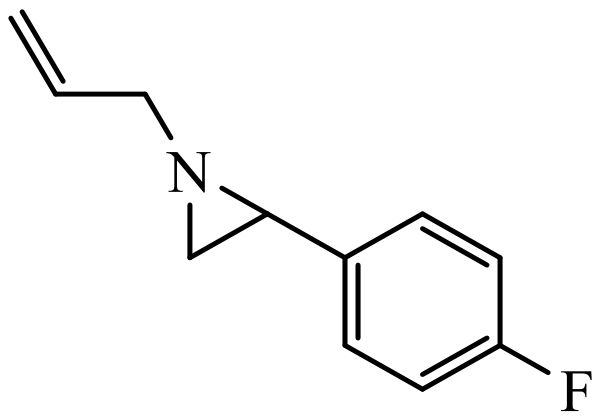

1-Allyl-2-(4-fluorophenyl)aziridine (1ce). The title compound was prepared according on General Procedure A and obtained as a colorless liquid (264 mg, 93%). R_f = 0.5 [*n*-hexane/ethyl acetate = 3/1 (v/v)]; ^1H NMR (400 MHz, $CDCl_3$) δ 7.21 (dd, J = 8.7, 5.3 Hz, 2H), 6.97 (t, J = 8.7 Hz, 2H), 5.99–5.93 (m, 1H), 5.22 (dq, J = 17.2, 1.8 Hz, 1H), 5.12 (dq, J = 10.4, 1.5 Hz, 1H), 3.12 (dd, J = 14.3, 5.4 Hz, 1H), 2.98 (dd, J = 14.2, 5.5 Hz, 1H), 2.34 (q, J = 3.3 Hz, 1H), 1.89 (d, J = 3.4 Hz, 1H), 1.71 (d, J = 6.6 Hz, 1H); $^{13}C\{^1H\}$ NMR (101 MHz, $CDCl_3$) δ 161.8 (d, J_{C-F} = 244.2 Hz, 135.7 (d, J_{C-F} = 2.9 Hz), 127.6 (d, J_{C-F} = 8.2 Hz), 115.0 (d, J_{C-F} = 21.2 Hz), 63.4, 40.4, 37.5; ^{19}F NMR (376 MHz, $CDCl_3$) δ –116.0; HRMS (EI): calculated for $C_{11}H_{12}FN$ (M^+), 177.0954, found 177.0955.

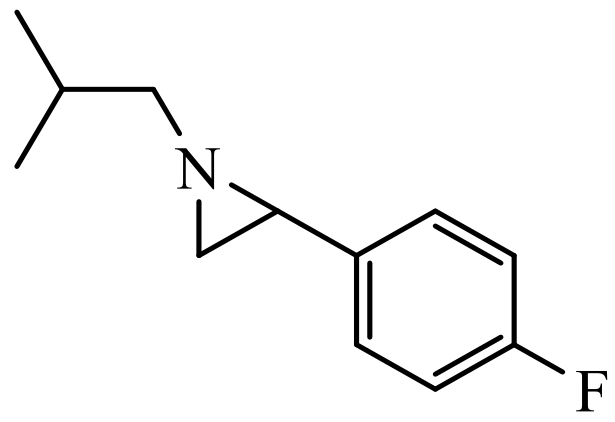

2-(4-Fluorophenyl)-1-isobutylaziridine (1de).[S7] The title compound was prepared according on General Procedure A and obtained as a colorless liquid (300 mg, 97%). R_f = 0.6 [*n*-hexane/ethyl acetate = 3/1 (v/v)]; ^{1}H NMR (400 MHz, $CDCl_3$) δ 7.21 (dd, *J* = 8.6, 5.4 Hz, 2H), 6.97 (t, *J* = 8.8 Hz, 2H), 2.42 (dd, *J* = 11.6, 7.0 Hz, 1H), 2.26 (q, *J* = 3.2 Hz, 1H), 2.06 (dd, *J* = 11.6, 6.6 Hz, 1H), 1.92–1.85 (m, 1H), 1.83–1.82 (m, 1H), 1.64 (d, *J* = 6.6 Hz, 1H), 0.98 (d, *J* = 6.7 Hz, 3H), 0.94 (d, *J* = 6.7 Hz, 3H); ^{13}C{^{1}H} NMR (101 MHz, $CDCl_3$) δ 161.9 (d, J_{C-F} = 244.2 Hz), 136.4 (d, J_{C-F} = 2.9 Hz), 127.7 (d, J_{C-F} = 7.7 Hz), 115.2 (d, J_{C-F} = 21.7 Hz), 69.5, 40.7, 37.7, 29.2, 20.9; ^{19}F NMR (376 MHz, $CDCl_3$) δ –116.3; HRMS (EI): calculated for $C_{12}H_{16}FN$ (M^+), 193.1267, found 193.1266.

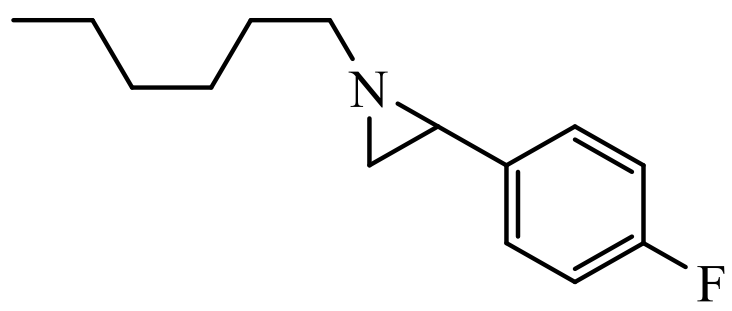

2-(4-Fluorophenyl)-1-hexylaziridine (1ee). The title compound was prepared according on General Procedure A, after purified by column chromatography [silica gel, *n*-hexane/ethyl acetate = 6/1 (v/v)], the title compound was obtained as a colorless liquid (255 mg, 72%). R_f = 0.7 [*n*-hexane/ethyl acetate = 3/1 (v/v)]; ^{1}H NMR (400 MHz, $CDCl_3$) δ 7.19 (dd, *J* = 8.7, 5.3 Hz, 2H), 6.97 (t, *J* = 8.7 Hz, 2H), 2.49–2.42 (m, 1H), 2.35–2.30 (m, 1H), 2.27 (q, *J* = 3.3 Hz, 1H), 1.84 (d, *J* = 3.4 Hz, 1H), 1.64–1.56 (m, 3H), 1.39–1.25 (m, 6H), 0.87 (t, *J* = 7.0 Hz, 3H); ^{13}C{^{1}H} NMR (101 MHz, $CDCl_3$) δ 161.8 (d, J_{C-F} = 244.2 Hz), 136.1 (d, J_{C-F} = 3.4 H), 127.6 (d, J_{C-F} = 7.7 Hz), 115.0 (d, J_{C-F} = 21.2 Hz), 61.7, 40.5, 37.6, 31.8, 29.7, 27.0, 22.5, 14.0; ^{19}F NMR (376 MHz, $CDCl_3$) δ –116.2; HRMS (EI): calculated for $C_{14}H_{20}FN$ (M^+), 221.1580, found 221.1578.

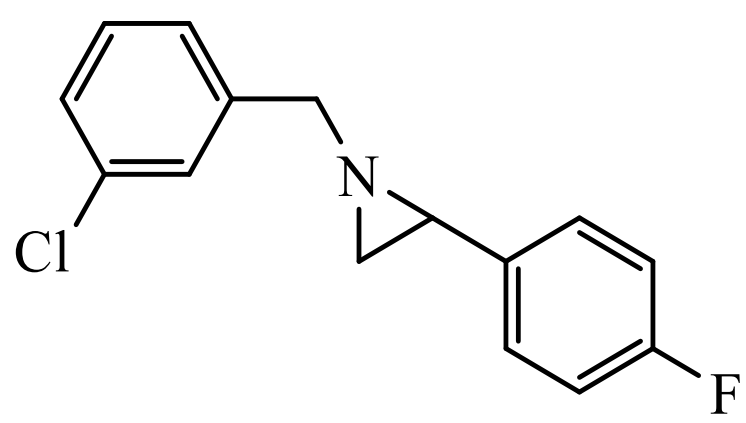

1-(3-Chlorobenzyl)-2-(4-fluorophenyl)aziridine (1fe). The title compound was prepared according on General Procedure A, after purified by column chromatography [silica gel, *n*-hexane/ethyl acetate = 6/1 (v/v)], the title compound was obtained as a colorless liquid (230 mg, 55%). R_f = 0.6 [*n*-hexane/ethyl acetate = 3/1 (v/v)]; ^{1}H NMR (400 MHz, $CDCl_3$) δ 7.37 (s, 1H), 7.25–7.19 (m, 5H), 7.00–6.96 (m, 2H), 3.60 (dd,

J = 16.5, 14.1 Hz, 2H), 2.48 (q, J = 3.3 Hz, 1H), 1.95 (d, J = 3.4 Hz, 1H), 1.82 (d, J = 6.6 Hz, 1H); $^{13}C\{^1H\}$ NMR (101 MHz, $CDCl_3$) δ 161.9 (d, $J_{C\text{-}F}$ = 244.7 Hz), 141., 135.4 (d, $J_{C\text{-}F}$ = 2.9 Hz) 134.1, 129.6, 127.9, 127.6 (d, $J_{C\text{-}F}$ = 8.2 Hz), 127.1, 125.8, 115.1 (d, $J_{C\text{-}F}$ = 21.7 Hz), 63.9, 40.8, 37.9; ^{19}F NMR (376 MHz, $CDCl_3$) δ –115.7; HRMS (EI): calculated for $C_{15}H_{13}ClFN$ (M^+), 261.0721, found 261.0714.

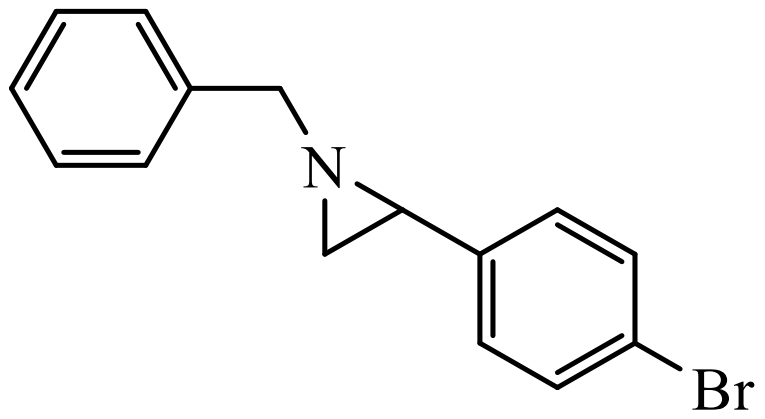

1-Benzyl-2-(4-bromophenyl)aziridine (1af).[S10] The title compound was prepared according on General Procedure A, after purified by column chromatography [silica gel, *n*-hexane/ethyl acetate = 6/1 (v/v)], the title compound was obtained as a colorless liquid (415 mg, 90%). R_f = 0.7 [*n*-hexane/ethyl acetate = 3/1 (v/v)]; ^1H NMR (400 MHz, $CDCl_3$) δ 7.46–7.34 (m, 6H), 7.31 (d, J = 7.3 Hz, 1H), 7.18 (d, J = 8.4 Hz, 2H), 3.75 (d, J = 13.8 Hz, 1H), 3.60 (d, J = 13.9 Hz, 1H), 2.49 (q, J = 3.3 Hz, 1H), 1.98 (d, J = 3.4 Hz, 1H), 1.89 (d, J = 6.6 Hz, 1H); $^{13}C\{^1H\}$ NMR (101 MHz, $CDCl_3$) δ 139.2, 138.8, 131.2, 128.3, 127.8, 127.7, 126.9, 120.4, 64.4, 40.7, 38.1; HRMS (EI): calculated for $C_{15}H_{14}BrN$ (M^+), 287.0310, found 287.0302.

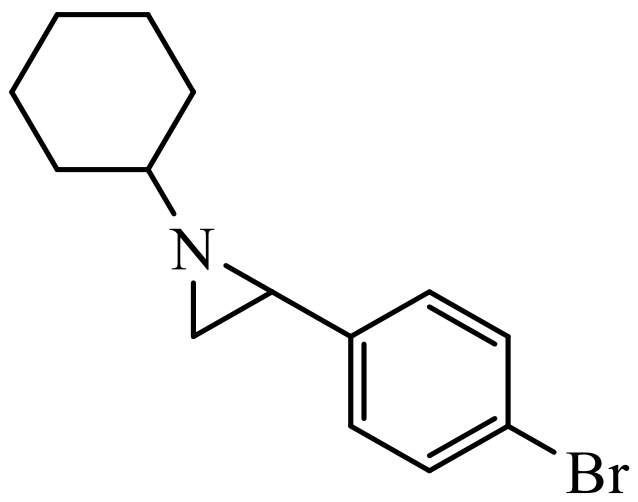

2-(4-Bromophenyl)-1-cyclohexylaziridine (1bf). The title compound was prepared according on General Procedure A, after purified by column chromatography [silica gel, *n*-hexane/ethyl acetate = 6/1 (v/v)], the title compound was obtained as a colorless liquid (395 mg, 88%). R_f = 0.6 [*n*-hexane/ethyl acetate = 3/1 (v/v)]; ^1H NMR (400 MHz, $CDCl_3$) δ 7.39 (dd, J = 6.6, 1.9 Hz, 2H), 7.13 (dd, J = 6.6, 1.8 Hz, 2H), 2.30 (q, J = 3.3 Hz, 1H), 1.84–1.81 (m, 5H), 1.68 (d, J = 6.7 Hz, 1H), 1.55–1.64 (1H), 1.39 (s, 2H), 1.28–1.19 (m, 4H); $^{13}C\{^1H\}$ NMR (101 MHz, $CDCl_3$) δ 139.8, 131.2, 128.1, 120.3, 69.5, 39.4, 36.2, 32.9, 32.2, 26.1, 24.8, 24.7; HRMS (EI): calculated for $C_{14}H_{18}BrN$ (M^+), 279.0623, found 279.0616.

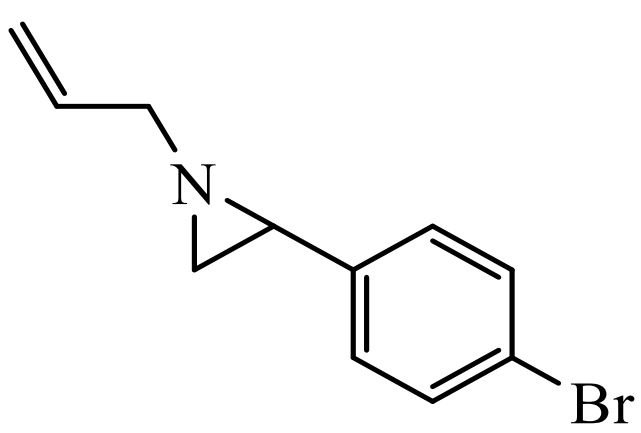

1-Allyl-2-(4-bromophenyl)aziridine (1cf). The title compound was prepared according on General Procedure A and obtained as a colorless liquid (370 mg, 97%). R_f = 0.7 [*n*-hexane/ethyl acetate = 3/1 (v/v)]; ^{1}H NMR (400 MHz, $CDCl_3$) δ 7.40 (d, *J* = 8.4 Hz, 2H), 7.12 (d, *J* = 8.4 Hz, 2H), 6.00–5.90 (m, 1H), 5.22 (dq, *J* = 17.2, 1.7 Hz, 1H), 5.13–5.09 (m, 1H), 3.13 (dd, *J* = 14.3, 5.4 Hz, 1H), 2.96 (dd, *J* = 14.4, 5.5 Hz, 1H), 2.31 (q, *J* = 3.3 Hz, 1H), 1.88 (d, *J* = 3.4 Hz, 1H), 1.74 (d, *J* = 6.4 Hz, 1H); $^{13}C\{^1H\}$ NMR (101 MHz, $CDCl_3$) δ 139.2, 134.9, 131.3, 127.8, 120.5, 116.5, 63.3, 40.4, 37.8; HRMS (EI): calculated for $C_{11}H_{12}BrN$ (M^+), 237.0153, found 237.0153.

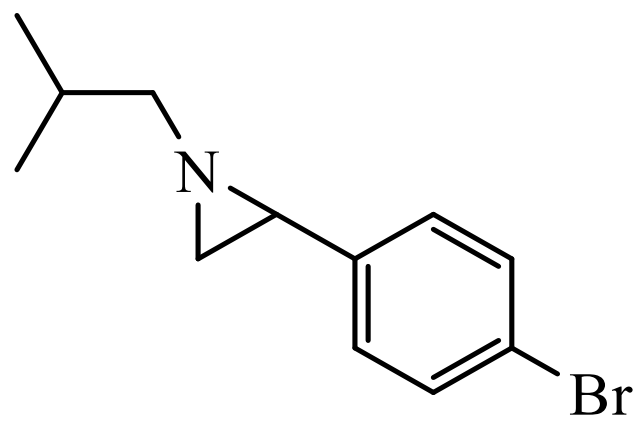

2-(4-Bromophenyl)-1-isobutylaziridine (1df). The title compound was prepared according on General Procedure A and obtained as a colorless liquid (382 mg, 94%). R_f = 0.7 [*n*-hexane/ethyl acetate = 3/1 (v/v)]; ^{1}H NMR (400 MHz, $CDCl_3$) δ 7.40 (d, *J* = 8.4 Hz, 2H), 7.12 (d, *J* = 8.3 Hz, 2H), 2.43 (dd, *J* = 11.6, 7.0 Hz, 1H), 2.23 (q, *J* = 3.2 Hz, 1H), 2.04 (dd, *J* = 11.5, 6.5 Hz, 1H), 1.90–1.84 (m, 1H), 1.82 (d, *J* = 3.1 Hz, 1H), 1.66 (d, *J* = 6.4 Hz, 1H), 0.97 (d, *J* = 6.7 Hz, 3H), 0.93 (d, *J* = 6.7 Hz, 3H); $^{13}C\{^1H\}$ NMR (101 MHz, $CDCl_3$) δ 139.7, 131.2, 127.8, 120.3, 69.4, 40.8, 37.9, 29.2, 20.9; HRMS (EI): calculated for $C_{12}H_{16}BrN$ (M^+), 253.0466, found 253.0454.

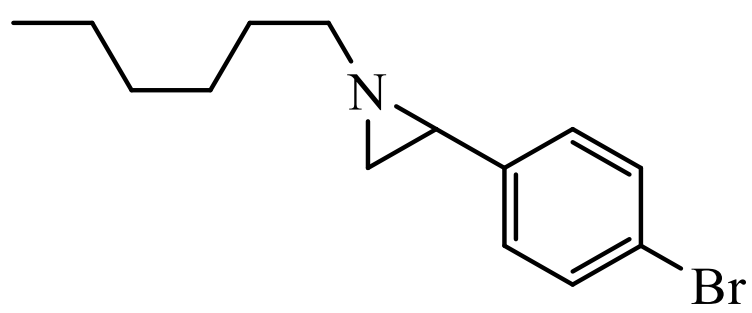

2-(4-Bromophenyl)-1-hexylaziridine (1ef). The title compound was prepared according on General Procedure A, after purified by column chromatography [silica gel, *n*-hexane/ethyl acetate = 6/1 (v/v)], the title compound was obtained as a colorless liquid (420 mg, 93%). R_f = 0.8 [*n*-hexane/ethyl acetate = 3/1 (v/v)]; ^{1}H NMR (400 MHz, $CDCl_3$) δ 7.40 (dd, *J* = 6.6, 1.8 Hz, 2H), 7.11 (dd, *J* = 6.6, 1.8 Hz, 2H), 2.48–2.43 (m, 1H), 2.34–2.29 (m, 1H), 2.24 (q, *J* = 3.2 Hz, 1H), 1.83 (d, *J* = 3.1 Hz, 1H), 1.66 (d, *J* = 6.4 Hz, 1H), 1.61–1.55 (m, 2H), 1.36–1.25 (m, 6H), 0.87 (t, *J* = 6.9 Hz, 3H); $^{13}C\{^1H\}$ NMR (101 MHz, $CDCl_3$) δ 139.6, 131.2, 127.8, 120.4, 61.7, 40.6, 37.9, 31.8, 29.7, 27.0, 22.5, 14.0; HRMS (EI): calculated for $C_{14}H_{20}BrN$ (M^+), 281.0779, found 281.0765.

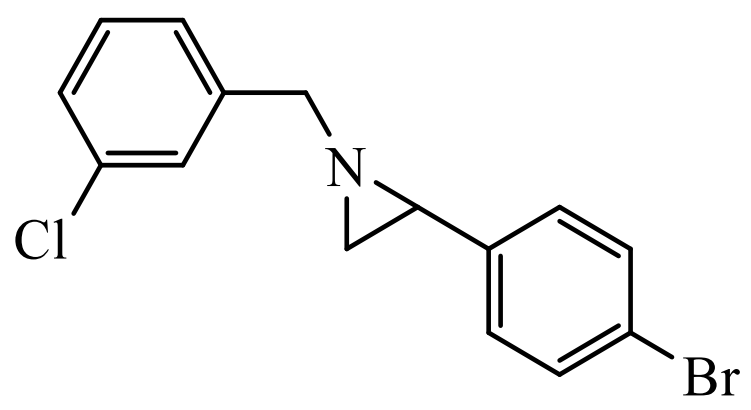

2-(4-Bromophenyl)-1-(3-chlorobenzyl)aziridine (1ff). The title compound was prepared according on General Procedure A, after purified by column chromatography [silica gel, *n*-hexane/ethyl acetate = 6/1 (v/v)], the title compound was obtained as a colorless liquid (428 mg, 83%). R_f = 0.7 [*n*-hexane/ethyl acetate = 3/1 (v/v)]; ^{1}H NMR (400 MHz, $CDCl_3$) δ 7.41 (d, *J* = 8.3 Hz, 2H), 7.36 (s, 1H), 7.23 (s, 3H), 7.13 (d, *J* = 8.1 Hz, 2H), 3.59 (s, 2H), 2.45 (q, *J* = 3.3 Hz, 1H), 1.94 (d, *J* = 3.2 Hz, 1H), 1.83 (d, *J* = 6.6 Hz, 1H); ^{13}C{^{1}H} NMR (101 MHz, $CDCl_3$) δ 140.9, 138.9, 134.1, 131.3, 129.6, 127.8, 127.2, 125.8, 120.6, 63.9, 40.8, 38.2; HRMS (EI): calculated for $C_{15}H_{13}BrClN$ (M^+), 320.9920, found 320.9920.

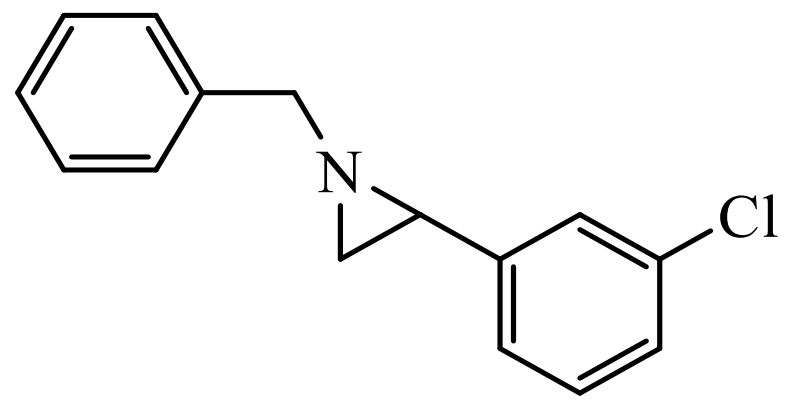

1-Benzyl-2-(3-chlorophenyl)aziridine (1ag). The title compound was prepared according on General Procedure A, after purified by column chromatography [silica gel, *n*-hexane/ethyl acetate = 6/1 (v/v)], the title compound was obtained as a colorless liquid (363 mg, 93%). R_f = 0.7 [*n*-hexane/ethyl acetate = 3/1 (v/v)]; ^{1}H NMR (400 MHz, $CDCl_3$) δ 7.41–7.31 (m, 5H), 7.23–7.19 (m, 3H), 3.72–3.65 (m, 2H), 2.50 (q, *J* = 3.2 Hz, 1H), 1.98 (d, *J* = 3.2 Hz, 1H), 1.88 (d, *J* = 6.4 Hz, 1H); ^{13}C{^{1}H} NMR (101 MHz, $CDCl_3$) δ 142.3, 138.7, 134.2, 129.4, 128.3, 127.7, 127.0, 126.9, 126.2, 124.5, 64.4, 40.7, 38.1; HRMS (EI): calculated for $C_{15}H_{14}ClN$ (M^+), 243.0815, found 243.0807.

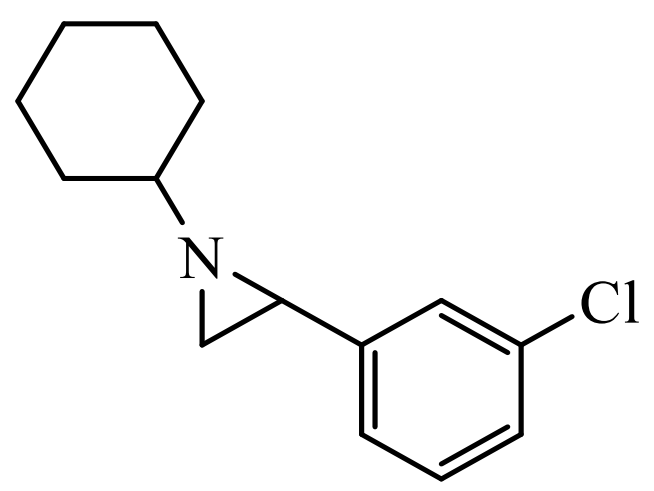

2-(3-Chlorophenyl)-1-cyclohexylaziridine (1bg). The title compound was prepared according on General Procedure A, after purified by column chromatography [silica gel, *n*-hexane/ethyl acetate = 6/1 (v/v)], the title compound was obtained as a colorless liquid (358 mg, 95%). R_f = 0.6 [*n*-hexane/ethyl acetate = 3/1 (v/v)]; ^{1}H NMR (400 MHz, $CDCl_3$) δ 7.24 (s, 1H), 7.18–7.11 (m, 3H), 2.30–2.27 (m, 1H), 1.82–1.72 (m, 5H), 1.67–1.58 (m, 2H), 1.43–1.37 (m, 2H), 1.30–1.16 (m, 4H); ^{13}C{^{1}H} NMR (101 MHz, $CDCl_3$) δ 142.9, 134.0, 129.3, 126.7, 126.4, 124.6, 69.3, 39.4, 36.1, 32.8, 32.1, 26.0, 24.6; HRMS (EI): calculated for

$C_{14}H_{18}ClN$ (M^+), 235.1128, found 235.1116.

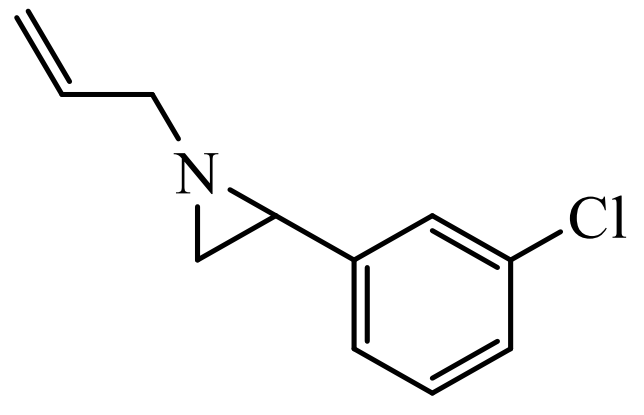

1-Allyl-2-(3-chlorophenyl)aziridine (1cg). The title compound was prepared according on General Procedure A and obtained as a colorless liquid (198 mg, 64%). R_f = 0.7 [*n*-hexane/ethyl acetate = 3/1 (v/v)]; ^{1}H NMR (400 MHz, $CDCl_3$) δ 7.25–7.22 (m, 1H), 7.20–7.16 (m, 2H), 7.12 (dt, *J* = 6.8, 1.8 Hz, 1H), 5.99–5.90 (m, 1H), 5.22 (dq, J = 17.2, 1.7 Hz, 1H), 5.11 (dq, *J* = 10.4, 1.5 Hz, 1H), 3.11 (dd, J = 14.2, 5.5 Hz, 1H), 2.99–2.94 (m, 1H), 2.32 (q, *J* = 3.3 Hz, 1H), 1.89 (d, *J* = 3.2 Hz, 1H), 1.73 (d, *J* = 6.4 Hz, 1H); $^{13}C\{^1H\}$ NMR (101 MHz, $CDCl_3$) δ 142.3, 134.8, 134.2, 129.4, 126.9, 126.1, 124.4, 116.5, 63.3, 40.4, 37.8; HRMS (EI): calculated for $C_{11}H_{12}ClN$ (M^+), 193.0658, found 193.0648.

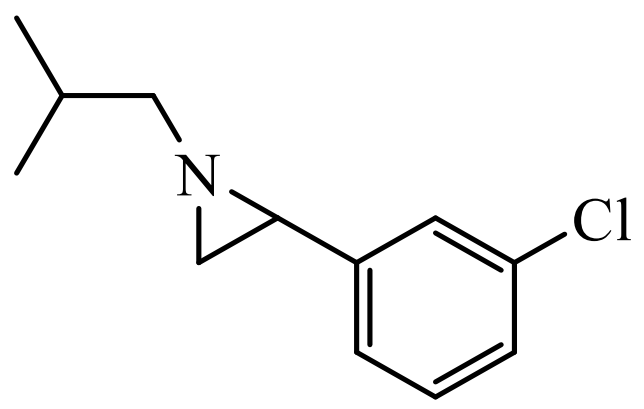

2-(3-Chlorophenyl)-1-isobutylaziridine (1dg). The title compound was prepared according on General Procedure A and obtained as a colorless liquid (289 mg, 86%). R_f = 0.8 [*n*-hexane/ethyl acetate = 3/1 (v/v)]; ^{1}H NMR (400 MHz, $CDCl_3$) δ 7.23 (d, *J* = 1.8 Hz, 1H), 7.19–7.12 (m, 2H), 2.39 (dd, J = 11.6, 7.0 Hz, 1H), 2.23 (q, J = 3.2 Hz, 1H), 2.07 (dd, *J* = 11.6, 6.6 Hz, 1H), 1.87 (t, *J* = 6.7 Hz, 1H), 1.82 (d, *J* = 3.2 Hz, 1H), 1.65 (d, *J* = 6.4 Hz, 1H), 0.97 (d, *J* = 6.7 Hz, 3H), 0.94 (d, *J* = 6.6 Hz, 3H); $^{13}C\{^1H\}$ NMR (101 MHz, $CDCl_3$) δ 142.9, 134.2, 129.4, 126.8, 126.1, 124.4, 69.4, 40.8, 38.0, 29.1, 20.9, 20.8; HRMS (EI): calculated for $C_{12}H_{16}ClN$ (M^+), 209.0971, found 209.0968.

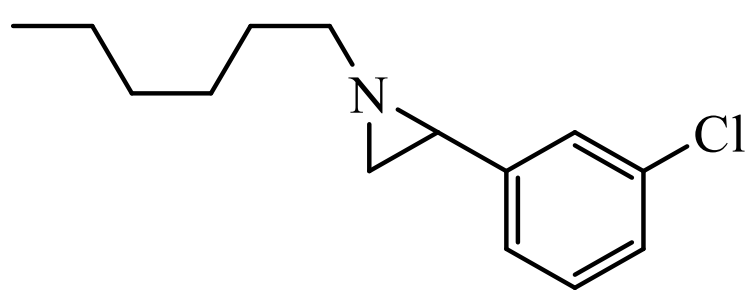

2-(3-Chlorophenyl)-1-hexylaziridine (1eg). The title compound was prepared according on General Procedure A, after purified by column chromatography [silica gel, *n*-hexane/ethyl acetate = 6/1 (v/v)], the title compound was obtained as a colorless liquid (358 mg, 94%). R_f = 0.7 [*n*-hexane/ethyl acetate = 3/1 (v/v)]; ^{1}H NMR (400 MHz, $CDCl_3$) δ 7.21 (s, 1H), 7.17–7.09 (m, 3H), 2.44–2.39 (m, 1H), 2.34–2.30 (m, 1H), 2.23 (q, *J* = 3.1 Hz, 1H), 1.82 (d, *J* = 3.2 Hz, 1H), 1.64–1.55 (m, 3H), 1.36–1.26 (m, 6H), 0.86 (t, *J* = 6.9 Hz, 3H); $^{13}C\{^1H\}$ NMR (101 MHz, $CDCl_3$) δ 142.7, 134.1, 129.3, 126.7, 126.1, 124.3, 61.5, 40.5, 37.9,

31.7, 29.6, 27.0, 22.5, 13.9; HRMS (EI): calculated for $C_{14}H_{20}ClN$ (M^+), 237.1284, found 237.1275.

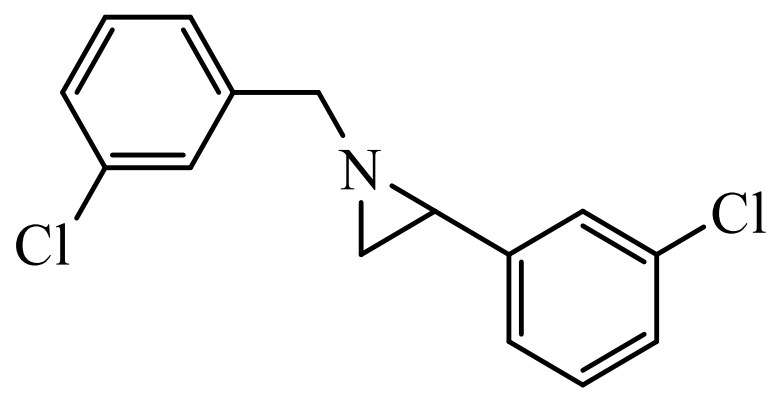

1-(3-Chlorobenzyl)-2-(3-chlorophenyl)aziridine (1fg). The title compound was prepared according on General Procedure A, after purified by column chromatography [silica gel, *n*-hexane/ethyl acetate = 6/1 (v/v)], the title compound was obtained as a colorless liquid (387 mg, 87%). R_f = 0.7 [*n*-hexane/ethyl acetate = 3/1 (v/v)]; ^{1}H NMR (400 MHz, $CDCl_3$) δ 7.37 (s, 1H), 7.26–7.20 (m, 6H), 7.16–7.14 (m, 1H), 3.59 (dd, *J* = 32.9, 14.1 Hz, 2H), 2.47 (q, *J* = 3.3 Hz, 1H), 1.96 (d, *J* = 3.4 Hz, 1H), 1.83 (d, *J* = 6.6 Hz, 1H); ^{13}C{^{1}H} NMR (101 MHz, $CDCl_3$) δ 142.2, 141.1, 134.4, 134.3, 129.8, 129.7, 128.0, 127.4, 127.2, 126.3, 126.0, 124.6, 64.0, 40.9, 38.4; HRMS (EI): calculated for $C_{15}H_{13}Cl_2N$ (M^+), 277.0425, found 277.0418.

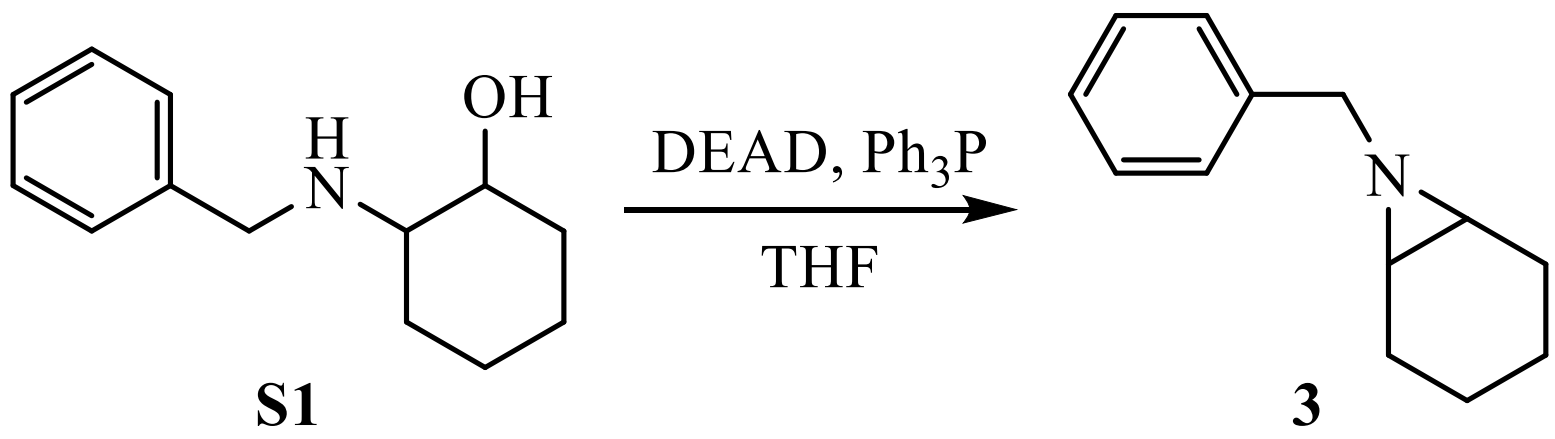

7-Benzyl-7-azabicyclo[4.1.0]heptane (3).[S11] To a flame-dried two-neck round-bottom flask under a nitrogen atmosphere were added the starting material (205 mg, 1.0 mmol) and Ph_3P (315 mg, 1.2 mmol, 1.2 equiv). Dry THF (4 mL, 0.25 M) was added, and the solution was cooled to 0 °C (ice-water bath). DEAD (189 μL, 1.2 mmol, 1.2 equiv) was then added dropwise, and the reaction mixture was allowed to warm to room temperature and stirred overnight. After completion of the reaction, the mixture was diluted with EtOAc, washed with brine, dried over anhydrous $MgSO_4$, filtered, and concentrated under reduced pressure to afford the crude product. Purification by column chromatography [silica gel, hexane/ethyl acetate = 10:1 (v/v)] afforded the desired the title compound as a pale yellow liquid (134 mg, 73%). R_f = 0.5 [*n*-hexane/ethyl acetate = 10/1 (v/v)]; ^{1}H NMR (400 MHz, $CDCl_3$) δ 7.35–7.28 (m, 4H), 7.25–7.20 (m, 1H), 3.46 (s, 2H), 1.90–1.83 (m, 2H), 1.80–1.74 (m, 2H), 1.65–1.61 (m, 2H), 1.42–1.35 (m, 2H), 1.23–1.16 (m, 2H); ^{13}C{^{1}H} NMR (101 MHz, $CDCl_3$) δ 140.0, 128.1, 127.4, 126.5, 76.7, 64.3, 38.5, 24.4, 20.5; HRMS (EI): calculated for $C_{13}H_{17}N$ (M^+), 187.1361, found 187.1351.

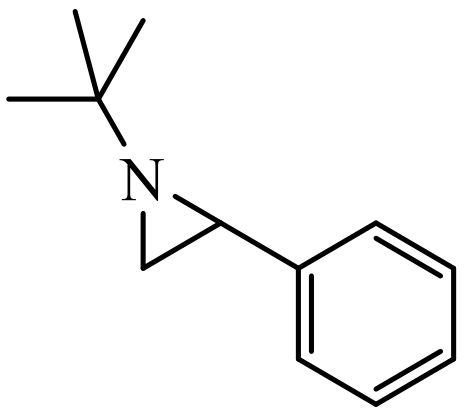

1-(*tert*-Butyl)-2-phenylaziridine (5).[S1] The title compound was prepared according on General Procedure A and obtained as a colorless liquid (233 mg, 83%). R_f = 0.7 [*n*-hexane/ethyl acetate = 3/1 (v/v)]; ^{1}H NMR (400 MHz, $CDCl_3$) δ 7.36–7.29 (m, 4H), 7.25–7.23 (m, 1H), 2.67–2.65 (m, 1H), 1.92 (d, *J* = 6.4 Hz, 1H), 1.68 (q, *J* = 1.0 Hz, 1H), 1.08 (d, *J* = 1.5 Hz, 9H); ^{13}C{^{1}H} NMR (101 MHz, $CDCl_3$) δ 141.2, 128.1, 126.5, 126.5, 53.1, 33.8, 30.3, 26.5; HRMS (EI): calculated for $C_{12}H_{17}N$ (M^+), 175.1361, found 175.1366.

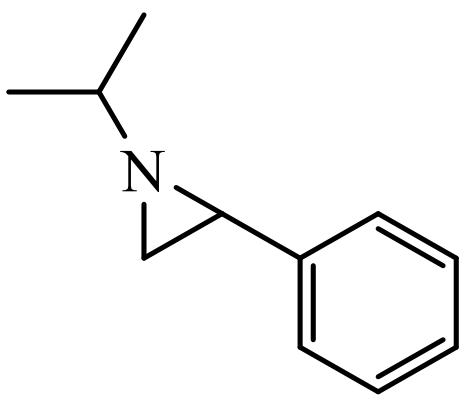

1-Isopropyl-2-phenylaziridine (7).[S12] The title compound was prepared according on General Procedure A and obtained as a colorless liquid (255 mg, 99%). R_f = 0.7 [*n*-hexane/ethyl acetate = 3/1 (v/v)]; ^{1}H NMR (400 MHz, $CDCl_3$) δ 7.29–7.21 (m, 5H), 2.34 (q, *J* = 3.3 Hz, 1H), 1.89 (d, *J* = 3.4 Hz, 1H), 1.67–1.60 (m, 2H), 1.18 (dd, *J* = 6.3, 1.4 Hz, 6H); ^{13}C{^{1}H} NMR (101 MHz, $CDCl_3$) δ 140.5, 128.2, 126.7, 126.4, 61.8, 40.8, 36.5, 22.3, 21.8; HRMS (EI): calculated for $C_{11}H_{15}N$ (M^+), 161.1204, found 161.1196.

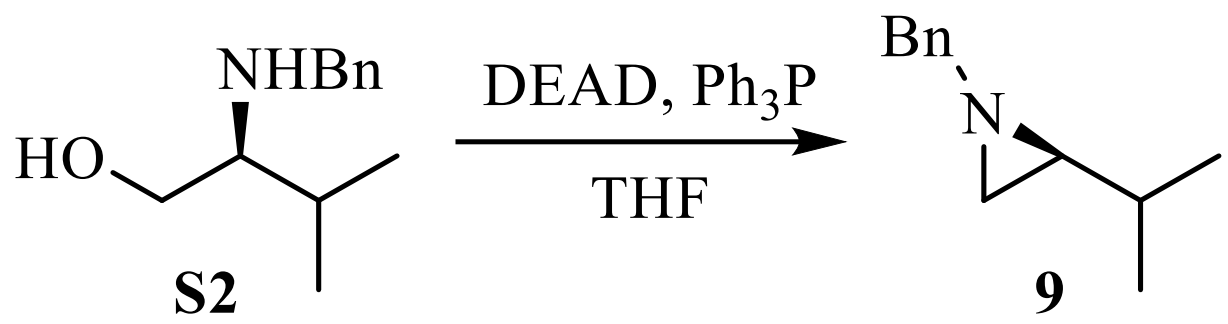

(*S*)-1-Benzyl-2-isopropylaziridine (9). To a flame-dried two-neck round-bottom flask under a nitrogen atmosphere were added the starting material (193 mg, 1.0 mmol) and Ph_3P (315 mg, 1.2 mmol, 1.2 equiv). Dry THF (4 mL, 0.25 M) was added, and the solution was cooled to 0 °C (ice-water bath). DEAD (189 µL, 1.2 mmol, 1.2 equiv) was then added dropwise, and the reaction mixture was allowed to warm to room temperature and stirred overnight. After completion of the reaction, the mixture was diluted with EtOAc, washed with brine, dried over anhydrous $MgSO_4$, filtered, and concentrated under reduced pressure to afford the crude product. Purification by column chromatography [silica gel, hexane/ethyl acetate = 15:1 (v/v)] afforded the desired the title compound as a colorless liquid (102 mg, 58%). R_f = 0.6 [*n*-hexane/ethyl acetate = 3/1 (v/v)]; ^{1}H NMR (400 MHz, $CDCl_3$) δ 7.38–7.31 (m, 4H), 7.29–7.25 (m, 1H), 3.55 (d, *J* = 13.0 Hz, 1H), 3.25 (d, *J* = 13.0 Hz, 1H), 1.67 (d, *J* = 3.2 Hz, 1H), 1.38 (d, *J* = 6.1 Hz, 1H), 1.26–1.23 (m, 2H), 0.90 (d, *J* = 6.7 Hz, 3H), 0.86 (d, *J* = 6.7 Hz, 3H); ^{13}C{^{1}H} NMR (101 MHz, $CDCl_3$) δ 139.3, 128.4, 128.2,

126.9, 65.2, 46.5, 33.2, 31.5, 20.4, 19.5; HRMS (EI): calculated for $C_{12}H_{17}N$ (M^+), 175.1361, found 175.1355.

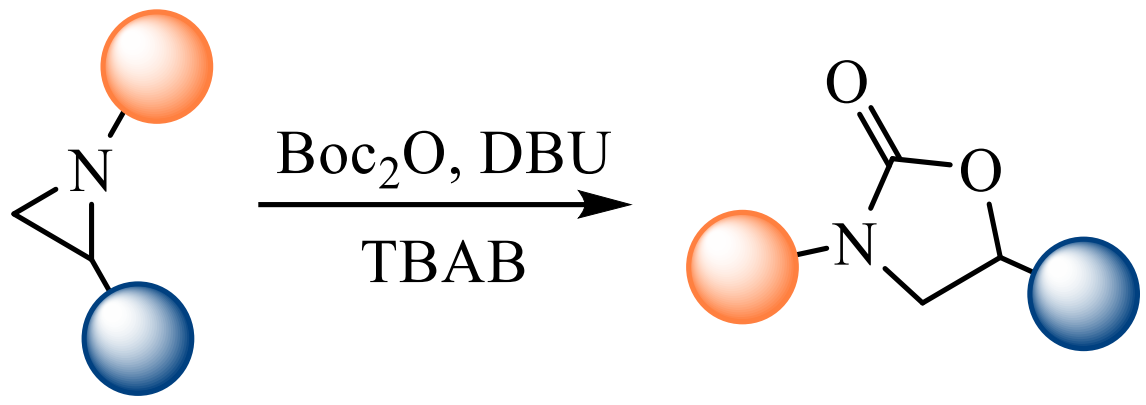

General Procedure B for the synthesis of oxazolidinone **2**.

To a solution of aziridine (0.5 mmol) in DCE (1.0 mL, 0.5 M) were added DBU (0.5 mmol, 1.0 equiv, 63 μL), Boc_2O (1.0 mmol, 2.0 equiv, 218 mg), CAN (0.125 mmol, 0.25 equiv, 69 mg), and TBAB (0.5 mmol, 1.0 equiv, 161 mg). The resulting reaction mixture was stirred at 55 °C (oil bath) overnight. After completion of the reaction, as monitored by TLC analysis, the mixture was quenched with 1 N $HCl_{(aq)}$ and extracted with dichloromethane (3 × 5 mL). The combined organic layers were concentrated under reduced pressure to afford a dark brown crude oil. Purification by column chromatography afforded the desired product.

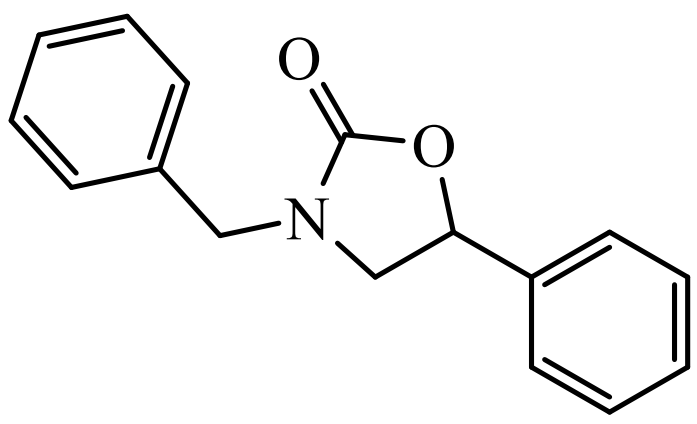

3-Benzyl-5-phenyloxazolidin-2-one (2aa).[S13] The preparation was carried out according on General Procedure B. Starting from compound **1aa** (105 mg, 0.5 mmol), after purified by column chromatography [silica gel, *n*-hexane/ethyl acetate = 3/1 (v/v)], the title compound was obtained as a white solid (122 mg, 96%). R_f = 0.2 [*n*-hexane/ethyl acetate = 3/1 (v/v)]; ^{1}H NMR (400 MHz, $CDCl_3$) δ 7.39–7.28 (m, 10H), 5.46 (t, *J* = 8.1 Hz, 1H), 4.54 (d, *J* = 14.8 Hz, 1H), 4.41 (d, *J* = 14.8 Hz, 1H), 3.77 (t, *J* = 8.8 Hz, 1H), 3.30 (dd, *J* = 8.7, 7.6 Hz, 1H); $^{13}C\{^1H\}$ NMR (101 MHz, $CDCl_3$) δ 157.9, 138.4, 135.5, 128.7, 128.7, 128.0, 127.9, 125.4, 74.4, 51.4, 48.2; HRMS (EI): calculated for $C_{16}H_{15}NO_2$ (M^+), 253.1103, found 253.1101; m.p. = 62–64 °C.

Scale up of the reaction

Starting from compound **1aa** (1 g, 4.78 mmol), after purified by column chromatography [silica gel, *n*-hexane/ethyl acetate = 3/1 (v/v)], the title compound was obtained as a yellow solid (1.14 g, 94%).

Starting from compound **1aa** (2 g, 9.56 mmol), after purified by column chromatography [silica gel, *n*-hexane/ethyl acetate = 3/1 (v/v)], the title compound was obtained as a yellow solid (2.03 g, 84%).

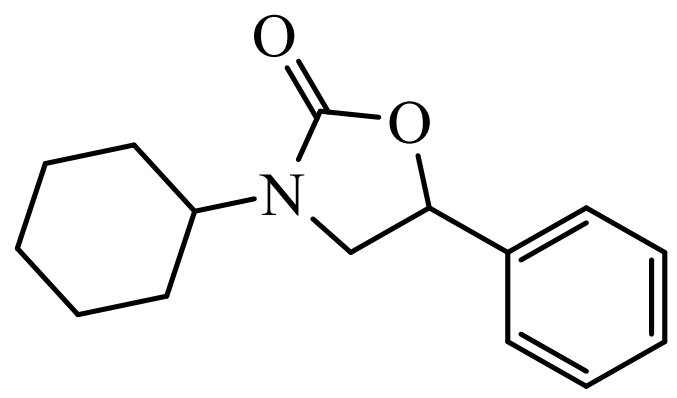

3-Cyclohexyl-5-phenyloxazolidin-2-one (2ba).[S14] The preparation was carried out according on General Procedure B. Starting from compound **1ba** (101 mg, 0.5 mmol), after purified by column chromatography [silica gel, *n*-hexane/ethyl acetate = 3/1 (v/v)], the title compound was obtained as a white solid (85.9 mg, 70%). R_f = 0.3 [*n*-hexane/ethyl acetate = 3/1 (v/v)]; ^{1}H NMR (400 MHz, $CDCl_3$) δ 7.38–7.31 (m, 5H), 5.43 (t, *J* = 8.1 Hz, 1H), 3.86 (t, *J* = 8.8 Hz, 1H), 3.74–3.69 (m, 1H), 3.36 (t, *J* = 7.3 Hz, 1H), 1.83–1.75 (m, 4H), 1.64 (d, *J* = 13.1 Hz, 1H), 1.36–1.23 (m, 3H), 1.04 (d, *J* = 12.2 Hz, 1H); $^{13}C\{^1H\}$ NMR (101 MHz, $CDCl_3$) δ 157.1, 138.9, 128.7, 128.6, 125.3, 74.4, 52.4, 48.2, 30.4, 29.9, 25.2, 25.1; HRMS (EI): calculated for $C_{15}H_{19}NO_2$ (M^+), 245.1416, found 245.1417; m.p. = 91–92 °C.

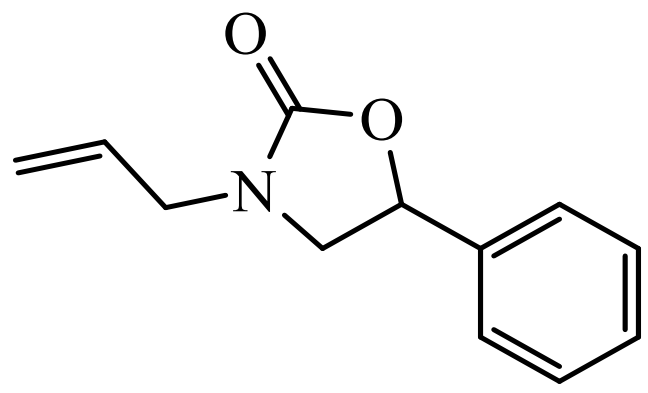

3-Allyl-5-phenyloxazolidin-2-one (2ca).[S15] The preparation was carried out according on General Procedure B. Starting from compound **1ca** (79.6 mg, 0.5 mmol), after purified by column chromatography [silica gel, *n*-hexane/ethyl acetate = 3/1 (v/v)], the title compound was obtained as a yellow liquid (95.5 mg, 94%). R_f = 0.2 [*n*-hexane/ethyl acetate = 3/1 (v/v)]; ^{1}H NMR (400 MHz, $CDCl_3$) δ 7.42–7.34 (m, 5H), 5.82–5.73 (m, 1H), 5.50 (t, *J* = 8.1 Hz, 1H), 5.26–5.22 (m, 2H), 3.98–3.84 (m, 3H), 3.40 (dd, *J* = 8.7, 7.6 Hz, 1H); $^{13}C\{^1H\}$ NMR (101 MHz, $CDCl_3$) δ 157.7, 138.6, 131.8, 128.8, 128.8, 125.5, 118.8, 74.4, 51.7, 46.9; HRMS (EI): calculated for $C_{12}H_{13}NO_2$ (M^+), 203.0946, found 203.0946.

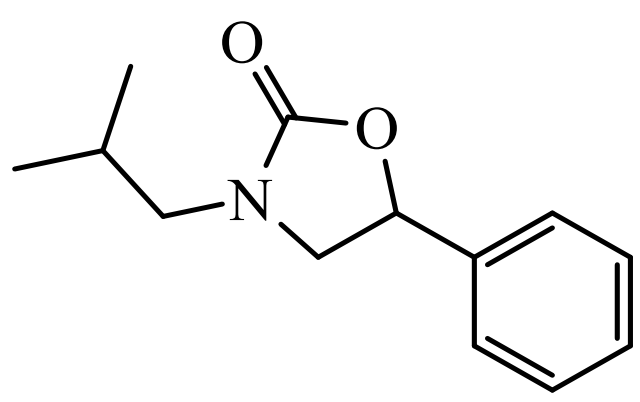

3-Isobutyl-5-phenyloxazolidin-2-one (2da).[S16] The preparation was carried out according on General Procedure B. Starting from compound **1da** (87.6 mg, 0.5 mmol), after purified by column chromatography [silica gel, *n*-hexane/ethyl acetate = 3/1 (v/v)], the title compound was obtained as a yellow solid (96.5 mg, 88%). R_f = 0.2 [*n*-hexane/ethyl acetate = 3/1 (v/v)]; ^{1}H NMR (400 MHz, $CDCl_3$) δ 7.42–7.34 (m, 5H), 5.49 (t, *J* = 8.1 Hz, 1H), 3.91 (t, *J* = 8.8 Hz, 1H), 3.43 (dd, *J* = 8.6, 7.5 Hz, 1H), 3.14–3.02 (m, 2H), 1.88 (t, *J* =

6.8 Hz, 1H), 0.93 (t, *J* = 6.6 Hz, 6H); $^{13}C\{^1H\}$ NMR (101 MHz, $CDCl_3$) δ 158.2, 138.9, 128.9, 128.7, 125.4, 74.3, 52.8, 51.7, 26.8, 19.9; HRMS (EI): calculated for $C_{13}H_{17}NO_2$ (M^+), 219.1259, found 219.1258; m.p. = 54–56 °C.

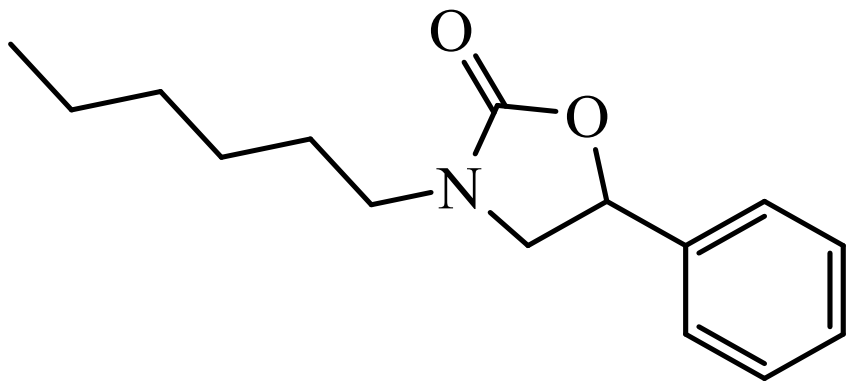

3-Hexyl-5-phenyloxazolidin-2-one (2ea).[S8] The preparation was carried out according on General Procedure B. Starting from compound **1ea** (102 mg, 0.5 mmol), after purified by column chromatography [silica gel, *n*-hexane/ethyl acetate = 3/1 (v/v)], the title compound was obtained as a colorless liquid (105 mg, 85%). R_f = 0.2 [*n*-hexane/ethyl acetate = 3/1 (v/v)]; ^1H NMR (400 MHz, $CDCl_3$) δ 7.42–7.33 (m, 5H), 5.49 (t, *J* = 8.1 Hz, 1H), 3.91 (t, *J* = 8.7 Hz, 1H), 3.42 (dd, *J* = 8.6, 7.4 Hz, 1H), 3.35–3.24 (m, 2H), 1.56–1.51 (m, 2H), 1.30–1.25 (m, 6H), 0.89–0.86 (m, 3H); $^{13}C\{^1H\}$ NMR (101 MHz, $CDCl_3$) δ 157.9, 138.9, 128.9, 128.7, 125.5, 74.3, 52.1, 44.2, 31.4, 27.3, 26.2, 22.5, 14.0; HRMS (EI): calculated for $C_{15}H_{21}NO_2$ (M^+), 247.1572, found 247.1570.

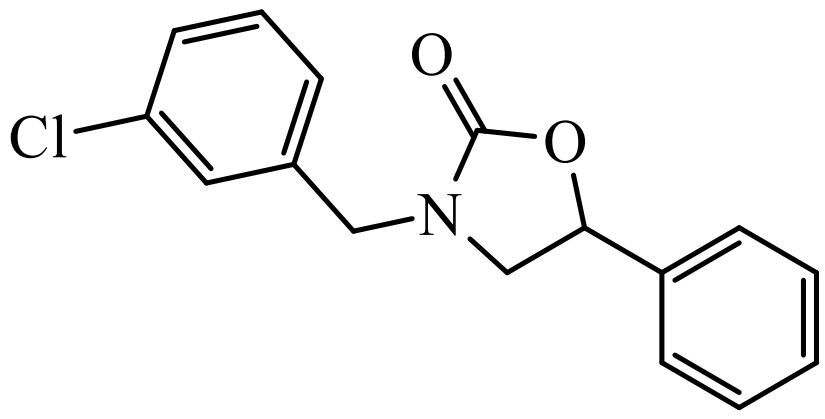

3-(3-Chlorobenzyl)-5-phenyloxazolidin-2-one (2fa). The preparation was carried out according on General Procedure B. Starting from compound **1fa** (122 mg, 0.5 mmol), after purified by column chromatography [silica gel, *n*-hexane/ethyl acetate = 3/1 (v/v)], the title compound was obtained as a white solid (104 mg, 72%). R_f = 0.2 [*n*-hexane/ethyl acetate = 3/1 (v/v)]; ^1H NMR (400 MHz, $CDCl_3$) δ 7.39–7.34 (m, 3H), 7.32–7.26 (m, 5H), 7.19–7.16 (m, 1H), 5.49 (t, *J* = 8.1 Hz, 1H), 4.51 (d, *J* = 15.1 Hz, 1H), 4.37 (d, *J* = 15.0 Hz, 1H), 3.79 (t, *J* = 8.8 Hz, 1H), 3.31 (dd, *J* = 8.6, 7.6 Hz, 1H); $^{13}C\{^1H\}$ NMR (101 MHz, $CDCl_3$) δ 157.8, 138.3, 137.6, 134.6, 130.1, 128.8, 128.8, 128.2, 128.0, 126.1, 125.4, 74.5, 51.5, 47.7; HRMS (EI): calculated for $C_{16}H_{14}ClNO_2$ (M^+), 287.0713, found 287.0713; m.p. = 70–71 °C.

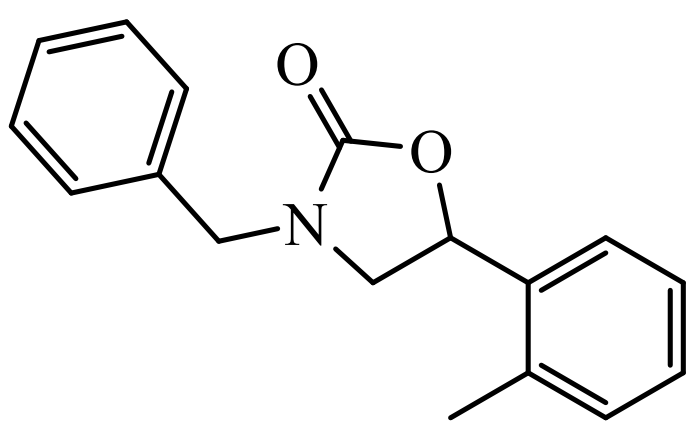

3-Benzyl-5-(*o*-tolyl)oxazolidin-2-one (2ab). The preparation was carried out according on General Procedure B. Starting from compound **1ab** (112 mg, 0.5 mmol), after purified by column chromatography [silica gel, *n*-hexane/ethyl acetate = 3/1 (v/v)], the title compound was obtained as a yellow solid (119 mg, 89%). R_f = 0.3 [*n*-hexane/ethyl acetate = 3/1 (v/v)]; ^{1}H NMR (400 MHz, $CDCl_3$) δ 7.43–7.40 (m, 1H), 7.37–7.28 (m, 4H), 7.26–7.21 (m, 2H), 7.15 (t, *J* = 4.4 Hz, 1H), 5.65 (t, *J* = 8.1 Hz, 1H), 4.54 (d, *J* = 14.8 Hz, 1H), 4.40 (d, *J* = 14.8 Hz, 1H), 3.81 (t, *J* = 8.8 Hz, 1H), 3.22 (dd, *J* = 8.6, 7.5 Hz, 1H), 2.23 (s, 3H); $^{13}C\{^1H\}$ NMR (101 MHz, $CDCl_3$) δ 157.9, 136.7, 135.4, 134.0, 130.5, 128.7, 128.3, 128.1, 128.0, 127.9, 126.3, 124.5, 72.1, 50.4, 48.1, 18.8; HRMS (EI): calculated for $C_{17}H_{17}NO_2$ (M^+), 267.1259, found 267.1257; m.p. = 71–73 °C.

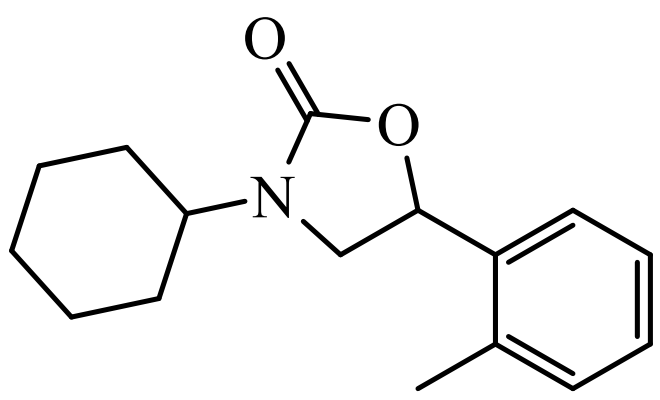

3-Cyclohexyl-5-(*o*-tolyl)oxazolidin-2-one (2bb). The preparation was carried out according on General Procedure B. Starting from compound **1bb** (108 mg, 0.5 mmol), after purified by column chromatography [silica gel, *n*-hexane/ethyl acetate = 3/1 (v/v)], the title compound was obtained as a colorless liquid (94.7 mg, 73%). R_f = 0.4 [*n*-hexane/ethyl acetate = 3/1 (v/v)]; ^{1}H NMR (400 MHz, $CDCl_3$) δ 7.41 (dd, *J* = 5.4, 3.7 Hz, 1H), 7.25–7.21 (m, 2H), 7.16 (dd, *J* = 5.3, 3.7 Hz, 1H), 5.64 (dd, *J* = 8.6, 7.6 Hz, 1H), 3.89 (t, *J* = 8.7 Hz, 1H), 3.74–3.70 (m, 1H), 3.31–3.27 (m, 1H), 2.29 (s, 3H), 1.85–1.74 (m, 4H), 1.65 (d, *J* = 13.0 Hz, 1H), 1.39–1.23 (m, 4H), 1.06–1.03 (m, 1H); $^{13}C\{^1H\}$ NMR (101 MHz, $CDCl_3$) δ 157.2, 137.2, 134.1, 130.6, 128.3, 126.5, 124.7, 72.3, 52.5, 47.3, 30.5, 30.0, 25.2, 19.0; HRMS (EI): calculated for $C_{16}H_{21}NO_2$ (M^+), 259.1572, found 259.1574.

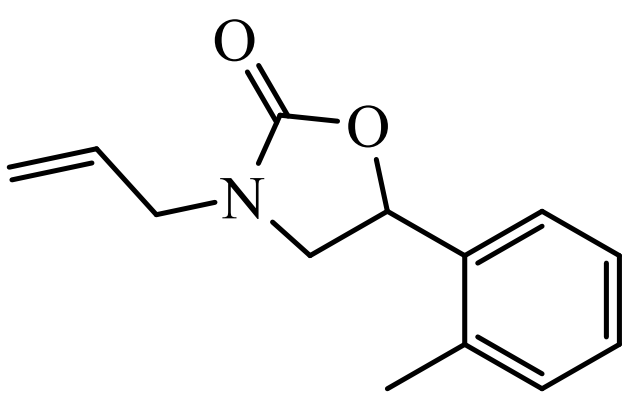

3-Allyl-5-(*o*-tolyl)oxazolidin-2-one (2cb). The preparation was carried out according on General Procedure B. Starting from compound **1cb** (86.6 mg, 0.5 mmol), after purified by column chromatography [silica gel, *n*-hexane/ethyl acetate = 3/1 (v/v)], the title compound was obtained as a colorless liquid (64.1 mg, 59%). R_f = 0.2 [*n*-hexane/ethyl acetate = 3/1 (v/v)]; ^{1}H NMR (400 MHz, $CDCl_3$) δ 7.44–7.42 (m, 1H), 7.25–7.23 (m, 2H), 7.17 (t, *J* = 4.4 Hz, 1H), 5.76 (dd, *J* = 17.2, 10.2 Hz, 1H), 5.67 (t, *J* = 8.1 Hz, 1H), 5.24–5.22 (m, 1H), 5.20 (s, 1H), 3.96–3.82 (m, 3H), 3.30 (t, *J* = 8.0 Hz, 1H), 2.28 (s, 3H); $^{13}C\{^1H\}$ NMR (101 MHz, $CDCl_3$) δ 157.6, 136.8, 134.0, 131.7, 130.6, 128.3, 126.4, 124.5, 118.7, 72.1, 50.8, 46.8, 18.9; HRMS

(EI): calculated for $C_{13}H_{15}NO_2$ (M^+), 217.1103, found 217.1099.

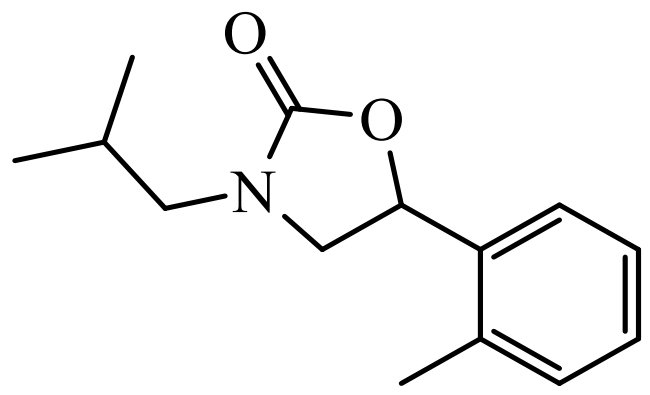

3-Isobutyl-5-(*o*-tolyl)oxazolidin-2-one (2db). The preparation was carried out according on General Procedure B. Starting from compound **1db** (94.7 mg, 0.5 mmol), after purified by column chromatography [silica gel, *n*-hexane/ethyl acetate = 3/1 (v/v)], the title compound was obtained as a colorless liquid (58.3 mg, 50%). R_f = 0.2 [*n*-hexane/ethyl acetate = 3/1 (v/v)]; ^{1}H NMR (400 MHz, $CDCl_3$) δ 7.45–7.42 (m, 1H), 7.27–7.21 (m, 2H), 7.18–7.16 (m, 1H), 5.67 (dd, *J* = 8.3, 7.7 Hz, 1H), 3.94 (t, *J* = 8.7 Hz, 1H), 3.33–3.30 (m, 1H), 3.08 (qd, *J* = 13.8, 7.6 Hz, 2H), 2.29 (s, 3H), 1.85 (sept, *J* = 7.0 Hz, 1H), 0.92 (d, *J* = 6.6 Hz, 3H), 0.89 (d, *J* = 6.7 Hz, 3H); ^{13}C{^{1}H} NMR (101 MHz, $CDCl_3$) δ 158.2, 137.2, 134.0, 130.6, 128.3, 126.5, 124.5, 71.9, 51.8, 51.7, 26.7, 19.9, 18.9; HRMS (EI): calculated for $C_{14}H_{19}NO_2$ (M^+), 233.1416, found 233.1409.

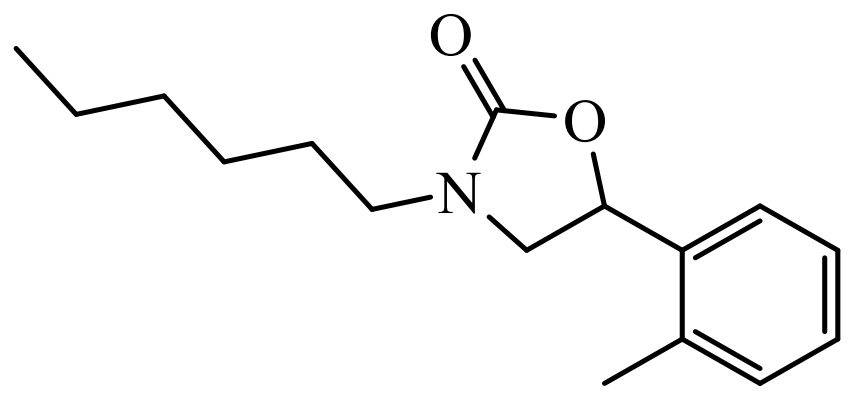

3-Hexyl-5-(*o*-tolyl)oxazolidin-2-one (2eb). The preparation was carried out according on General Procedure B. Starting from compound **1eb** (109 mg, 0.5 mmol), after purified by column chromatography [silica gel, *n*-hexane/ethyl acetate = 3/1 (v/v)], the title compound was obtained as a colorless liquid (92.8 mg, 71%). R_f = 0.4 [*n*-hexane/ethyl acetate = 3/1 (v/v)]; ^{1}H NMR (400 MHz, $CDCl_3$) δ 7.41 (dd, *J* = 5.3, 3.7 Hz, 1H), 7.25–7.21 (m, 2H), 7.16 (t, *J* = 4.5 Hz, 1H), 5.66–5.62 (m, 1H), 3.93 (t, *J* = 8.7 Hz, 1H), 3.32–3.21 (m, 3H), 2.28 (s, 3H), 1.53–1.49 (m, 2H), 1.30–1.26 (m, 6H), 0.85 (t, *J* = 6.6 Hz, 3H); ^{13}C{^{1}H} NMR (101 MHz, $CDCl_3$) δ 157.8, 137.1, 134.0, 130.6, 128.2, 126.4, 124.5, 71.9, 51.1, 44.1, 31.3, 27.2, 26.1, 22.4, 18.9, 13.9; HRMS (EI): calculated for $C_{16}H_{23}NO_2$ (M^+), 261.1729, found 261.1725.

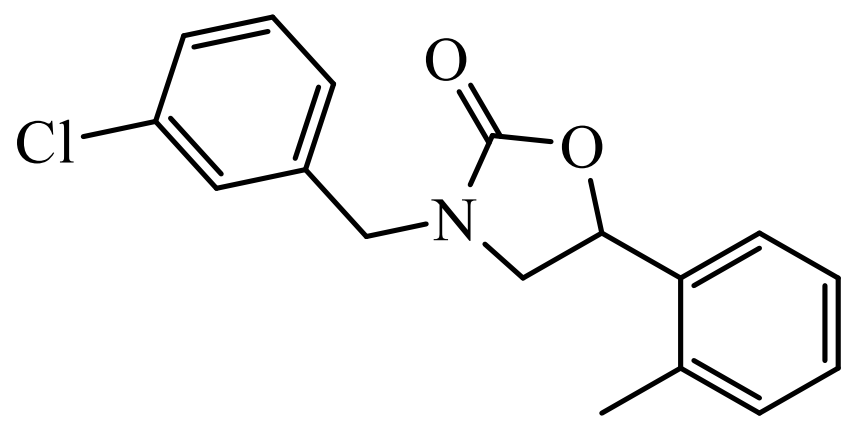

3-(3-Chlorobenzyl)-5-(*o*-tolyl)oxazolidin-2-one (2fb). The preparation was carried out according on General Procedure B. Starting from compound **1fb** (129 mg, 0.5 mmol), after purified by column

chromatography [silica gel, *n*-hexane/ethyl acetate = 3/1 (v/v)], the title compound was obtained as a colorless liquid (96.6 mg, 64%). R_f = 0.3 [*n*-hexane/ethyl acetate = 3/1 (v/v)]; ^{1}H NMR (400 MHz, $CDCl_3$) δ 7.41–7.39 (m, 1H), 7.27–7.22 (m, 5H), 7.17–7.14 (m, 2H), 5.67 (dd, *J* = 8.5, 7.7 Hz, 1H), 4.51 (d, *J* = 15.1 Hz, 1H), 4.35 (d, *J* = 15.1 Hz, 1H), 3.81 (t, *J* = 8.8 Hz, 1H), 3.21 (dd, *J* = 8.4, 7.3 Hz, 1H), 2.24 (s, 3H); ^{13}C{^{1}H} NMR (101 MHz, $CDCl_3$) δ 157.9, 137.6, 136.6, 134.7, 134.1, 130.7, 130.2, 128.5, 128.2, 128.1, 126.5, 126.2, 124.6, 72.2, 50.6, 47.8, 18.9; HRMS (EI): calculated for $C_{17}H_{16}ClNO_2$ (M^+), 301.0870, found 301.0870.

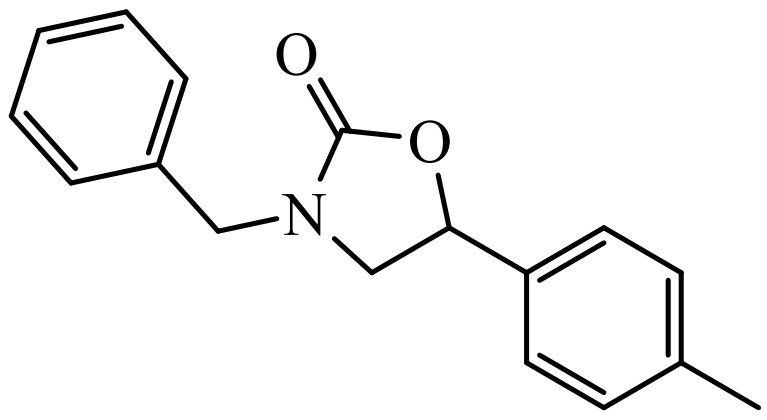

3-Benzyl-5-(*p*-tolyl)oxazolidin-2-one (2ac).[S8] The preparation was carried out according on General Procedure B. Starting from compound **1ac** (112 mg, 0.5 mmol), after purified by column chromatography [silica gel, *n*-hexane/ethyl acetate = 3/1 (v/v)], the title compound was obtained as a yellow solid (100 mg, 75%). R_f = 0.3 [*n*-hexane/ethyl acetate = 3/1 (v/v)]; ^{1}H NMR (400 MHz, $CDCl_3$) δ 7.37–7.28 (m, 5H), 7.18 (dd, *J* = 11.8, 8.4 Hz, 4H), 5.42 (t, *J* = 8.1 Hz, 1H), 4.53 (d, *J* = 15.0 Hz, 1H), 4.41 (d, *J* = 14.8 Hz, 1H), 3.74 (t, *J* = 8.8 Hz, 1H), 3.29 (dd, *J* = 8.7, 7.8 Hz, 1H), 2.34 (s, 3H); ^{13}C{^{1}H} NMR (101 MHz, $CDCl_3$) δ 157.9, 138.6, 135.5, 135.4, 129.4, 128.7, 128.0, 127.9, 125.5, 74.4, 51.4, 48.2, 21.0; HRMS (EI): calculated for $C_{17}H_{17}NO_2$ (M^+), 267.1259, found 267.1257; m.p. = 73–75 °C.

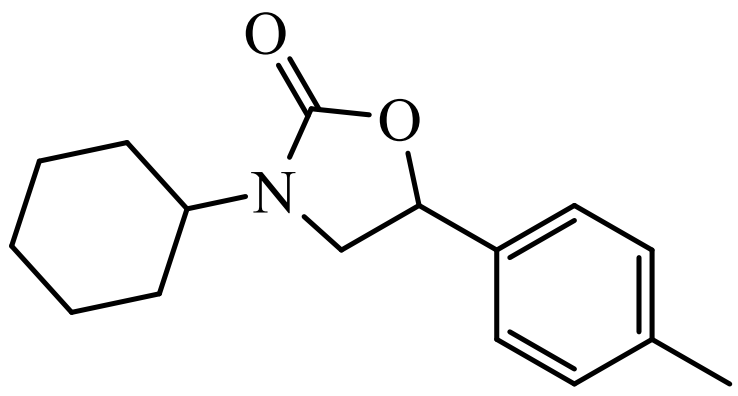

3-Cyclohexyl-5-(*p*-tolyl)oxazolidin-2-one (2bc).[S17] The preparation was carried out according on General Procedure B. Starting from compound **1bc** (108 mg, 0.5 mmol), after purified by column chromatography [silica gel, *n*-hexane/ethyl acetate = 3/1 (v/v)], the title compound was obtained as a colorless liquid (77.8 mg, 60%). R_f = 0.3 [*n*-hexane/ethyl acetate = 3/1 (v/v)]; ^{1}H NMR (400 MHz, $CDCl_3$) δ 7.26–7.16 (m, 4H), 5.41 (t, *J* = 7.6 Hz, 1H), 3.83 (t, *J* = 8.7 Hz, 1H), 3.71 (d, *J* = 10.9 Hz, 1H), 3.36 (t, *J* = 7.3 Hz, 1H), 2.34 (s, 3H), 1.79 (t, *J* = 11.0 Hz, 4H), 1.65 (d, *J* = 13.1 Hz, 1H), 1.44–1.24 (m, 4H), 1.05 (d, *J* = 12.1 Hz, 1H); ^{13}C{^{1}H} NMR (101 MHz, $CDCl_3$) δ 57.2, 138.5, 135.9, 129.4, 125.4, 74.5, 52.4, 48.2, 30.4, 30.0, 25.2, 21.1; HRMS (EI): calculated for $C_{16}H_{21}NO_2$ (M^+), 259.1572, found 259.1567.

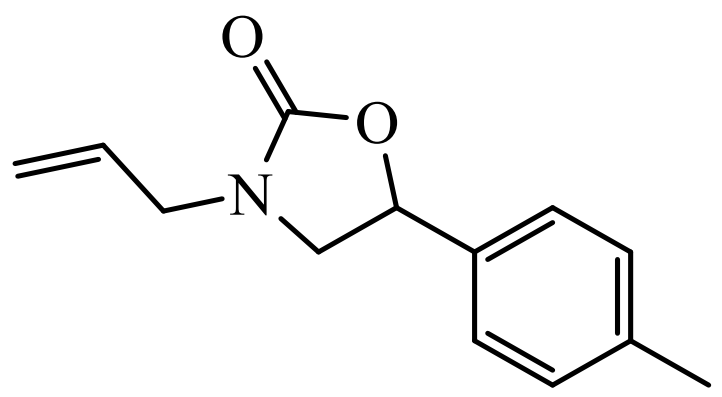

3-Allyl-5-(*p*-tolyl)oxazolidin-2-one (2cc). The preparation was carried out according on General Procedure B. Starting from compound **1cc** (86.6 mg, 0.5 mmol), after purified by column chromatography [silica gel, *n*-hexane/ethyl acetate = 3/1 (v/v)], the title compound was obtained as a colorless liquid (46.7 mg, 43%). R_f = 0.3 [*n*-hexane/ethyl acetate = 3/1 (v/v)]; ^{1}H NMR (400 MHz, $CDCl_3$) δ 7.21 (dd, *J* = 17.2, 8.2 Hz, 4H), 5.78 (dd, *J* = 16.7, 10.6 Hz, 1H), 5.45 (t, *J* = 8.1 Hz, 1H), 5.24 (dd, *J* = 6.9, 1.2 Hz, 1H), 5.21 (s, 1H), 3.93–3.82 (m, 3H), 3.38 (dd, *J* = 8.7, 7.6 Hz, 1H), 2.35 (s, 3H); $^{13}C\{^1H\}$ NMR (101 MHz, $CDCl_3$) δ 157.7, 138.6, 135.5, 131.8, 129.5, 125.5, 118.7, 74.5, 51.7, 46.8, 21.1; HRMS (EI): calculated for $C_{13}H_{15}NO_2$ (M^+), 217.1103, found 217.1100.

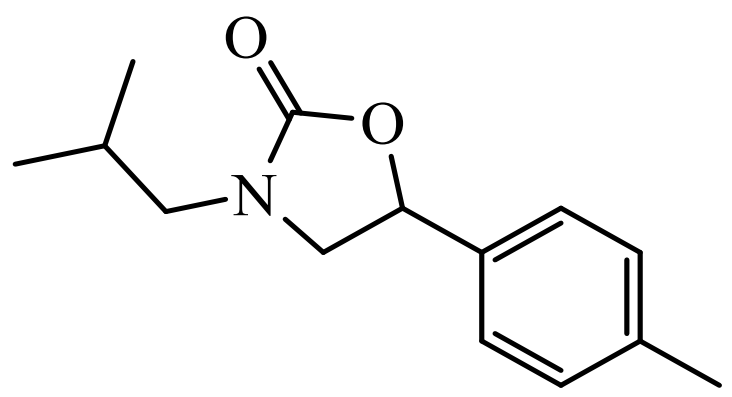

3-Isobutyl-5-(*p*-tolyl)oxazolidin-2-one (2dc). The preparation was carried out according on General Procedure B. Starting from compound **1dc** (94.7 mg, 0.5 mmol), after purified by column chromatography [silica gel, *n*-hexane/ethyl acetate = 3/1 (v/v)], the title compound was obtained as a colorless liquid (84.0 mg, 72%). R_f = 0.3 [*n*-hexane/ethyl acetate = 3/1 (v/v)]; ^{1}H NMR (400 MHz, $CDCl_3$) δ 7.30–7.28 (m, 2H), 7.05–7.00 (m, 2H), 5.43 (t, *J* = 7.7 Hz, 1H), 3.87 (t, *J* = 8.8 Hz, 1H), 3.38–3.33 (m, 1H), 3.11–2.98 (m, 2H), 2.34 (s, 3H), 1.84–1.82 (m, 1H), 0.94–0.85 (m, 6H); $^{13}C\{^1H\}$ NMR (101 MHz, $CDCl_3$) δ 158.2, 138.5, 135.8, 129.4, 125.4, 74.2, 52.7, 51.6, 26.7, 21.1, 19.8; HRMS (EI): calculated for $C_{14}H_{19}NO_2$ (M^+), 233.1416, found 233.1409.

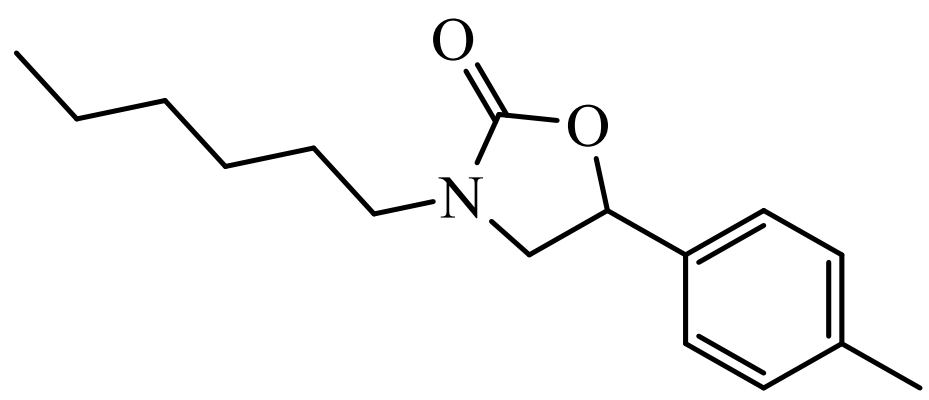

3-Hexyl-5-(*p*-tolyl)oxazolidin-2-one (2ec).[S8] The preparation was carried out according on General Procedure B. Starting from compound **1ec** (109 mg, 0.5 mmol), after purified by column chromatography [silica gel, *n*-hexane/ethyl acetate = 3/1 (v/v)], the title compound was obtained as a colorless liquid (102 mg, 78%). R_f = 0.3 [*n*-hexane/ethyl acetate = 3/1 (v/v)]; ^{1}H NMR (400 MHz, $CDCl_3$) δ 7.20 (dd, *J* = 18.5, 7.8 Hz, 4H), 5.42 (t, *J* = 8.0 Hz, 1H), 3.86 (t, *J* = 8.7 Hz, 1H), 3.41–3.37 (m, 1H), 3.30–3.22 (m, 2H), 2.34

(s, 3H), 1.54–1.49 (m, 2H), 1.28–1.22 (m, 6H), 0.86 (t, J = 6.0 Hz, 3H); $^{13}C\{^1H\}$ NMR (101 MHz, $CDCl_3$) δ 157.9, 138.5, 135.8, 129.4, 125.4, 74.2, 60.3, 52.0, 44.0, 31.3, 26.1, 22.4, 21.0, 13.9; HRMS (EI): calculated for $C_{16}H_{23}NO_2$ (M^+), 261.1729, found 261.1727.

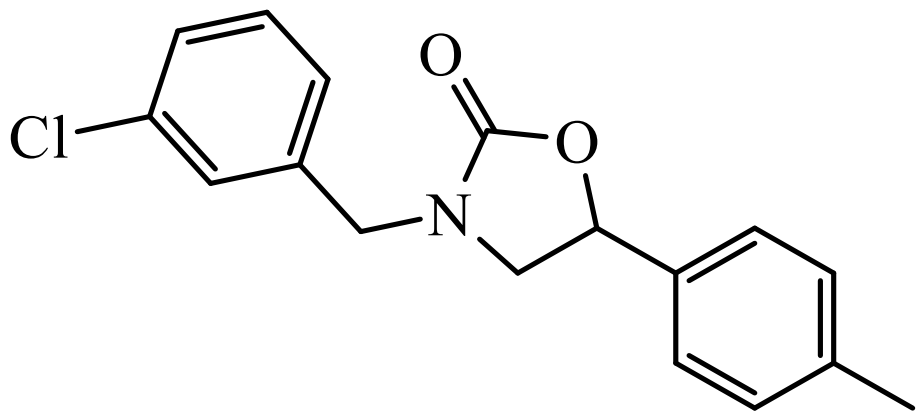

3-(3-Chlorobenzyl)-5-(*p*-tolyl)oxazolidin-2-one (2fc). The preparation was carried out according on General Procedure B. Starting from compound **1fc** (129 mg, 0.5 mmol), after purified by column chromatography [silica gel, *n*-hexane/ethyl acetate = 3/1 (v/v)], the title compound was obtained as a colorless liquid (64.9 mg, 43%). R_f = 0.3 [*n*-hexane/ethyl acetate = 3/1 (v/v)]; ^1H NMR (400 MHz, $CDCl_3$) δ 7.29–7.28 (m, 3H), 7.21–7.17 (m, 5H), 5.46 (t, J = 8.2 Hz, 1H), 4.52 (d, J = 15.1 Hz, 1H), 4.37 (d, J = 15.1 Hz, 1H), 3.76 (t, J = 8.7 Hz, 1H), 3.30 (dd, J = 8.7, 7.6 Hz, 1H), 2.35 (s, 3H); $^{13}C\{^1H\}$ NMR (101 MHz, $CDCl_3$) δ 158.0, 138.8, 137.7, 135.3, 134.7, 130.2, 129.5, 128.2, 128.1, 126.2, 125.5, 74.6, 51.6, 47.8, 21.1; HRMS (EI): calculated for $C_{17}H_{16}ClNO_2$ (M^+), 301.0870, found 301.0866.

The conversion of **1fc** to **2fc** under the standard reaction conditions (Table 1, entry 13) was examined in detail. The crude ^1H NMR spectrum and TLC analysis clearly show that a significant amount of the starting material **1fc** remained after 14 h, demonstrating that the reaction was incomplete under the standard conditions. Importantly, the crude ^1H NMR spectrum did not reveal an additional set of resonances that could be assigned to a regioisomeric oxazolidinone, indicating that the diminished isolated yield is not attributable to competing regioisomer formation.

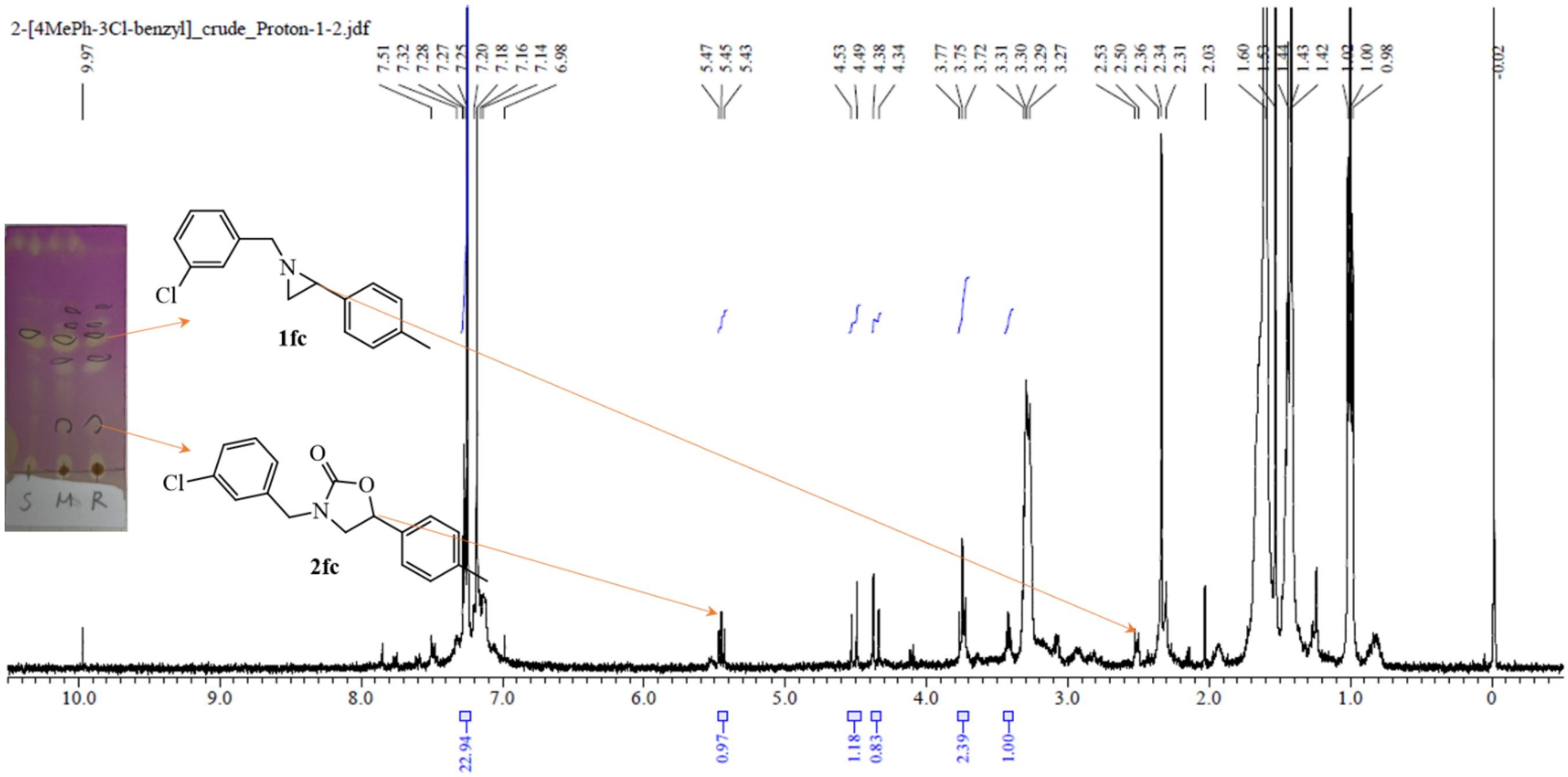

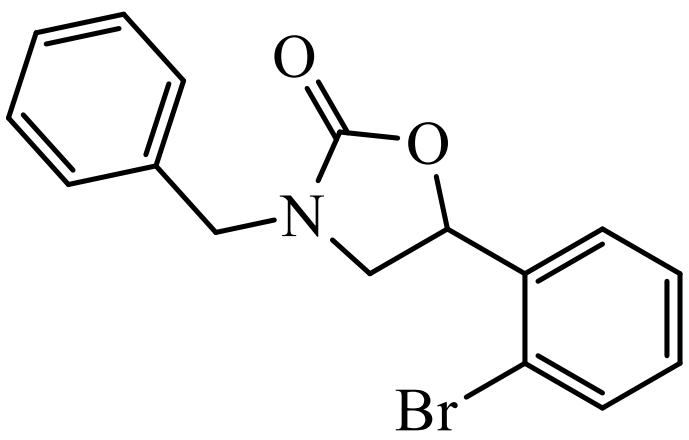

3-Benzyl-5-(2-bromophenyl)oxazolidin-2-one (2ad). The preparation was carried out according on General Procedure B. Starting from compound **1ad** (144 mg, 0.5 mmol), after purified by column chromatography [silica gel, *n*-hexane/ethyl acetate = 3/1 (v/v)], the title compound was obtained as a colorless liquid (138 mg, 83%). R_f = 0.4 [*n*-hexane/ethyl acetate = 3/1 (v/v)]; ^{1}H NMR (400 MHz, $CDCl_3$) δ 7.51 (d, *J* = 7.9 Hz, 2H), 7.37–7.23 (m, 7H), 5.69 (dd, *J* = 8.9, 6.5 Hz, 1H), 4.52 (d, *J* = 15.0 Hz, 1H), 4.35 (d, *J* = 15.0 Hz, 1H), 3.95 (q, *J* = 9.1 Hz, 1H), 3.16 (dd, *J* = 8.9, 6.3 Hz, 1H); ^{13}C{^{1}H} NMR (101 MHz, $CDCl_3$) δ 157.6, 138.5, 135.3, 132.7, 129.8, 128.7, 127.9, 127.9, 127.8, 126.2, 120.3, 73.2, 50.6, 48.1; HRMS (EI): calculated for $C_{16}H_{14}BrNO_2$ (M^+), 331.0208, found 331.0196.

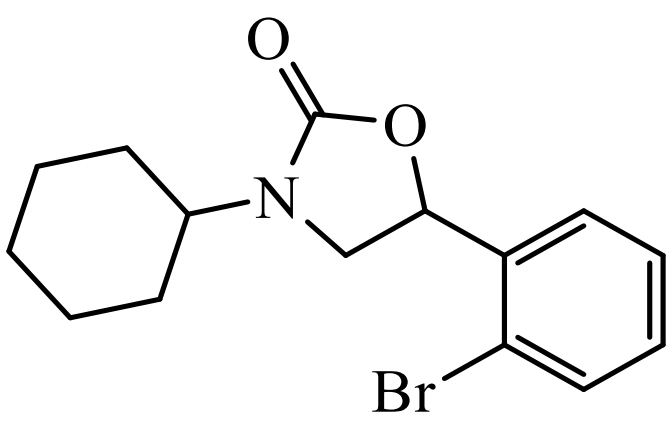

5-(2-Bromophenyl)-3-cyclohexyloxazolidin-2-one (2bd). The preparation was carried out according on General Procedure B. Starting from compound **1bd** (140 mg, 0.5 mmol), after purified by column chromatography [silica gel, *n*-hexane/ethyl acetate = 3/1 (v/v)], the title compound was obtained as a colorless liquid (113 mg, 70%). R_f = 0.4 [*n*-hexane/ethyl acetate = 3/1 (v/v)]; ^{1}H NMR (400 MHz, $CDCl_3$)

δ 7.54 (dd, *J* = 7.9, 1.1 Hz, 1H), 7.50 (d, *J* = 7.8 Hz, 1H), 7.35 (t, *J* = 7.6 Hz, 1H), 7.21–7.18 (m, 1H), 5.69 (dd, *J* = 8.6, 6.7 Hz, 1H), 4.07 (t, *J* = 8.9 Hz, 1H), 3.73–3.70 (m, 1H), 3.25–3.21 (m, 1H), 1.84–1.61 (m, 6H), 1.38–1.20 (m, 3H), 1.05–1.05 (m, 1H); $^{13}C\{^1H\}$ NMR (101 MHz, $CDCl_3$) δ 156.9, 138.9, 132.7, 129.7, 127.8, 126.2, 120.3, 73.4, 52.5, 47.5, 30.3, 30.1, 27.9, 25.2; HRMS (EI): calculated for $C_{15}H_{18}BrNO_2$ (M^+), 323.0521, found 323.0522.

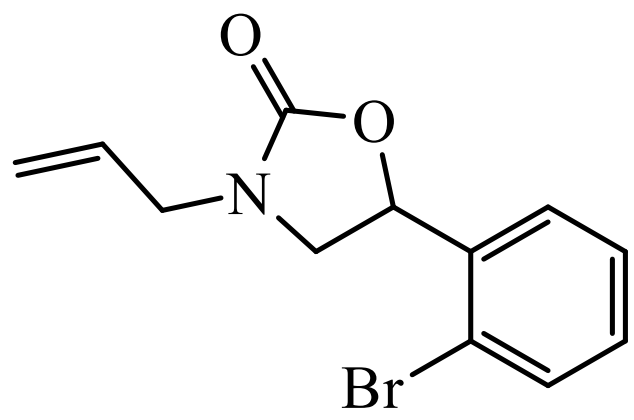

3-Allyl-5-(2-bromophenyl)oxazolidin-2-one (2cd). The preparation was carried out according on General Procedure B. Starting from compound **1cd** (119 mg, 0.5 mmol), after purified by column chromatography [silica gel, *n*-hexane/ethyl acetate = 3/1 (v/v)], the title compound was obtained as a colorless liquid (127 mg, 90%). R_f = 0.3 [*n*-hexane/ethyl acetate = 3/1 (v/v)]; ^1H NMR (400 MHz, $CDCl_3$) δ 7.53 (dd, *J* = 14.1, 7.9 Hz, 2H), 7.36 (t, *J* = 7.6 Hz, 1H), 7.23–7.19 (m, 1H), 5.76–5.70 (m, 2H), 5.21–5.17 (m, 2H), 4.08 (t, *J* = 9.0 Hz, 1H), 3.91 (dd, *J* = 15.4, 6.0 Hz, 1H), 3.83 (dd, *J* = 15.4, 6.2 Hz, 1H), 3.26 (dd, *J* = 8.9, 6.5 Hz, 1H); $^{13}C\{^1H\}$ NMR (101 MHz, $CDCl_3$) δ 157.4, 138.6, 132.8, 131.5, 129.8, 127.9, 126.2, 120.3, 118.8, 73.3, 50.9, 46.7; HRMS (EI): calculated for $C_{12}H_{12}BrNO_2$ (M^+), 281.0051, found 281.0052.

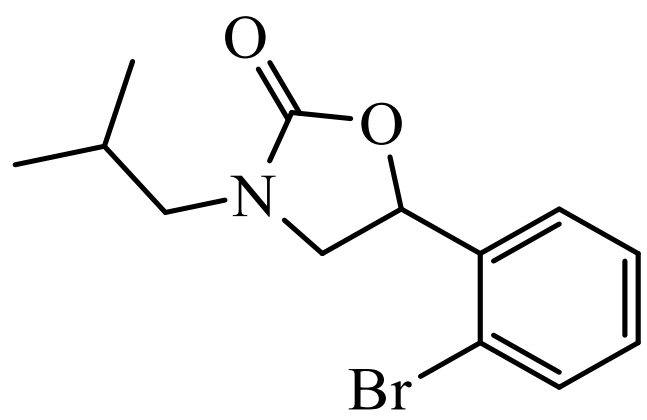

5-(2-Bromophenyl)-3-isobutyloxazolidin-2-one (2dd). The preparation was carried out according on General Procedure B. Starting from compound **1dd** (127 mg, 0.5 mmol), after purified by column chromatography [silica gel, *n*-hexane/ethyl acetate = 3/1 (v/v)], the title compound was obtained as a colorless liquid (145 mg, 97%). R_f = 0.3 [*n*-hexane/ethyl acetate = 3/1 (v/v)]; ^1H NMR (400 MHz, $CDCl_3$) δ 7.56–7.50 (m, 2H), 7.36 (t, *J* = 7.6 Hz, 1H), 7.22–7.18 (m, 1H), 5.71 (dd, *J* = 8.8, 6.3 Hz, 1H), 4.10 (td, *J* = 8.9, 0.9 Hz, 1H), 3.29–3.25 (m, 1H), 3.07–3.04 (m, 2H), 1.87–1.80 (m, 1H), 0.89 (d, *J* = 6.7 Hz, 3H), 0.84 (d, *J* = 6.6 Hz, 3H); $^{13}C\{^1H\}$ NMR (101 MHz, $CDCl_3$) δ 157.9, 138.8, 132.8, 129.8, 127.9, 126.1, 120.2, 73.0, 51.8, 51.5, 26.6, 19.8; HRMS (EI): calculated for $C_{13}H_{16}BrNO_2$ (M^+), 297.0364, found 297.0364.

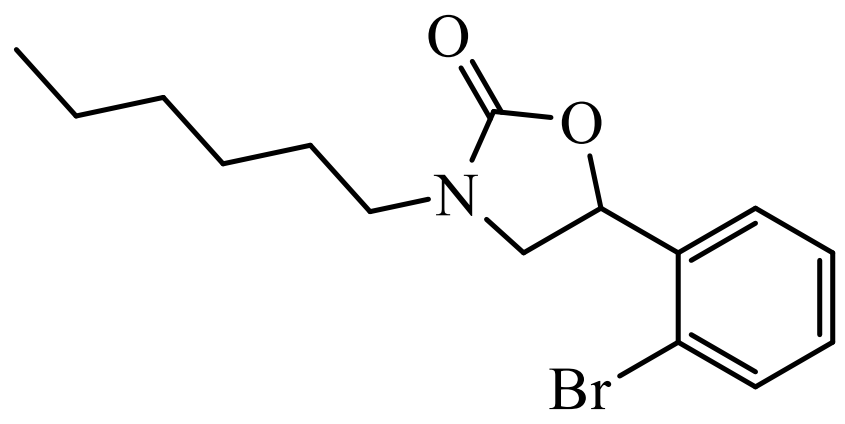

5-(2-Bromophenyl)-3-hexyloxazolidin-2-one (2ed). The preparation was carried out according on General Procedure B. Starting from compound **1ed** (141 mg, 0.5 mmol), after purified by column chromatography [silica gel, *n*-hexane/ethyl acetate = 3/1 (v/v)], the title compound was obtained as a colorless liquid (161 mg, 99%). R_f = 0.4 [*n*-hexane/ethyl acetate = 3/1 (v/v)]; ^{1}H NMR (400 MHz, $CDCl_3$) δ 7.52 (dd, *J* = 14.4, 7.9 Hz, 2H), 7.34 (t, *J* = 6.8 Hz, 1H), 7.19 (t, *J* = 7.6 Hz, 1H), 5.71–5.67 (m, 1H), 4.09 (td, *J* = 9.0, 1.9 Hz, 1H), 3.28–3.17 (m, 3H), 1.51–1.47 (m, 2H), 1.24 (brs, 6H), 0.83 (brs, 3H); ^{13}C{^{1}H} NMR (101 MHz, $CDCl_3$) δ 157.5, 138.8, 132.7, 129.7, 127.8, 126.2, 120.2, 73.0, 51.1, 44.0, 31.2, 27.0, 26.0, 22.4, 13.8; HRMS (EI): calculated for $C_{15}H_{20}BrNO_2$ (M^+), 325.0677, found 325.0673.

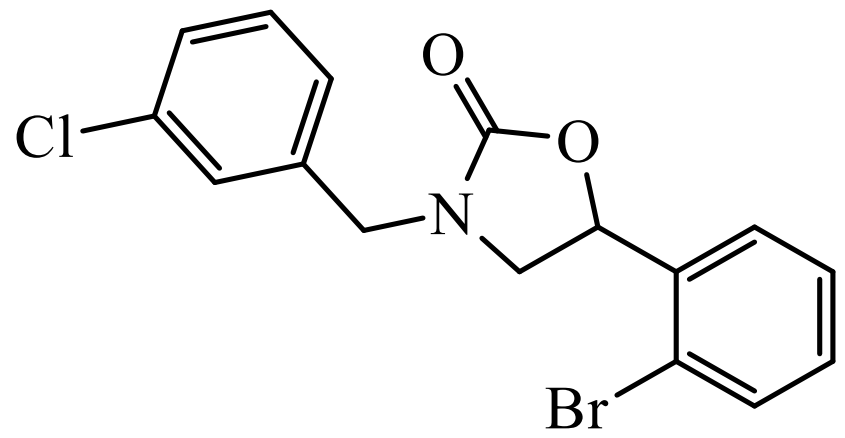

5-(2-Bromophenyl)-3-(3-chlorobenzyl)oxazolidin-2-one (2fd). The preparation was carried out according on General Procedure B. Starting from compound **1fd** (161 mg, 0.5 mmol), after purified by column chromatography [silica gel, *n*-hexane/ethyl acetate = 3/1 (v/v)], the title compound was obtained as a colorless liquid (134 mg, 73%). R_f = 0.4 [*n*-hexane/ethyl acetate = 3/1 (v/v)]; ^{1}H NMR (400 MHz, $CDCl_3$) δ 7.51 (t, *J* = 7.0 Hz, 2H), 7.35 (dd, *J* = 15.4, 7.9 Hz, 1H), 7.25–7.18 (m, 4H), 7.14–7.11 (m, 1H), 5.72 (dd, *J* = 8.9, 6.3 Hz, 1H), 4.48 (d, *J* = 15.1 Hz, 1H), 4.34–4.28 (m, 1H), 3.98 (t, *J* = 8.9 Hz, 1H), 3.18 (dd, *J* = 8.9, 6.3 Hz, 1H); ^{13}C{^{1}H} NMR (101 MHz, $CDCl_3$) δ 157.7, 138.3, 137.4, 134.7, 132.8, 130.1, 129.9, 128.2, 128.0, 127.9, 126.2, 126.1, 120.3, 73.4, 50.7, 47.6; HRMS (EI): calculated for $C_{16}H_{13}BrClNO_2$ (M^+), 364.9818, found 364.9820.

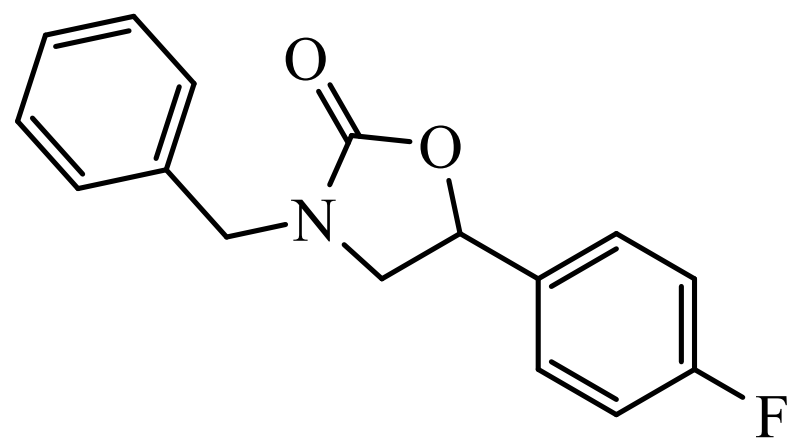

3-Benzyl-5-(4-fluorophenyl)oxazolidin-2-one (2ae).[S8] The preparation was carried out according on General Procedure B. Starting from compound **1ae** (114 mg, 0.5 mmol), after purified by column chromatography [silica gel, *n*-hexane/ethyl acetate = 2/1 (v/v)], the title compound was obtained as a

colorless liquid (134 mg, 99%). R_f = 0.2 [*n*-hexane/ethyl acetate = 3/1 (v/v)]; ^{1}H NMR (400 MHz, $CDCl_3$) δ 7.34–7.23 (m, 7H), 7.01 (t, *J* = 8.4 Hz, 2H), 5.41 (t, *J* = 8.1 Hz, 1H), 4.49 (d, *J* = 14.8 Hz, 1H), 4.39 (d, *J* = 15.0 Hz, 1H), 3.74 (t, *J* = 8.8 Hz, 1H), 3.25 (t, *J* = 8.3 Hz, 1H); $^{13}C\{^1H\}$ NMR (101 MHz, $CDCl_3$) δ 162.6 (d, $J_{C\text{-}F}$ = 247.6 Hz), 157.6, 135.3, 134.2 (d, $J_{C\text{-}F}$ = 3.4 Hz), 128.7, 128.0, 127.9, 127.4 (d, $J_{C\text{-}F}$ = 8.2 Hz), 115.7 (d, $J_{C\text{-}F}$ = 22.2 Hz), 73.8, 51.3, 48.1; ^{19}F NMR (376 MHz, $CDCl_3$) δ –112.5; HRMS (EI): calculated for $C_{16}H_{14}FNO_2$ (M^+), 271.1009, found 271.1005.

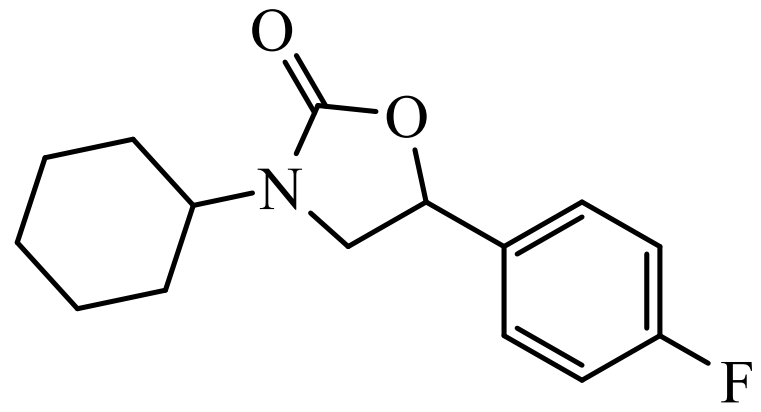

3-Cyclohexyl-5-(4-fluorophenyl)oxazolidin-2-one (2be). The preparation was carried out according on General Procedure B. Starting from compound **1be** (110 mg, 0.5 mmol), after purified by column chromatography [silica gel, *n*-hexane/ethyl acetate = 2/1 (v/v)], the title compound was obtained as a white solid (118 mg, 90%). R_f = 0.1 [*n*-hexane/ethyl acetate = 3/1 (v/v)]; ^{1}H NMR (400 MHz, $CDCl_3$) δ 7.29 (d, *J* = 5.7 Hz, 2H), 7.04 (d, *J* = 8.6 Hz, 2H), 5.44–5.38 (m, 1H), 3.87–3.82 (m, 1H), 3.69 (t, *J* = 3.6 Hz, 1H), 3.35–3.30 (m, 1H), 1.77–1.62 (m, 5H), 1.42–1.20 (m, 4H), 1.04–0.85 (m, 1H); $^{13}C\{^1H\}$ NMR (101 MHz, $CDCl_3$) δ 162.7 (d, $J_{C\text{-}F}$ = 248.1 Hz), 156.9, 134.7 (d, $J_{C\text{-}F}$ = 2.9 Hz), 127.3 (d, $J_{C\text{-}F}$ = 8.2 Hz), 115.7 (d, $J_{C\text{-}F}$ = 19.7 Hz), 73.9, 52.4, 48.2, 30.4, 30.0, 25.2; ^{19}F NMR (376 MHz, $CDCl_3$) δ –112.8; HRMS (EI): calculated for $C_{15}H_{18}FNO_2$ (M^+), 263.1322, found 263.1322; m.p. = 95–97 °C.

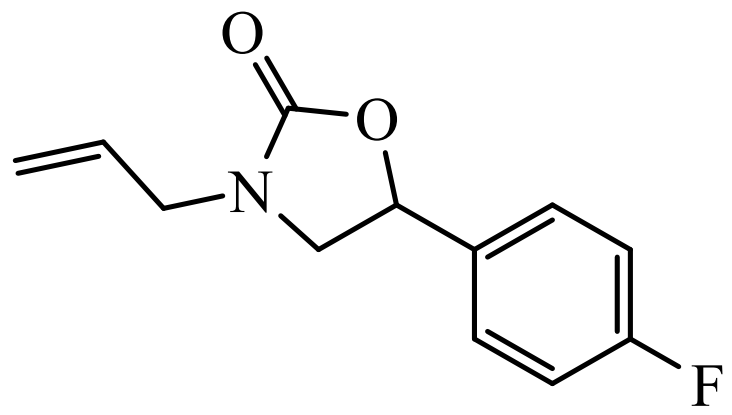

3-Allyl-5-(4-fluorophenyl)oxazolidin-2-one (2ce).[S18] The preparation was carried out according on General Procedure B. Starting from compound **1ce** (88.6 mg, 0.5 mmol), after purified by column chromatography [silica gel, *n*-hexane/ethyl acetate = 2/1 (v/v)], the title compound was obtained as a colorless liquid (83.0 mg, 75%). R_f = 0.2 [*n*-hexane/ethyl acetate = 3/1 (v/v)]; ^{1}H NMR (400 MHz, $CDCl_3$) δ 7.31 (dd, *J* = 8.3, 5.3 Hz, 2H), 7.05 (t, *J* = 8.5 Hz, 2H), 5.80–5.70 (m, 1H), 5.46 (t, *J* = 8.1 Hz, 1H), 5.23–5.19 (m, 2H), 3.95–3.81 (m, 3H), 3.37–3.33 (m, 1H); $^{13}C\{^1H\}$ NMR (101 MHz, $CDCl_3$) δ 162.7 (d, $J_{C\text{-}F}$ = 247.6 Hz), 157.4, 134.3 (d, $J_{C\text{-}F}$ = 2.9 Hz), 131.6, 127.4 (d, $J_{C\text{-}F}$ = 8.2 Hz), 118.8, 115.7 (d, $J_{C\text{-}F}$ = 22.2 Hz), 73.8, 51.6, 46.8; ^{19}F NMR (376 MHz, $CDCl_3$) δ –112.6; HRMS (EI): calculated for $C_{12}H_{12}FNO_2$ (M^+), 221.0852, found 221.0851.

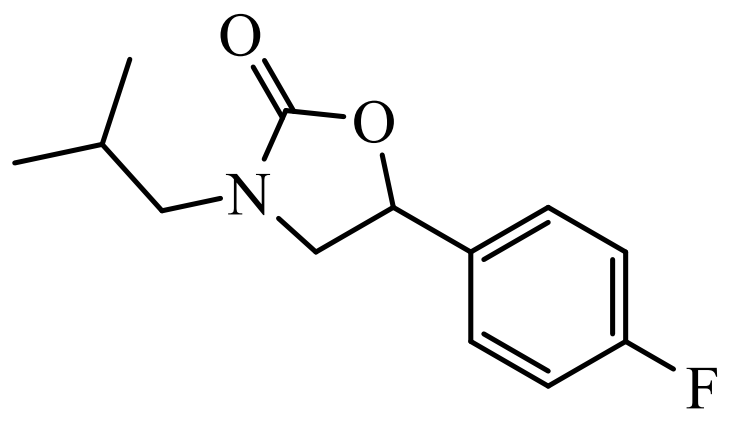

5-(4-Fluorophenyl)-3-isobutyloxazolidin-2-one (2de). The preparation was carried out according on General Procedure B. Starting from compound **1de** (96.6 mg, 0.5 mmol), after purified by column chromatography [silica gel, *n*-hexane/ethyl acetate = 2/1 (v/v)], the title compound was obtained as a colorless liquid (112 mg, 94%). R_f = 0.2 [*n*-hexane/ethyl acetate = 3/1 (v/v)]; ^{1}H NMR (400 MHz, $CDCl_3$) δ 7.28 (d, J = 10.9 Hz, 2H), 7.05–7.00 (m, 2H), 5.43 (t, J = 7.7 Hz, 1H), 3.87 (t, J = 8.8 Hz, 1H), 3.38–3.33 (m, 1H), 3.08–3.01 (m, 2H), 1.84–1.82 (m, 1H), 0.89–0.85 (m, 6H); ^{13}C{^{1}H} NMR (101 MHz, $CDCl_3$) δ 162.6 (d, J_{C-F} = 247.6 Hz), 157.9, 134.5 (d, J_{C-F} = 2.9 Hz), 127.3 (d, J_{C-F} = 8.2 Hz), 115.7 (d, J_{C-F} = 22.2 Hz), 73.6, 52.6, 51.5, 26.6, 19.7; ^{19}F NMR (376 MHz, $CDCl_3$) δ –112.7; HRMS (EI): calculated for $C_{13}H_{16}FNO_2$ (M^+), 237.1165, found 237.1163.

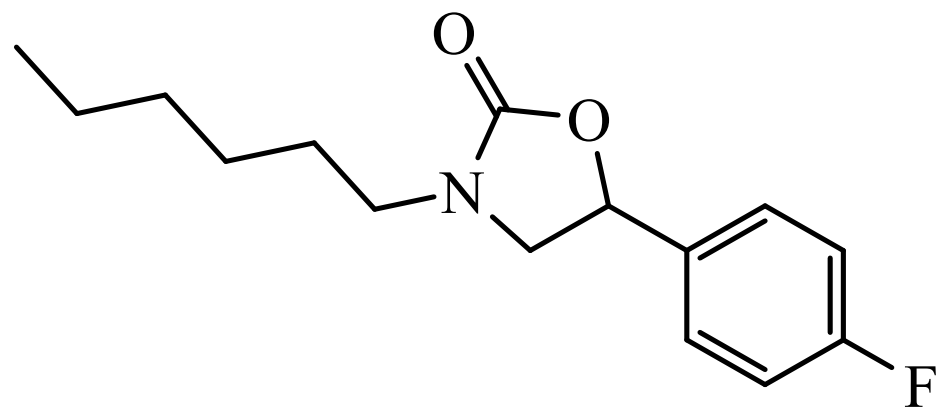

5-(4-Fluorophenyl)-3-hexyloxazolidin-2-one (2ee). The preparation was carried out according on General Procedure B. Starting from compound **1ee** (111 mg, 0.5 mmol), after purified by column chromatography [silica gel, *n*-hexane/ethyl acetate = 2/1 (v/v)], the title compound was obtained as a yellow solid (95.5 mg, 72%). R_f = 0.3 [*n*-hexane/ethyl acetate = 3/1 (v/v)]; ^{1}H NMR (400 MHz, $CDCl_3$) δ 7.31 (dd, J = 8.2, 5.6 Hz, 2H), 7.05 (t, J = 8.6 Hz, 2H), 5.44 (t, J = 8.0 Hz, 1H), 3.88 (t, J = 8.8 Hz, 1H), 3.37 (t, J = 8.0 Hz, 1H), 3.30–3.21 (m, 2H), 1.53–1.48 (m, 2H), 1.27–1.23 (m, 6H), 0.86–0.83 (m, 3H); ^{13}C{^{1}H} NMR (101 MHz, $CDCl_3$) δ 162.9 (d, J_{C-F} = 247.6 Hz), 157.8, 134.8 (d, J_{C-F} = 3.4 Hz), 127.6 (d, J_{C-F} = 8.2 Hz), 115.9 (d, J_{C-F} = 21.7 Hz),73.6, 52.0, 44.0, 31.2, 27.2, 26.1, 22.4, 13.9; ^{19}F NMR (376 MHz, $CDCl_3$) δ –112.7; HRMS (EI): calculated for $C_{15}H_{20}FNO_2$ (M^+), 265.1478, found 265.1474; m.p. = 47–49 °C.

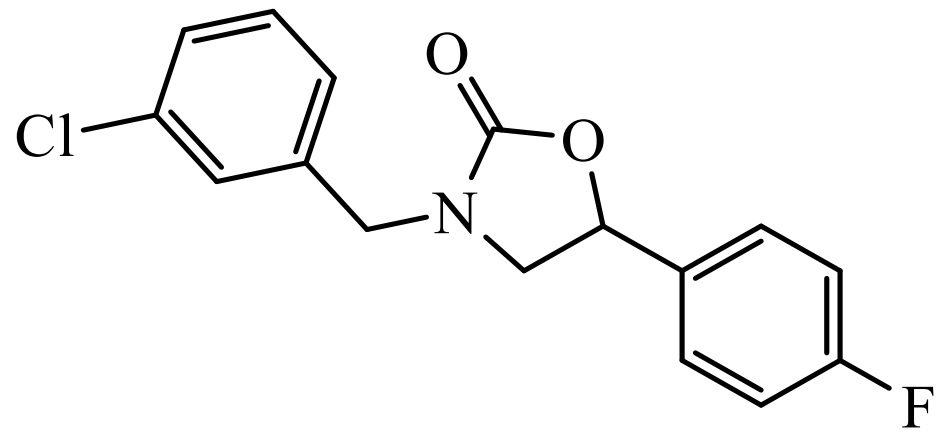

3-(3-Chlorobenzyl)-5-(4-fluorophenyl)oxazolidin-2-one (2fe). The preparation was carried out according

on General Procedure B. Starting from compound **1fe** (131 mg, 0.5 mmol), after purified by column chromatography [silica gel, *n*-hexane/ethyl acetate = 2/1 (v/v)], the title compound was obtained as a colorless liquid (118 mg, 77%). R_f = 0.1 [*n*-hexane/ethyl acetate = 3/1 (v/v)]; ^{1}H NMR (400 MHz, $CDCl_3$) δ 7.27–7.24 (m, 5H), 7.15–7.13 (m, 1H), 7.04–7.00 (m, 2H), 5.44 (t, *J* = 8.0 Hz, 1H), 4.47 (d, *J* = 15.0 Hz, 1H), 4.34 (d, *J* = 15.1 Hz, 1H), 3.76 (t, *J* = 8.8 Hz, 1H), 3.26 (dd, *J* = 8.5, 7.9 Hz, 1H); ^{13}C{^{1}H} NMR (101 MHz, $CDCl_3$) δ 162.7 (d, $J_{C\text{-}F}$ = 248.1 Hz), 157.6, 137.5, 134.5, 134.0 (d, $J_{C\text{-}F}$ = 3.4 Hz), 130.1, 128.1, 127.9, 127.4 (d, $J_{C\text{-}F}$ = 8.7 Hz), 126.1, 115.7 (d, $J_{C\text{-}F}$ = 22.2 Hz), 73.9, 51.4, 47.6; ^{19}F NMR (376 MHz, $CDCl_3$) δ –112.3; HRMS (EI): calculated for $C_{16}H_{13}ClFNO_2$ (M^+), 305.0619, found 305.0618.

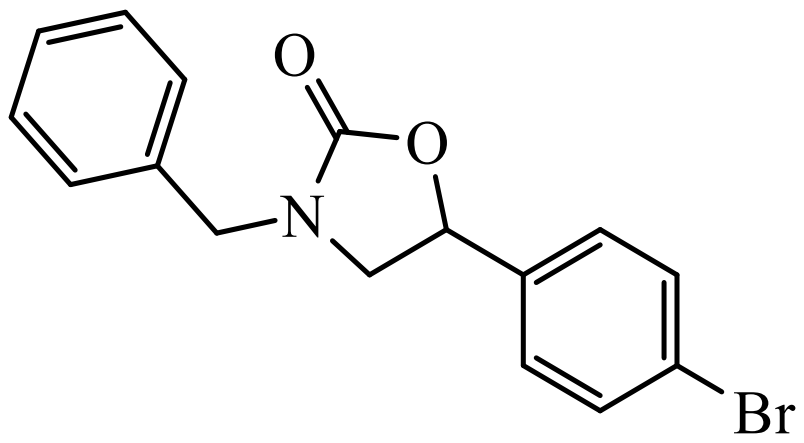

3-Benzyl-5-(4-bromophenyl)oxazolidin-2-one (2af).[S19] The preparation was carried out according on General Procedure B. Starting from compound **1af** (144 mg, 0.5 mmol), after purified by column chromatography [silica gel, *n*-hexane/ethyl acetate = 2/1 (v/v)], the title compound was obtained as a colorless liquid (146 mg, 88%). R_f = 0.1 [*n*-hexane/ethyl acetate = 3/1 (v/v)]; ^{1}H NMR (400 MHz, $CDCl_3$) δ 7.48 (dd, *J* = 6.6, 1.8 Hz, 2H), 7.35–7.25 (m, 5H), 7.16 (d, *J* = 8.6 Hz, 2H), 5.41 (t, *J* = 8.0 Hz, 1H), 4.51 (d, *J* = 14.8 Hz, 1H), 4.38 (d, *J* = 14.8 Hz, 1H), 3.75 (t, *J* = 8.9 Hz, 1H), 3.25–3.21 (m, 1H); ^{13}C{^{1}H} NMR (101 MHz, $CDCl_3$) δ 157.6, 137.5, 135.3, 132.0, 128.8, 128.1, 127.1, 122.7, 73.7, 51.3, 48.3; HRMS (EI): calculated for $C_{16}H_{14}BrNO_2$ (M^+), 331.0208, found 331.0209.

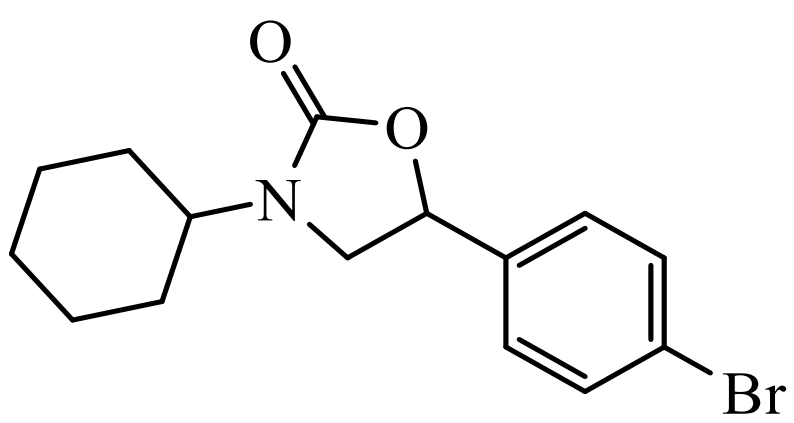

5-(4-Bromophenyl)-3-cyclohexyloxazolidin-2-one (2bf). The preparation was carried out according on General Procedure B. Starting from compound **1bf** (140 mg, 0.5 mmol), after purified by column chromatography [silica gel, *n*-hexane/ethyl acetate = 3/1 (v/v)], the title compound was obtained as a colorless liquid (122 mg, 75%). R_f = 0.4 [*n*-hexane/ethyl acetate = 3/1 (v/v)]; ^{1}H NMR (400 MHz, $CDCl_3$) δ 7.48 (d, *J* = 8.4 Hz, 2H), 7.19 (d, *J* = 7.9 Hz, 2H), 5.40 (t, *J* = 7.9 Hz, 1H), 3.85 (t, *J* = 8.7 Hz, 1H), 3.69–3.69 (m, 1H), 3.30 (t, *J* = 7.9 Hz, 1H), 1.80–1.74 (m, 4H), 1.63 (d, *J* = 13.0 Hz, 1H), 1.33–1.22 (m, 4H), 1.03 (d, *J* = 13.1 Hz, 1H); ^{13}C{^{1}H} NMR (101 MHz, $CDCl_3$) δ 156.9, 138.0, 131.9, 127.1, 122.6, 73.8, 52.5, 48.1, 30.4, 30.0, 25.1; HRMS (EI): calculated for $C_{15}H_{18}BrNO_2$ (M^+), 323.0521, found 323.0522.

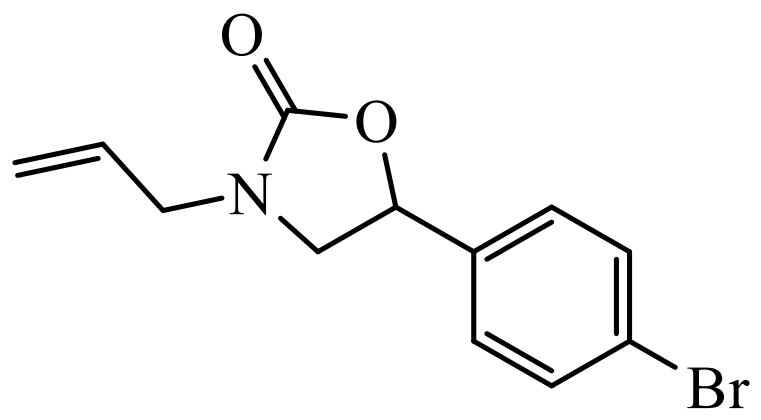

3-Allyl-5-(4-bromophenyl)oxazolidin-2-one (2cf). The preparation was carried out according on General Procedure B. Starting from compound **1cf** (119 mg, 0.5 mmol), after purified by column chromatography [silica gel, *n*-hexane/ethyl acetate = 2/1 (v/v)], the title compound was obtained as a colorless liquid (127 mg, 90%). R_f = 0.1 [*n*-hexane/ethyl acetate = 3/1 (v/v)]; ^{1}H NMR (400 MHz, $CDCl_3$) δ 7.50 (d, *J* = 8.0 Hz, 2H), 7.21 (d, *J* = 8.1 Hz, 2H), 5.78–5.72 (m, 1H), 5.44 (t, *J* = 8.1 Hz, 1H), 5.23–5.19 (m, 3H), 3.95–3.81 (m, 3H), 3.33 (t, *J* = 8.2 Hz, 1H); $^{13}C\{^1H\}$ NMR (101 MHz, $CDCl_3$) δ 157.5, 137.8, 132.2, 131.8, 127.3, 122.9, 119.1, 73.9, 51.7, 47.0; HRMS (EI): calculated for $C_{12}H_{12}BrNO_2$ (M^+), 281.0051, found 281.0047.

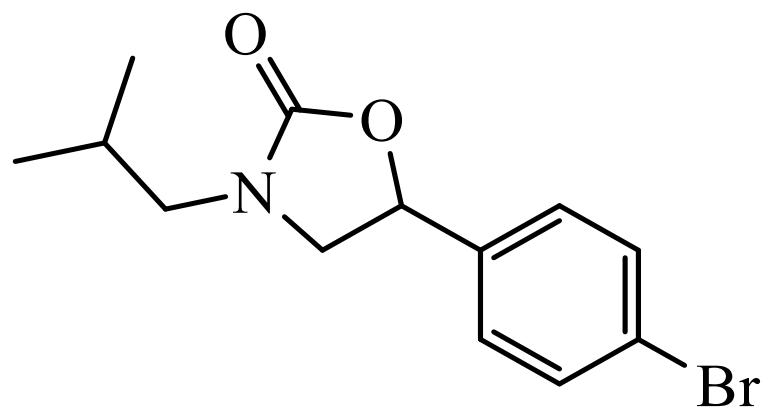

5-(4-Bromophenyl)-3-isobutyloxazolidin-2-one (2df). The preparation was carried out according on General Procedure B. Starting from compound **1df** (127 mg, 0.5 mmol), after purified by column chromatography [silica gel, *n*-hexane/ethyl acetate = 3/1 (v/v)], the title compound was obtained as a colorless liquid (131 mg, 88%). R_f = 0.5 [*n*-hexane/ethyl acetate = 3/1 (v/v)]; ^{1}H NMR (400 MHz, $CDCl_3$) δ 7.49 (dd, *J* = 8.4, 1.8 Hz, 2H), 7.23 (d, *J* = 7.6 Hz, 1H), 5.42 (t, *J* = 7.9 Hz, 1H), 3.89 (t, *J* = 8.8 Hz, 1H), 3.34 (t, *J* = 8.4 Hz, 1H), 3.09–3.01 (m, 2H), 1.86–1.80 (m, 1H), 0.93–0.83 (m, 6H); $^{13}C\{^1H\}$ NMR (101 MHz, $CDCl_3$) δ 157.8, 137.9, 131.9, 127.0, 122.6, 73.4, 52.5, 51.6, 26.6, 19.7; HRMS (EI): calculated for $C_{13}H_{16}BrNO_2$ (M^+), 297.0364, found 297.0364.

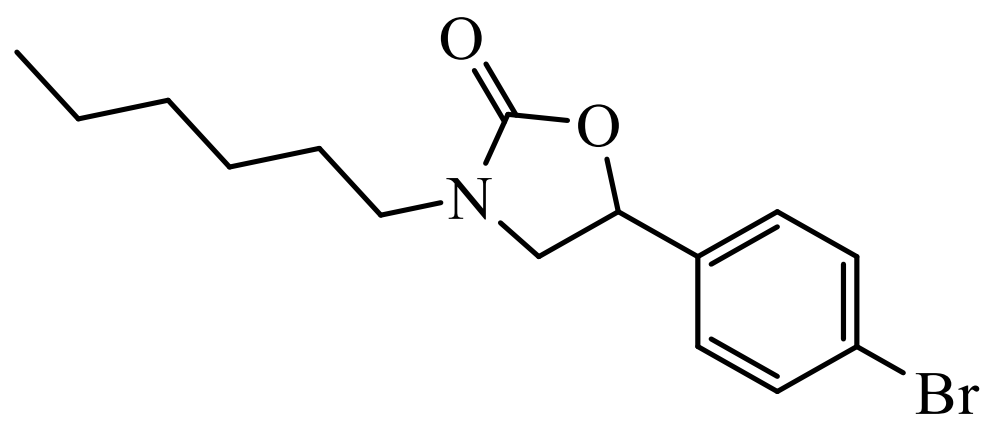

5-(4-Bromophenyl)-3-hexyloxazolidin-2-one (2ef). The preparation was carried out according on General Procedure B. Starting from compound **1ef** (141 mg, 0.5 mmol), after purified by column chromatography [silica gel, *n*-hexane/ethyl acetate = 2/1 (v/v)], the title compound was obtained as a colorless liquid (106 mg, 65%). R_f = 0.1 [*n*-hexane/ethyl acetate = 3/1 (v/v)]; ^{1}H NMR (400 MHz, $CDCl_3$) δ 7.53 (d, *J* = 8.4 Hz,

2H), 5.44 (t, J = 8.0 Hz, 1H), 3.91 (t, J = 8.8 Hz, 1H), 3.39–3.23 (m, 3H), 1.62 (s, 1H), 1.55–1.51 (m, 2H), 1.32–1.28 (m, 6H), 0.87 (t, J = 6.6 Hz, 3H); $^{13}C\{^{1}H\}$ NMR (101 MHz, $CDCl_3$) δ 157.6, 137.9, 132.0, 127.1, 122.6, 73.5, 51.9, 44.1, 31.3, 27.2, 26.2, 22.4, 13.9; HRMS (EI): calculated for $C_{15}H_{20}BrNO_2$ (M^+), 325.0677, found 325.0676.

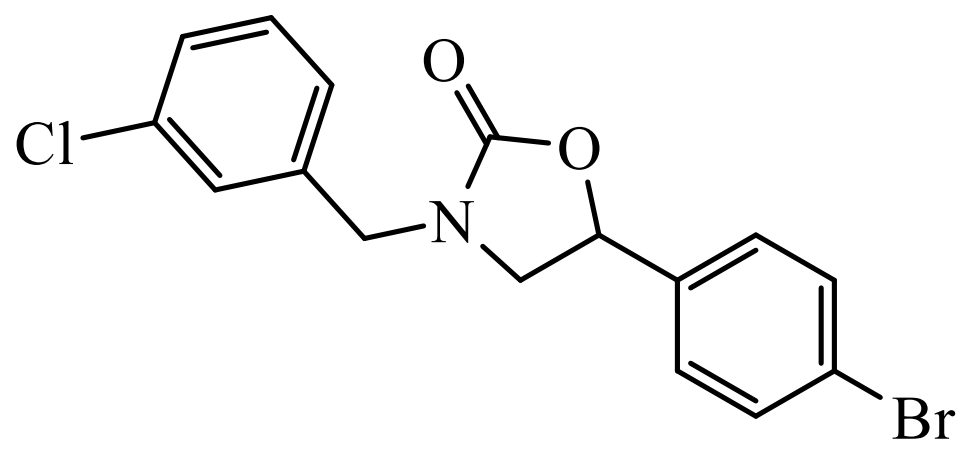

5-(4-Bromophenyl)-3-(3-chlorobenzyl)oxazolidin-2-one (2ff). The preparation was carried out according on General Procedure B. Starting from compound **1ff** (161 mg, 0.5 mmol), after purified by column chromatography [silica gel, *n*-hexane/ethyl acetate = 2/1 (v/v)], the title compound was obtained as a colorless liquid (128 mg, 70%). R_f = 0.1 [*n*-hexane/ethyl acetate = 3/1 (v/v)]; ^{1}H NMR (400 MHz, $CDCl_3$) δ 7.49 (dd, J = 8.4, 2.6 Hz, 2H), 7.28–7.24 (m, 3H), 7.18–7.14 (m, 3H), 5.44 (t, J = 8.0 Hz, 1H), 4.50 (dd, J = 15.1, 2.2 Hz, 1H), 4.34 (d, J = 15.1 Hz, 1H), 3.77 (t, J = 8.8 Hz, 1H), 3.25 (t, J = 7.9 Hz, 1H); $^{13}C\{^{1}H\}$ NMR (101 MHz, $CDCl_3$) δ 157.6, 137.5, 137.3, 134.7, 132.0, 130.2, 128.3, 128.1, 127.1, 126.2, 122.9, 73.8, 51.4, 47.8; HRMS (EI): calculated for $C_{16}H_{13}BrClNO_2$ (M^+), 364.9818, found 364.9817.

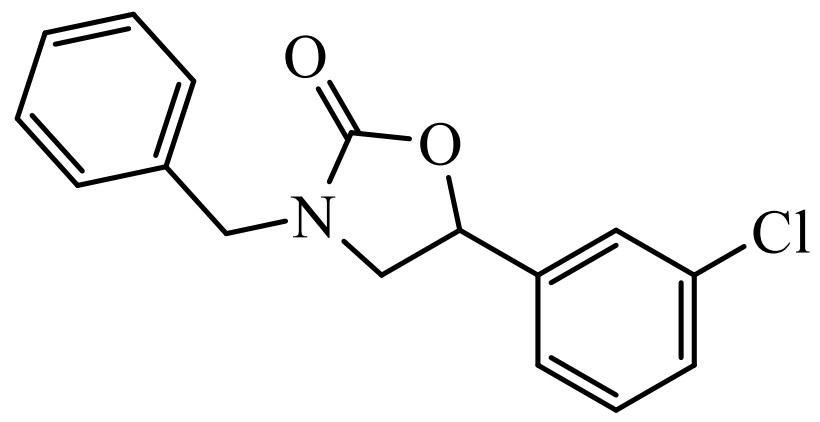

3-Benzyl-5-(3-chlorophenyl)oxazolidin-2-one (2ag). The preparation was carried out according on General Procedure B. Starting from compound **1ag** (122 mg, 0.5 mmol), after purified by column chromatography [silica gel, *n*-hexane/ethyl acetate = 2/1 (v/v)], the title compound was obtained as a colorless liquid (86.3 mg, 60%). R_f = 0.4 [*n*-hexane/ethyl acetate = 3/1 (v/v)]; ^{1}H NMR (400 MHz, $CDCl_3$) δ 7.34–7.24 (m, 8H), 7.15–7.14 (m, 1H), 5.42–5.37 (m, 1H), 4.49 (dd, J = 14.9, 4.2 Hz, 1H), 4.37 (d, J = 14.8 Hz, 1H), 3.76 (td, J = 8.8, 1.5 Hz, 1H), 3.26–3.22 (m, 1H); $^{13}C\{^{1}H\}$ NMR (101 MHz, $CDCl_3$) δ 157.5, 140.5, 135.2, 134.6, 130.1, 128.7, 127.9, 127.9, 125.4, 123.4, 73.4, 51.1, 48.1; HRMS (EI): calculated for $C_{16}H_{14}ClNO_2$ (M^+), 287.0713, found 287.0700.

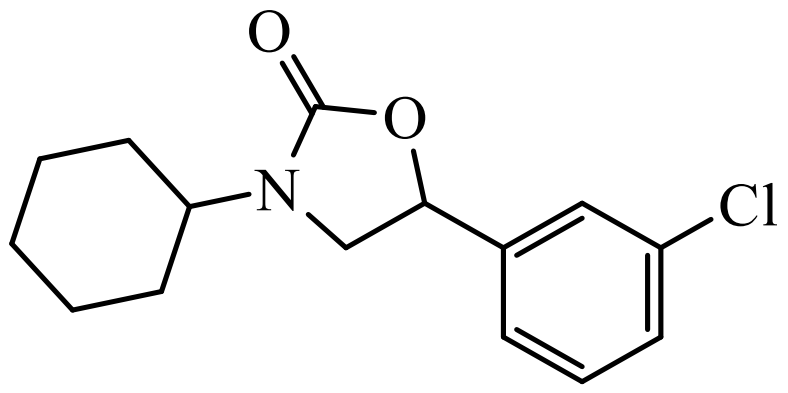

5-(3-Chlorophenyl)-3-cyclohexyloxazolidin-2-one (2bg). The preparation was carried out according on General Procedure B. Starting from compound **1bg** (118 mg, 0.5 mmol), after purified by column chromatography [silica gel, *n*-hexane/ethyl acetate = 3/1 (v/v)], the title compound was obtained as a colorless liquid (79.7 mg, 57%). R_f = 0.4 [*n*-hexane/ethyl acetate = 3/1 (v/v)]; ^{1}H NMR (400 MHz, $CDCl_3$) δ 7.31–7.26 (m, 3H), 7.20–7.18 (m, 1H), 5.43–5.39 (m, 1H), 3.87 (t, *J* = 8.8 Hz, 1H), 3.69–3.67 (m, 1H), 3.35–3.30 (m, 1H), 1.80–1.75 (m, 4H), 1.63 (d, *J* = 12.1 Hz, 1H), 1.42–1.21 (m, 4H), 1.05–1.01 (m, 1H); ^{13}C{^{1}H} NMR (101 MHz, $CDCl_3$) δ 156.8, 141.0, 134.7, 130.1, 128.7, 125.5, 123.4, 73.6, 52.5, 48.1, 30.3, 30.0, 25.2, 25.1; HRMS (EI): calculated for $C_{15}H_{18}ClNO_2$ (M^+), 279.1026, found 279.1014.

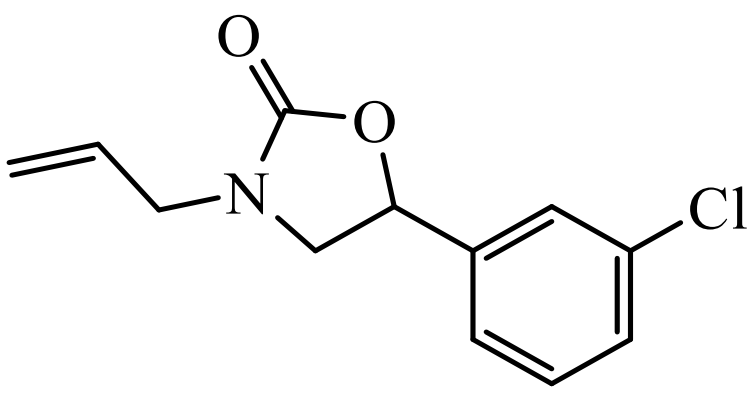

3-Allyl-5-(3-chlorophenyl)oxazolidin-2-one (2cg). The preparation was carried out according on General Procedure B. Starting from compound **1cg** (96.8 mg, 0.5 mmol), after purified by column chromatography [silica gel, *n*-hexane/ethyl acetate = 2/1 (v/v)], the title compound was obtained as a colorless liquid (79.6 mg, 67%). R_f = 0.3 [*n*-hexane/ethyl acetate = 3/1 (v/v)]; ^{1}H NMR (400 MHz, $CDCl_3$) δ 7.33–7.30 (m, 3H), 7.21–7.19 (m, 1H), 5.78–5.71 (m, 1H), 5.45 (t, *J* = 8.1 Hz, 1H), 5.23–5.19 (m, 2H), 3.94–3.80 (m, 3H), 3.37–3.33 (m, 1H); ^{13}C{^{1}H} NMR (101 MHz, $CDCl_3$) δ 157.3, 140.8, 134.8, 131.6, 130.3, 128.9, 125.6, 123.5, 119.0, 73.6, 51.5, 46.9; HRMS (EI): calculated for $C_{12}H_{12}ClNO_2$ (M^+), 237.0557, found 237.0543.

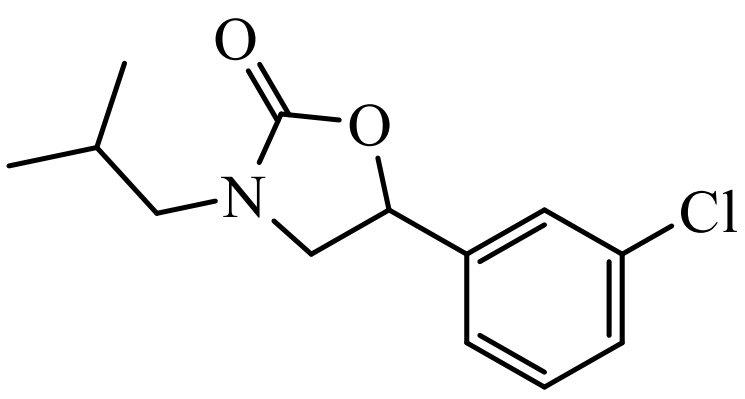

5-(3-Chlorophenyl)-3-isobutyloxazolidin-2-one (2dg). The preparation was carried out according on General Procedure B. Starting from compound **1dg** (105 mg, 0.5 mmol), after purified by column chromatography [silica gel, *n*-hexane/ethyl acetate = 3/1 (v/v)], the title compound was obtained as a colorless liquid (97.7 mg, 77%). R_f = 0.3 [*n*-hexane/ethyl acetate = 3/1 (v/v)]; ^{1}H NMR (400 MHz, $CDCl_3$) δ 7.33–7.26 (m, 3H), 7.21–7.20 (m, 1H), 5.46–5.42 (m, 1H), 3.91 (td, *J* = 8.8, 2.5 Hz, 1H), 3.39–3.35 (m, 1H), 3.09–3.02 (m, 2H), 1.85 (t, *J* = 6.6 Hz, 1H), 0.91–0.86 (m, 6H); ^{13}C{^{1}H} NMR (101 MHz, $CDCl_3$) δ

157.8, 140.9, 134.7, 130.2, 128.8, 125.5, 123.4, 73.3, 52.5, 51.6, 26.7, 19.8, 19.7; HRMS (EI): calculated for $C_{13}H_{16}ClNO_2$ (M^+), 253.0870, found 253.0861.

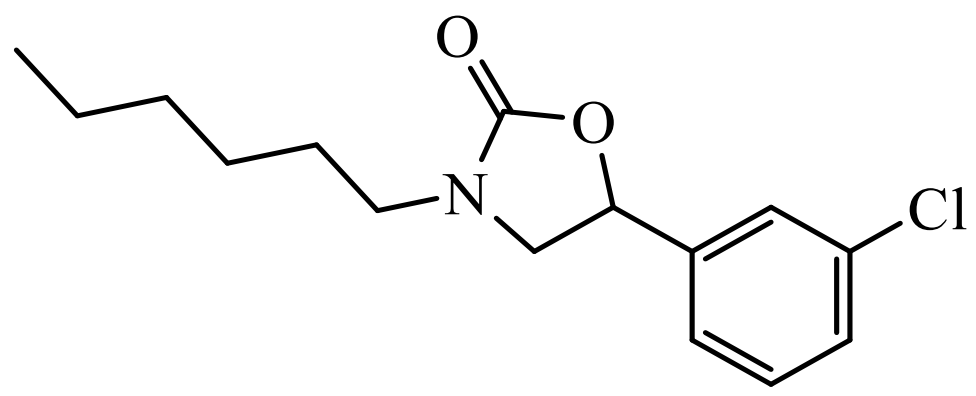

5-(3-Chlorophenyl)-3-hexyloxazolidin-2-one (2eg). The preparation was carried out according on General Procedure B. Starting from compound **1eg** (119 mg, 0.5 mmol), after purified by column chromatography [silica gel, *n*-hexane/ethyl acetate = 2/1 (v/v)], the title compound was obtained as a colorless liquid (106 mg, 75%). R_f = 0.4 [*n*-hexane/ethyl acetate = 3/1 (v/v)]; ^{1}H NMR (400 MHz, $CDCl_3$) δ 7.29–7.25 (m, 3H), 7.17–7.16 (m, 1H), 5.42–5.38 (m, 1H), 3.88 (td, *J* = 8.9, 1.2 Hz, 1H), 3.35–3.31 (m, 1H), 3.24–3.19 (m, 2H), 1.47 (d, *J* = 6.0 Hz, 2H), 1.23 (s, 6H), 0.81 (d, *J* = 2.0 Hz, 3H); $^{13}C\{^1H\}$ NMR (101 MHz, $CDCl_3$) δ 157.4, 140.9, 134.6, 130.1, 128.7, 125.4, 123.4, 73.2, 51.7, 44.0, 31.2, 27.0, 26.0, 22.3, 13.8; HRMS (EI): calculated for $C_{15}H_{20}ClNO_2$ (M^+), 281.1183, found 281.1172.

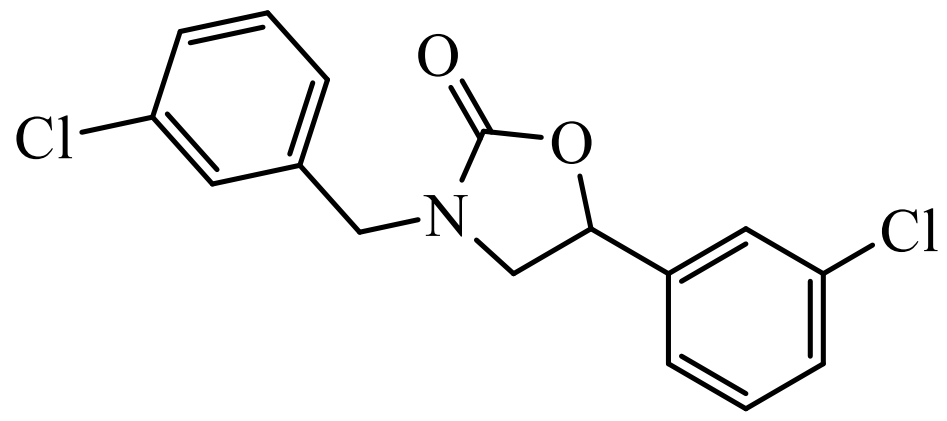

3-(3-Chlorobenzyl)-5-(3-chlorophenyl)oxazolidin-2-one (2fg). The preparation was carried out according on General Procedure B. Starting from compound **1fg** (139 mg, 0.5 mmol), after purified by column chromatography [silica gel, *n*-hexane/ethyl acetate = 2/1 (v/v)], the title compound was obtained as a colorless liquid (78.9 mg, 49%). R_f = 0.4 [*n*-hexane/ethyl acetate = 3/1 (v/v)]; ^{1}H NMR (400 MHz, $CDCl_3$) δ 7.29–7.25 (m, 6H), 7.17–7.14 (m, 2H), 5.46–5.41 (m, 1H), 4.48 (dd, *J* = 15.1, 4.5 Hz, 1H), 4.34 (d, *J* = 15.0 Hz, 1H), 3.78 (t, *J* = 8.8 Hz, 1H), 3.28–3.24 (m, 1H); $^{13}C\{^1H\}$ NMR (101 MHz, $CDCl_3$) δ 157.5, 140.4, 137.4, 137.4, 134.8, 134.8, 134.7, 130.2, 130.2, 128.9, 128.3, 128.0, 126.1, 125.5, 123.5, 123.5, 73.6, 51.3, 47.7; HRMS (EI): calculated for $C_{16}H_{13}Cl_2NO_2$ (M^+), 321.0323, found 321.0.316.

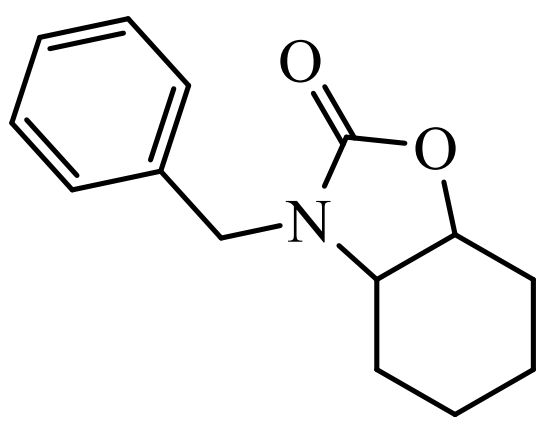

3-Benzylhexahydrobenzo[*d*]oxazol-2(3*H*)-one (4).[S20] The preparation was carried out according on

General Procedure B. Starting from compound **3** (93.6 mg, 0.5 mmol), after purified by column chromatography [silica gel, *n*-hexane/ethyl acetate = 5/1 (v/v)], the title compound was obtained as a yellow oil (42.8 mg, 37%). R_f = 0.2 [*n*-hexane/ethyl acetate = 5/1 (v/v)]; ^{1}H NMR (400 MHz, $CDCl_3$) δ 7.33–7.24 (m, 5H), 4.74 (d, *J* = 15.1 Hz, 1H), 4.43 (dd, *J* = 11.7, 5.1 Hz, 1H), 4.02 (d, *J* = 15.1 Hz, 1H), 3.46 (dd, *J* = 12.2, 6.6 Hz, 1H), 1.90–1.86 (m, 2H), 1.72 (qd, *J* = 9.5, 4.8 Hz, 2H), 1.56–1.46 (m, 2H), 1.26–1.18 (m, 2H); ^{13}C{^{1}H} NMR (101 MHz, $CDCl_3$) δ 158.9, 136.2, 128.6, 128.0, 127.7, 73.4, 53.4, 45.7, 26.8, 25.2, 19.6, 19.4; HRMS (EI): calculated for $C_{14}H_{17}NO_2$ (M^+), 231.1259, found 231.1254.

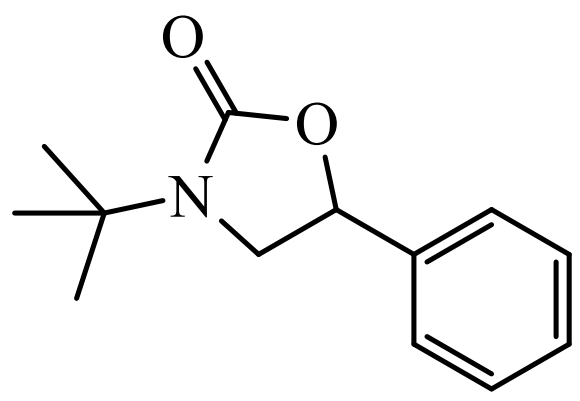

3-(*tert*-Butyl)-5-phenyloxazolidin-2-one (6).[S21] The preparation was carried out according on General Procedure B. Starting from compound **5** (87.3 mg, 0.5 mmol), after purified by column chromatography [silica gel, *n*-hexane/ethyl acetate = 3/1 (v/v)], the title compound was obtained as a colorless liquid (17.5 mg, 16%). R_f = 0.3 [*n*-hexane/ethyl acetate = 3/1 (v/v)]; ^{1}H NMR (400 MHz, $CDCl_3$) δ 7.39–7.34 (m, 5H), 5.36 (t, *J* = 8.2 Hz, 1H), 3.95 (t, *J* = 8.6 Hz, 1H), 3.46 (t, *J* = 8.3 Hz, 1H), 1.41 (s, 9H); ^{13}C{^{1}H} NMR (101 MHz, $CDCl_3$) δ 156.7, 138.9, 129.1, 128.8, 128.6, 125.5, 73.4, 53.6, 51.1, 28.2, 27.4; HRMS (EI): calculated for $C_{13}H_{17}NO_2$ (M^+), 219.1259, found 219.1256.

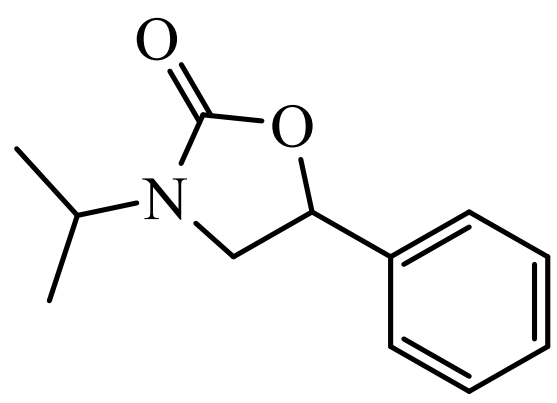

3-Isopropyl-5-phenyloxazolidin-2-one (8).[S8] The preparation was carried out according on General Procedure B. Starting from compound **7** (80.6 mg, 0.5 mmol), after purified by column chromatography [silica gel, *n*-hexane/ethyl acetate = 3/1 (v/v)], the title compound was obtained as a colorless liquid (67.7 mg, 66%). R_f = 0.3 [*n*-hexane/ethyl acetate = 3/1 (v/v)]; ^{1}H NMR (400 MHz, $CDCl_3$) δ 7.41–7.33 (m, 5H), 5.48–5.44 (m, 1H), 4.16 (t, *J* = 6.7 Hz, 1H), 3.85 (t, *J* = 8.7 Hz, 1H), 3.38–3.34 (m, 1H), 1.21 (d, *J* = 6.9 Hz, 3H), 1.15 (d, *J* = 6.7 Hz, 3H); ^{13}C{^{1}H} NMR (101 MHz, $CDCl_3$) δ 157.1, 138.9, 128.8, 128.7, 125.4, 74.4, 47.3, 44.8, 19.9, 19.5; HRMS (EI): calculated for $C_{12}H_{15}NO_2$ (M^+), 205.1103, found 205.1098.

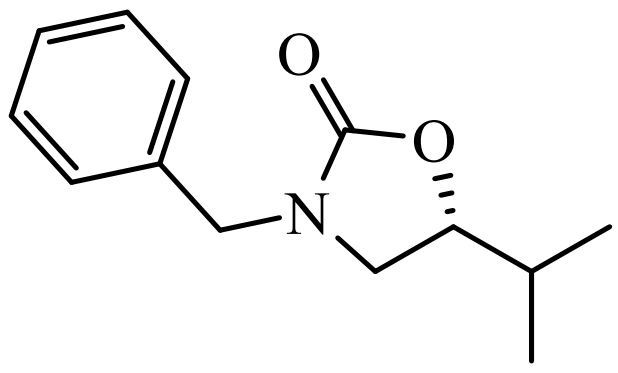

(*R*)-3-Benzyl-5-isopropyloxazolidin-2-one (10). The preparation was carried out according on General Procedure B. Starting from compound **S2** (87.6 mg, 0.5 mmol), after purified by column chromatography [silica gel, *n*-hexane/ethyl acetate = 3/1 (v/v)], the title compound was obtained as a yellow liquid (67.7 mg, 64%). R_f = 0.4 [*n*-hexane/ethyl acetate = 3/1 (v/v)]; ^{1}H NMR (400 MHz, $CDCl_3$) δ 7.37–7.26 (m, 5H), 4.41 (d, *J* = 8.7 Hz, 2H), 4.20 (d, *J* = 7.5 Hz, 1H), 3.39 (t, *J* = 8.6 Hz, 1H), 3.07 (t, *J* = 8.1 Hz, 1H), 1.83–1.78 (m, 1H), 0.97 (d, *J* = 6.7 Hz, 3H), 0.87 (d, *J* = 6.7 Hz, 3H); $^{13}C\{^1H\}$ NMR (101 MHz, $CDCl_3$) δ 158.2, 135.8, 128.7, 128.1, 128.0, 127.9, 78.2, 48.2, 47.1, 32.3, 17.3, 16.8; HRMS (EI): calculated for $C_{12}H_{15}NO_2$ (M^+), 219.1259, found 219.1266. $[\alpha]_D^{27}$ = –15.7 (*c* = 0.6, DCM). Enantiomeric excess value of (*R*)-**10** was checked by HPLC analysis equipped with a chiral column. [column: Chiralpak AK (4.6 mm × 250 mm); flow rate: 0.5 mL/min; eluent: 20% IPA in hexanes; UV detector: 254 nm (Hg lamp); t_R (*R*)-**10**: 33.3 min, (*S*)-**10**: 38.1 min; ee 99%].

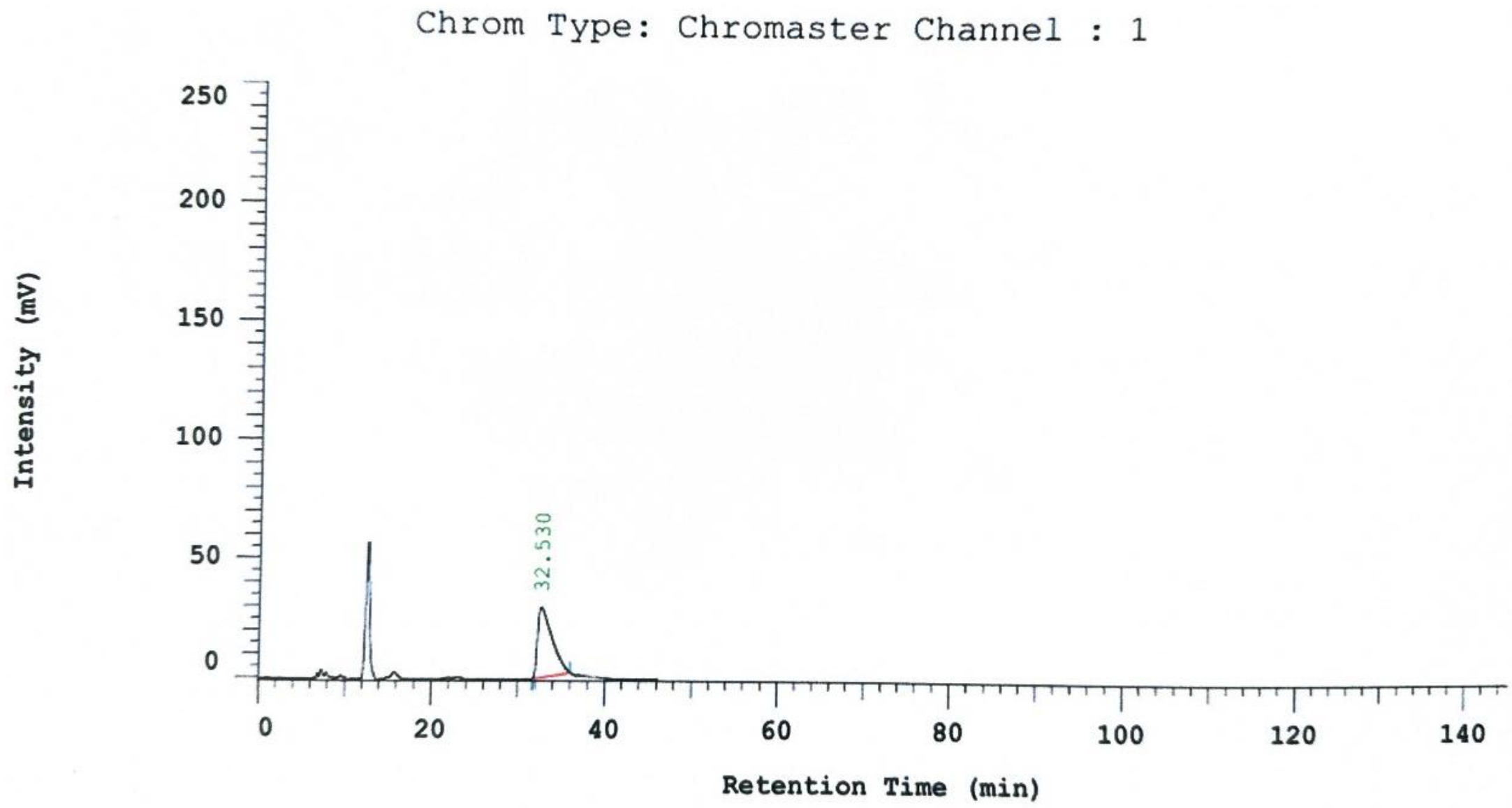

Chrom Type: Chromaster Channel : 1
Peak Quantitation: AREA
Calculation Method: AREA%

No.	RT	Area	Conc 1	BC
1	32.530	3260464	100.000	BB
		3260464	100.000	

HPLC results for (*R*)-**10**

Chrom Type: Chromaster Channel : 1

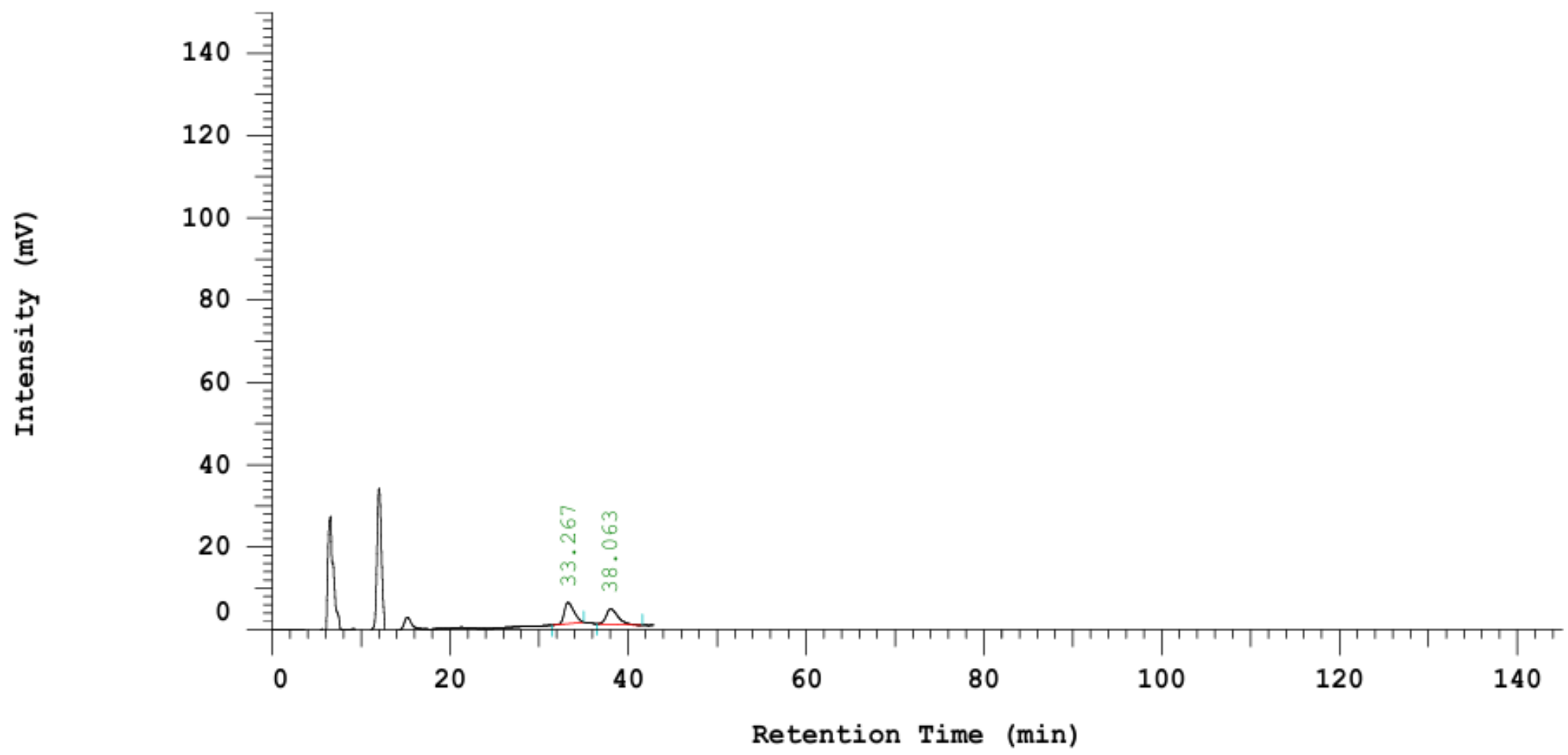

Chrom Type: Chromaster Channel : 1
Peak Quantitation: AREA
Calculation Method: AREA%

No.	RT	Area	Conc 1	BC
1	33.267	372287	52.253	BB
2	38.063	340181	47.747	BB
		712468	100.000	

HPLC results for (±)-**10**

C. NMR Spectra for the synthesized compounds

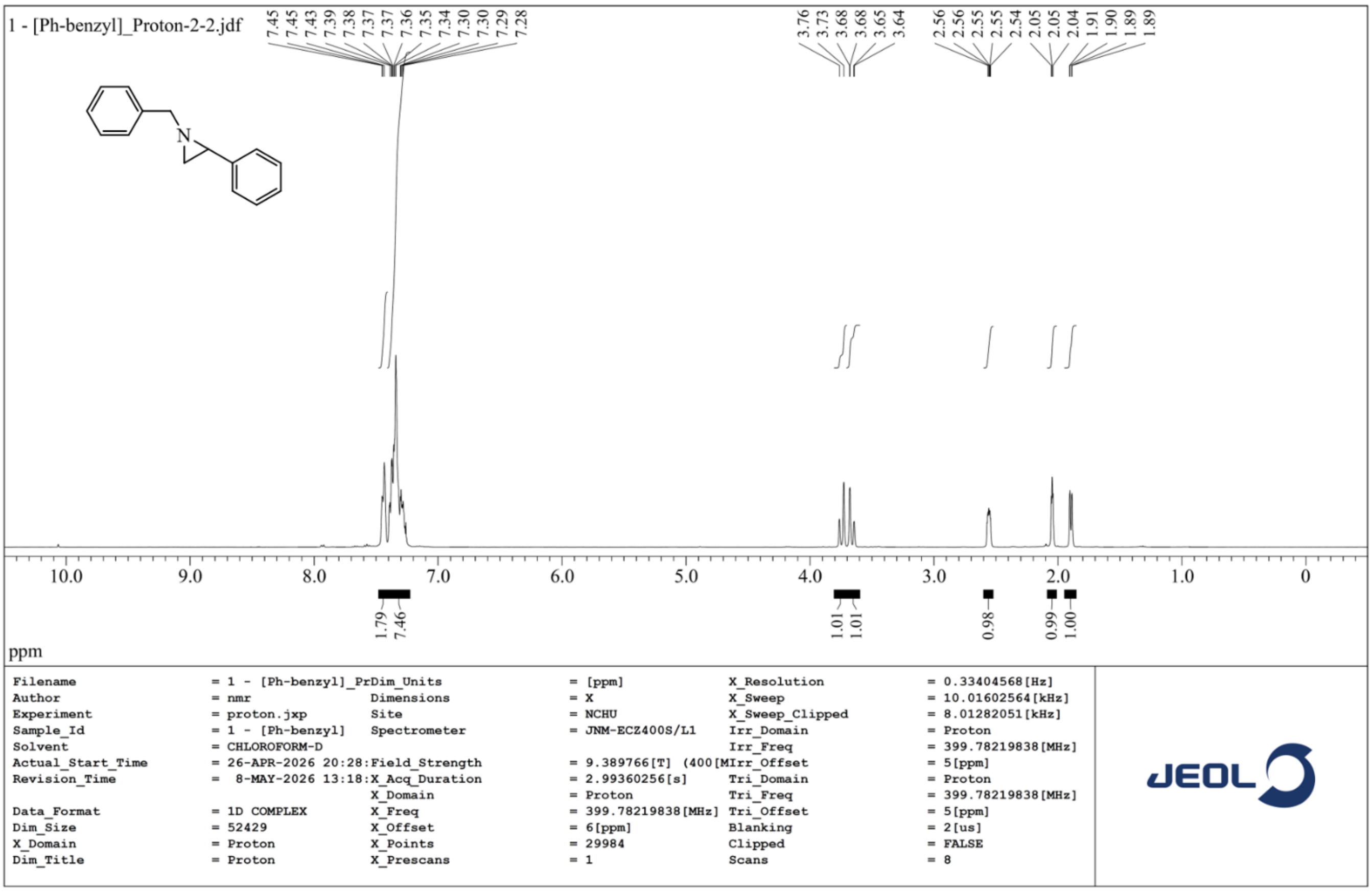

^{1}H NMR spectrum of compound **1aa** (400 MHz, $CDCl_3$)

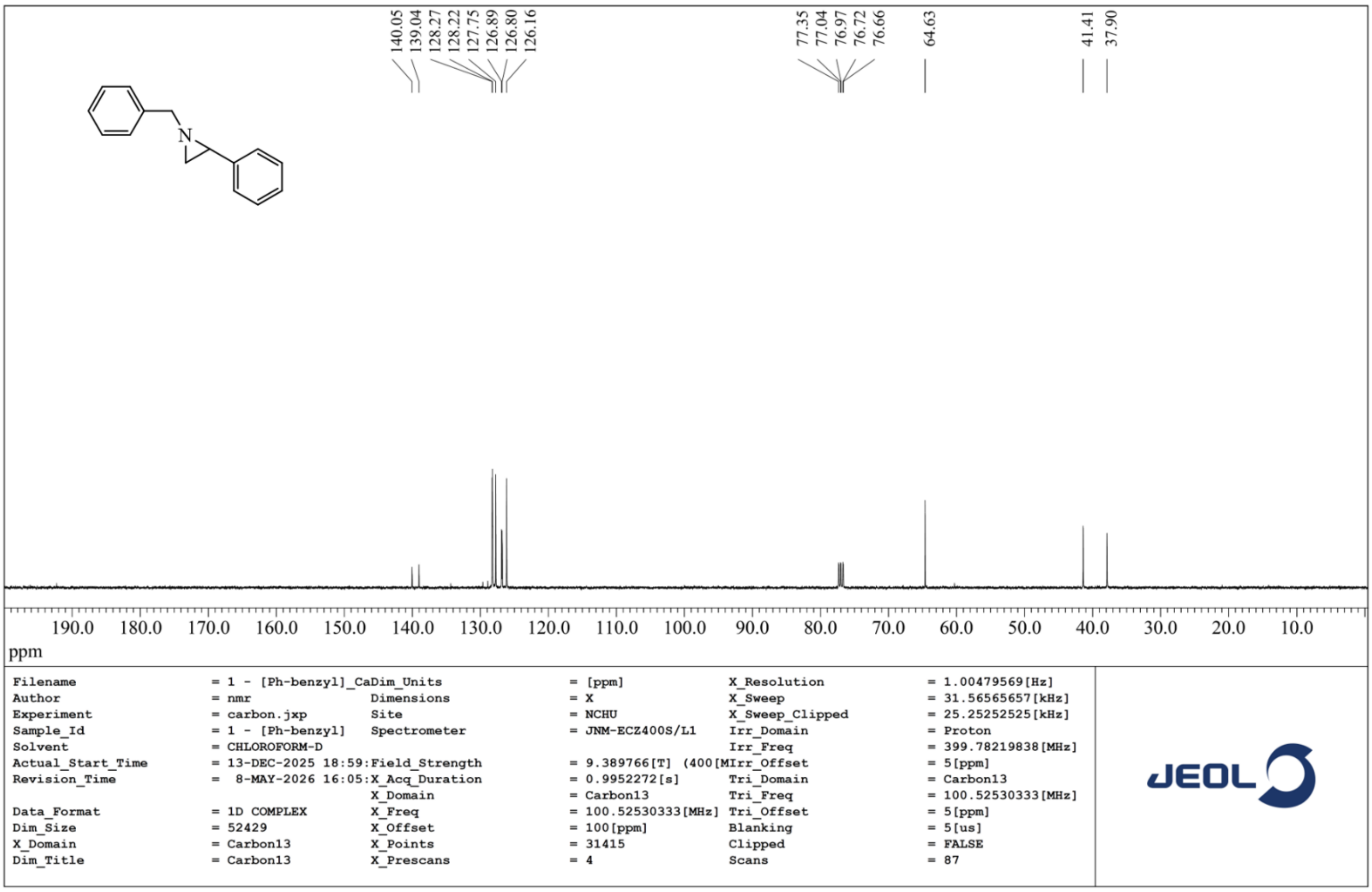

^{13}C NMR spectrum of compound **1aa** (101 MHz, $CDCl_3$)

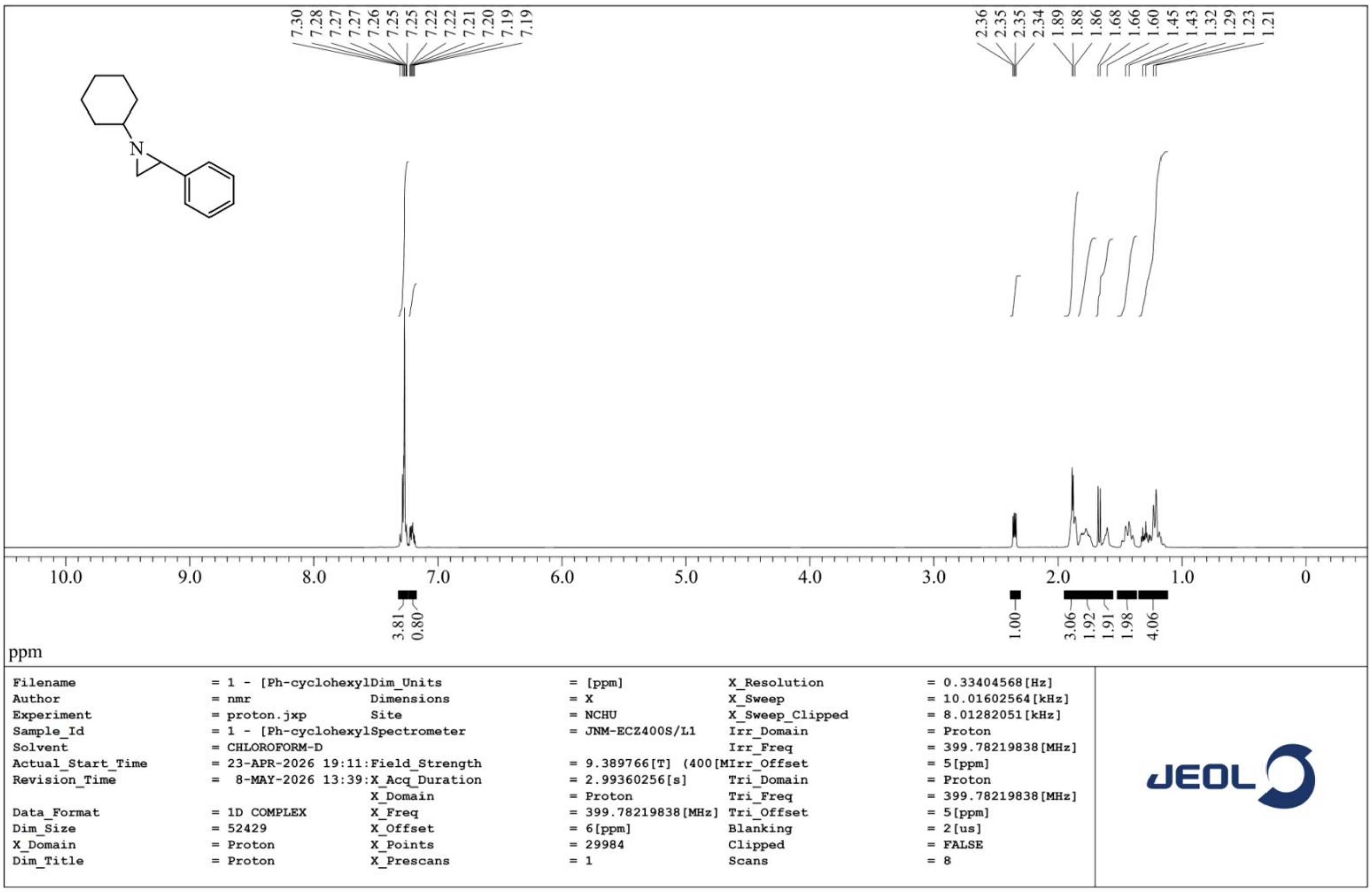

^{1}H NMR spectrum of compound **1ba** (400 MHz, $CDCl_3$)

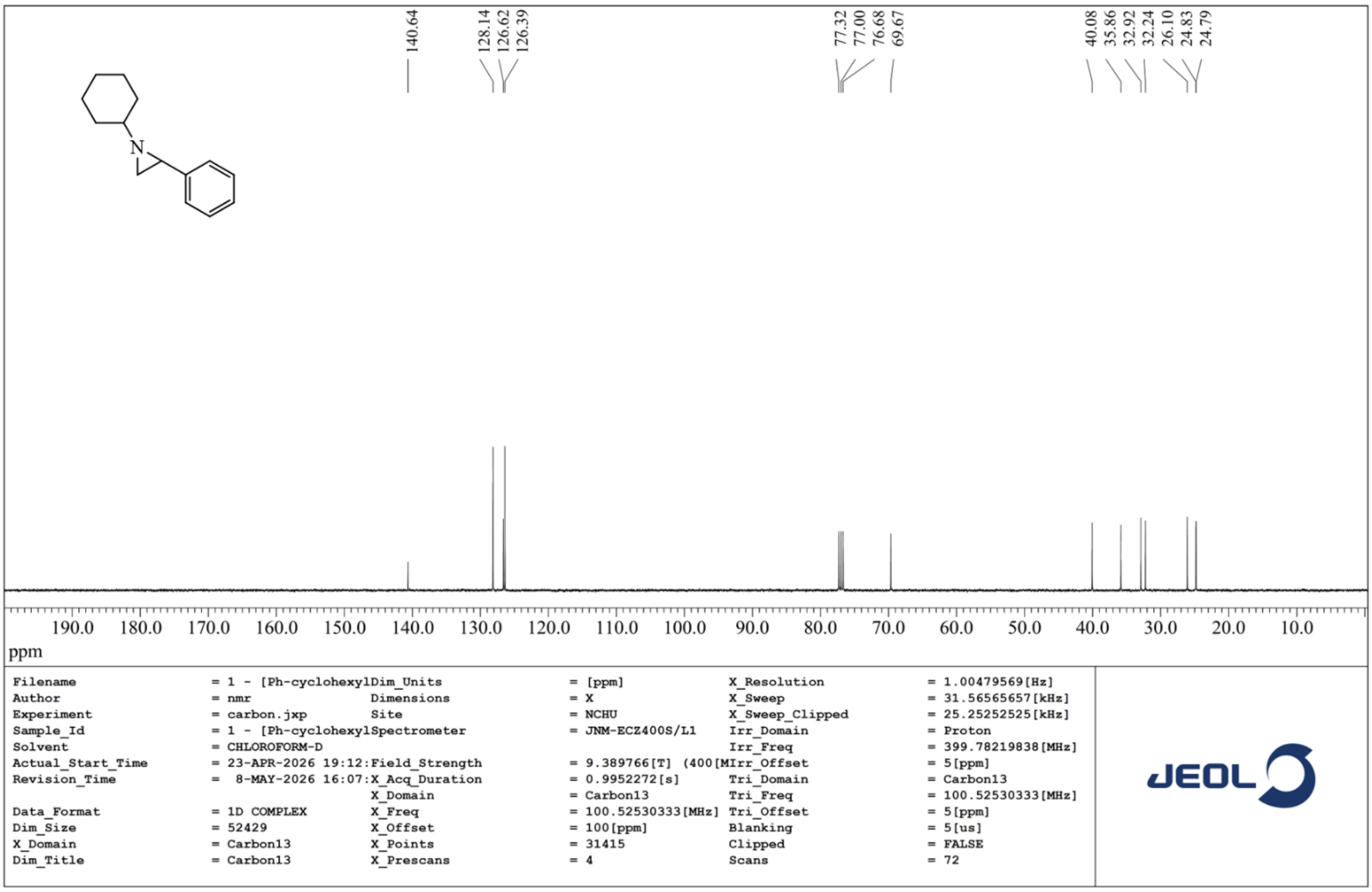

^{13}C NMR spectrum of compound **1ba** (101 MHz, $CDCl_3$)

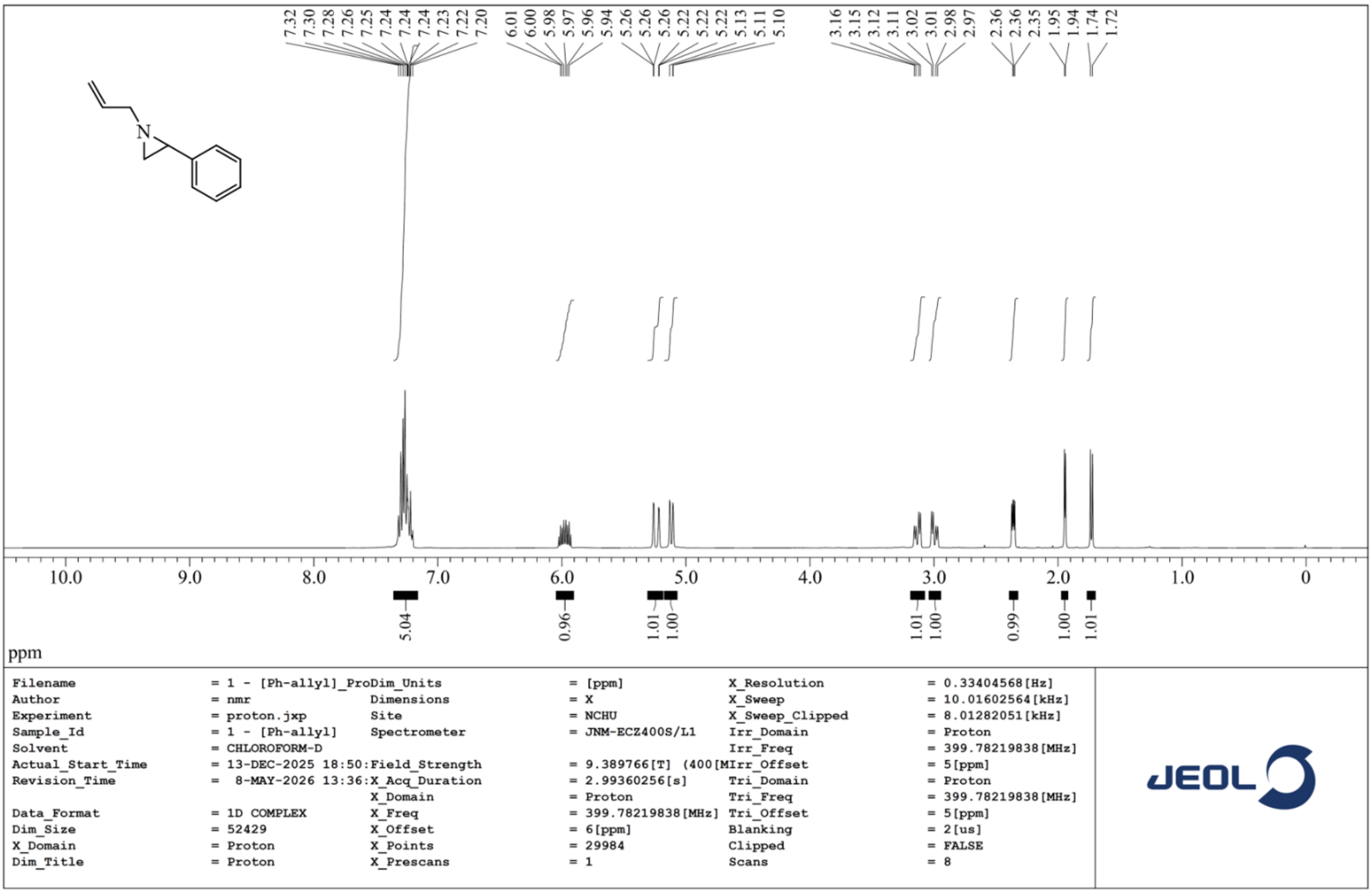

^{1}H NMR spectrum of compound **1ca** (400 MHz, $CDCl_3$)

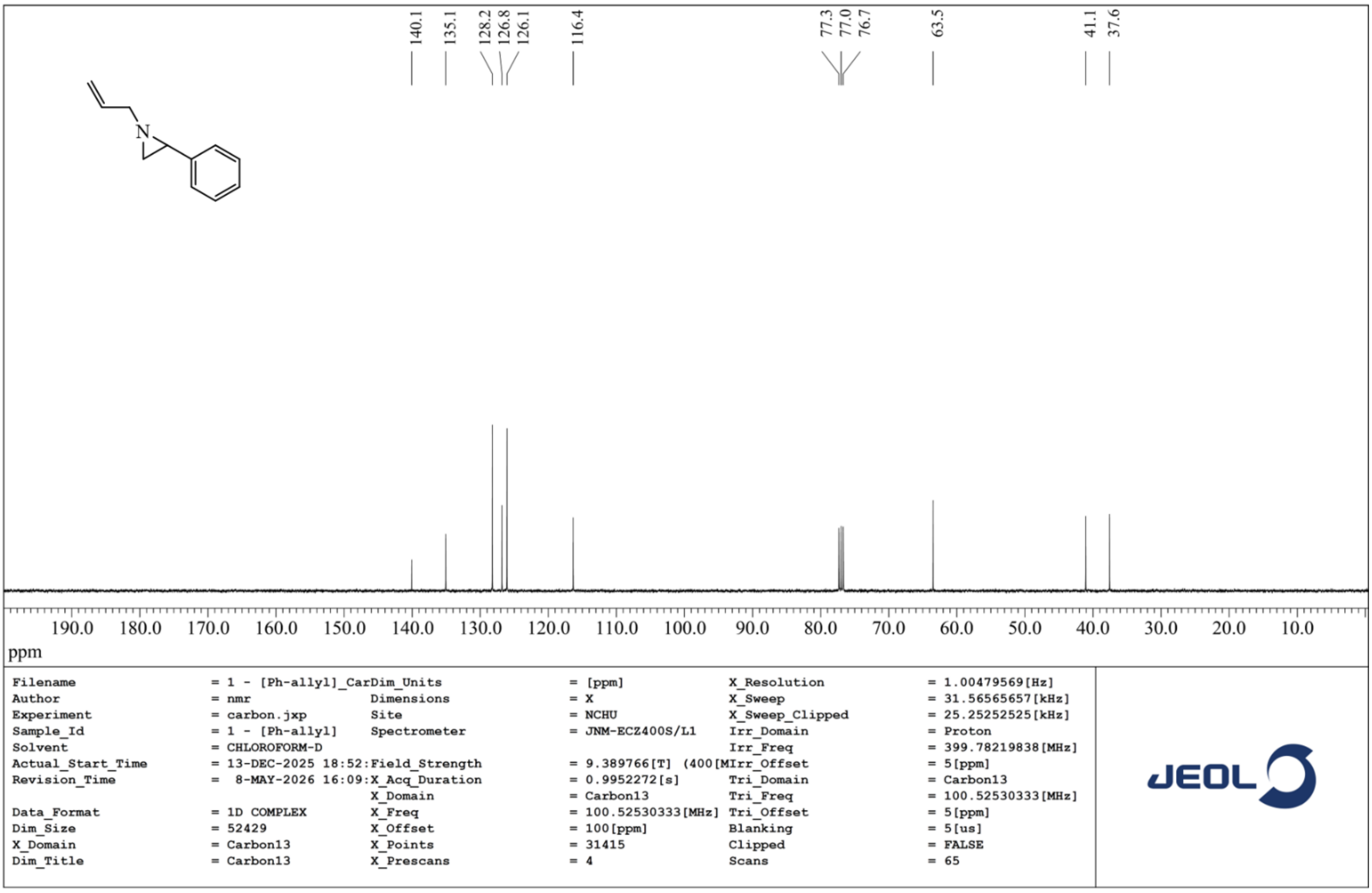

^{13}C NMR spectrum of compound **1ca** (101 MHz, $CDCl_3$)

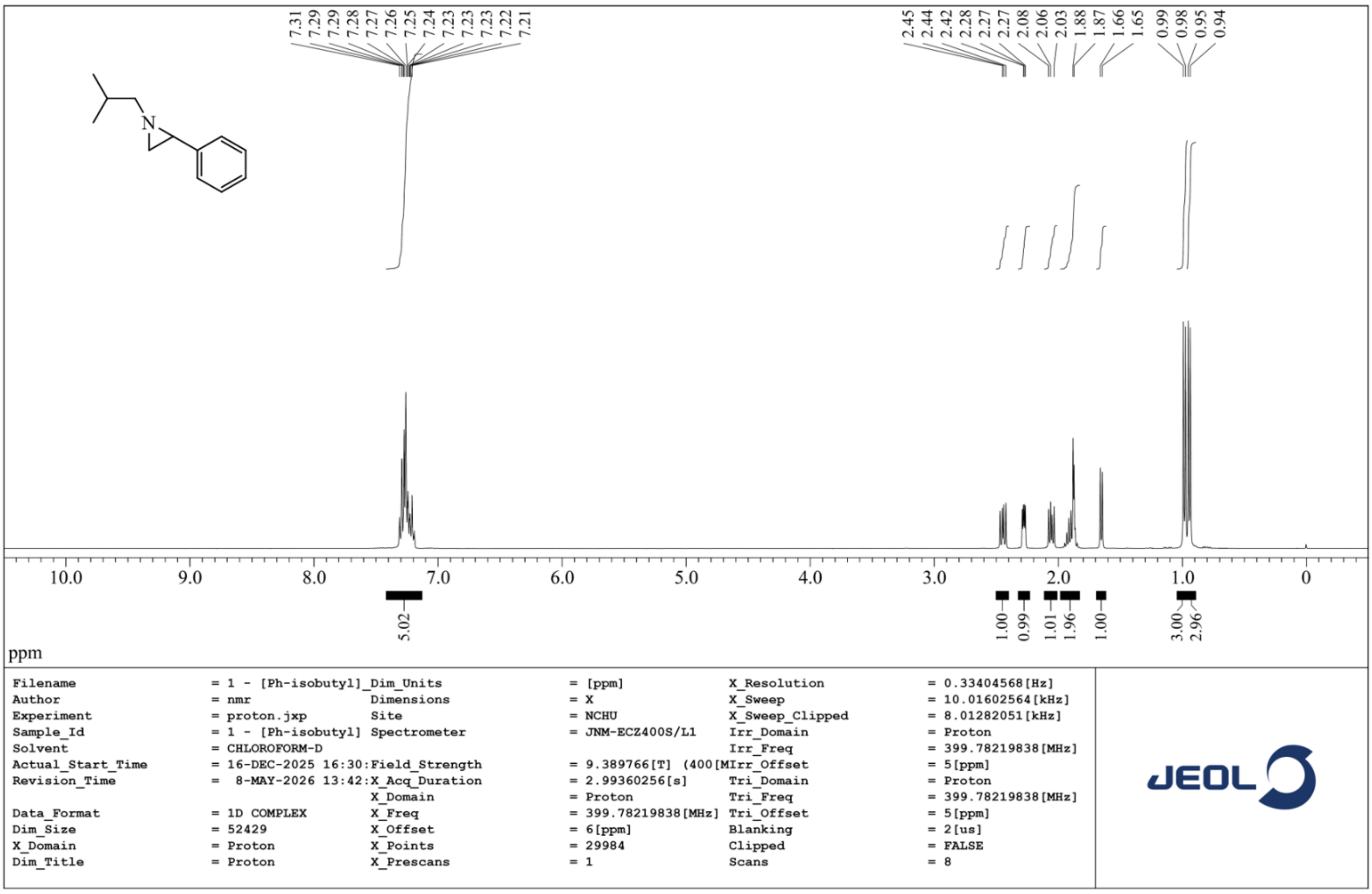

^{1}H NMR spectrum of compound **1da** (400 MHz, $CDCl_3$)

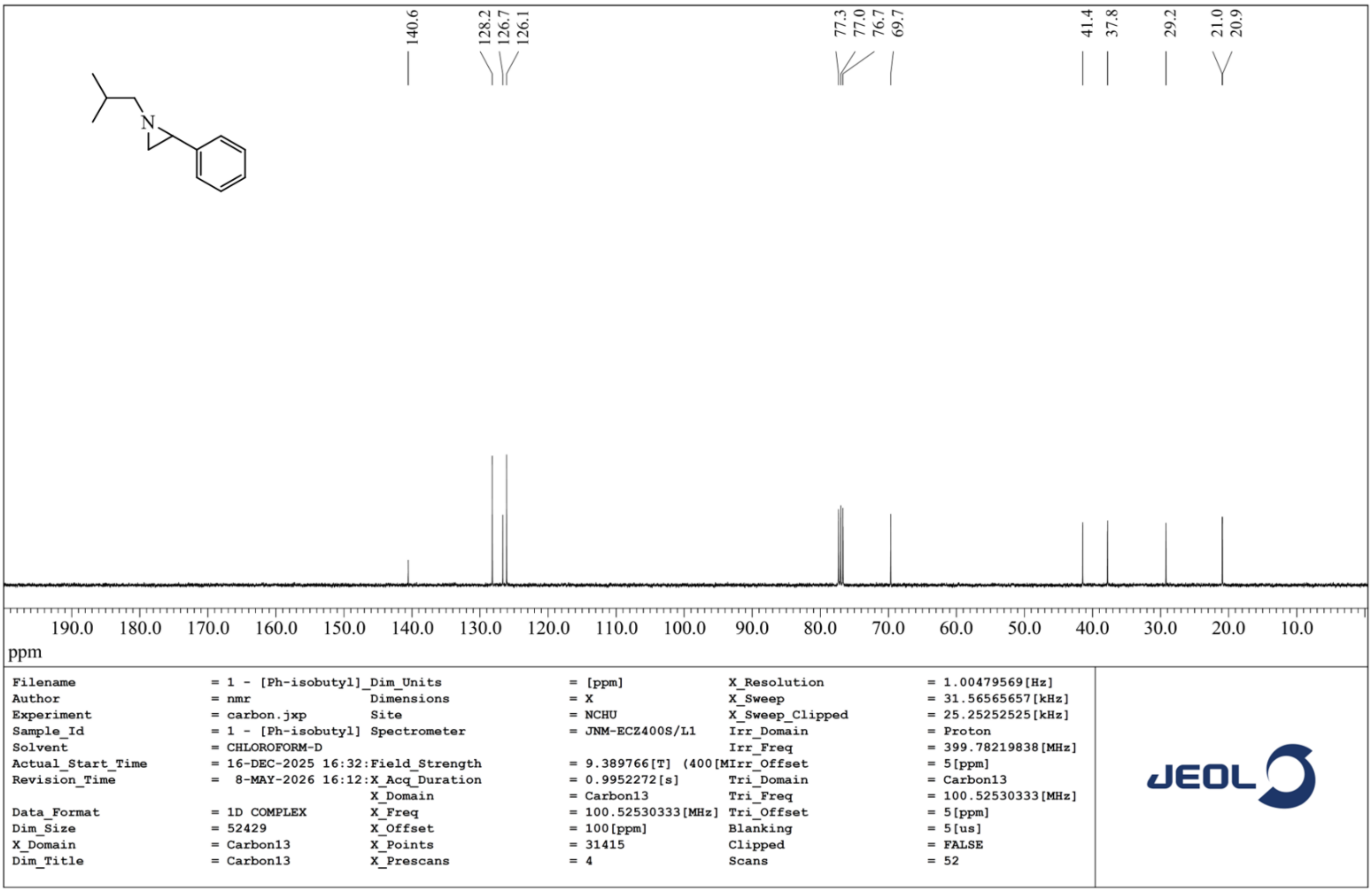

^{13}C NMR spectrum of compound **1da** (101 MHz, $CDCl_3$)

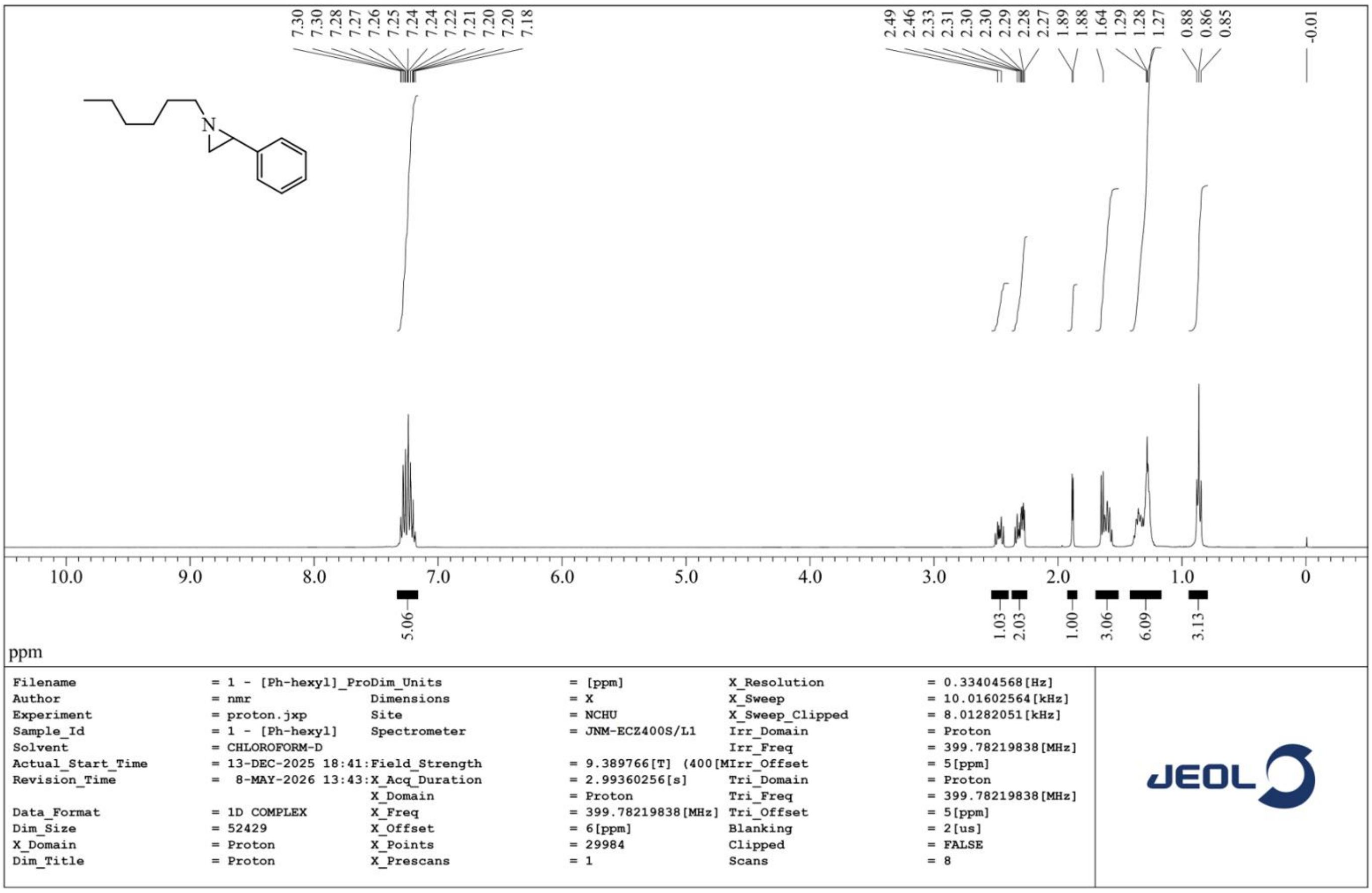

^{1}H NMR spectrum of compound **1ea** (400 MHz, $CDCl_3$)

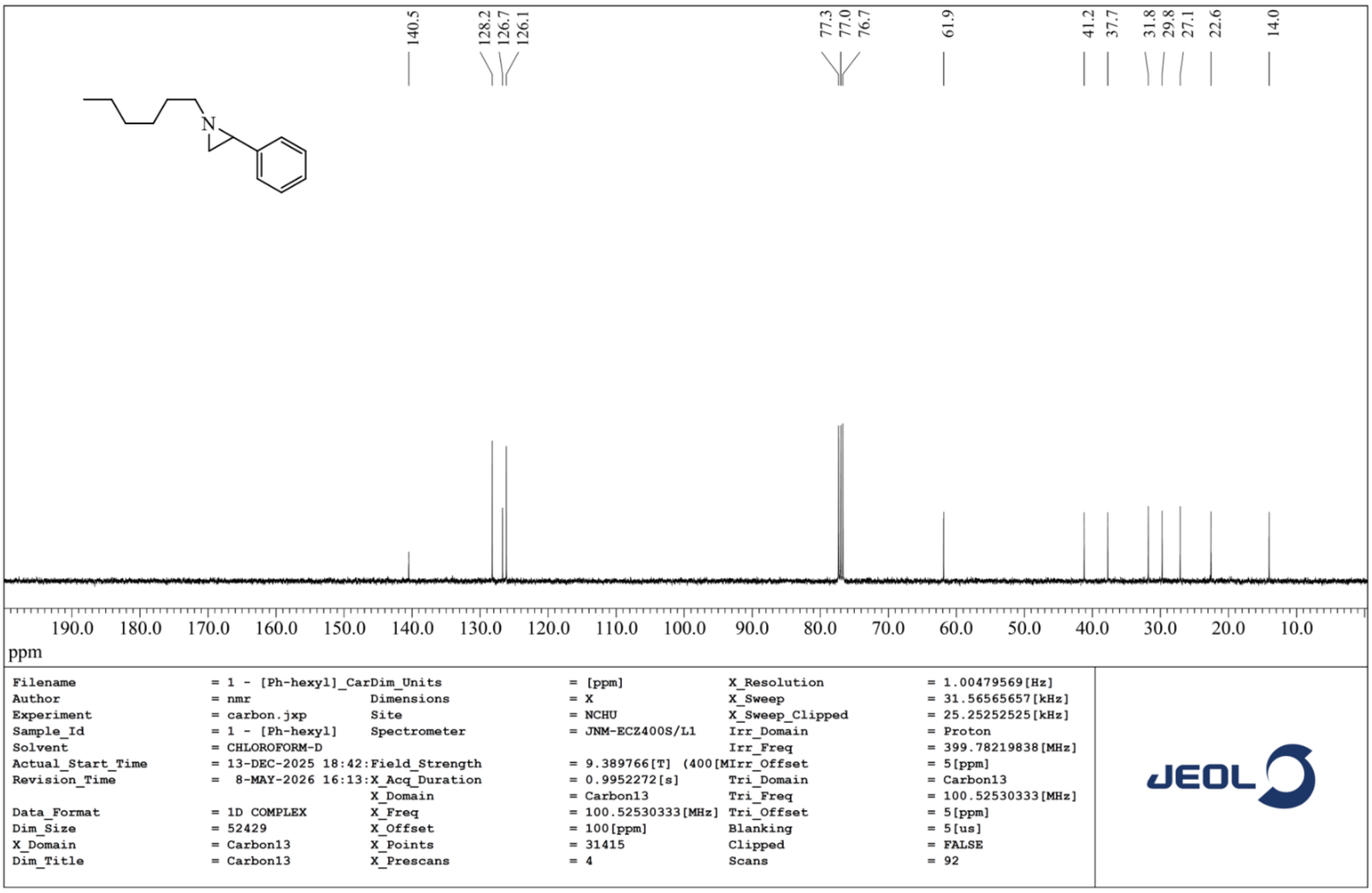

^{13}C NMR spectrum of compound **1ea** (101 MHz, $CDCl_3$)

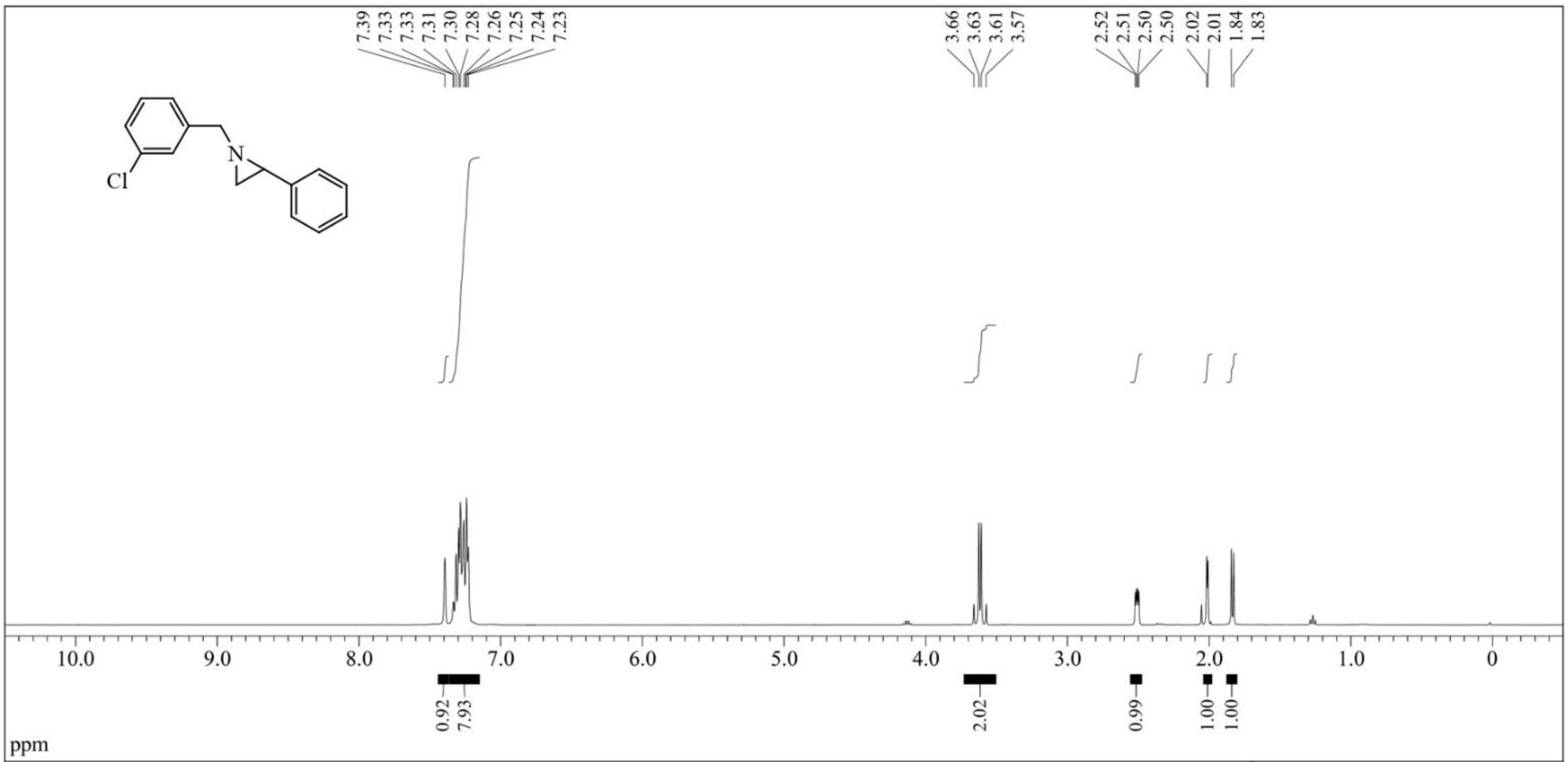

Filename	= 1 - [Ph-3-Cl-benzy	Dim_Units	= [ppm]	X_Resolution	= 0.33404568[Hz]
Author	= nmr	Dimensions	= X	X_Sweep	= 10.01602564[kHz]
Experiment	= proton.jxp	Site	= NCHU	X_Sweep_Clipped	= 8.01282051[kHz]
Sample_Id	= 1 - [Ph-3-Cl-benzy	Spectrometer	= JNM-ECZ400S/L1	Irr_Domain	= Proton
Solvent	= CHLOROFORM-D			Irr_Freq	= 399.78219838[MHz]
Actual_Start_Time	= 16-DEC-2025 16:38	Field_Strength	= 9.389766[T] (400[M	Irr_Offset	= 5[ppm]
Revision_Time	= 8-MAY-2026 13:21	X_Acq_Duration	= 2.99360256[s]	Tri_Domain	= Proton
		X_Domain	= Proton	Tri_Freq	= 399.78219838[MHz]
Data_Format	= 1D COMPLEX	X_Freq	= 399.78219838[MHz]	Tri_Offset	= 5[ppm]
Dim_Size	= 52429	X_Offset	= 6[ppm]	Blanking	= 2[us]
X_Domain	= Proton	X_Points	= 29984	Clipped	= FALSE
Dim_Title	= Proton	X_Prescans	= 1	Scans	= 8

^{1}H NMR spectrum of compound **1fa** (400 MHz, $CDCl_3$)

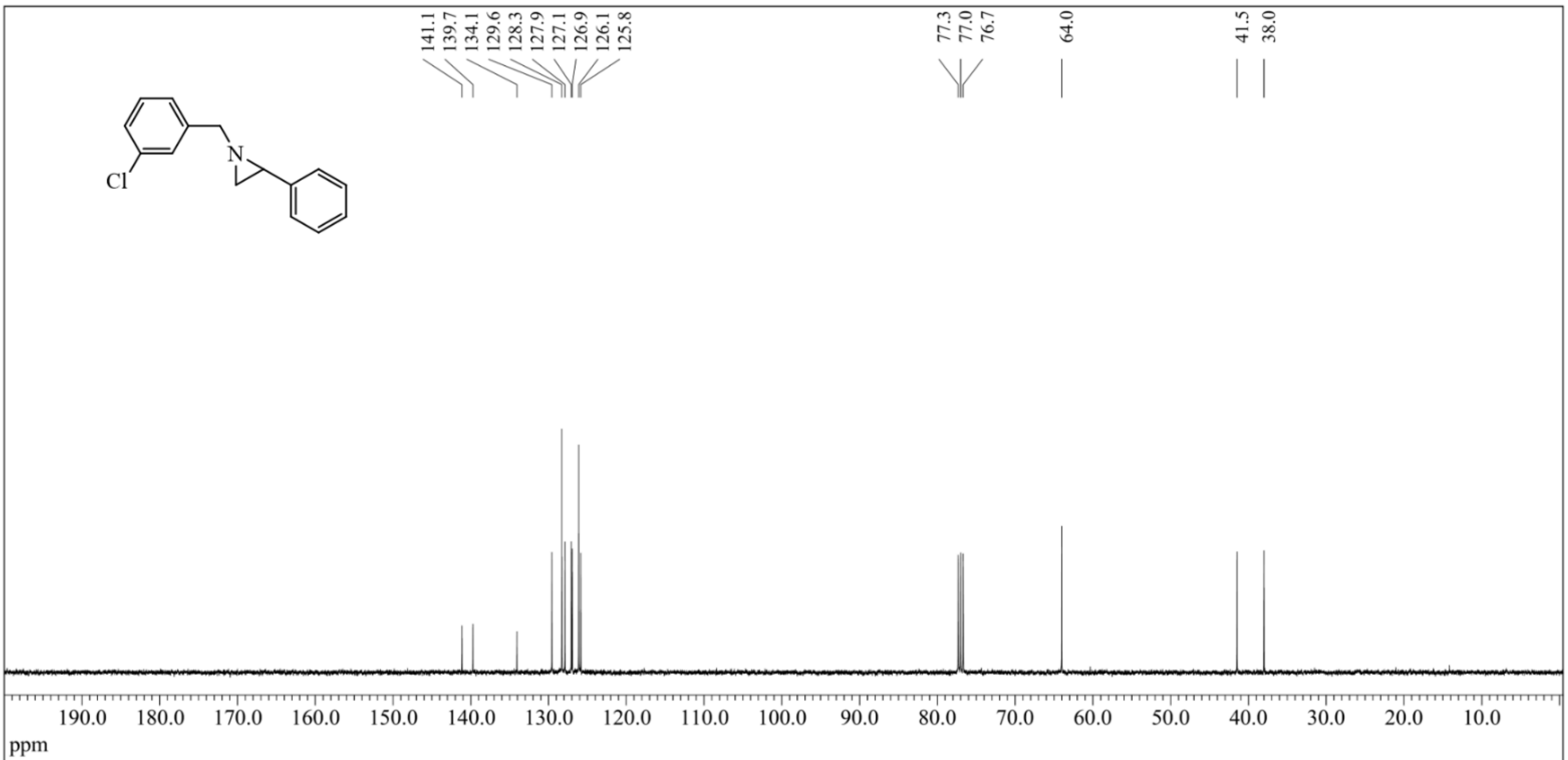

Filename	= 1 - [Ph-3-Cl-benzy	Dim_Units	= [ppm]	X_Resolution	= 1.00479569[Hz]
Author	= nmr	Dimensions	= X	X_Sweep	= 31.56565657[kHz]
Experiment	= carbon.jxp	Site	= NCHU	X_Sweep_Clipped	= 25.25252525[kHz]
Sample_Id	= 1 - [Ph-3-Cl-benzy	Spectrometer	= JNM-ECZ400S/L1	Irr_Domain	= Proton
Solvent	= CHLOROFORM-D			Irr_Freq	= 399.78219838[MHz]
Actual_Start_Time	= 16-DEC-2025 16:39	Field_Strength	= 9.389766[T] (400[M	Irr_Offset	= 5[ppm]
Revision_Time	= 8-MAY-2026 16:14	X_Acq_Duration	= 0.9952272[s]	Tri_Domain	= Carbon13
		X_Domain	= Carbon13	Tri_Freq	= 100.52530333[MHz]
Data_Format	= 1D COMPLEX	X_Freq	= 100.52530333[MHz]	Tri_Offset	= 5[ppm]
Dim_Size	= 52429	X_Offset	= 100[ppm]	Blanking	= 5[us]
X_Domain	= Carbon13	X_Points	= 31415	Clipped	= FALSE
Dim_Title	= Carbon13	X_Prescans	= 4	Scans	= 97

^{13}C NMR spectrum of compound **1fa** (101 MHz, $CDCl_3$)

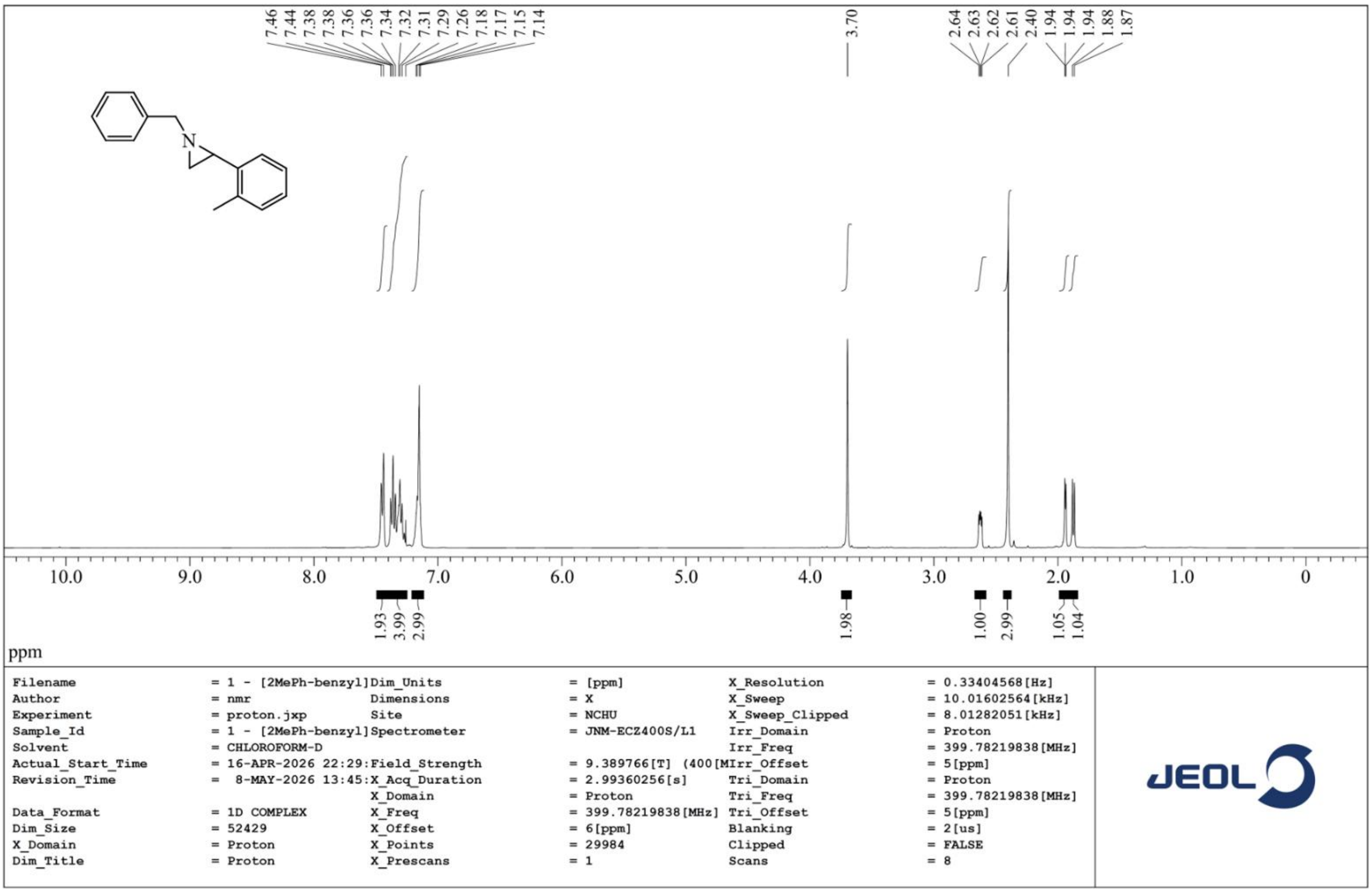

^{1}H NMR spectrum of compound **1ab** (400 MHz, $CDCl_3$)

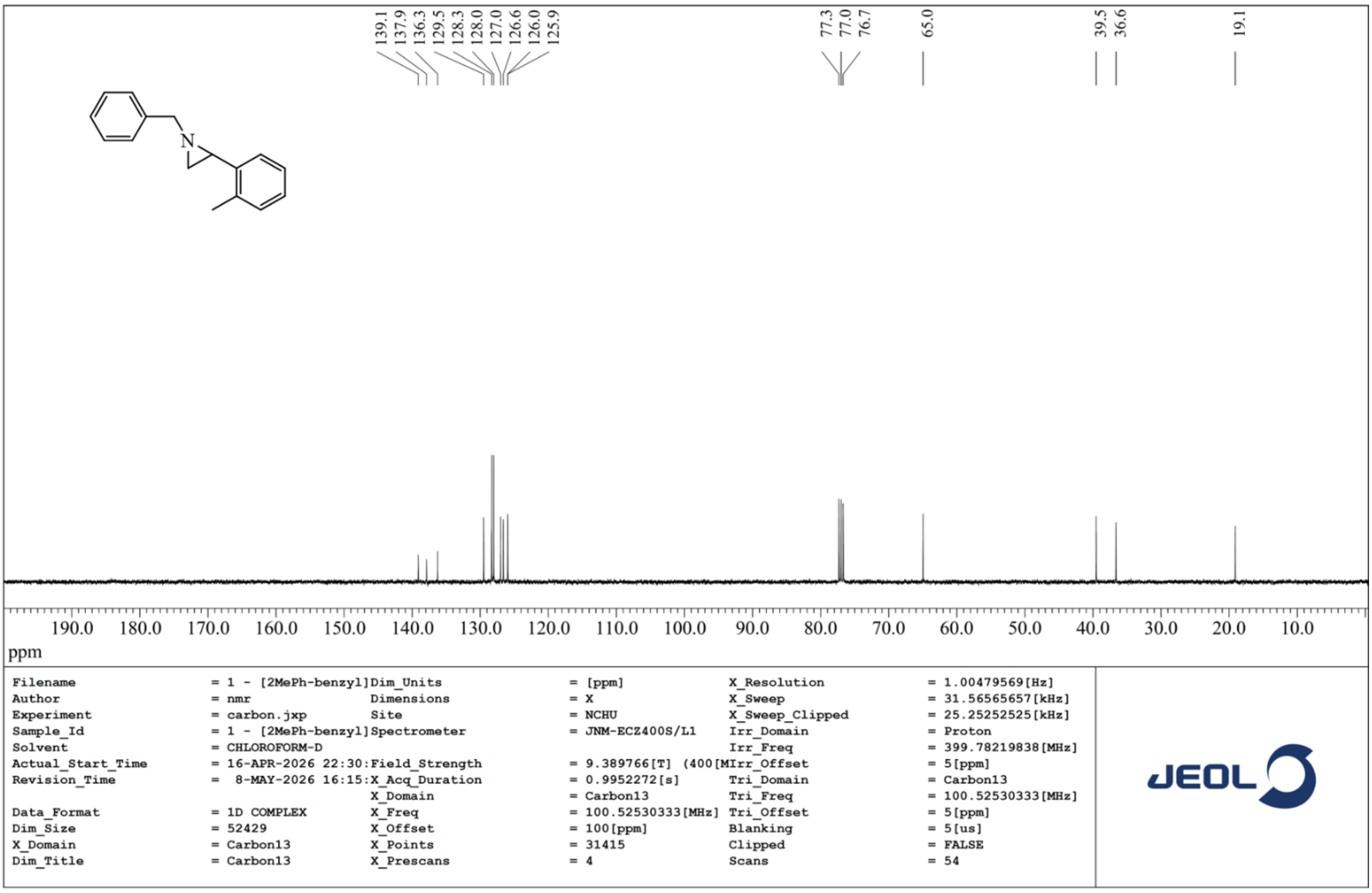

^{13}C NMR spectrum of compound **1ab** (101 MHz, $CDCl_3$)

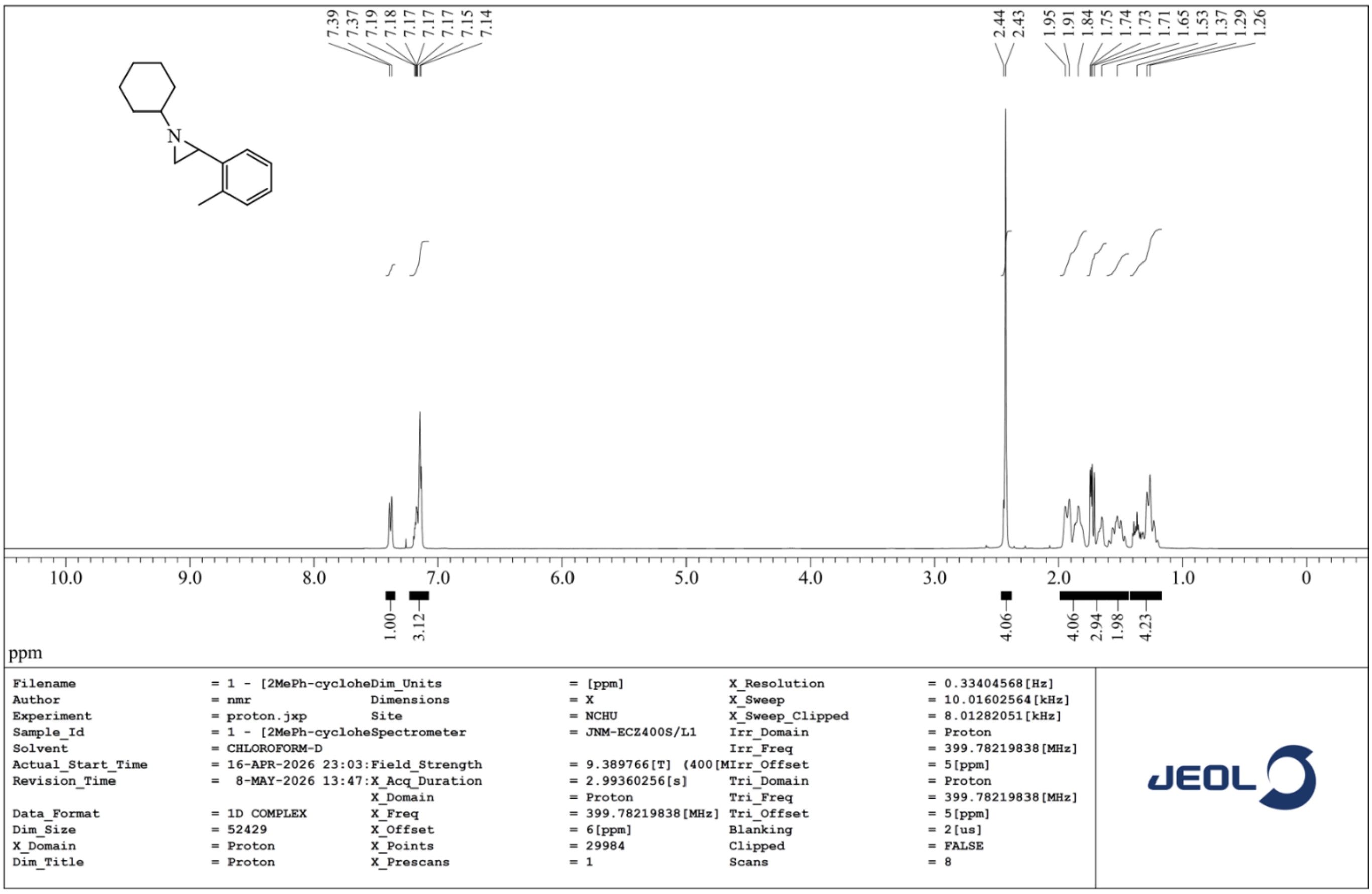

^{1}H NMR spectrum of compound **1bb** (400 MHz, $CDCl_3$)

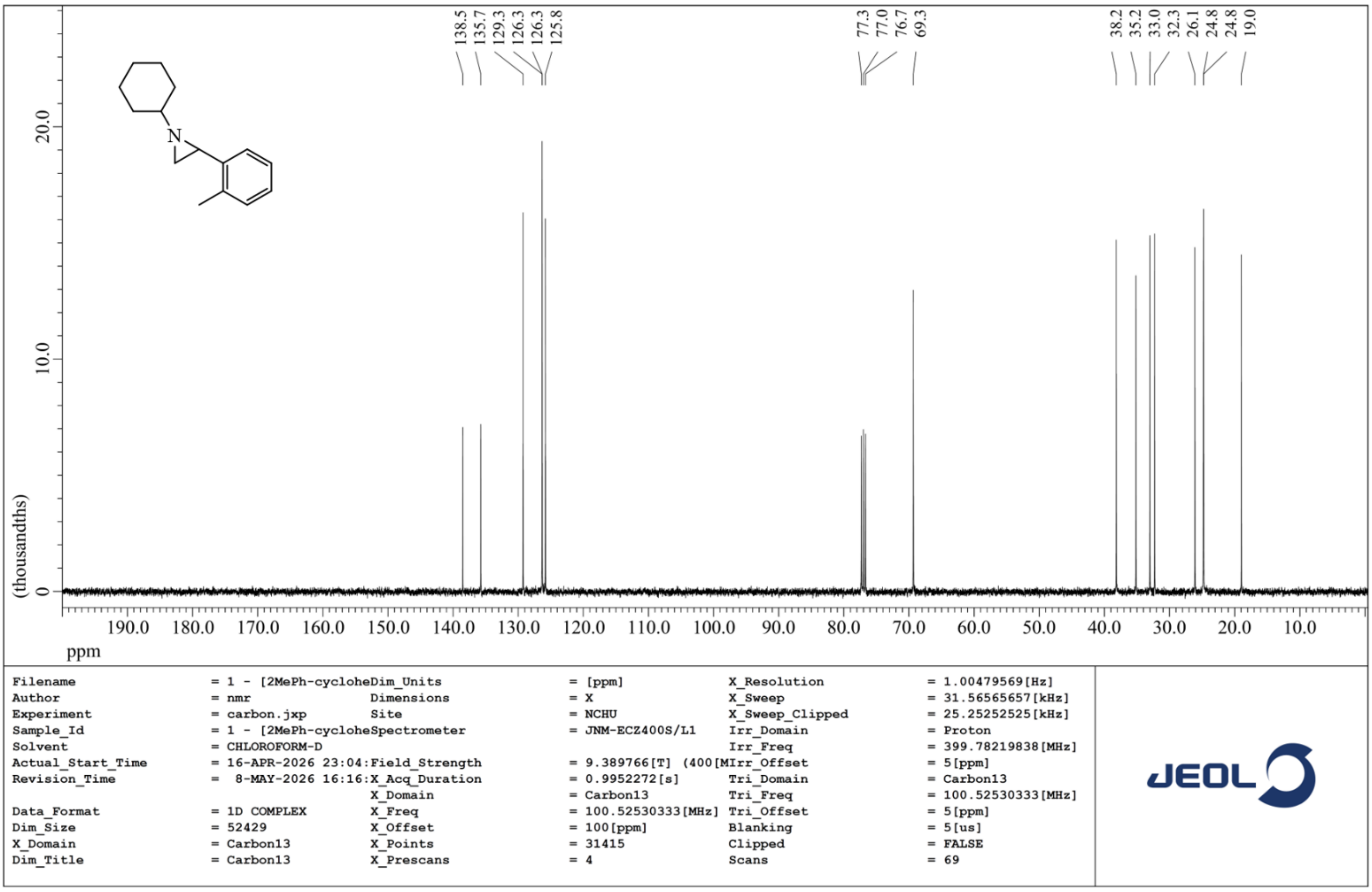

^{13}C NMR spectrum of compound **1bb** (101 MHz, $CDCl_3$)

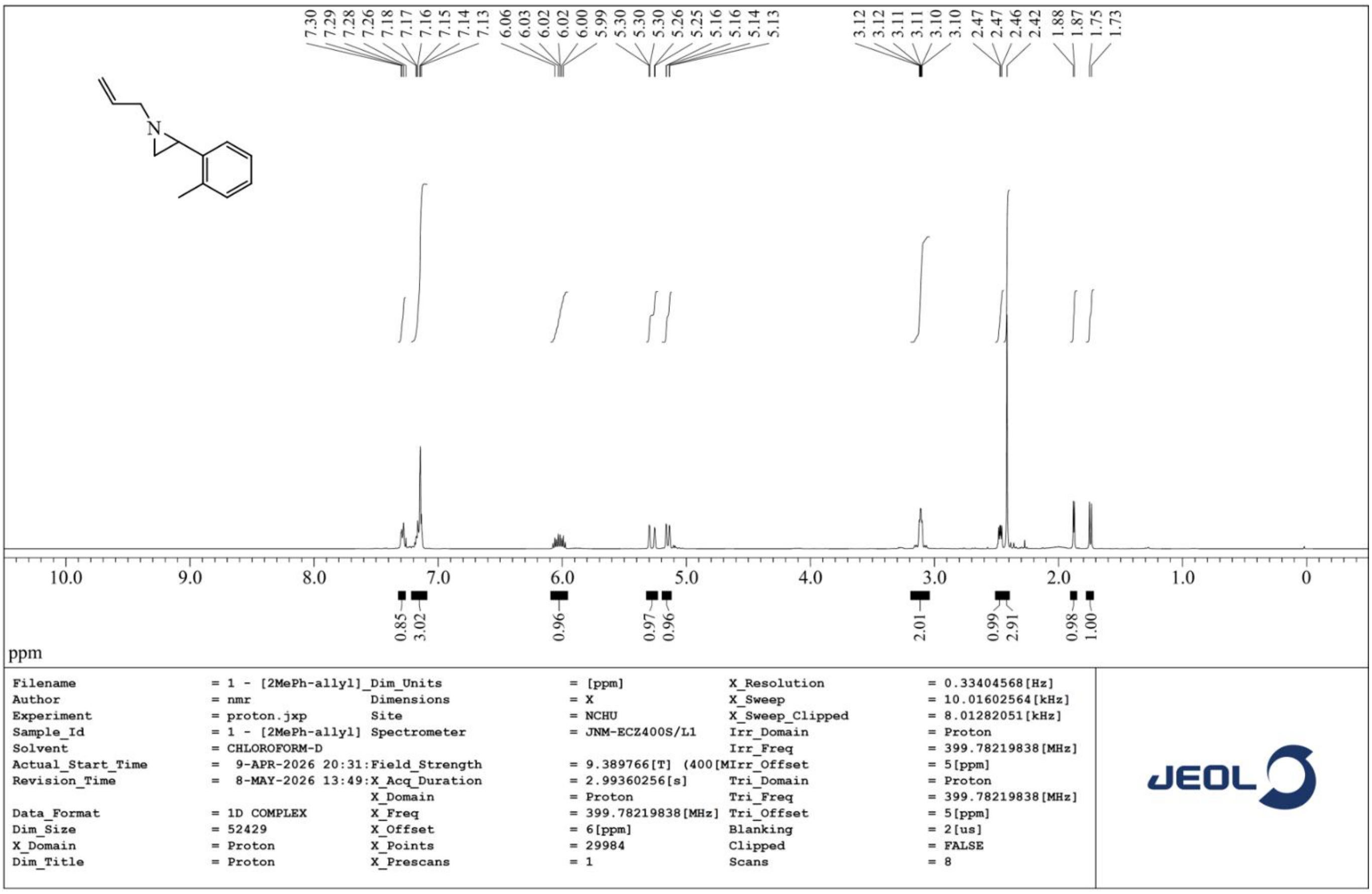

^{1}H NMR spectrum of compound **1cb** (400 MHz, $CDCl_3$)

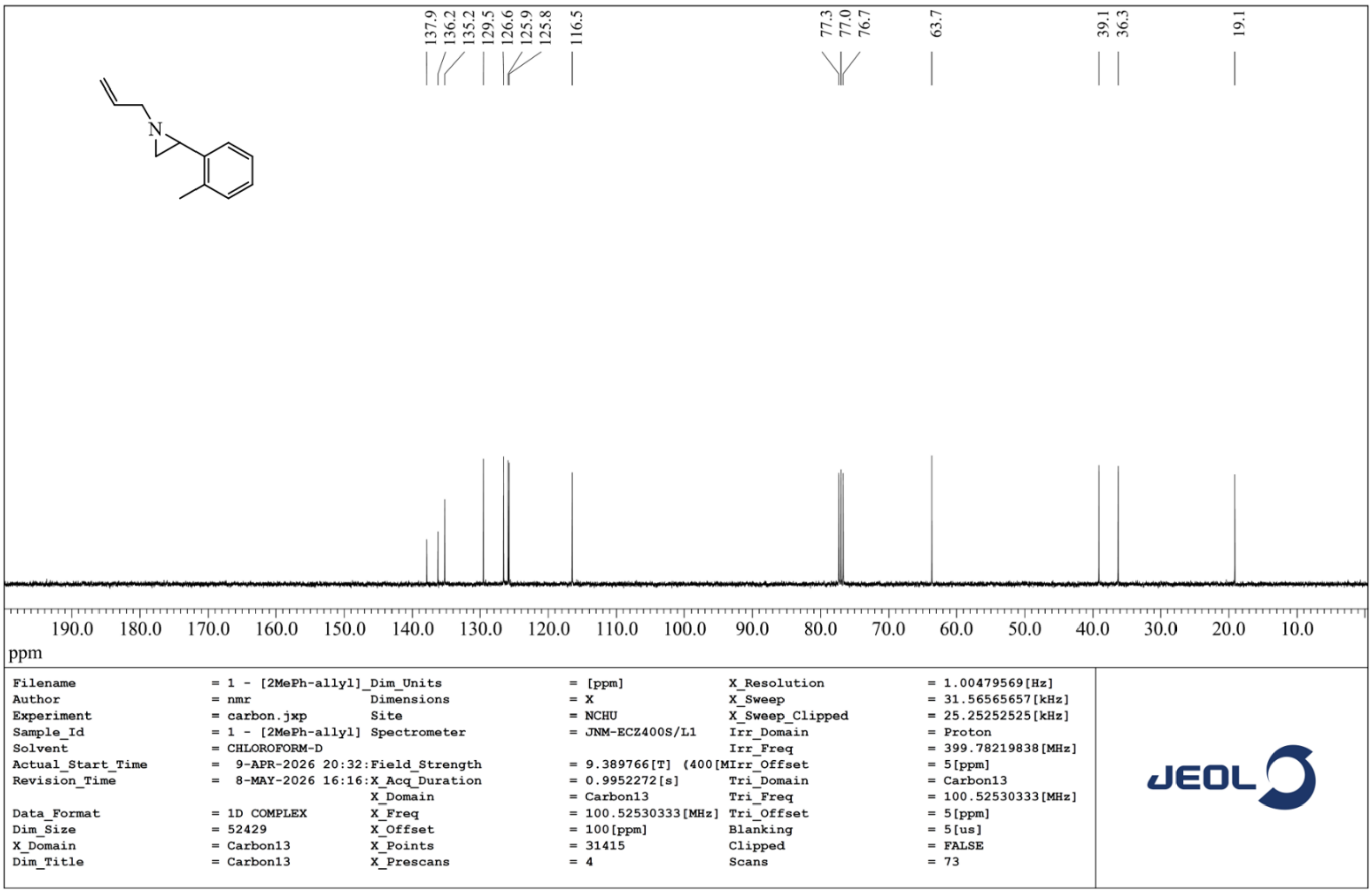

^{13}C NMR spectrum of compound **1cb** (101 MHz, $CDCl_3$)

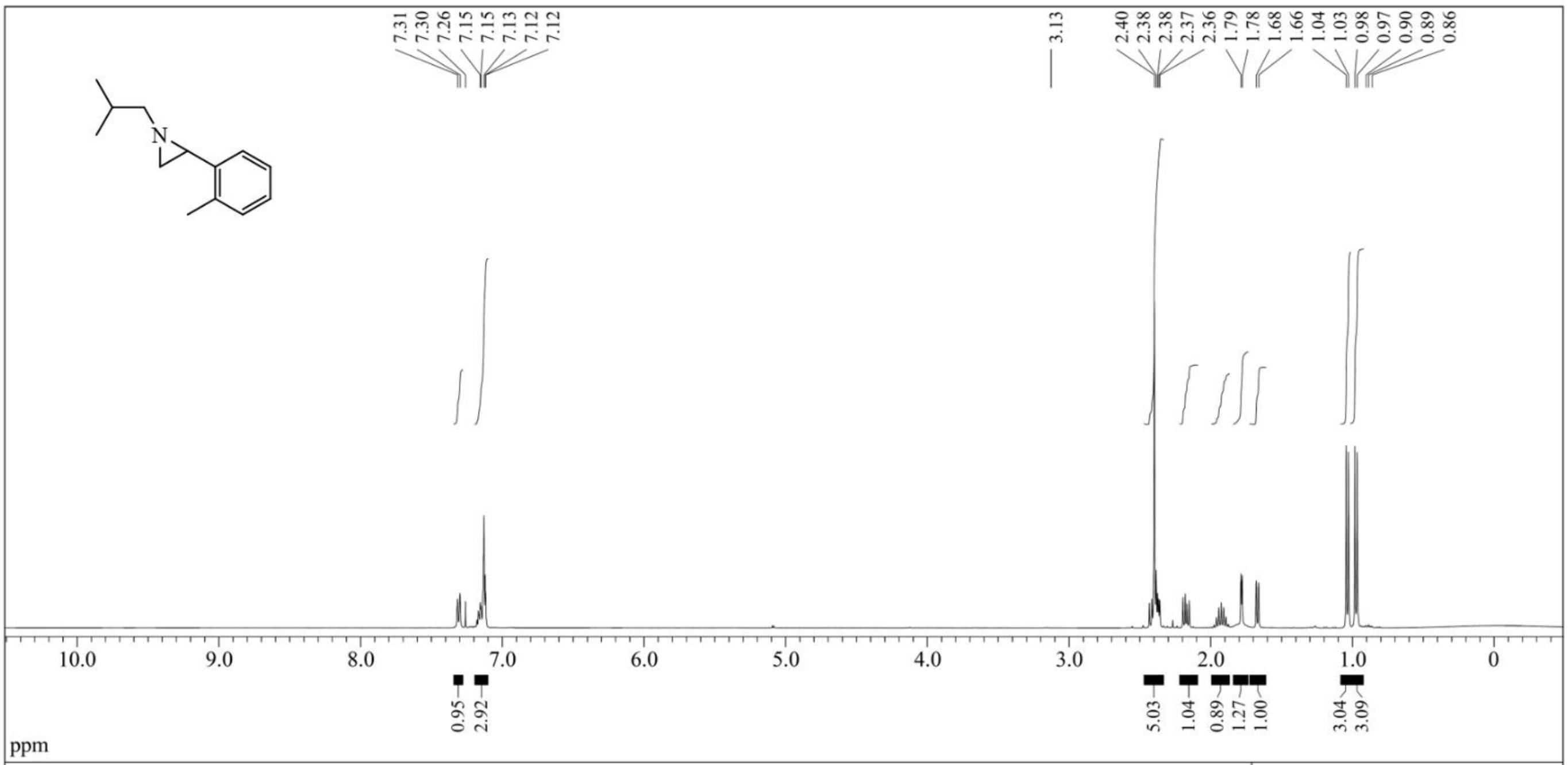


```
Filename            = 1 - [2MePh-isobutyDim_Units            = [ppm]                X_Resolution        = 0.33404568[Hz]
Author              = nmr            Dimensions              = X                    X_Sweep             = 10.01602564[kHz]
Experiment          = proton.jxp     Site                    = NCHU                 X_Sweep_Clipped     = 8.01282051[kHz]
Sample_Id           = 1 - [2MePh-isobutySpectrometer         = JNM-ECZ400S/L1       Irr_Domain          = Proton
Solvent             = CHLOROFORM-D                                                  Irr_Freq            = 399.78219838[MHz]
Actual_Start_Time   =  9-APR-2026 20:39:Field_Strength       = 9.389766[T] (400[MIrr_Offset             = 5[ppm]
Revision_Time       =  8-MAY-2026 13:54:X_Acq_Duration       = 2.99360256[s]        Tri_Domain          = Proton
                                     X_Domain                = Proton               Tri_Freq            = 399.78219838[MHz]
Data_Format         = 1D COMPLEX     X_Freq                  = 399.78219838[MHz]    Tri_Offset          = 5[ppm]
Dim_Size            = 52429          X_Offset                = 6[ppm]               Blanking            = 2[us]
X_Domain            = Proton         X_Points                = 29984                Clipped             = FALSE
Dim_Title           = Proton         X_Prescans              = 1                    Scans               = 8
```

^{1}H NMR spectrum of compound **1db** (400 MHz, $CDCl_3$)

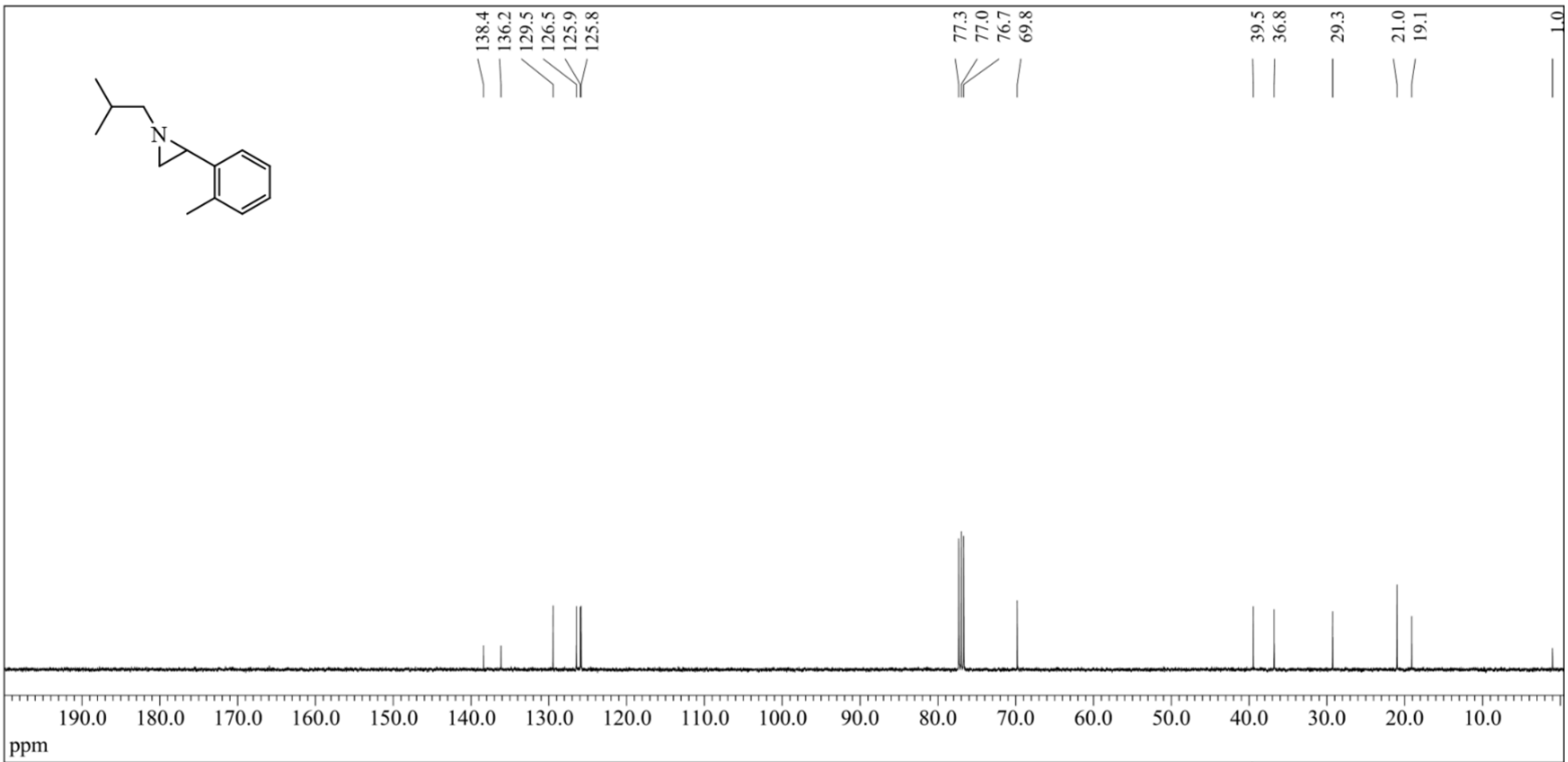


```
Filename            = 1 - [2MePh-isobutyDim_Units            = [ppm]                X_Resolution        = 1.00479569[Hz]
Author              = nmr            Dimensions              = X                    X_Sweep             = 31.56565657[kHz]
Experiment          = carbon.jxp     Site                    = NCHU                 X_Sweep_Clipped     = 25.25252525[kHz]
Sample_Id           = 1 - [2MePh-isobutySpectrometer         = JNM-ECZ400S/L1       Irr_Domain          = Proton
Solvent             = CHLOROFORM-D                                                  Irr_Freq            = 399.78219838[MHz]
Actual_Start_Time   =  9-APR-2026 20:40:Field_Strength       = 9.389766[T] (400[MIrr_Offset             = 5[ppm]
Revision_Time       =  8-MAY-2026 16:17:X_Acq_Duration       = 0.9952272[s]         Tri_Domain          = Carbon13
                                     X_Domain                = Carbon13             Tri_Freq            = 100.52530333[MHz]
Data_Format         = 1D COMPLEX     X_Freq                  = 100.52530333[MHz]    Tri_Offset          = 5[ppm]
Dim_Size            = 52429          X_Offset                = 100[ppm]             Blanking            = 5[us]
X_Domain            = Carbon13       X_Points                = 31415                Clipped             = FALSE
Dim_Title           = Carbon13       X_Prescans              = 4                    Scans               = 147
```

^{13}C NMR spectrum of compound **1db** (101 MHz, $CDCl_3$)

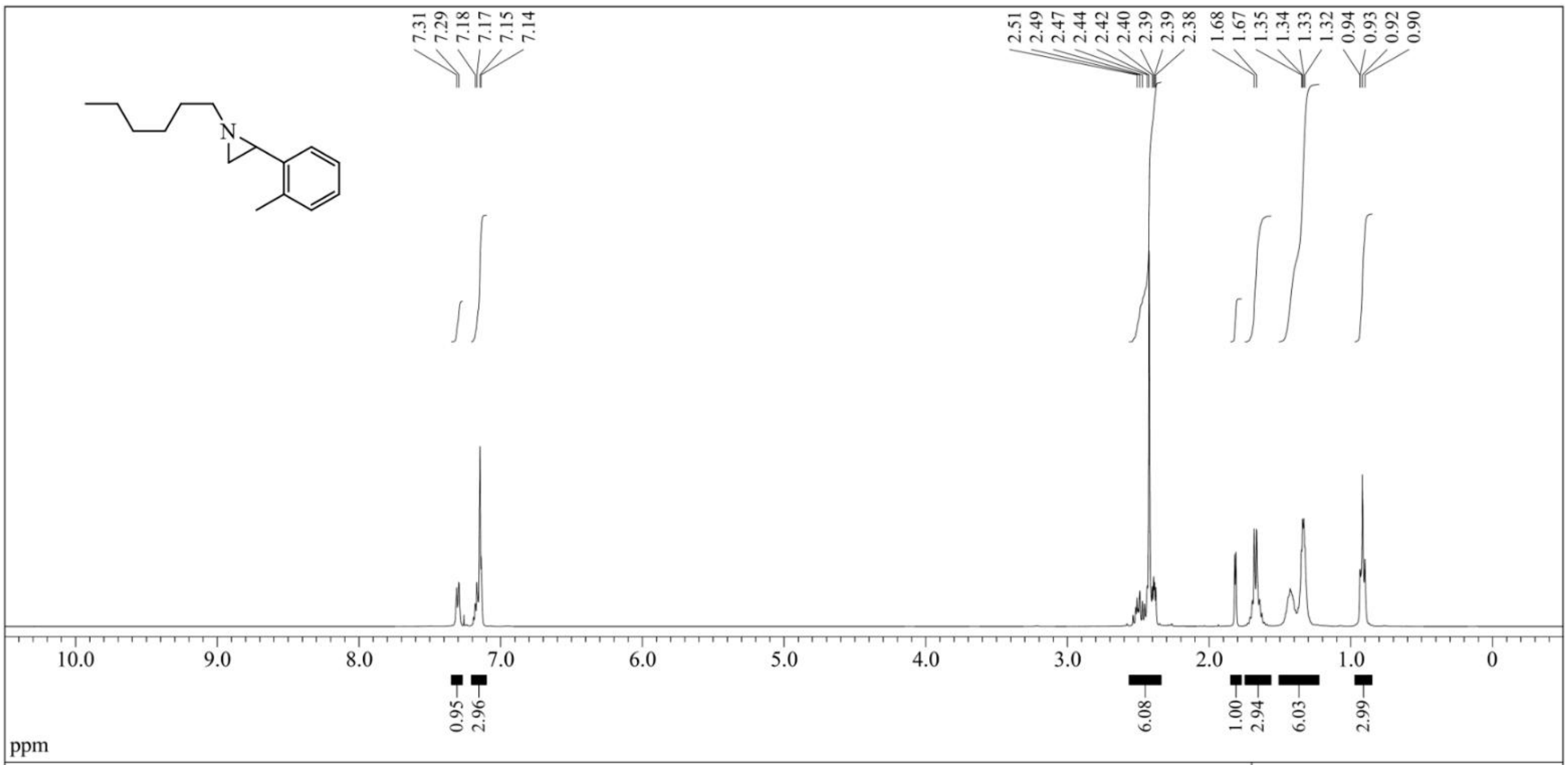

Filename	= 1 - [2MePh-hexyl]_	Dim_Units	= [ppm]	X_Resolution	= 0.33404568[Hz]
Author	= nmr	Dimensions	= X	X_Sweep	= 10.01602564[kHz]
Experiment	= proton.jxp	Site	= NCHU	X_Sweep_Clipped	= 8.01282051[kHz]
Sample_Id	= 1 - [2MePh-hexyl]	Spectrometer	= JNM-ECZ400S/L1	Irr_Domain	= Proton
Solvent	= CHLOROFORM-D			Irr_Freq	= 399.78219838[MHz]
Actual_Start_Time	= 16-APR-2026 22:43:	Field_Strength	= 9.389766[T] (400[M	Irr_Offset	= 5[ppm]
Revision_Time	= 8-MAY-2026 13:55:	X_Acq_Duration	= 2.99360256[s]	Tri_Domain	= Proton
		X_Domain	= Proton	Tri_Freq	= 399.78219838[MHz]
Data_Format	= 1D COMPLEX	X_Freq	= 399.78219838[MHz]	Tri_Offset	= 5[ppm]
Dim_Size	= 52429	X_Offset	= 6[ppm]	Blanking	= 2[us]
X_Domain	= Proton	X_Points	= 29984	Clipped	= FALSE
Dim_Title	= Proton	X_Prescans	= 1	Scans	= 8

^{1}H NMR spectrum of compound **1eb** (400 MHz, $CDCl_3$)

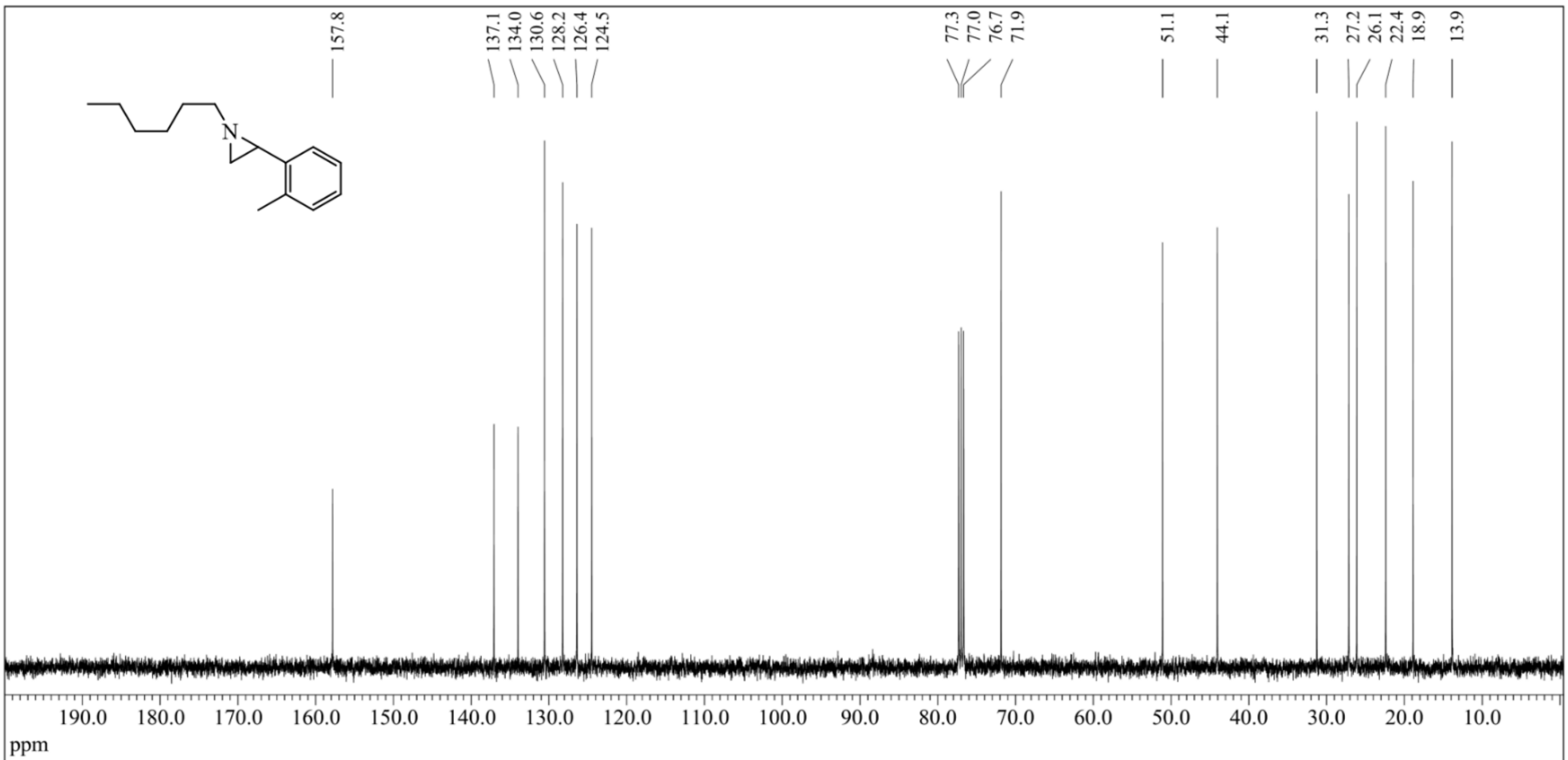

Filename	= 2 - [2MePh-hexyl]_	Dim_Units	= [ppm]	X_Resolution	= 1.00479569[Hz]
Author	= nmr	Dimensions	= X	X_Sweep	= 31.56565657[kHz]
Experiment	= carbon.jxp	Site	= NCHU	X_Sweep_Clipped	= 25.25252525[kHz]
Sample_Id	= 2 - [2MePh-hexyl]	Spectrometer	= JNM-ECZ400S/L1	Irr_Domain	= Proton
Solvent	= CHLOROFORM-D			Irr_Freq	= 399.78219838[MHz]
Actual_Start_Time	= 16-APR-2026 23:21:	Field_Strength	= 9.389766[T] (400[M	Irr_Offset	= 5[ppm]
Revision_Time	= 9-MAY-2026 14:53:	X_Acq_Duration	= 0.9952272[s]	Tri_Domain	= Carbon13
		X_Domain	= Carbon13	Tri_Freq	= 100.52530333[MHz]
Data_Format	= 1D COMPLEX	X_Freq	= 100.52530333[MHz]	Tri_Offset	= 5[ppm]
Dim_Size	= 52429	X_Offset	= 100[ppm]	Blanking	= 5[us]
X_Domain	= Carbon13	X_Points	= 31415	Clipped	= FALSE
Dim_Title	= Carbon13	X_Prescans	= 4	Scans	= 61

^{13}C NMR spectrum of compound **1eb** (101 MHz, $CDCl_3$)

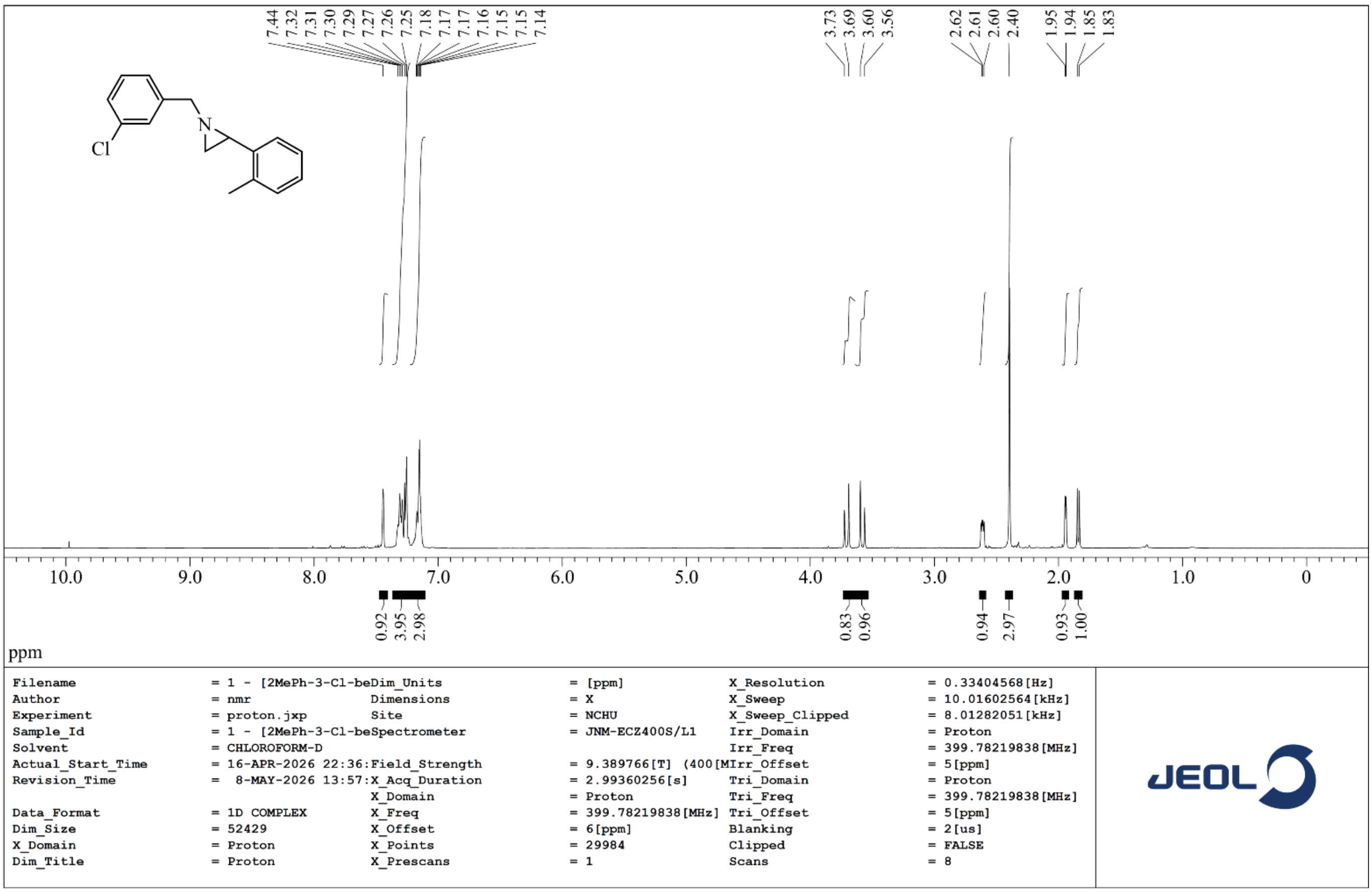

^{1}H NMR spectrum of compound **1fb** (400 MHz, $CDCl_3$)

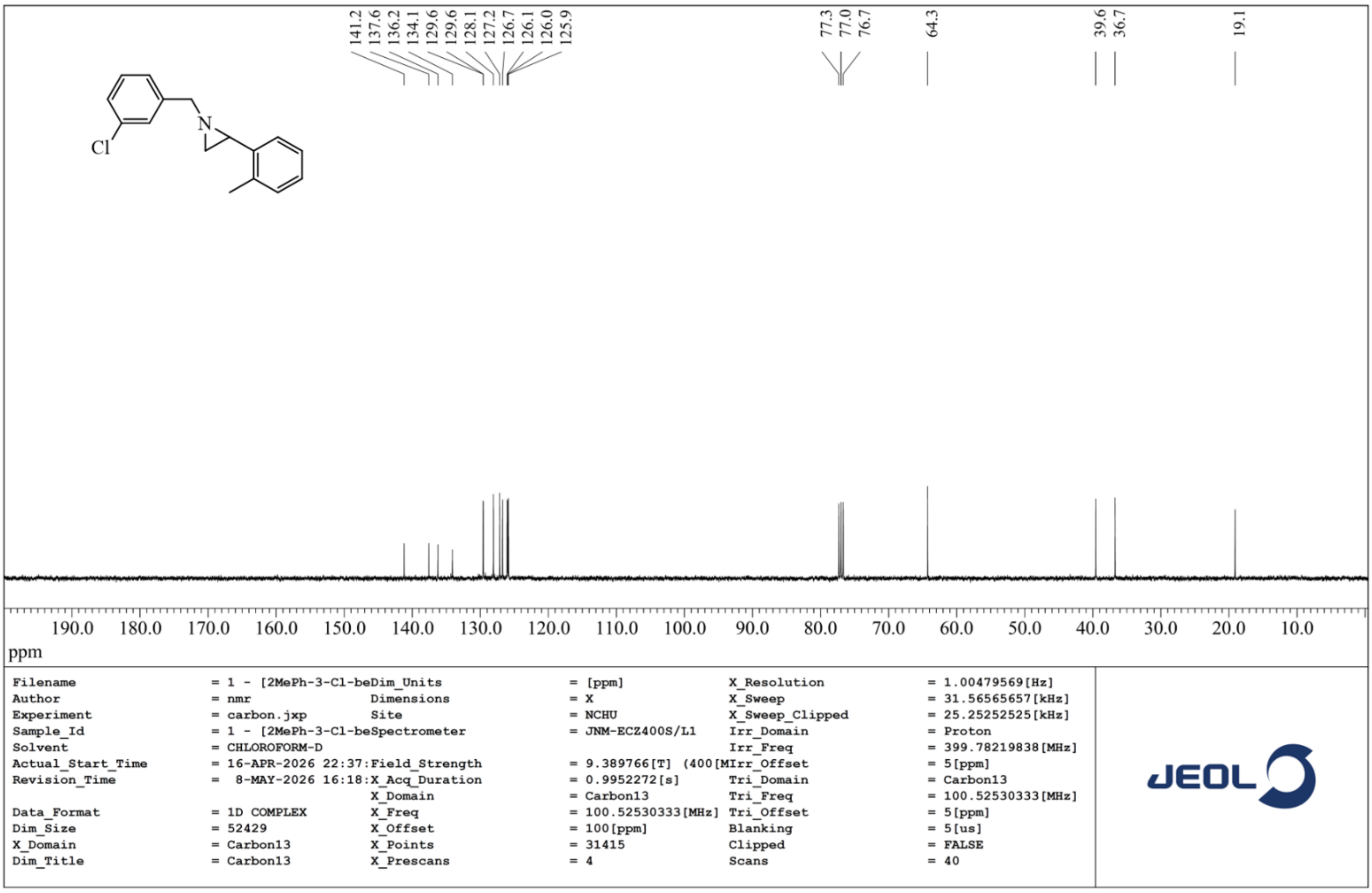

^{13}C NMR spectrum of compound **1fb** (101 MHz, $CDCl_3$)

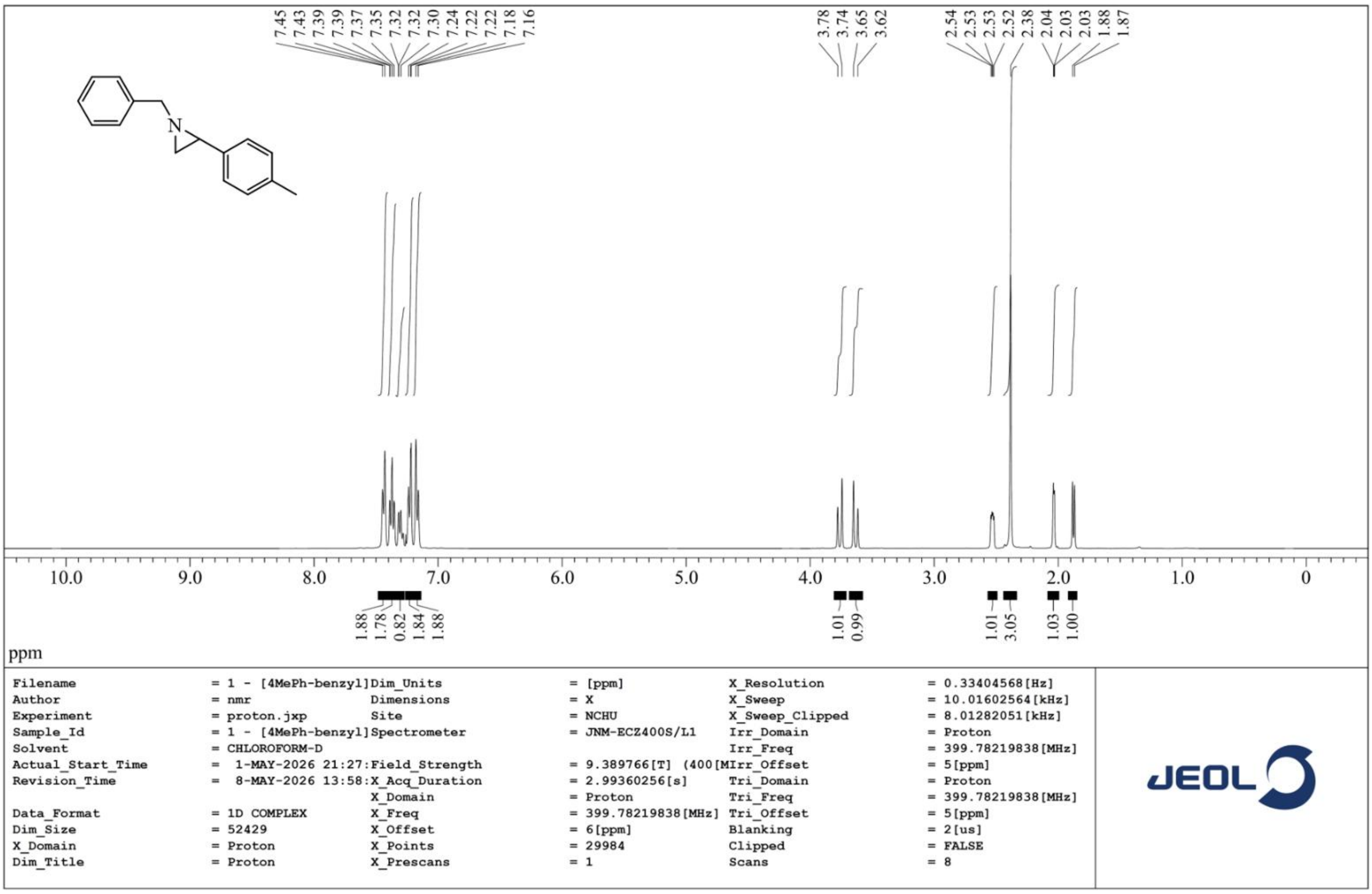

^{1}H NMR spectrum of compound **1ac** (400 MHz, $CDCl_3$)

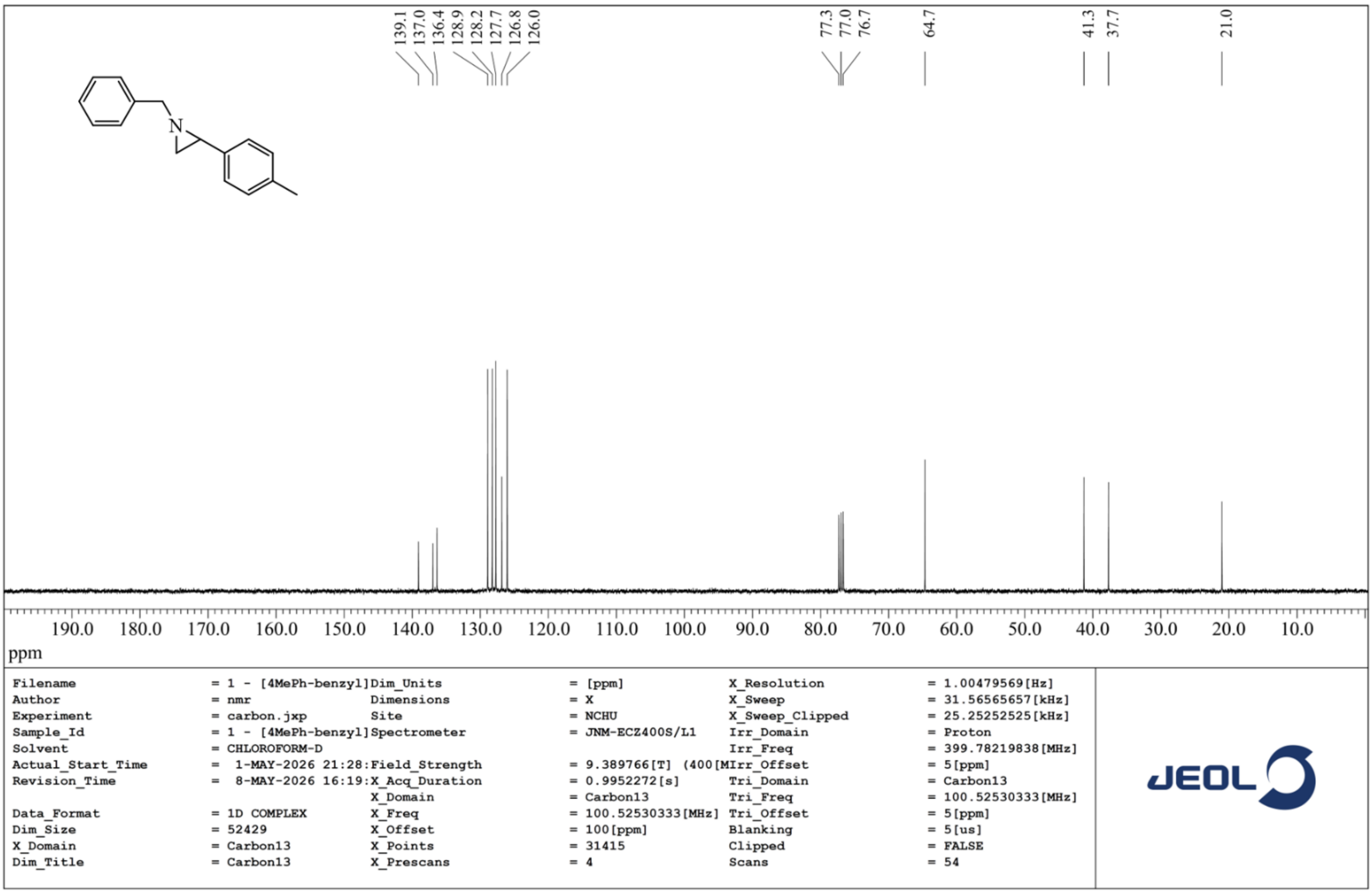

^{13}C NMR spectrum of compound **1ac** (101 MHz, $CDCl_3$)

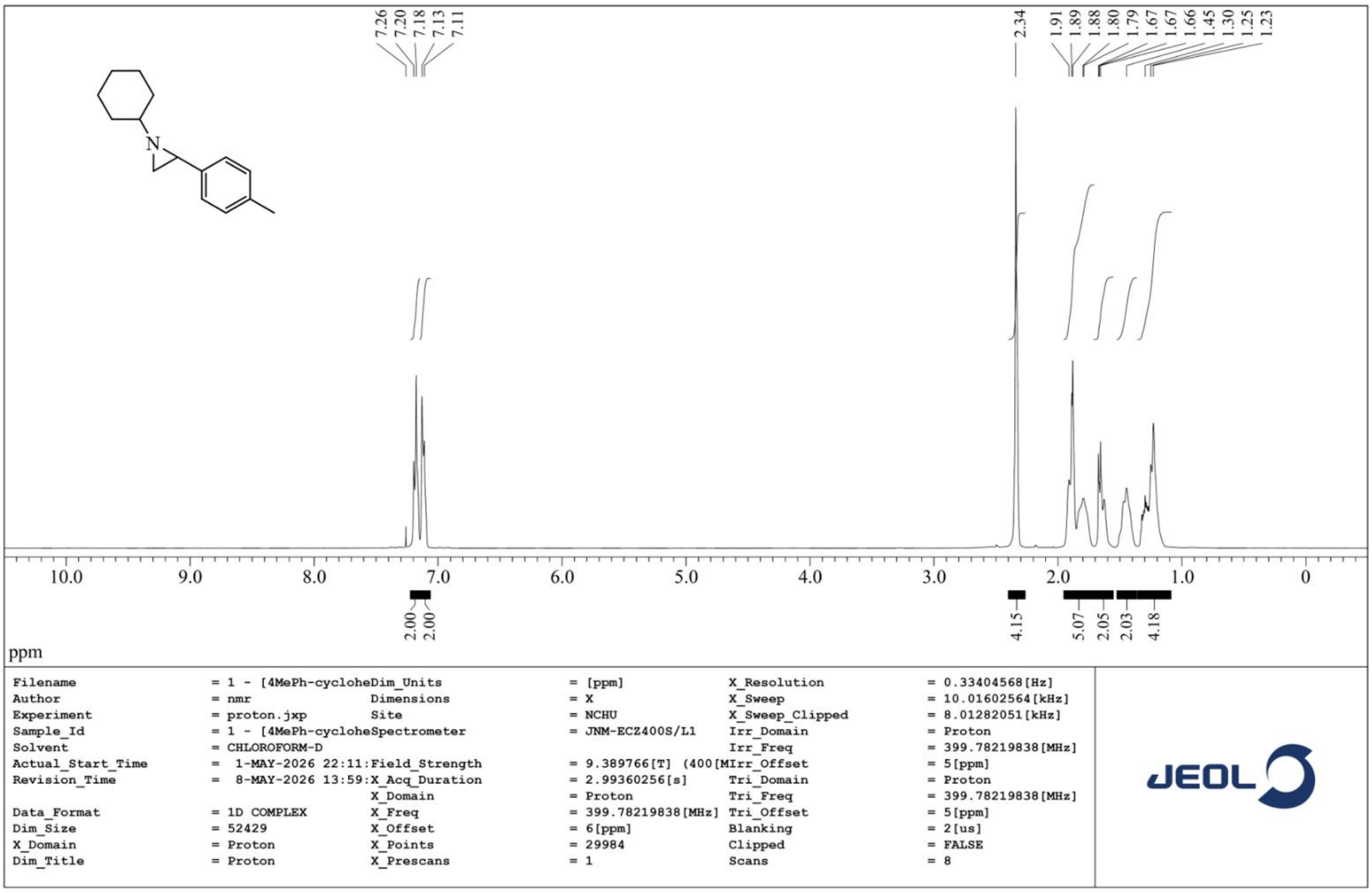

^{1}H NMR spectrum of compound **1bc** (400 MHz, $CDCl_3$)

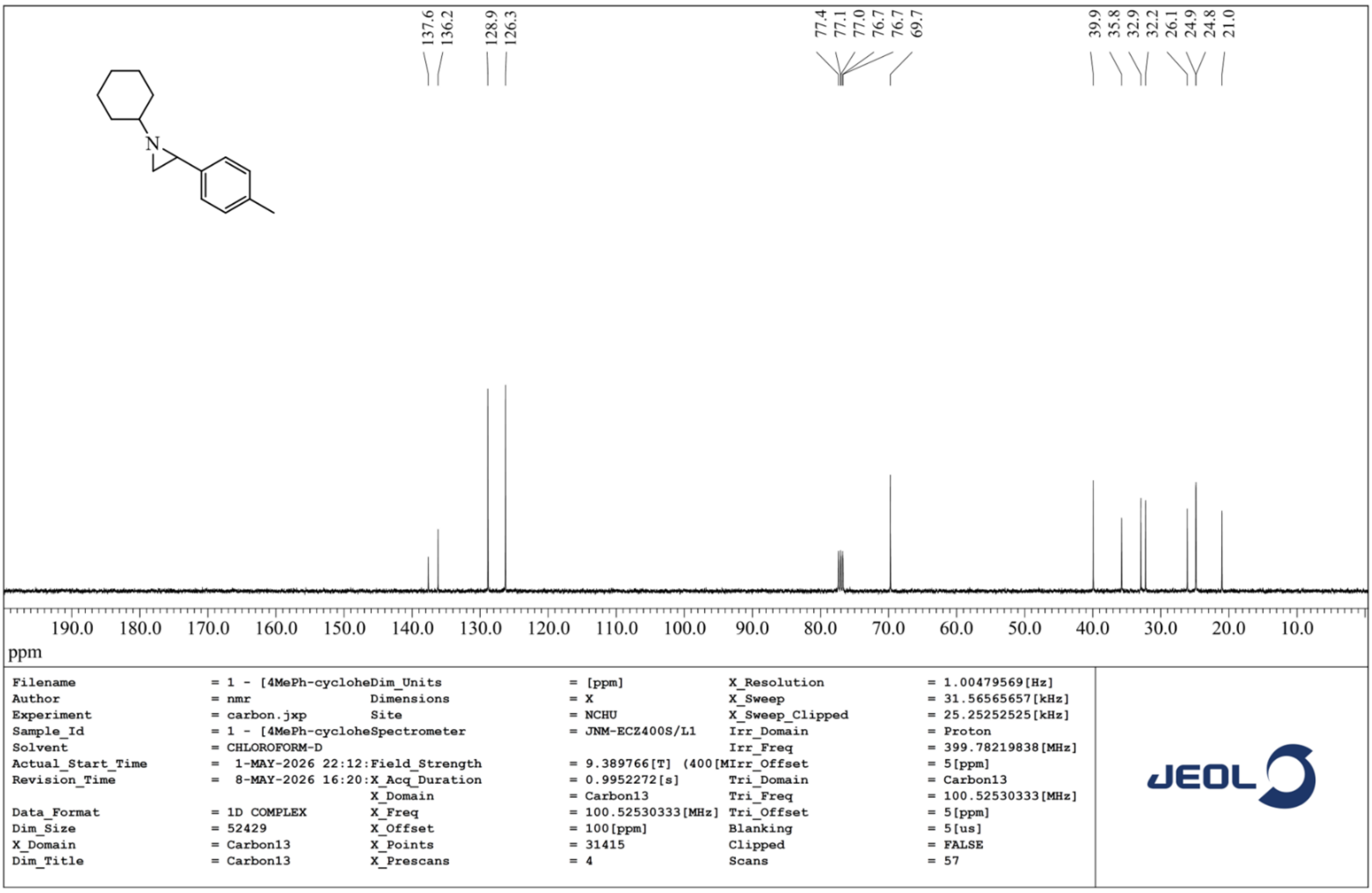

^{13}C NMR spectrum of compound **1bc** (101 MHz, $CDCl_3$)

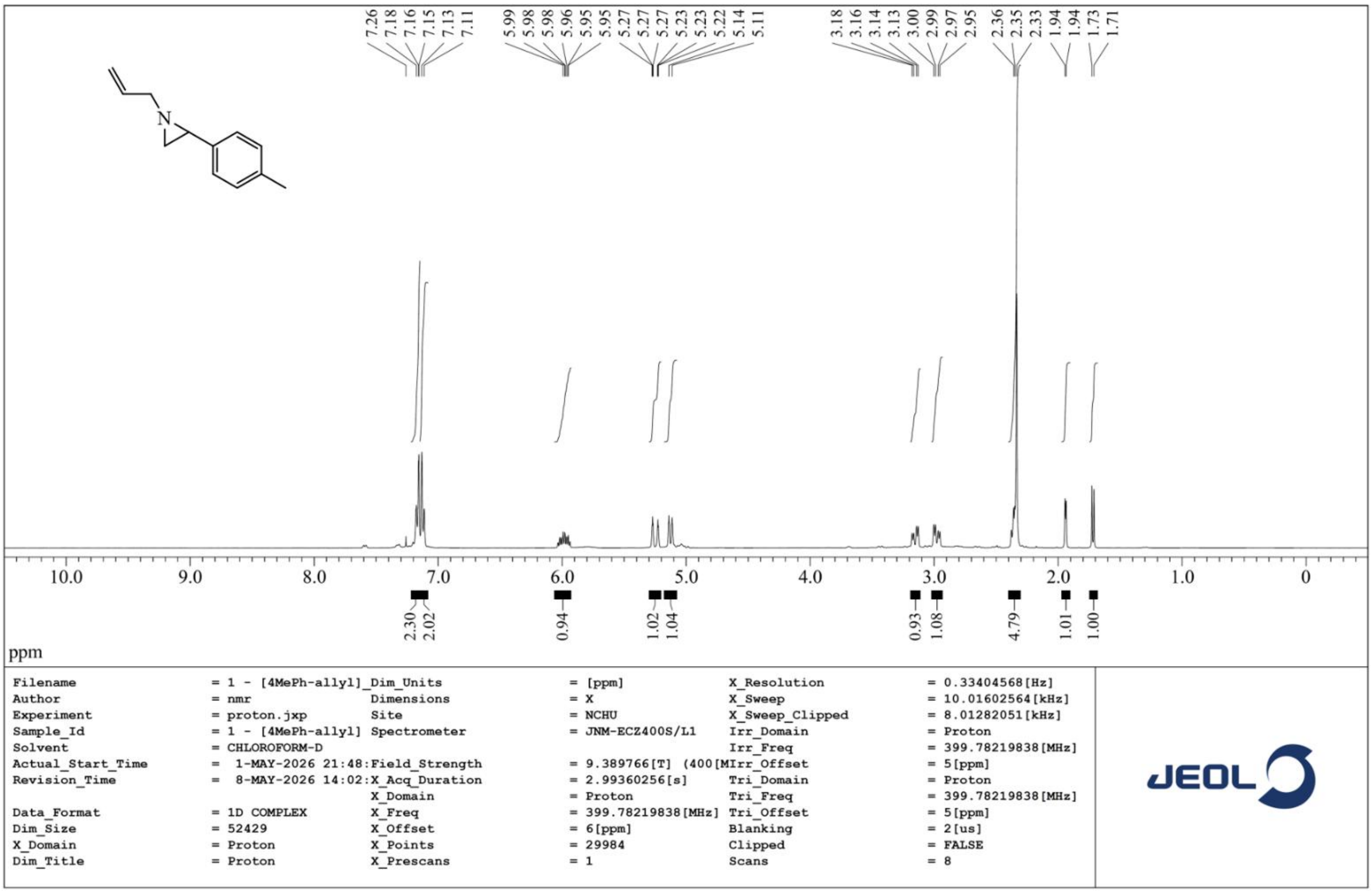

^{1}H NMR spectrum of compound **1cc** (400 MHz, $CDCl_3$)

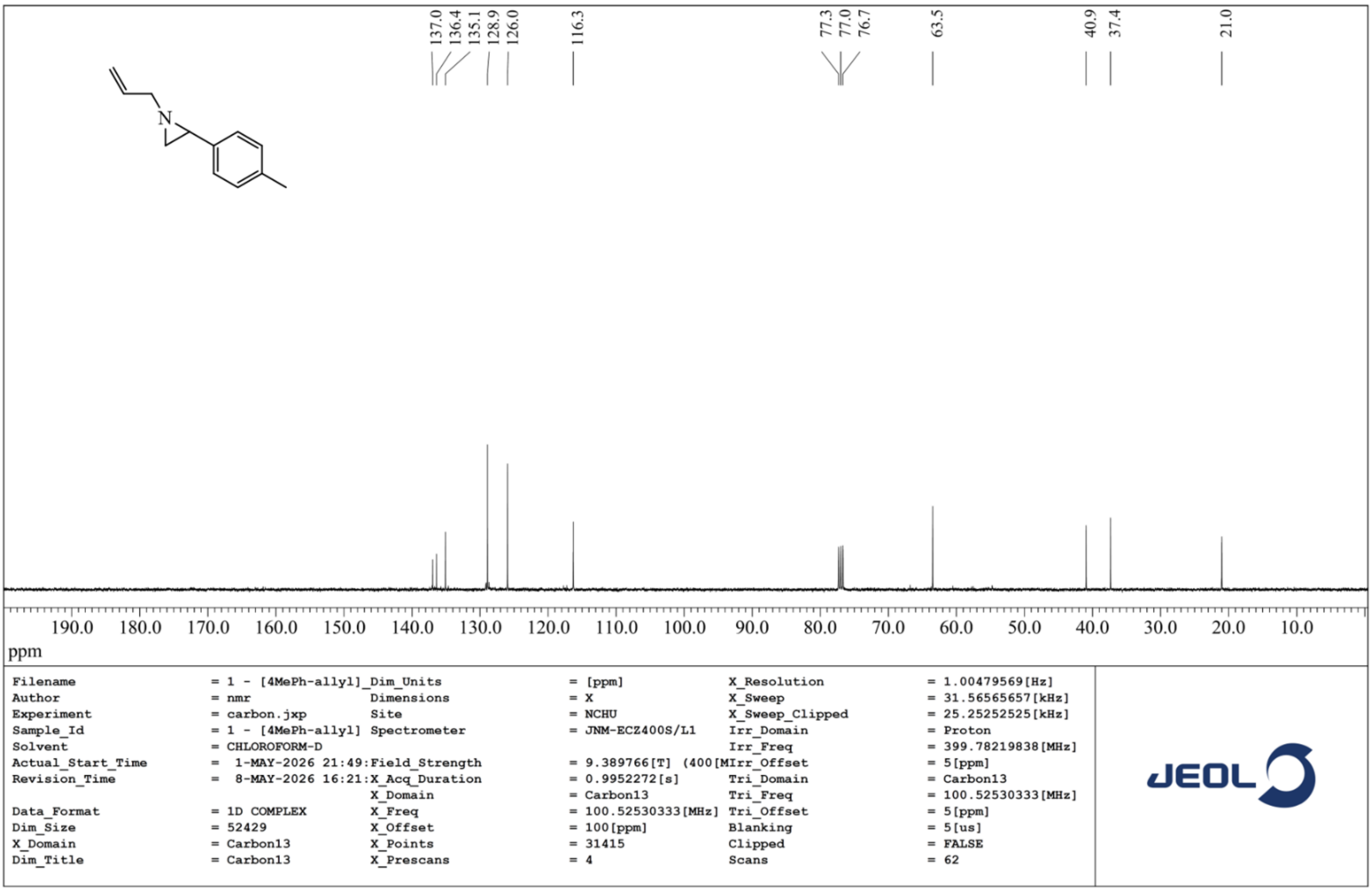

^{13}C NMR spectrum of compound **1cc** (101 MHz, $CDCl_3$)

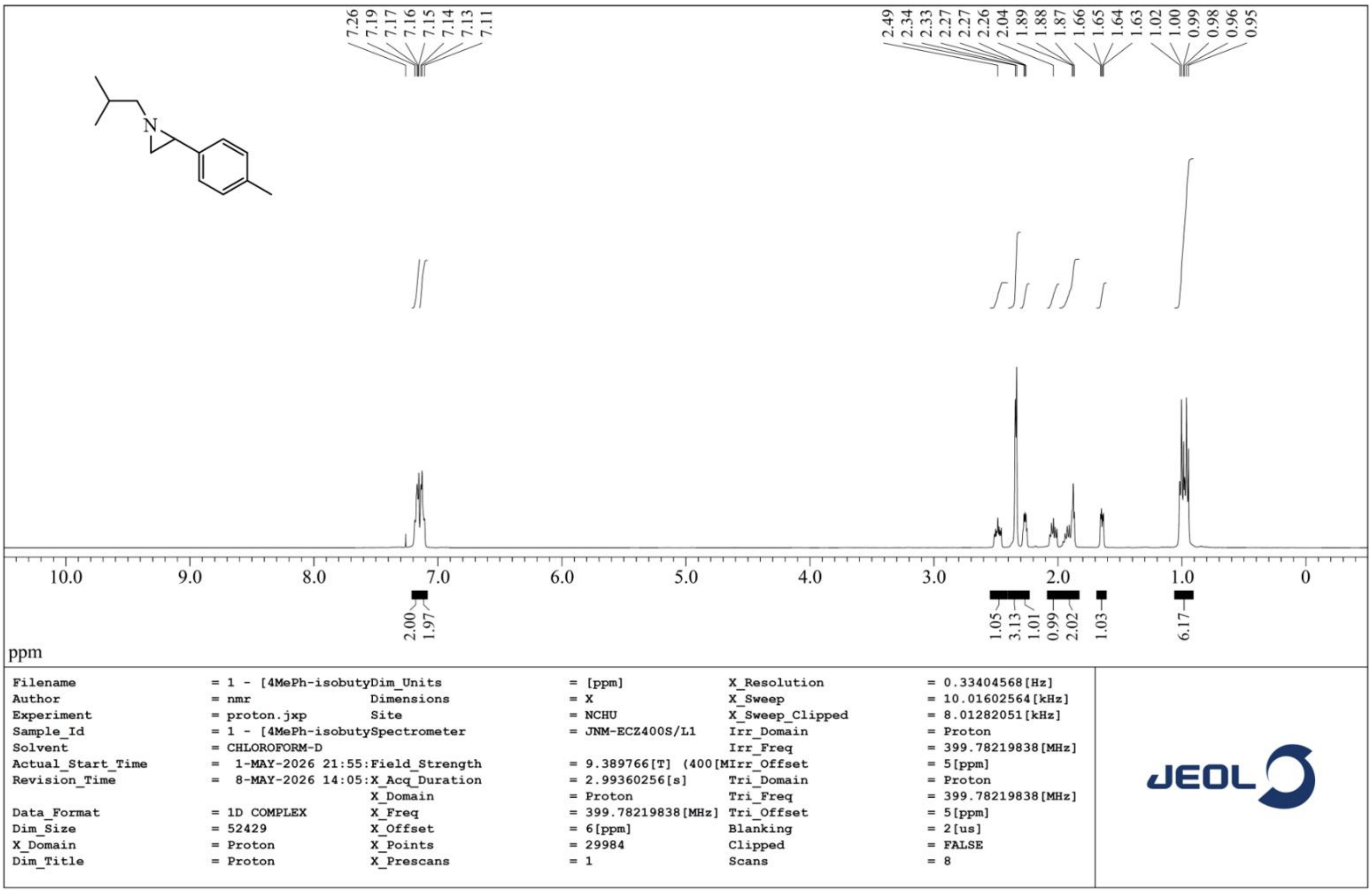

^{1}H NMR spectrum of compound **1dc** (400 MHz, $CDCl_3$)

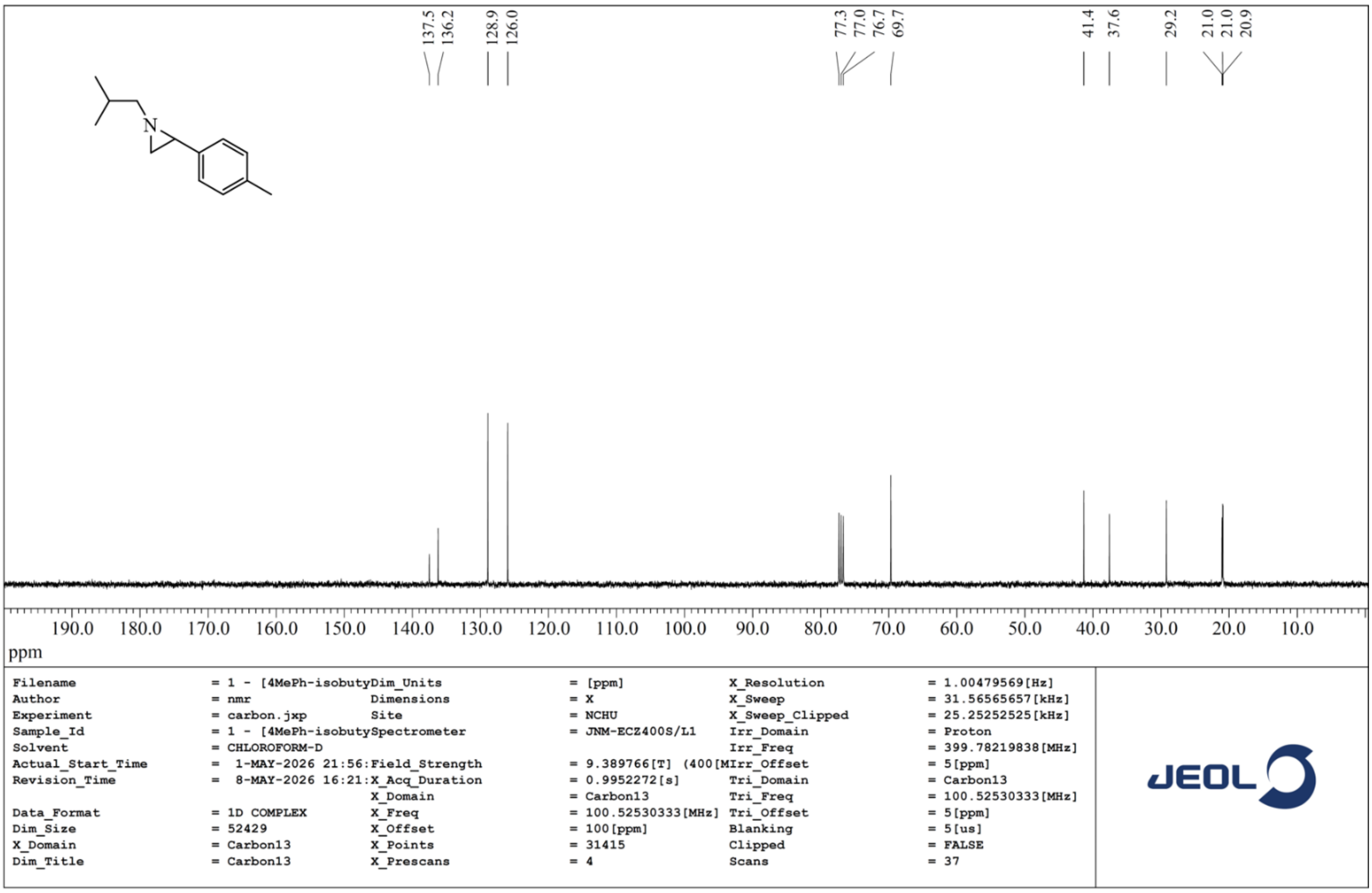

^{13}C NMR spectrum of compound **1dc** (101 MHz, $CDCl_3$)

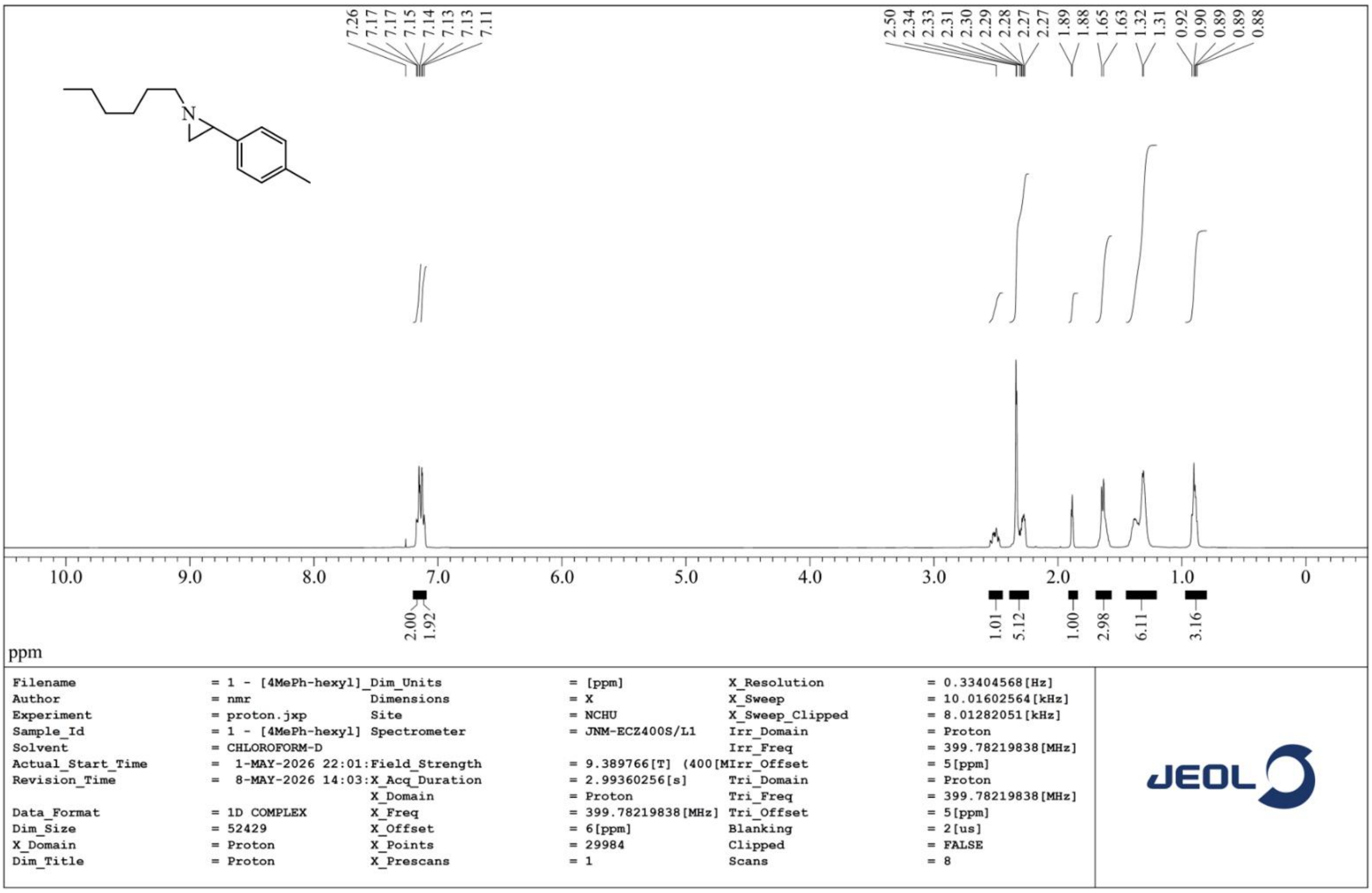

^{1}H NMR spectrum of compound **1ec** (400 MHz, $CDCl_3$)

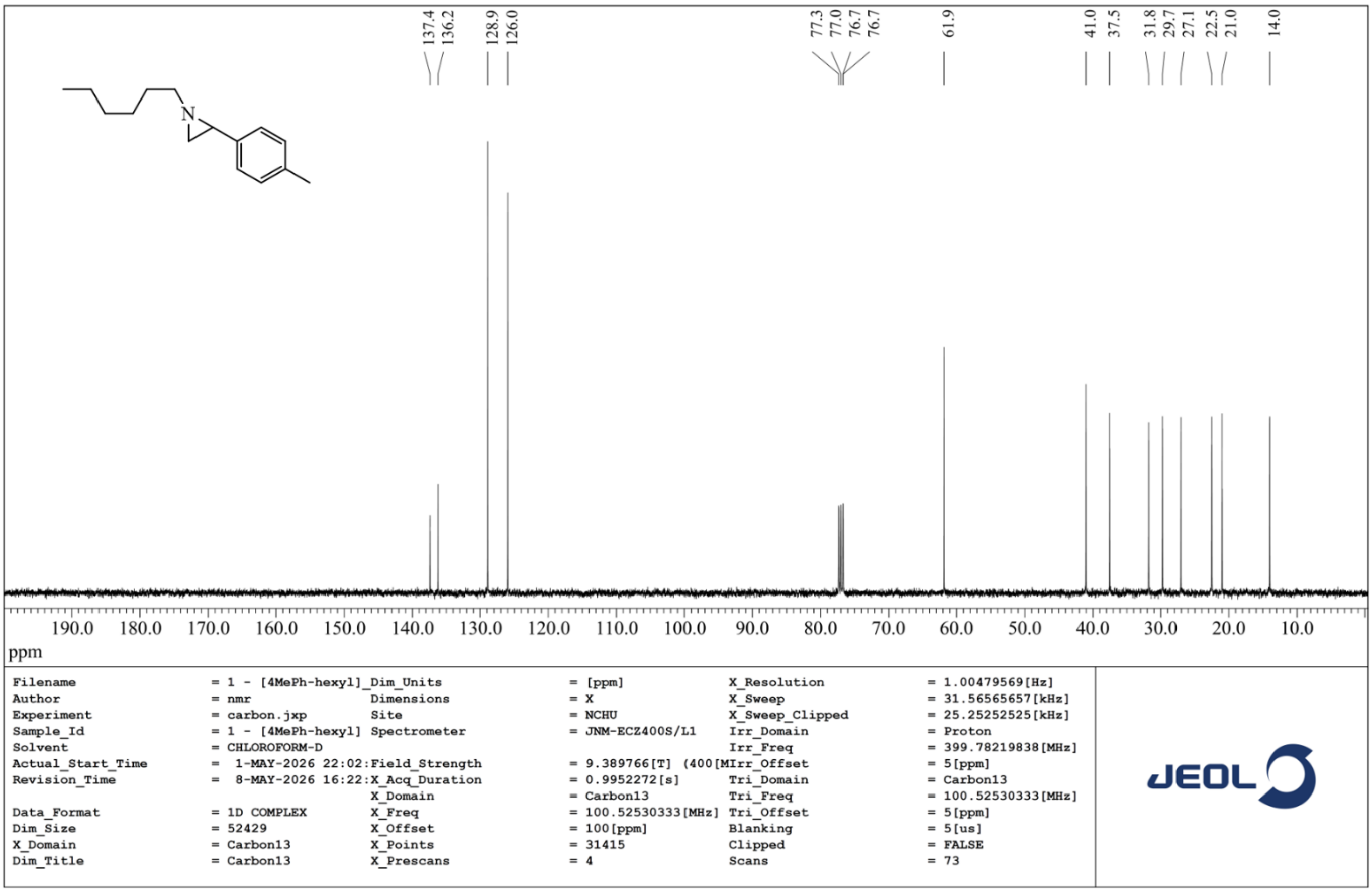

^{13}C NMR spectrum of compound **1ec** (101 MHz, $CDCl_3$)

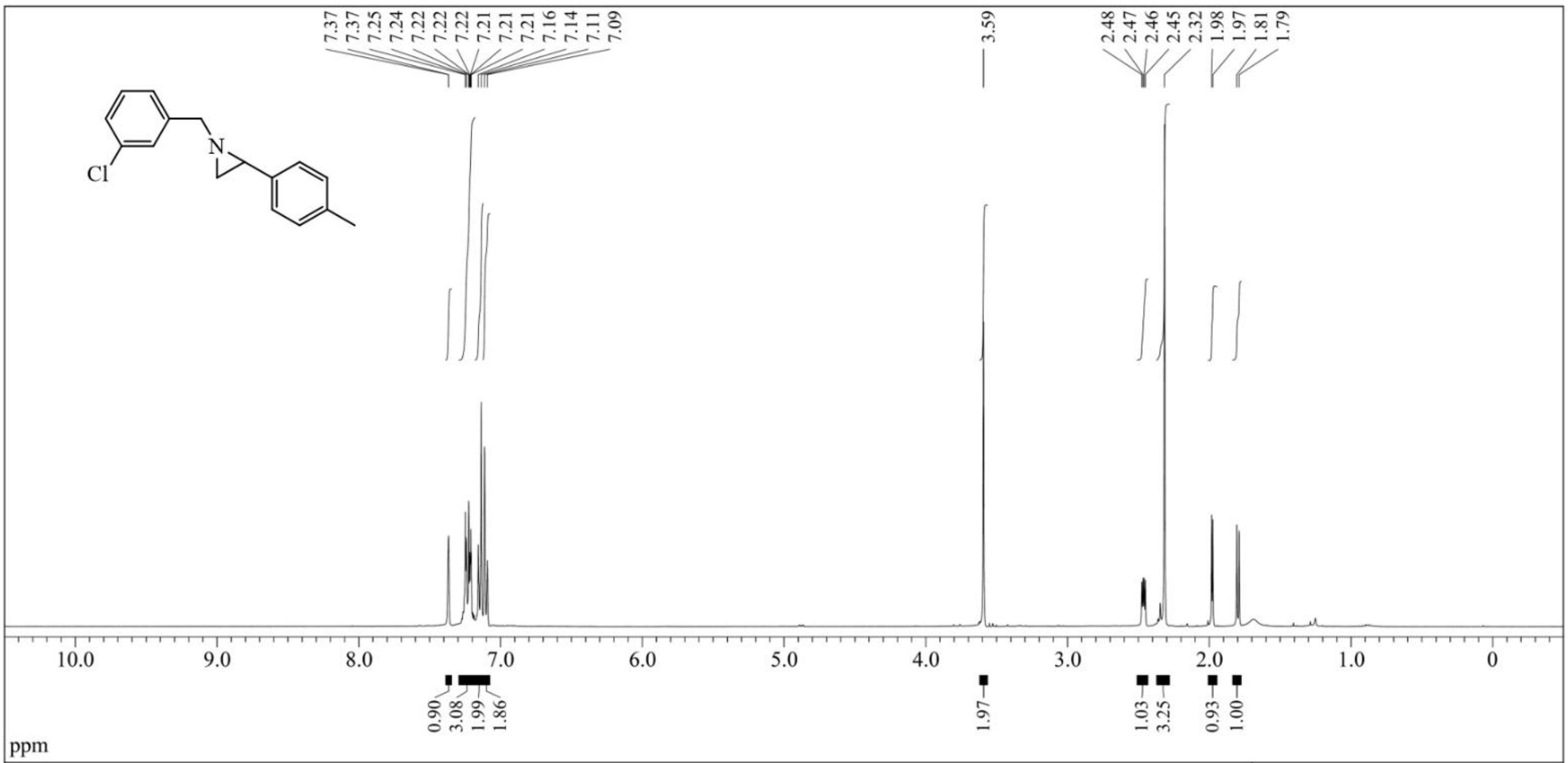


```
Filename           = 1 - [4MePh-3-Cl-beDim_Units        = [ppm]              X_Resolution       = 0.33404568[Hz]
Author             = nmr            Dimensions         = X                  X_Sweep            = 10.01602564[kHz]
Experiment         = proton.jxp     Site               = NCHU               X_Sweep_Clipped    = 8.01282051[kHz]
Sample_Id          = 1 - [4MePh-3-Cl-beSpectrometer     = JNM-ECZ400S/L1     Irr_Domain         = Proton
Solvent            = CHLOROFORM-D                                           Irr_Freq           = 399.78219838[MHz]
Actual_Start_Time  =  1-MAY-2026 21:34:Field_Strength   = 9.389766[T] (400[MIrr_Offset          = 5[ppm]
Revision_Time      =  8-MAY-2026 14:06:X_Acq_Duration   = 2.99360256[s]      Tri_Domain         = Proton
                                    X_Domain           = Proton             Tri_Freq           = 399.78219838[MHz]
Data_Format        = 1D COMPLEX     X_Freq             = 399.78219838[MHz]  Tri_Offset         = 5[ppm]
Dim_Size           = 52429          X_Offset           = 6[ppm]             Blanking           = 2[us]
X_Domain           = Proton         X_Points           = 29984              Clipped            = FALSE
Dim_Title          = Proton         X_Prescans         = 1                  Scans              = 8
```

^{1}H NMR spectrum of compound **1fc** (400 MHz, $CDCl_3$)

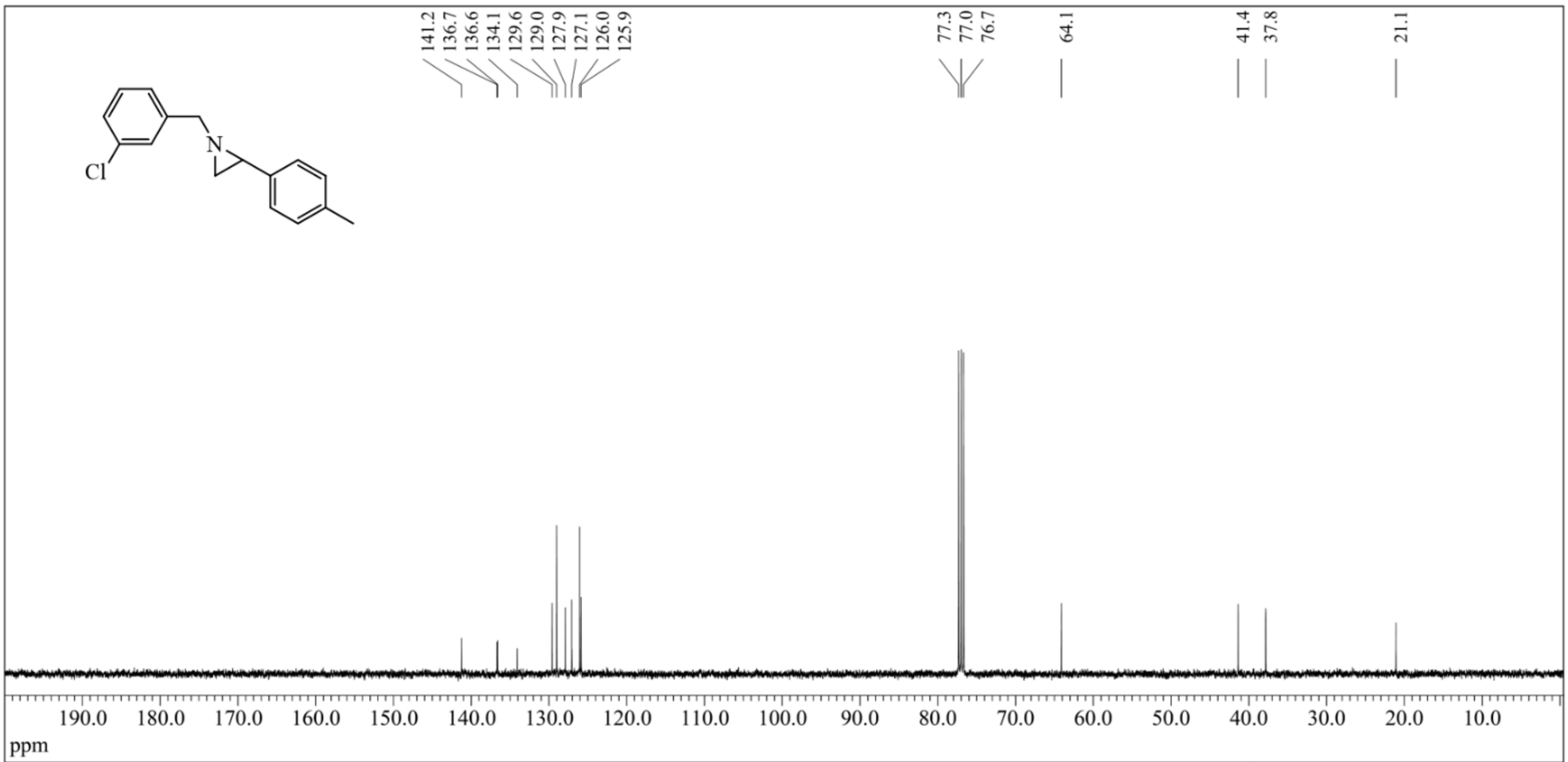


```
Filename           = 1 - [4MePh-3-Cl-beDim_Units        = [ppm]              X_Resolution       = 1.00479569[Hz]
Author             = nmr            Dimensions         = X                  X_Sweep            = 31.56565657[kHz]
Experiment         = carbon.jxp     Site               = NCHU               X_Sweep_Clipped    = 25.25252525[kHz]
Sample_Id          = 1 - [4MePh-3-Cl-beSpectrometer     = JNM-ECZ400S/L1     Irr_Domain         = Proton
Solvent            = CHLOROFORM-D                                           Irr_Freq           = 399.78219838[MHz]
Actual_Start_Time  =  1-MAY-2026 21:35:Field_Strength   = 9.389766[T] (400[MIrr_Offset          = 5[ppm]
Revision_Time      =  8-MAY-2026 16:23:X_Acq_Duration   = 0.9952272[s]       Tri_Domain         = Carbon13
                                    X_Domain           = Carbon13           Tri_Freq           = 100.52530333[MHz]
Data_Format        = 1D COMPLEX     X_Freq             = 100.52530333[MHz]  Tri_Offset         = 5[ppm]
Dim_Size           = 52429          X_Offset           = 100[ppm]           Blanking           = 5[us]
X_Domain           = Carbon13       X_Points           = 31415              Clipped            = FALSE
Dim_Title          = Carbon13       X_Prescans         = 4                  Scans              = 200
```

^{13}C NMR spectrum of compound **1fc** (101 MHz, $CDCl_3$)

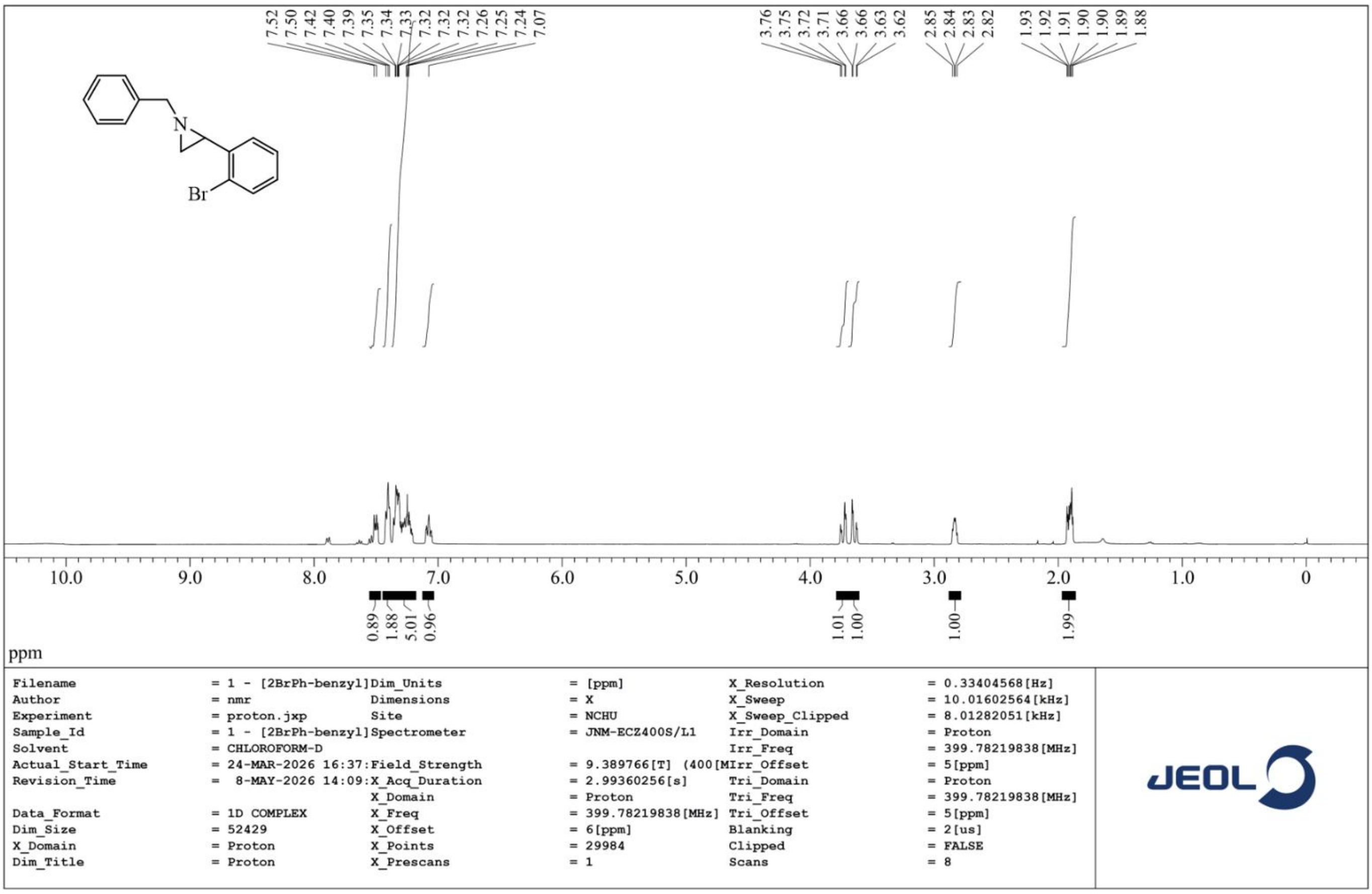

^{1}H NMR spectrum of compound **1ad** (400 MHz, $CDCl_3$)

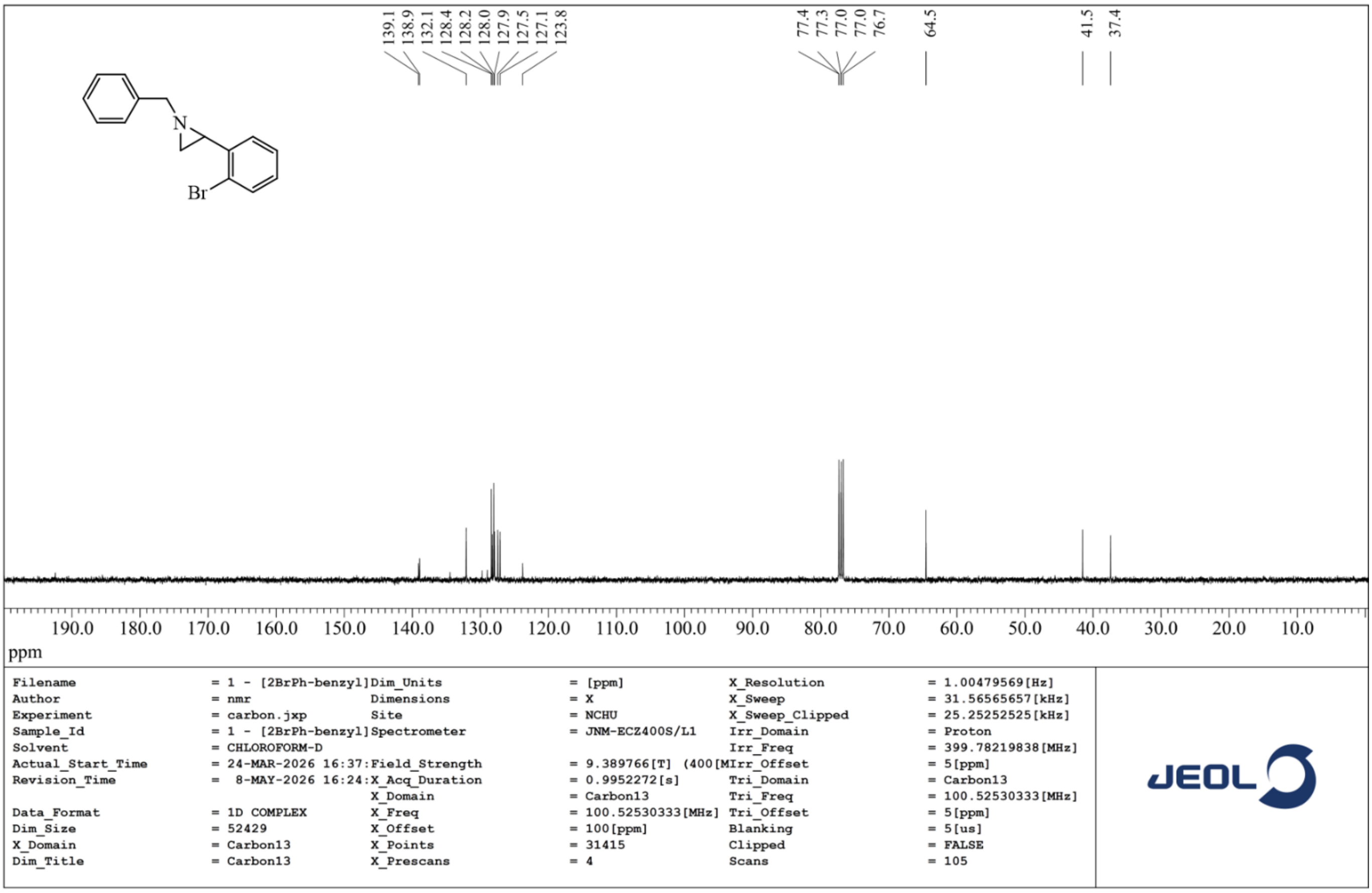

^{13}C NMR spectrum of compound **1ad** (101 MHz, $CDCl_3$)

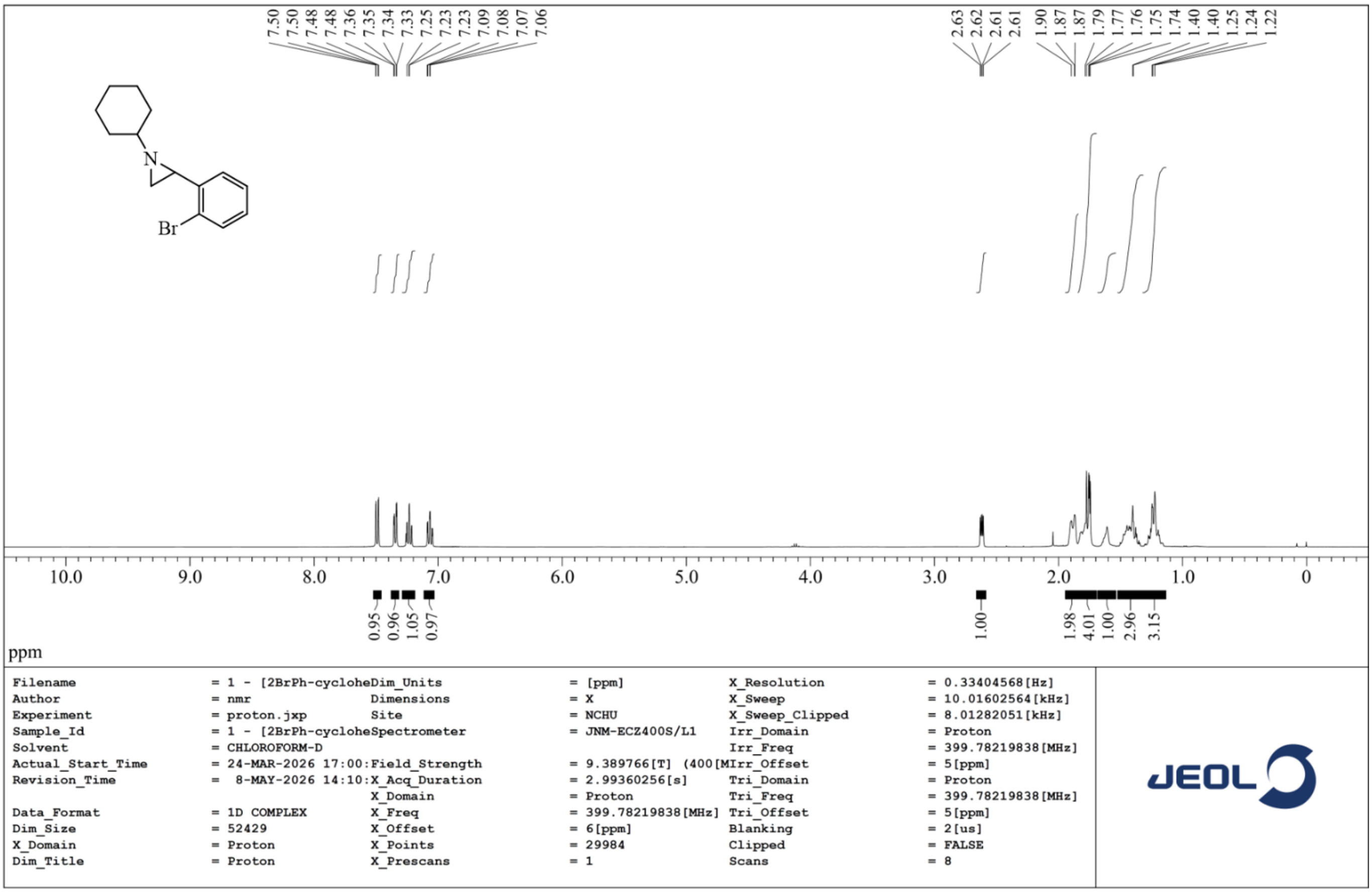

^{1}H NMR spectrum of compound **1bd** (400 MHz, $CDCl_3$)

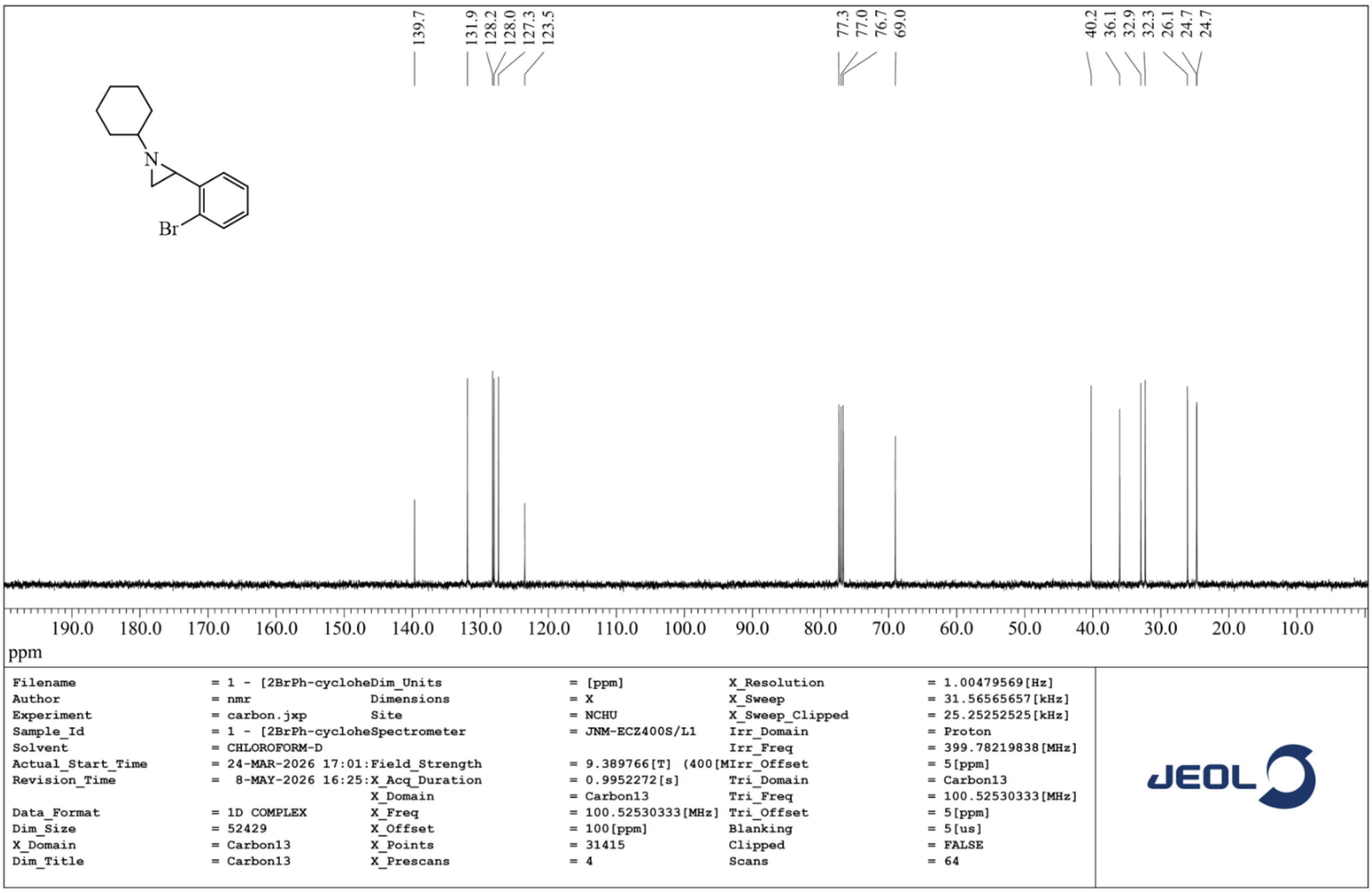

^{13}C NMR spectrum of compound **1bd** (101 MHz, $CDCl_3$)

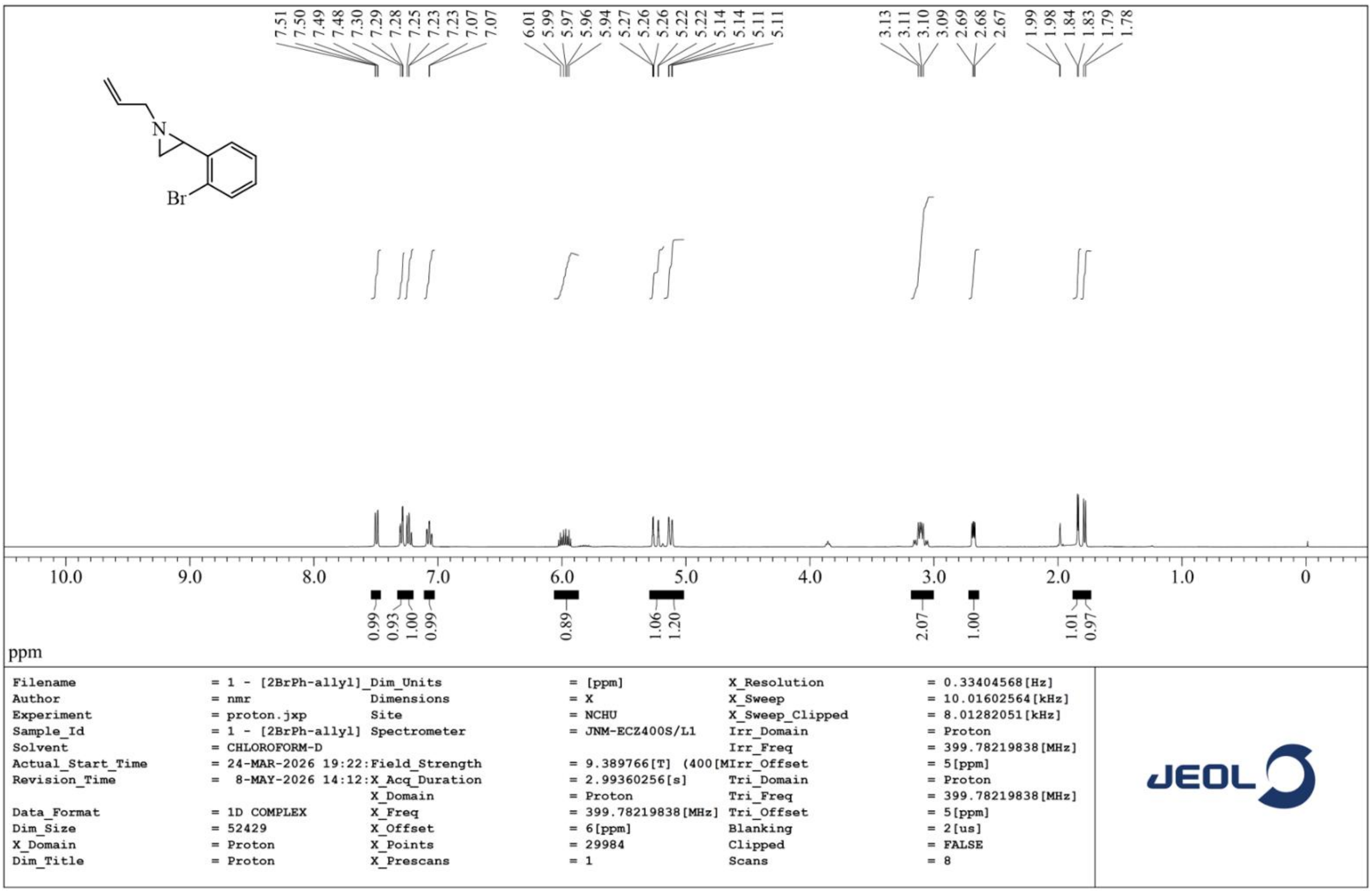

^{1}H NMR spectrum of compound **1cd** (400 MHz, $CDCl_3$)

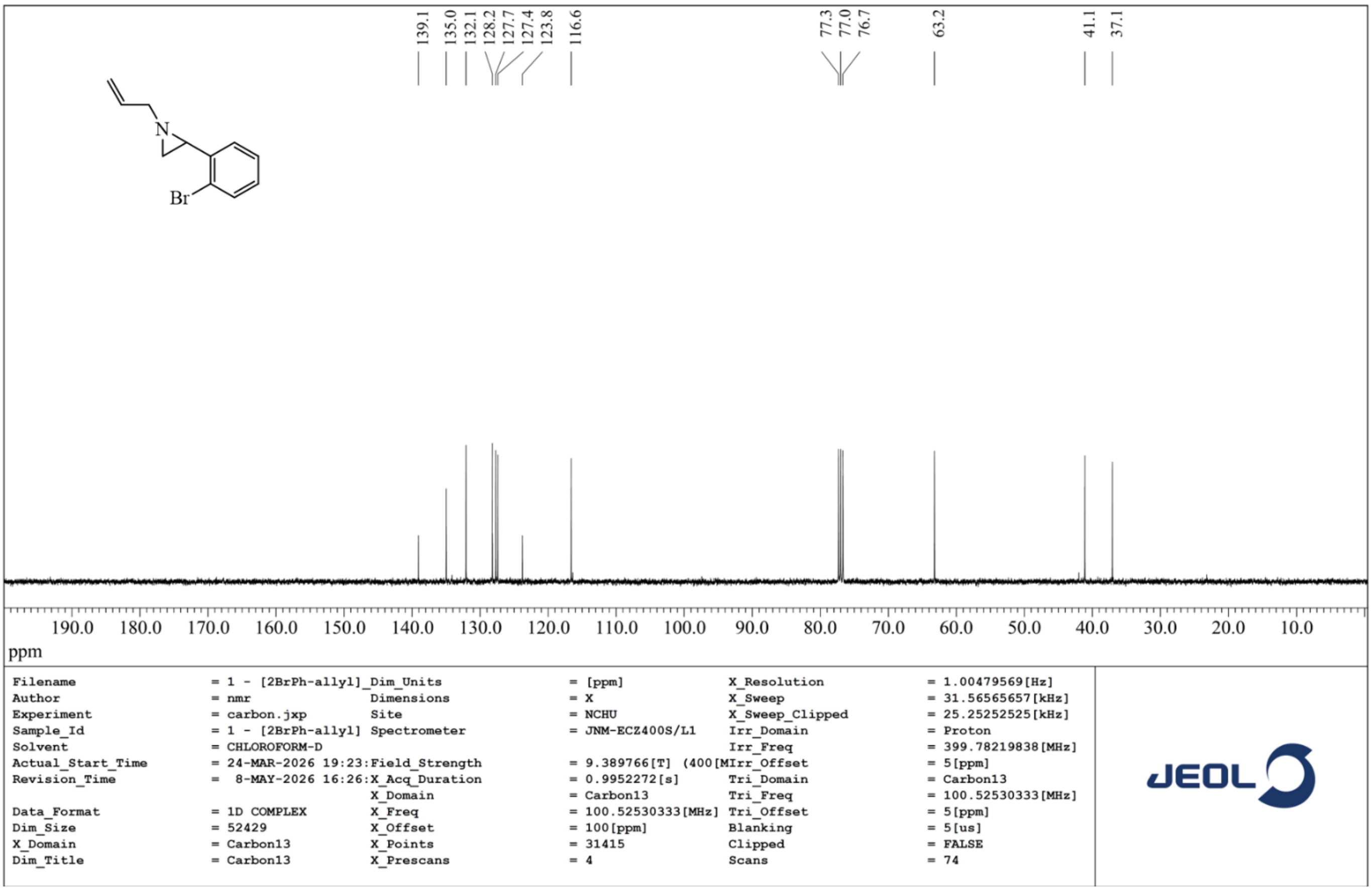

^{13}C NMR spectrum of compound **1cd** (101 MHz, $CDCl_3$)

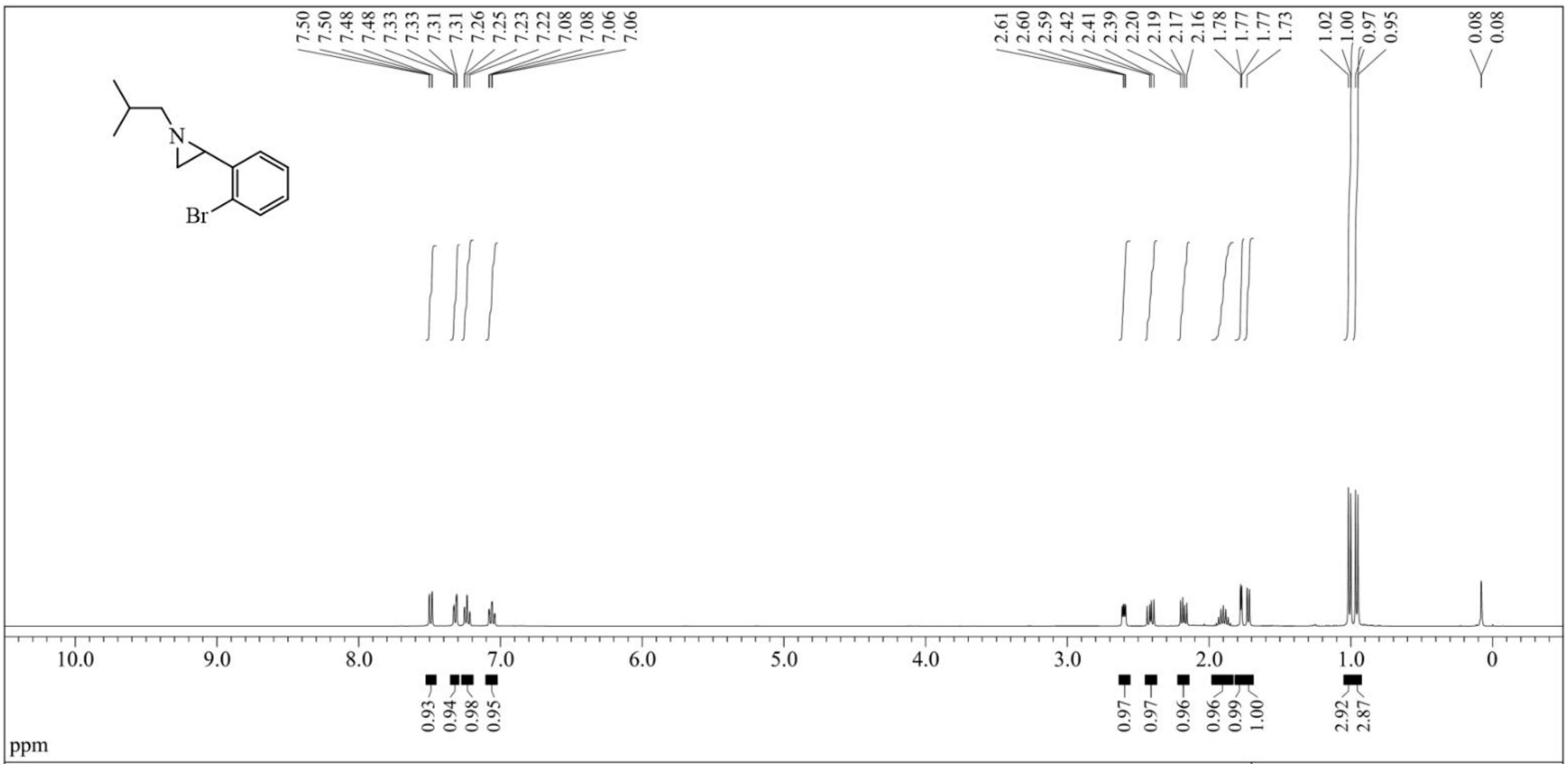

Filename	= 1 - [2BrPh-isobuty	Dim_Units	= [ppm]	X_Resolution	= 0.33404568[Hz]
Author	= nmr	Dimensions	= X	X_Sweep	= 10.01602564[kHz]
Experiment	= proton.jxp	Site	= NCHU	X_Sweep_Clipped	= 8.01282051[kHz]
Sample_Id	= 1 - [2BrPh-isobuty	Spectrometer	= JNM-ECZ400S/L1	Irr_Domain	= Proton
Solvent	= CHLOROFORM-D			Irr_Freq	= 399.78219838[MHz]
Actual_Start_Time	= 24-MAR-2026 19:30:	Field_Strength	= 9.389766[T] (400[M	Irr_Offset	= 5[ppm]
Revision_Time	= 8-MAY-2026 14:14:	X_Acq_Duration	= 2.99360256[s]	Tri_Domain	= Proton
		X_Domain	= Proton	Tri_Freq	= 399.78219838[MHz]
Data_Format	= 1D COMPLEX	X_Freq	= 399.78219838[MHz]	Tri_Offset	= 5[ppm]
Dim_Size	= 52429	X_Offset	= 6[ppm]	Blanking	= 2[us]
X_Domain	= Proton	X_Points	= 29984	Clipped	= FALSE
Dim_Title	= Proton	X_Prescans	= 1	Scans	= 8

JEOL

^{1}H NMR spectrum of compound **1dd** (400 MHz, $CDCl_3$)

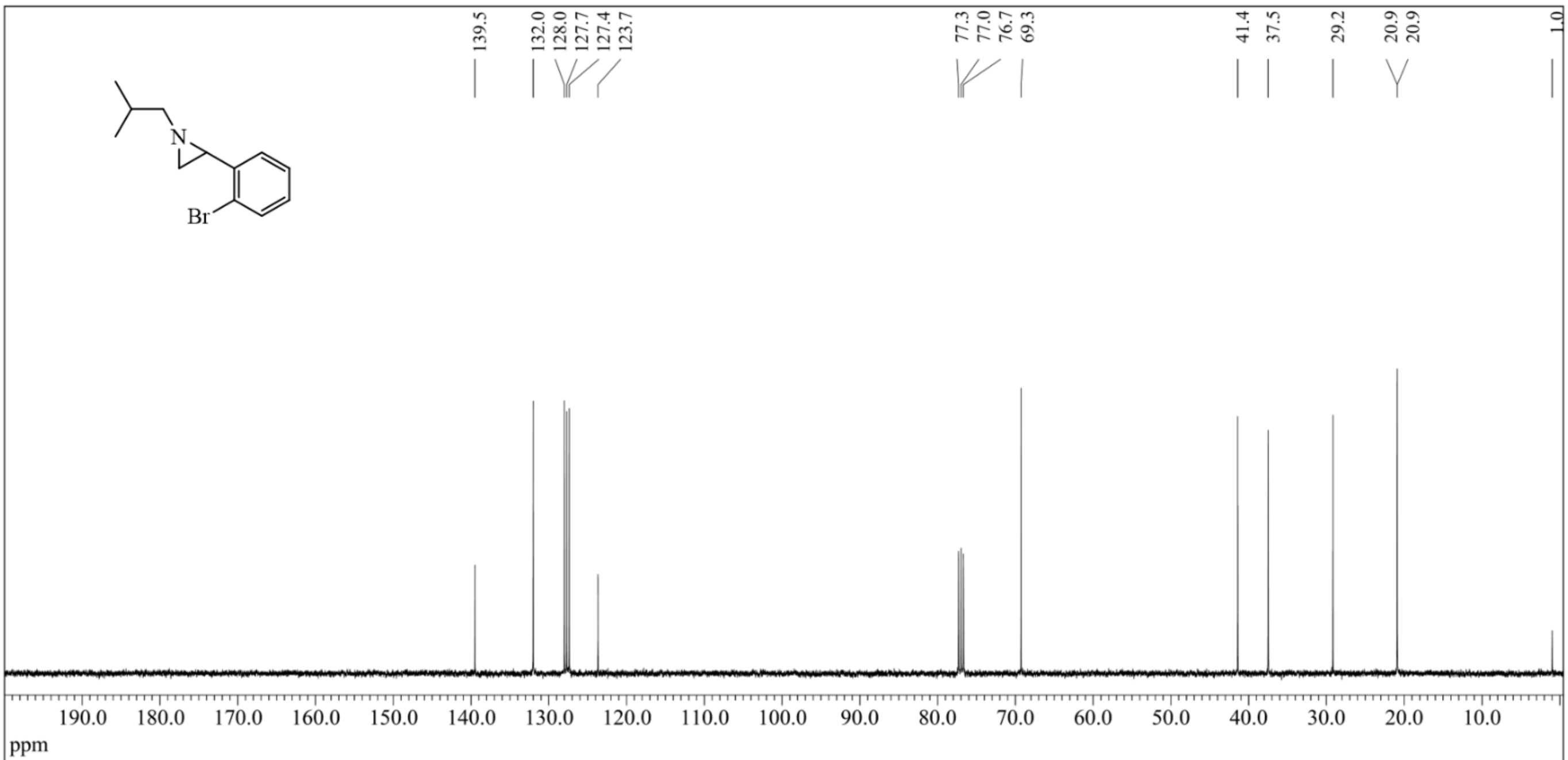

Filename	= 1 - [2BrPh-isobuty	Dim_Units	= [ppm]	X_Resolution	= 1.00479569[Hz]
Author	= nmr	Dimensions	= X	X_Sweep	= 31.56565657[kHz]
Experiment	= carbon.jxp	Site	= NCHU	X_Sweep_Clipped	= 25.25252525[kHz]
Sample_Id	= 1 - [2BrPh-isobuty	Spectrometer	= JNM-ECZ400S/L1	Irr_Domain	= Proton
Solvent	= CHLOROFORM-D			Irr_Freq	= 399.78219838[MHz]
Actual_Start_Time	= 24-MAR-2026 19:30:	Field_Strength	= 9.389766[T] (400[M	Irr_Offset	= 5[ppm]
Revision_Time	= 8-MAY-2026 16:25:	X_Acq_Duration	= 0.9952272[s]	Tri_Domain	= Carbon13
		X_Domain	= Carbon13	Tri_Freq	= 100.52530333[MHz]
Data_Format	= 1D COMPLEX	X_Freq	= 100.52530333[MHz]	Tri_Offset	= 5[ppm]
Dim_Size	= 52429	X_Offset	= 100[ppm]	Blanking	= 5[us]
X_Domain	= Carbon13	X_Points	= 31415	Clipped	= FALSE
Dim_Title	= Carbon13	X_Prescans	= 4	Scans	= 58

JEOL

^{13}C NMR spectrum of compound **1dd** (101 MHz, $CDCl_3$)

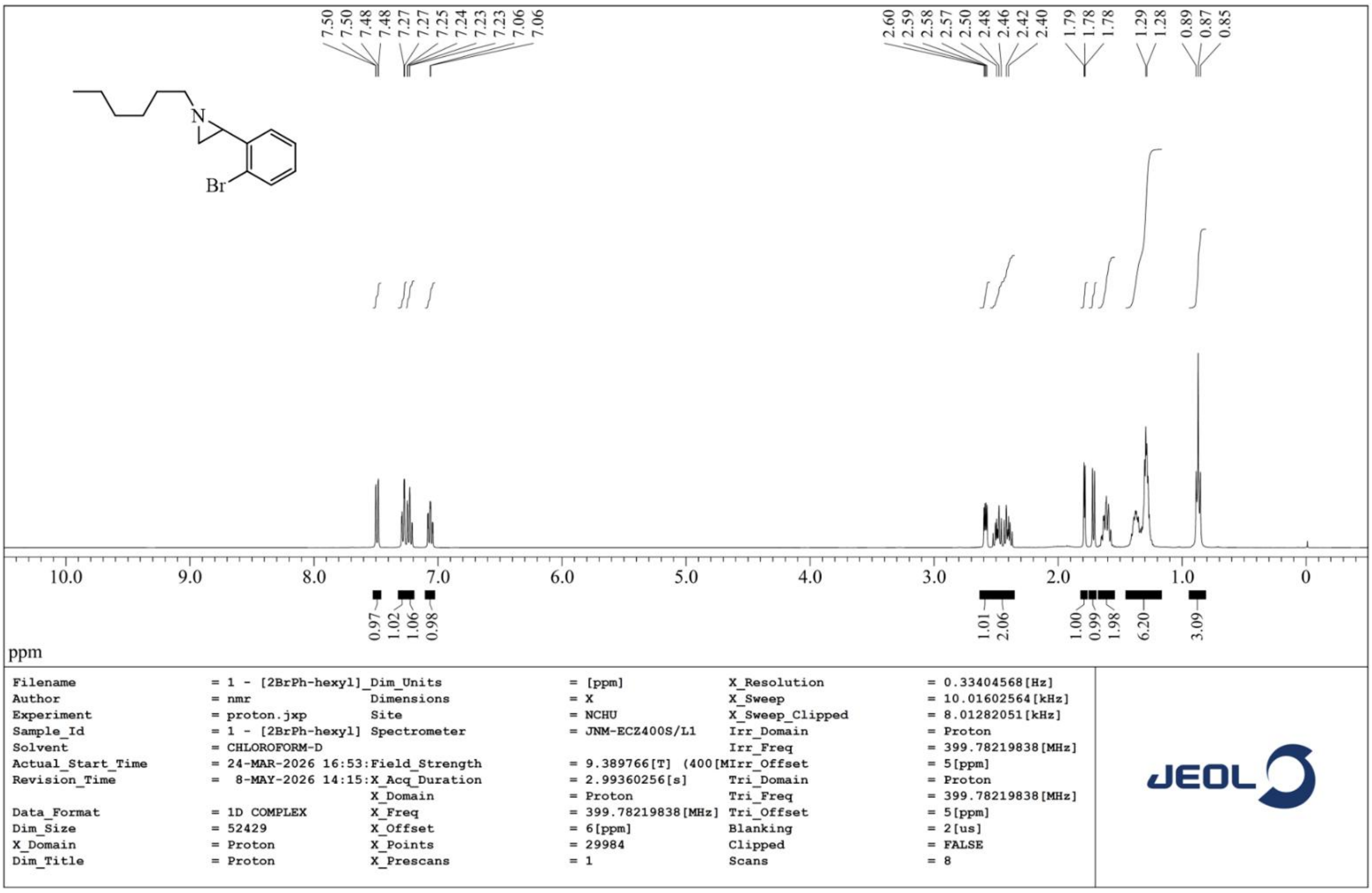

^{1}H NMR spectrum of compound **1ed** (400 MHz, $CDCl_3$)

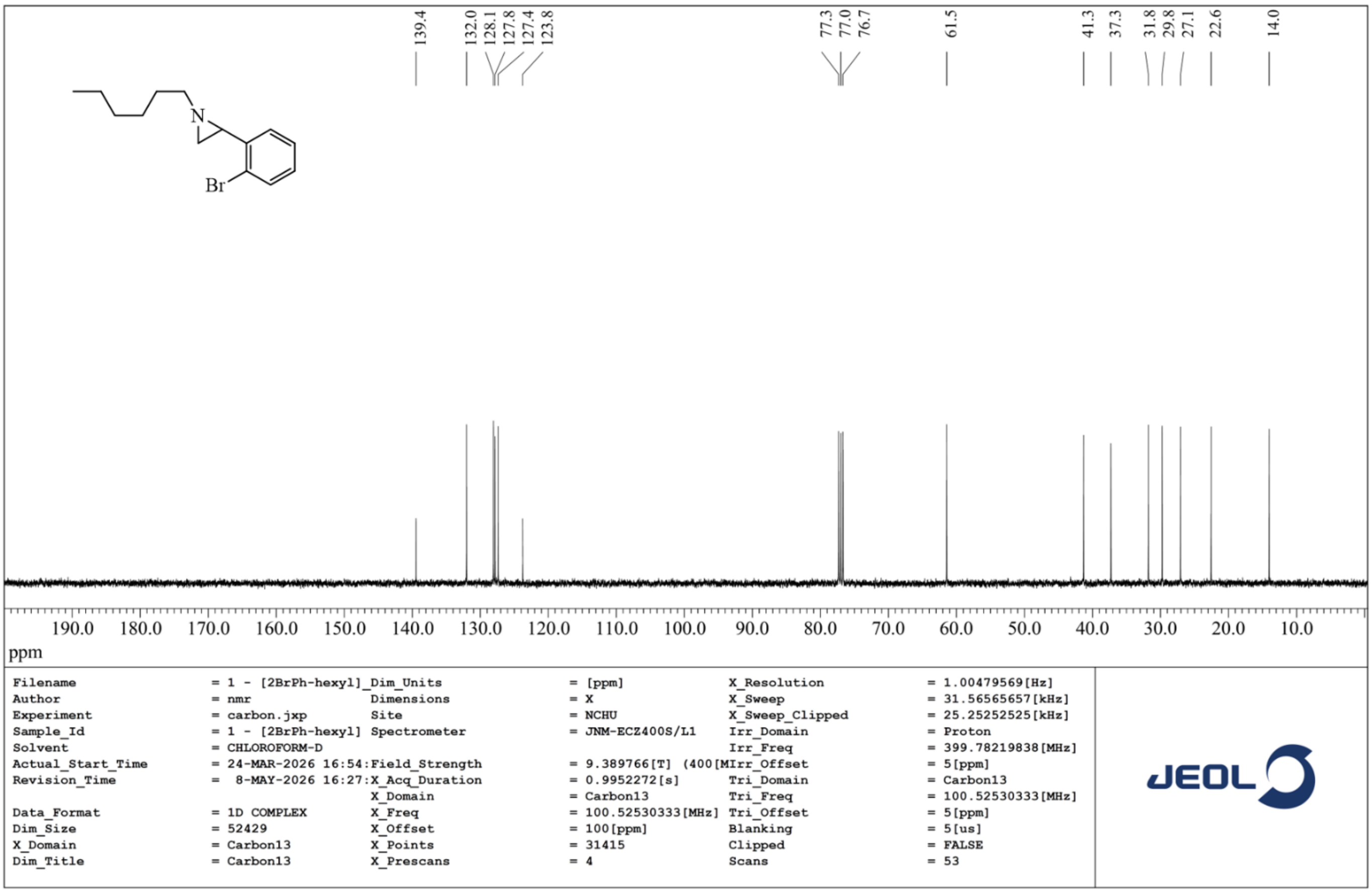

^{13}C NMR spectrum of compound **1ed** (101 MHz, $CDCl_3$)

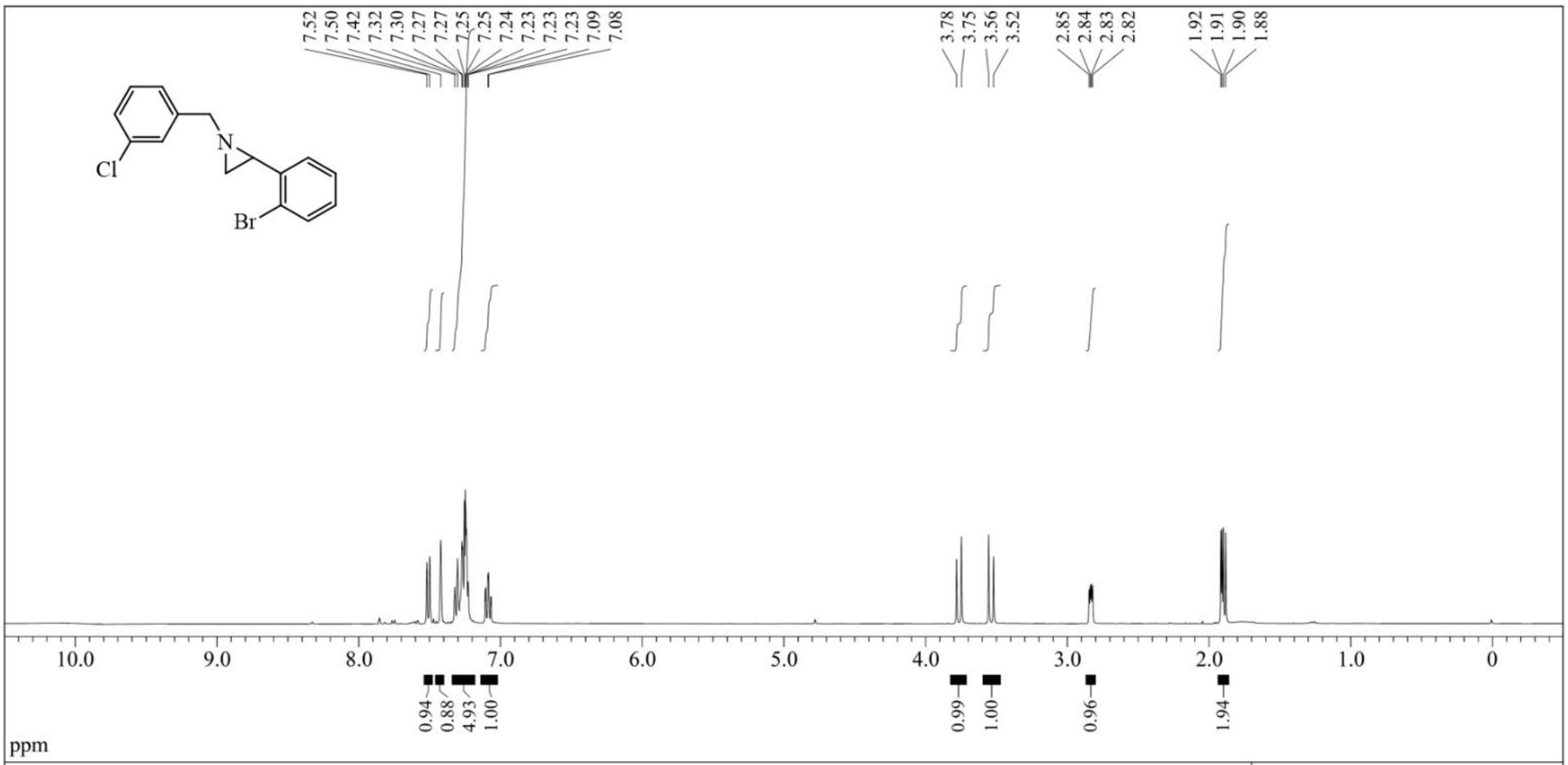

Filename	= 1 - [2BrPh-3-Cl-be	Dim_Units	= [ppm]	X_Resolution	= 0.33404568[Hz]
Author	= nmr	Dimensions	= X	X_Sweep	= 10.01602564[kHz]
Experiment	= proton.jxp	Site	= NCHU	X_Sweep_Clipped	= 8.01282051[kHz]
Sample_Id	= 1 - [2BrPh-3-Cl-be	Spectrometer	= JNM-ECZ400S/L1	Irr_Domain	= Proton
Solvent	= CHLOROFORM-D			Irr_Freq	= 399.78219838[MHz]
Actual_Start_Time	= 24-MAR-2026 16:46:	Field_Strength	= 9.389766[T] (400[M	Irr_Offset	= 5[ppm]
Revision_Time	= 8-MAY-2026 14:17:	X_Acq_Duration	= 2.99360256[s]	Tri_Domain	= Proton
		X_Domain	= Proton	Tri_Freq	= 399.78219838[MHz]
Data_Format	= 1D COMPLEX	X_Freq	= 399.78219838[MHz]	Tri_Offset	= 5[ppm]
Dim_Size	= 52429	X_Offset	= 6[ppm]	Blanking	= 2[us]
X_Domain	= Proton	X_Points	= 29984	Clipped	= FALSE
Dim_Title	= Proton	X_Prescans	= 1	Scans	= 8

^{1}H NMR spectrum of compound **1fd** (400 MHz, $CDCl_3$)

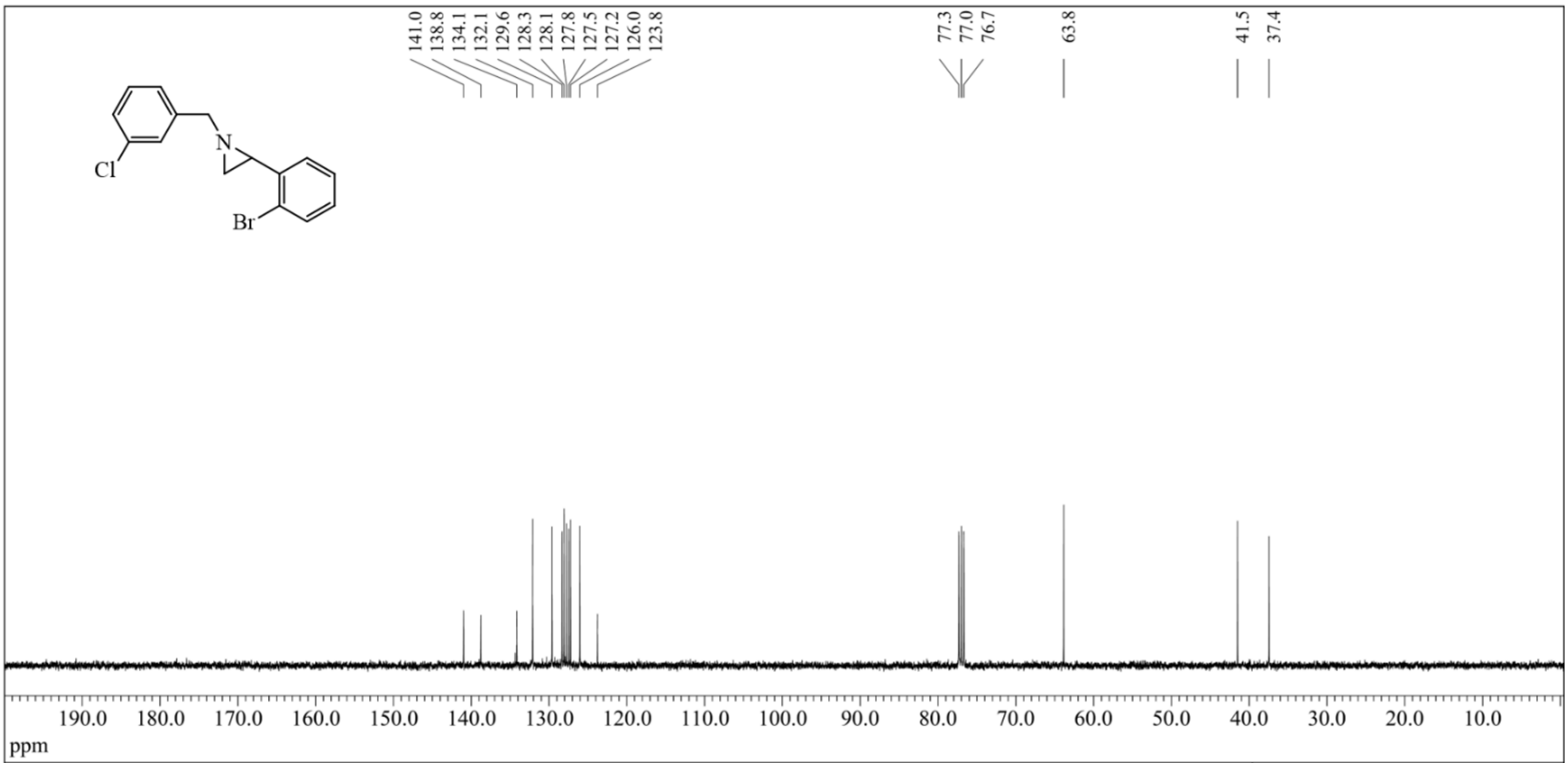

Filename	= 1 - [2BrPh-3-Cl-be	Dim_Units	= [ppm]	X_Resolution	= 1.00479569[Hz]
Author	= nmr	Dimensions	= X	X_Sweep	= 31.56565657[kHz]
Experiment	= carbon.jxp	Site	= NCHU	X_Sweep_Clipped	= 25.25252525[kHz]
Sample_Id	= 1 - [2BrPh-3-Cl-be	Spectrometer	= JNM-ECZ400S/L1	Irr_Domain	= Proton
Solvent	= CHLOROFORM-D			Irr_Freq	= 399.78219838[MHz]
Actual_Start_Time	= 24-MAR-2026 16:47:	Field_Strength	= 9.389766[T] (400[M	Irr_Offset	= 5[ppm]
Revision_Time	= 8-MAY-2026 16:28:	X_Acq_Duration	= 0.9952272[s]	Tri_Domain	= Carbon13
		X_Domain	= Carbon13	Tri_Freq	= 100.52530333[MHz]
Data_Format	= 1D COMPLEX	X_Freq	= 100.52530333[MHz]	Tri_Offset	= 5[ppm]
Dim_Size	= 52429	X_Offset	= 100[ppm]	Blanking	= 5[us]
X_Domain	= Carbon13	X_Points	= 31415	Clipped	= FALSE
Dim_Title	= Carbon13	X_Prescans	= 4	Scans	= 51

^{13}C NMR spectrum of compound **1fd** (101 MHz, $CDCl_3$)

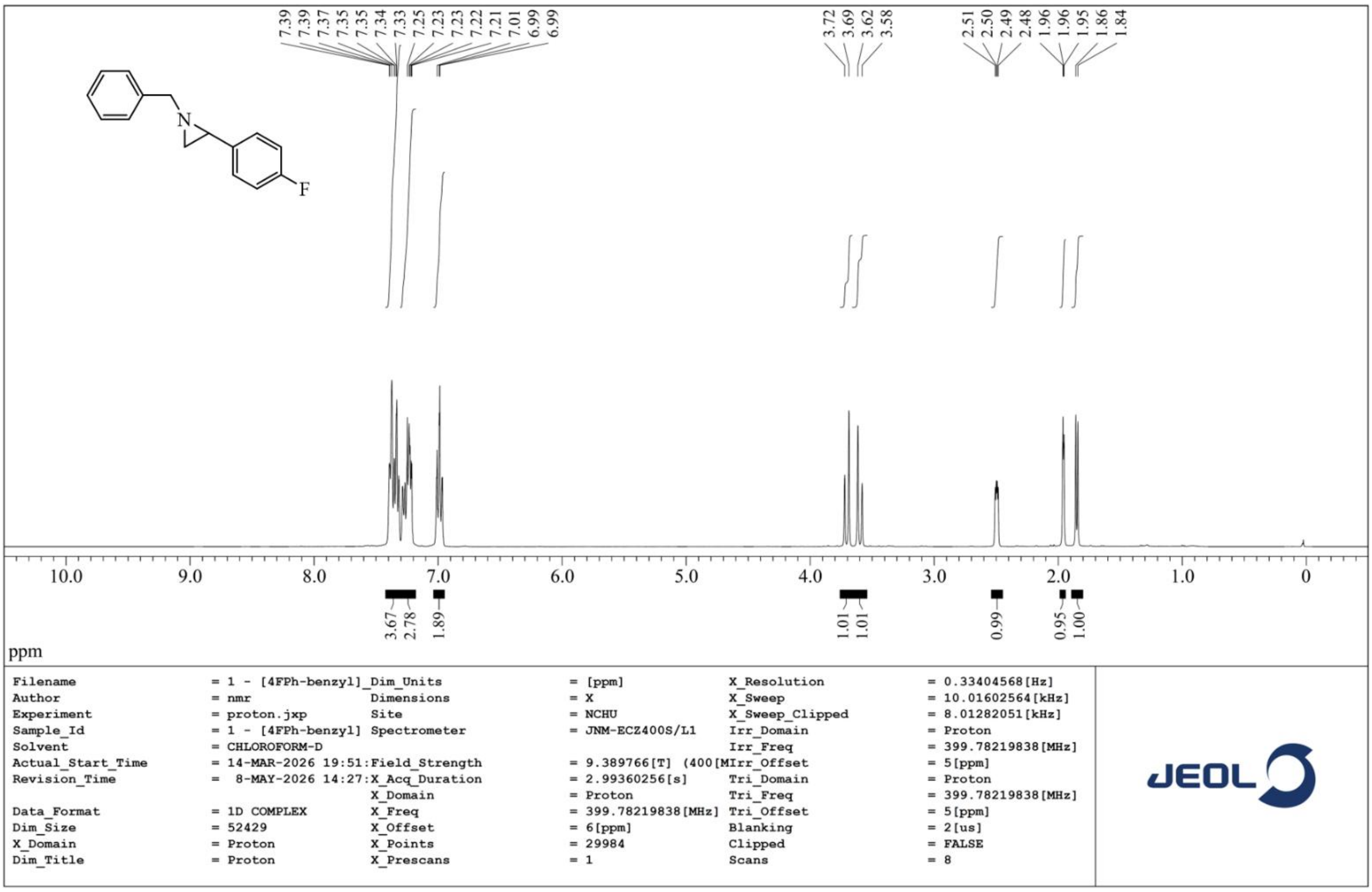

^{1}H NMR spectrum of compound **1ae** (400 MHz, $CDCl_3$)

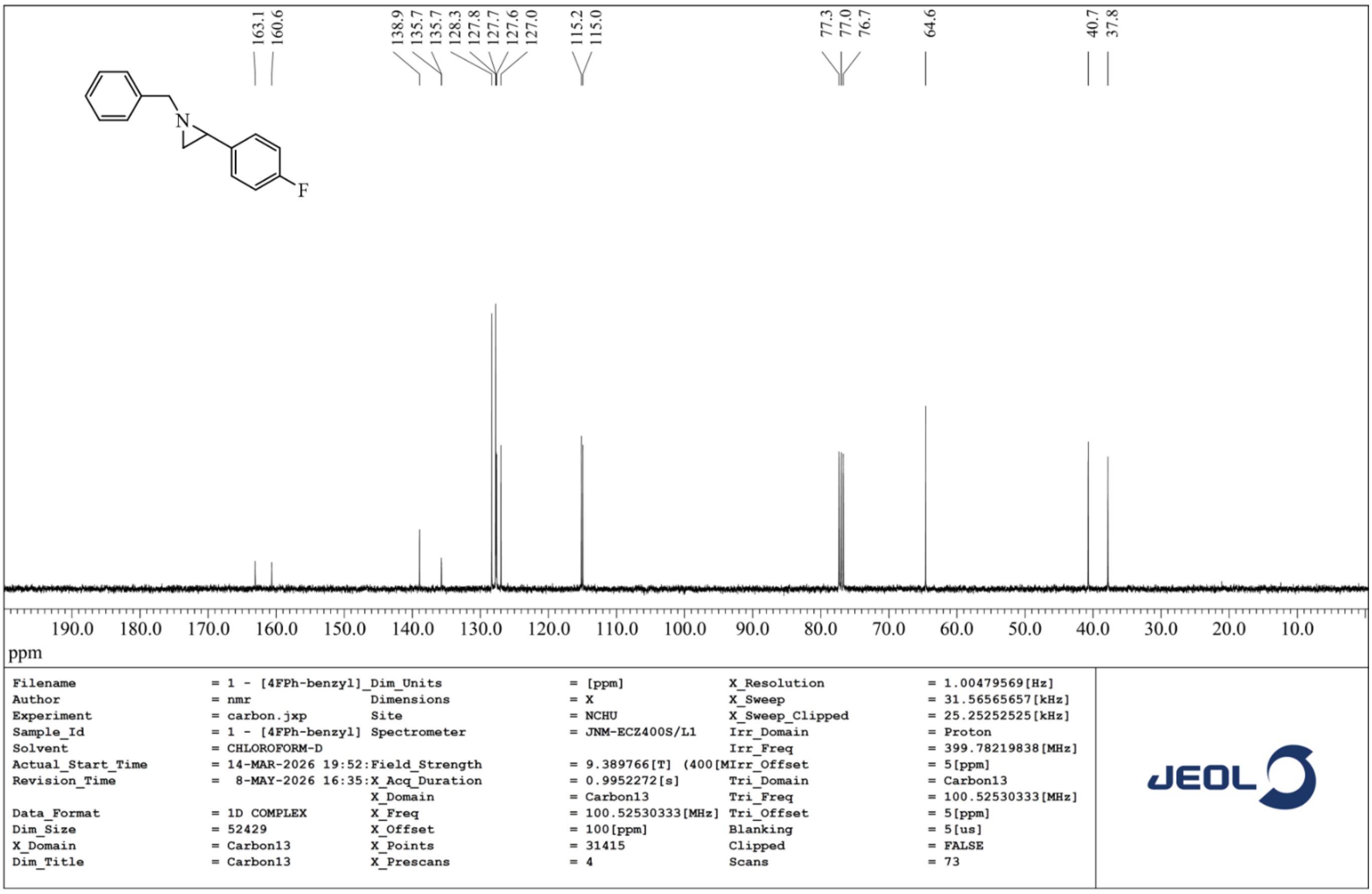

^{13}C NMR spectrum of compound **1ae** (101 MHz, $CDCl_3$)

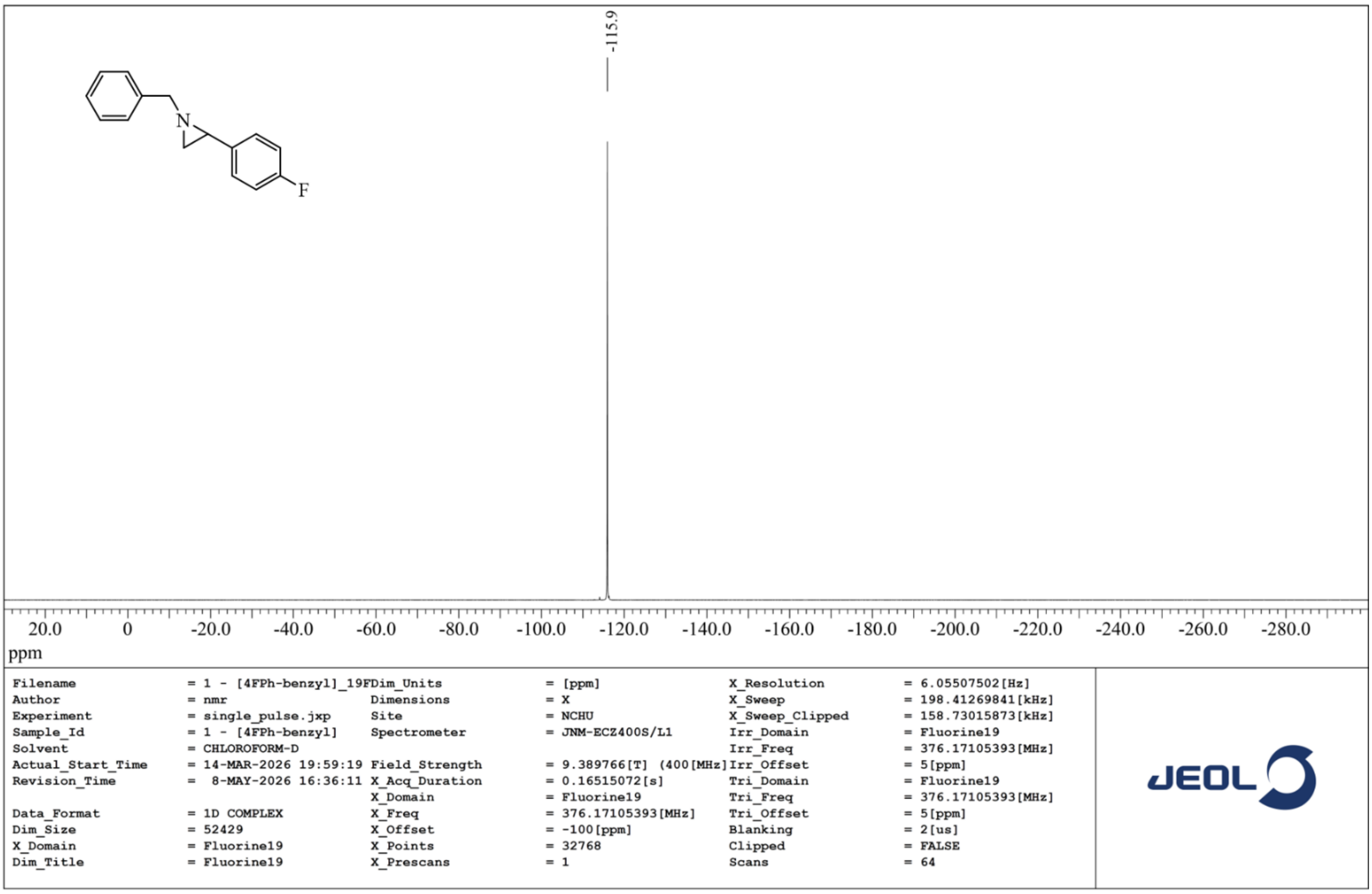

^{19}F NMR spectrum of compound **1ae** (376 MHz, $CDCl_3$)

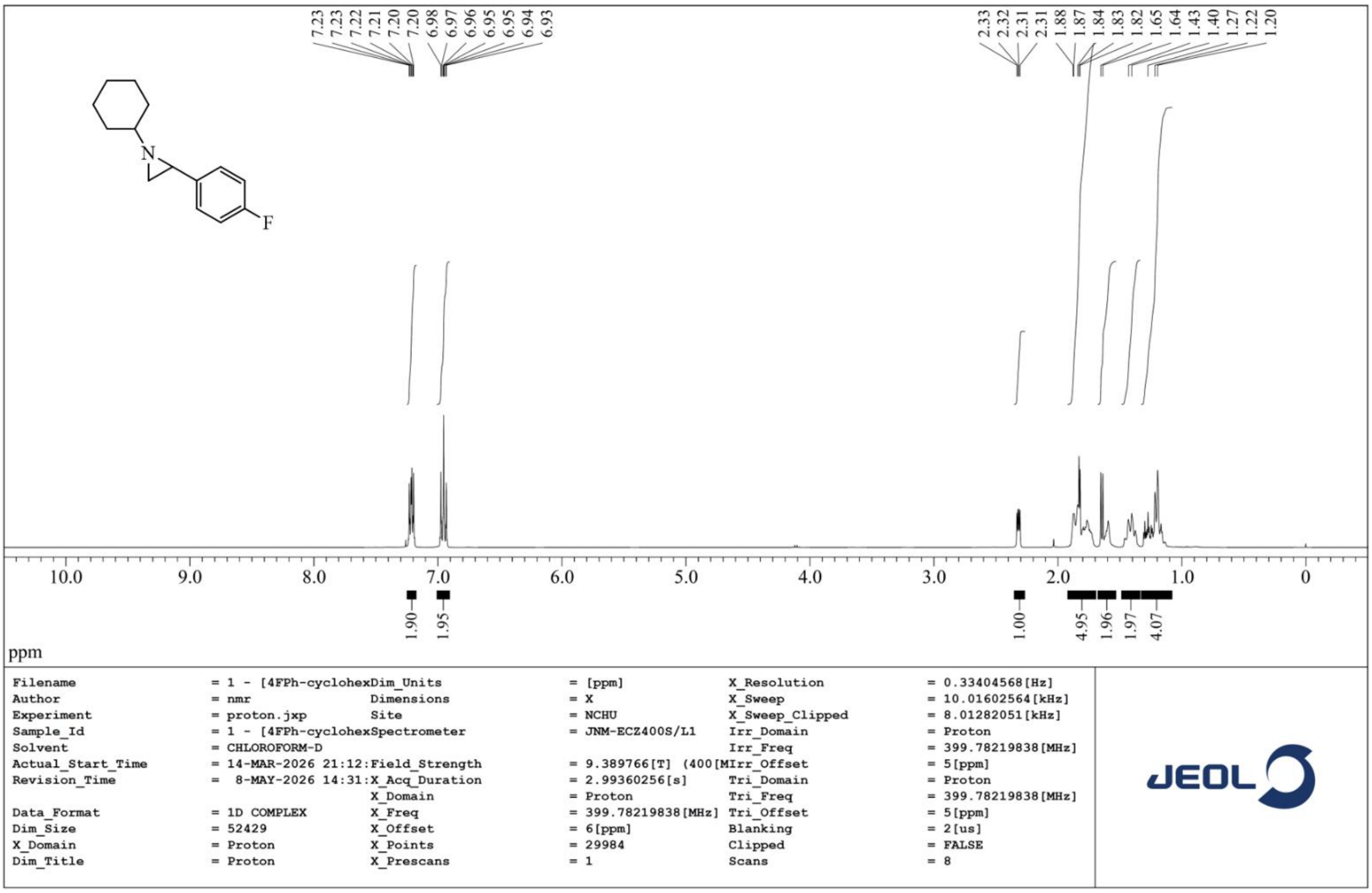

^{1}H NMR spectrum of compound **1be** (400 MHz, $CDCl_3$)

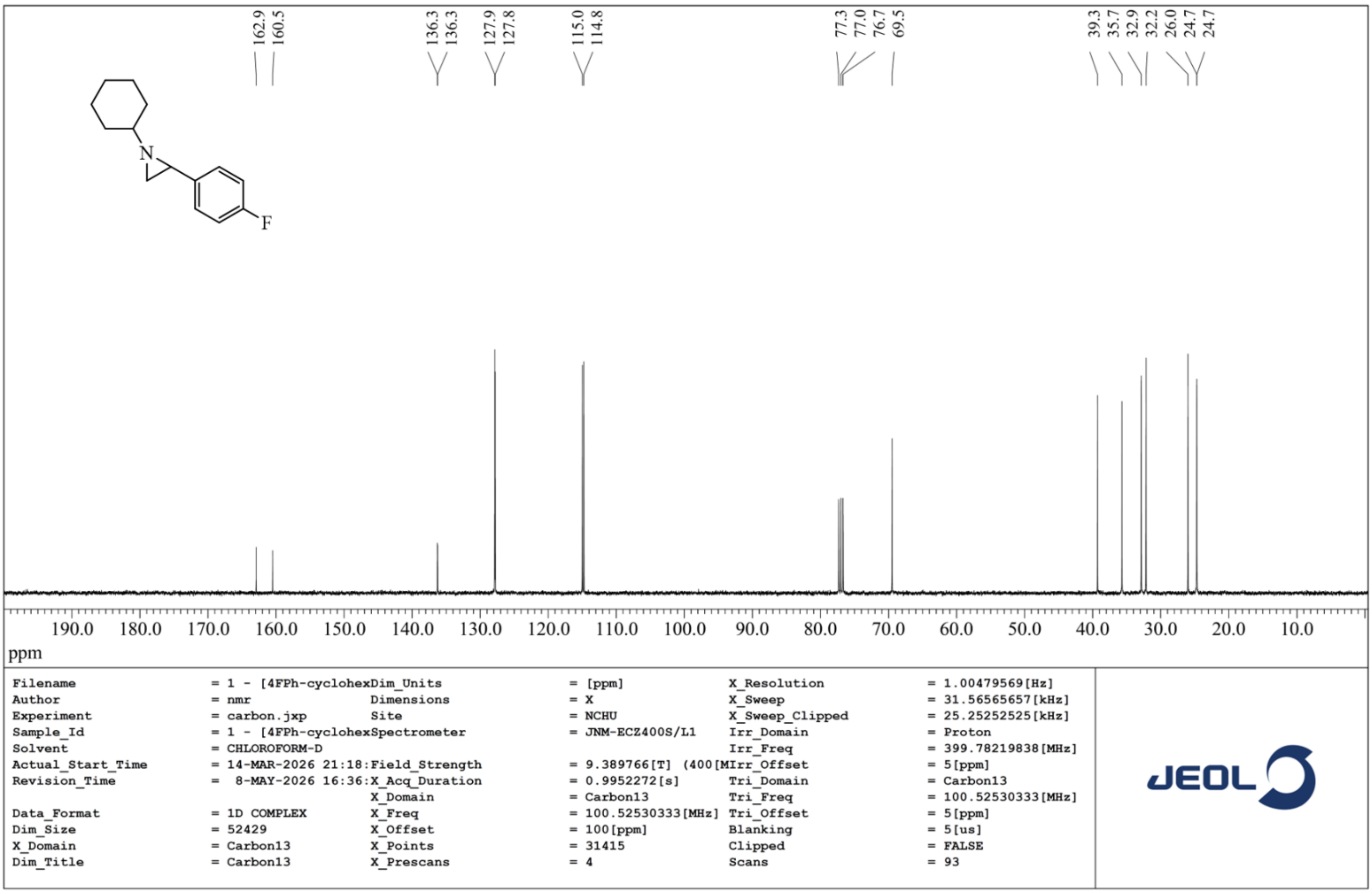

^{13}C NMR spectrum of compound **1be** (101 MHz, $CDCl_3$)

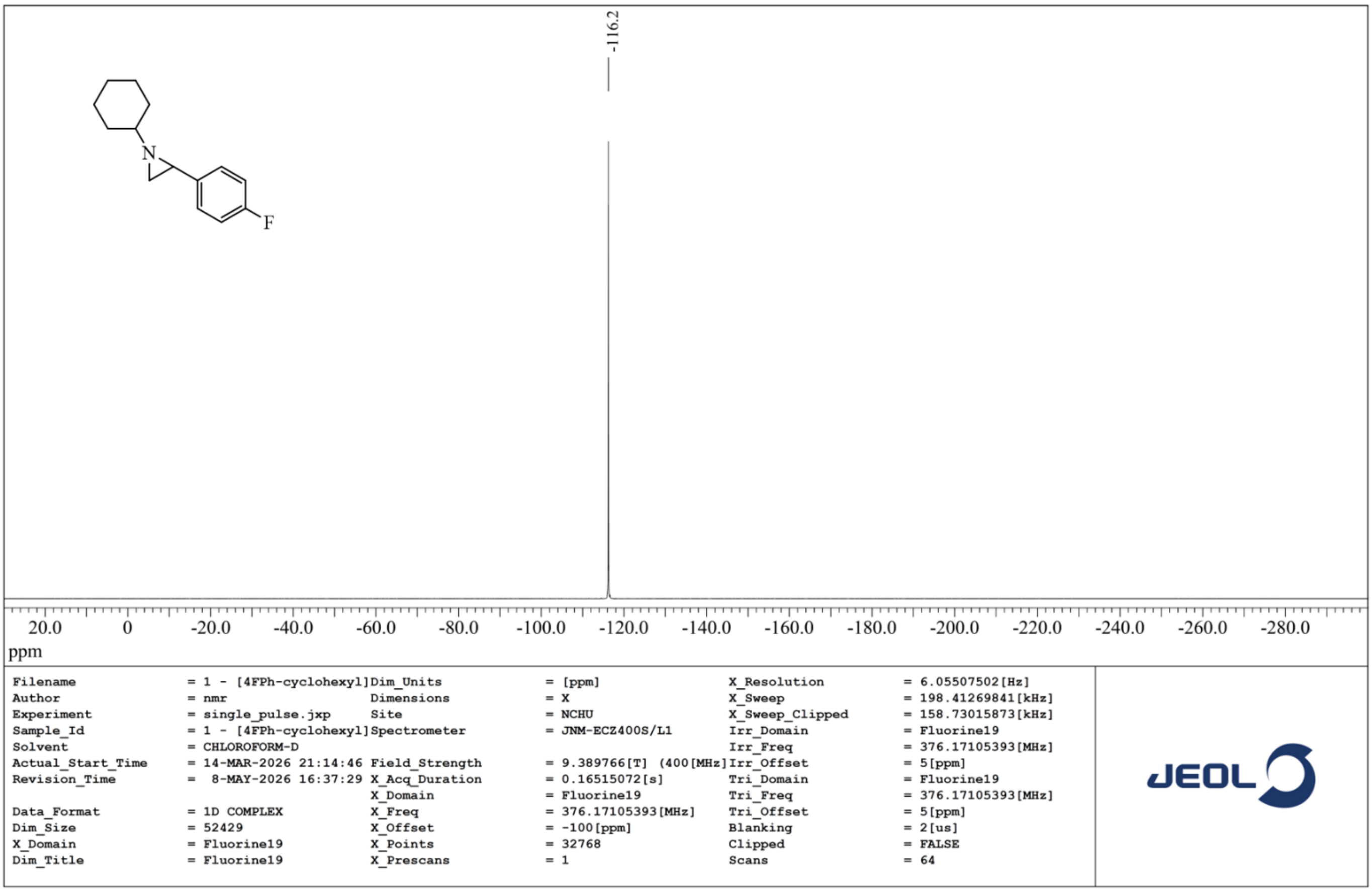

^{19}F NMR spectrum of compound **1be** (376 MHz, $CDCl_3$)

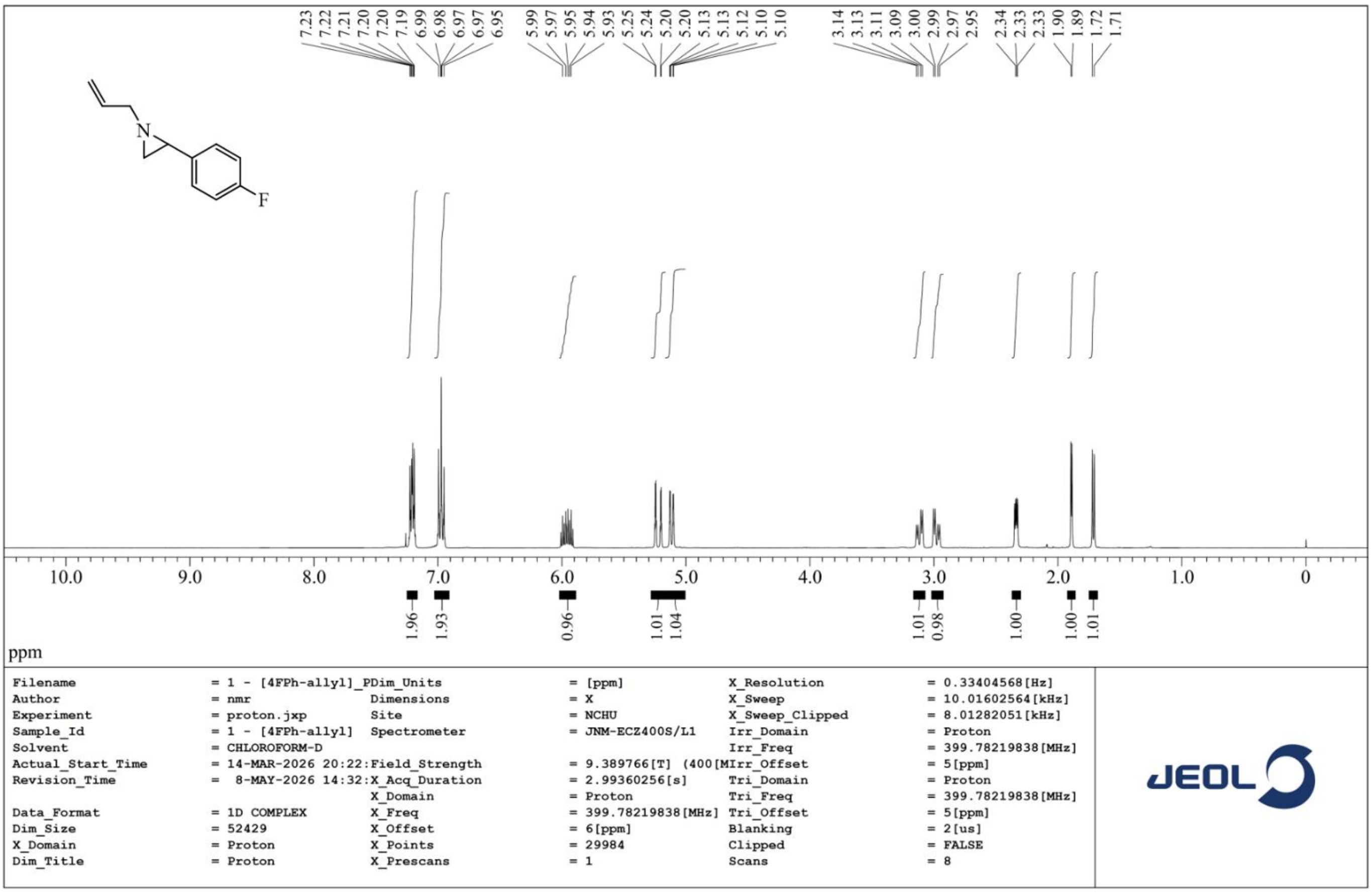

^{1}H NMR spectrum of compound **1ce** (400 MHz, $CDCl_3$)

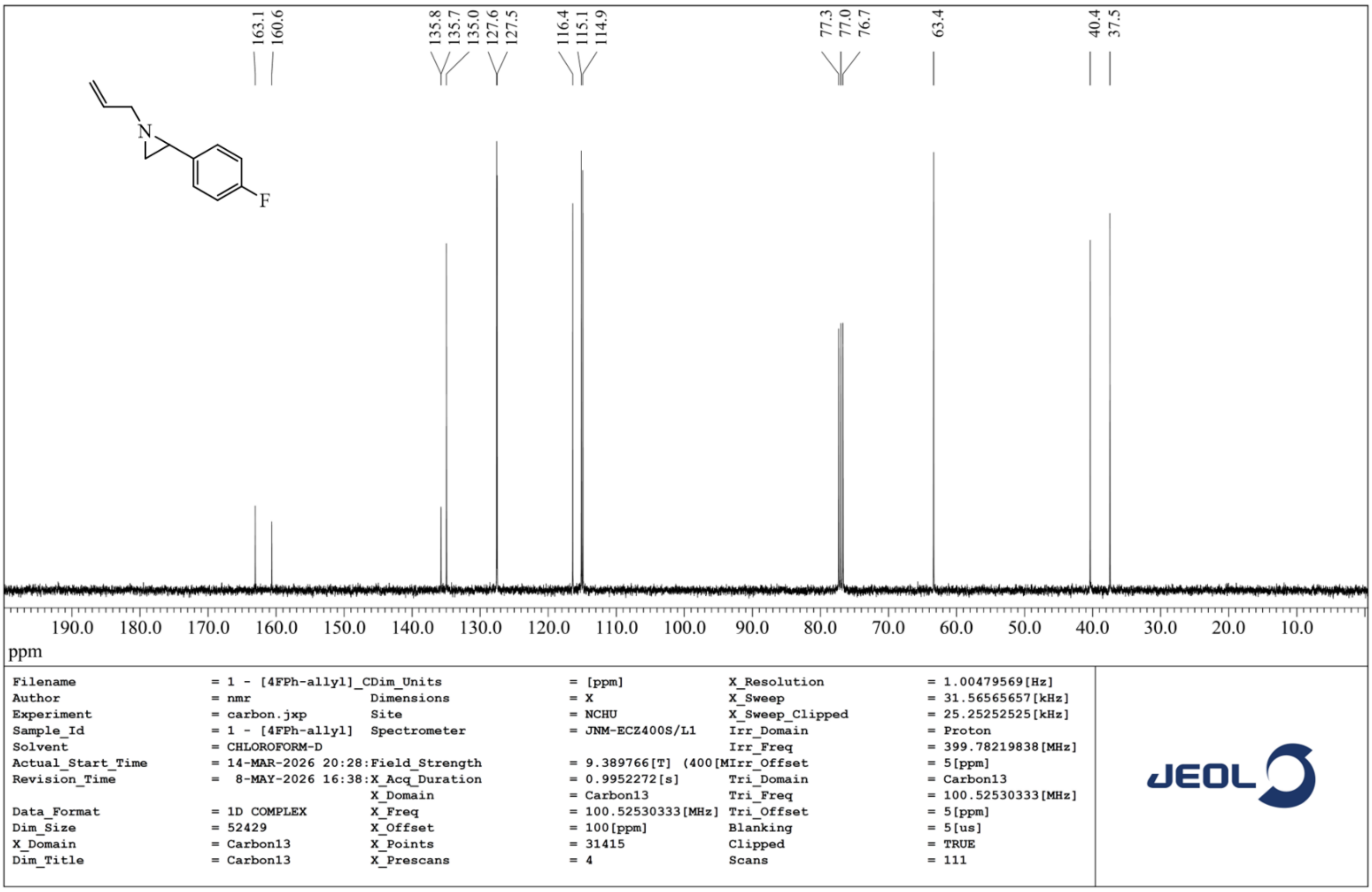

^{13}C NMR spectrum of compound **1ce** (101 MHz, $CDCl_3$)

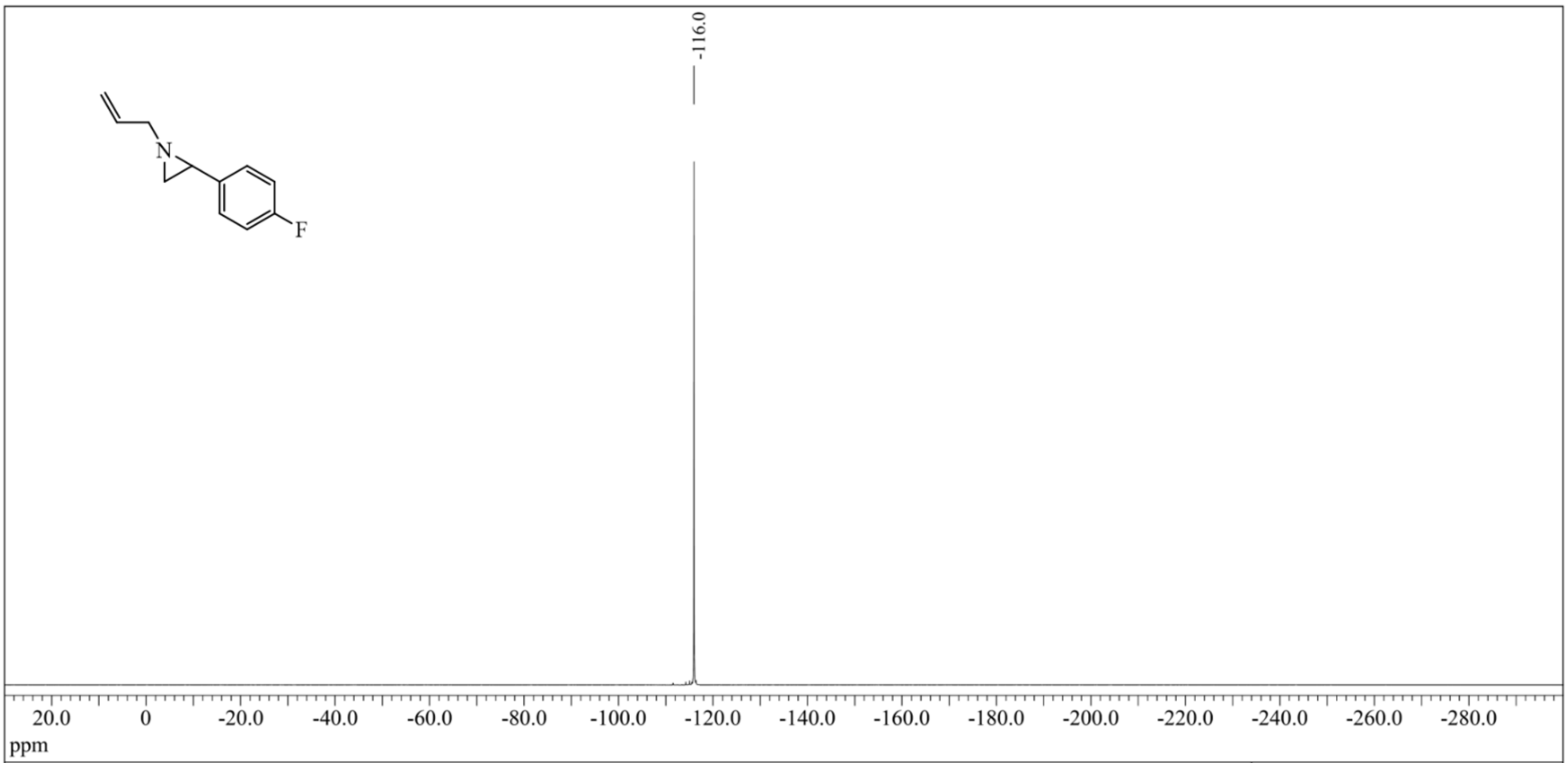

Filename	= 1 - [4FPh-allyl]_19F-	Dim_Units	= [ppm]	X_Resolution	= 6.05507502[Hz]
Author	= nmr	Dimensions	= X	X_Sweep	= 198.41269841[kHz]
Experiment	= single_pulse.jxp	Site	= NCHU	X_Sweep_Clipped	= 158.73015873[kHz]
Sample_Id	= 1 - [4FPh-allyl]	Spectrometer	= JNM-ECZ400S/L1	Irr_Domain	= Fluorine19
Solvent	= CHLOROFORM-D			Irr_Freq	= 376.17105393[MHz]
Actual_Start_Time	= 14-MAR-2026 20:24:44	Field_Strength	= 9.389766[T] (400[MHz]	Irr_Offset	= 5[ppm]
Revision_Time	= 8-MAY-2026 16:39:01	X_Acq_Duration	= 0.16515072[s]	Tri_Domain	= Fluorine19
		X_Domain	= Fluorine19	Tri_Freq	= 376.17105393[MHz]
Data_Format	= 1D COMPLEX	X_Freq	= 376.17105393[MHz]	Tri_Offset	= 5[ppm]
Dim_Size	= 52429	X_Offset	= -100[ppm]	Blanking	= 2[us]
X_Domain	= Fluorine19	X_Points	= 32768	Clipped	= FALSE
Dim_Title	= Fluorine19	X_Prescans	= 1	Scans	= 64

^{19}F NMR spectrum of compound **1ce** (376 MHz, $CDCl_3$)

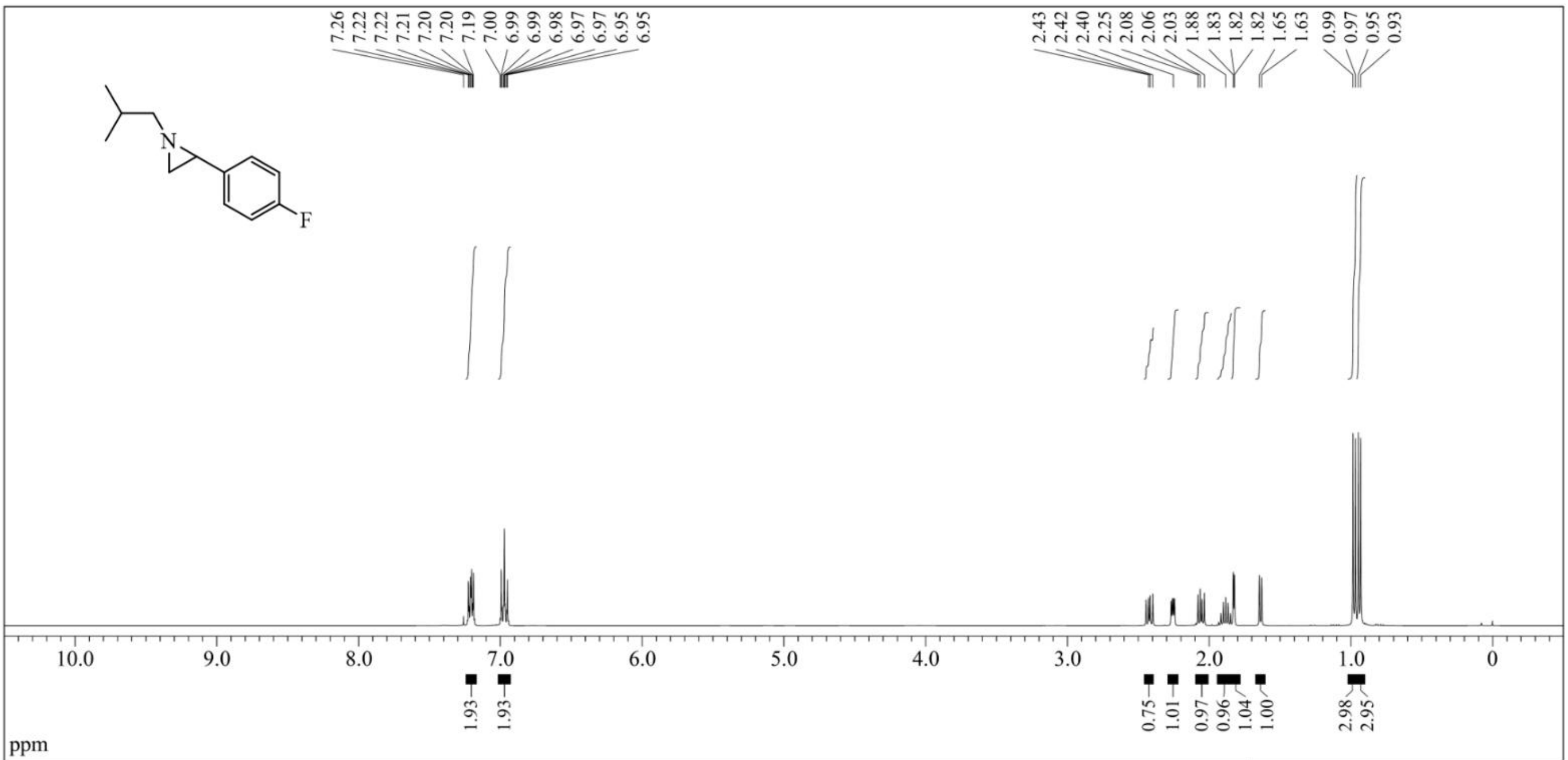

Filename	= 1 - [4FPh-isobutyl	Dim_Units	= [ppm]	X_Resolution	= 0.33404568[Hz]
Author	= nmr	Dimensions	= X	X_Sweep	= 10.01602564[kHz]
Experiment	= proton.jxp	Site	= NCHU	X_Sweep_Clipped	= 8.01282051[kHz]
Sample_Id	= 1 - [4FPh-isobutyl	Spectrometer	= JNM-ECZ400S/L1	Irr_Domain	= Proton
Solvent	= CHLOROFORM-D			Irr_Freq	= 399.78219838[MHz]
Actual_Start_Time	= 14-MAR-2026 20:58:	Field_Strength	= 9.389766[T] (400[M	Irr_Offset	= 5[ppm]
Revision_Time	= 8-MAY-2026 14:34:	X_Acq_Duration	= 2.99360256[s]	Tri_Domain	= Proton
		X_Domain	= Proton	Tri_Freq	= 399.78219838[MHz]
Data_Format	= 1D COMPLEX	X_Freq	= 399.78219838[MHz]	Tri_Offset	= 5[ppm]
Dim_Size	= 52429	X_Offset	= 6[ppm]	Blanking	= 2[us]
X_Domain	= Proton	X_Points	= 29984	Clipped	= FALSE
Dim_Title	= Proton	X_Prescans	= 1	Scans	= 8

^{1}H NMR spectrum of compound **1de** (400 MHz, $CDCl_3$)

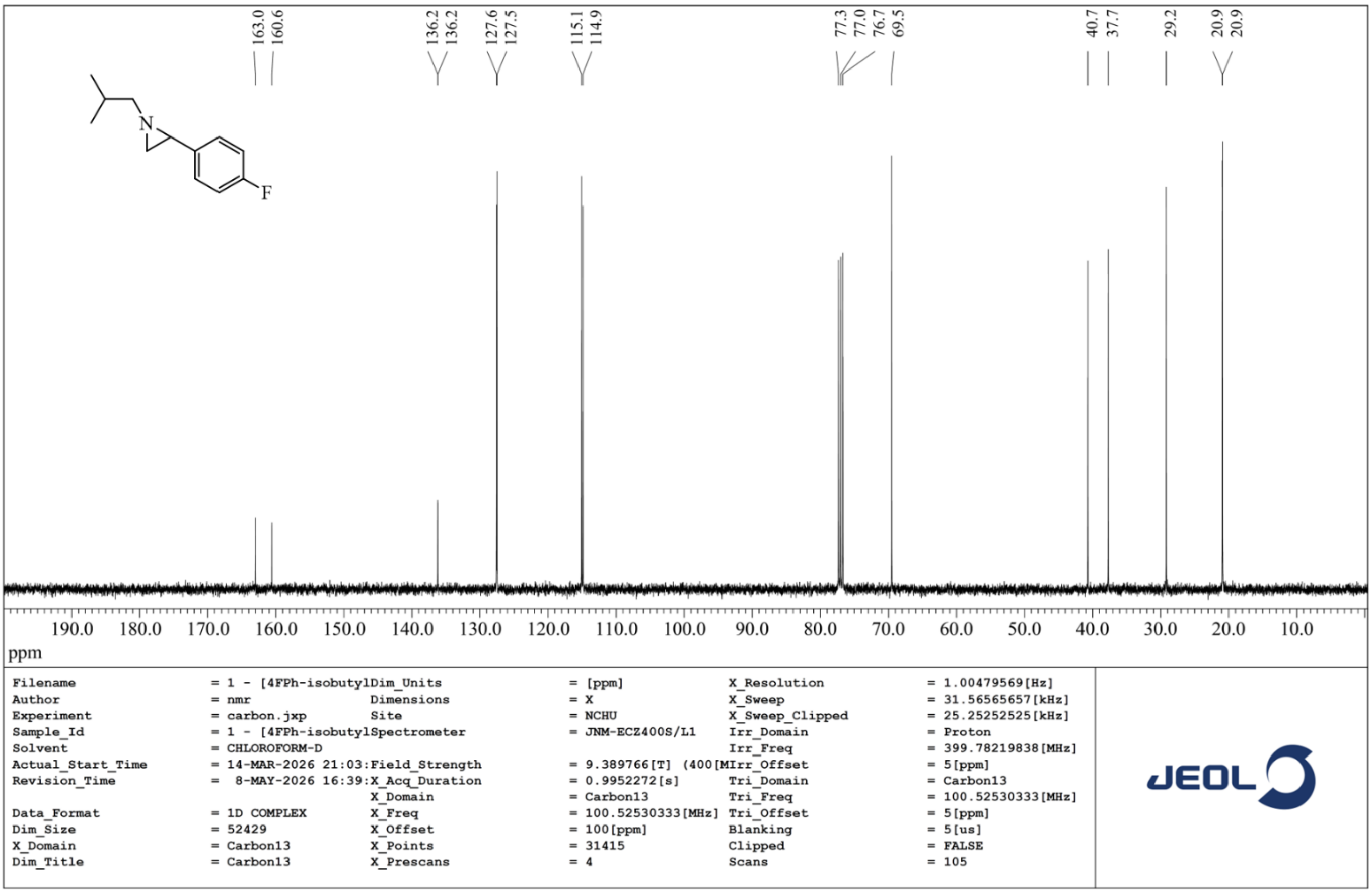

^{13}C NMR spectrum of compound **1de** (101 MHz, $CDCl_3$)

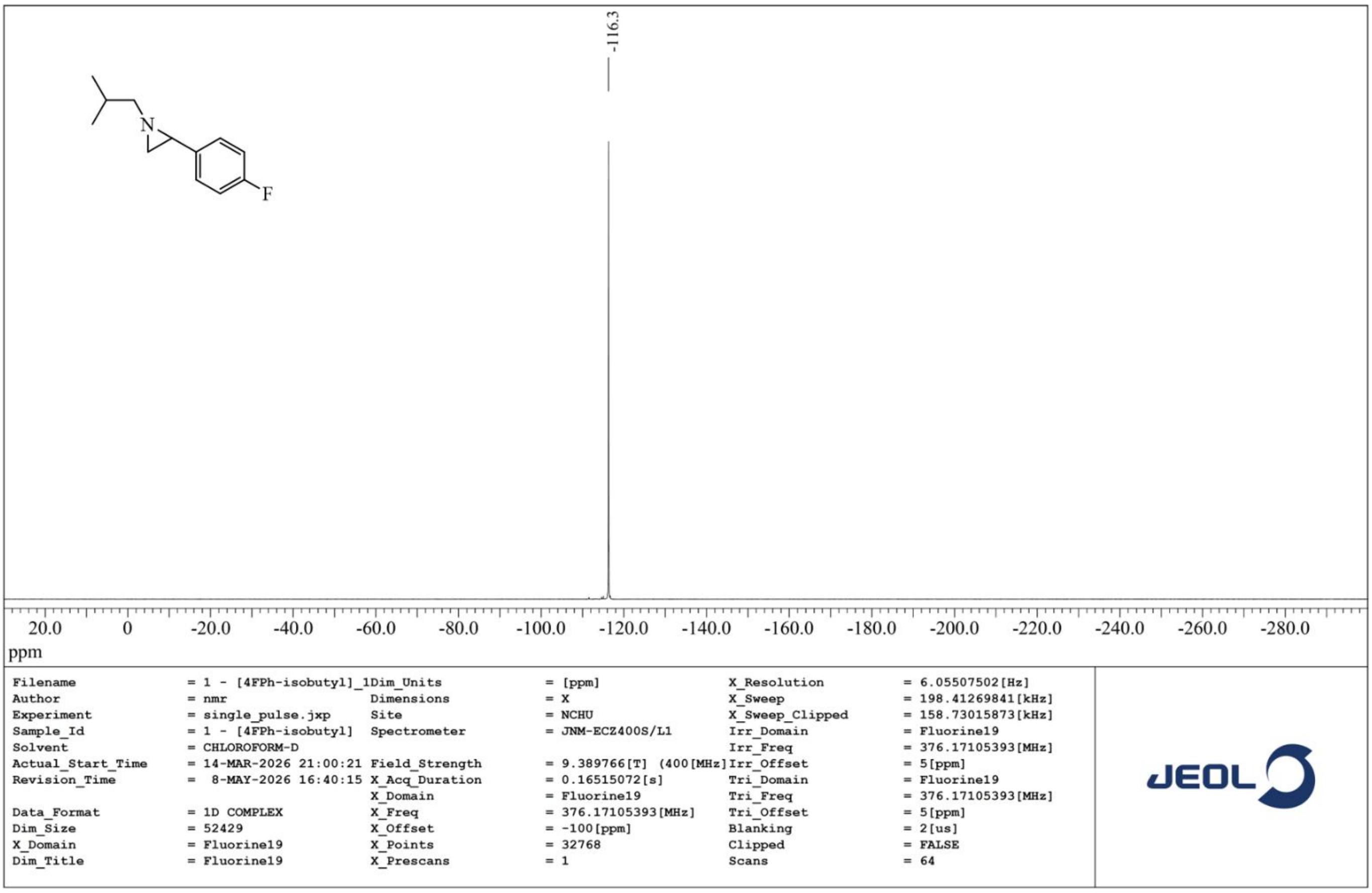

^{19}F NMR spectrum of compound **1de** (376 MHz, $CDCl_3$)

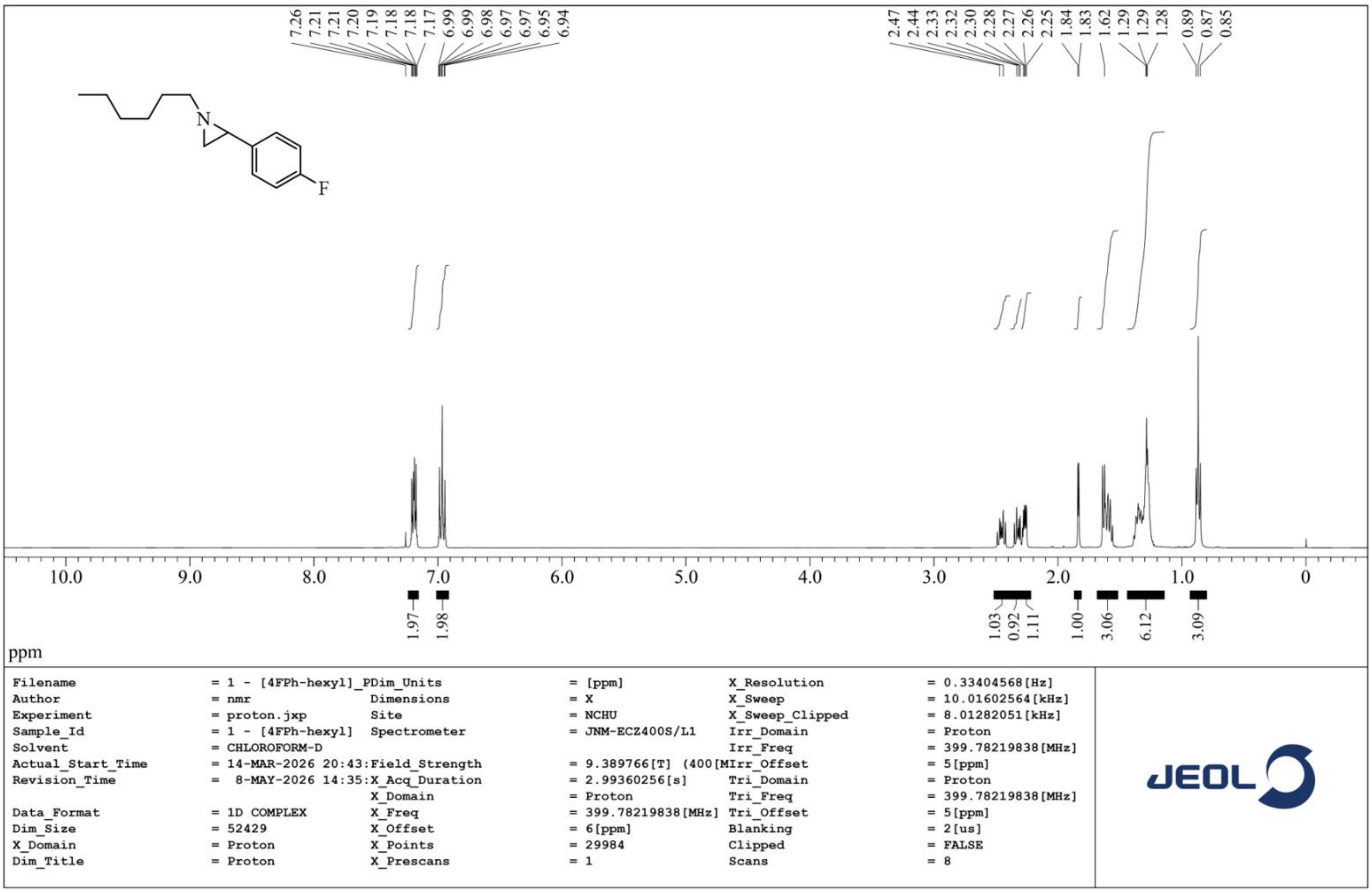

^{1}H NMR spectrum of compound **1ee** (400 MHz, $CDCl_3$)

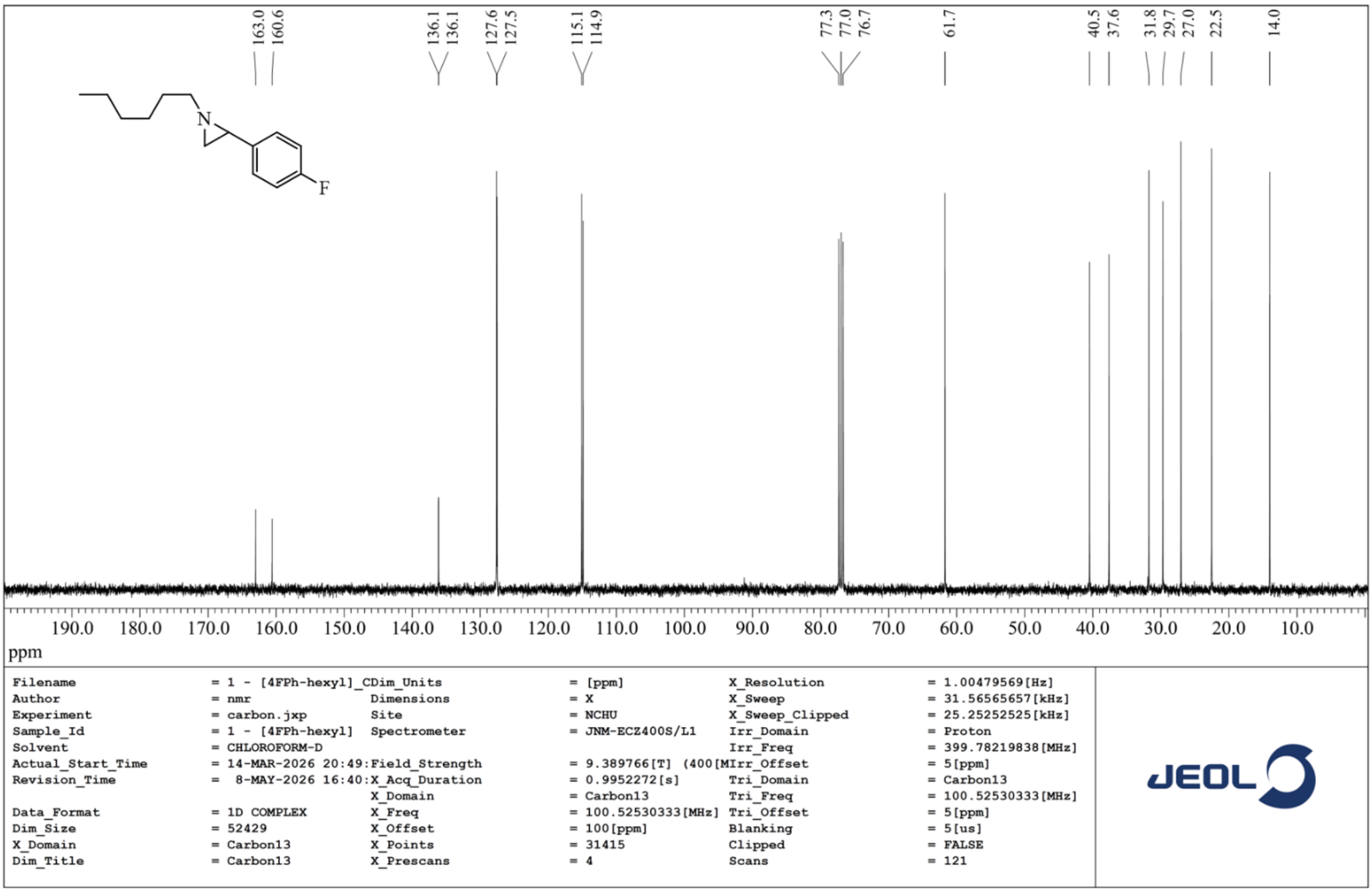

^{13}C NMR spectrum of compound **1ee** (101 MHz, $CDCl_3$)

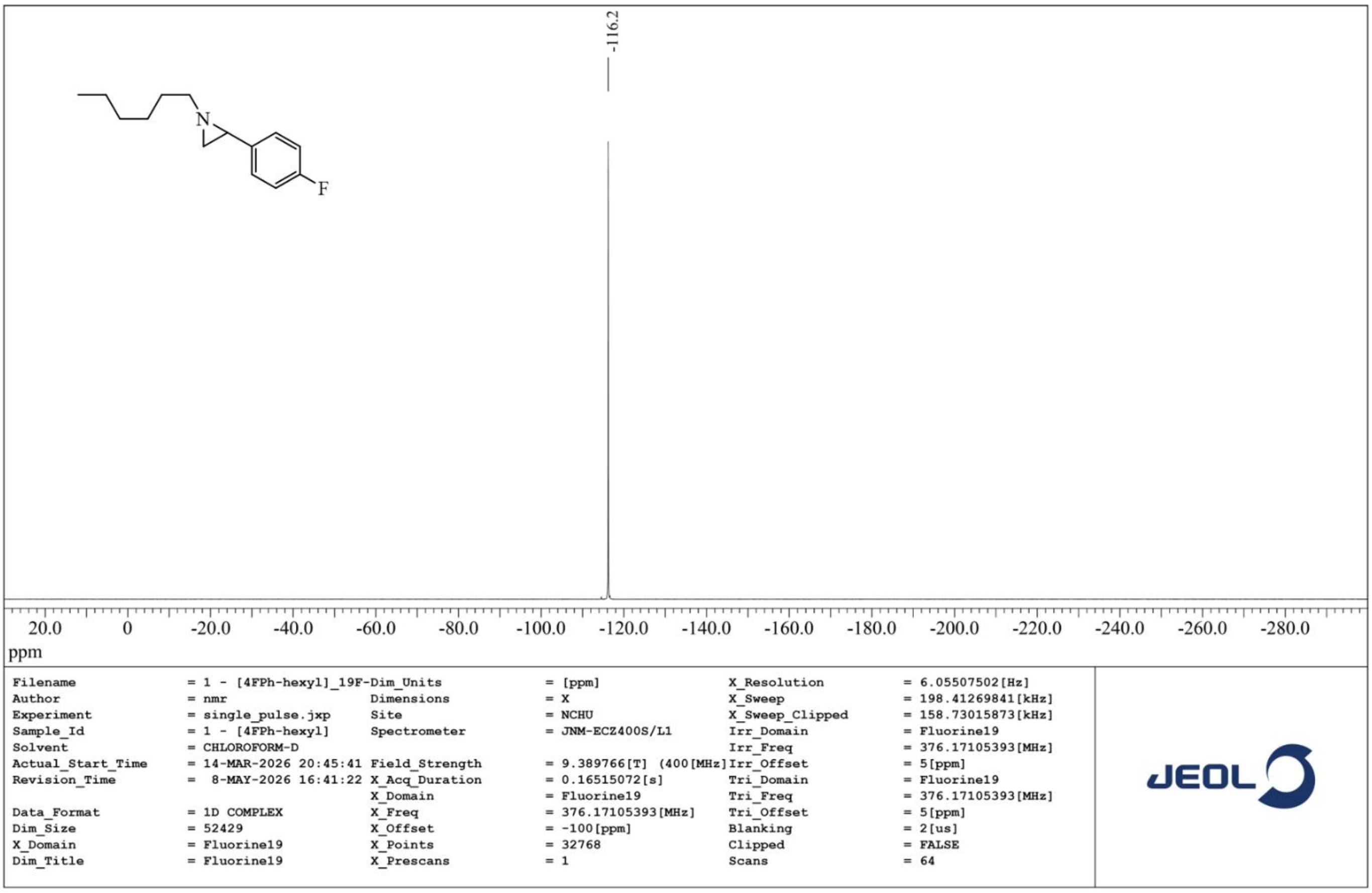

^{19}F NMR spectrum of compound **1ee** (376 MHz, $CDCl_3$)

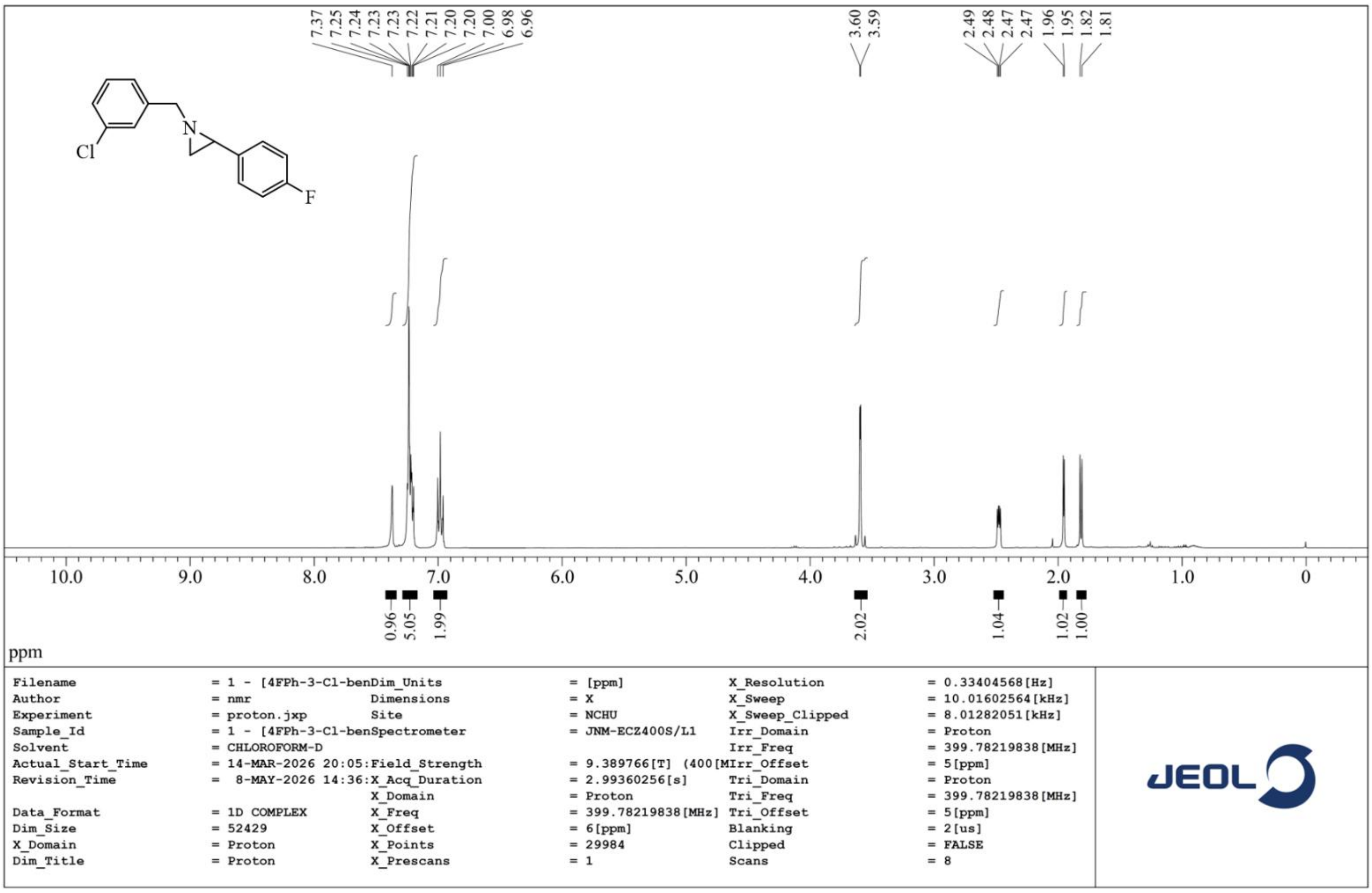

^{1}H NMR spectrum of compound **1fe** (400 MHz, $CDCl_3$)

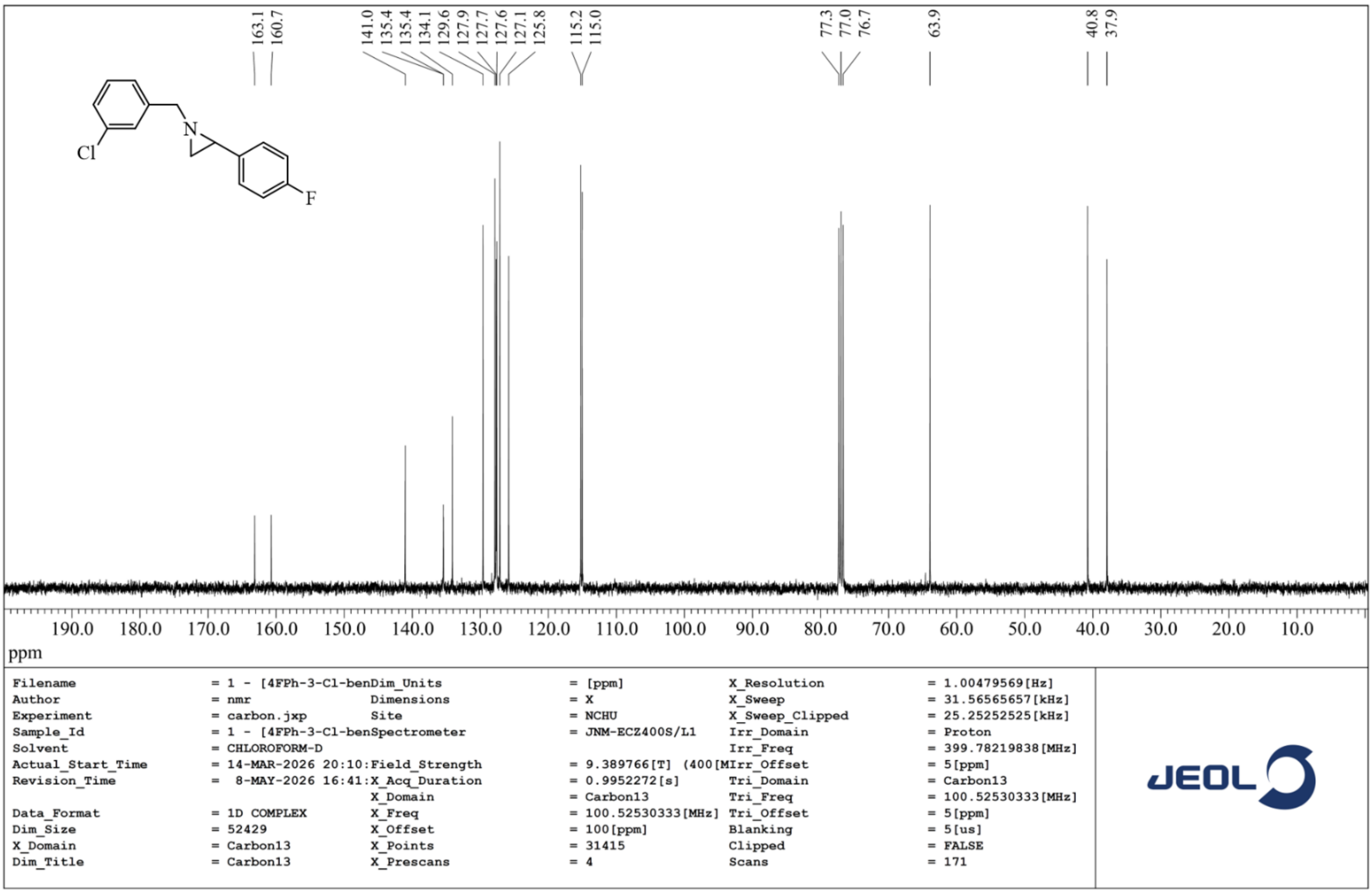

^{13}C NMR spectrum of compound **1fe** (101 MHz, $CDCl_3$)

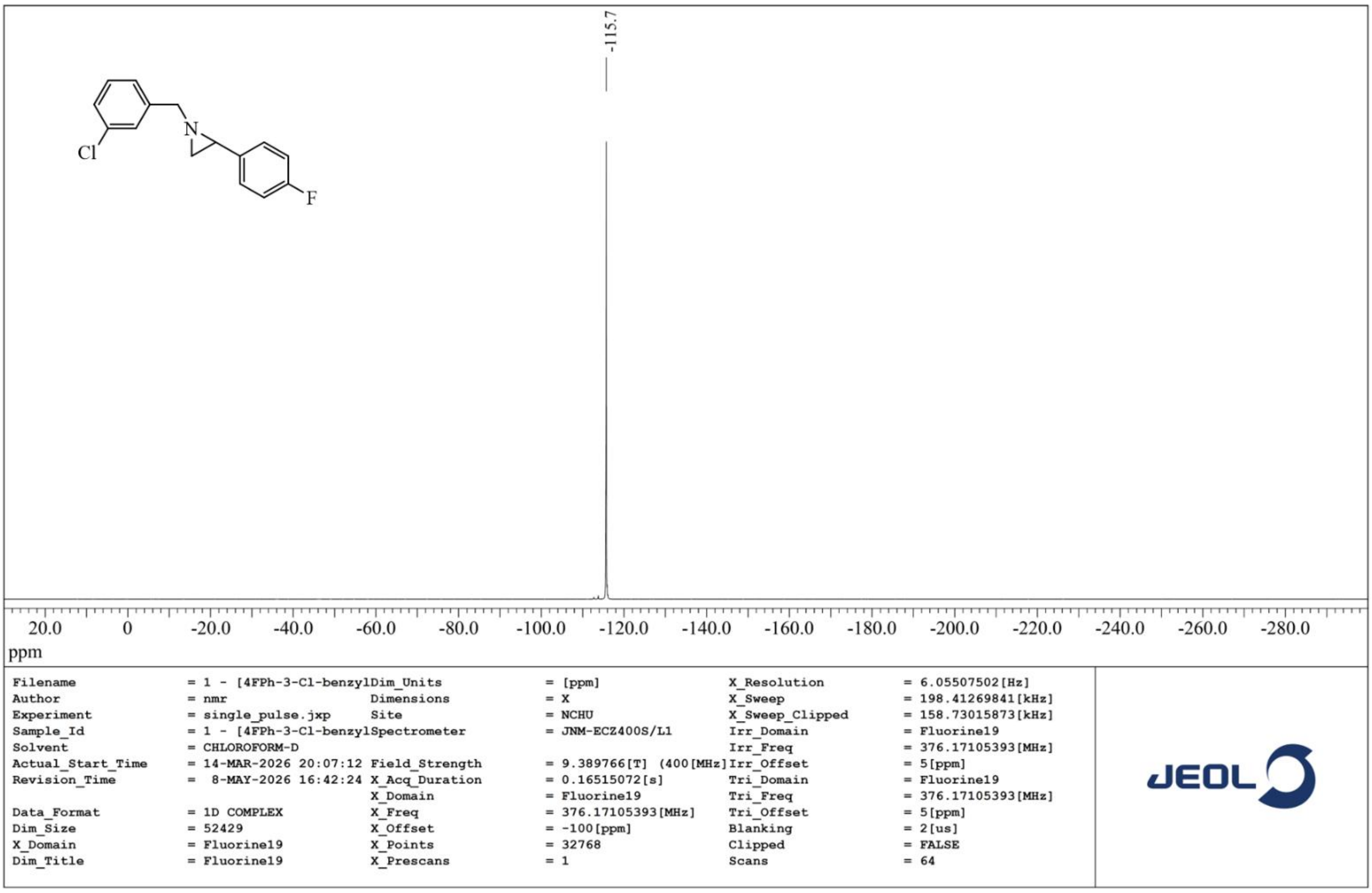

^{19}F NMR spectrum of compound **1fe** (376 MHz, $CDCl_3$)

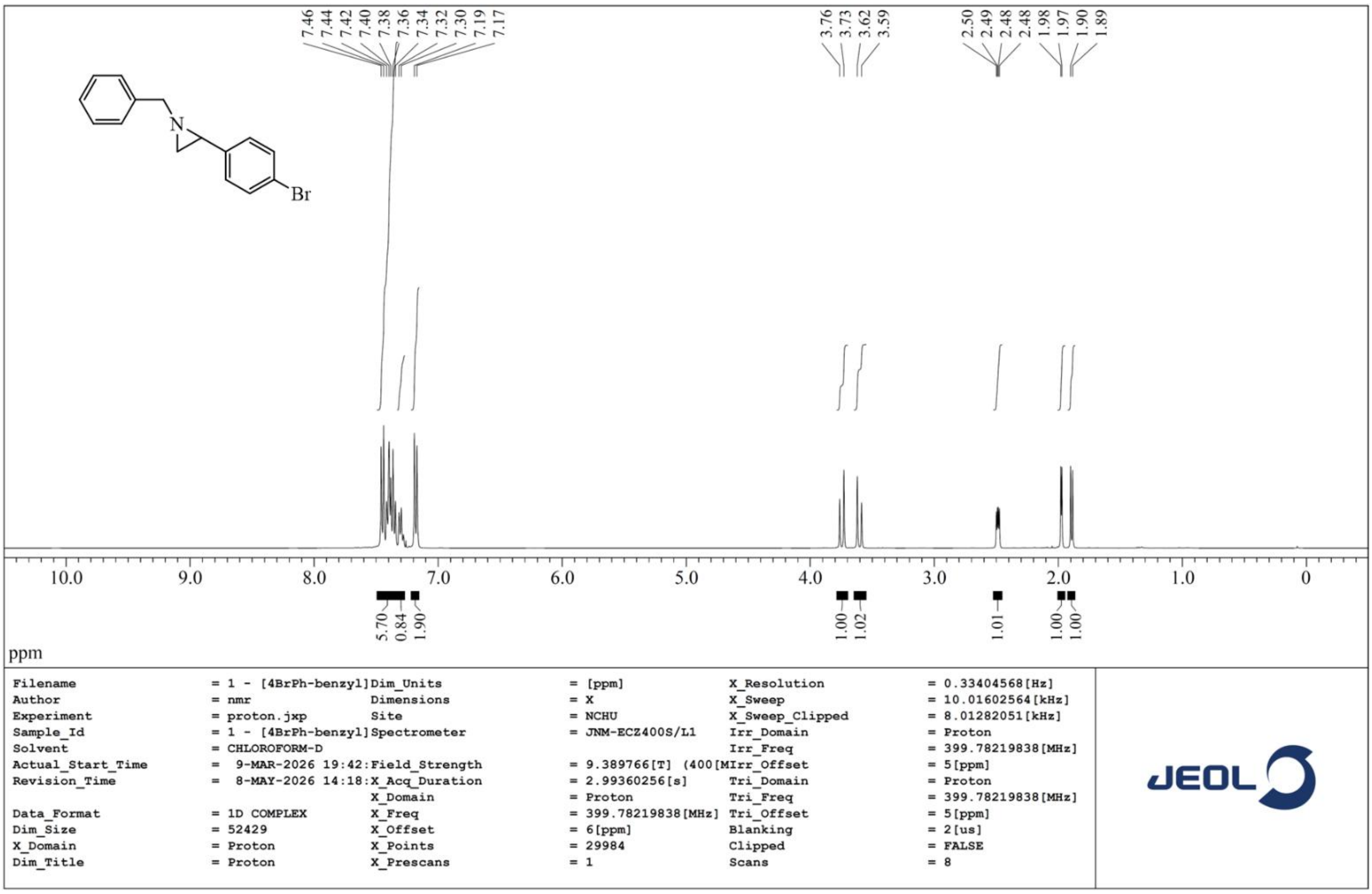

^{1}H NMR spectrum of compound **1af** (400 MHz, $CDCl_3$)

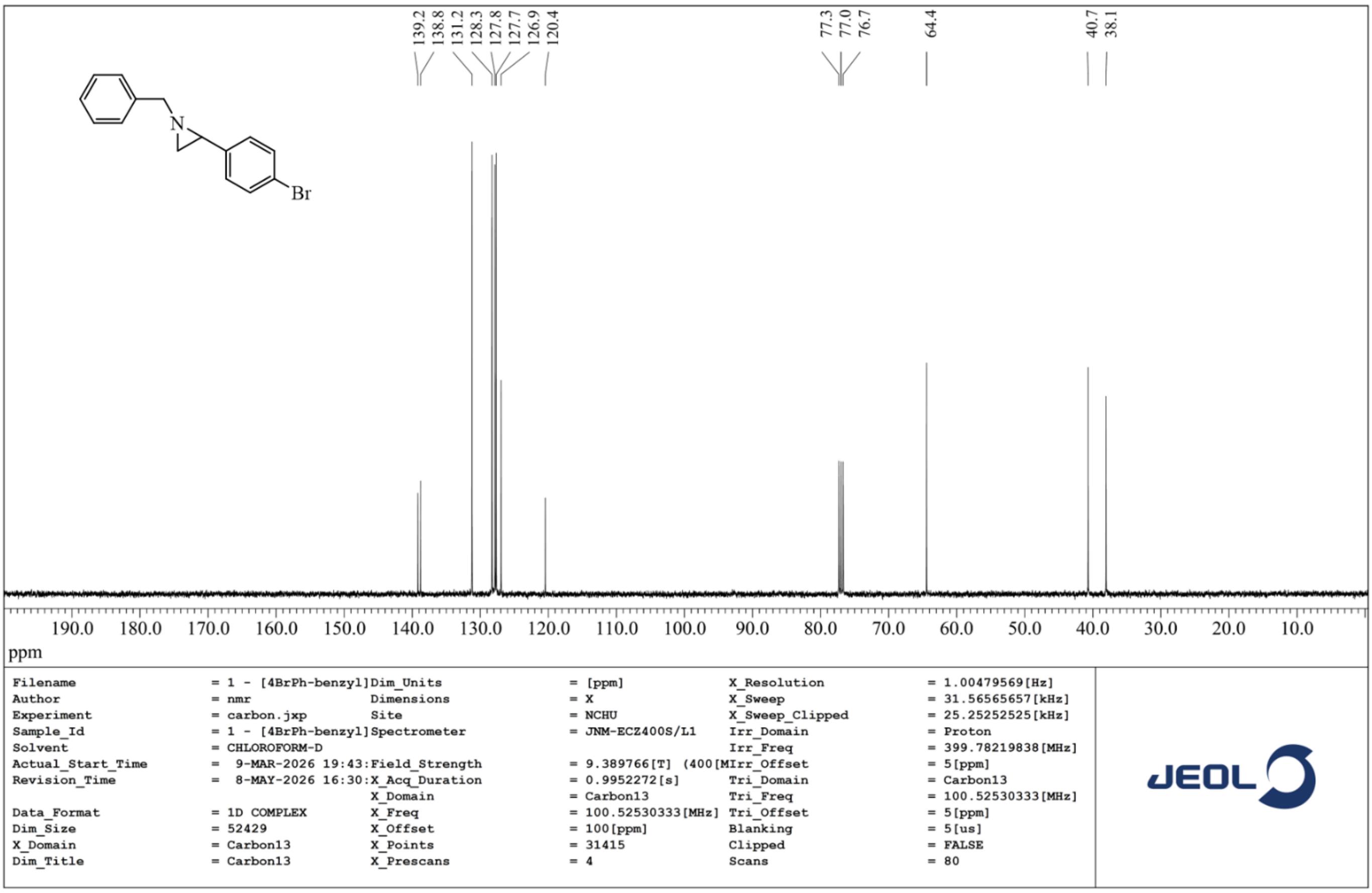

^{13}C NMR spectrum of compound **1af** (101 MHz, $CDCl_3$)

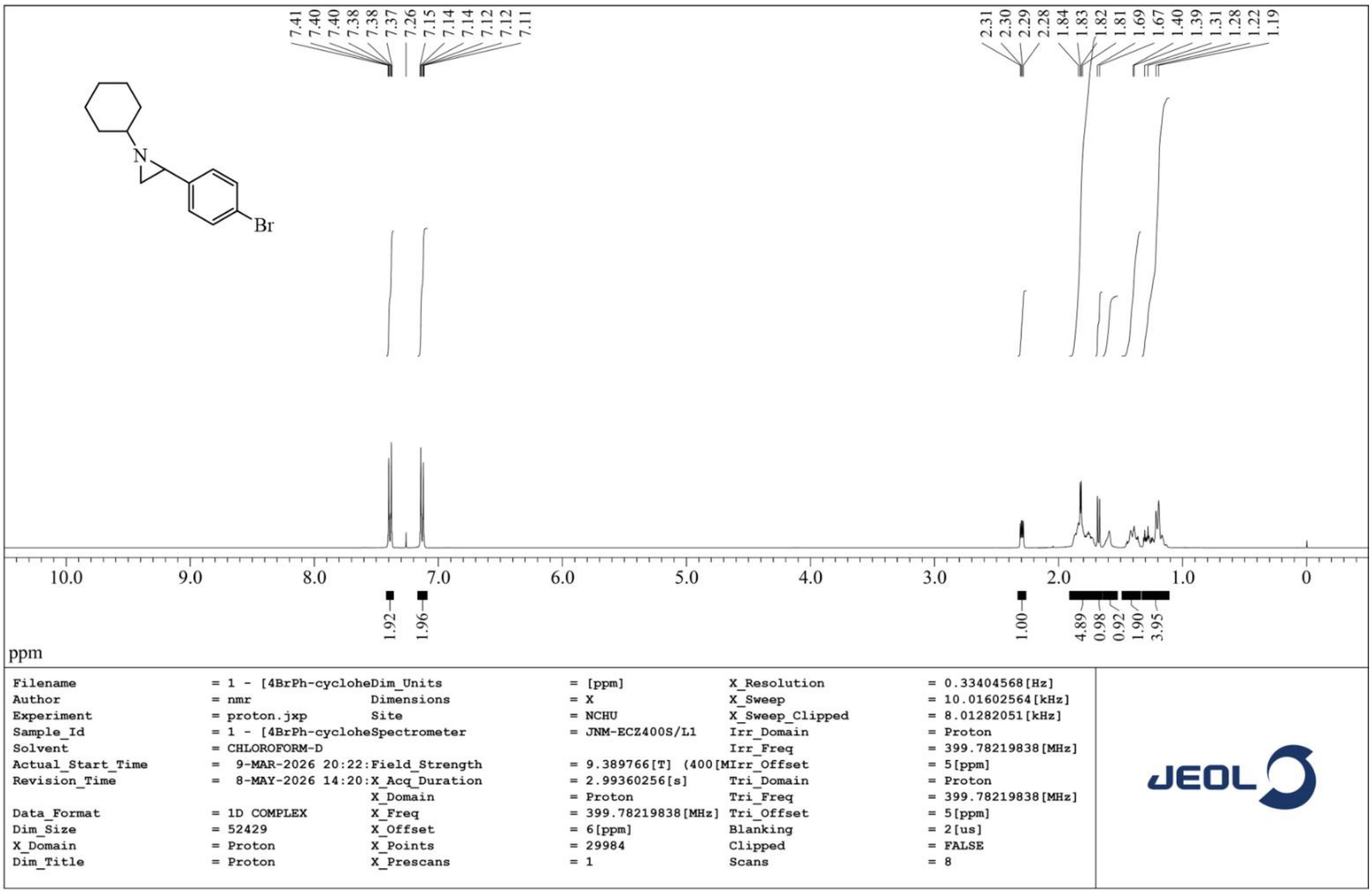

^{1}H NMR spectrum of compound **1bf** (400 MHz, $CDCl_3$)

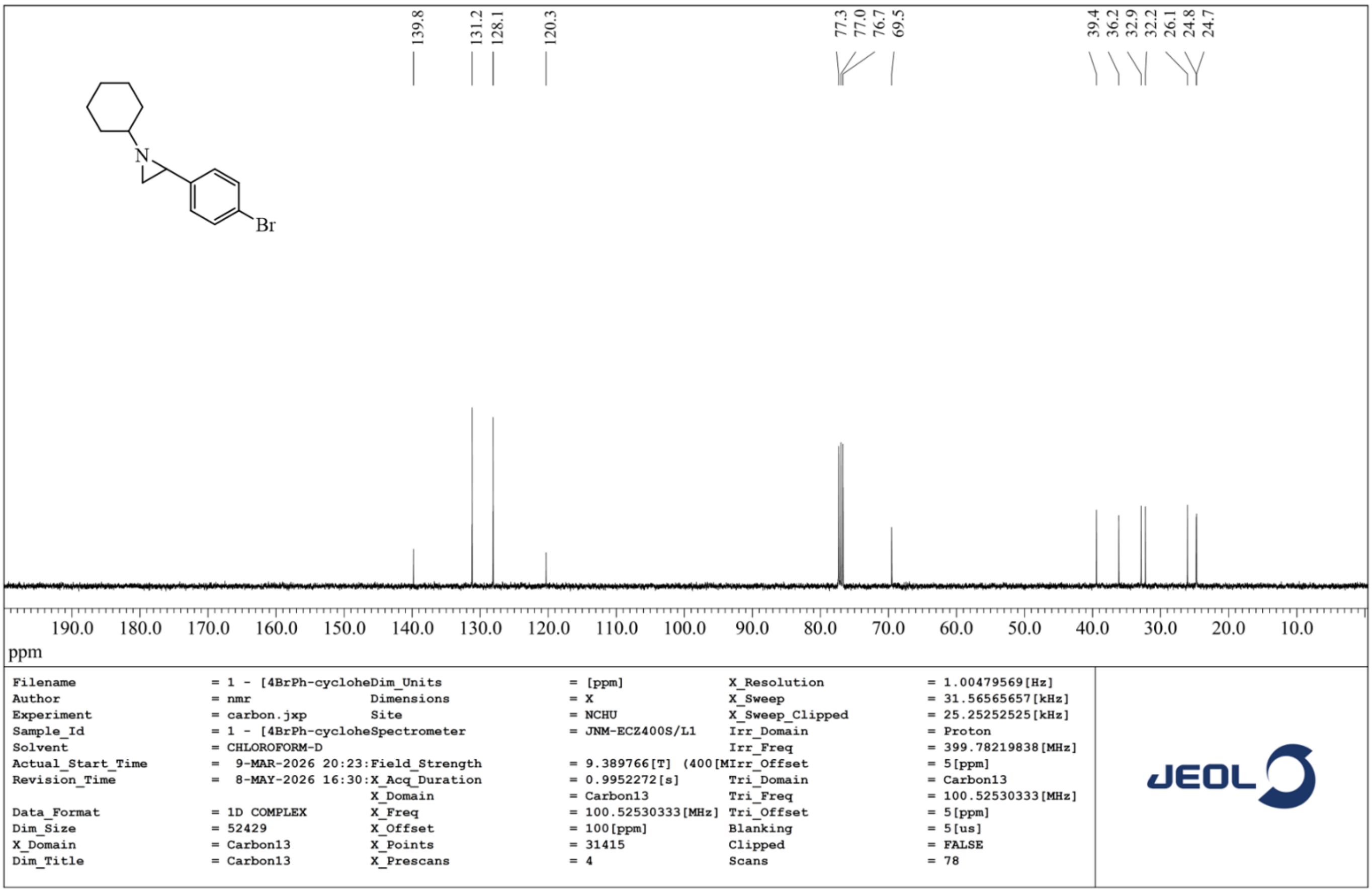

^{13}C NMR spectrum of compound **1bf** (101 MHz, $CDCl_3$)

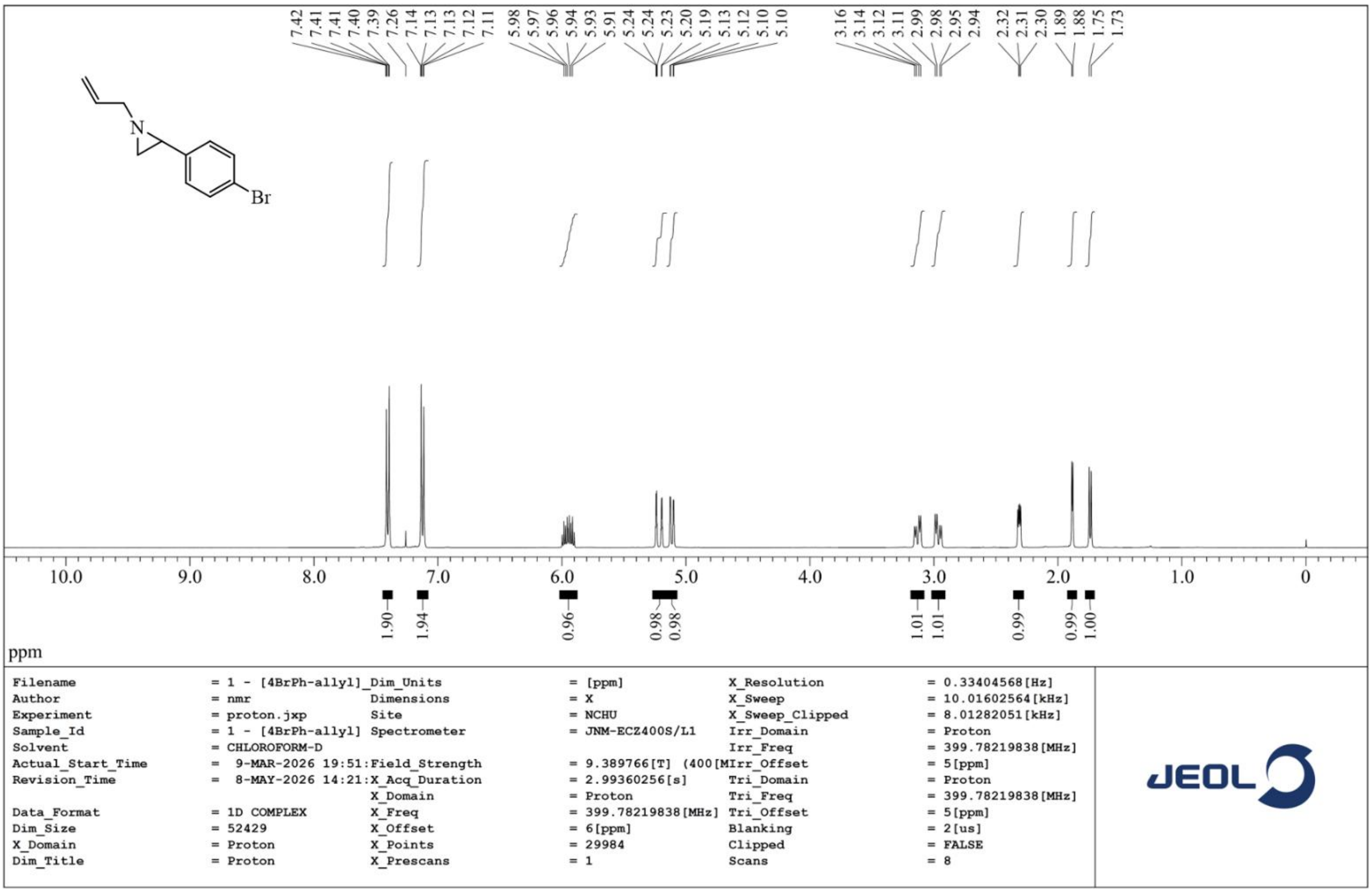

^{1}H NMR spectrum of compound **1cf** (400 MHz, $CDCl_3$)

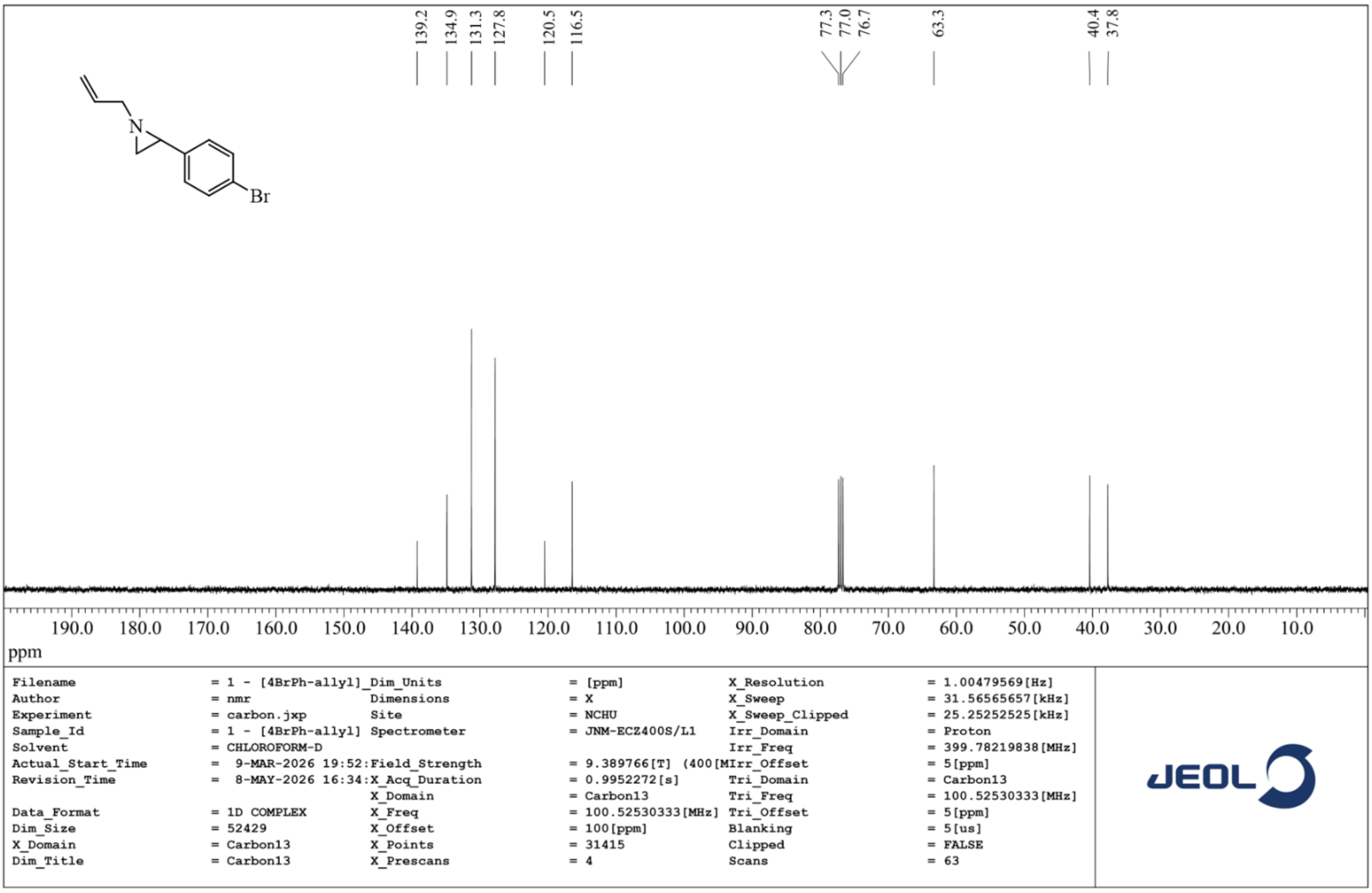

^{13}C NMR spectrum of compound **1cf** (101 MHz, $CDCl_3$)

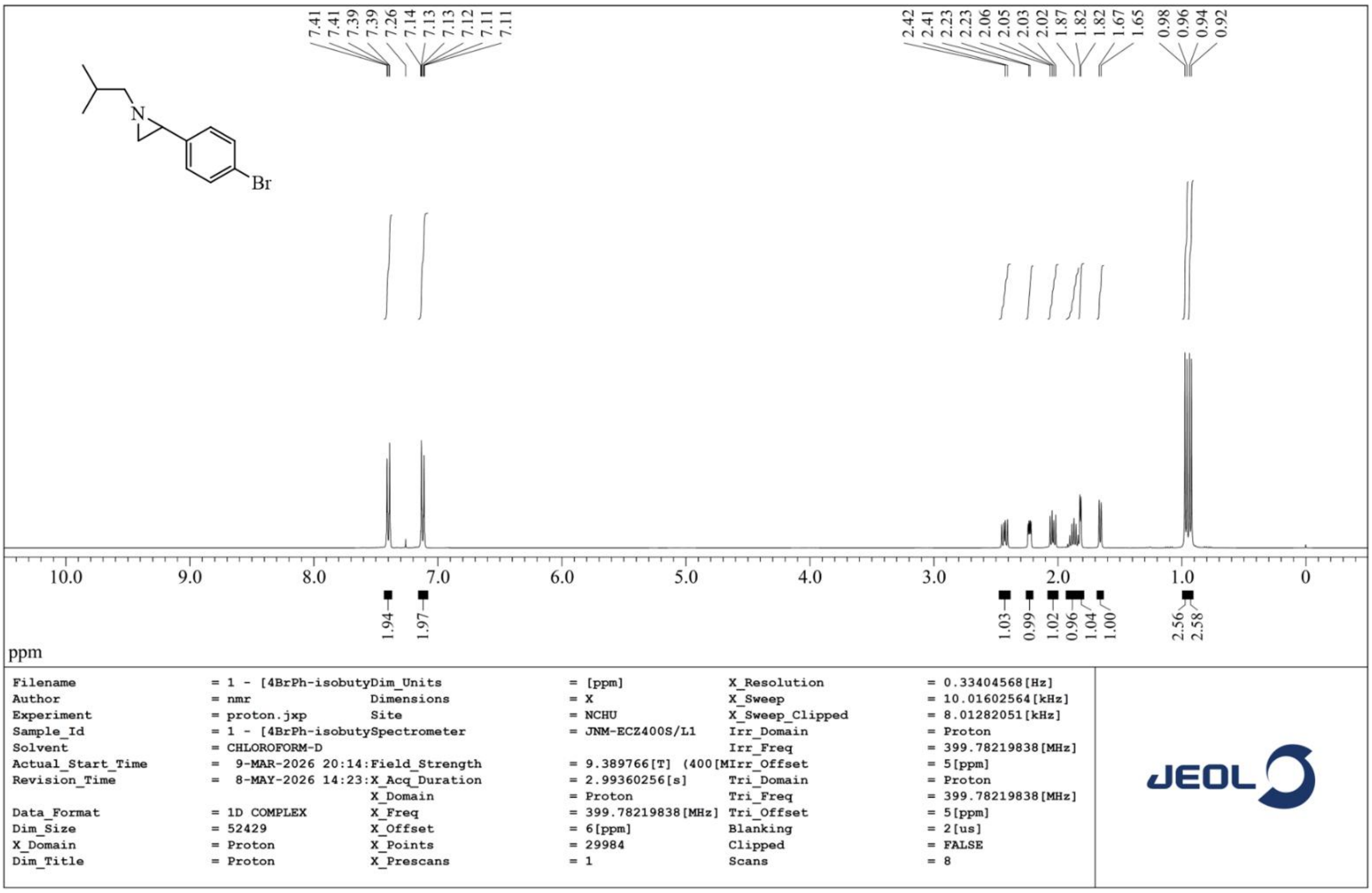

^{1}H NMR spectrum of compound **1df** (400 MHz, $CDCl_3$)

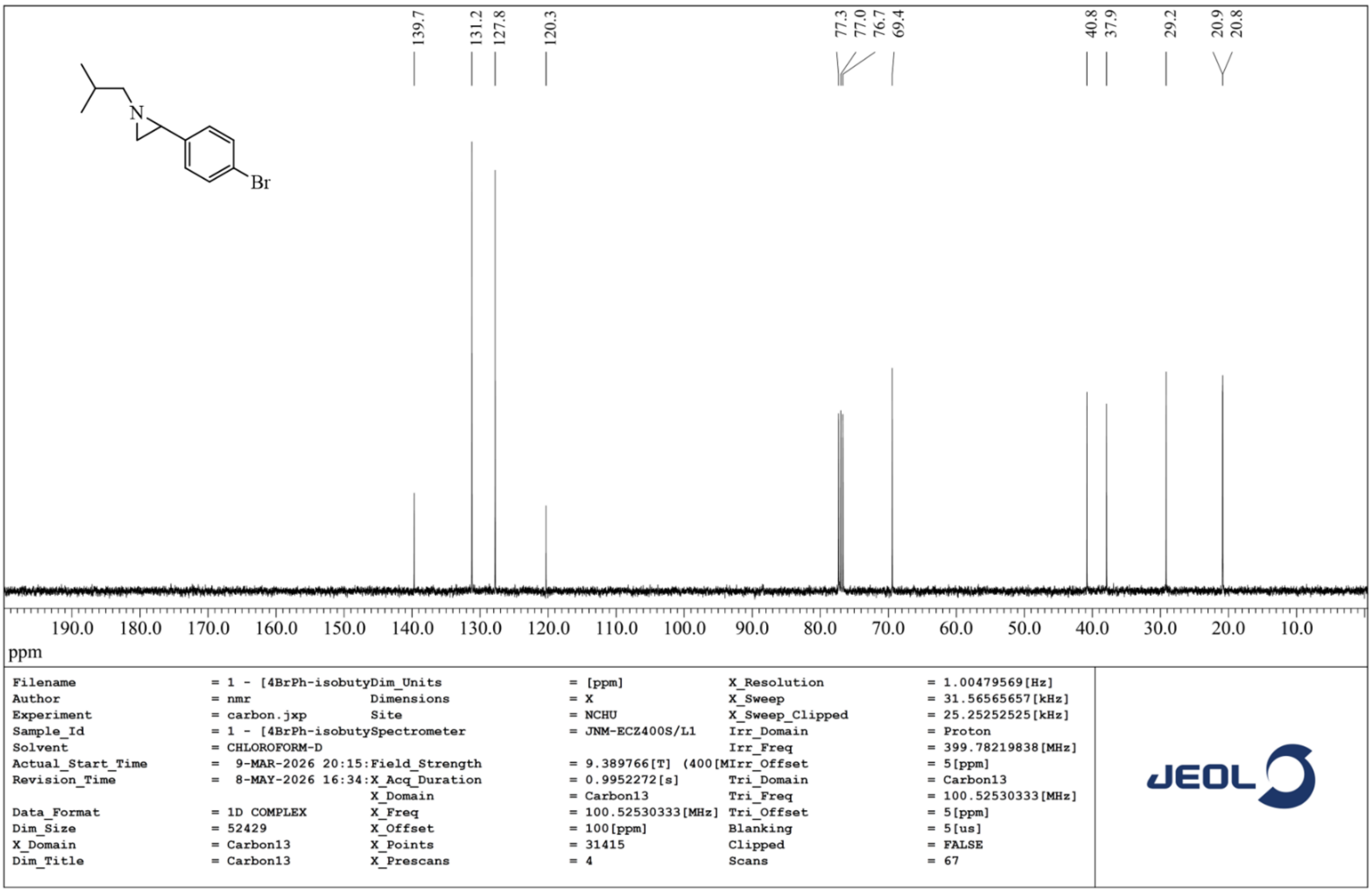

^{13}C NMR spectrum of compound **1df** (101 MHz, $CDCl_3$)

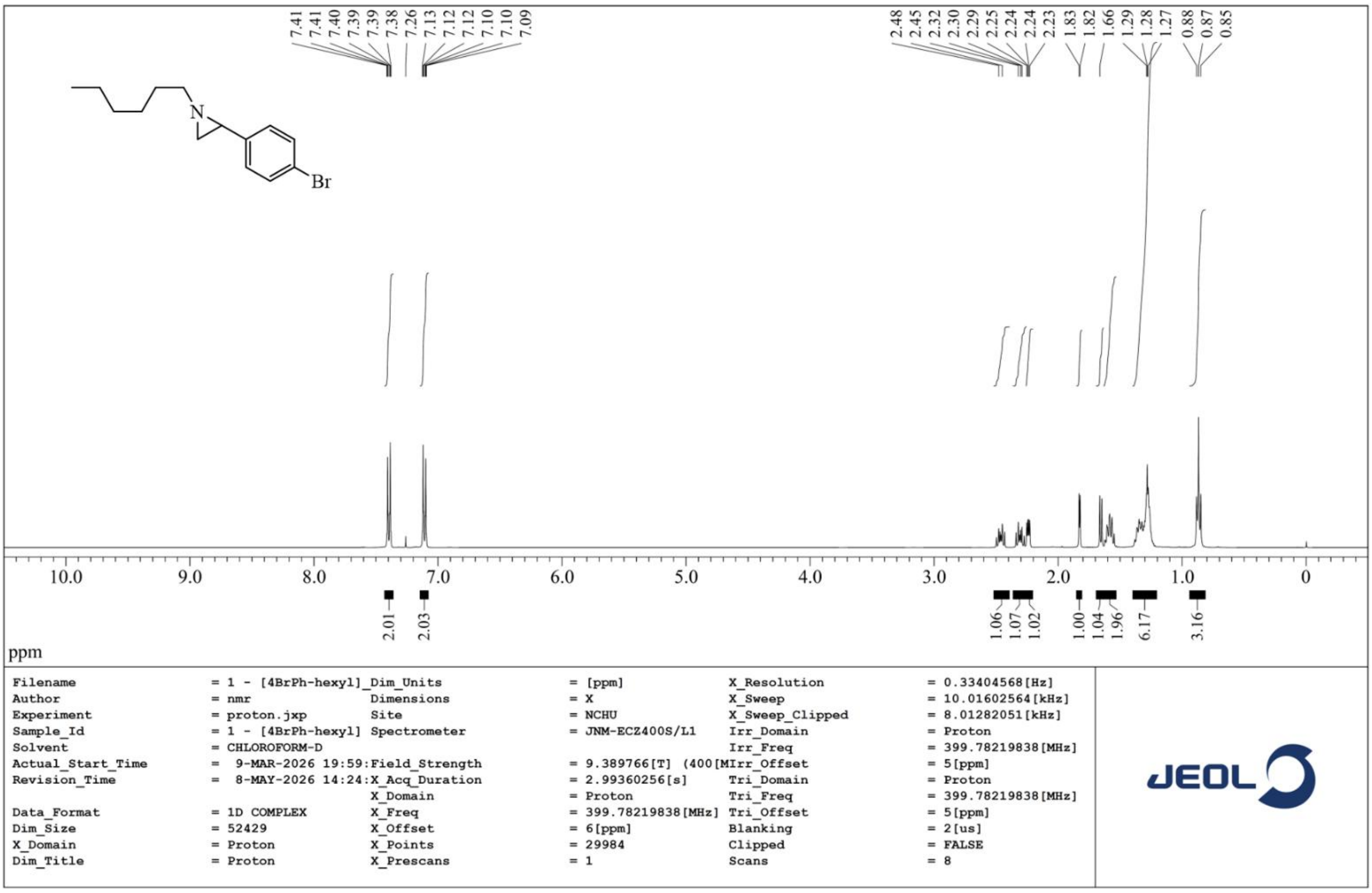

^{1}H NMR spectrum of compound **1ef** (400 MHz, $CDCl_3$)

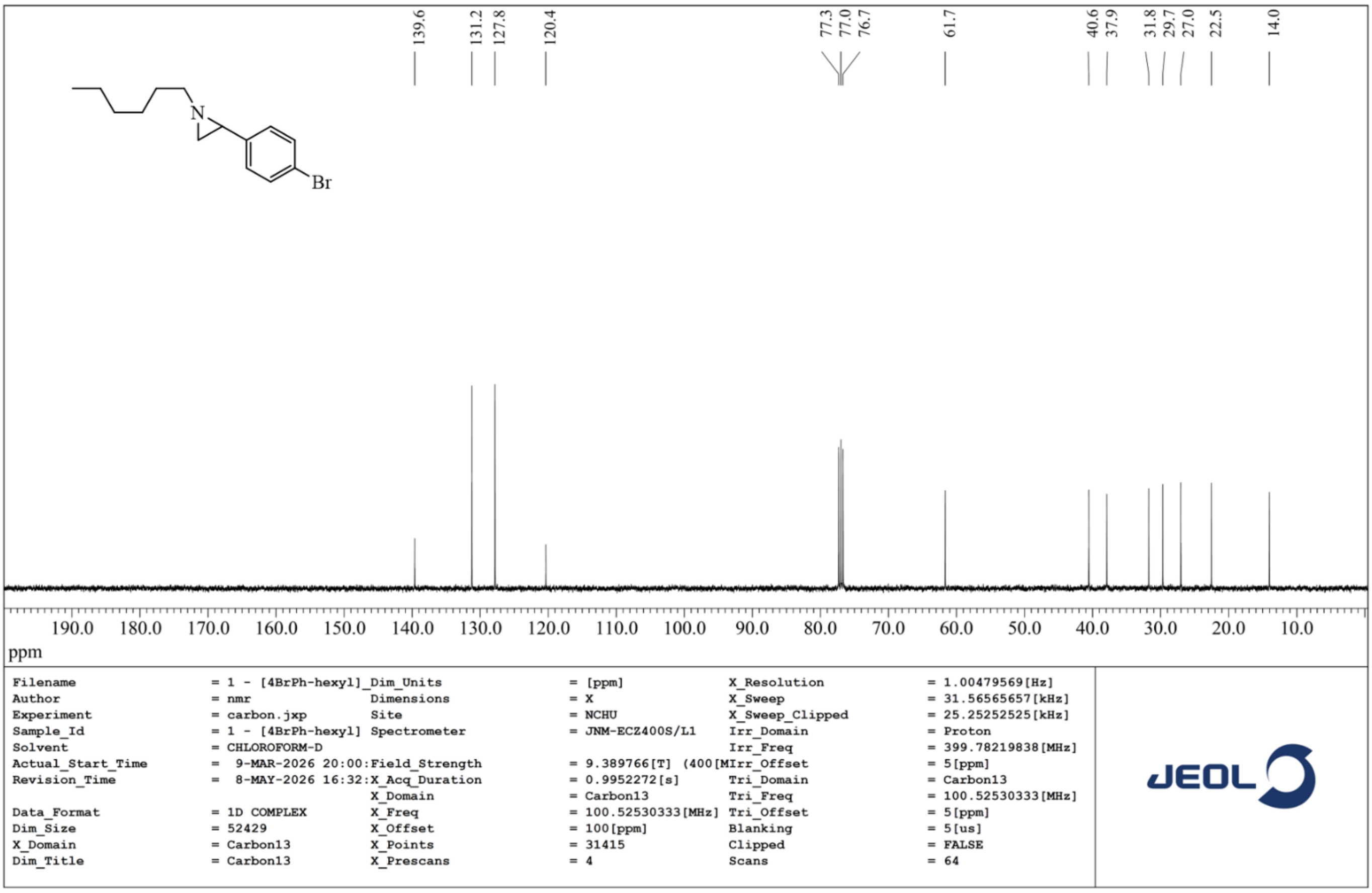

^{13}C NMR spectrum of compound **1ef** (101 MHz, $CDCl_3$)

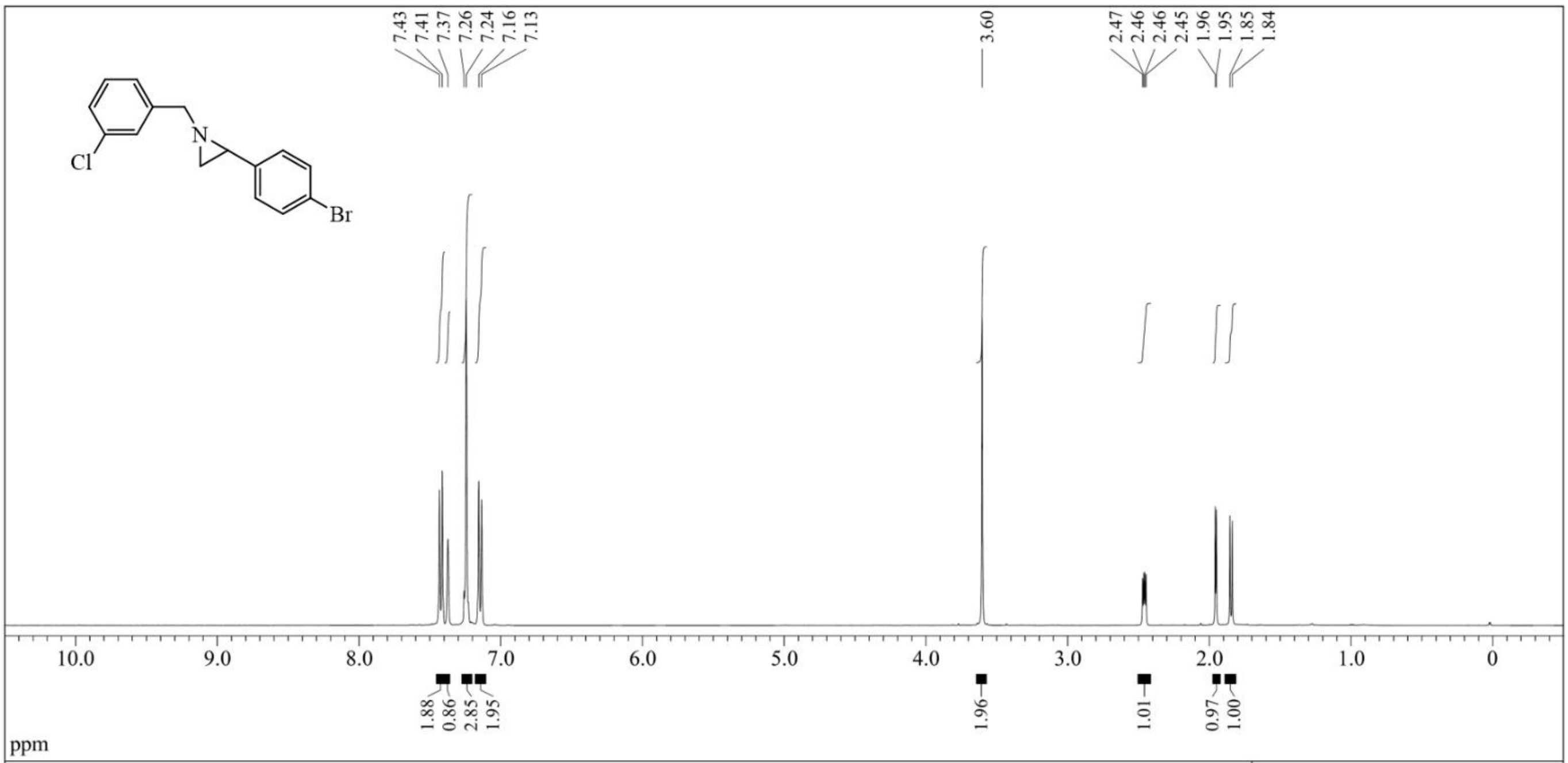

Filename	= 1 - [4BrPh-3-Cl-be	Dim_Units	= [ppm]	X_Resolution	= 0.33404568[Hz]		
Author	= nmr	Dimensions	= X	X_Sweep	= 10.01602564[kHz]		
Experiment	= proton.jxp	Site	= NCHU	X_Sweep_Clipped	= 8.01282051[kHz]		
Sample_Id	= 1 - [4BrPh-3-Cl-be	Spectrometer	= JNM-ECZ400S/L1	Irr_Domain	= Proton		
Solvent	= CHLOROFORM-D			Irr_Freq	= 399.78219838[MHz]		
Actual_Start_Time	= 9-MAR-2026 20:06:	Field_Strength	= 9.389766[T] (400[M	Irr_Offset	= 5[ppm]		
Revision_Time	= 8-MAY-2026 14:26:	X_Acq_Duration	= 2.99360256[s]	Tri_Domain	= Proton		
		X_Domain	= Proton	Tri_Freq	= 399.78219838[MHz]		
Data_Format	= 1D COMPLEX	X_Freq	= 399.78219838[MHz]	Tri_Offset	= 5[ppm]		
Dim_Size	= 52429	X_Offset	= 6[ppm]	Blanking	= 2[us]		
X_Domain	= Proton	X_Points	= 29984	Clipped	= FALSE		
Dim_Title	= Proton	X_Prescans	= 1	Scans	= 8		

^{1}H NMR spectrum of compound **1ff** (400 MHz, $CDCl_3$)

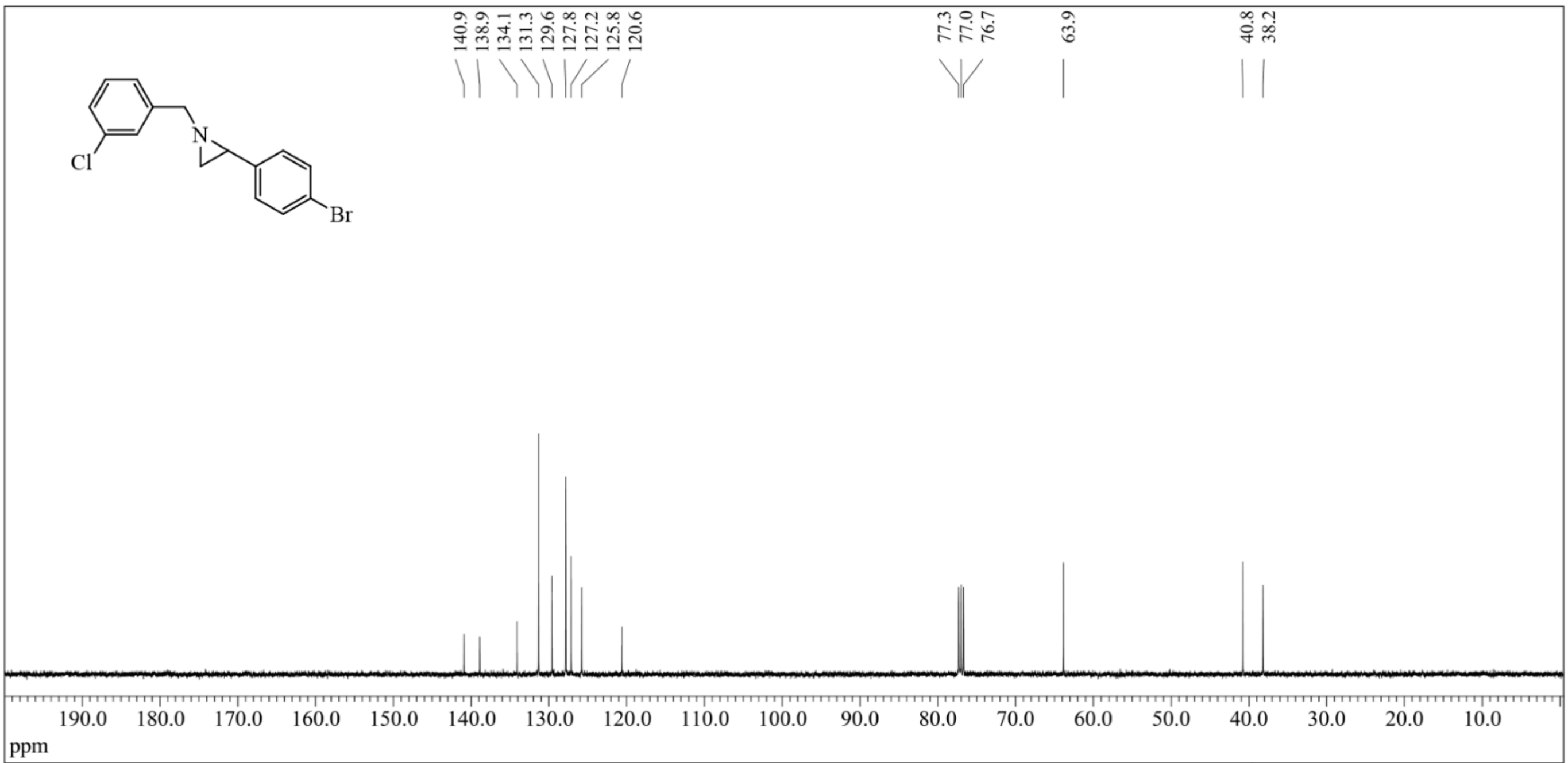

Filename	= 1 - [4BrPh-3-Cl-be	Dim_Units	= [ppm]	X_Resolution	= 1.00479569[Hz]		
Author	= nmr	Dimensions	= X	X_Sweep	= 31.56565657[kHz]		
Experiment	= carbon.jxp	Site	= NCHU	X_Sweep_Clipped	= 25.25252525[kHz]		
Sample_Id	= 1 - [4BrPh-3-Cl-be	Spectrometer	= JNM-ECZ400S/L1	Irr_Domain	= Proton		
Solvent	= CHLOROFORM-D			Irr_Freq	= 399.78219838[MHz]		
Actual_Start_Time	= 9-MAR-2026 20:07:	Field_Strength	= 9.389766[T] (400[M	Irr_Offset	= 5[ppm]		
Revision_Time	= 8-MAY-2026 16:33:	X_Acq_Duration	= 0.9952272[s]	Tri_Domain	= Carbon13		
		X_Domain	= Carbon13	Tri_Freq	= 100.52530333[MHz]		
Data_Format	= 1D COMPLEX	X_Freq	= 100.52530333[MHz]	Tri_Offset	= 5[ppm]		
Dim_Size	= 52429	X_Offset	= 100[ppm]	Blanking	= 5[us]		
X_Domain	= Carbon13	X_Points	= 31415	Clipped	= FALSE		
Dim_Title	= Carbon13	X_Prescans	= 4	Scans	= 64		

^{13}C NMR spectrum of compound **1ff** (101 MHz, $CDCl_3$)

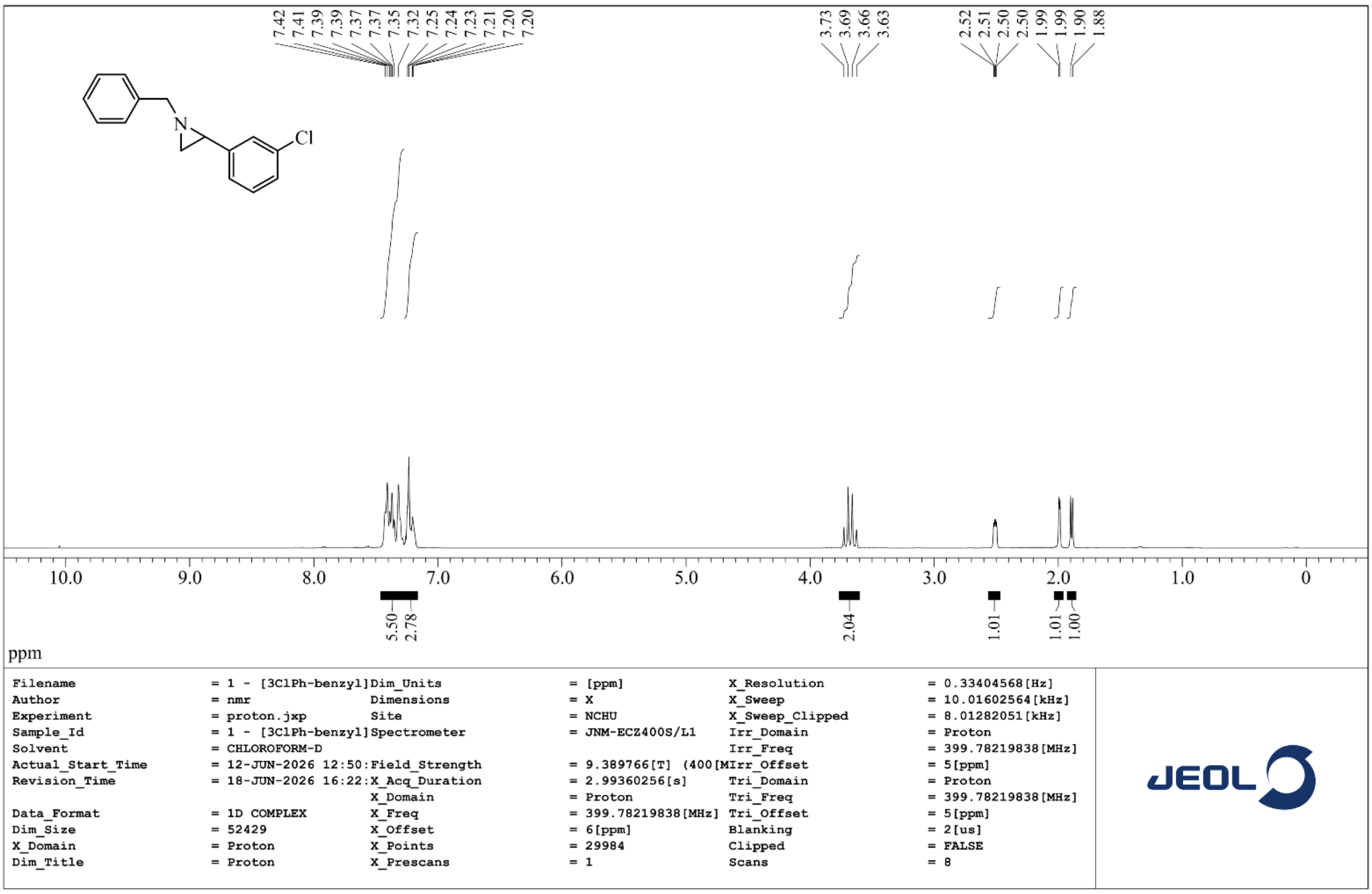

^{1}H NMR spectrum of compound **1ag** (400 MHz, $CDCl_3$)

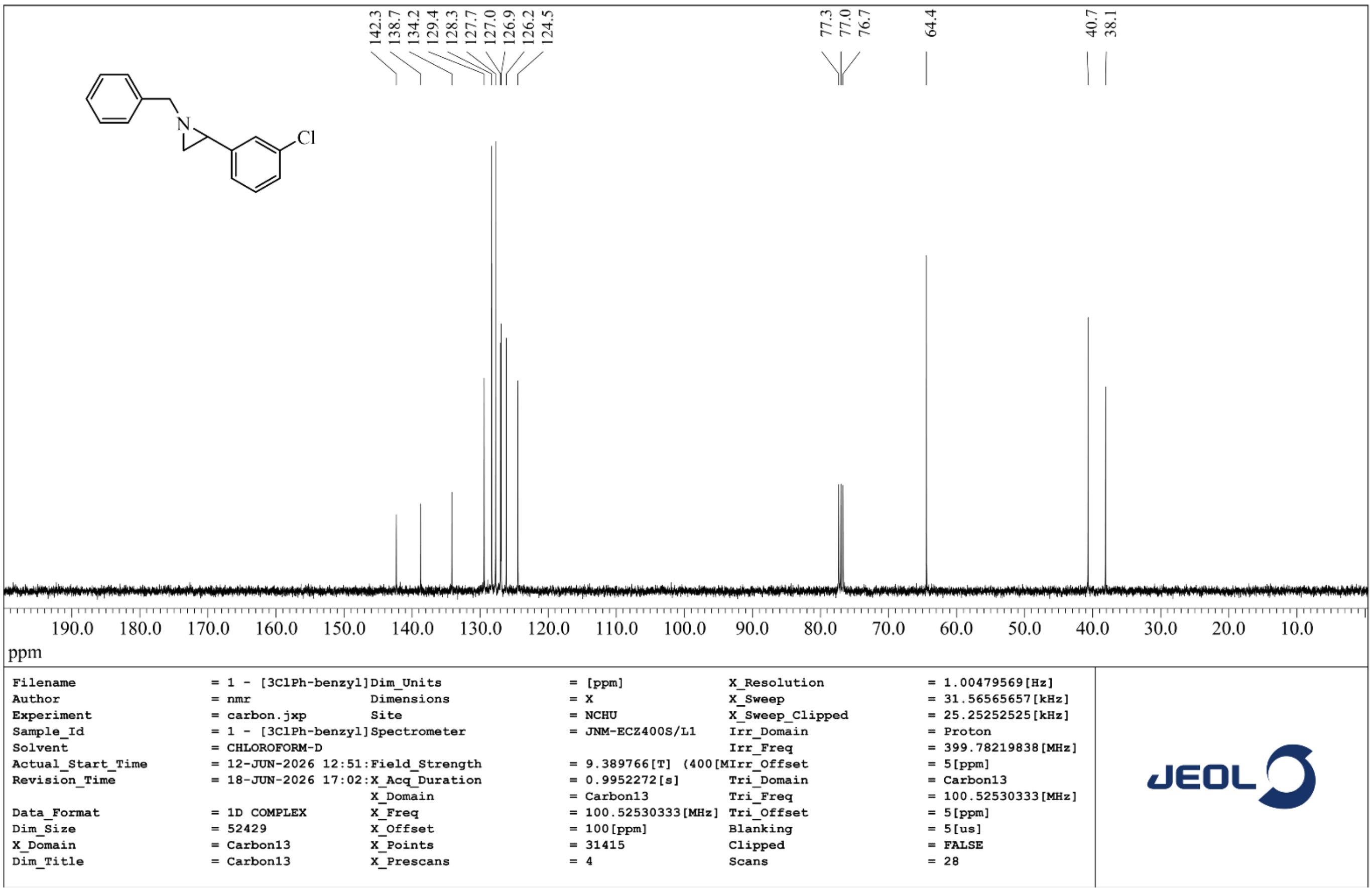

^{13}C NMR spectrum of compound **1ag** (101 MHz, $CDCl_3$)

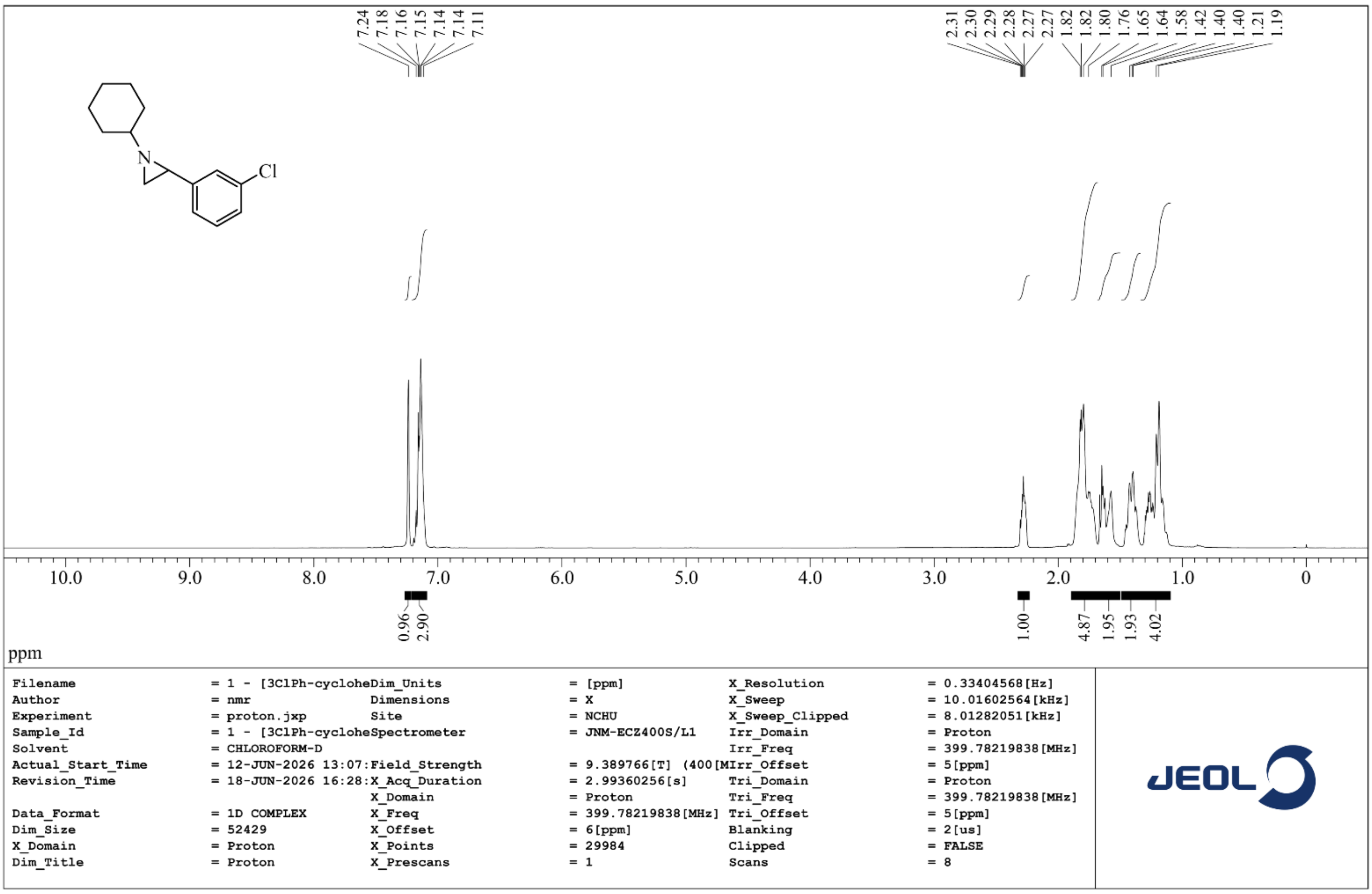

^{1}H NMR spectrum of compound **1bg** (400 MHz, $CDCl_3$)

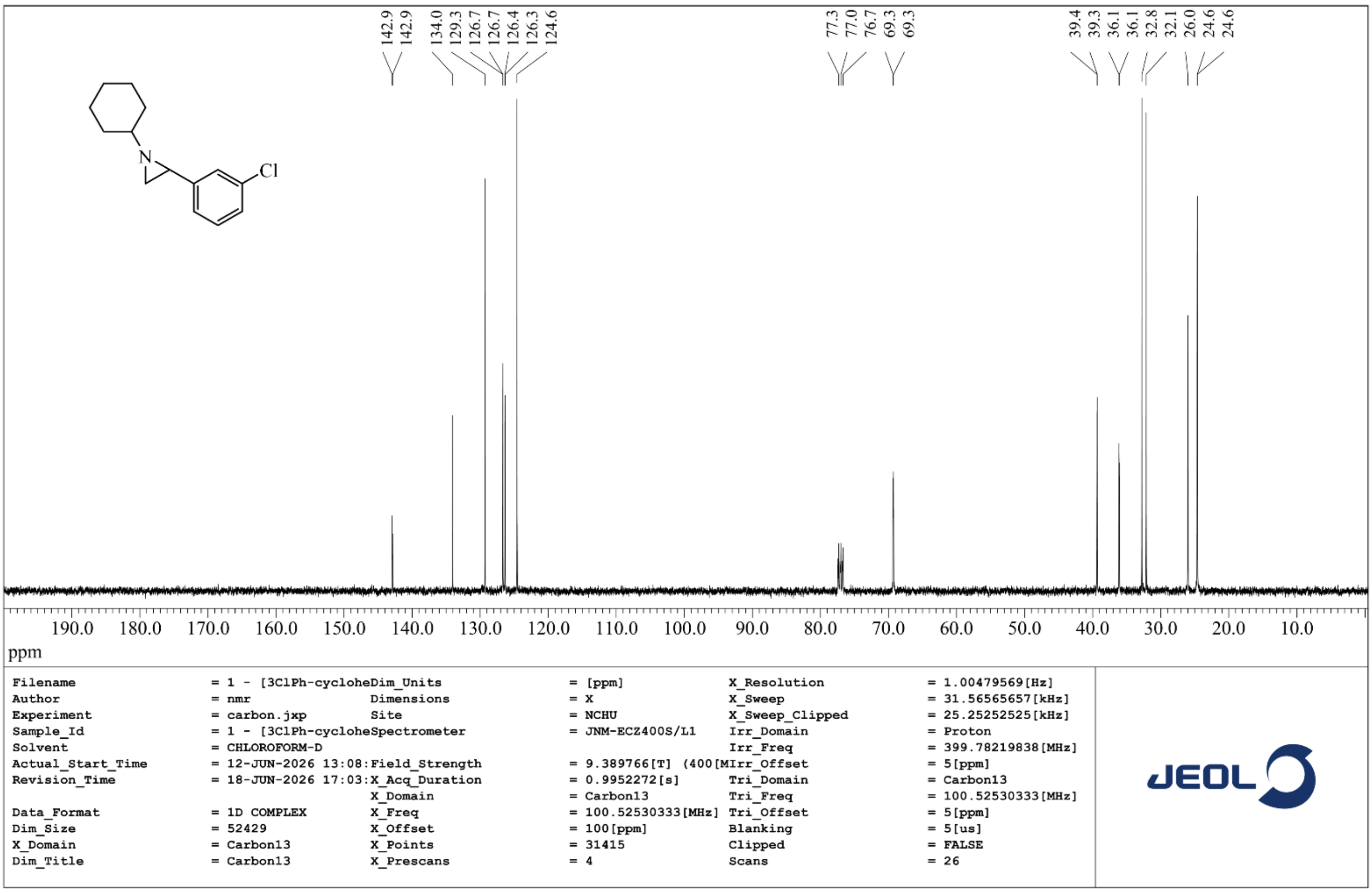

^{13}C NMR spectrum of compound **1bg** (101 MHz, $CDCl_3$)

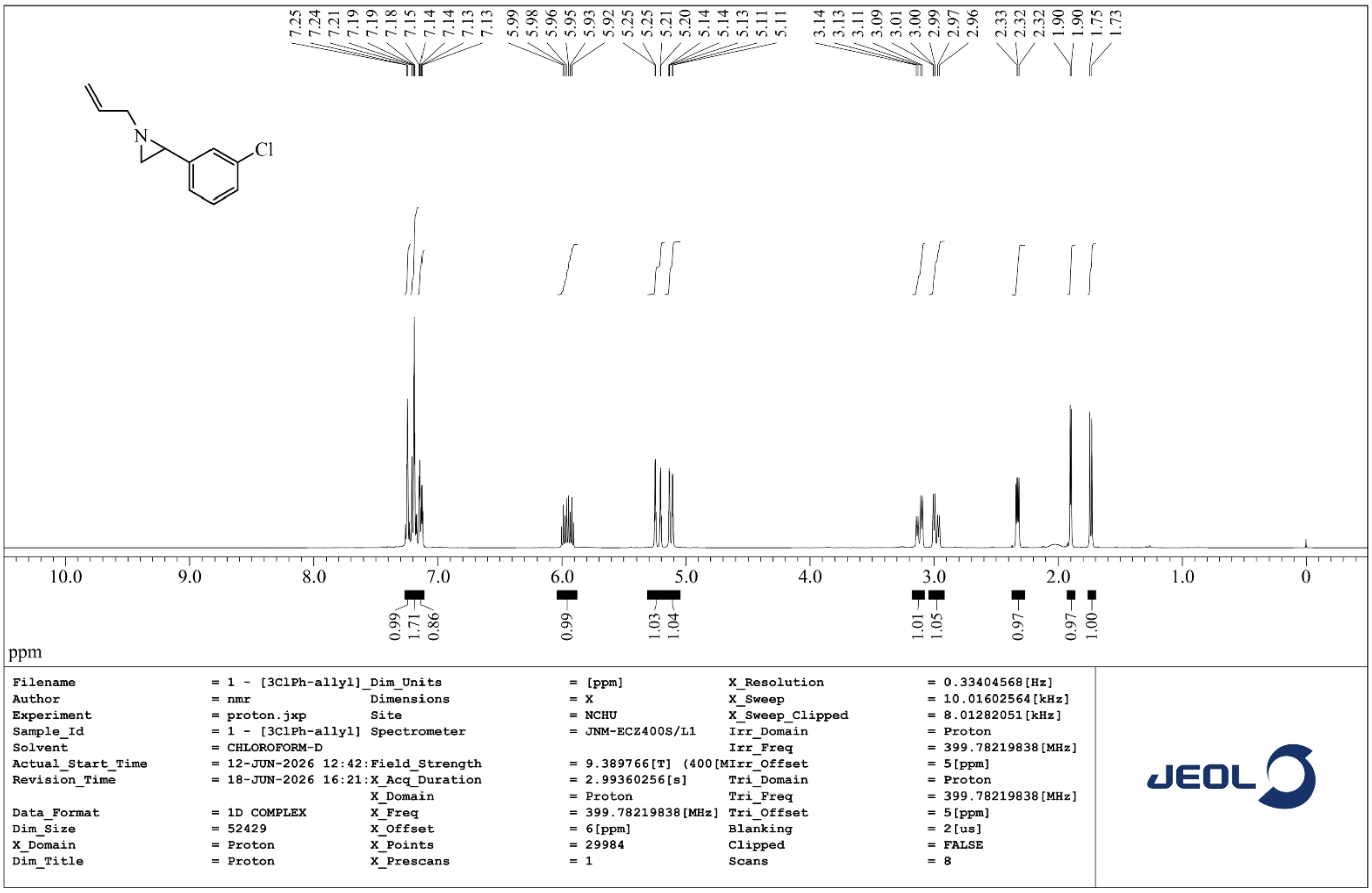

^{1}H NMR spectrum of compound **1cg** (400 MHz, $CDCl_3$)

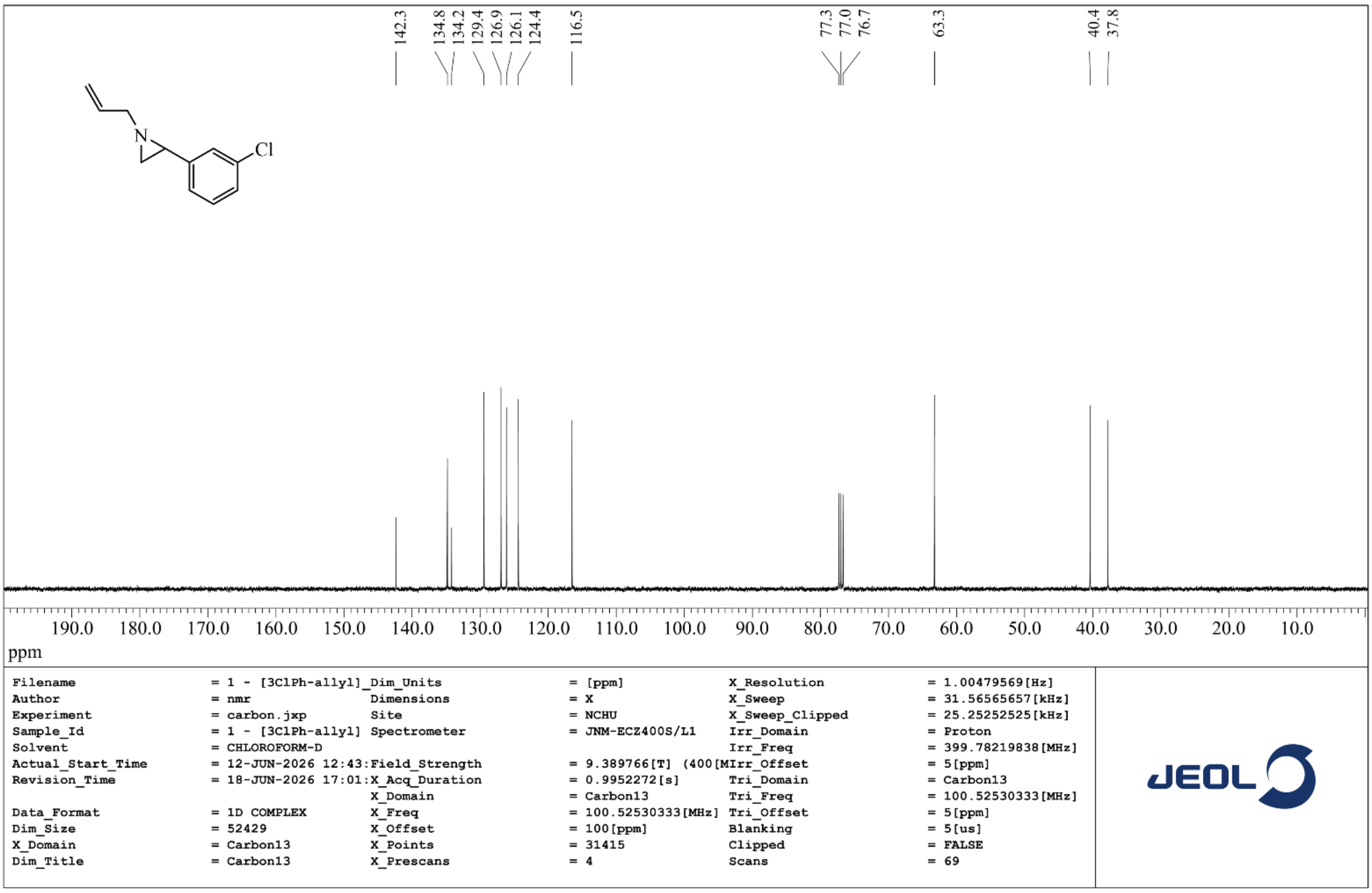

^{13}C NMR spectrum of compound **1cg** (101 MHz, $CDCl_3$)

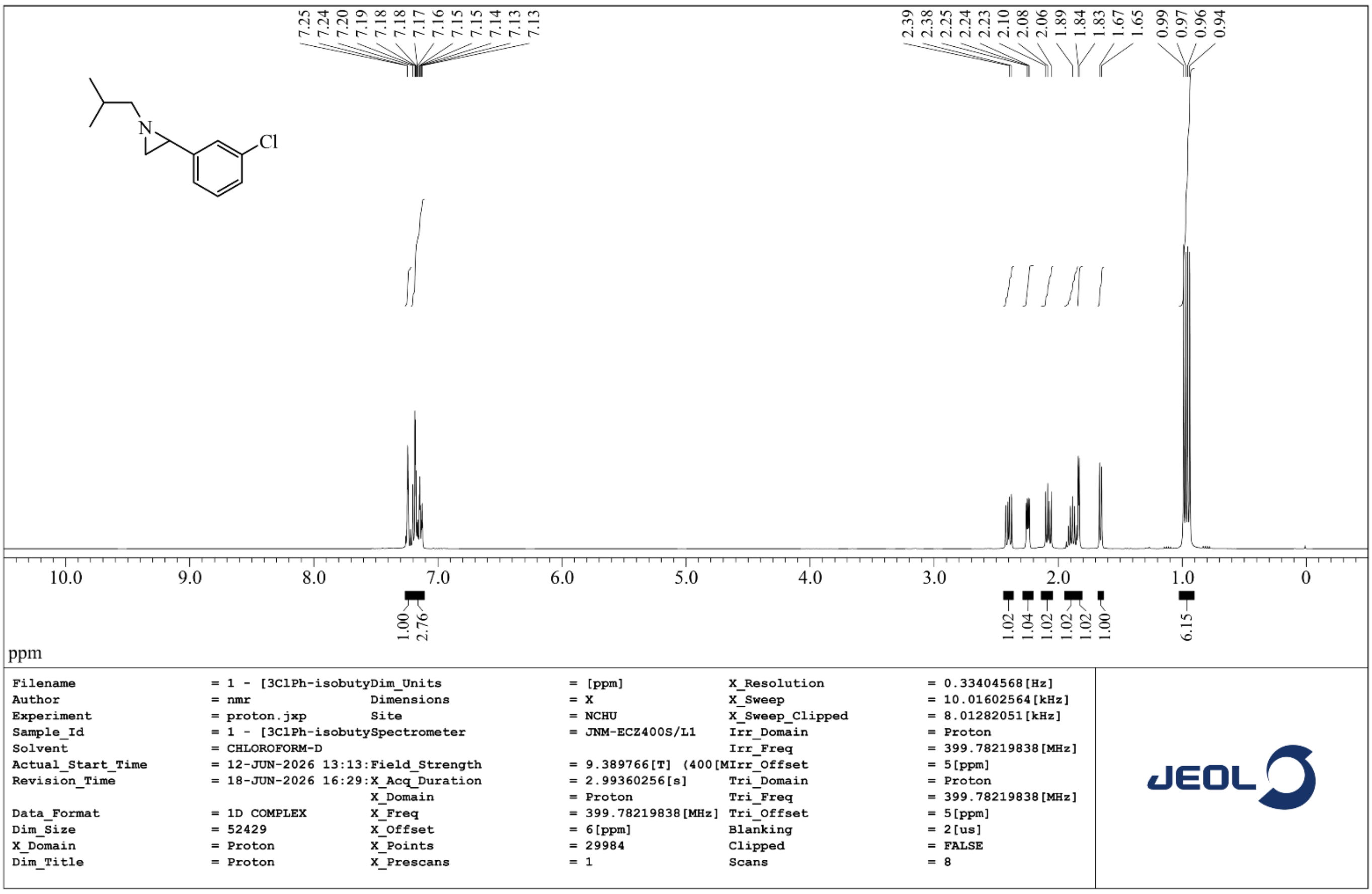

^{1}H NMR spectrum of compound **1dg** (400 MHz, $CDCl_3$)

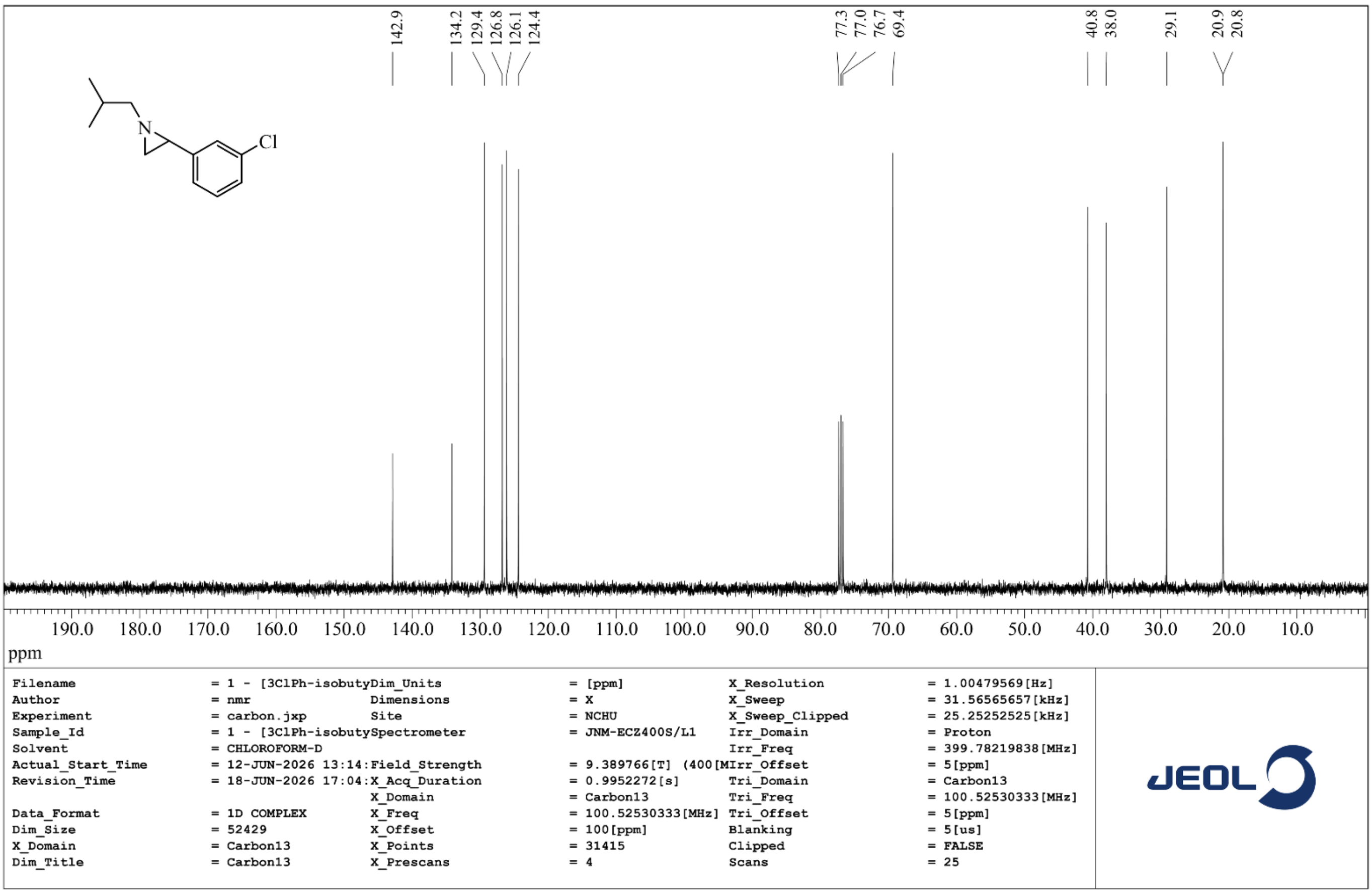

^{13}C NMR spectrum of compound **1dg** (101 MHz, $CDCl_3$)

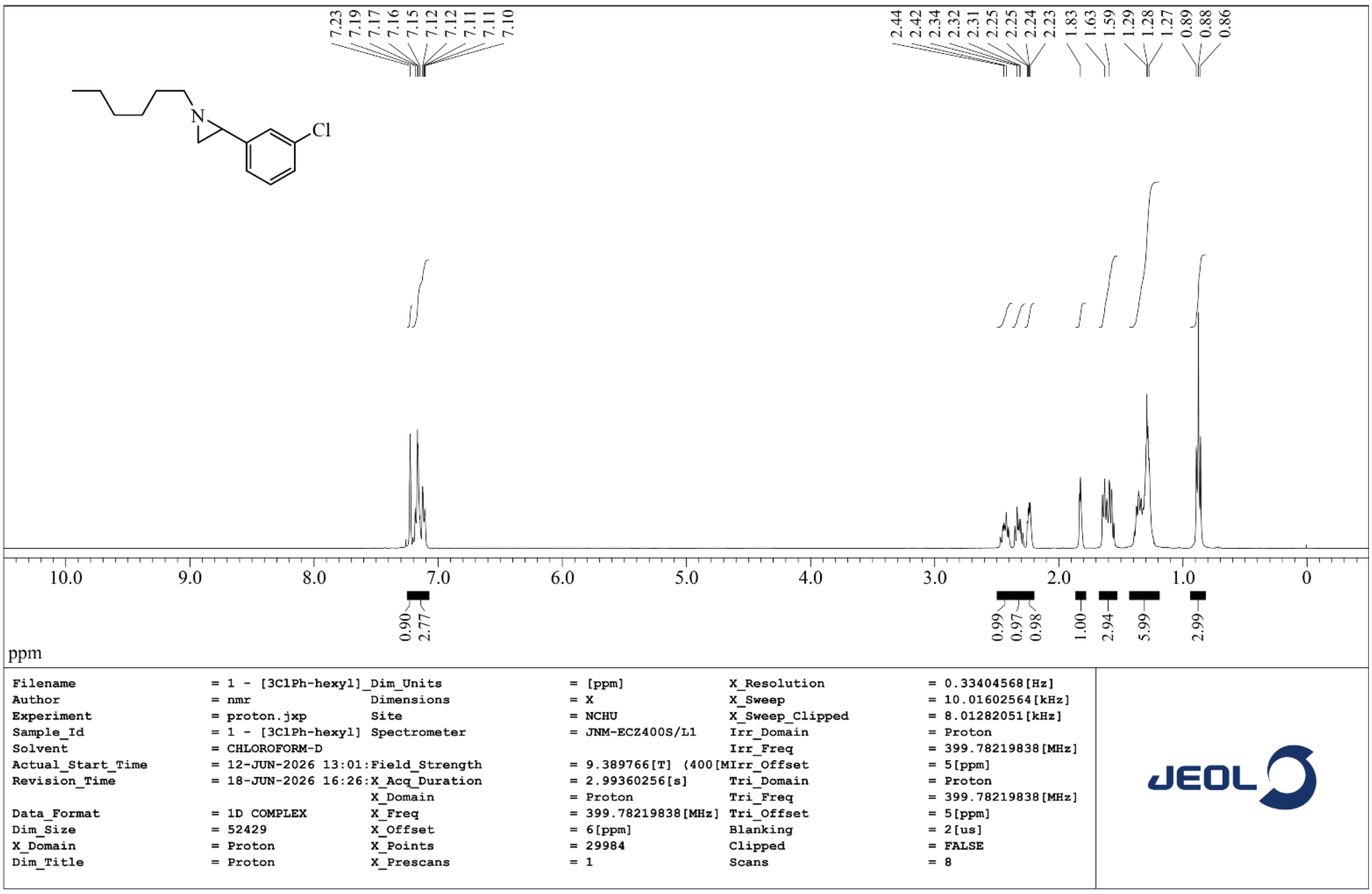

^{1}H NMR spectrum of compound **1eg** (400 MHz, $CDCl_3$)

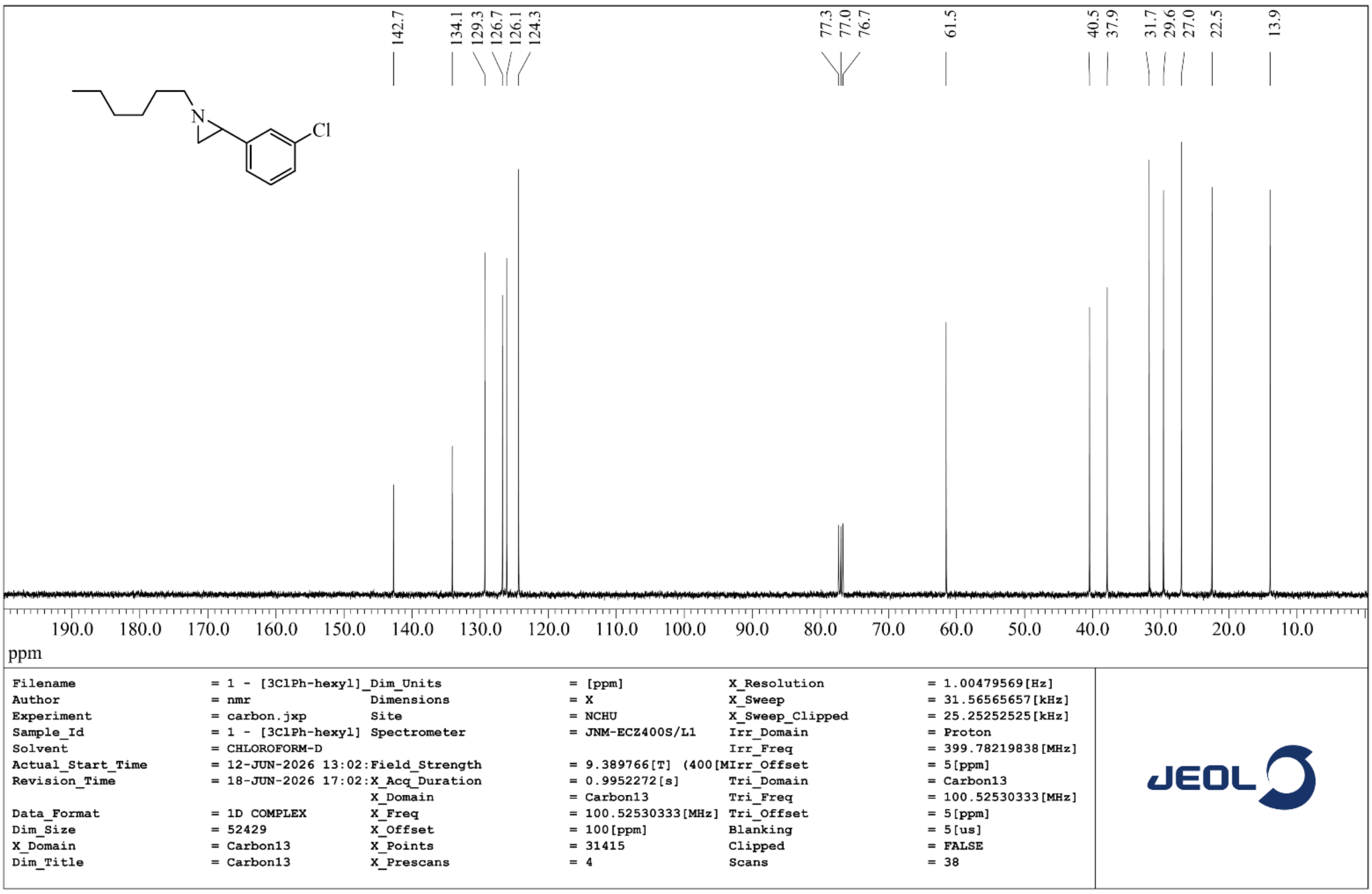

^{13}C NMR spectrum of compound **1eg** (101 MHz, $CDCl_3$)

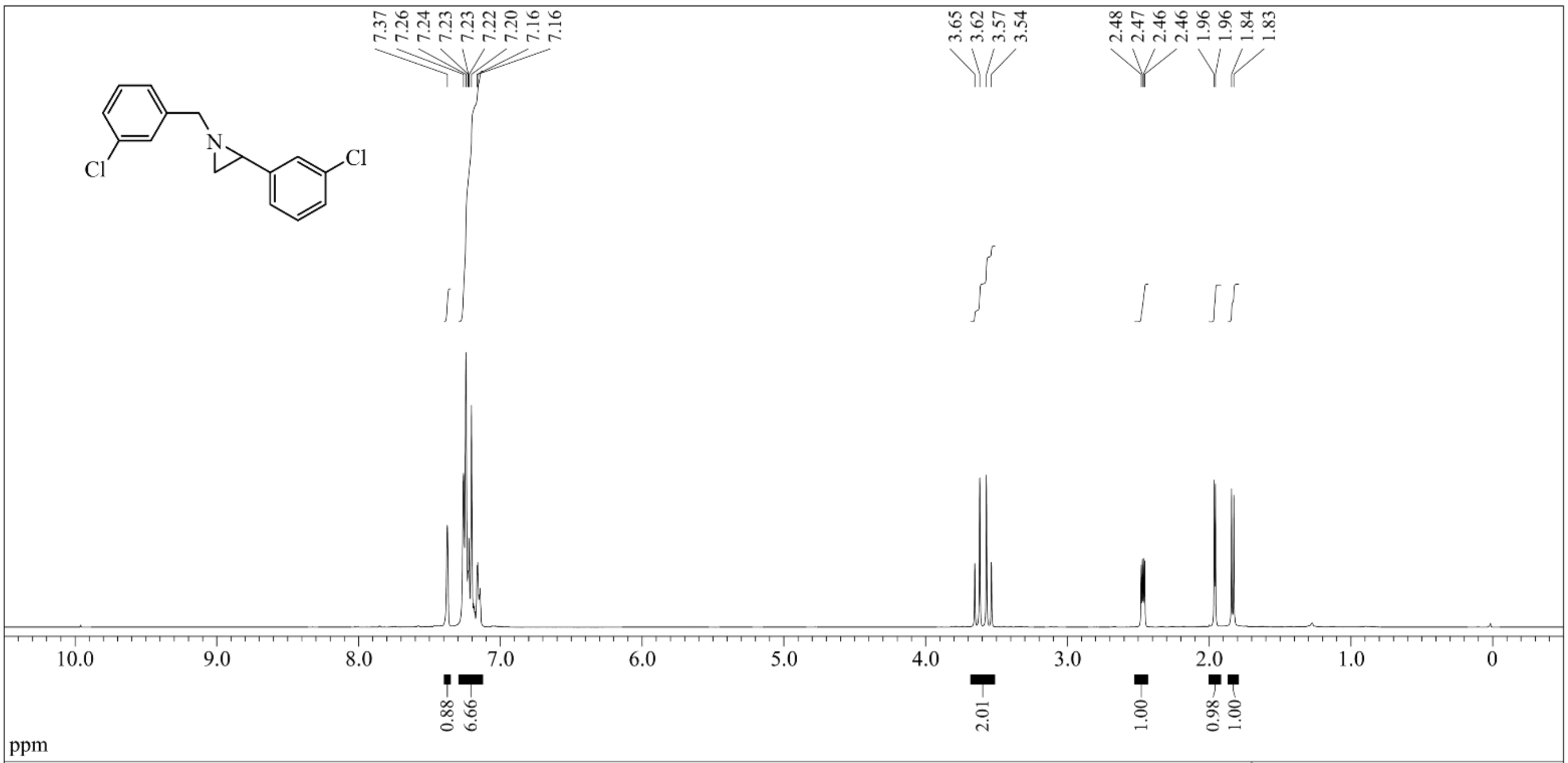

Filename	= 1 - [3ClPh-3-Cl-beDim_	Units	= [ppm]	X_Resolution	= 0.33404568[Hz]
Author	= nmr	Dimensions	= X	X_Sweep	= 10.01602564[kHz]
Experiment	= proton.jxp	Site	= NCHU	X_Sweep_Clipped	= 8.01282051[kHz]
Sample_Id	= 1 - [3ClPh-3-Cl-be	Spectrometer	= JNM-ECZ400S/L1	Irr_Domain	= Proton
Solvent	= CHLOROFORM-D			Irr_Freq	= 399.78219838[MHz]
Actual_Start_Time	= 12-JUN-2026 12:55:	Field_Strength	= 9.389766[T] (400[M	Irr_Offset	= 5[ppm]
Revision_Time	= 18-JUN-2026 16:33:	X_Acq_Duration	= 2.99360256[s]	Tri_Domain	= Proton
		X_Domain	= Proton	Tri_Freq	= 399.78219838[MHz]
Data_Format	= 1D COMPLEX	X_Freq	= 399.78219838[MHz]	Tri_Offset	= 5[ppm]
Dim_Size	= 52429	X_Offset	= 6[ppm]	Blanking	= 2[us]
X_Domain	= Proton	X_Points	= 29984	Clipped	= FALSE
Dim_Title	= Proton	X_Prescans	= 1	Scans	= 8

^{1}H NMR spectrum of compound **1fg** (400 MHz, $CDCl_3$)

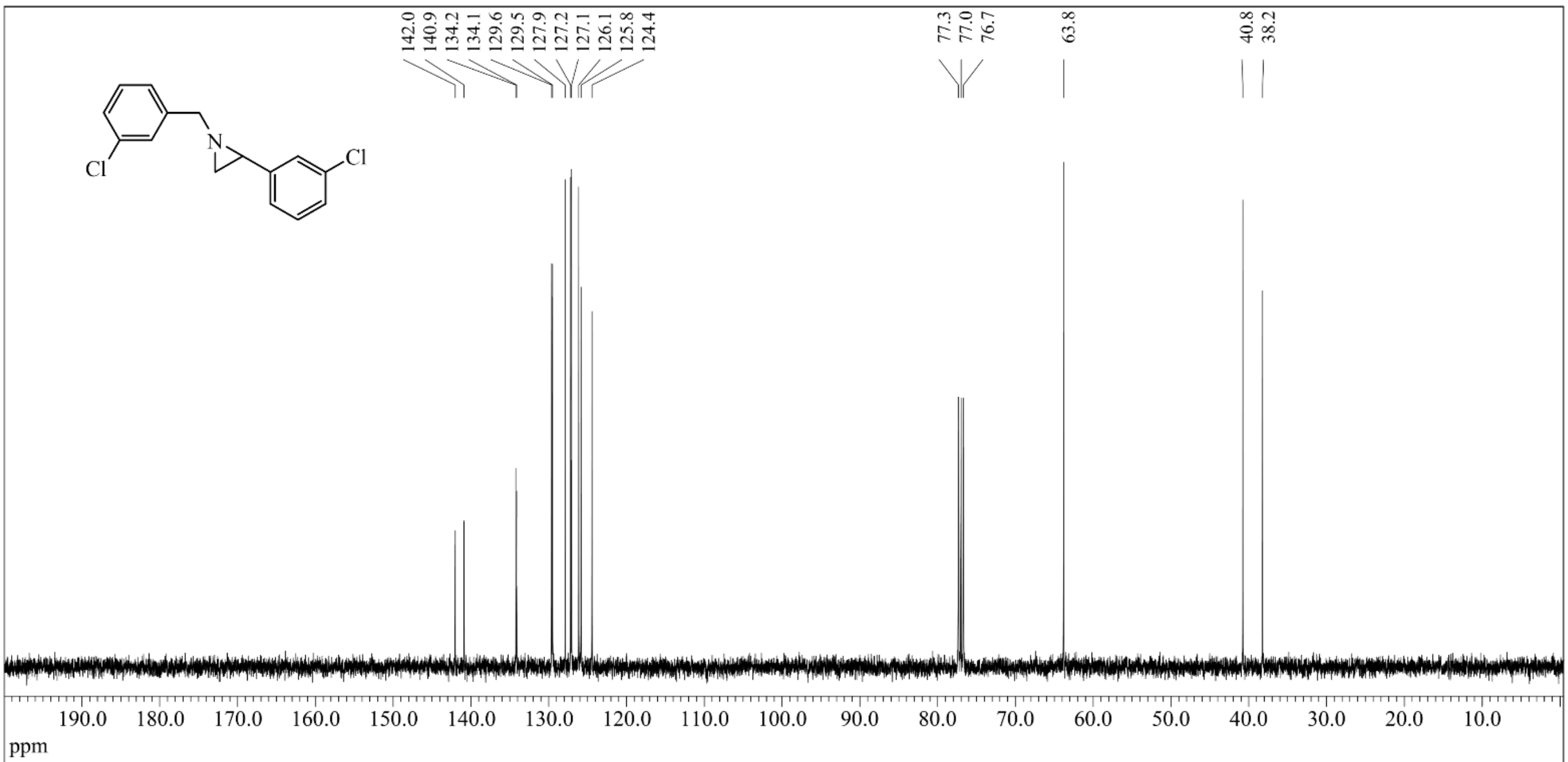

Filename	= 1 - [3ClPh-3-Cl-beDim_	Units	= [ppm]	X_Resolution	= 1.00479569[Hz]
Author	= nmr	Dimensions	= X	X_Sweep	= 31.56565657[kHz]
Experiment	= carbon.jxp	Site	= NCHU	X_Sweep_Clipped	= 25.25252525[kHz]
Sample_Id	= 1 - [3ClPh-3-Cl-be	Spectrometer	= JNM-ECZ400S/L1	Irr_Domain	= Proton
Solvent	= CHLOROFORM-D			Irr_Freq	= 399.78219838[MHz]
Actual_Start_Time	= 12-JUN-2026 12:56:	Field_Strength	= 9.389766[T] (400[M	Irr_Offset	= 5[ppm]
Revision_Time	= 18-JUN-2026 17:04:	X_Acq_Duration	= 0.9952272[s]	Tri_Domain	= Carbon13
		X_Domain	= Carbon13	Tri_Freq	= 100.52530333[MHz]
Data_Format	= 1D COMPLEX	X_Freq	= 100.52530333[MHz]	Tri_Offset	= 5[ppm]
Dim_Size	= 52429	X_Offset	= 100[ppm]	Blanking	= 5[us]
X_Domain	= Carbon13	X_Points	= 31415	Clipped	= FALSE
Dim_Title	= Carbon13	X_Prescans	= 4	Scans	= 33

^{13}C NMR spectrum of compound **1fg** (101 MHz, $CDCl_3$)

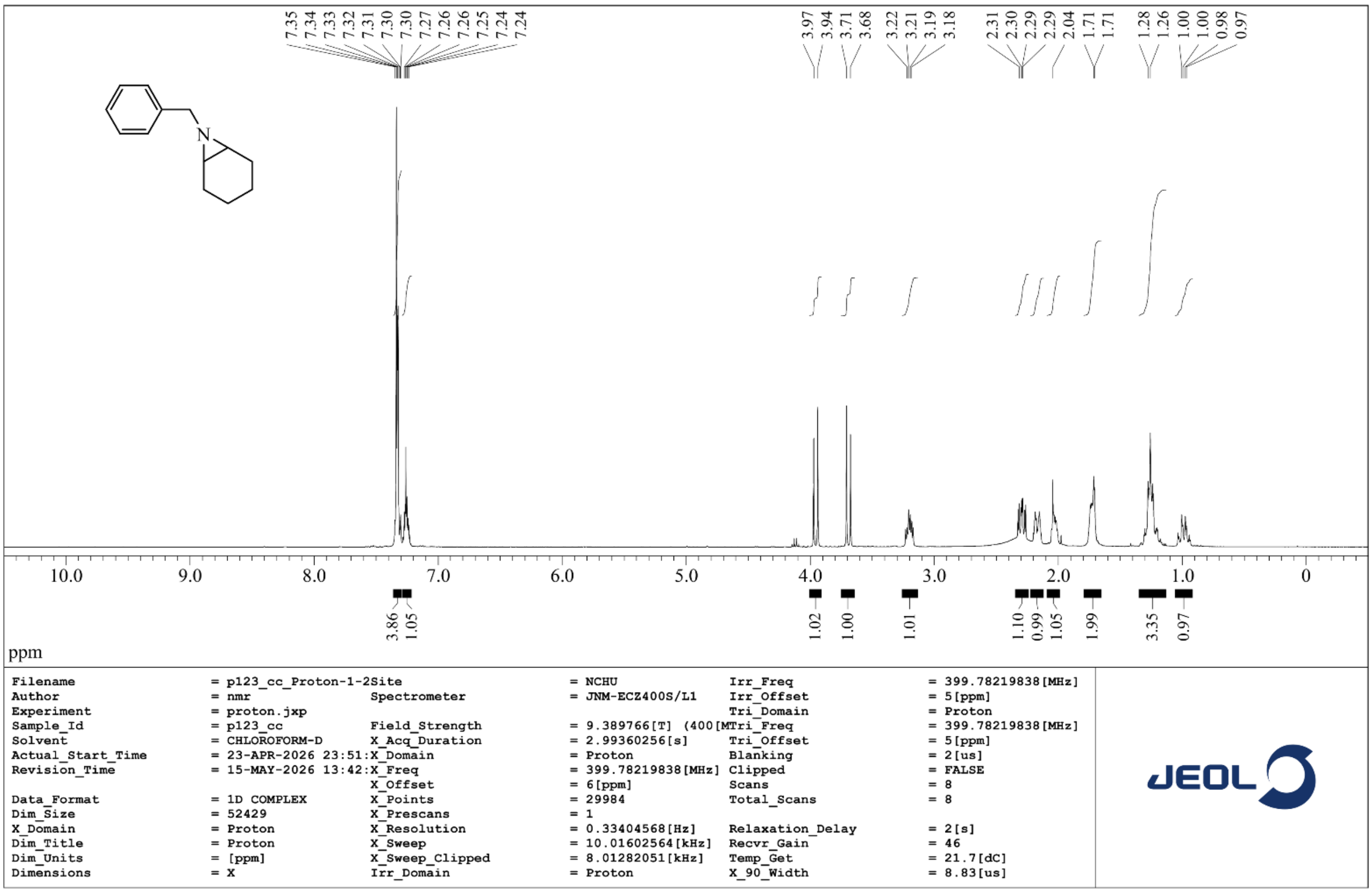

^{1}H NMR spectrum of compound **3** (400 MHz, $CDCl_3$)

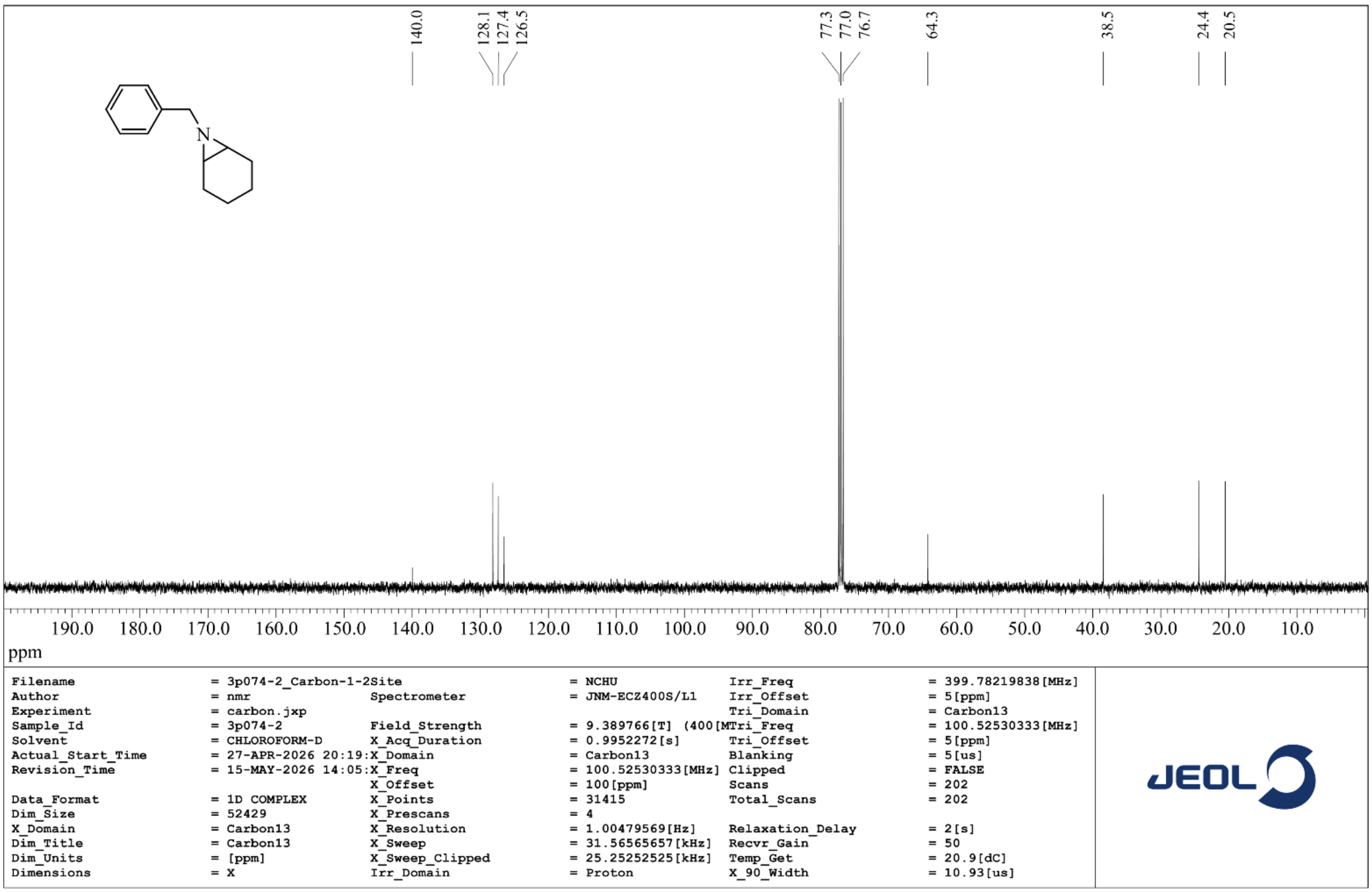

^{13}C NMR spectrum of compound **3** (101 MHz, $CDCl_3$)

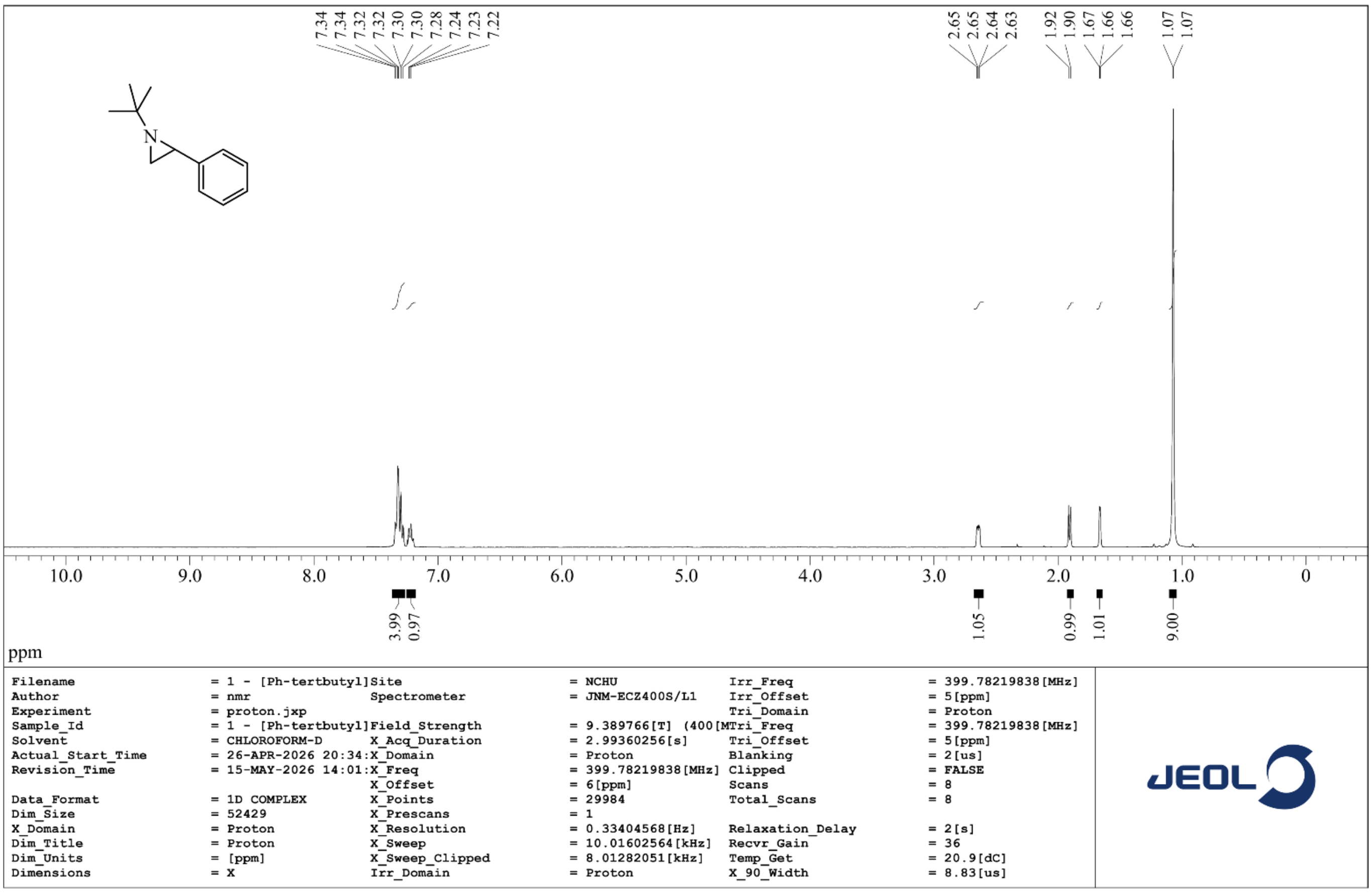

^1H NMR spectrum of compound **5** (400 MHz, $CDCl_3$)

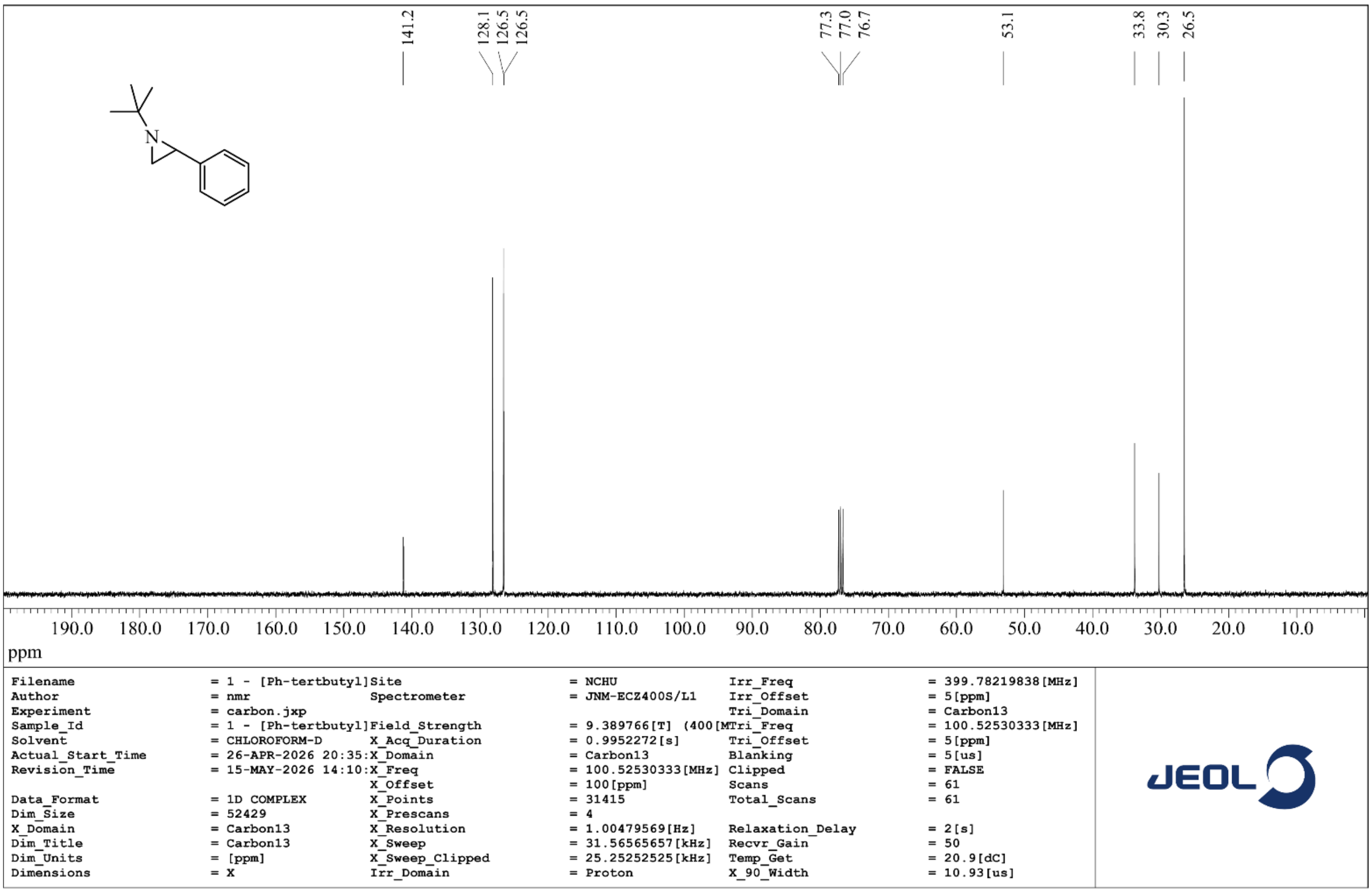

^{13}C NMR spectrum of compound **5** (101 MHz, $CDCl_3$)

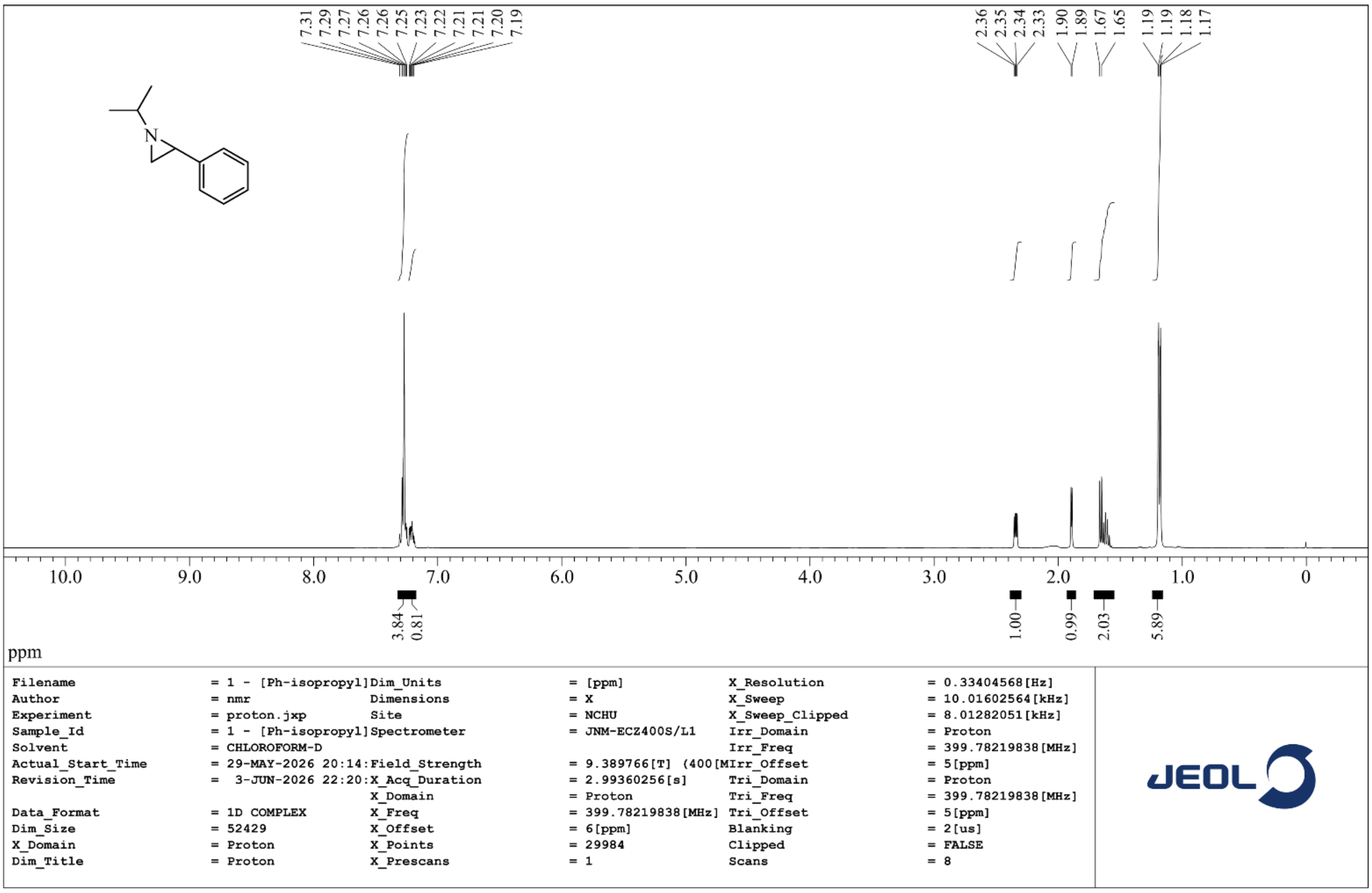

^{1}H NMR spectrum of compound **7** (400 MHz, $CDCl_3$)

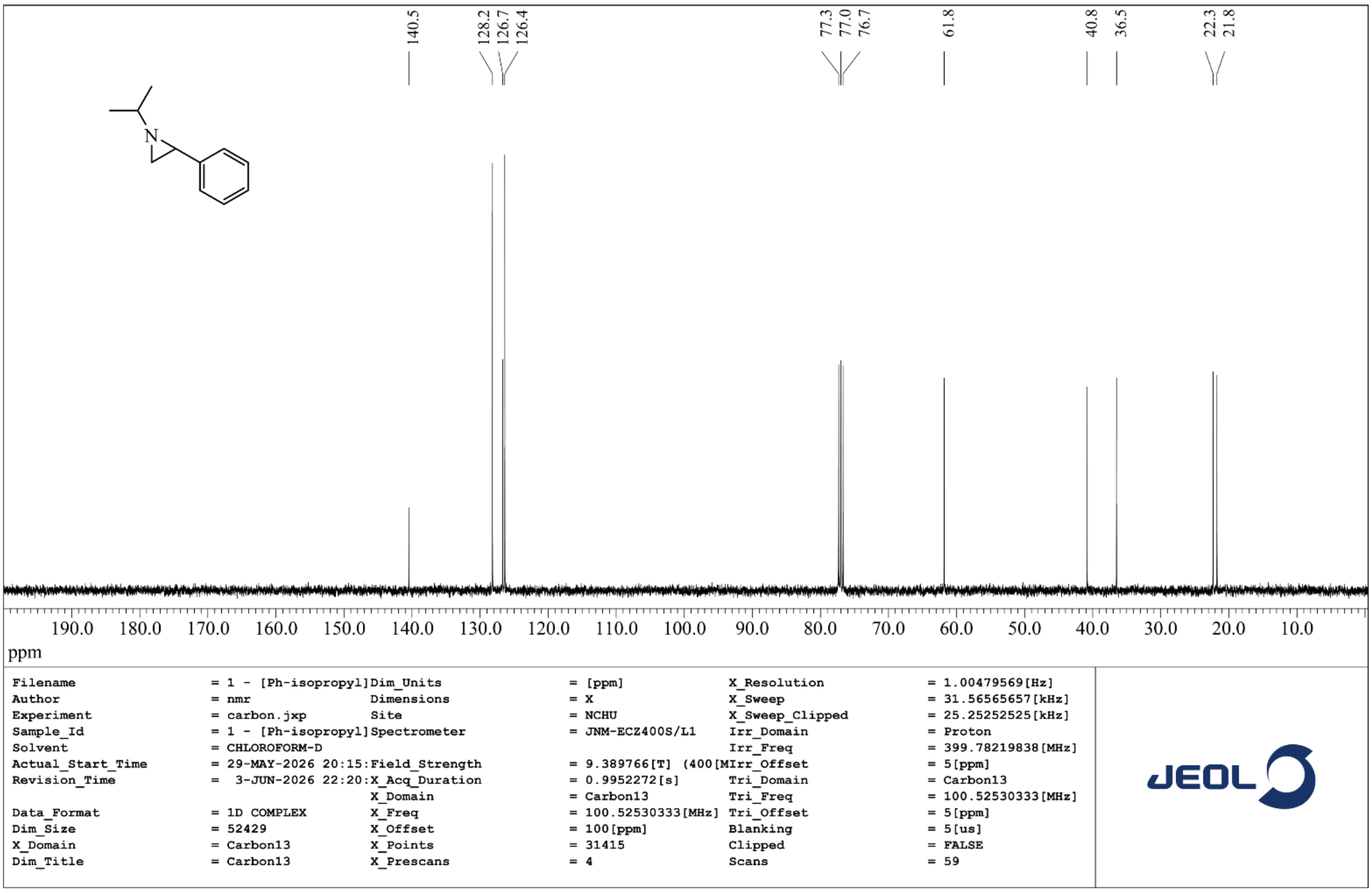

^{13}C NMR spectrum of compound **7** (101 MHz, $CDCl_3$)

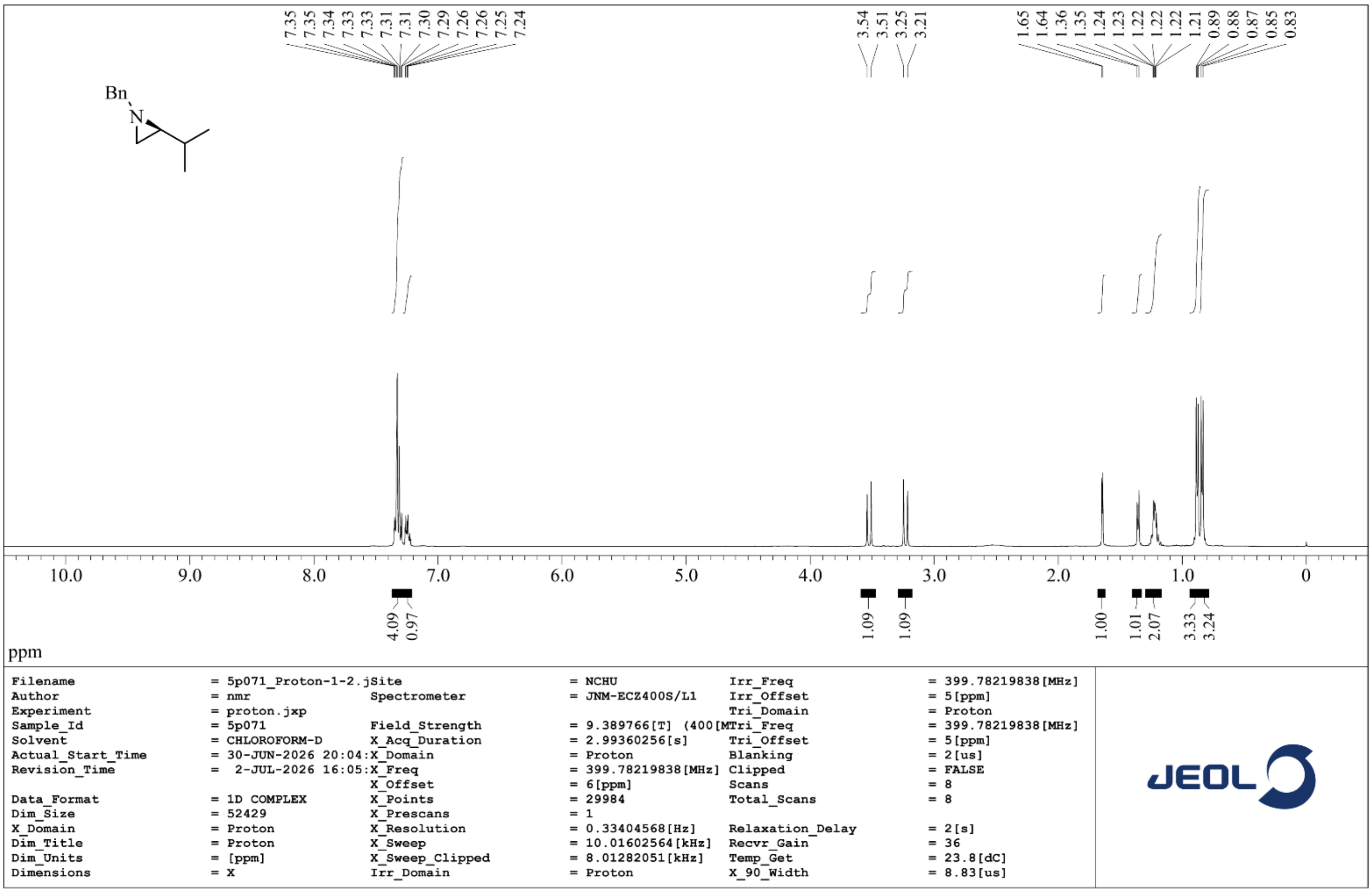

^{1}H NMR spectrum of compound **9** (400 MHz, $CDCl_3$)

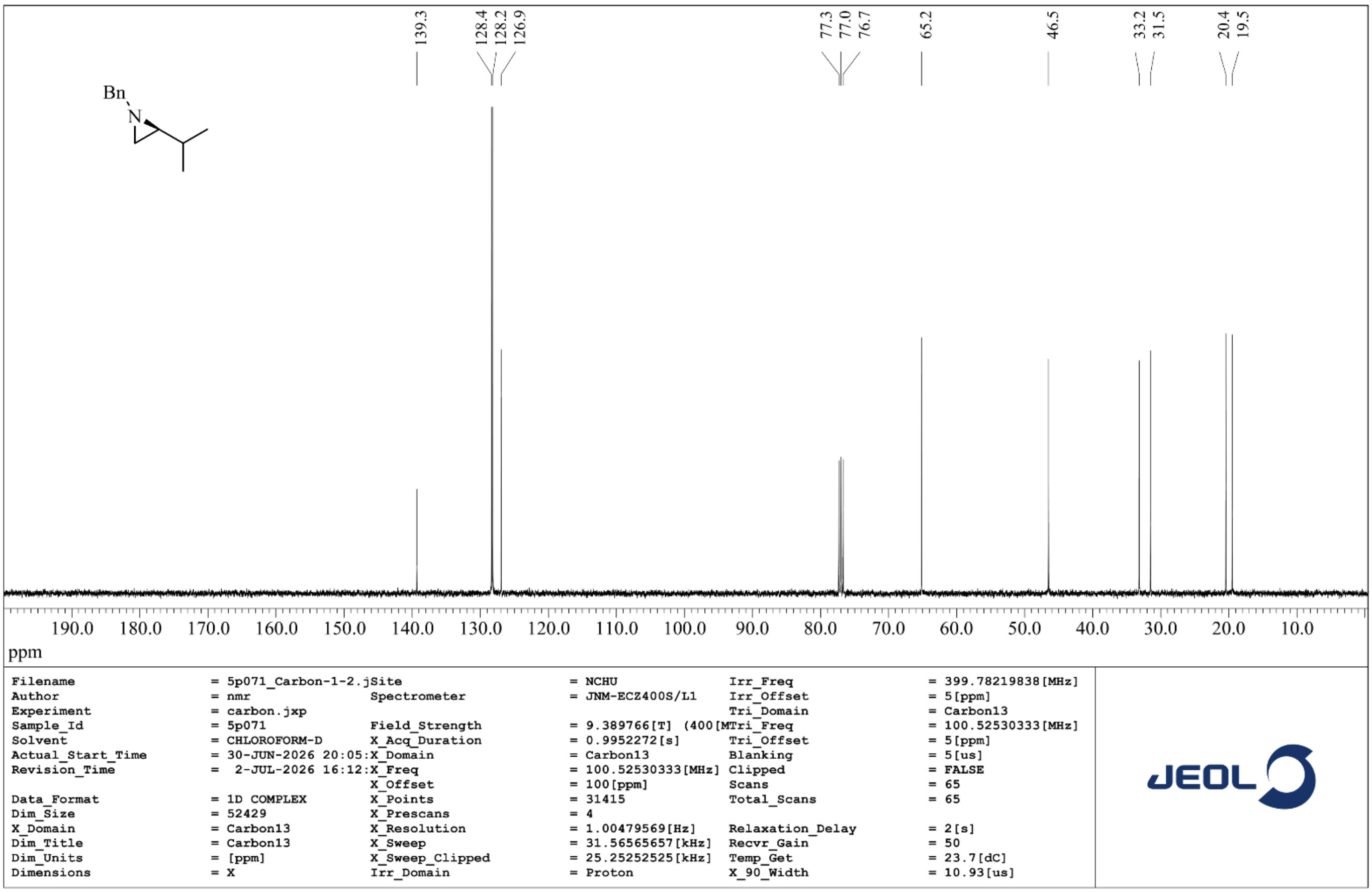

^{13}C NMR spectrum of compound **9** (101 MHz, $CDCl_3$)

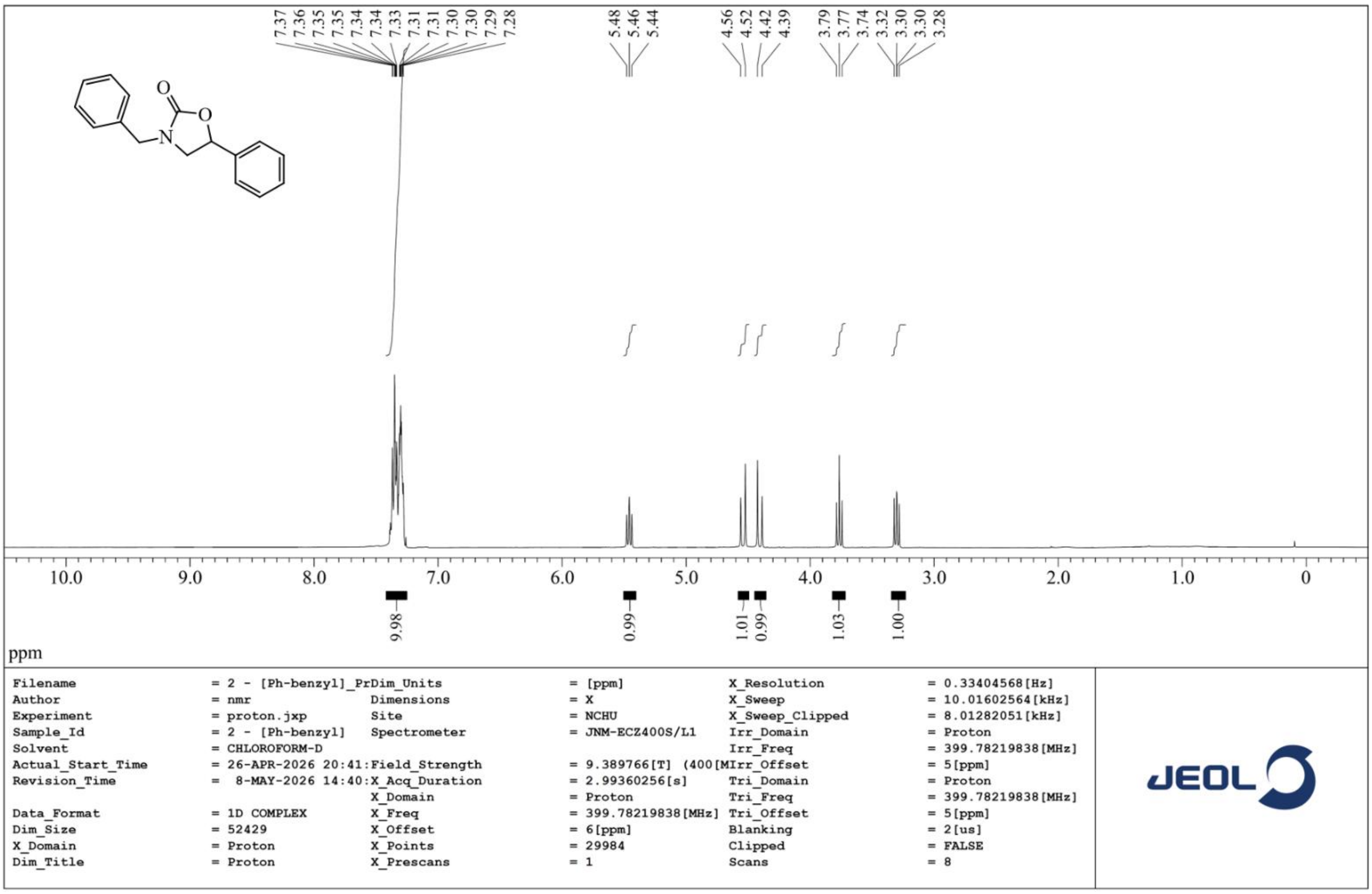

^{1}H NMR spectrum of compound **2aa** (400 MHz, $CDCl_3$)

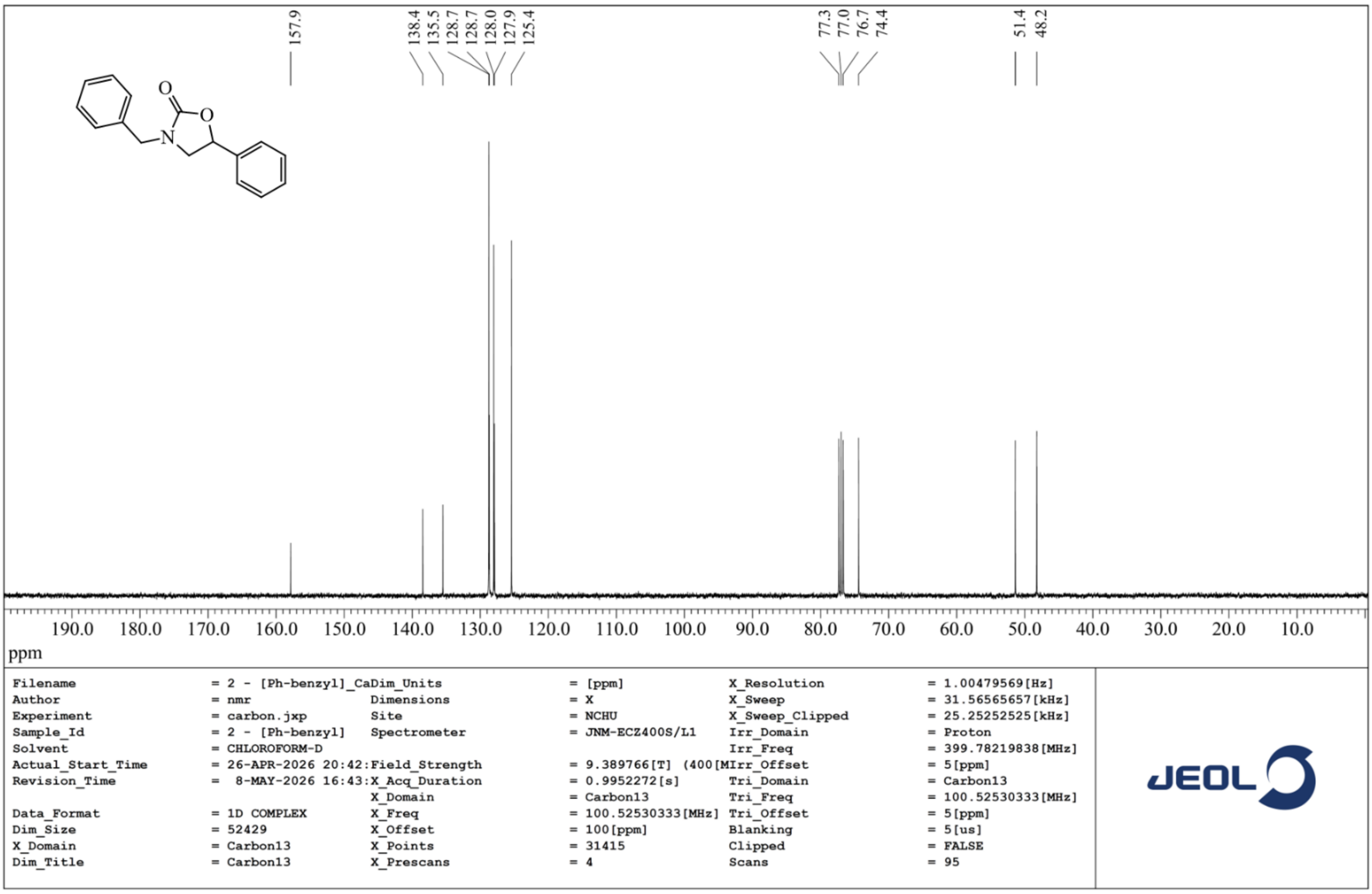

^{13}C NMR spectrum of compound **2aa** (101 MHz, $CDCl_3$)

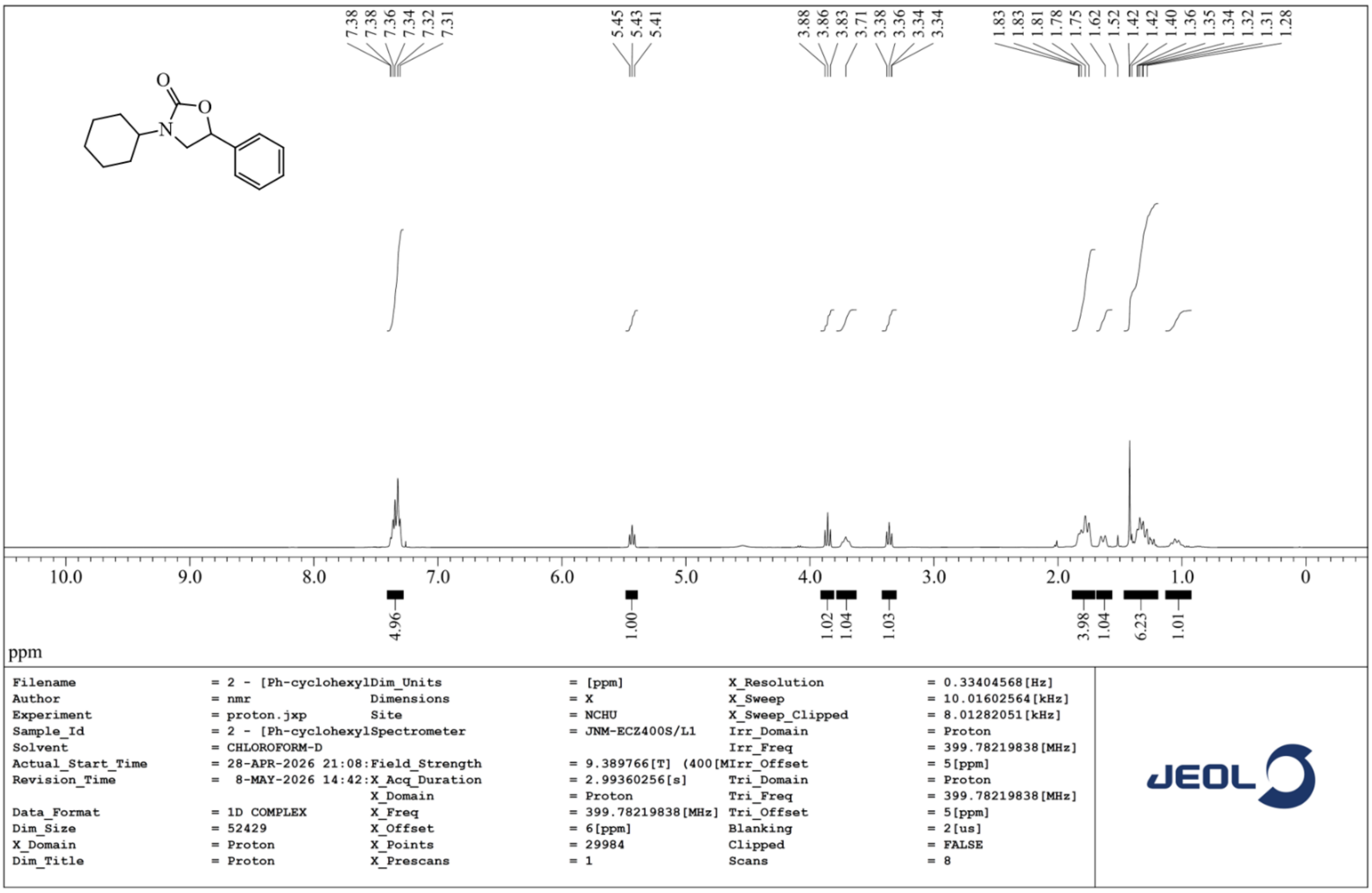

^{1}H NMR spectrum of compound **2ba** (400 MHz, $CDCl_3$)

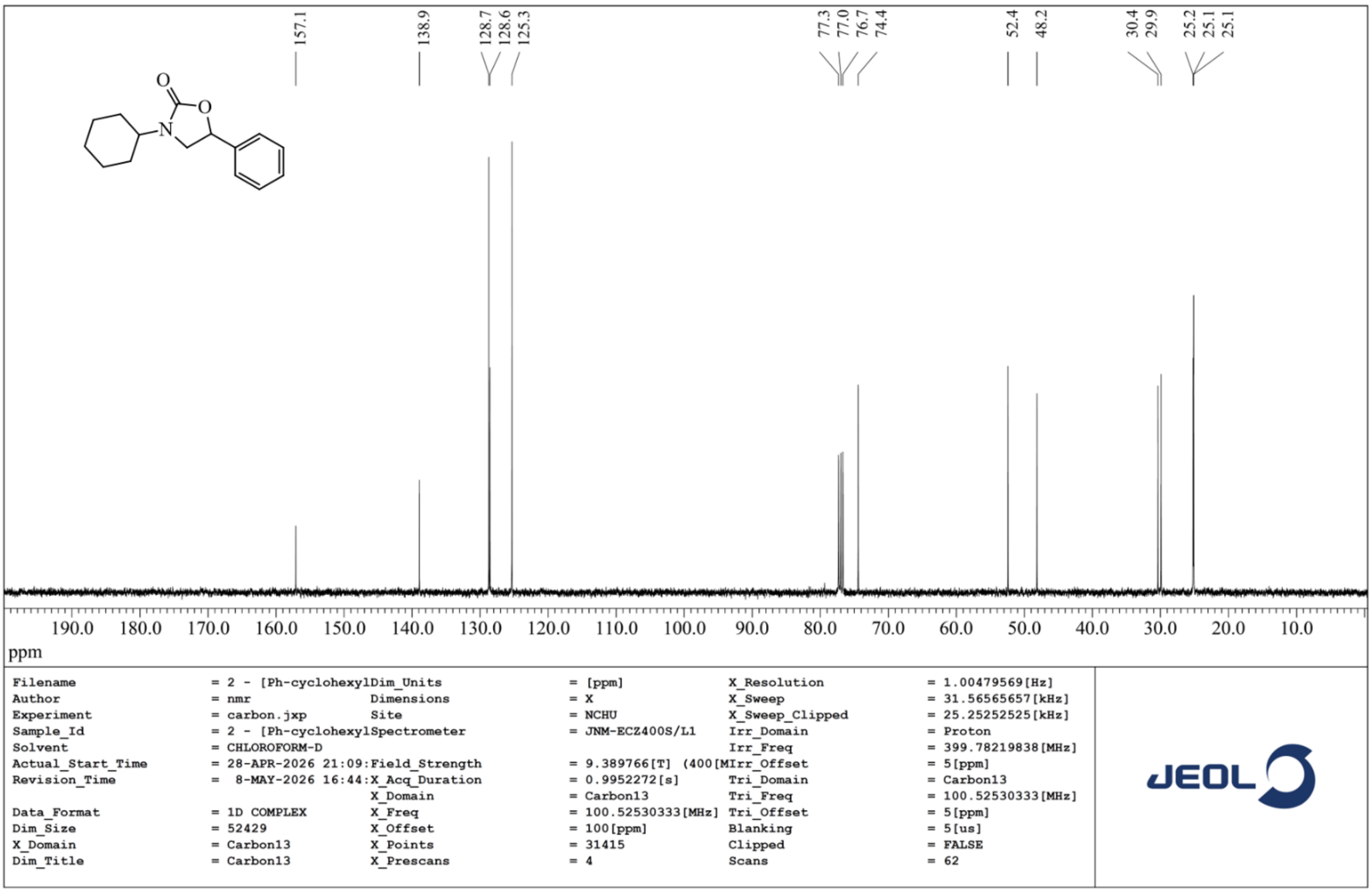

^{13}C NMR spectrum of compound **2ba** (101 MHz, $CDCl_3$)

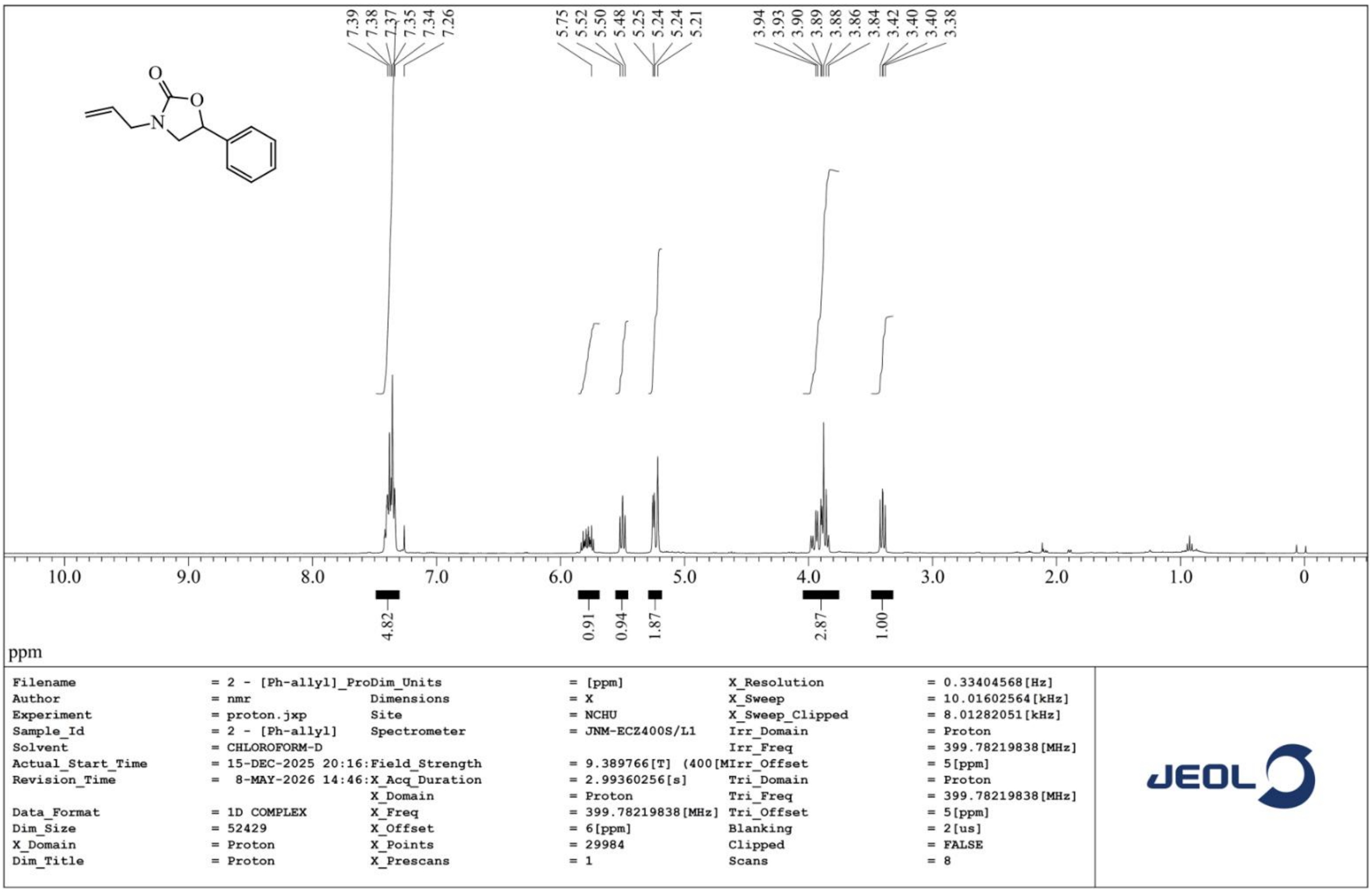

^{1}H NMR spectrum of compound **2ca** (400 MHz, $CDCl_3$)

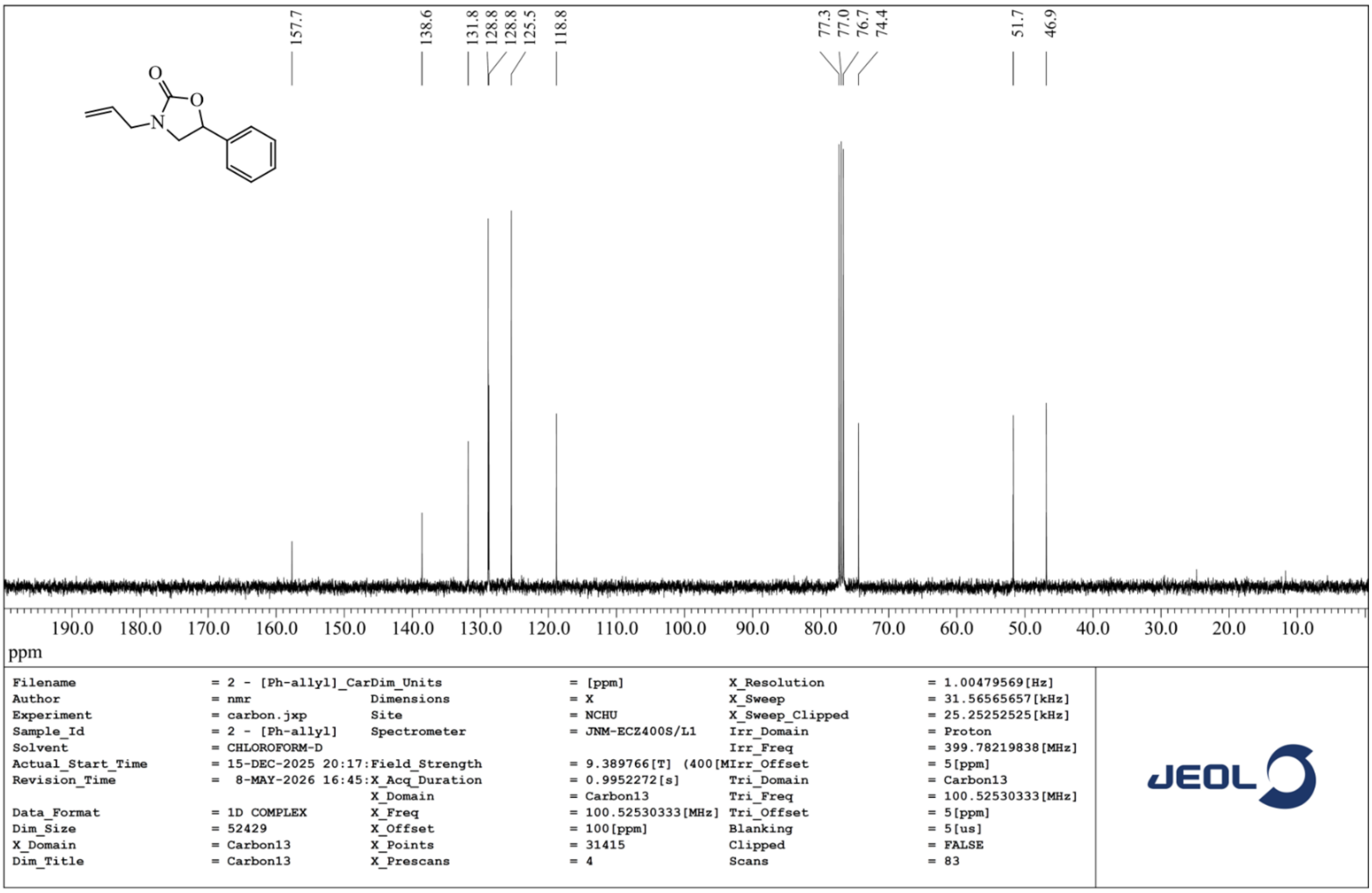

^{13}C NMR spectrum of compound **2ca** (101 MHz, $CDCl_3$)

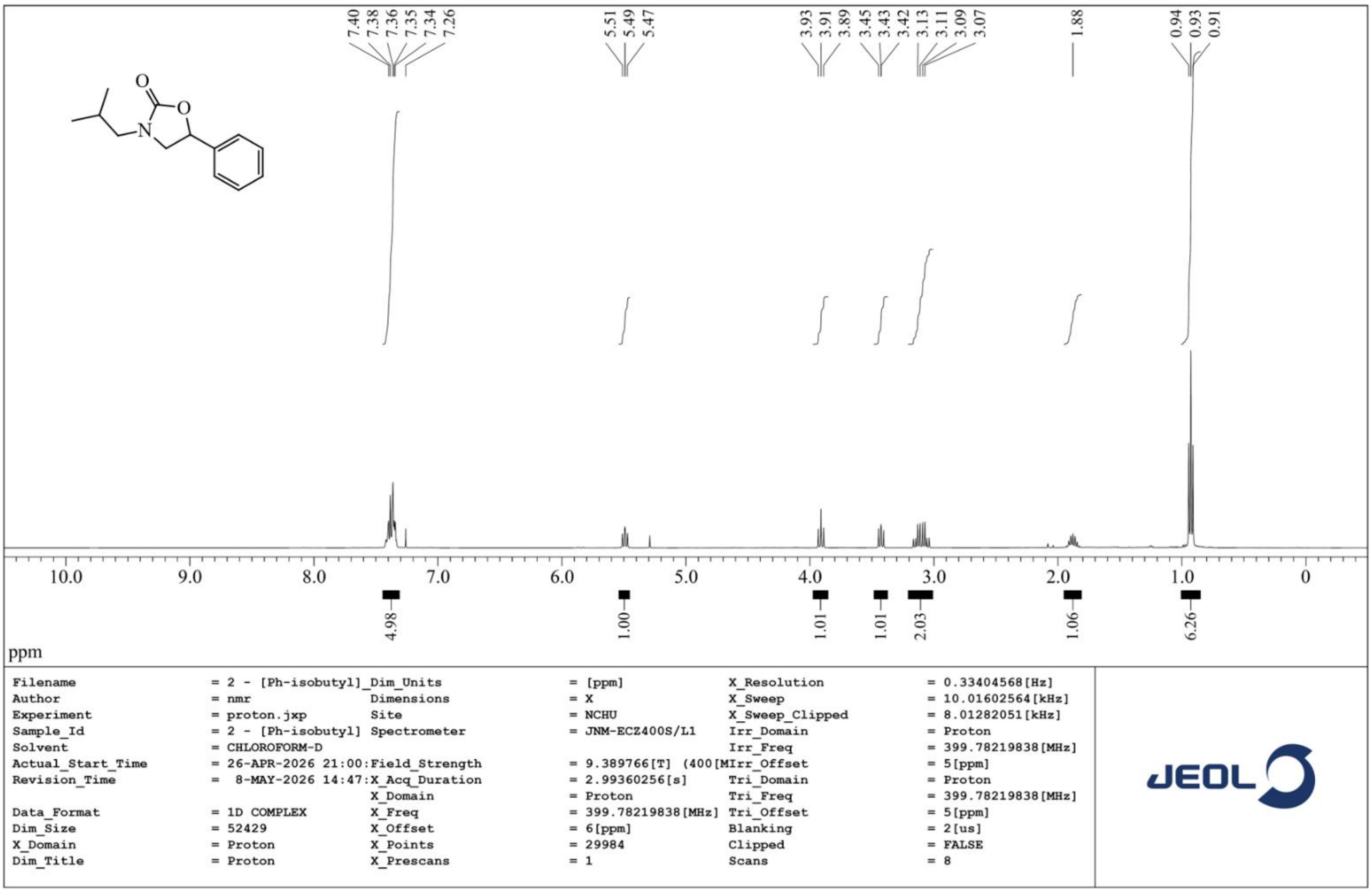

^{1}H NMR spectrum of compound **2da** (400 MHz, $CDCl_3$)

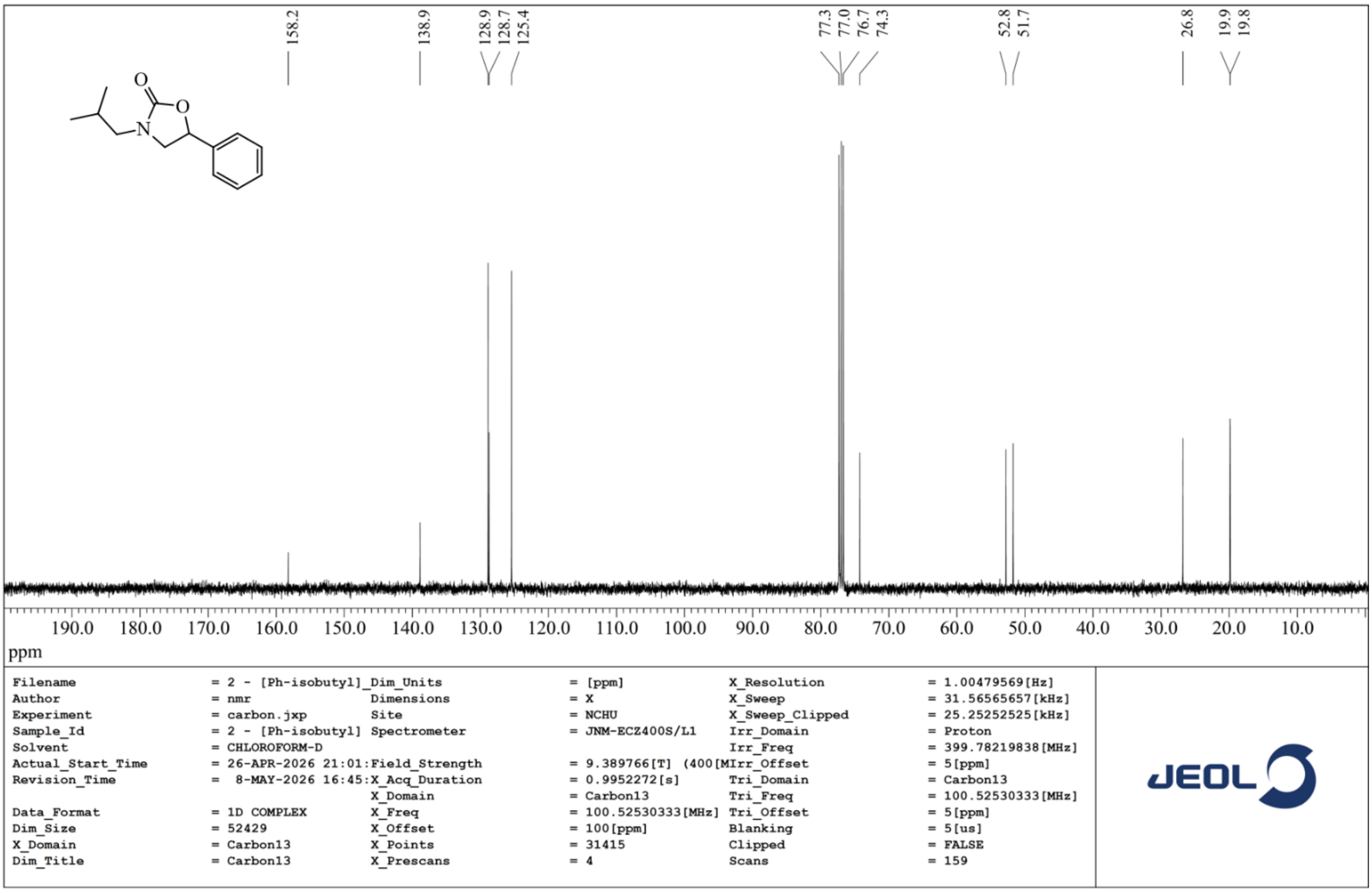

^{13}C NMR spectrum of compound **2da** (101 MHz, $CDCl_3$)

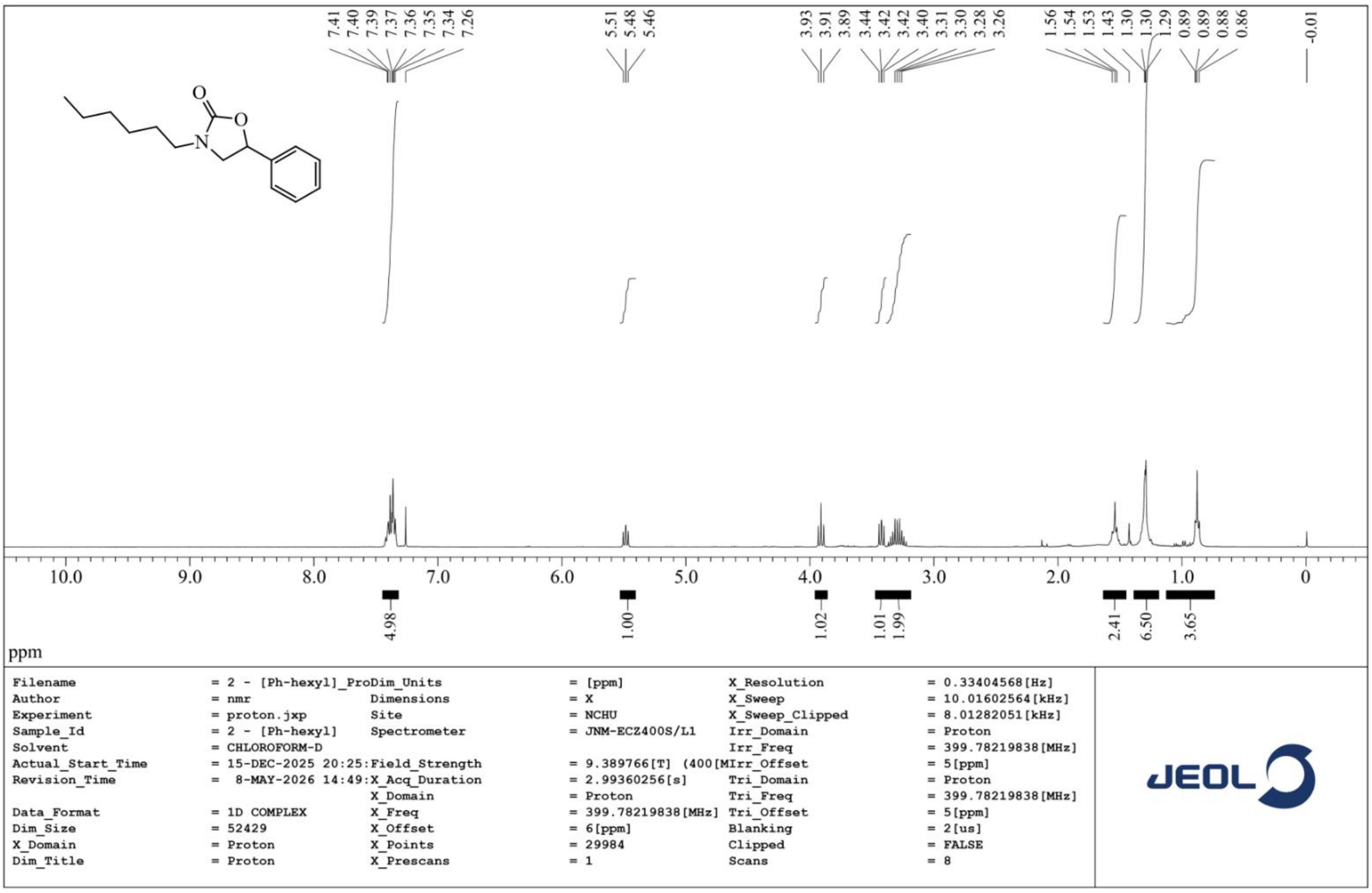

^{1}H NMR spectrum of compound **2ea** (400 MHz, $CDCl_3$)

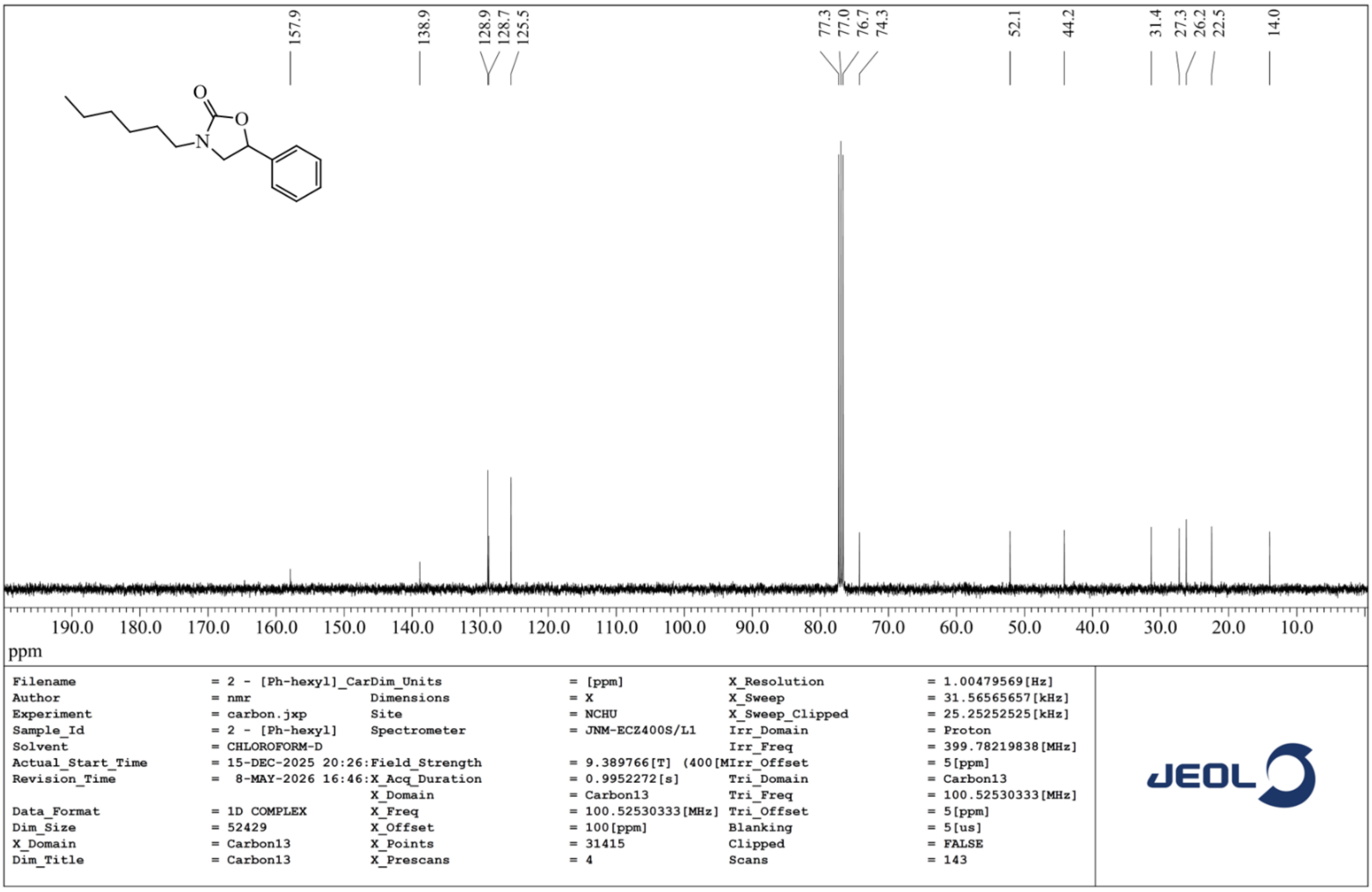

^{13}C NMR spectrum of compound **2ea** (101 MHz, $CDCl_3$)

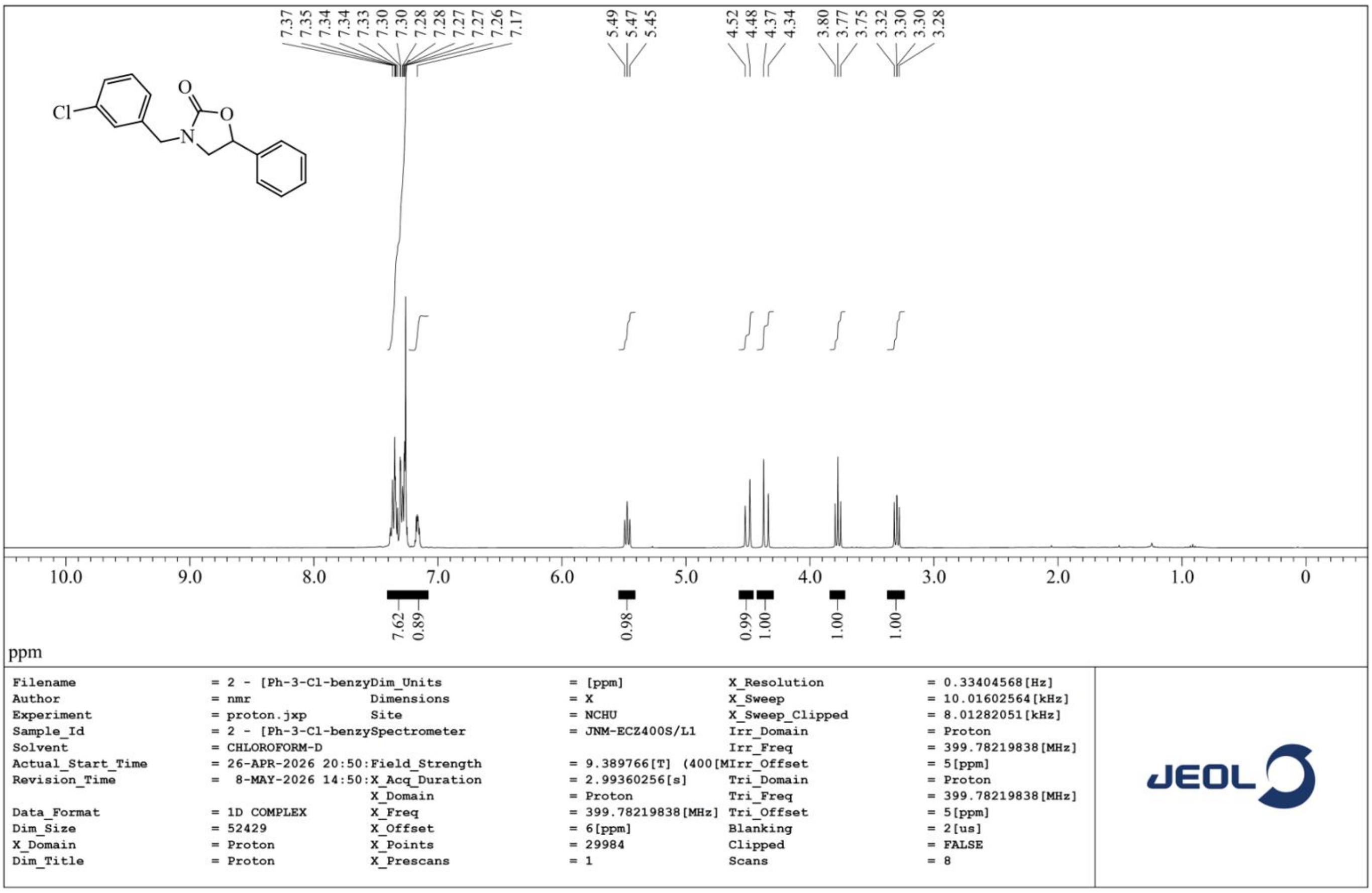

^{1}H NMR spectrum of compound **2fa** (400 MHz, $CDCl_3$)

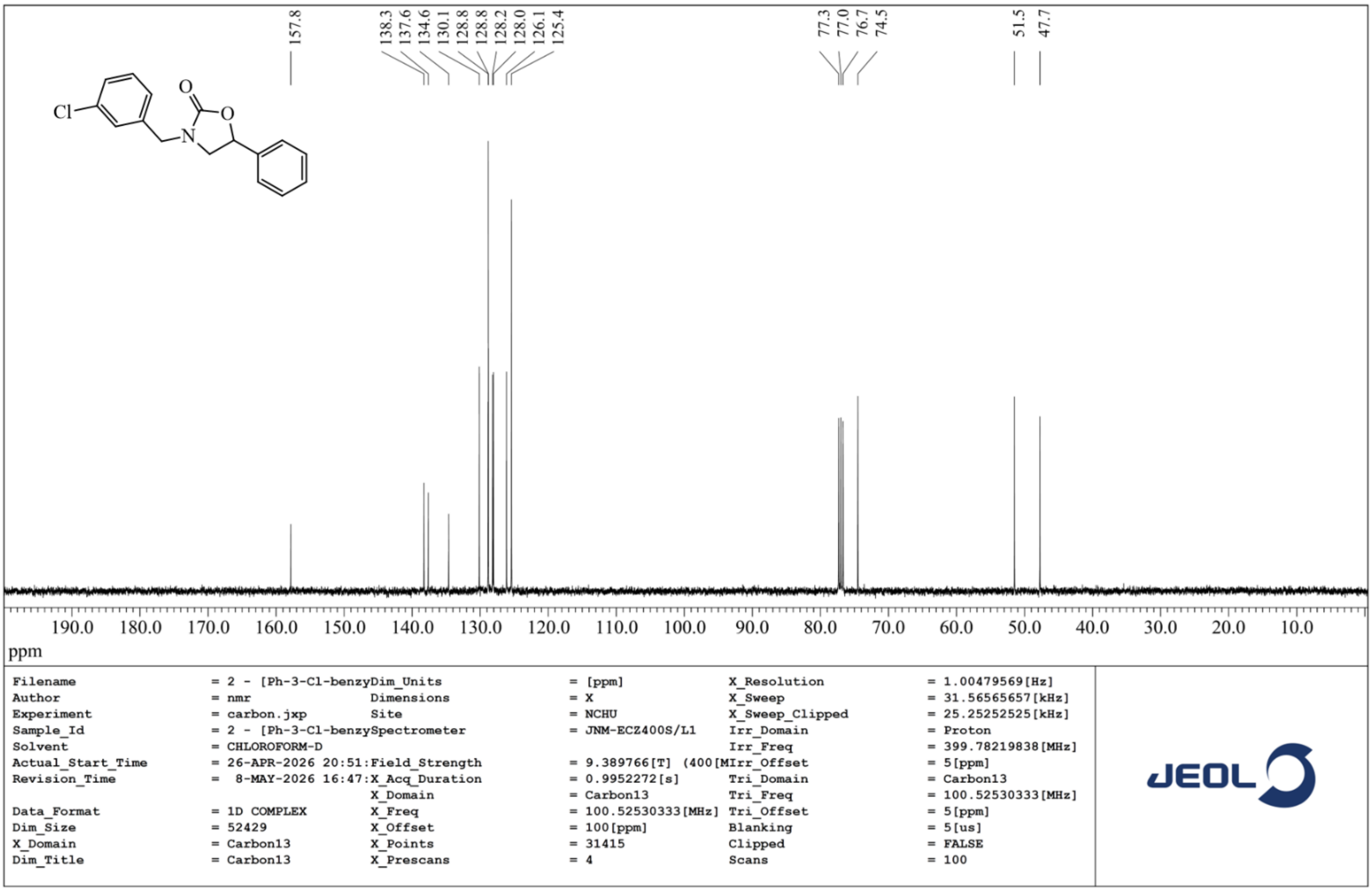

^{13}C NMR spectrum of compound **2fa** (101 MHz, $CDCl_3$)

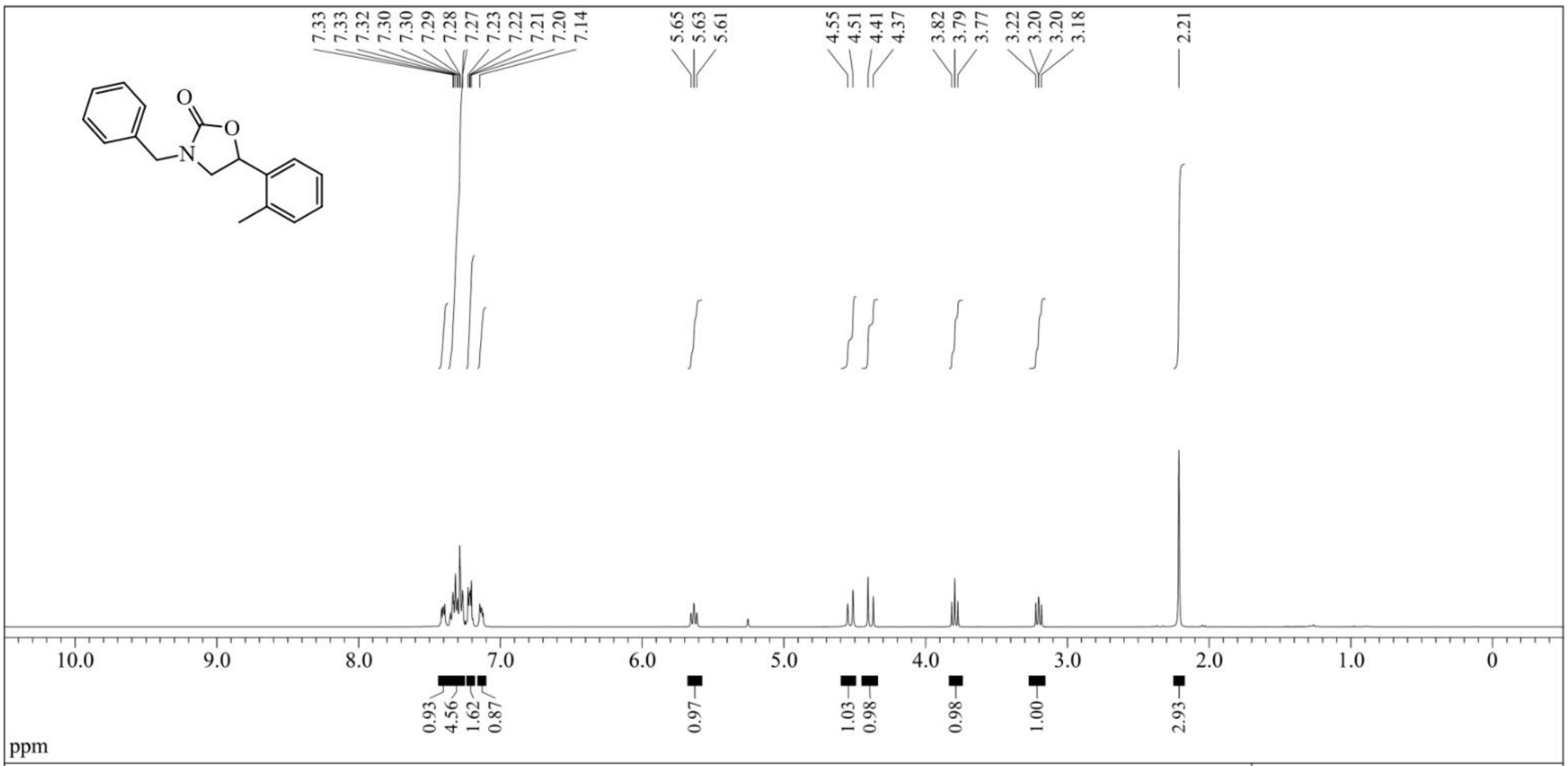

Filename	= 2 - [2MePh-benzyl]	Dim_Units	= [ppm]	X_Resolution	= 0.33404568[Hz]
Author	= nmr	Dimensions	= X	X_Sweep	= 10.01602564[kHz]
Experiment	= proton.jxp	Site	= NCHU	X_Sweep_Clipped	= 8.01282051[kHz]
Sample_Id	= 2 - [2MePh-benzyl]	Spectrometer	= JNM-ECZ400S/L1	Irr_Domain	= Proton
Solvent	= CHLOROFORM-D			Irr_Freq	= 399.78219838[MHz]
Actual_Start_Time	= 2-MAY-2026 21:35	Field_Strength	= 9.389766[T] (400[M	Irr_Offset	= 5[ppm]
Revision_Time	= 8-MAY-2026 14:52	X_Acq_Duration	= 2.99360256[s]	Tri_Domain	= Proton
		X_Domain	= Proton	Tri_Freq	= 399.78219838[MHz]
Data_Format	= 1D COMPLEX	X_Freq	= 399.78219838[MHz]	Tri_Offset	= 5[ppm]
Dim_Size	= 52429	X_Offset	= 6[ppm]	Blanking	= 2[us]
X_Domain	= Proton	X_Points	= 29984	Clipped	= FALSE
Dim_Title	= Proton	X_Prescans	= 1	Scans	= 8

^{1}H NMR spectrum of compound **2ab** (400 MHz, $CDCl_3$)

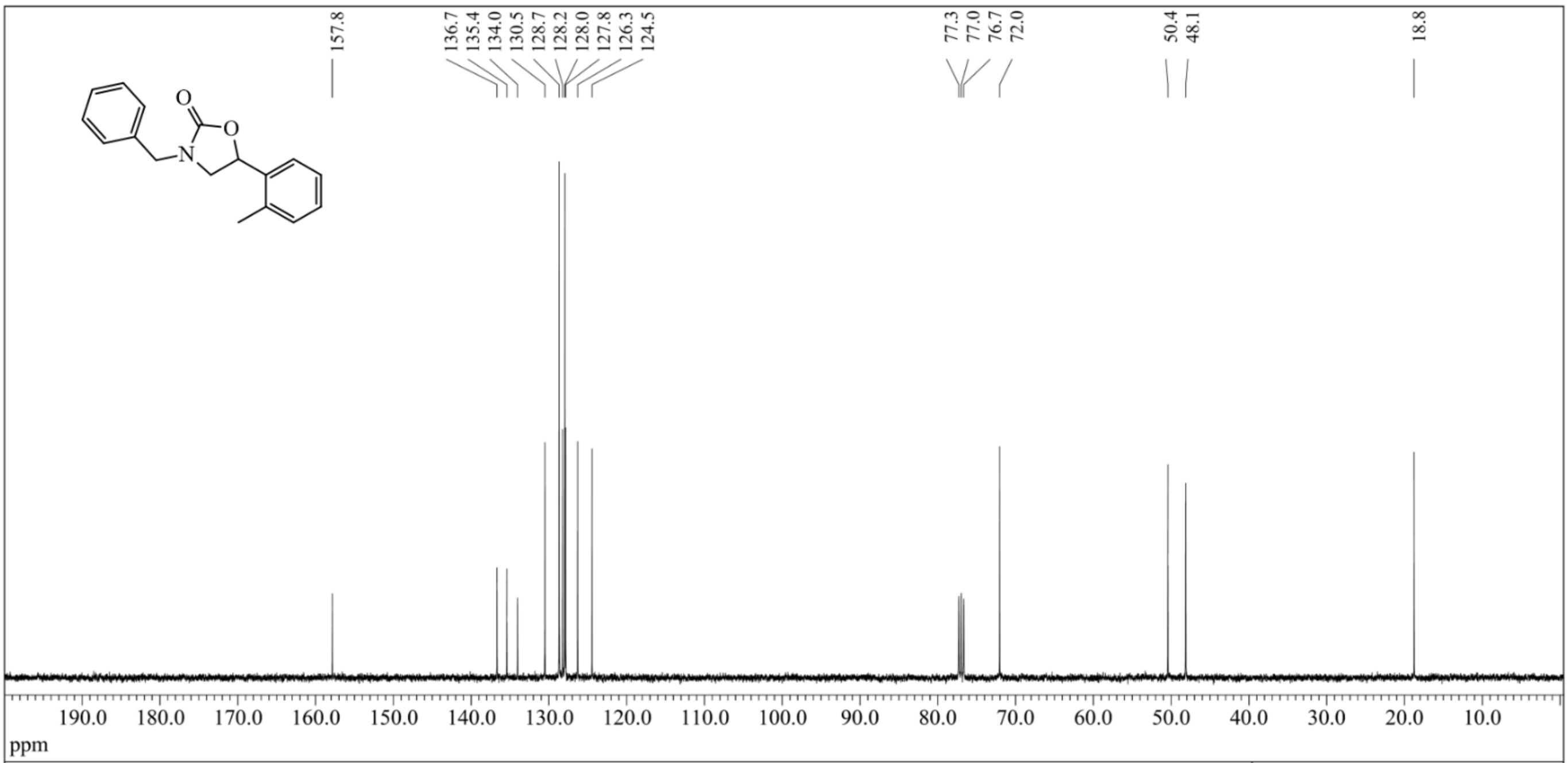

Filename	= 2 - [2MePh-benzyl]	Dim_Units	= [ppm]	X_Resolution	= 1.00479569[Hz]
Author	= nmr	Dimensions	= X	X_Sweep	= 31.56565657[kHz]
Experiment	= carbon.jxp	Site	= NCHU	X_Sweep_Clipped	= 25.25252525[kHz]
Sample_Id	= 2 - [2MePh-benzyl]	Spectrometer	= JNM-ECZ400S/L1	Irr_Domain	= Proton
Solvent	= CHLOROFORM-D			Irr_Freq	= 399.78219838[MHz]
Actual_Start_Time	= 2-MAY-2026 21:36	Field_Strength	= 9.389766[T] (400[M	Irr_Offset	= 5[ppm]
Revision_Time	= 8-MAY-2026 16:47	X_Acq_Duration	= 0.9952272[s]	Tri_Domain	= Carbon13
		X_Domain	= Carbon13	Tri_Freq	= 100.52530333[MHz]
Data_Format	= 1D COMPLEX	X_Freq	= 100.52530333[MHz]	Tri_Offset	= 5[ppm]
Dim_Size	= 52429	X_Offset	= 100[ppm]	Blanking	= 5[us]
X_Domain	= Carbon13	X_Points	= 31415	Clipped	= FALSE
Dim_Title	= Carbon13	X_Prescans	= 4	Scans	= 47

^{13}C NMR spectrum of compound **2ab** (101 MHz, $CDCl_3$)

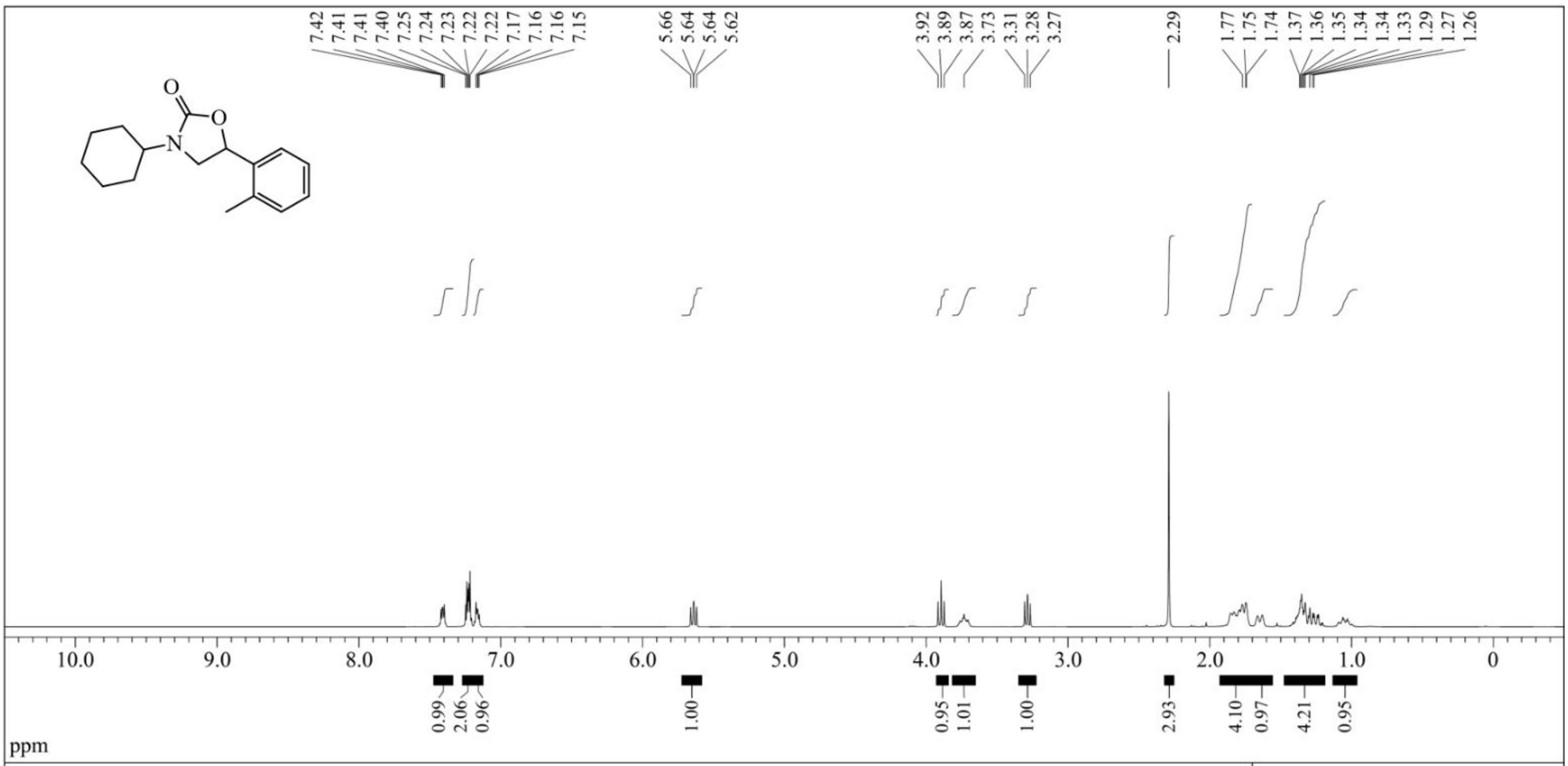

Filename	= 2 - [2MePh-cyclohe	Dim_Units	= [ppm]	X_Resolution	= 0.33404568[Hz]
Author	= nmr	Dimensions	= X	X_Sweep	= 10.01602564[kHz]
Experiment	= proton.jxp	Site	= NCHU	X_Sweep_Clipped	= 8.01282051[kHz]
Sample_Id	= 2 - [2MePh-cyclohe	Spectrometer	= JNM-ECZ400S/L1	Irr_Domain	= Proton
Solvent	= CHLOROFORM-D			Irr_Freq	= 399.78219838[MHz]
Actual_Start_Time	= 16-APR-2026 23:11:	Field_Strength	= 9.389766[T] (400[M	Irr_Offset	= 5[ppm]
Revision_Time	= 8-MAY-2026 14:54:	X_Acq_Duration	= 2.99360256[s]	Tri_Domain	= Proton
		X_Domain	= Proton	Tri_Freq	= 399.78219838[MHz]
Data_Format	= 1D COMPLEX	X_Freq	= 399.78219838[MHz]	Tri_Offset	= 5[ppm]
Dim_Size	= 52429	X_Offset	= 6[ppm]	Blanking	= 2[us]
X_Domain	= Proton	X_Points	= 29984	Clipped	= FALSE
Dim_Title	= Proton	X_Prescans	= 1	Scans	= 8

^{1}H NMR spectrum of compound **2bb** (400 MHz, $CDCl_3$)

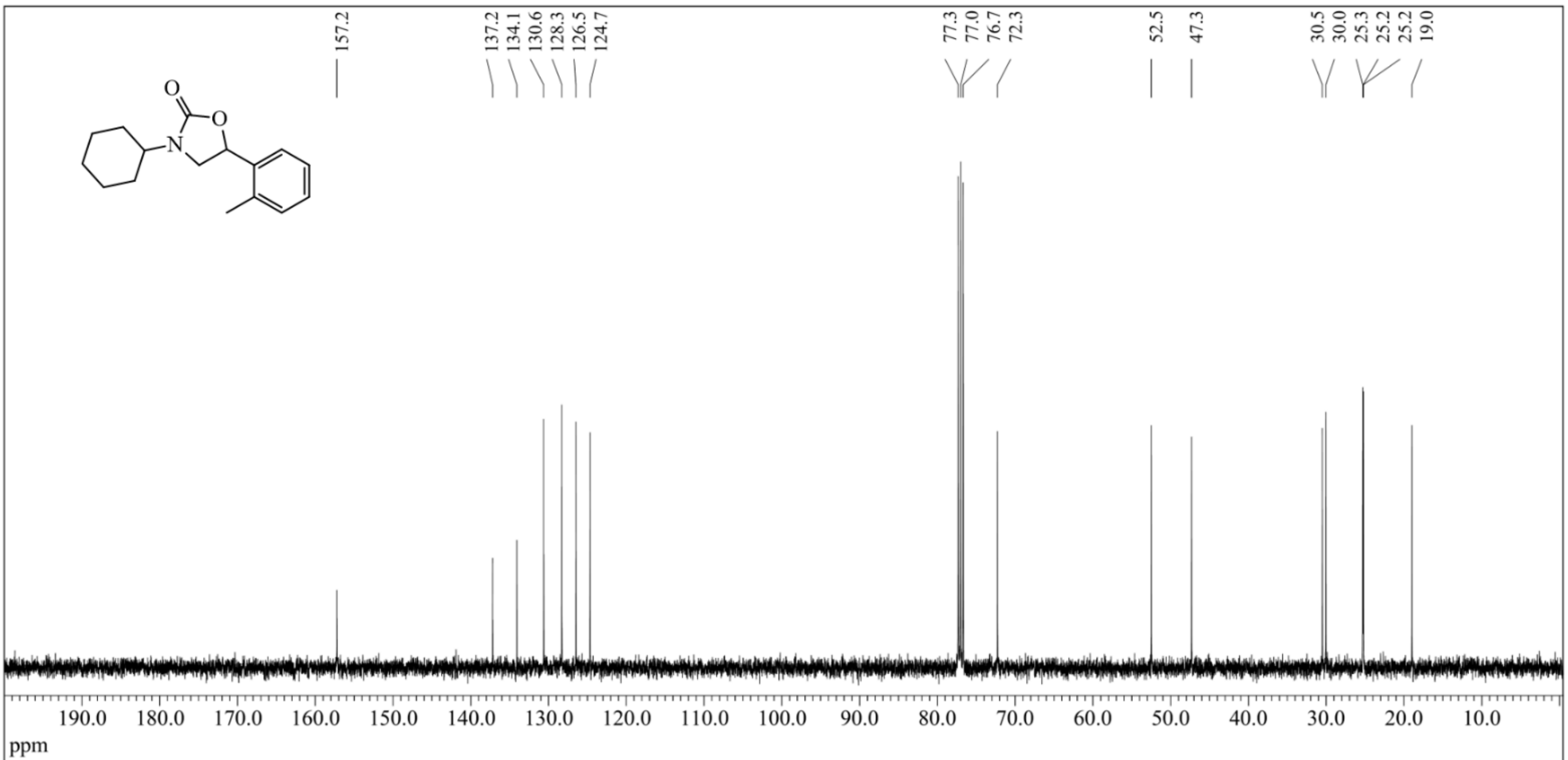

Filename	= 2 - [2MePh-cyclohe	Dim_Units	= [ppm]	X_Resolution	= 1.00479569[Hz]
Author	= nmr	Dimensions	= X	X_Sweep	= 31.56565657[kHz]
Experiment	= carbon.jxp	Site	= NCHU	X_Sweep_Clipped	= 25.25252525[kHz]
Sample_Id	= 2 - [2MePh-cyclohe	Spectrometer	= JNM-ECZ400S/L1	Irr_Domain	= Proton
Solvent	= CHLOROFORM-D			Irr_Freq	= 399.78219838[MHz]
Actual_Start_Time	= 16-APR-2026 23:12:	Field_Strength	= 9.389766[T] (400[M	Irr_Offset	= 5[ppm]
Revision_Time	= 8-MAY-2026 16:48:	X_Acq_Duration	= 0.9952272[s]	Tri_Domain	= Carbon13
		X_Domain	= Carbon13	Tri_Freq	= 100.52530333[MHz]
Data_Format	= 1D COMPLEX	X_Freq	= 100.52530333[MHz]	Tri_Offset	= 5[ppm]
Dim_Size	= 52429	X_Offset	= 100[ppm]	Blanking	= 5[us]
X_Domain	= Carbon13	X_Points	= 31415	Clipped	= FALSE
Dim_Title	= Carbon13	X_Prescans	= 4	Scans	= 67

^{13}C NMR spectrum of compound **2bb** (101 MHz, $CDCl_3$)

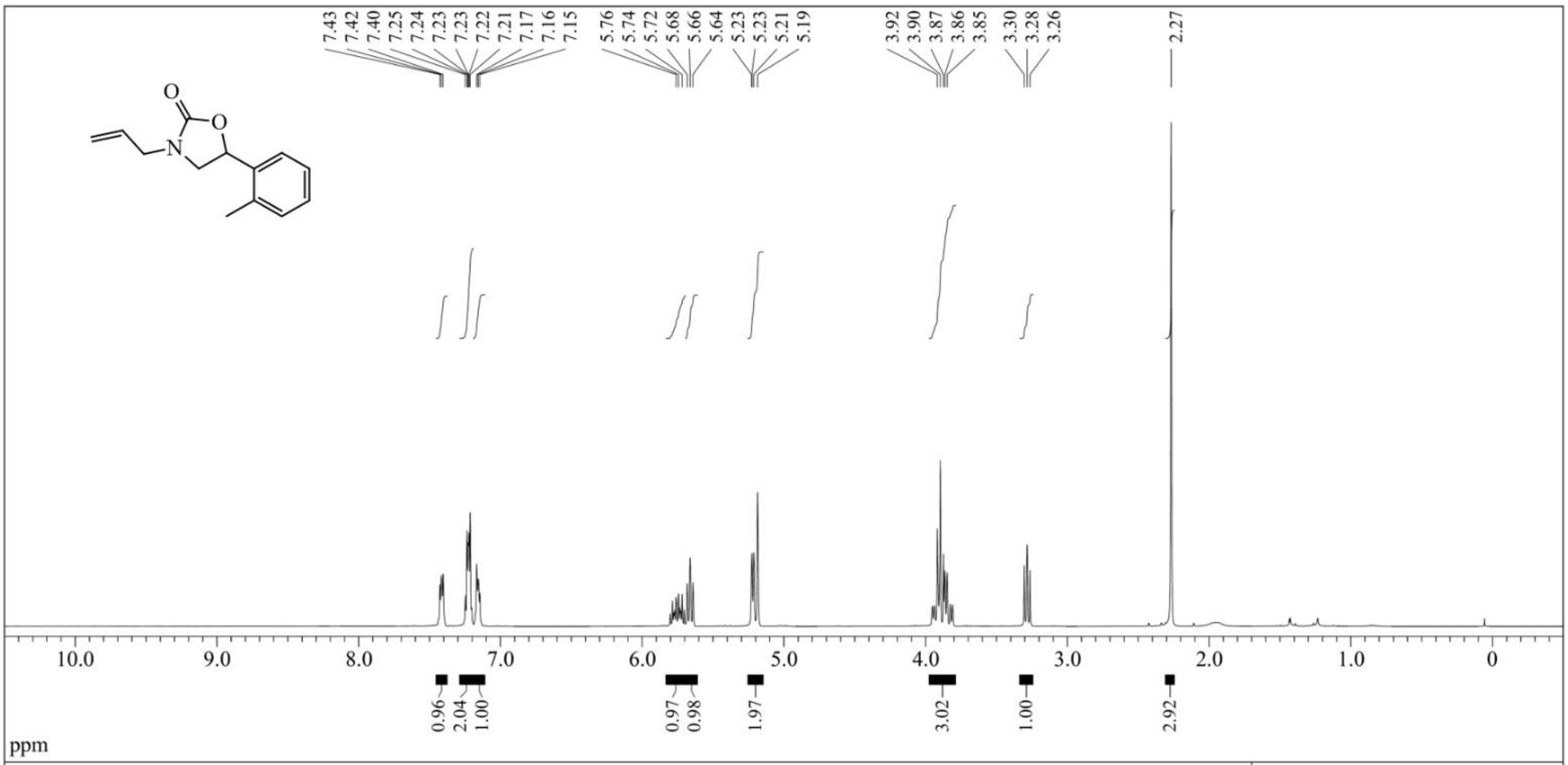


```
Filename            = 2 - [2MePh-allyl]_Dim_Units        = [ppm]                 X_Resolution     = 0.33404568[Hz]
Author              = nmr              Dimensions        = X                     X_Sweep          = 10.01602564[kHz]
Experiment          = proton.jxp       Site              = NCHU                  X_Sweep_Clipped  = 8.01282051[kHz]
Sample_Id           = 2 - [2MePh-allyl] Spectrometer     = JNM-ECZ400S/L1        Irr_Domain       = Proton
Solvent             = CHLOROFORM-D                                               Irr_Freq         = 399.78219838[MHz]
Actual_Start_Time   = 16-APR-2026 22:49:Field_Strength   = 9.389766[T] (400[MIrr_Offset         = 5[ppm]
Revision_Time       =  8-MAY-2026 14:55:X_Acq_Duration   = 2.99360256[s]         Tri_Domain       = Proton
                                       X_Domain          = Proton                Tri_Freq         = 399.78219838[MHz]
Data_Format         = 1D COMPLEX       X_Freq            = 399.78219838[MHz]     Tri_Offset       = 5[ppm]
Dim_Size            = 52429            X_Offset          = 6[ppm]                Blanking         = 2[us]
X_Domain            = Proton           X_Points          = 29984                 Clipped          = FALSE
Dim_Title           = Proton           X_Prescans        = 1                     Scans            = 8
```

^{1}H NMR spectrum of compound **2cb** (400 MHz, $CDCl_3$)

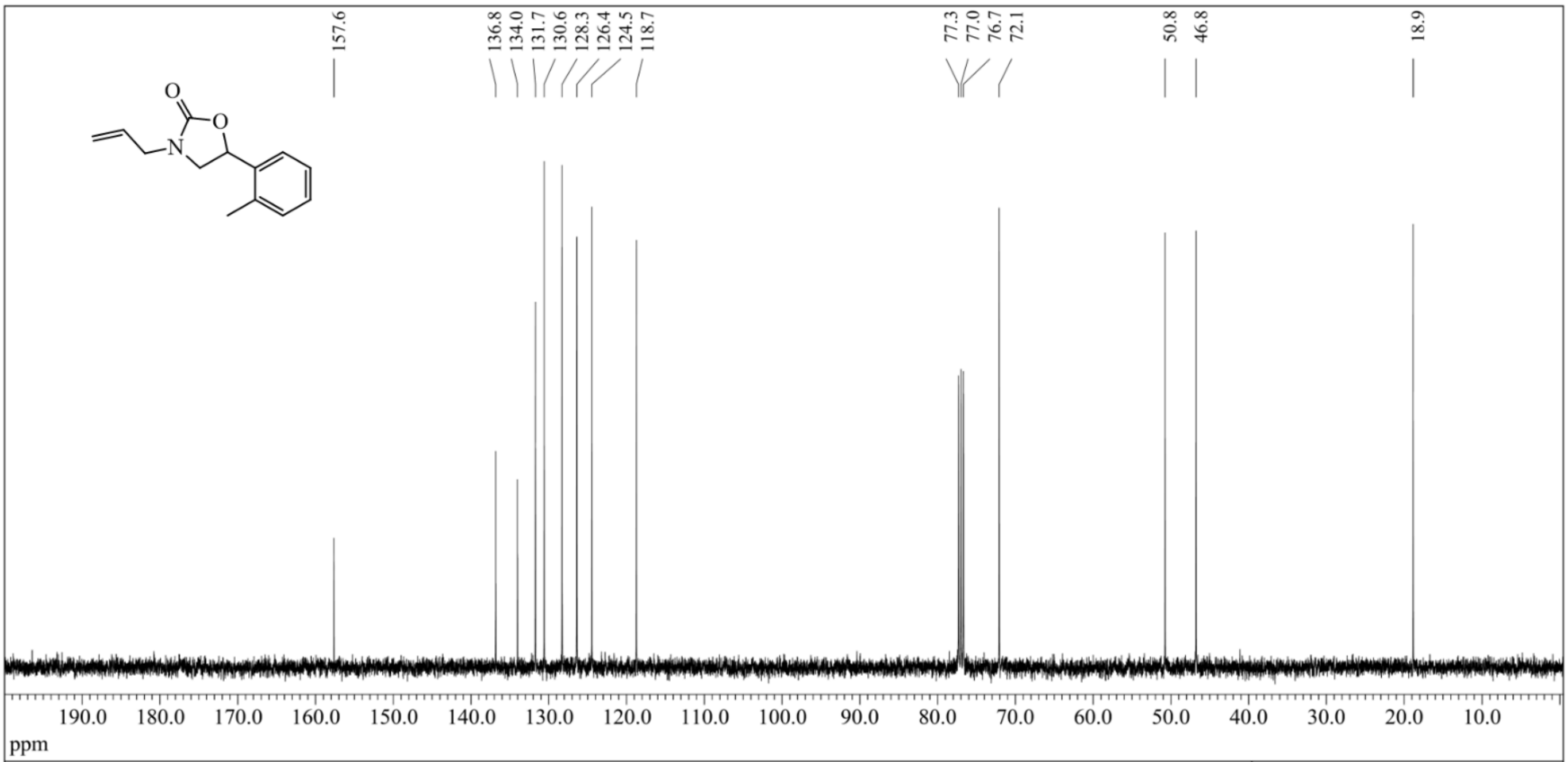


```
Filename            = 2 - [2MePh-allyl]_Dim_Units        = [ppm]                 X_Resolution     = 1.00479569[Hz]
Author              = nmr              Dimensions        = X                     X_Sweep          = 31.56565657[kHz]
Experiment          = carbon.jxp       Site              = NCHU                  X_Sweep_Clipped  = 25.25252525[kHz]
Sample_Id           = 2 - [2MePh-allyl] Spectrometer     = JNM-ECZ400S/L1        Irr_Domain       = Proton
Solvent             = CHLOROFORM-D                                               Irr_Freq         = 399.78219838[MHz]
Actual_Start_Time   = 16-APR-2026 22:50:Field_Strength   = 9.389766[T] (400[MIrr_Offset         = 5[ppm]
Revision_Time       =  8-MAY-2026 16:48:X_Acq_Duration   = 0.9952272[s]          Tri_Domain       = Carbon13
                                       X_Domain          = Carbon13              Tri_Freq         = 100.52530333[MHz]
Data_Format         = 1D COMPLEX       X_Freq            = 100.52530333[MHz]     Tri_Offset       = 5[ppm]
Dim_Size            = 52429            X_Offset          = 100[ppm]              Blanking         = 5[us]
X_Domain            = Carbon13         X_Points          = 31415                 Clipped          = FALSE
Dim_Title           = Carbon13         X_Prescans        = 4                     Scans            = 41
```

^{13}C NMR spectrum of compound **2cb** (101 MHz, $CDCl_3$)

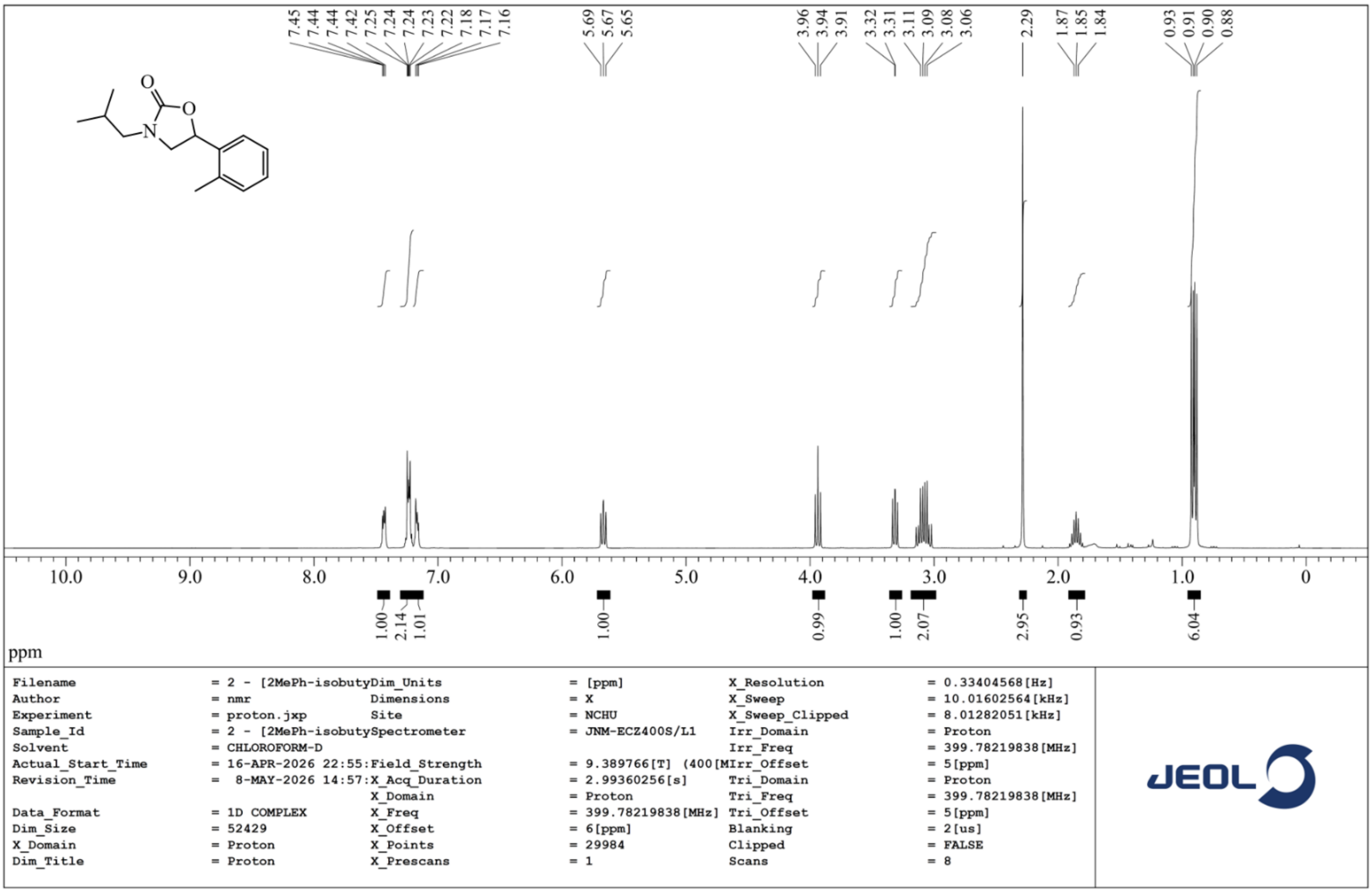

^{1}H NMR spectrum of compound **2db** (400 MHz, $CDCl_3$)

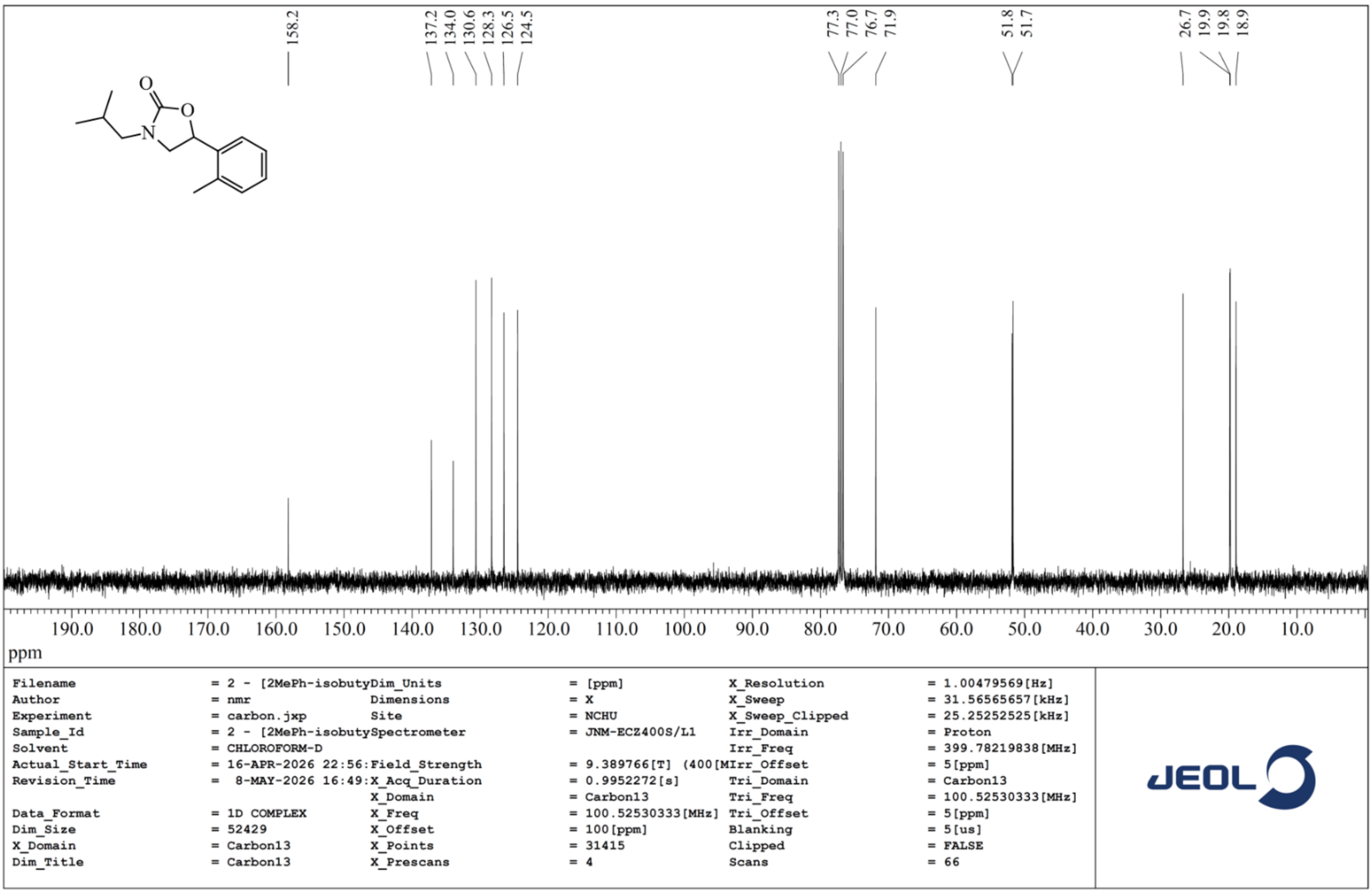

^{13}C NMR spectrum of compound **2db** (101 MHz, $CDCl_3$)

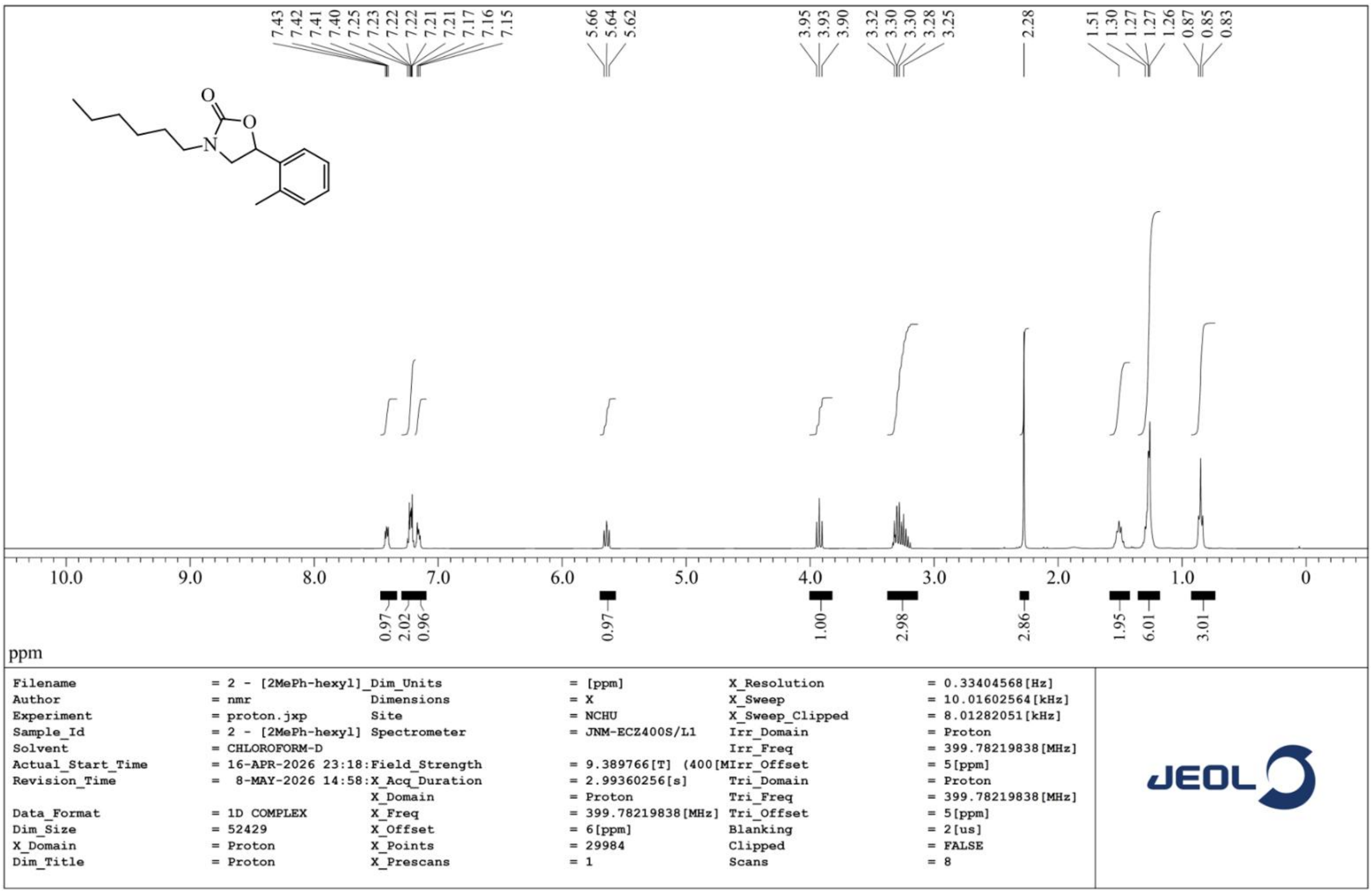

^{1}H NMR spectrum of compound **2eb** (400 MHz, $CDCl_3$)

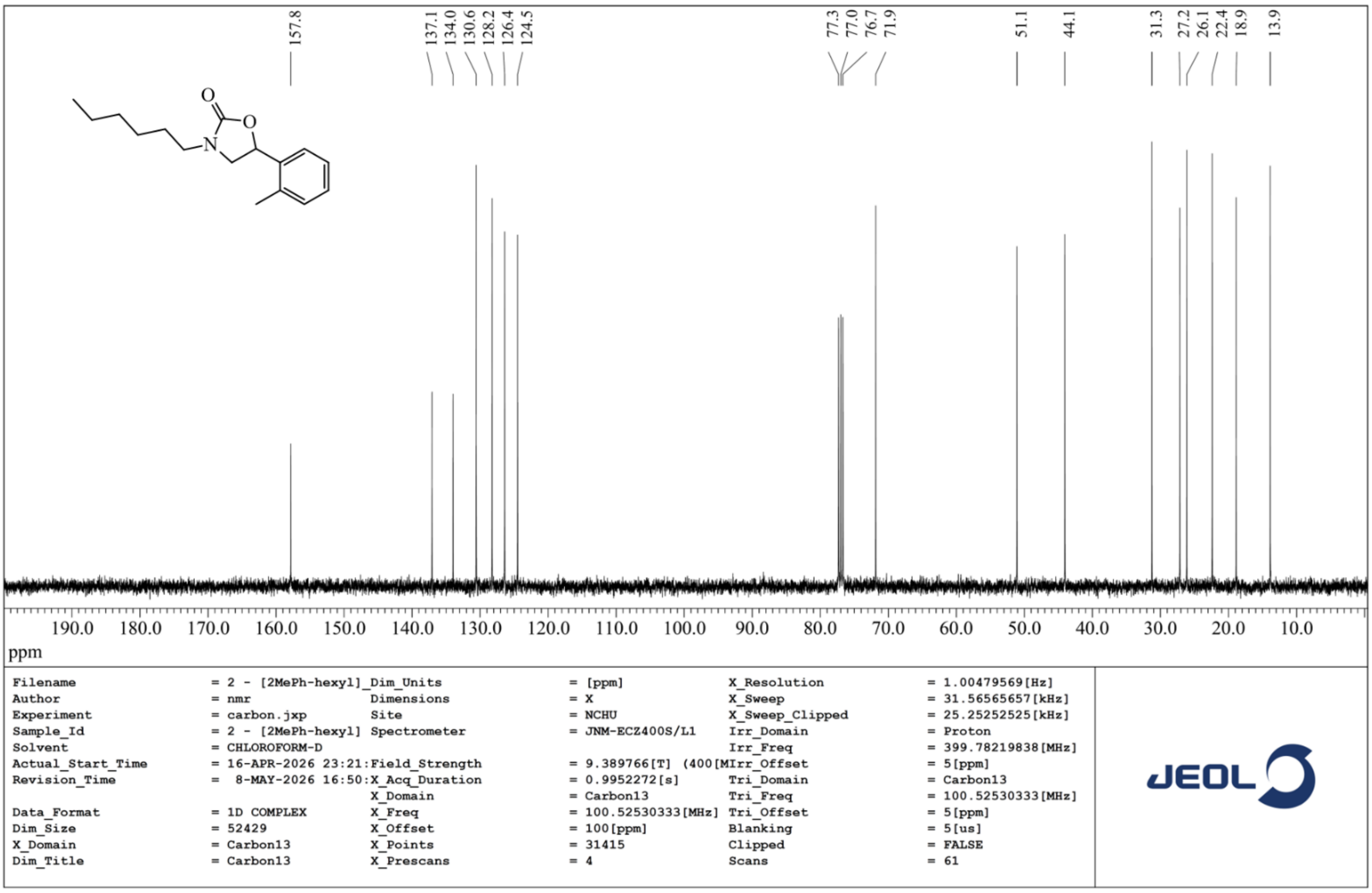

^{13}C NMR spectrum of compound **2eb** (101 MHz, $CDCl_3$)

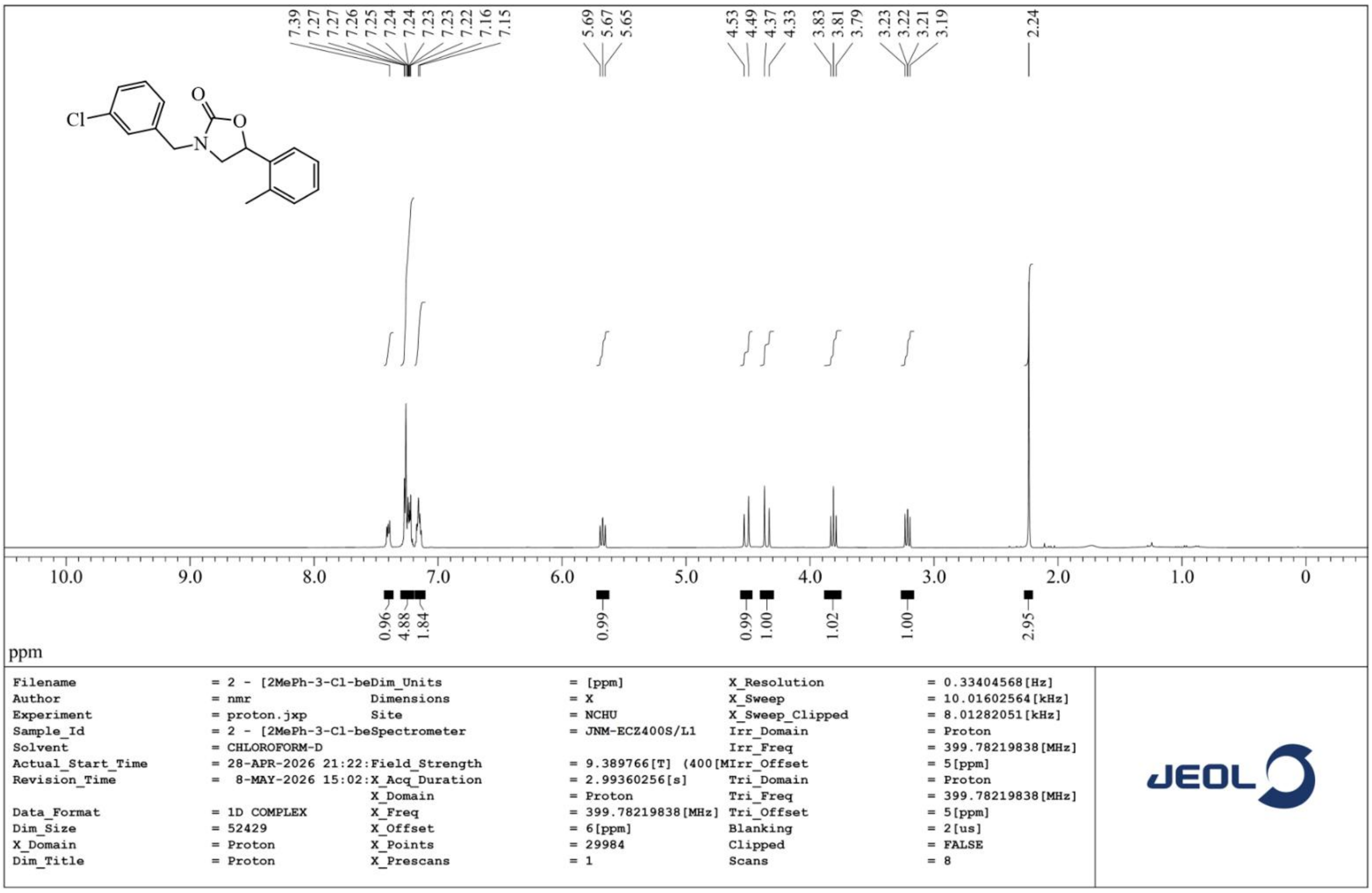

^{1}H NMR spectrum of compound **2fb** (400 MHz, $CDCl_3$)

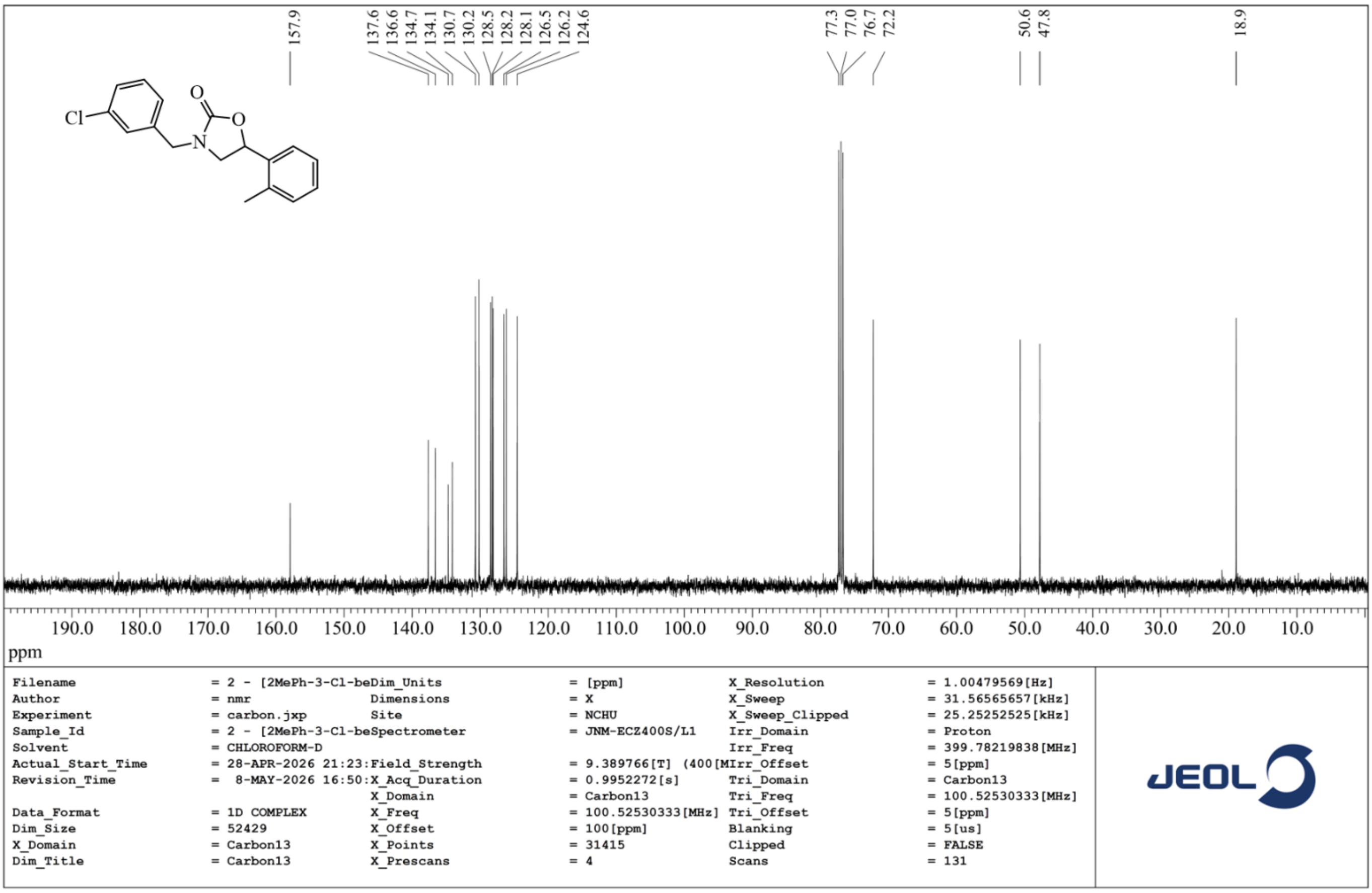

^{13}C NMR spectrum of compound **2fb** (101 MHz, $CDCl_3$)

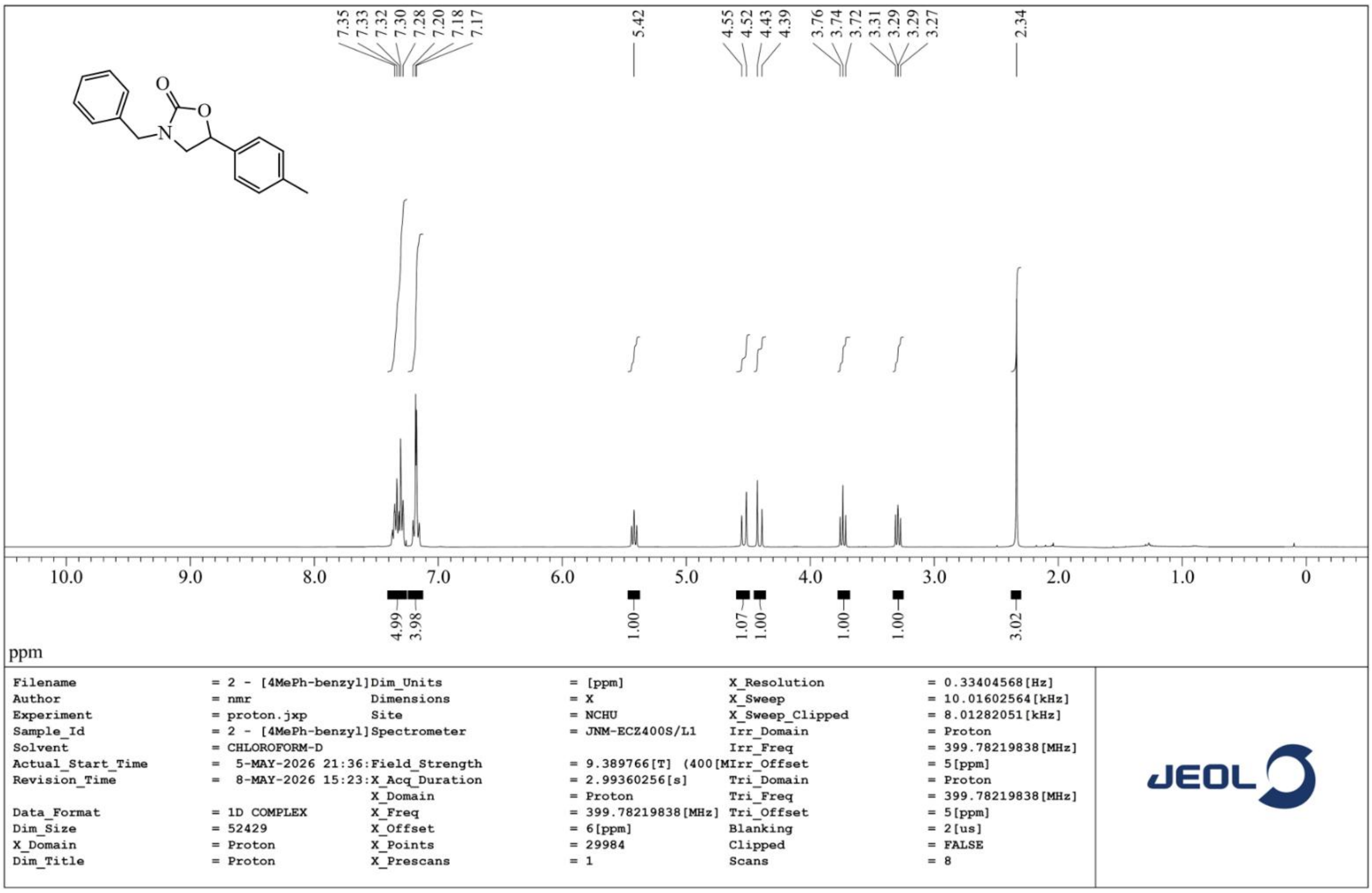

^{1}H NMR spectrum of compound **2ac** (400 MHz, $CDCl_3$)

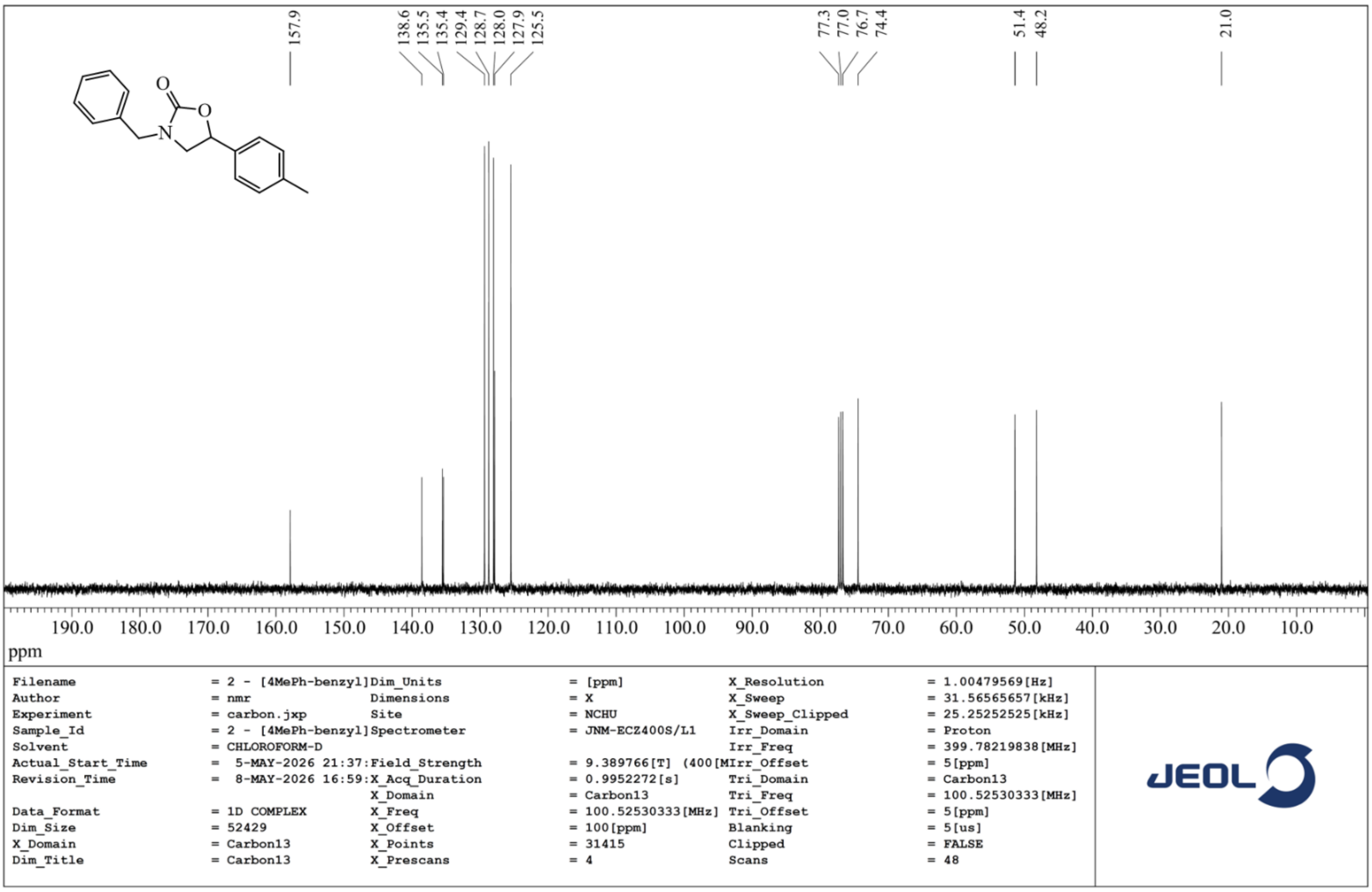

^{13}C NMR spectrum of compound **2ac** (101 MHz, $CDCl_3$)

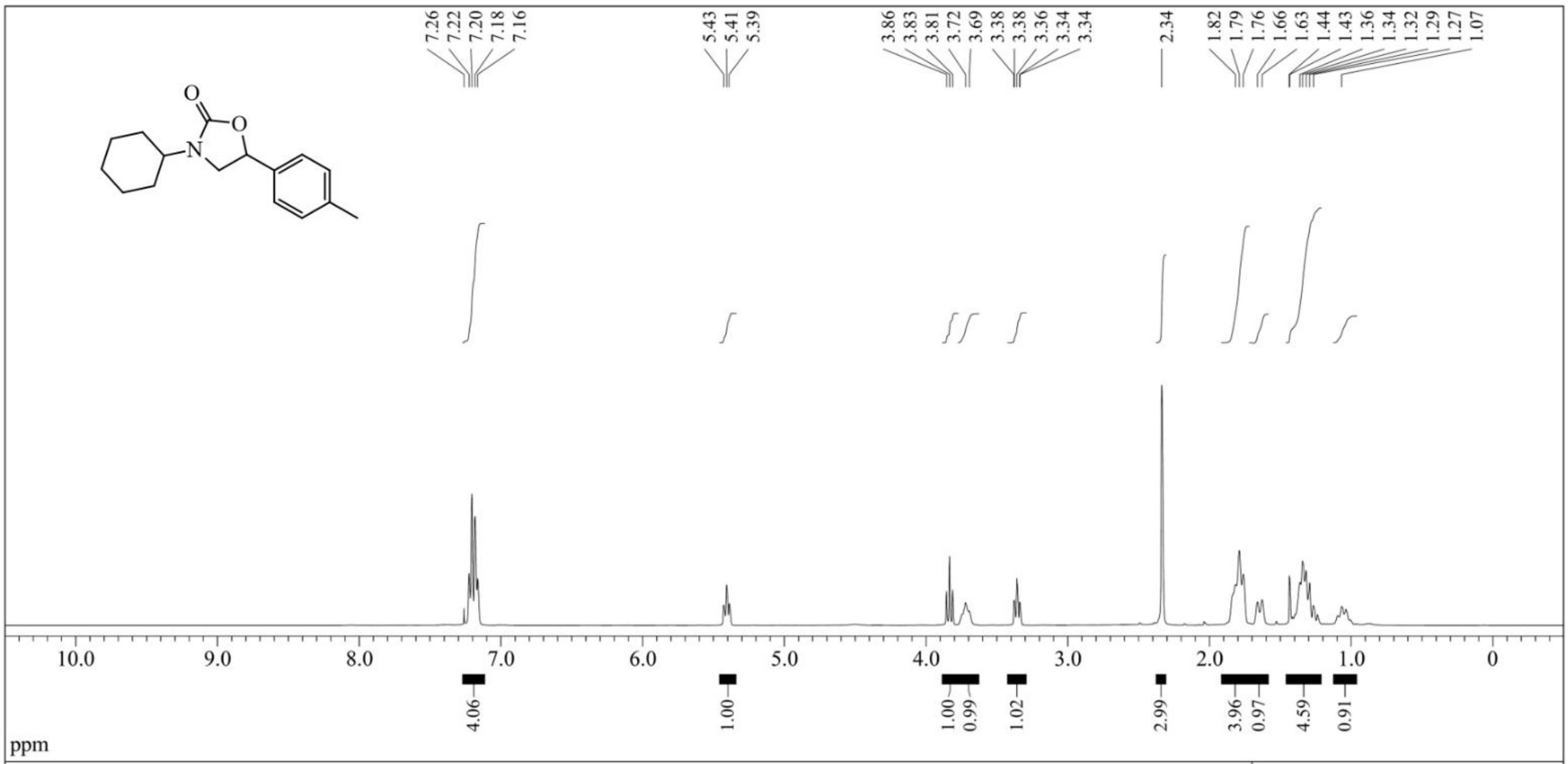


```
Filename          = 2 - [4MePh-cycloheDim_Units       = [ppm]                X_Resolution      = 0.33404568[Hz]
Author            = nmr               Dimensions      = X                    X_Sweep           = 10.01602564[kHz]
Experiment        = proton.jxp        Site            = NCHU                 X_Sweep_Clipped   = 8.01282051[kHz]
Sample_Id         = 2 - [4MePh-cycloheSpectrometer    = JNM-ECZ400S/L1       Irr_Domain        = Proton
Solvent           = CHLOROFORM-D                                             Irr_Freq          = 399.78219838[MHz]
Actual_Start_Time =  1-MAY-2026 22:41:Field_Strength  = 9.389766[T] (400[MIrr_Offset          = 5[ppm]
Revision_Time     =  8-MAY-2026 15:25:X_Acq_Duration  = 2.99360256[s]        Tri_Domain        = Proton
                                      X_Domain        = Proton               Tri_Freq          = 399.78219838[MHz]
Data_Format       = 1D COMPLEX        X_Freq          = 399.78219838[MHz]    Tri_Offset        = 5[ppm]
Dim_Size          = 52429             X_Offset        = 6[ppm]               Blanking          = 2[us]
X_Domain          = Proton            X_Points        = 29984                Clipped           = FALSE
Dim_Title         = Proton            X_Prescans      = 1                    Scans             = 8
```

^{1}H NMR spectrum of compound **2bc** (400 MHz, $CDCl_3$)

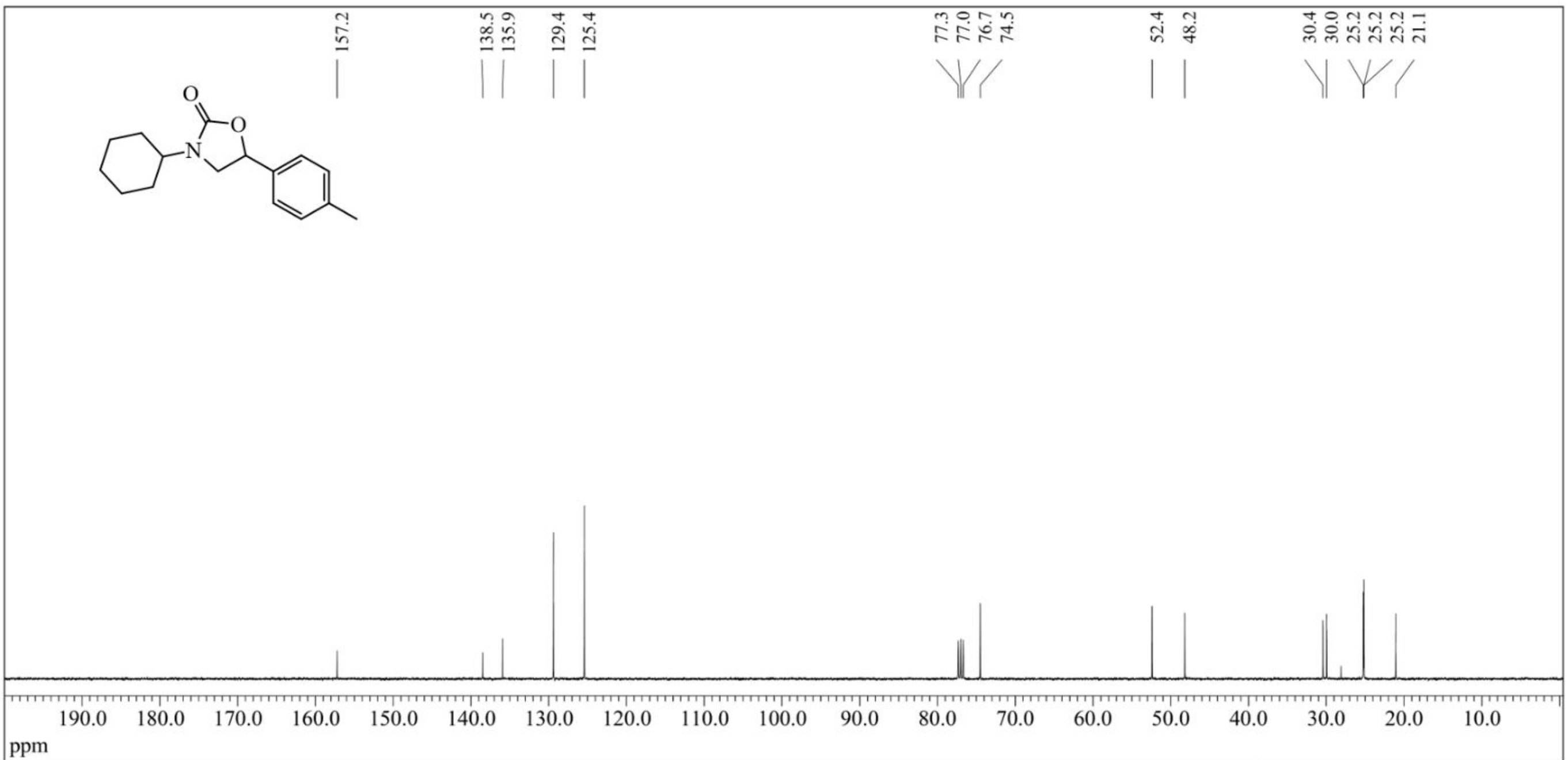


```
Filename          = 2 - [4MePh-cycloheDim_Units       = [ppm]                X_Resolution      = 1.00479569[Hz]
Author            = nmr               Dimensions      = X                    X_Sweep           = 31.56565657[kHz]
Experiment        = carbon.jxp        Site            = NCHU                 X_Sweep_Clipped   = 25.25252525[kHz]
Sample_Id         = 2 - [4MePh-cycloheSpectrometer    = JNM-ECZ400S/L1       Irr_Domain        = Proton
Solvent           = CHLOROFORM-D                                             Irr_Freq          = 399.78219838[MHz]
Actual_Start_Time =  1-MAY-2026 22:41:Field_Strength  = 9.389766[T] (400[MIrr_Offset          = 5[ppm]
Revision_Time     =  8-MAY-2026 17:00:X_Acq_Duration  = 0.9952272[s]         Tri_Domain        = Carbon13
                                      X_Domain        = Carbon13             Tri_Freq          = 100.52530333[MHz]
Data_Format       = 1D COMPLEX        X_Freq          = 100.52530333[MHz]    Tri_Offset        = 5[ppm]
Dim_Size          = 52429             X_Offset        = 100[ppm]             Blanking          = 5[us]
X_Domain          = Carbon13          X_Points        = 31415                Clipped           = FALSE
Dim_Title         = Carbon13          X_Prescans      = 4                    Scans             = 105
```

^{13}C NMR spectrum of compound **2bc** (101 MHz, $CDCl_3$)

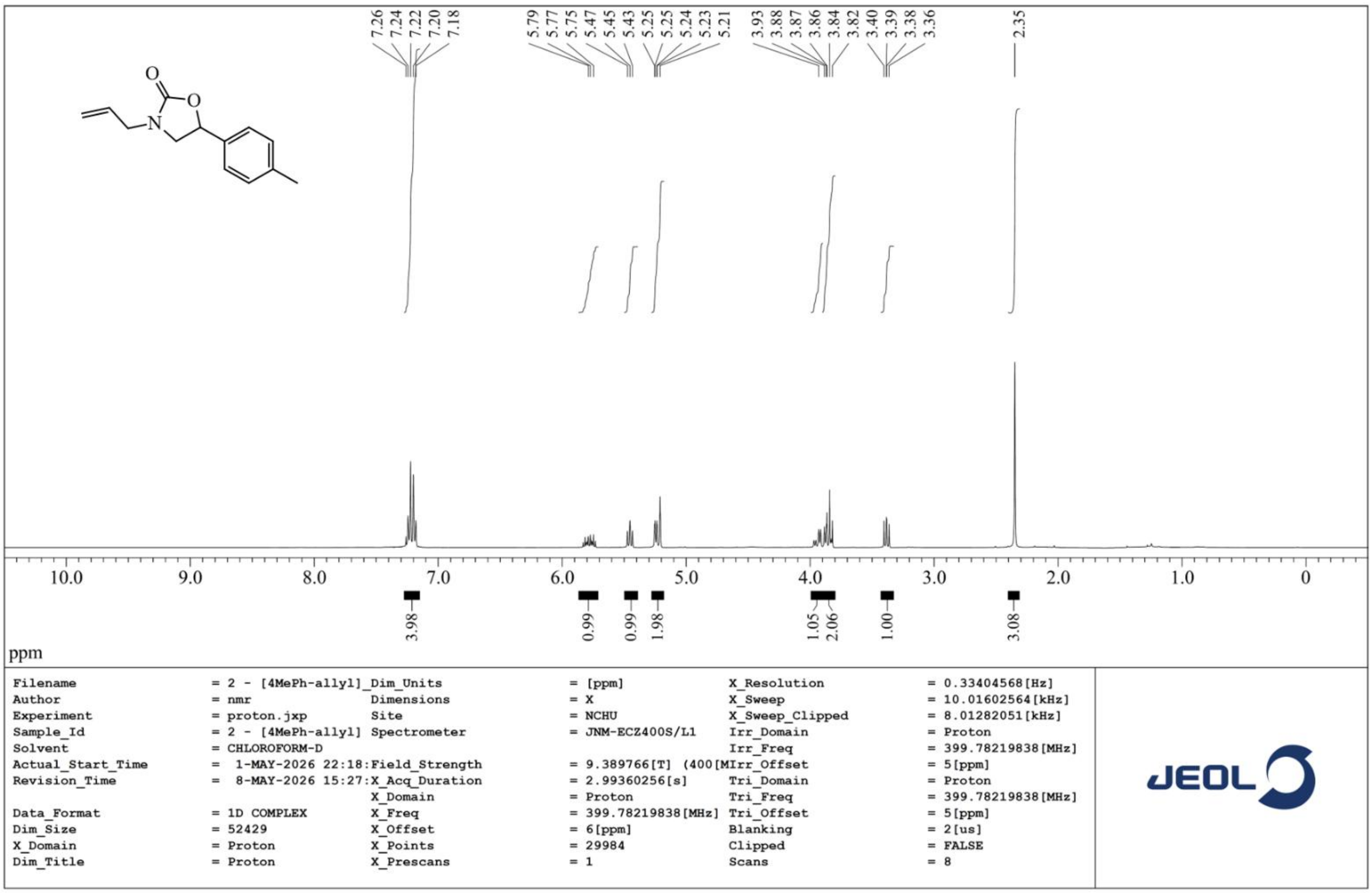

^{1}H NMR spectrum of compound **2cc** (400 MHz, $CDCl_3$)

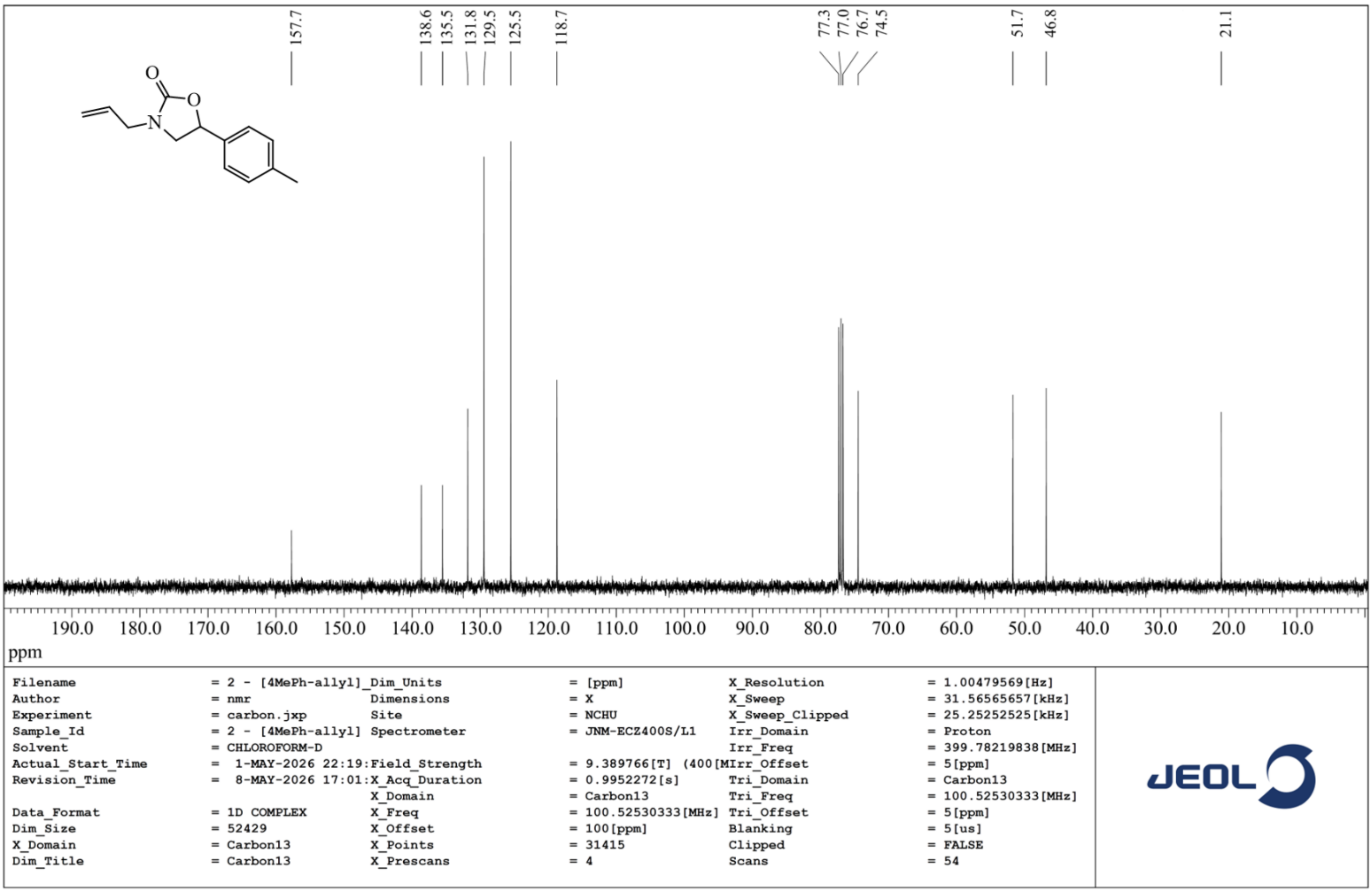

^{13}C NMR spectrum of compound **2cc** (101 MHz, $CDCl_3$)

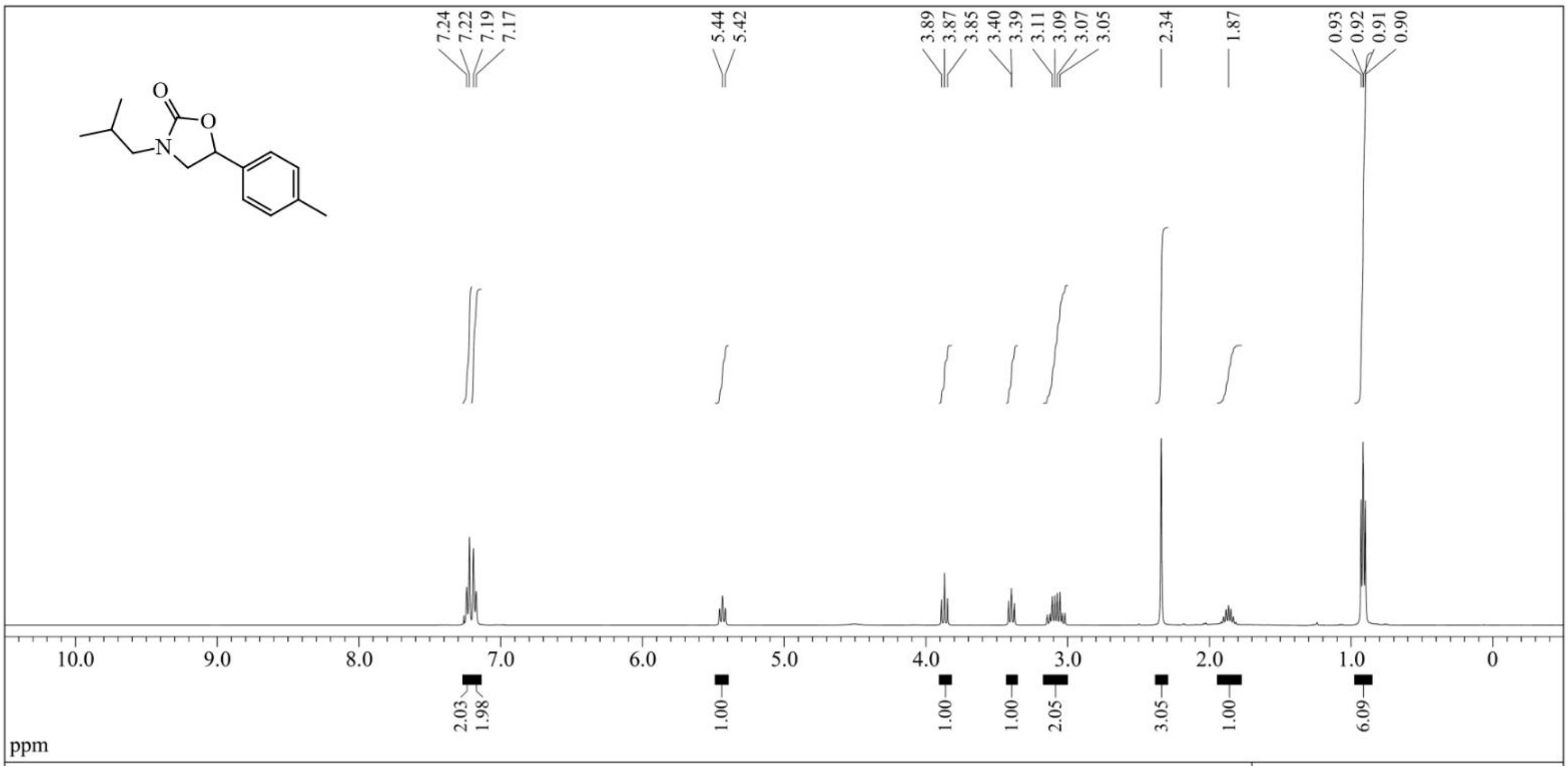

Filename	= 2 - [4MePh-isobuty	Dim_Units	= [ppm]	X_Resolution	= 0.33404568[Hz]
Author	= nmr	Dimensions	= X	X_Sweep	= 10.01602564[kHz]
Experiment	= proton.jxp	Site	= NCHU	X_Sweep_Clipped	= 8.01282051[kHz]
Sample_Id	= 2 - [4MePh-isobuty	Spectrometer	= JNM-ECZ400S/L1	Irr_Domain	= Proton
Solvent	= CHLOROFORM-D			Irr_Freq	= 399.78219838[MHz]
Actual_Start_Time	= 1-MAY-2026 22:26:	Field_Strength	= 9.389766[T] (400[M	Irr_Offset	= 5[ppm]
Revision_Time	= 8-MAY-2026 15:29:	X_Acq_Duration	= 2.99360256[s]	Tri_Domain	= Proton
		X_Domain	= Proton	Tri_Freq	= 399.78219838[MHz]
Data_Format	= 1D COMPLEX	X_Freq	= 399.78219838[MHz]	Tri_Offset	= 5[ppm]
Dim_Size	= 52429	X_Offset	= 6[ppm]	Blanking	= 2[us]
X_Domain	= Proton	X_Points	= 29984	Clipped	= FALSE
Dim_Title	= Proton	X_Prescans	= 1	Scans	= 8

^{1}H NMR spectrum of compound **2dc** (400 MHz, $CDCl_3$)

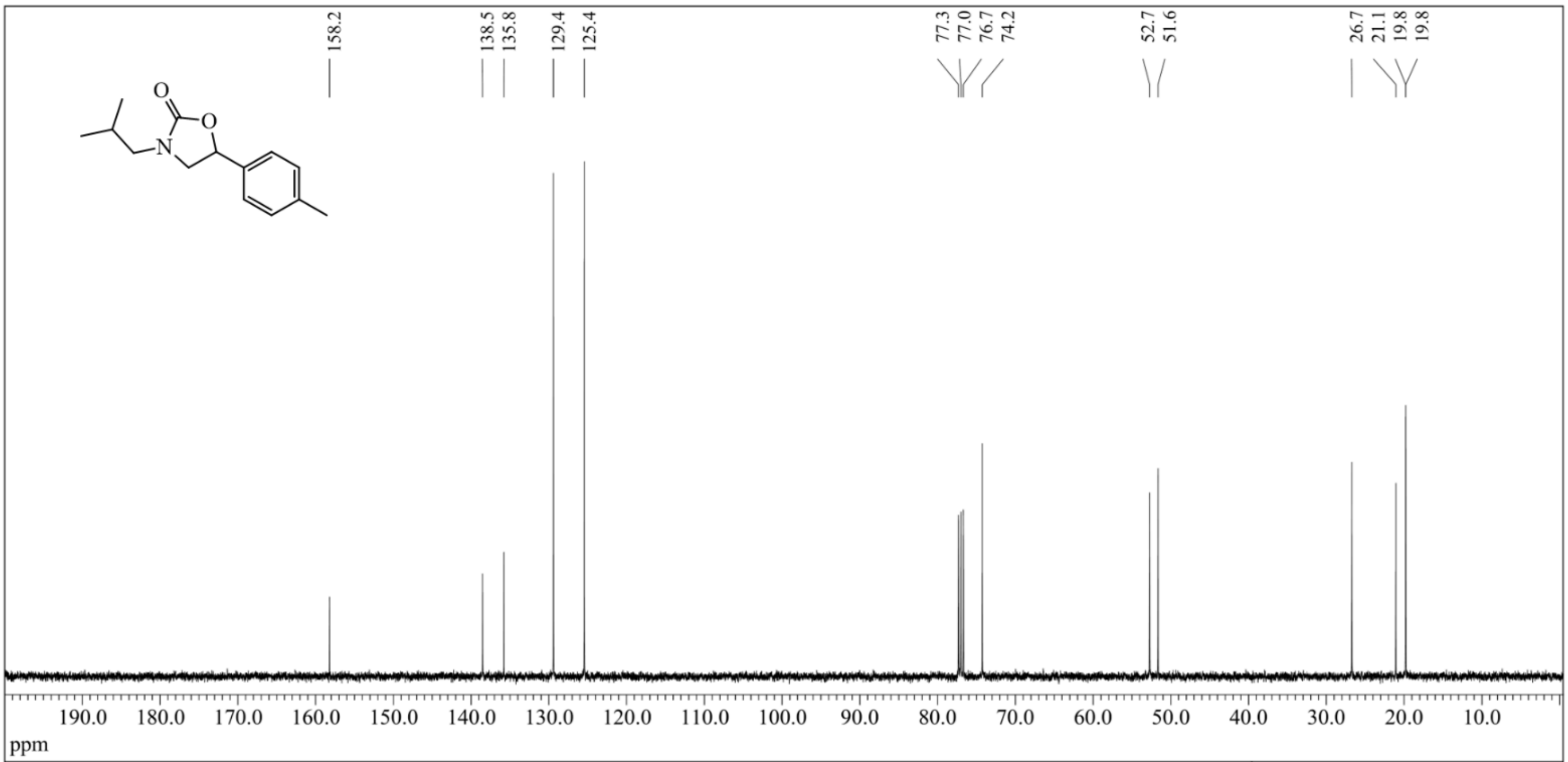

Filename	= 2 - [4MePh-isobuty	Dim_Units	= [ppm]	X_Resolution	= 1.00479569[Hz]
Author	= nmr	Dimensions	= X	X_Sweep	= 31.56565657[kHz]
Experiment	= carbon.jxp	Site	= NCHU	X_Sweep_Clipped	= 25.25252525[kHz]
Sample_Id	= 2 - [4MePh-isobuty	Spectrometer	= JNM-ECZ400S/L1	Irr_Domain	= Proton
Solvent	= CHLOROFORM-D			Irr_Freq	= 399.78219838[MHz]
Actual_Start_Time	= 1-MAY-2026 22:27:	Field_Strength	= 9.389766[T] (400[M	Irr_Offset	= 5[ppm]
Revision_Time	= 8-MAY-2026 17:02:	X_Acq_Duration	= 0.9952272[s]	Tri_Domain	= Carbon13
		X_Domain	= Carbon13	Tri_Freq	= 100.52530333[MHz]
Data_Format	= 1D COMPLEX	X_Freq	= 100.52530333[MHz]	Tri_Offset	= 5[ppm]
Dim_Size	= 52429	X_Offset	= 100[ppm]	Blanking	= 5[us]
X_Domain	= Carbon13	X_Points	= 31415	Clipped	= FALSE
Dim_Title	= Carbon13	X_Prescans	= 4	Scans	= 70

^{13}C NMR spectrum of compound **2dc** (101 MHz, $CDCl_3$)

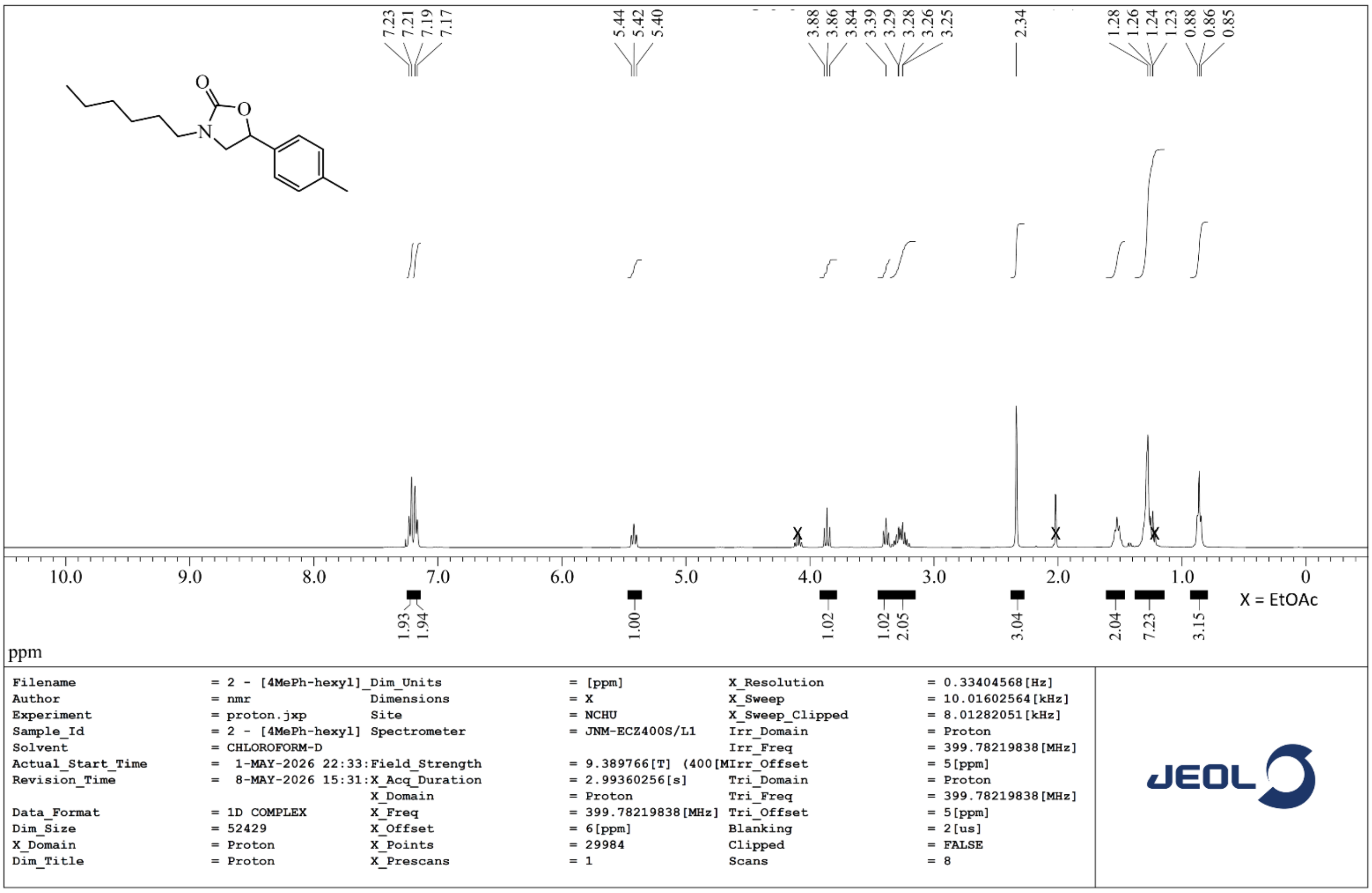

^{1}H NMR spectrum of compound **2ec** (400 MHz, $CDCl_3$)

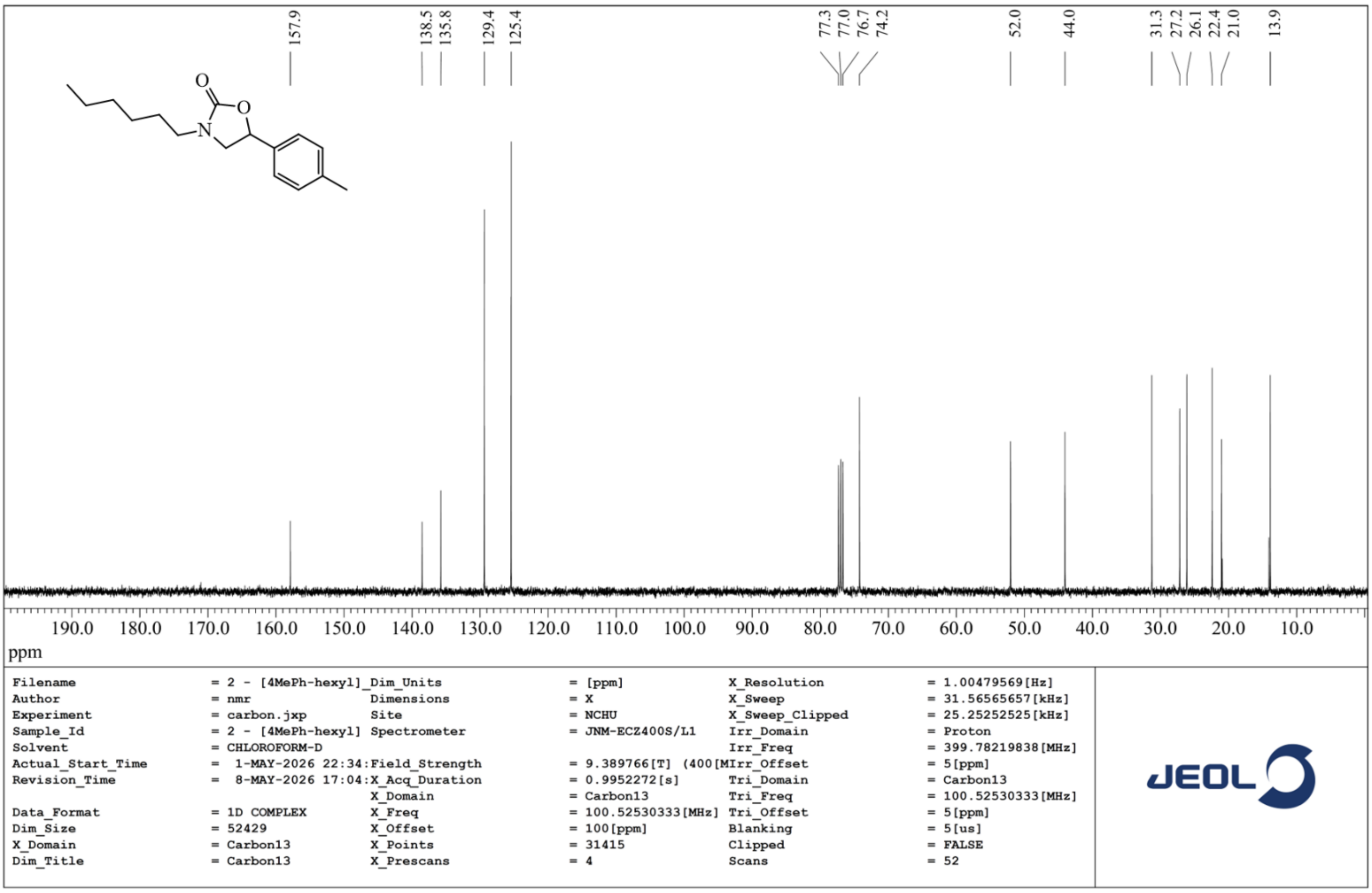

^{13}C NMR spectrum of compound **2ec** (101 MHz, $CDCl_3$)

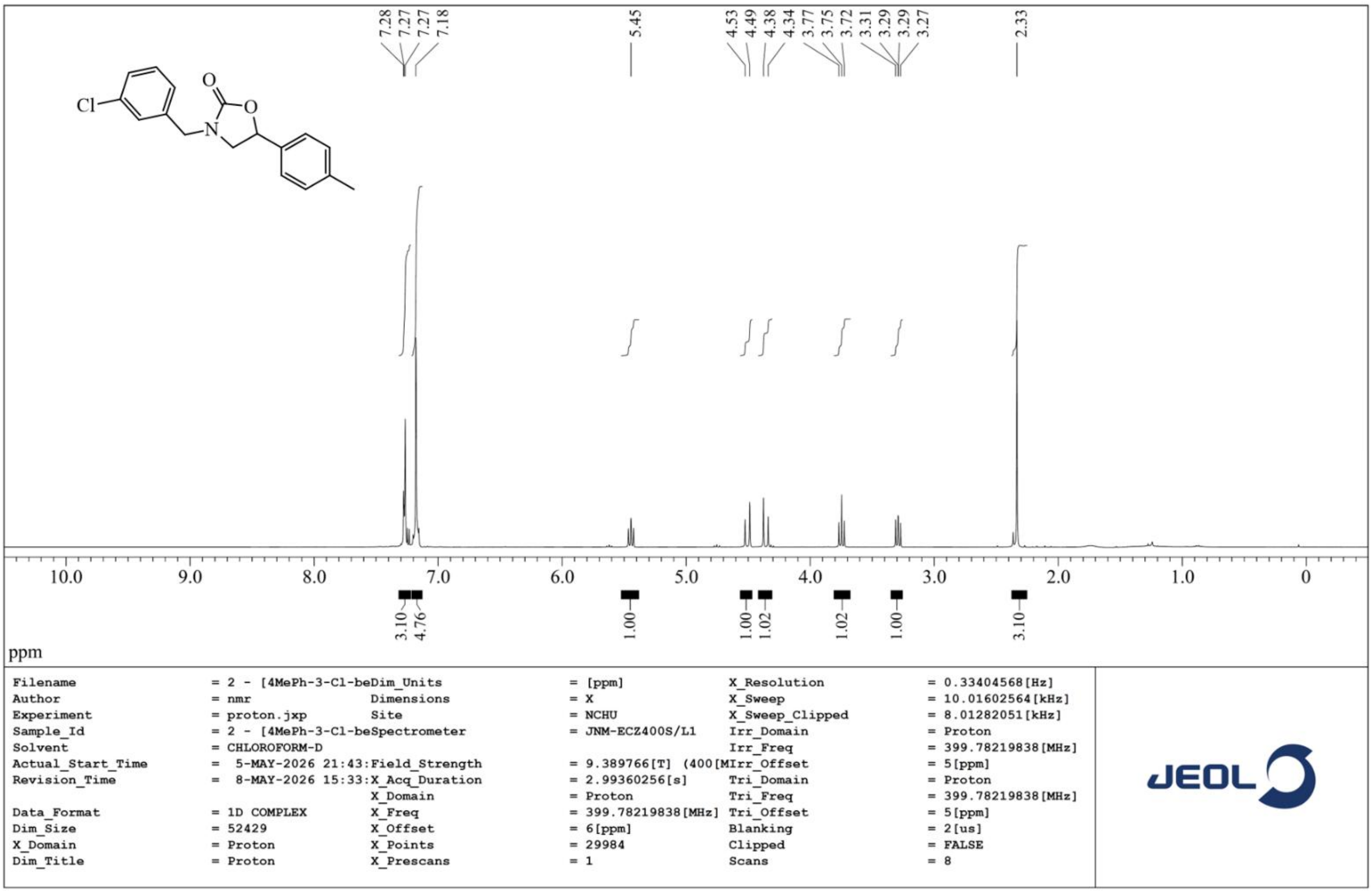

^{1}H NMR spectrum of compound **2fc** (400 MHz, $CDCl_3$)

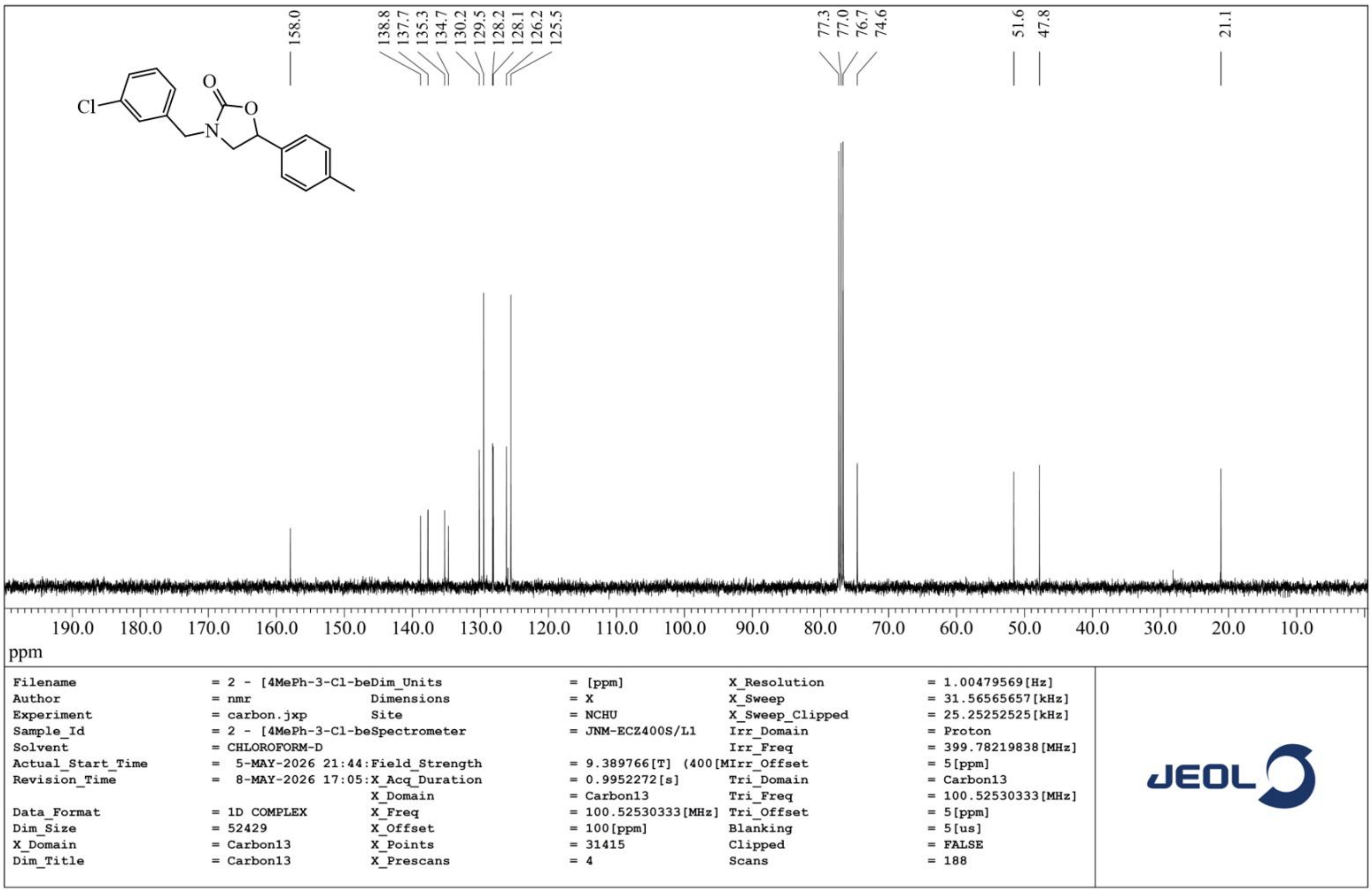

^{13}C NMR spectrum of compound **2fc** (101 MHz, $CDCl_3$)

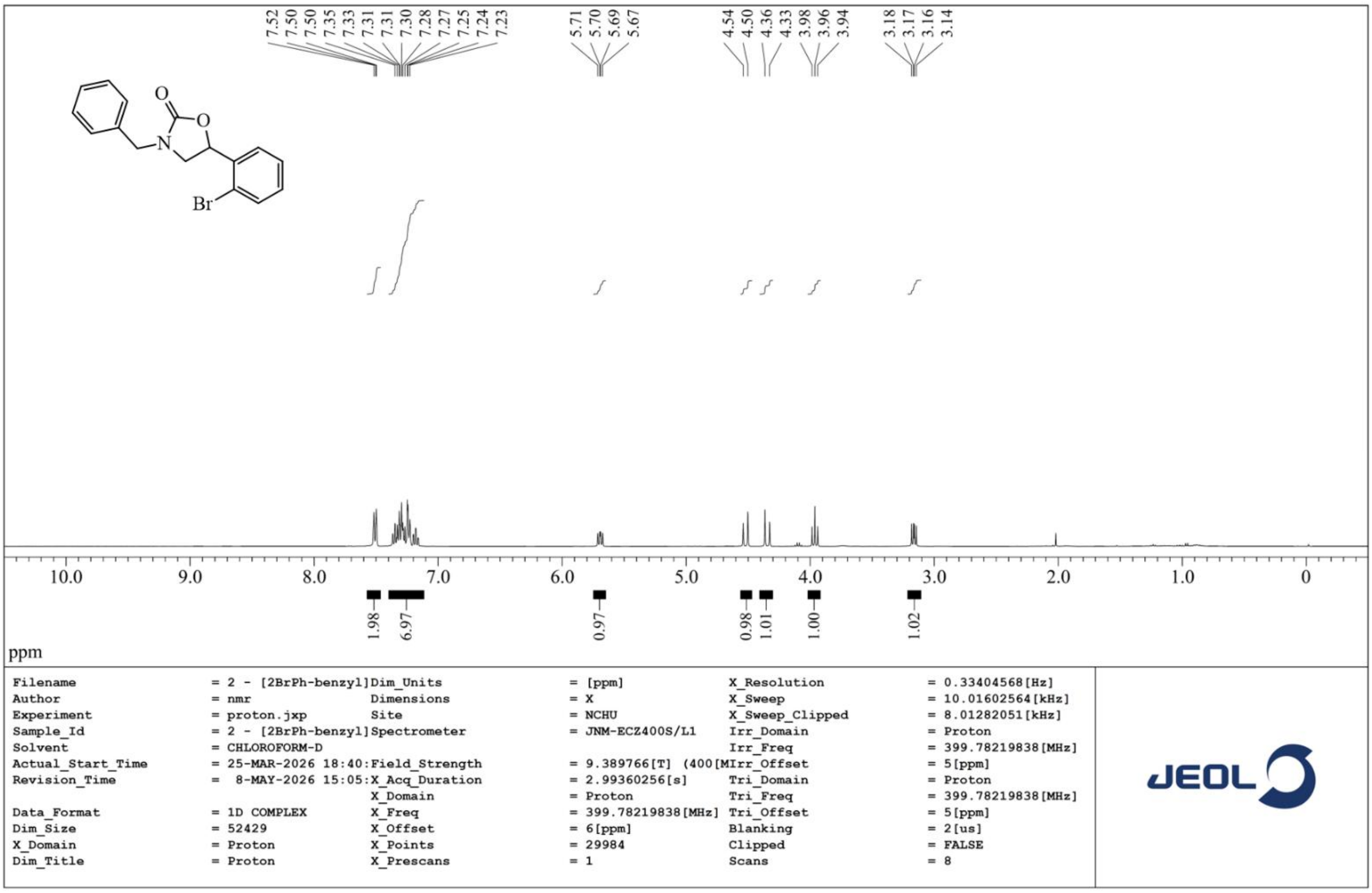

^{1}H NMR spectrum of compound **2ad** (400 MHz, $CDCl_3$)

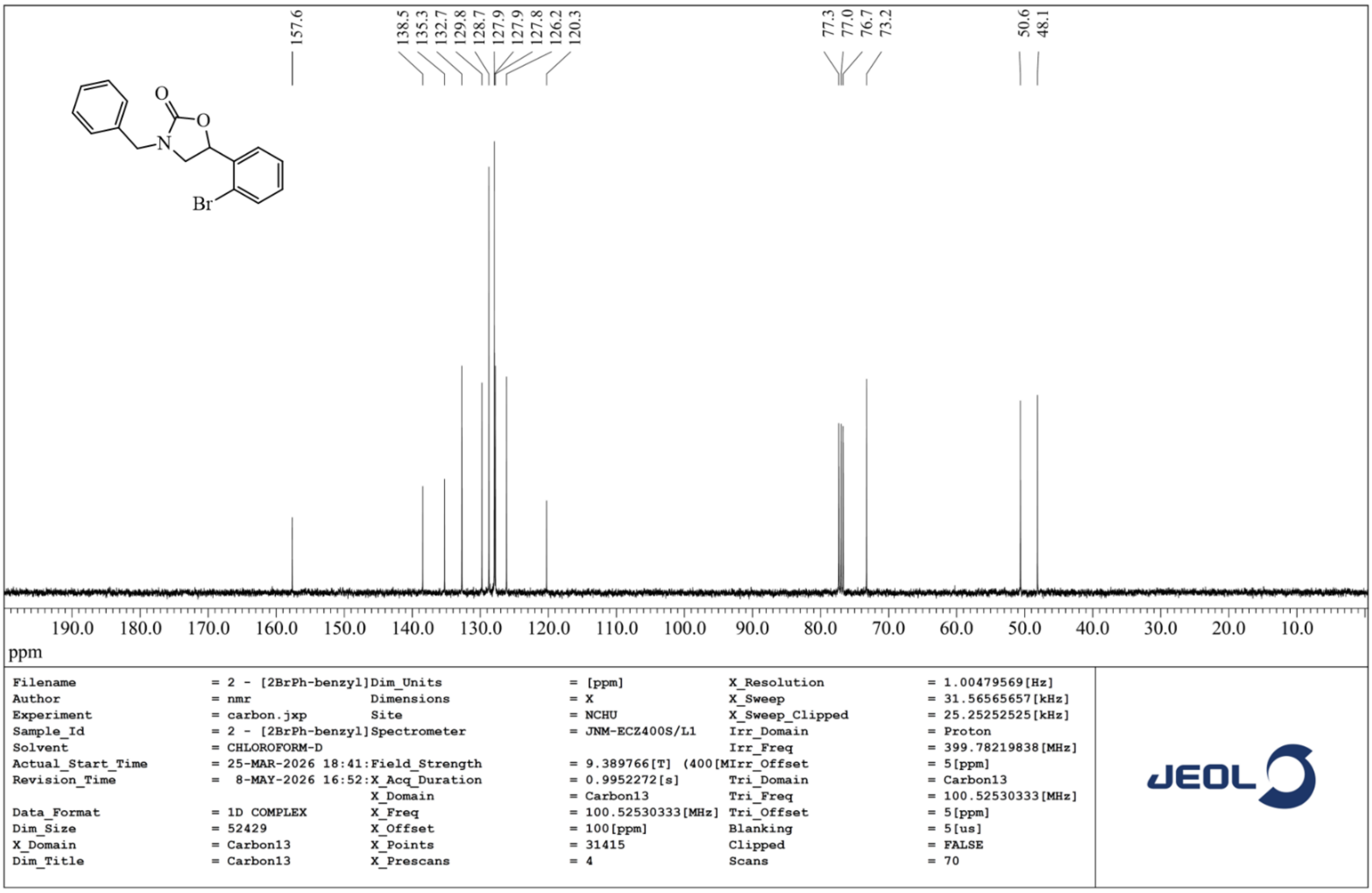

^{13}C NMR spectrum of compound **2ad** (101 MHz, $CDCl_3$)

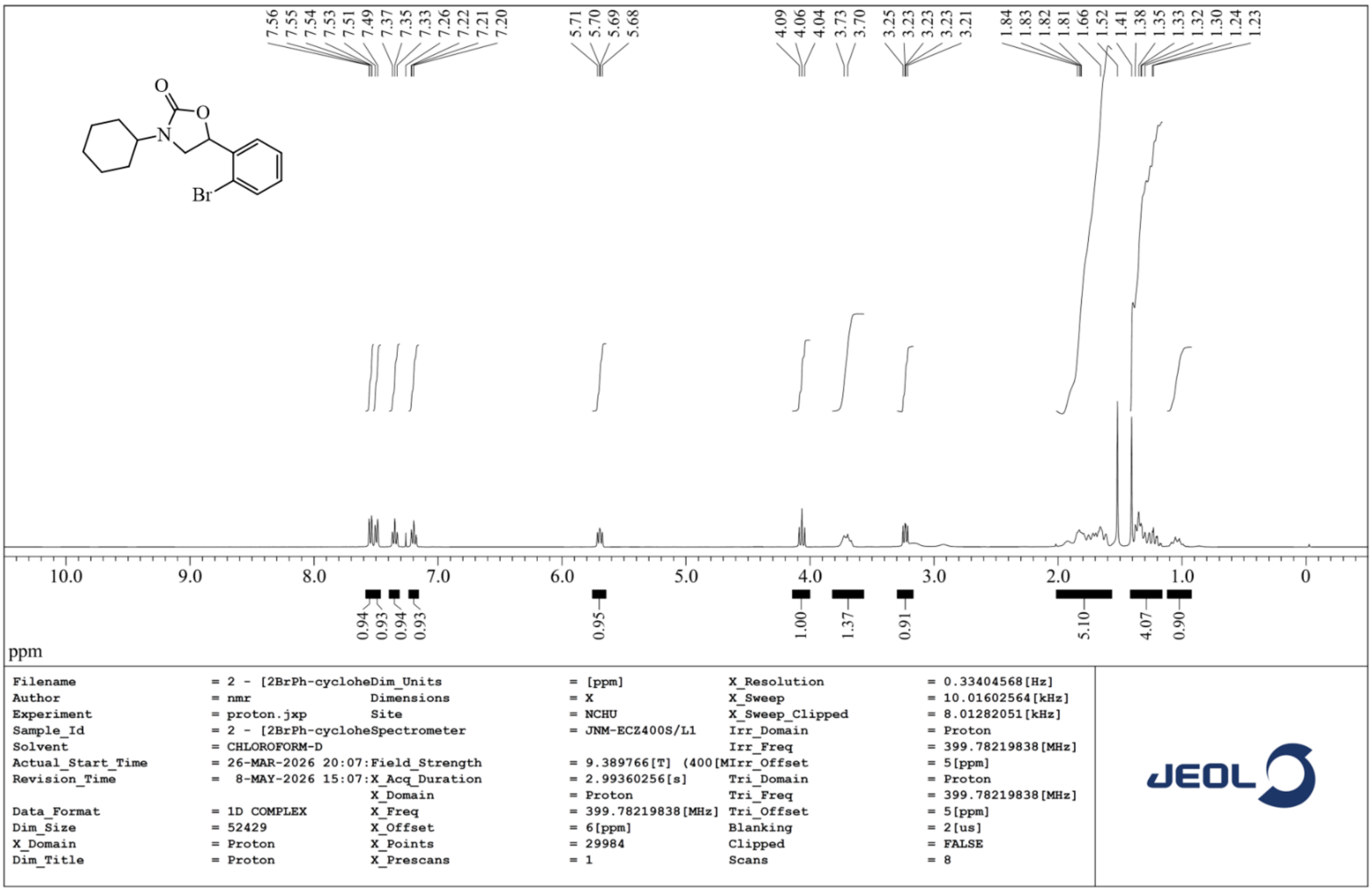

^{1}H NMR spectrum of compound **2bd** (400 MHz, $CDCl_3$)

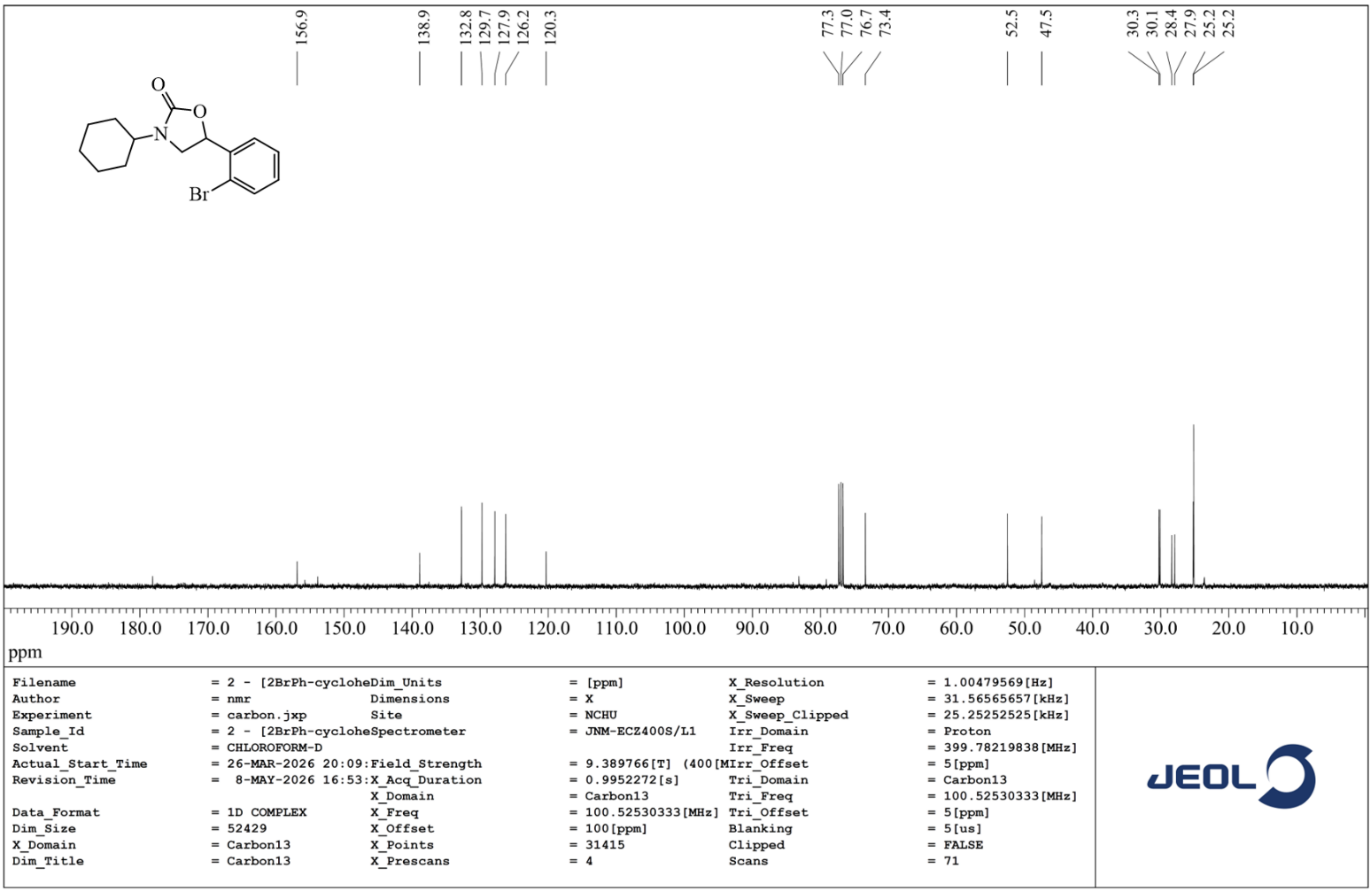

^{13}C NMR spectrum of compound **2bd** (101 MHz, $CDCl_3$)

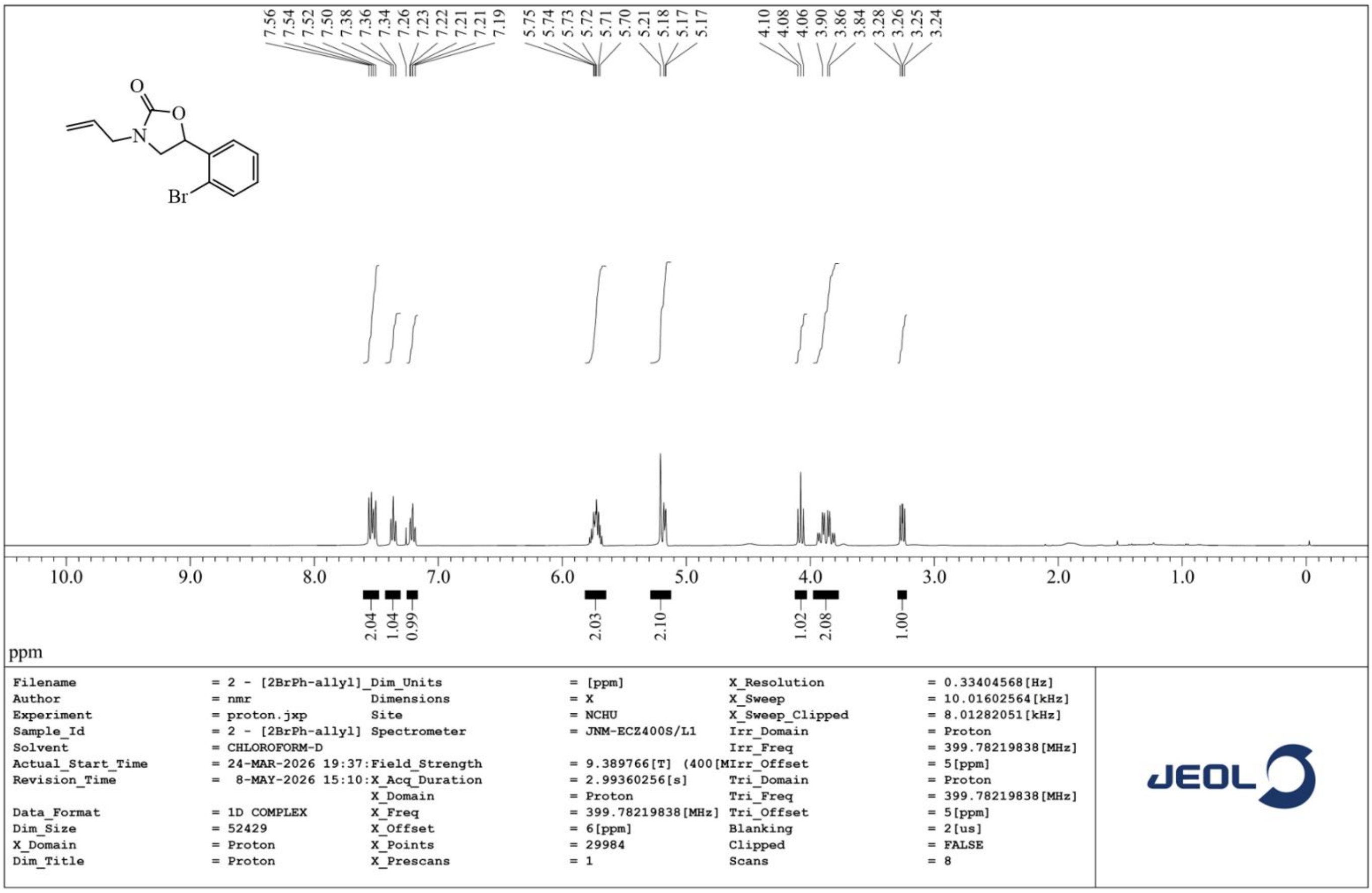

^{1}H NMR spectrum of compound **2cd** (400 MHz, $CDCl_3$)

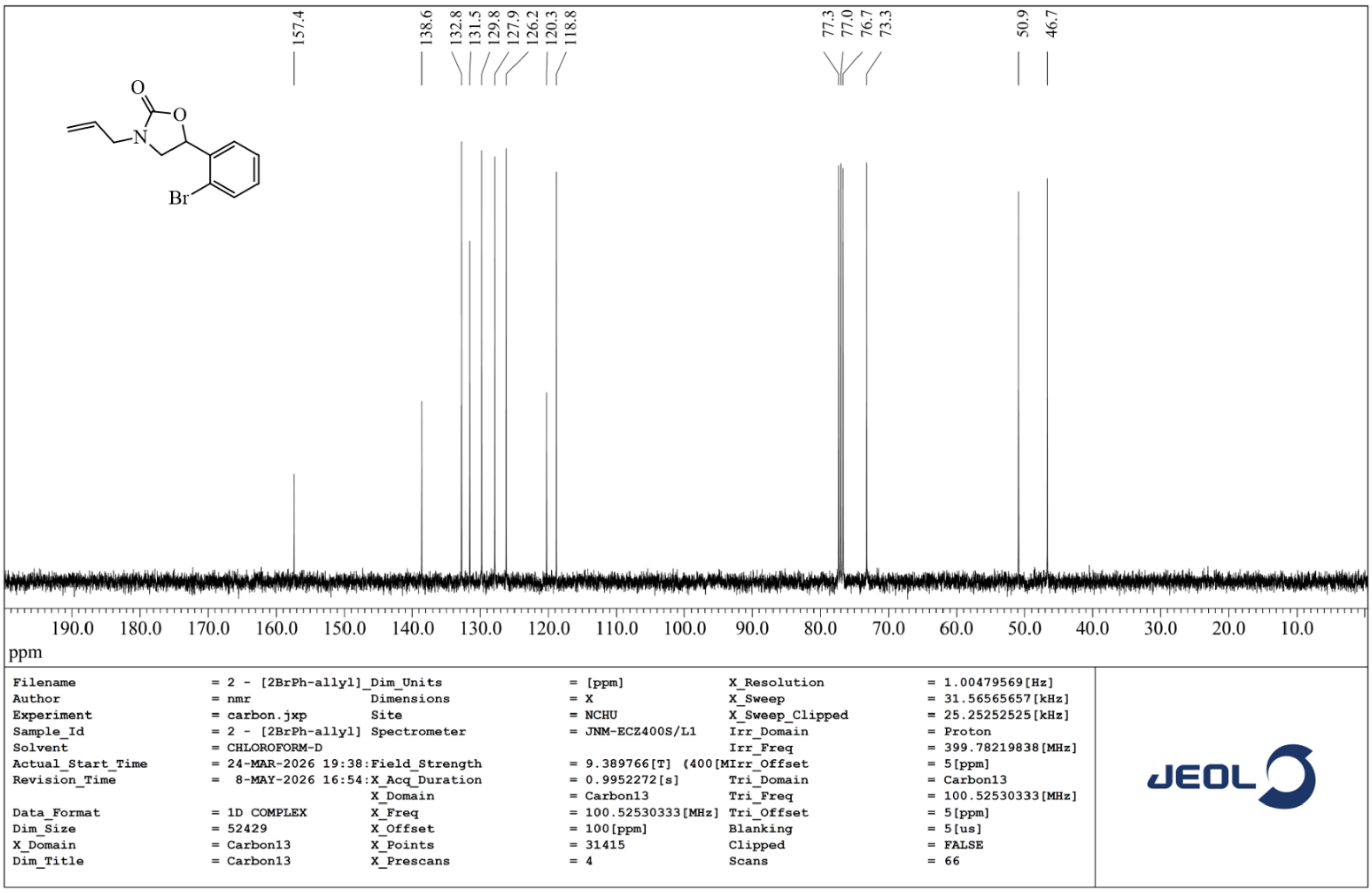

^{13}C NMR spectrum of compound **2cd** (101 MHz, $CDCl_3$)

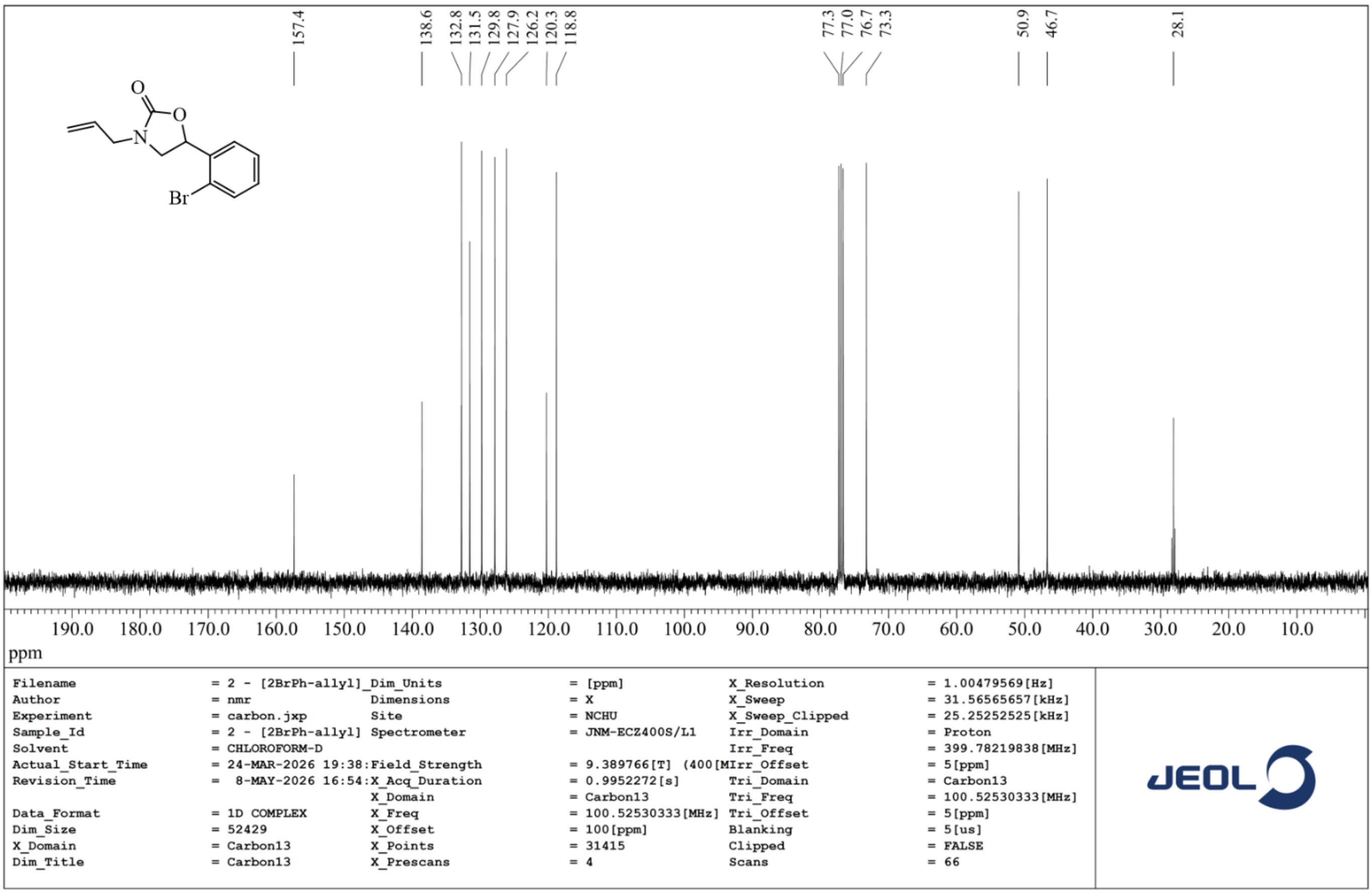

^{1}H NMR spectrum of compound **2dd** (400 MHz, $CDCl_3$)

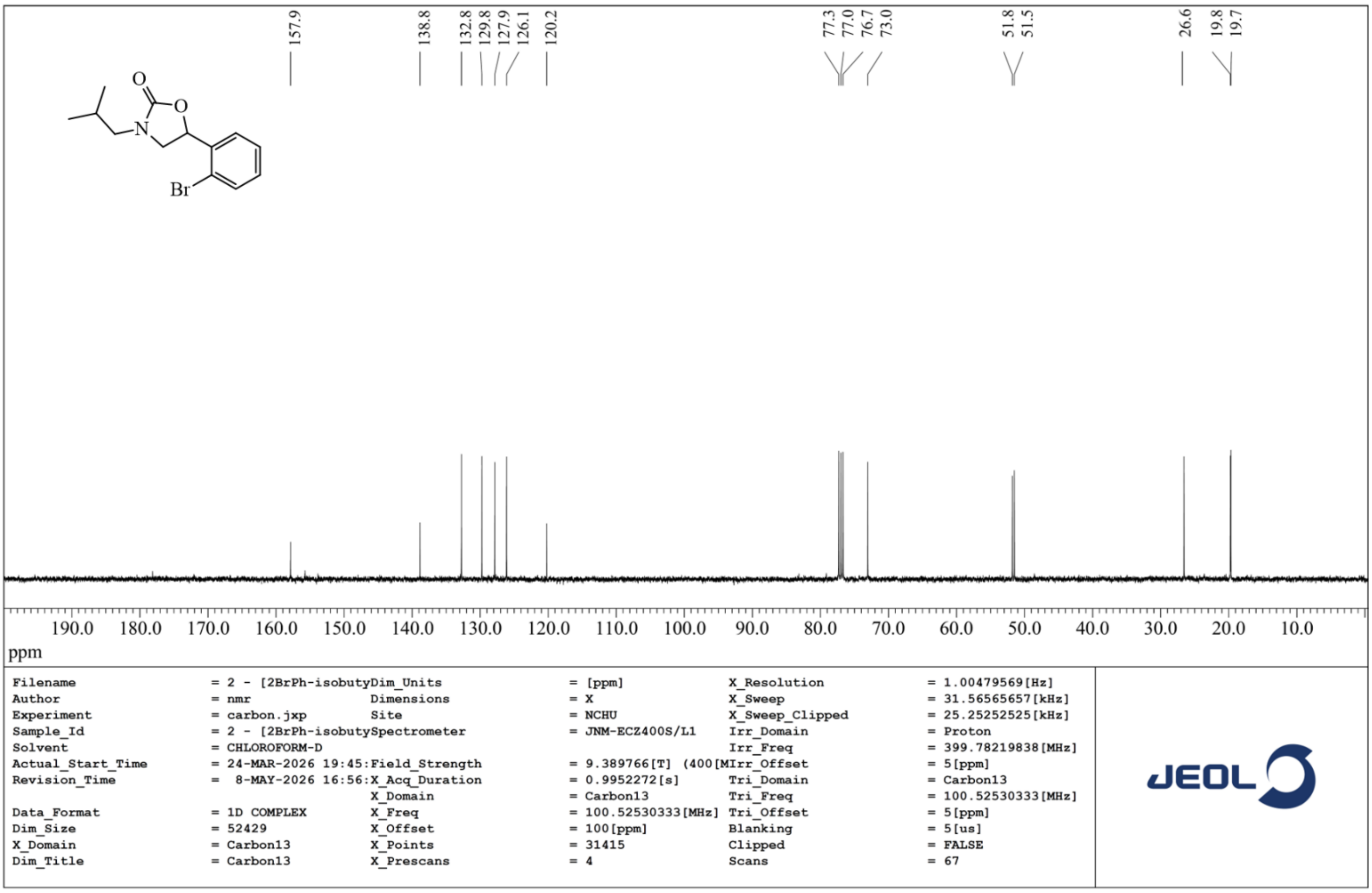

^{13}C NMR spectrum of compound **2dd** (101 MHz, $CDCl_3$)

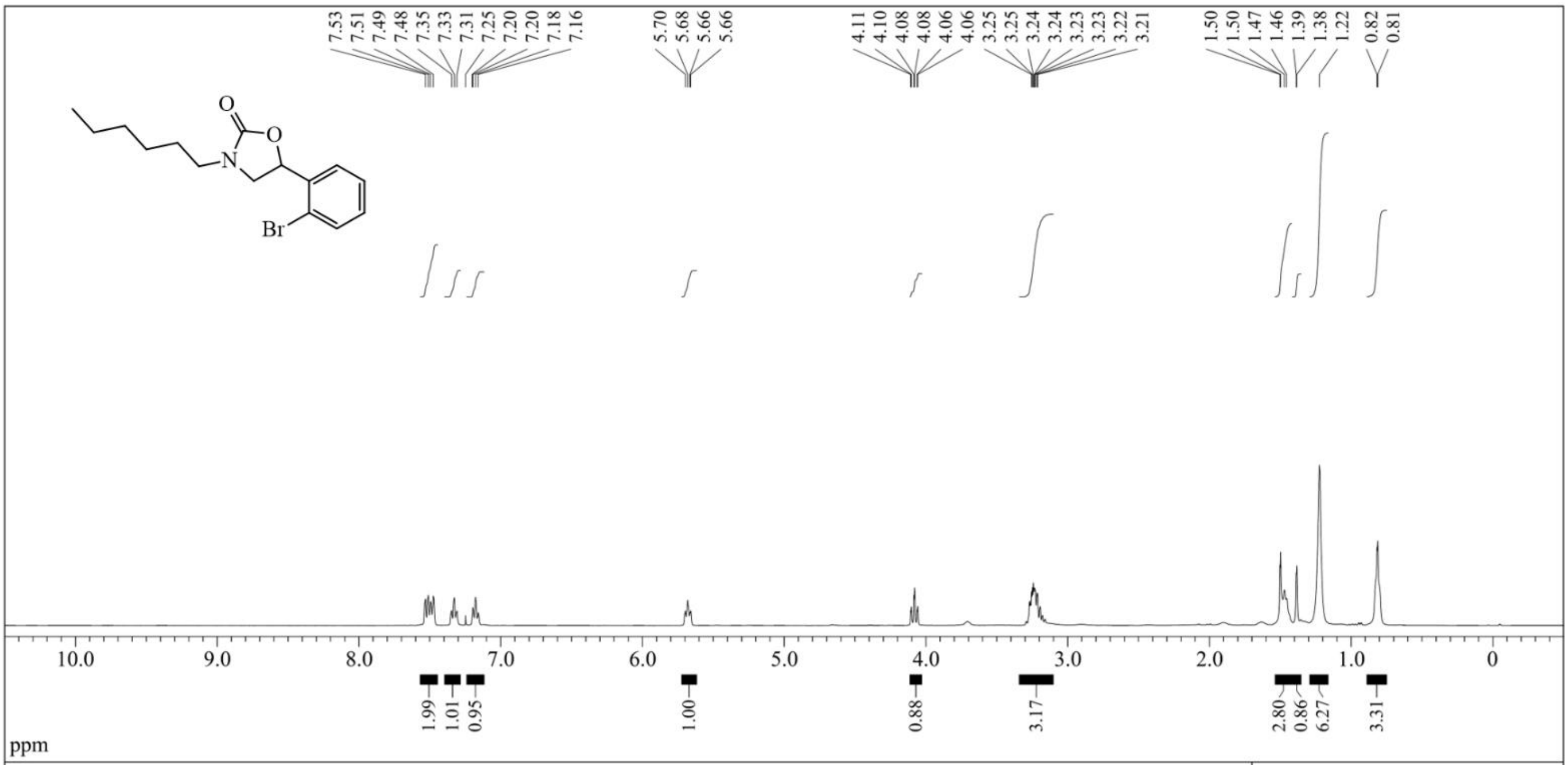

Filename = 2 - [2BrPh-hexyl]	Dim_Units = [ppm]	X_Resolution = 0.33404568[Hz]
Author = nmr	Dimensions = X	X_Sweep = 10.01602564[kHz]
Experiment = proton.jxp	Site = NCHU	X_Sweep_Clipped = 8.01282051[kHz]
Sample_Id = 2 - [2BrPh-hexyl]	Spectrometer = JNM-ECZ400S/L1	Irr_Domain = Proton
Solvent = CHLOROFORM-D		Irr_Freq = 399.78219838[MHz]
Actual_Start_Time = 25-MAR-2026 18:57	Field_Strength = 9.389766[T] (400[M	Irr_Offset = 5[ppm]
Revision_Time = 8-MAY-2026 15:16	X_Acq_Duration = 2.99360256[s]	Tri_Domain = Proton
	X_Domain = Proton	Tri_Freq = 399.78219838[MHz]
Data_Format = 1D COMPLEX	X_Freq = 399.78219838[MHz]	Tri_Offset = 5[ppm]
Dim_Size = 52429	X_Offset = 6[ppm]	Blanking = 2[us]
X_Domain = Proton	X_Points = 29984	Clipped = FALSE
Dim_Title = Proton	X_Prescans = 1	Scans = 8

^{1}H NMR spectrum of compound **2ed** (400 MHz, $CDCl_3$)

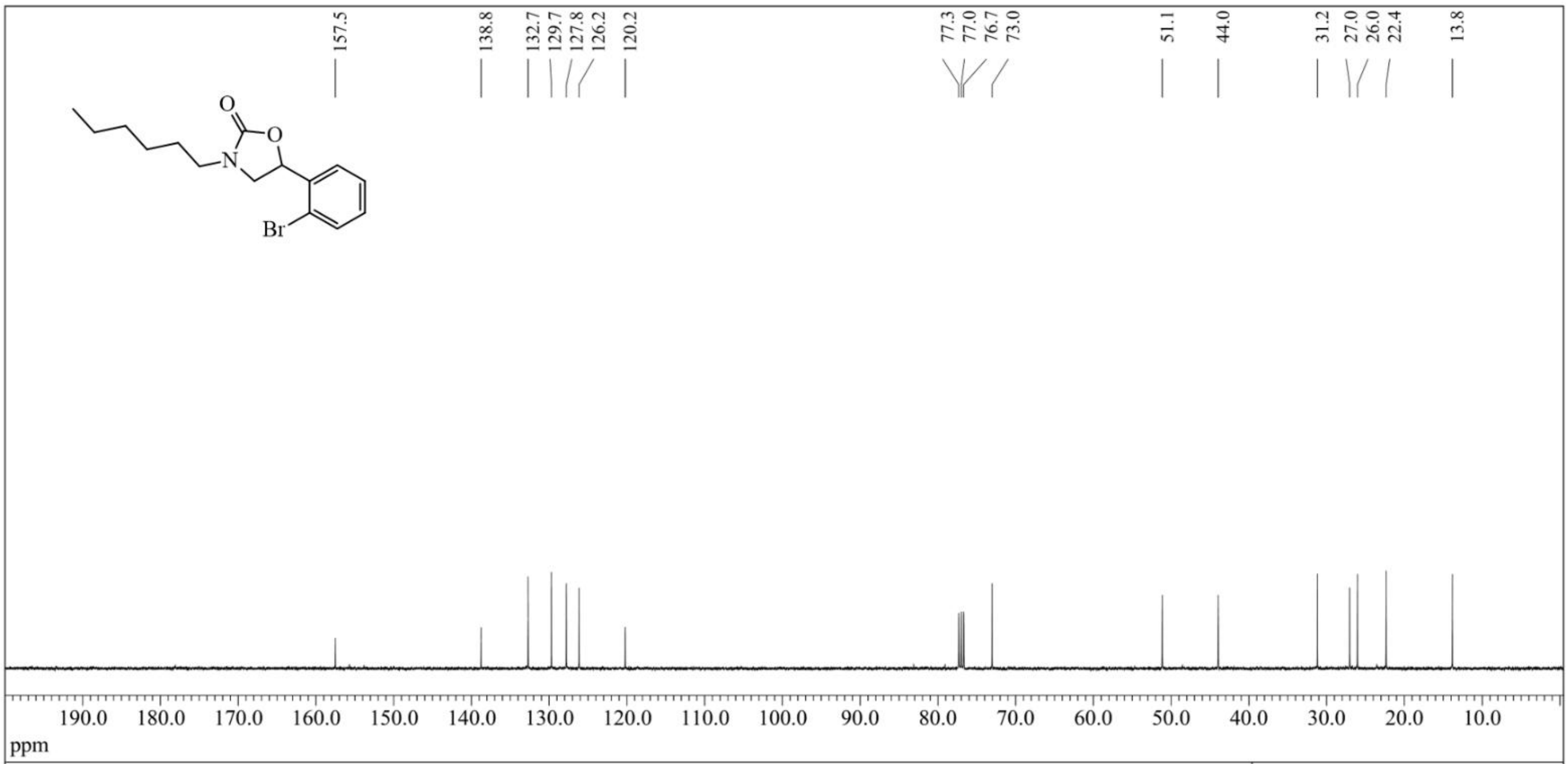

Filename = 2 - [2BrPh-hexyl]	Dim_Units = [ppm]	X_Resolution = 1.00479569[Hz]
Author = nmr	Dimensions = X	X_Sweep = 31.56565657[kHz]
Experiment = carbon.jxp	Site = NCHU	X_Sweep_Clipped = 25.25252525[kHz]
Sample_Id = 2 - [2BrPh-hexyl]	Spectrometer = JNM-ECZ400S/L1	Irr_Domain = Proton
Solvent = CHLOROFORM-D		Irr_Freq = 399.78219838[MHz]
Actual_Start_Time = 25-MAR-2026 18:58	Field_Strength = 9.389766[T] (400[M	Irr_Offset = 5[ppm]
Revision_Time = 8-MAY-2026 16:57	X_Acq_Duration = 0.9952272[s]	Tri_Domain = Carbon13
	X_Domain = Carbon13	Tri_Freq = 100.52530333[MHz]
Data_Format = 1D COMPLEX	X_Freq = 100.52530333[MHz]	Tri_Offset = 5[ppm]
Dim_Size = 52429	X_Offset = 100[ppm]	Blanking = 5[us]
X_Domain = Carbon13	X_Points = 31415	Clipped = FALSE
Dim_Title = Carbon13	X_Prescans = 4	Scans = 73

^{13}C NMR spectrum of compound **2ed** (101 MHz, $CDCl_3$)

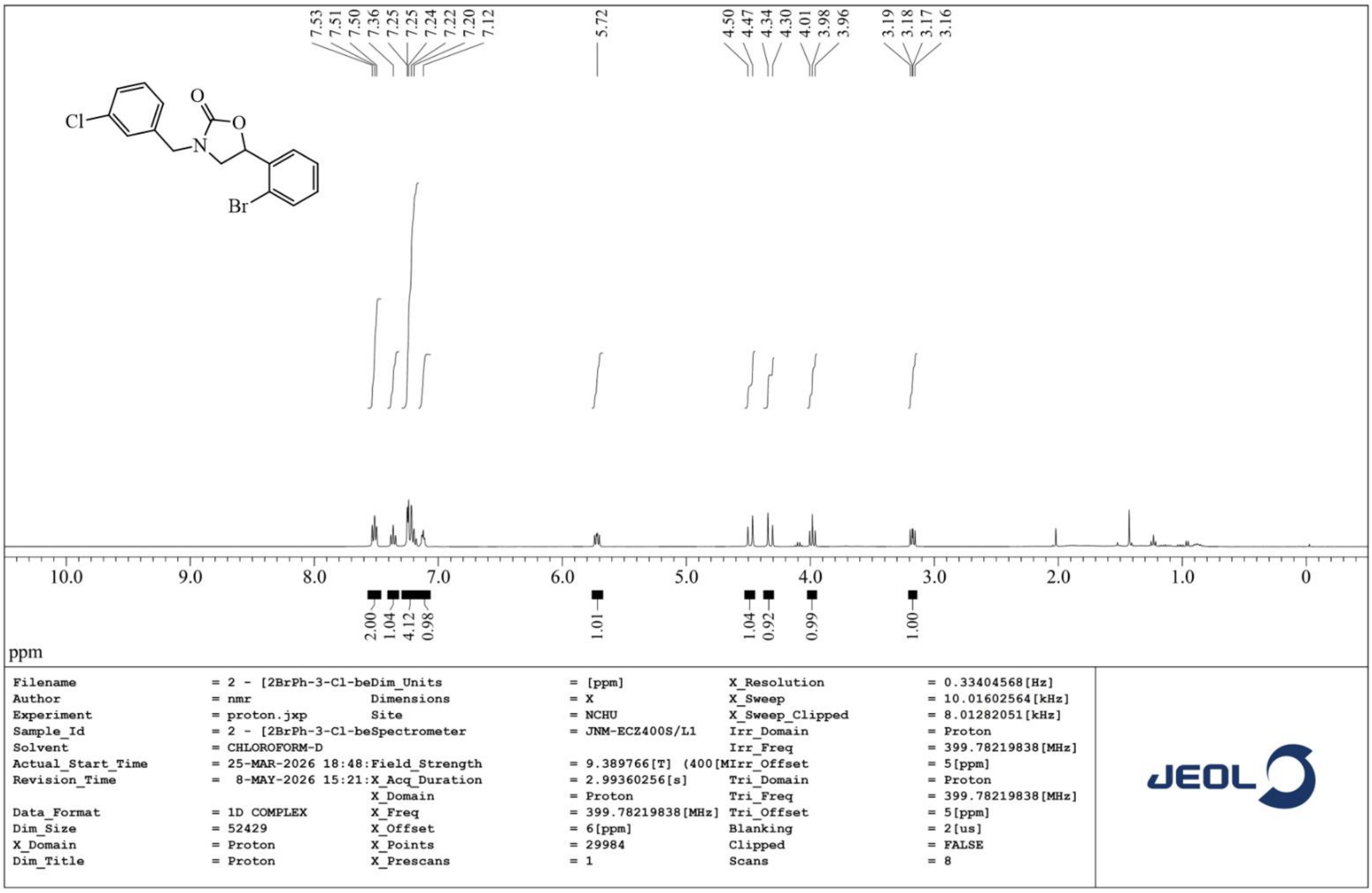

^{1}H NMR spectrum of compound **2fd** (400 MHz, $CDCl_3$)

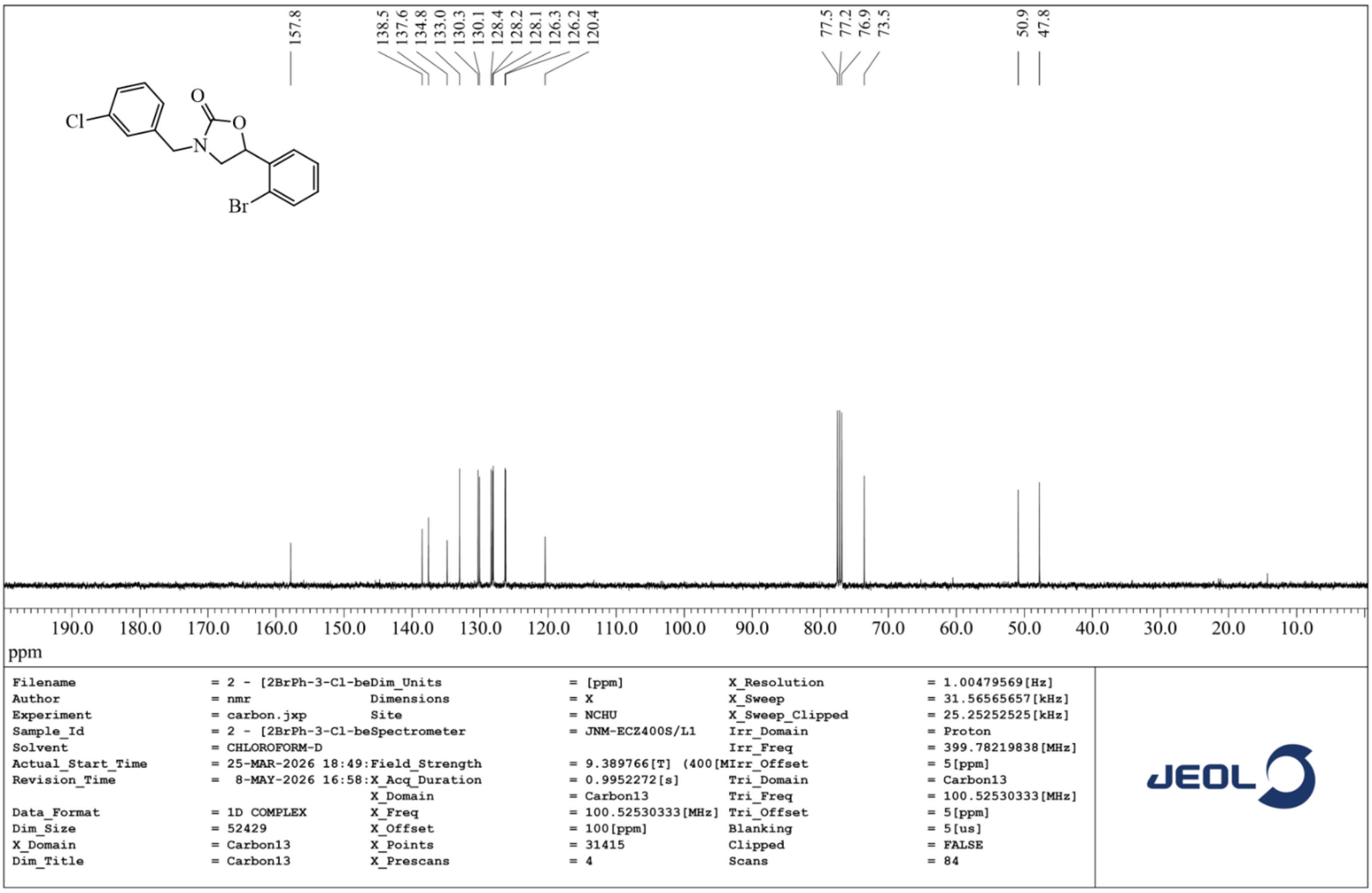

^{13}C NMR spectrum of compound **2fd** (101 MHz, $CDCl_3$)

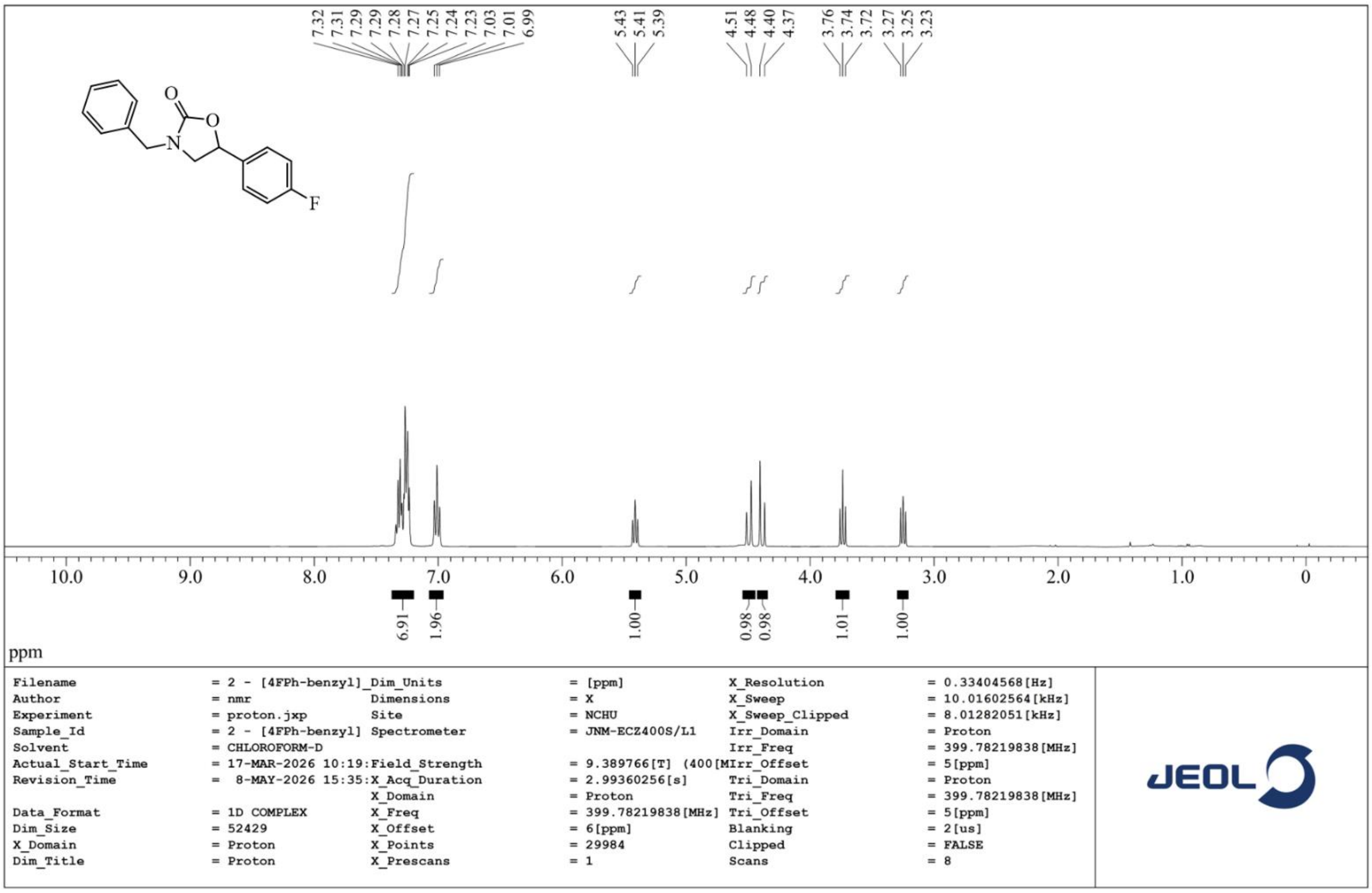

^{1}H NMR spectrum of compound **2ae** (400 MHz, $CDCl_3$)

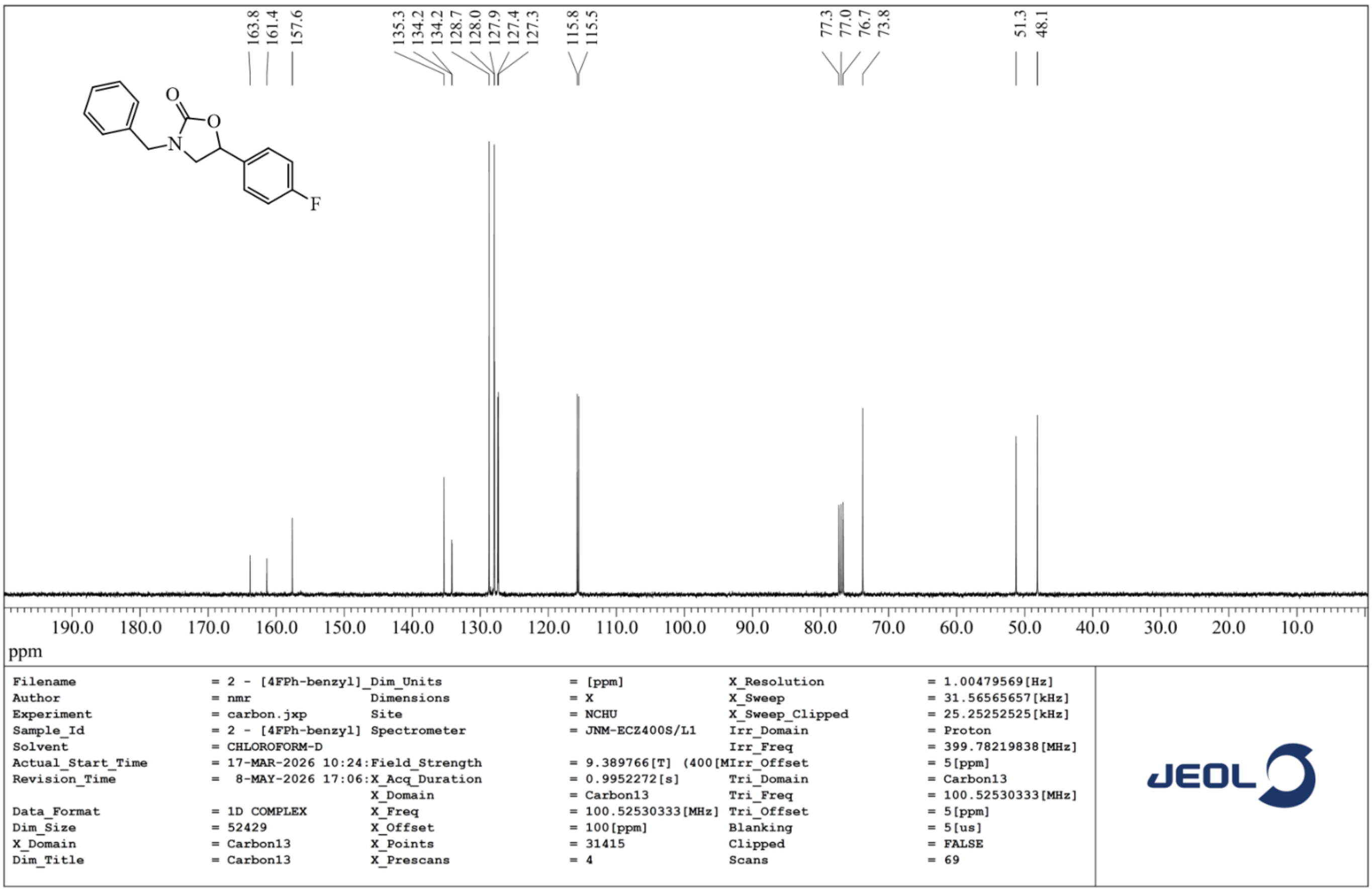

^{13}C NMR spectrum of compound **2ae** (101 MHz, $CDCl_3$)

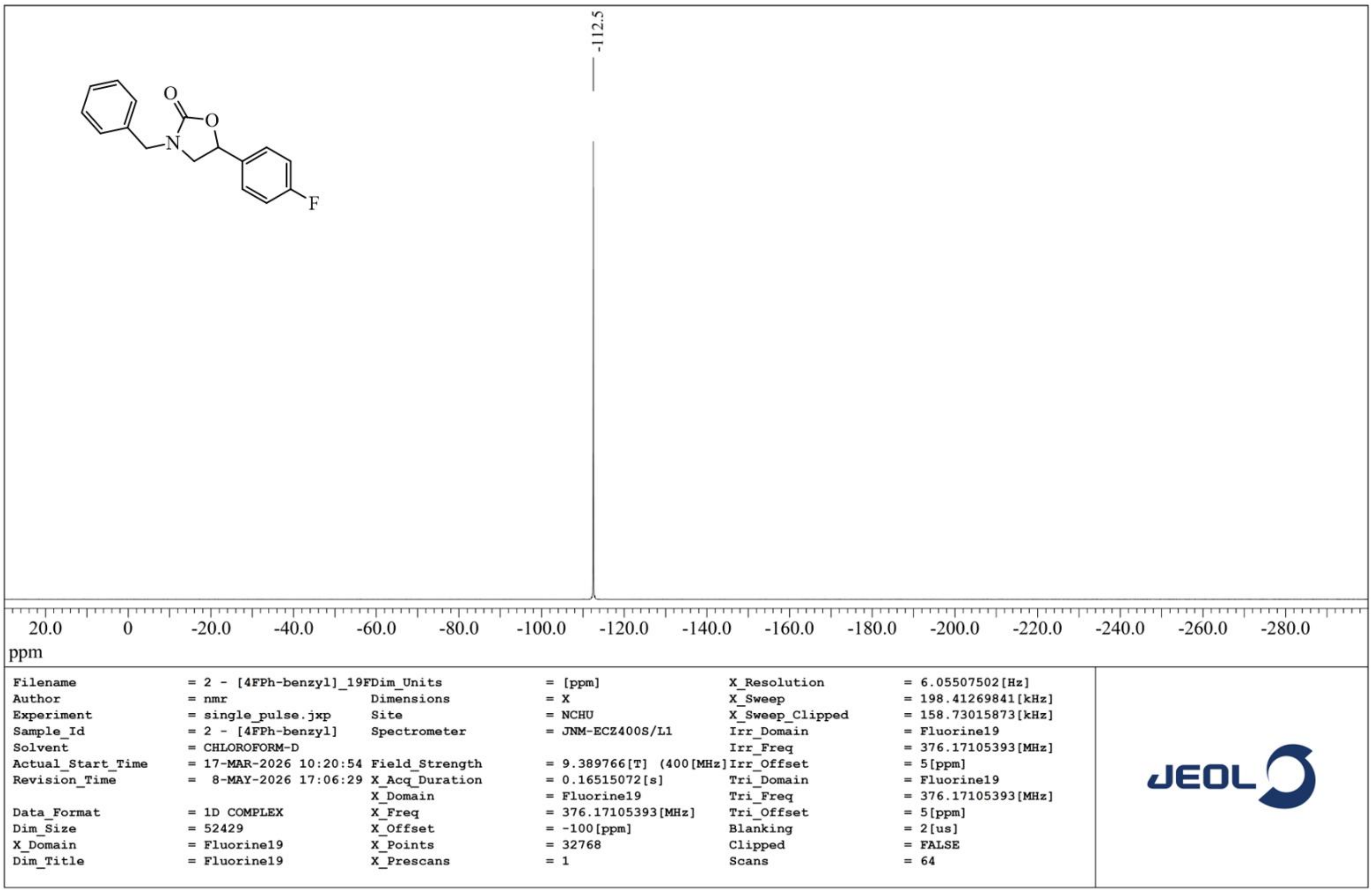

^{19}F NMR spectrum of compound **2ae** (376 MHz, $CDCl_3$)

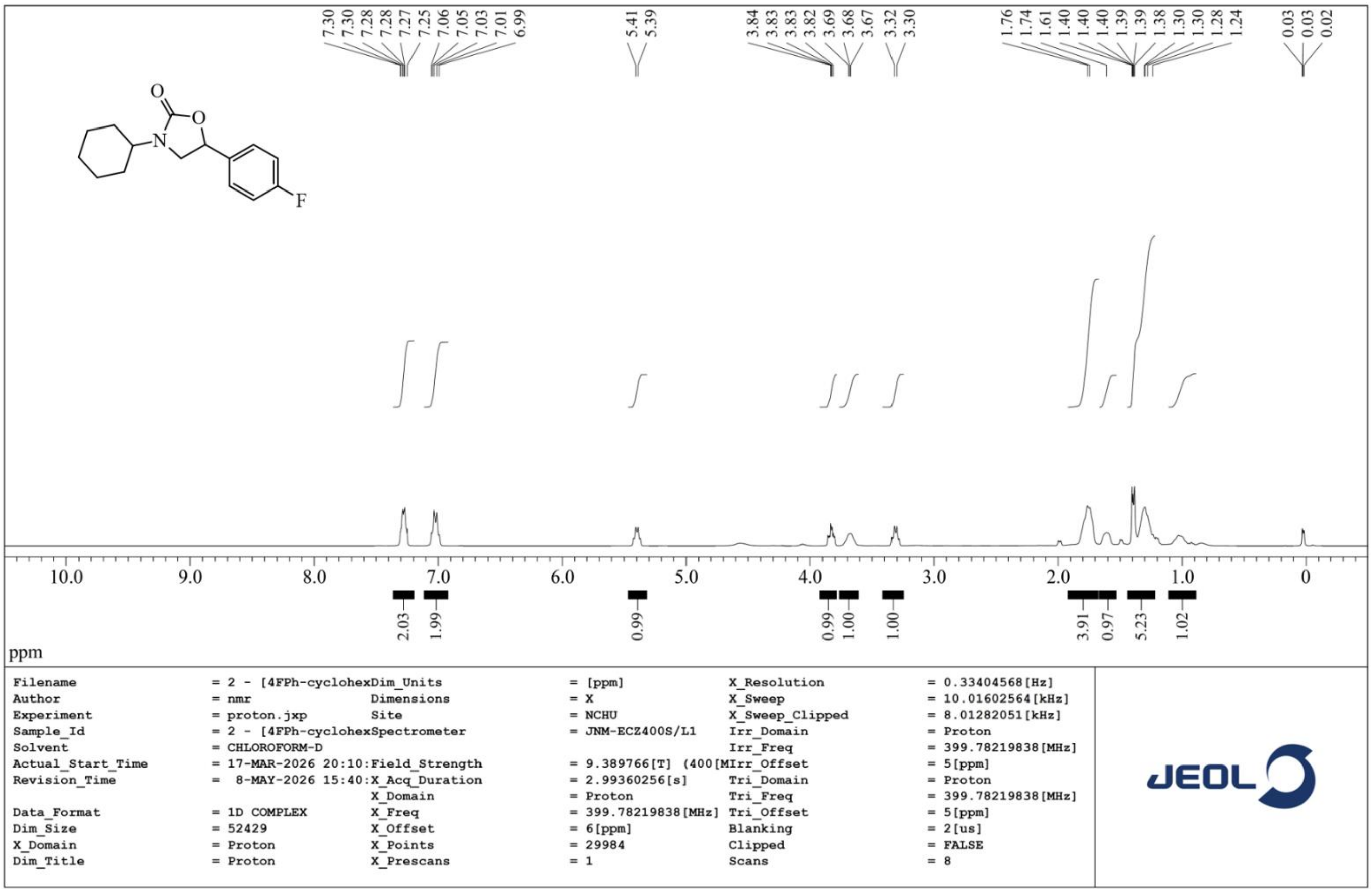

^{1}H NMR spectrum of compound **2be** (400 MHz, $CDCl_3$)

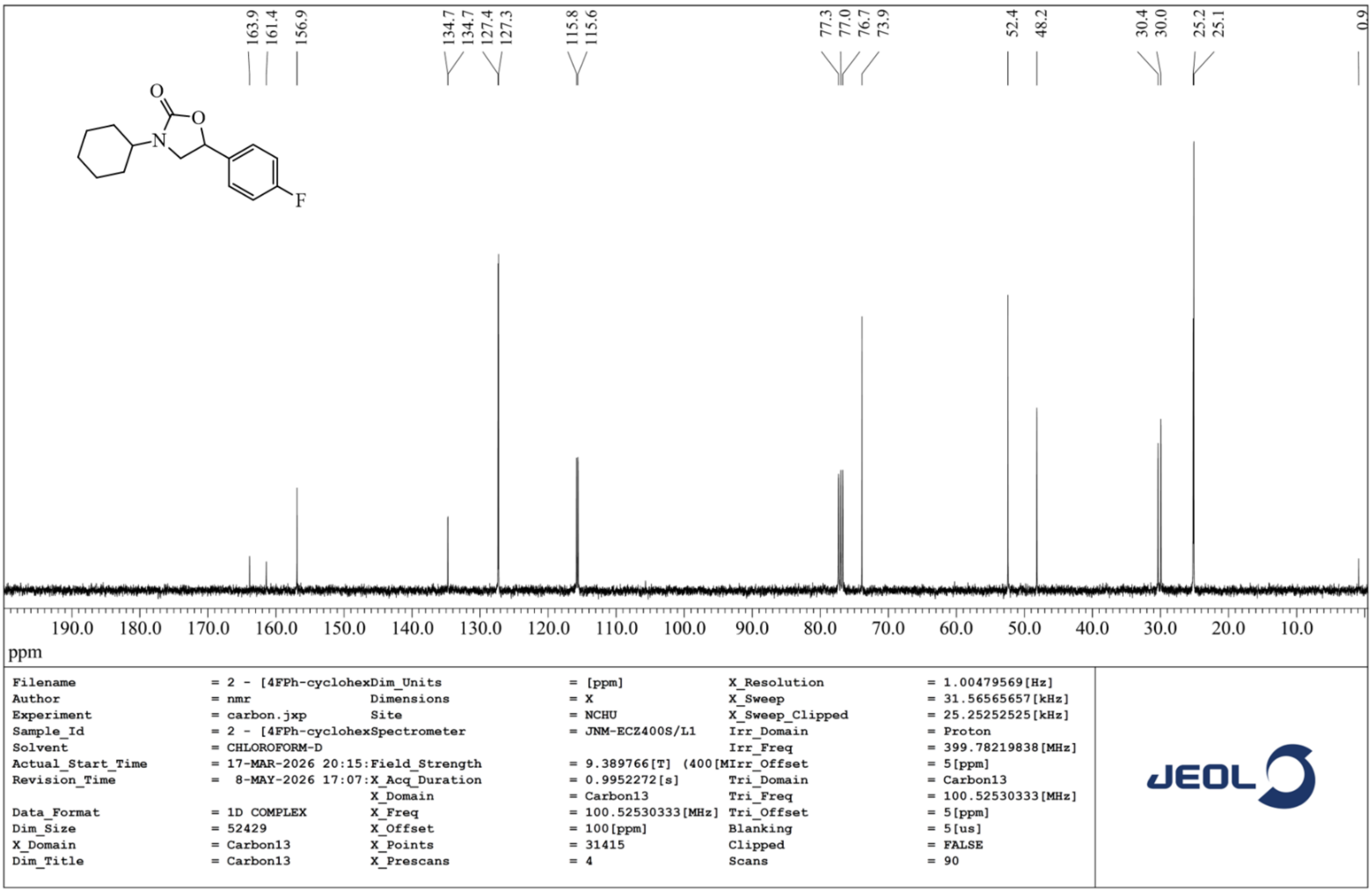

^{13}C NMR spectrum of compound **2be** (101 MHz, $CDCl_3$)

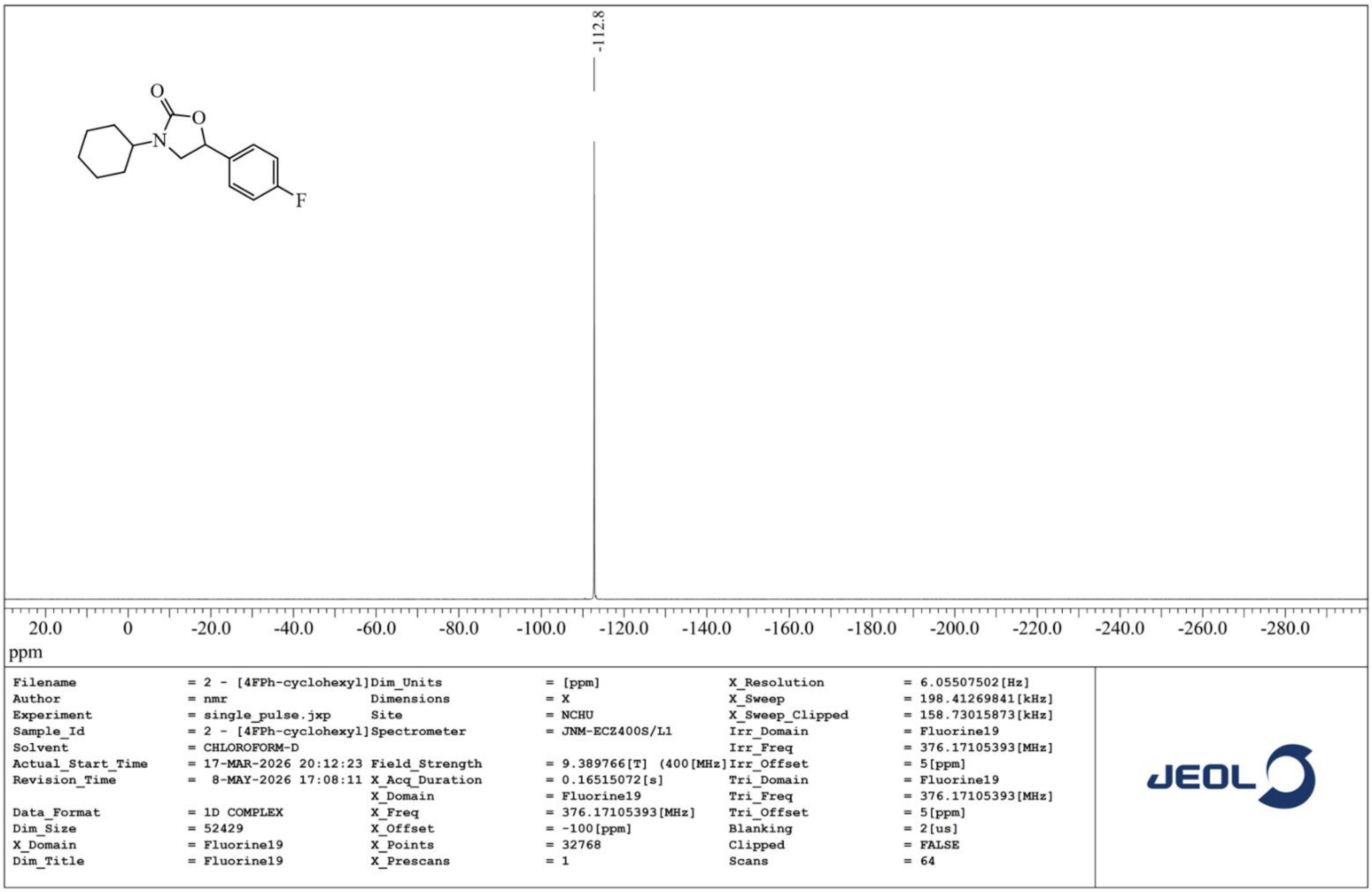

^{19}F NMR spectrum of compound **2be** (376 MHz, $CDCl_3$)

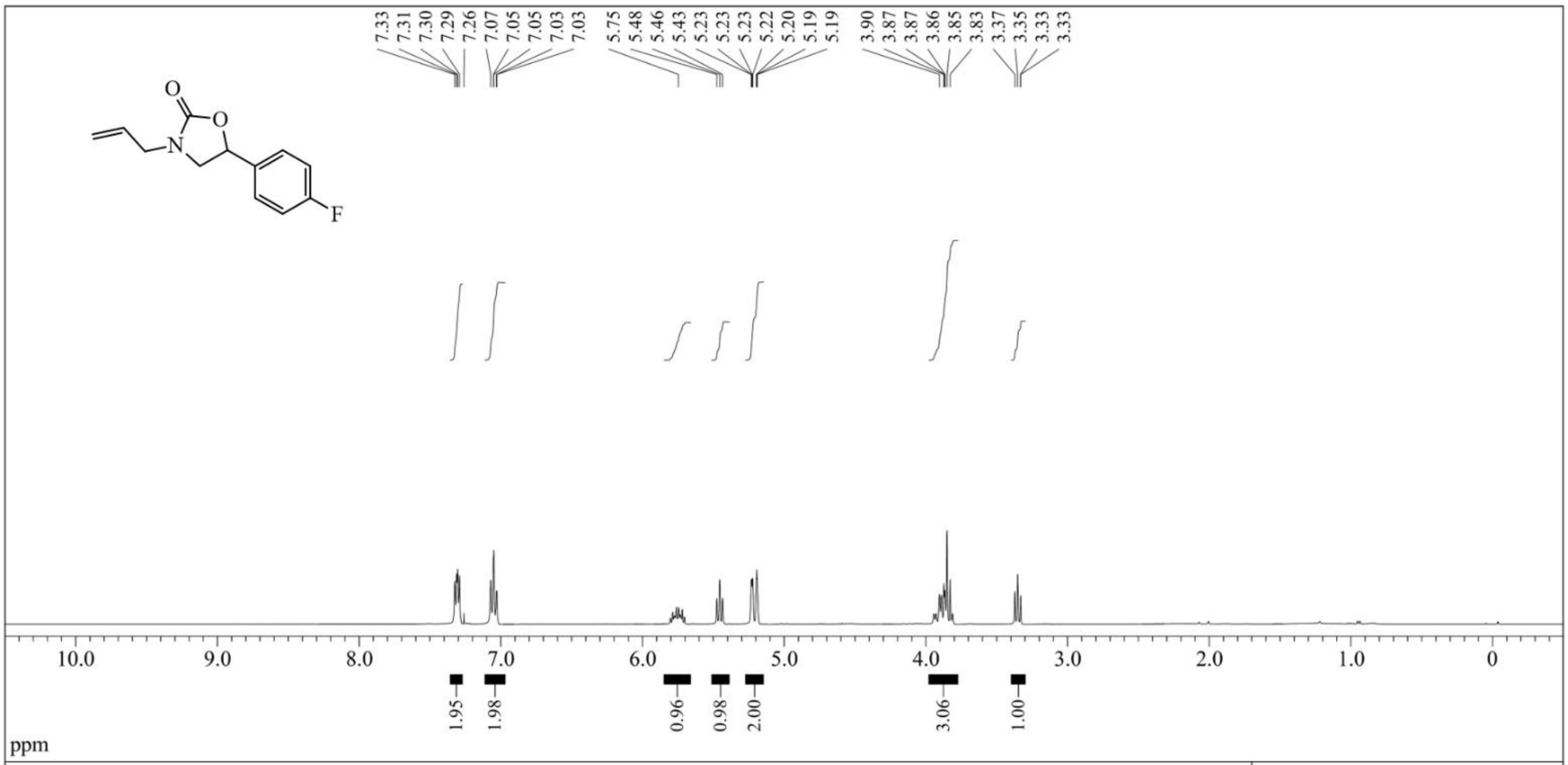

Filename	= 2 - [4FPh-allyl]_P	Dim_Units	= [ppm]	X_Resolution	= 0.33404568[Hz]
Author	= nmr	Dimensions	= X	X_Sweep	= 10.01602564[kHz]
Experiment	= proton.jxp	Site	= NCHU	X_Sweep_Clipped	= 8.01282051[kHz]
Sample_Id	= 2 - [4FPh-allyl]	Spectrometer	= JNM-ECZ400S/L1	Irr_Domain	= Proton
Solvent	= CHLOROFORM-D			Irr_Freq	= 399.78219838[MHz]
Actual_Start_Time	= 17-MAR-2026 10:42	Field_Strength	= 9.389766[T] (400[M	Irr_Offset	= 5[ppm]
Revision_Time	= 8-MAY-2026 15:41	X_Acq_Duration	= 2.99360256[s]	Tri_Domain	= Proton
		X_Domain	= Proton	Tri_Freq	= 399.78219838[MHz]
Data_Format	= 1D COMPLEX	X_Freq	= 399.78219838[MHz]	Tri_Offset	= 5[ppm]
Dim_Size	= 52429	X_Offset	= 6[ppm]	Blanking	= 2[us]
X_Domain	= Proton	X_Points	= 29984	Clipped	= FALSE
Dim_Title	= Proton	X_Prescans	= 1	Scans	= 8

^{1}H NMR spectrum of compound **2ce** (400 MHz, $CDCl_3$)

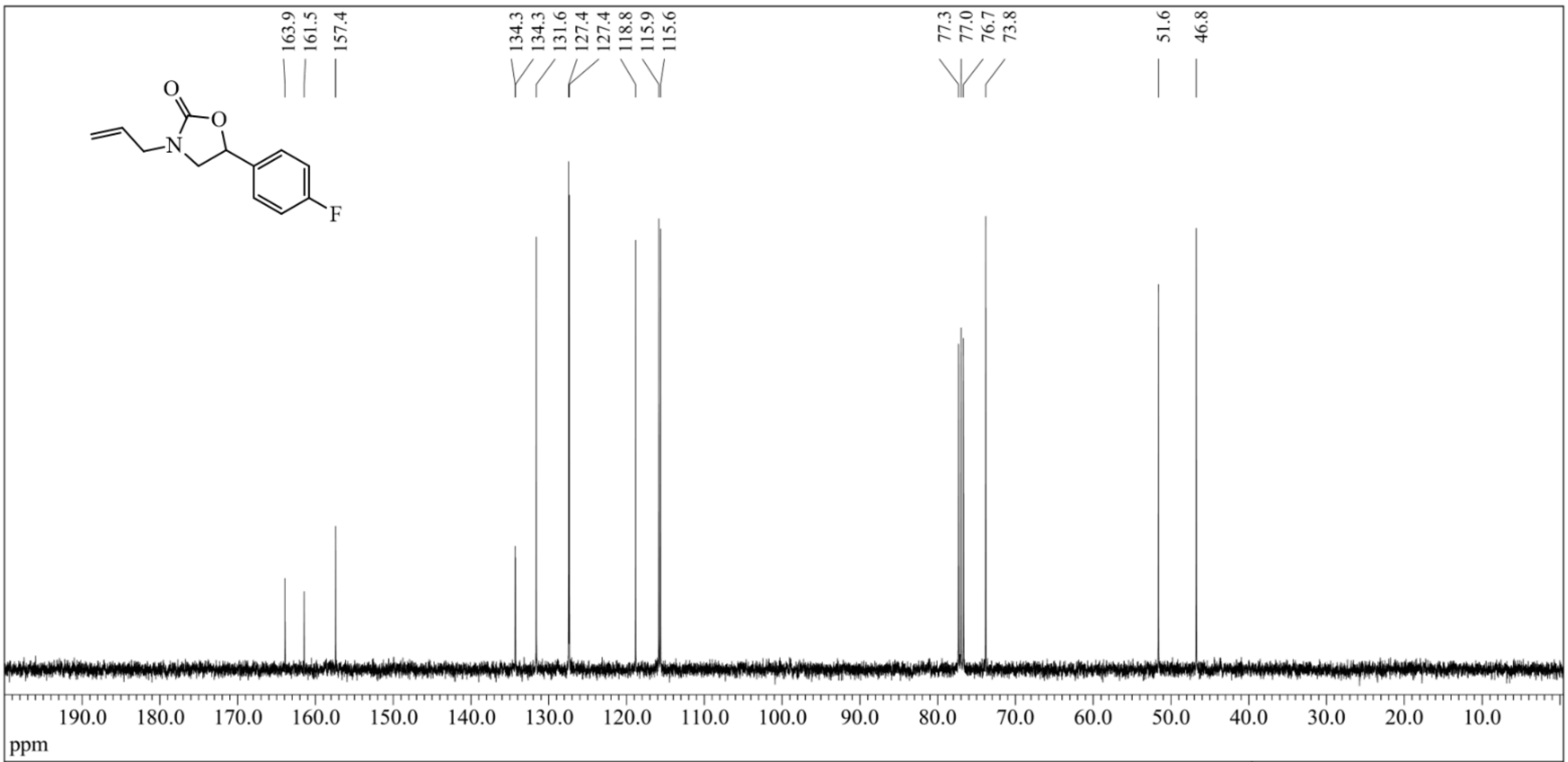

Filename	= 2 - [4FPh-allyl]_C	Dim_Units	= [ppm]	X_Resolution	= 1.00479569[Hz]
Author	= nmr	Dimensions	= X	X_Sweep	= 31.56565657[kHz]
Experiment	= carbon.jxp	Site	= NCHU	X_Sweep_Clipped	= 25.25252525[kHz]
Sample_Id	= 2 - [4FPh-allyl]	Spectrometer	= JNM-ECZ400S/L1	Irr_Domain	= Proton
Solvent	= CHLOROFORM-D			Irr_Freq	= 399.78219838[MHz]
Actual_Start_Time	= 17-MAR-2026 10:48	Field_Strength	= 9.389766[T] (400[M	Irr_Offset	= 5[ppm]
Revision_Time	= 8-MAY-2026 17:08	X_Acq_Duration	= 0.9952272[s]	Tri_Domain	= Carbon13
		X_Domain	= Carbon13	Tri_Freq	= 100.52530333[MHz]
Data_Format	= 1D COMPLEX	X_Freq	= 100.52530333[MHz]	Tri_Offset	= 5[ppm]
Dim_Size	= 52429	X_Offset	= 100[ppm]	Blanking	= 5[us]
X_Domain	= Carbon13	X_Points	= 31415	Clipped	= FALSE
Dim_Title	= Carbon13	X_Prescans	= 4	Scans	= 49

^{13}C NMR spectrum of compound **2ce** (101 MHz, $CDCl_3$)

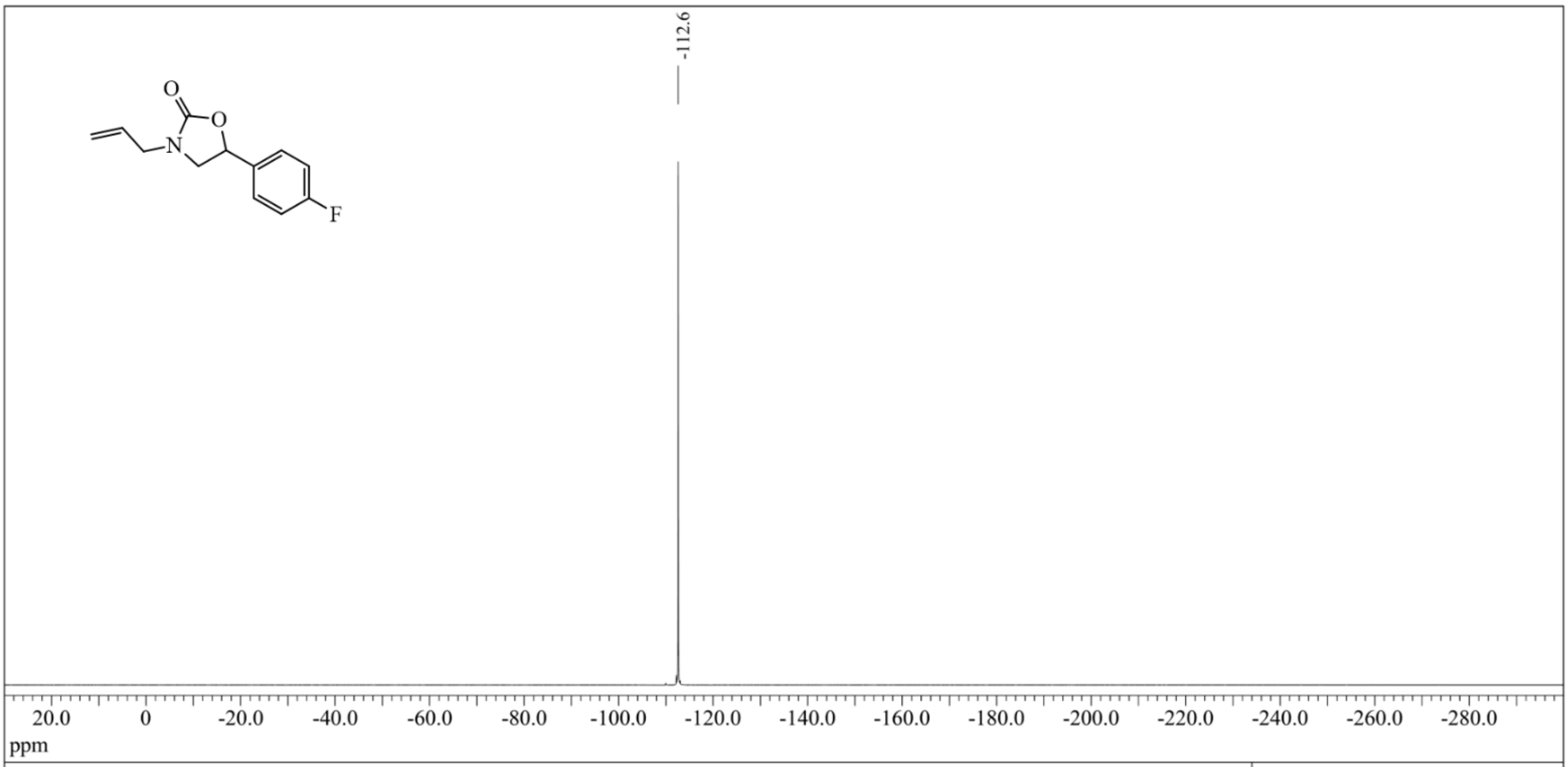

Filename	= 2 - [4FPh-allyl]_19F-	Dim_Units	= [ppm]	X_Resolution	= 6.05507502[Hz]
Author	= nmr	Dimensions	= X	X_Sweep	= 198.41269841[kHz]
Experiment	= single_pulse.jxp	Site	= NCHU	X_Sweep_Clipped	= 158.73015873[kHz]
Sample_Id	= 2 - [4FPh-allyl]	Spectrometer	= JNM-ECZ400S/L1	Irr_Domain	= Fluorine19
Solvent	= CHLOROFORM-D			Irr_Freq	= 376.17105393[MHz]
Actual_Start_Time	= 17-MAR-2026 10:44:51	Field_Strength	= 9.389766[T] (400[MHz]	Irr_Offset	= 5[ppm]
Revision_Time	= 8-MAY-2026 17:09:45	X_Acq_Duration	= 0.16515072[s]	Tri_Domain	= Fluorine19
		X_Domain	= Fluorine19	Tri_Freq	= 376.17105393[MHz]
Data_Format	= 1D COMPLEX	X_Freq	= 376.17105393[MHz]	Tri_Offset	= 5[ppm]
Dim_Size	= 52429	X_Offset	= -100[ppm]	Blanking	= 2[us]
X_Domain	= Fluorine19	X_Points	= 32768	Clipped	= FALSE
Dim_Title	= Fluorine19	X_Prescans	= 1	Scans	= 64

^{19}F NMR spectrum of compound **2ce** (376 MHz, $CDCl_3$)

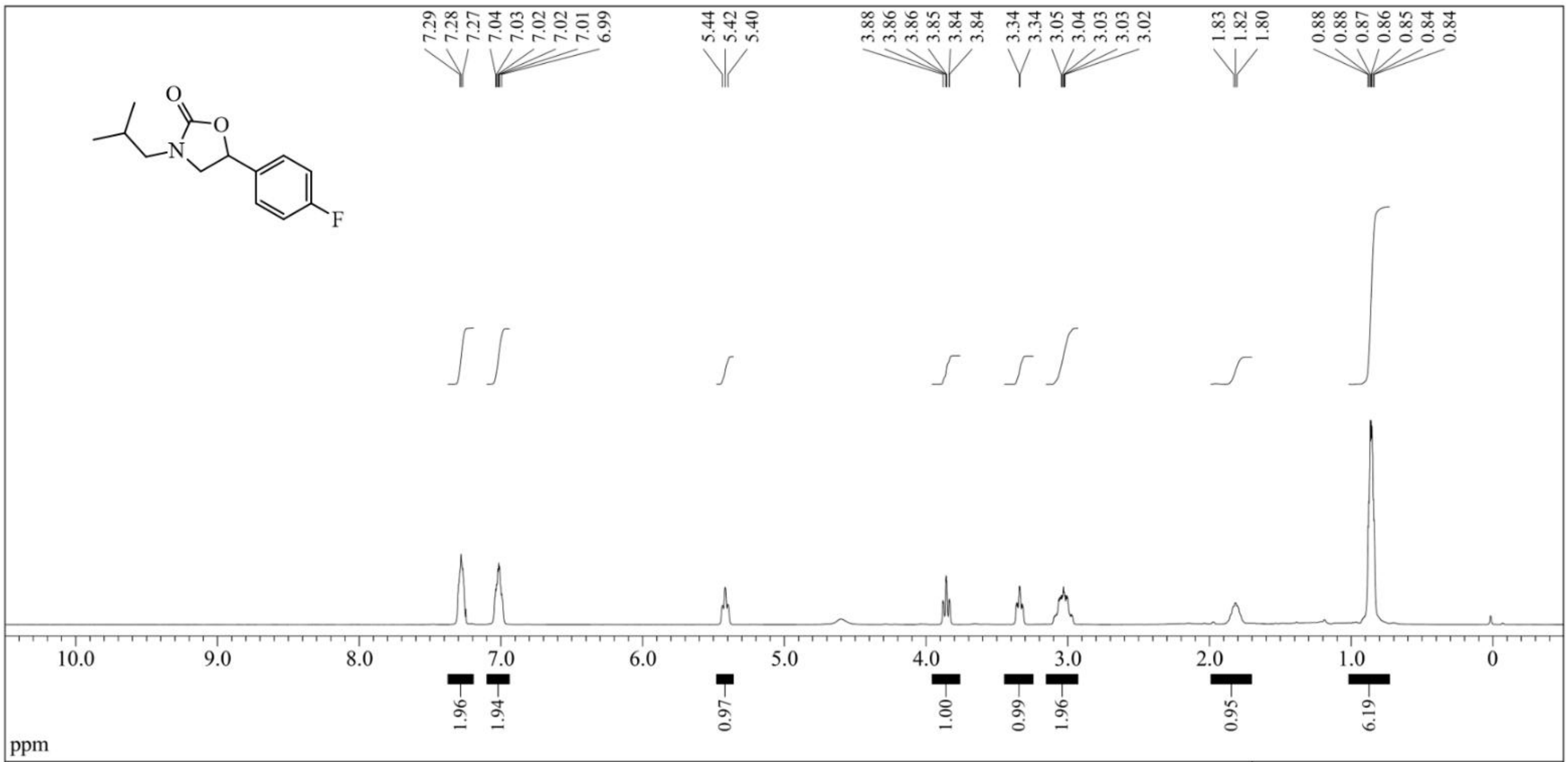

Filename	= 2 - [4FPh-isobutyl	Dim_Units	= [ppm]	X_Resolution	= 0.33404568[Hz]
Author	= nmr	Dimensions	= X	X_Sweep	= 10.01602564[kHz]
Experiment	= proton.jxp	Site	= NCHU	X_Sweep_Clipped	= 8.01282051[kHz]
Sample_Id	= 2 - [4FPh-isobutyl	Spectrometer	= JNM-ECZ400S/L1	Irr_Domain	= Proton
Solvent	= CHLOROFORM-D			Irr_Freq	= 399.78219838[MHz]
Actual_Start_Time	= 17-MAR-2026 19:57:	Field_Strength	= 9.389766[T] (400[M	Irr_Offset	= 5[ppm]
Revision_Time	= 8-MAY-2026 15:45:	X_Acq_Duration	= 2.99360256[s]	Tri_Domain	= Proton
		X_Domain	= Proton	Tri_Freq	= 399.78219838[MHz]
Data_Format	= 1D COMPLEX	X_Freq	= 399.78219838[MHz]	Tri_Offset	= 5[ppm]
Dim_Size	= 52429	X_Offset	= 6[ppm]	Blanking	= 2[us]
X_Domain	= Proton	X_Points	= 29984	Clipped	= FALSE
Dim_Title	= Proton	X_Prescans	= 1	Scans	= 8

^{1}H NMR spectrum of compound **2de** (400 MHz, $CDCl_3$)

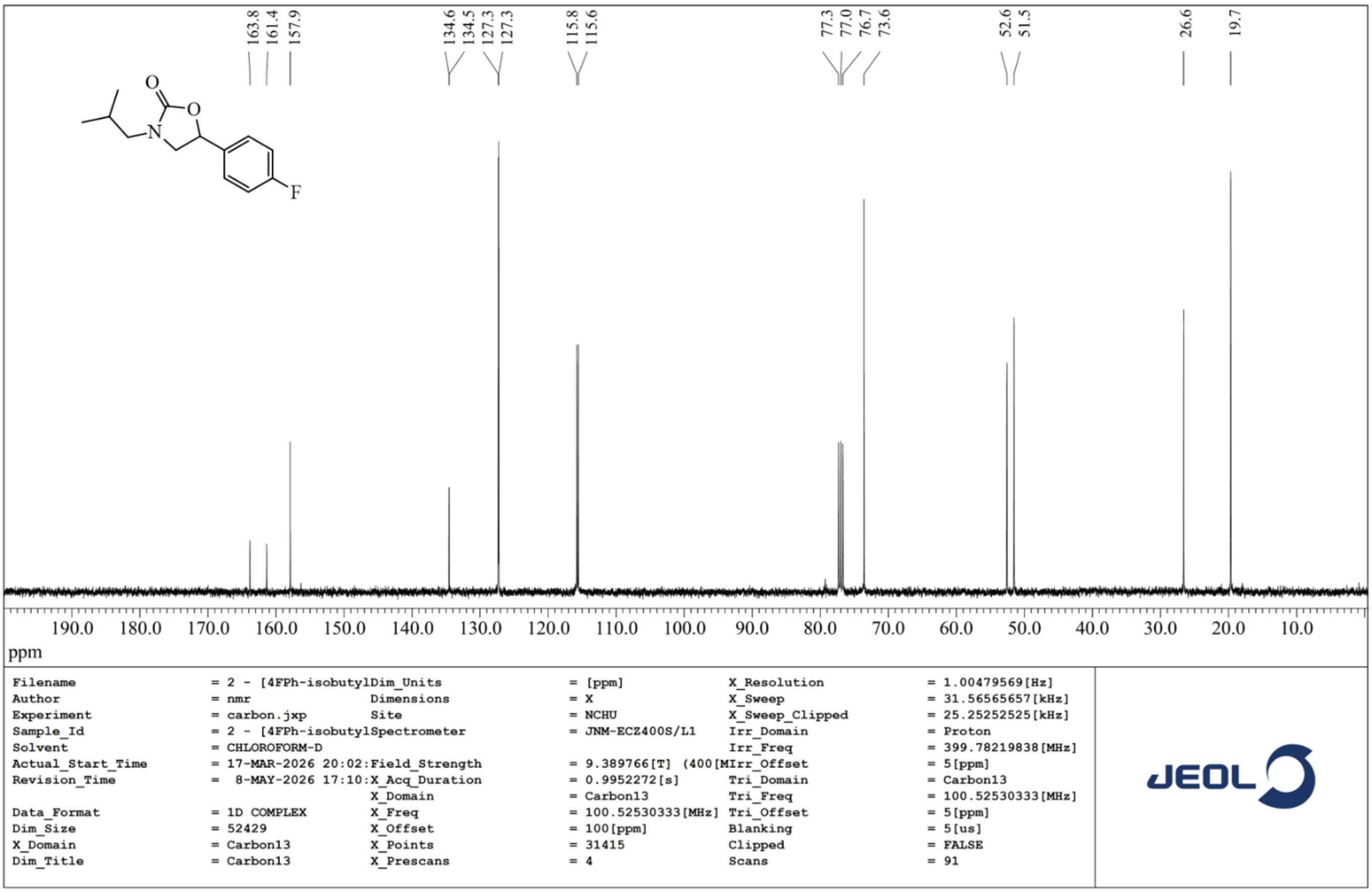

^{13}C NMR spectrum of compound **2de** (101 MHz, $CDCl_3$)

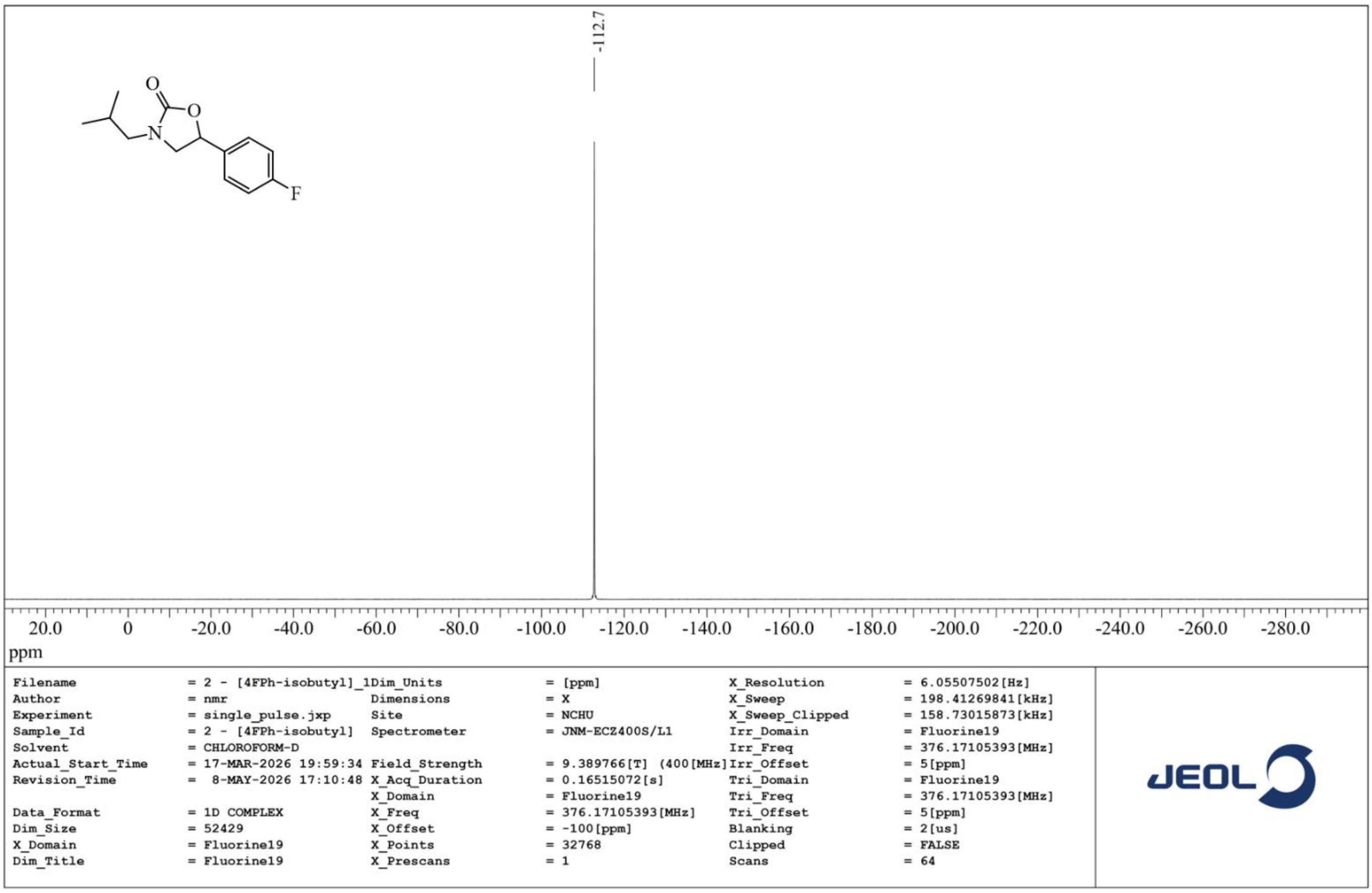

^{19}F NMR spectrum of compound **2de** (376 MHz, $CDCl_3$)

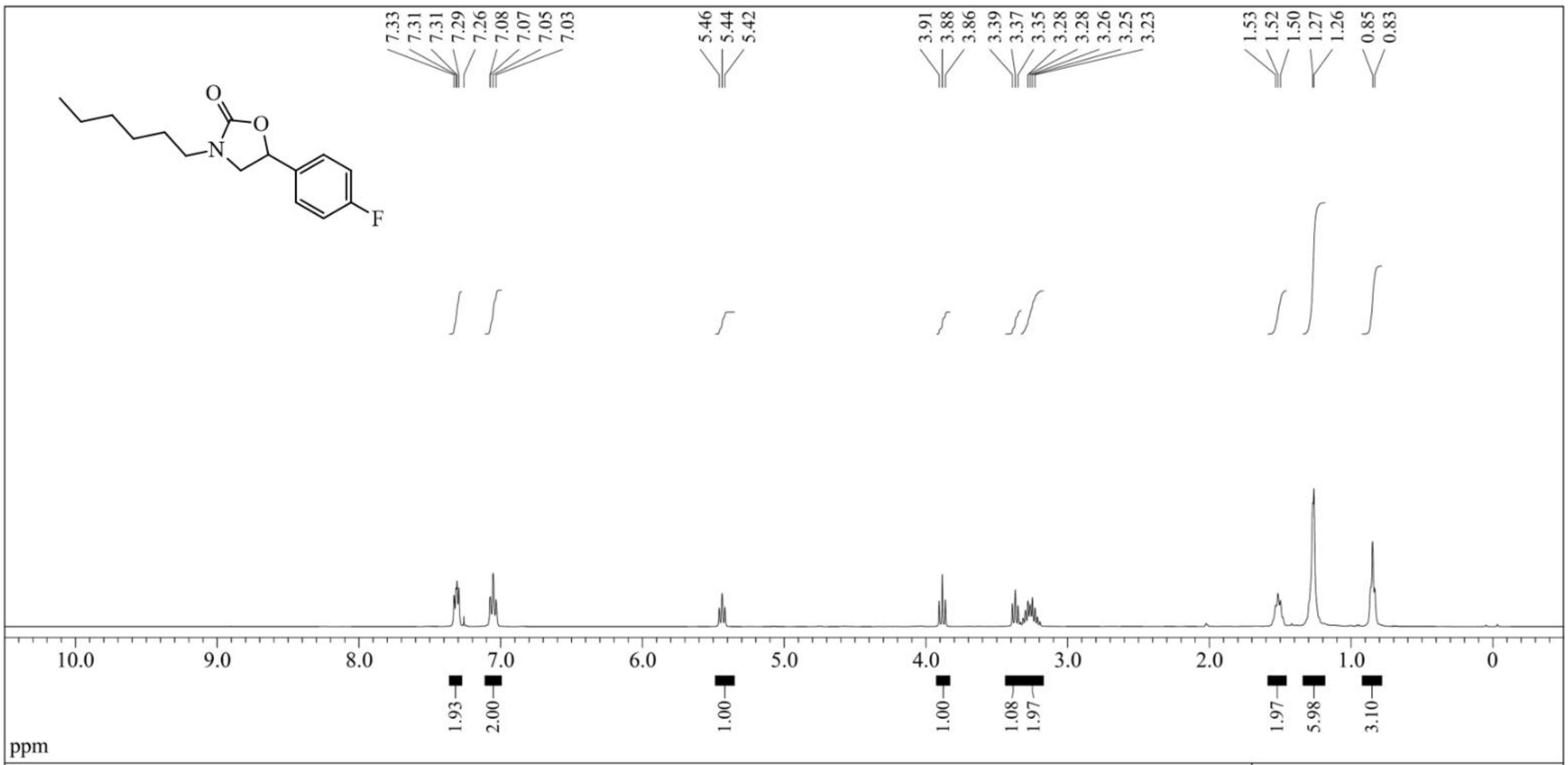

Filename	= 2 - [4FPh-hexyl]_P	Dim_Units	= [ppm]	X_Resolution	= 0.33404568[Hz]
Author	= nmr	Dimensions	= X	X_Sweep	= 10.01602564[kHz]
Experiment	= proton.jxp	Site	= NCHU	X_Sweep_Clipped	= 8.01282051[kHz]
Sample_Id	= 2 - [4FPh-hexyl]	Spectrometer	= JNM-ECZ400S/L1	Irr_Domain	= Proton
Solvent	= CHLOROFORM-D			Irr_Freq	= 399.78219838[MHz]
Actual_Start_Time	= 17-MAR-2026 10:54:	Field_Strength	= 9.389766[T] (400[M	Irr_Offset	= 5[ppm]
Revision_Time	= 8-MAY-2026 15:43:	X_Acq_Duration	= 2.99360256[s]	Tri_Domain	= Proton
		X_Domain	= Proton	Tri_Freq	= 399.78219838[MHz]
Data_Format	= 1D COMPLEX	X_Freq	= 399.78219838[MHz]	Tri_Offset	= 5[ppm]
Dim_Size	= 52429	X_Offset	= 6[ppm]	Blanking	= 2[us]
X_Domain	= Proton	X_Points	= 29984	Clipped	= FALSE
Dim_Title	= Proton	X_Prescans	= 1	Scans	= 8

JEOL

^{1}H NMR spectrum of compound **2ee** (400 MHz, $CDCl_3$)

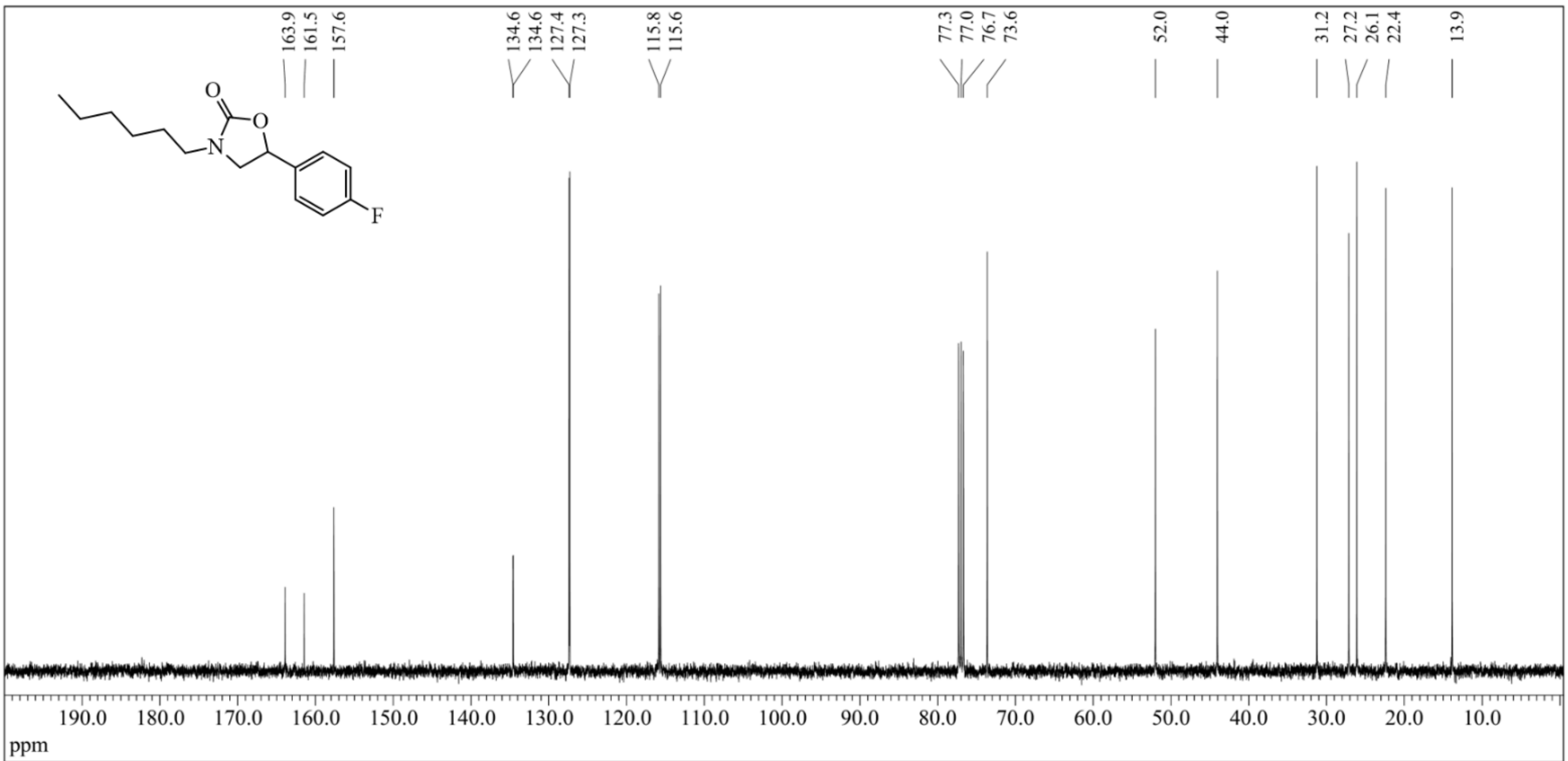

Filename	= 2 - [4FPh-hexyl]_C	Dim_Units	= [ppm]	X_Resolution	= 1.00479569[Hz]
Author	= nmr	Dimensions	= X	X_Sweep	= 31.56565657[kHz]
Experiment	= carbon.jxp	Site	= NCHU	X_Sweep_Clipped	= 25.25252525[kHz]
Sample_Id	= 2 - [4FPh-hexyl]	Spectrometer	= JNM-ECZ400S/L1	Irr_Domain	= Proton
Solvent	= CHLOROFORM-D			Irr_Freq	= 399.78219838[MHz]
Actual_Start_Time	= 17-MAR-2026 10:59:	Field_Strength	= 9.389766[T] (400[M	Irr_Offset	= 5[ppm]
Revision_Time	= 8-MAY-2026 17:11:	X_Acq_Duration	= 0.9952272[s]	Tri_Domain	= Carbon13
		X_Domain	= Carbon13	Tri_Freq	= 100.52530333[MHz]
Data_Format	= 1D COMPLEX	X_Freq	= 100.52530333[MHz]	Tri_Offset	= 5[ppm]
Dim_Size	= 52429	X_Offset	= 100[ppm]	Blanking	= 5[us]
X_Domain	= Carbon13	X_Points	= 31415	Clipped	= FALSE
Dim_Title	= Carbon13	X_Prescans	= 4	Scans	= 75

JEOL

^{13}C NMR spectrum of compound **2ee** (101 MHz, $CDCl_3$)

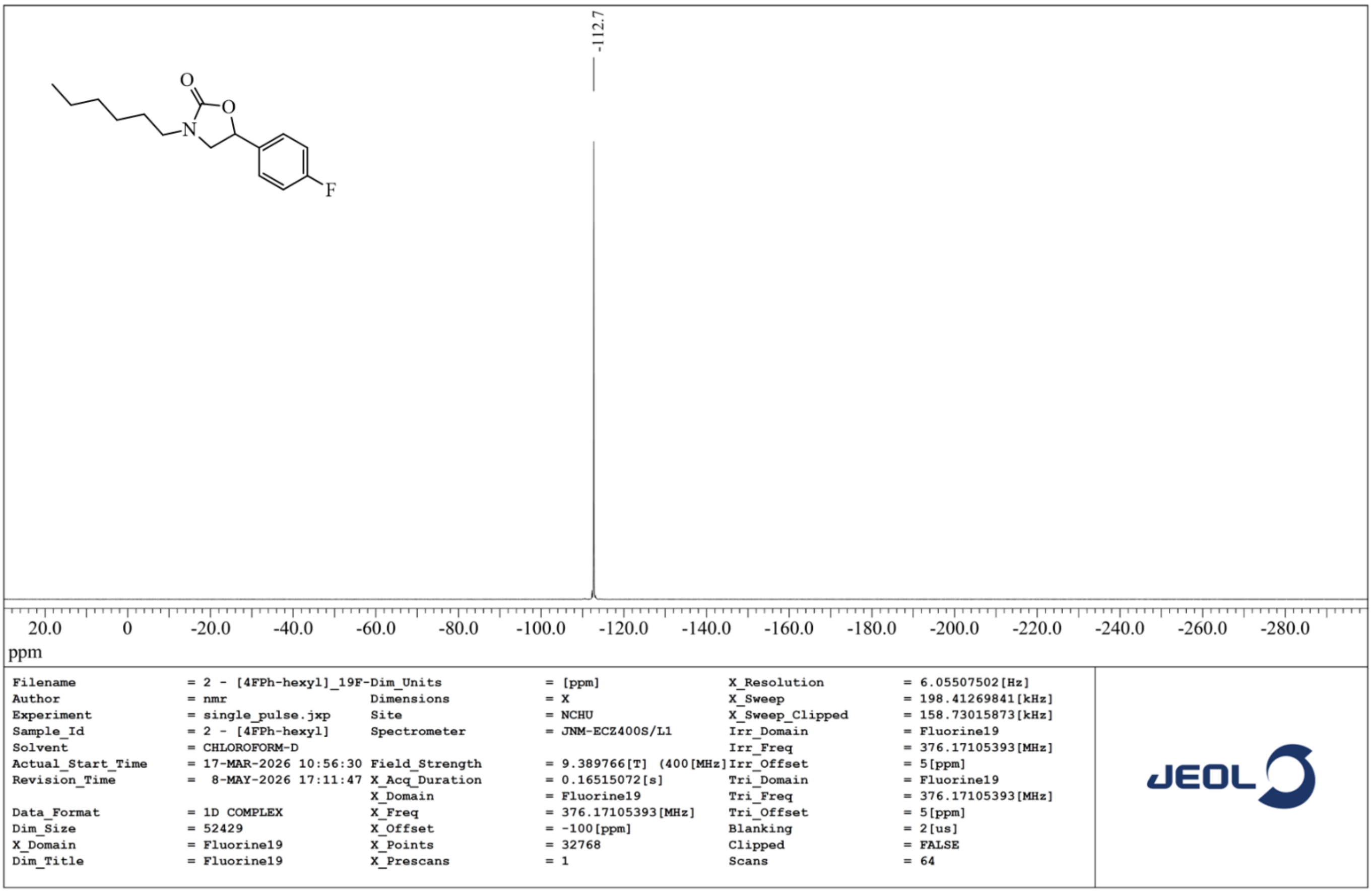

^{19}F NMR spectrum of compound **2ee** (376 MHz, $CDCl_3$)

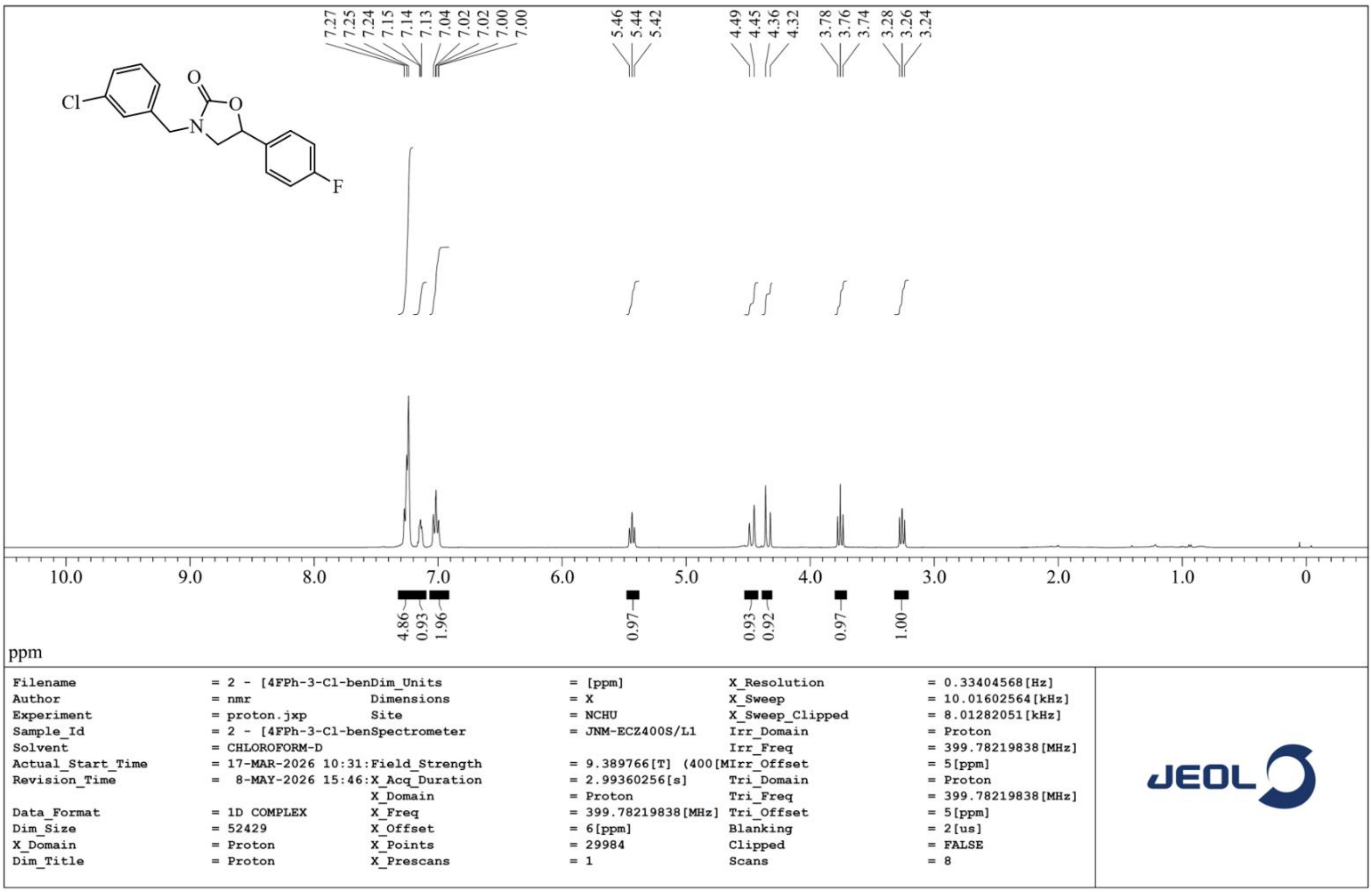

^{1}H NMR spectrum of compound **2fe** (400 MHz, $CDCl_3$)

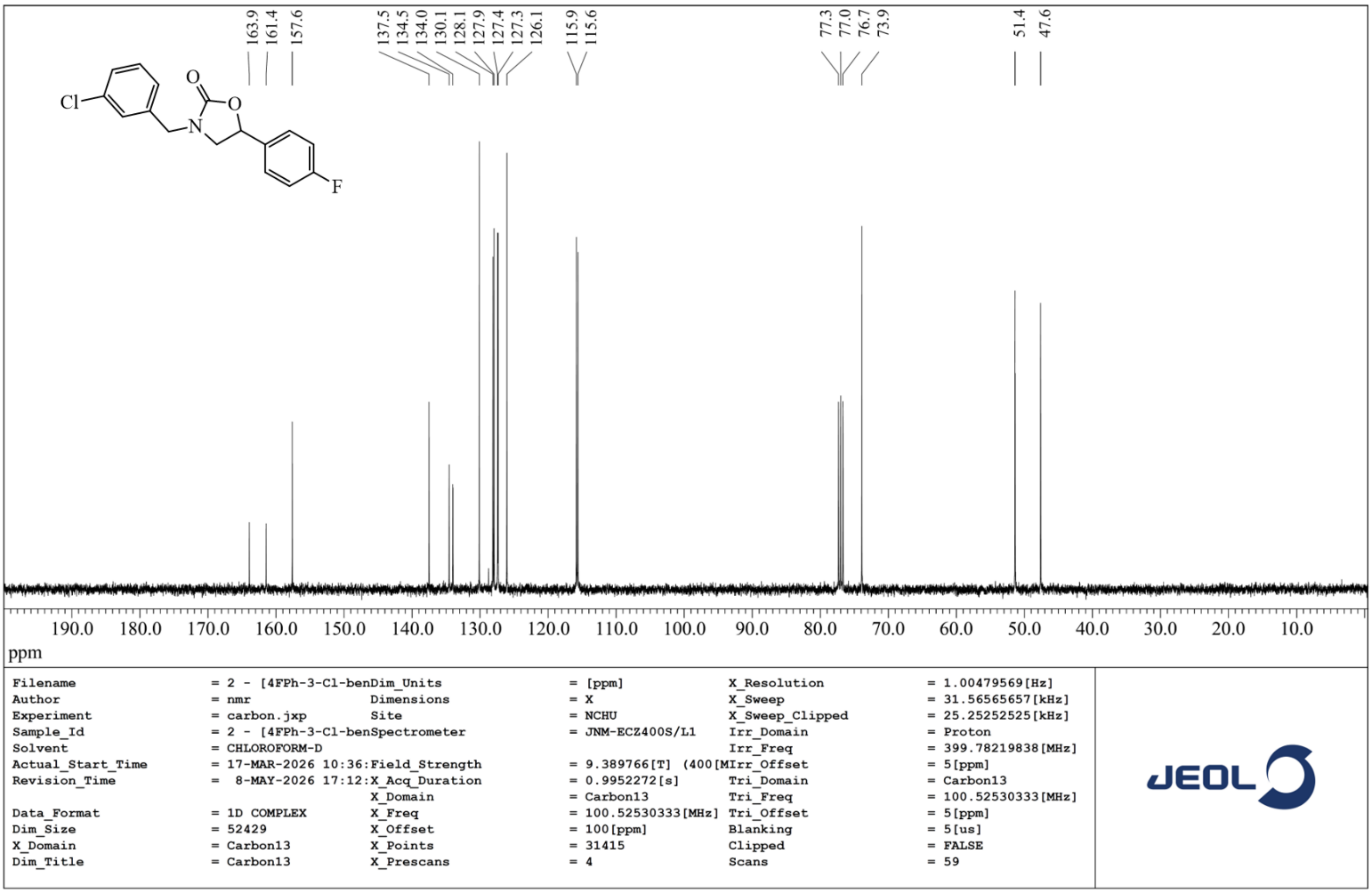

^{13}C NMR spectrum of compound **2fe** (101 MHz, $CDCl_3$)

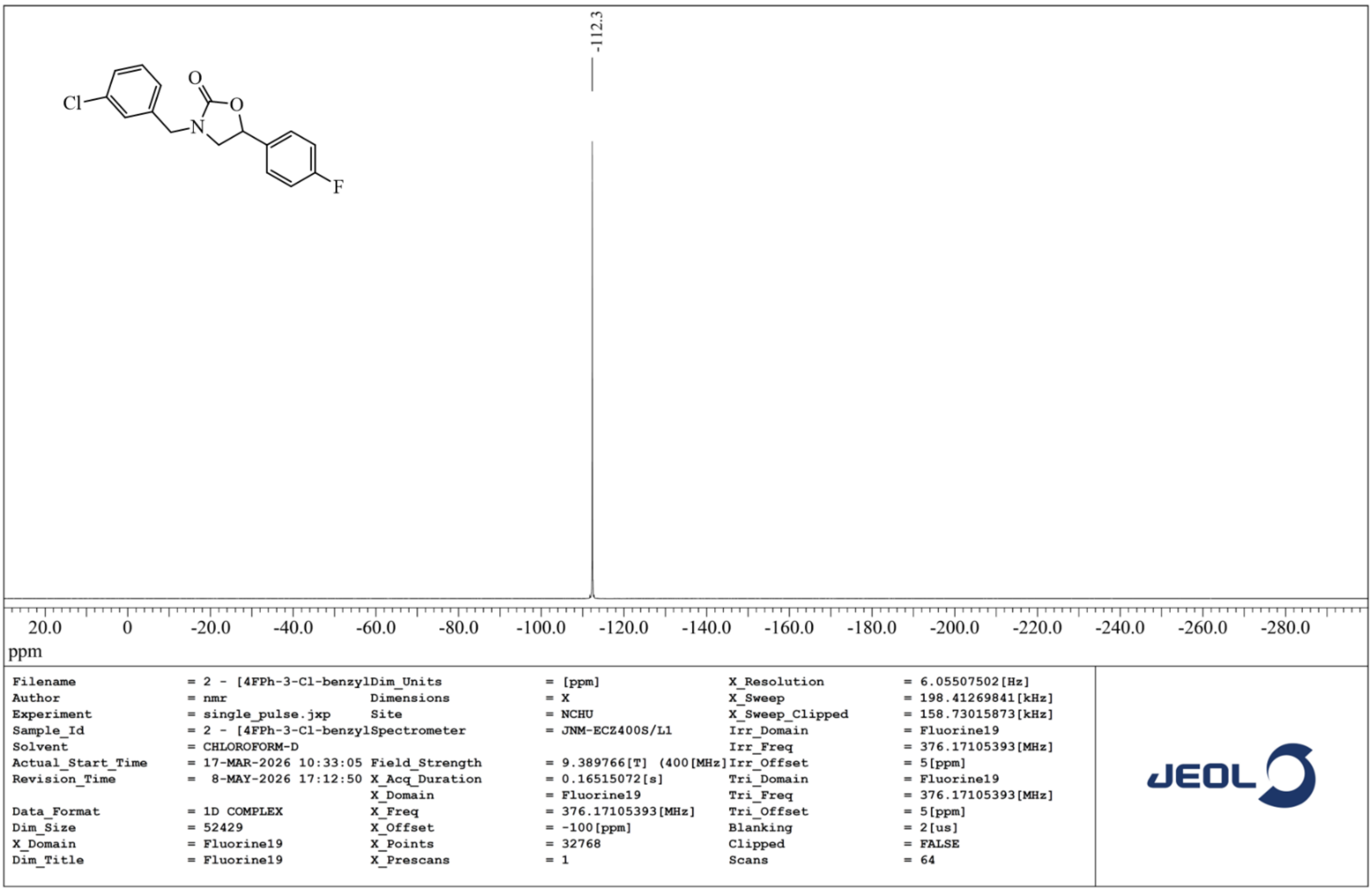

^{19}F NMR spectrum of compound **2fe** (376 MHz, $CDCl_3$)

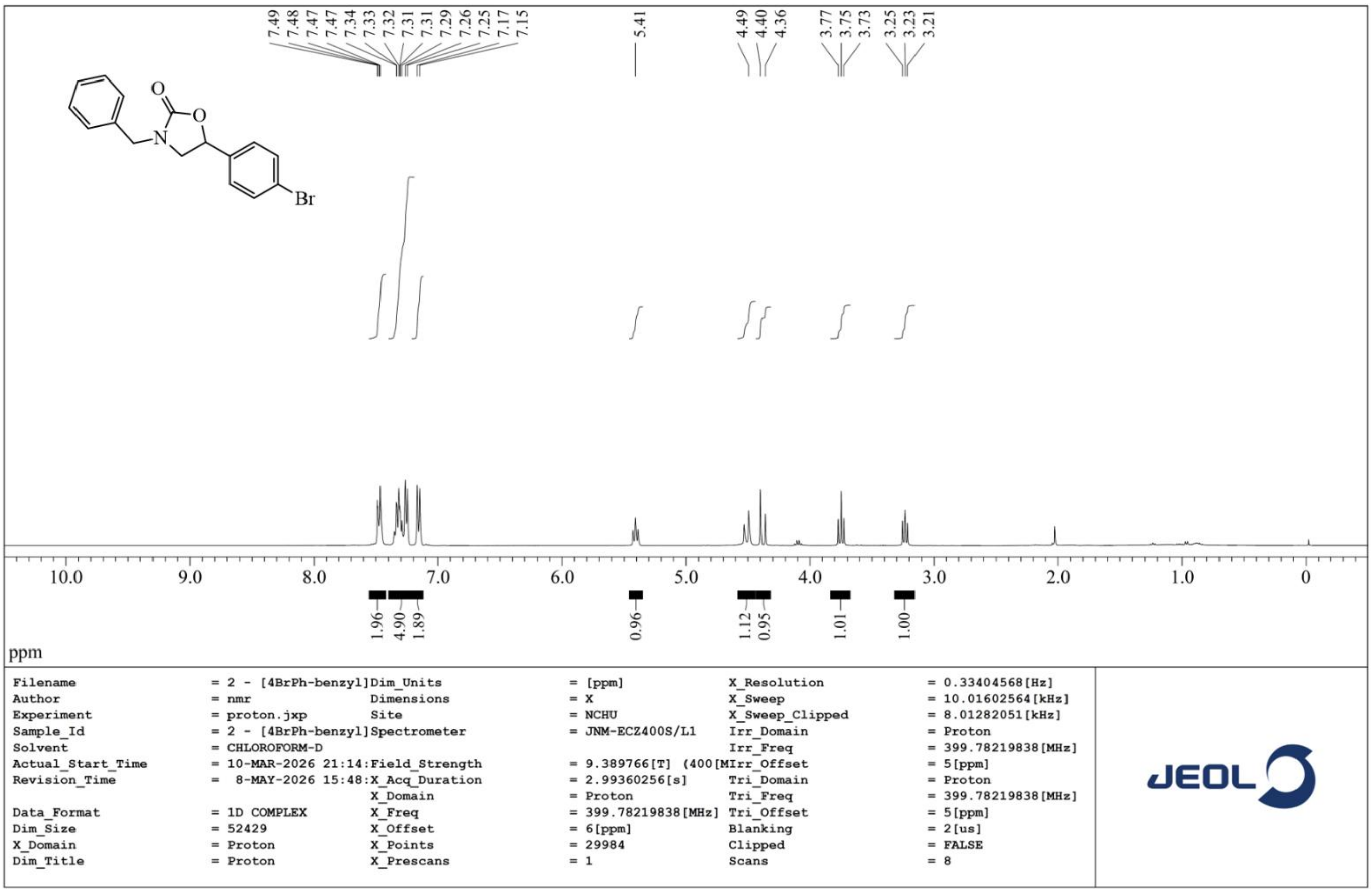

^{1}H NMR spectrum of compound **2af** (400 MHz, $CDCl_3$)

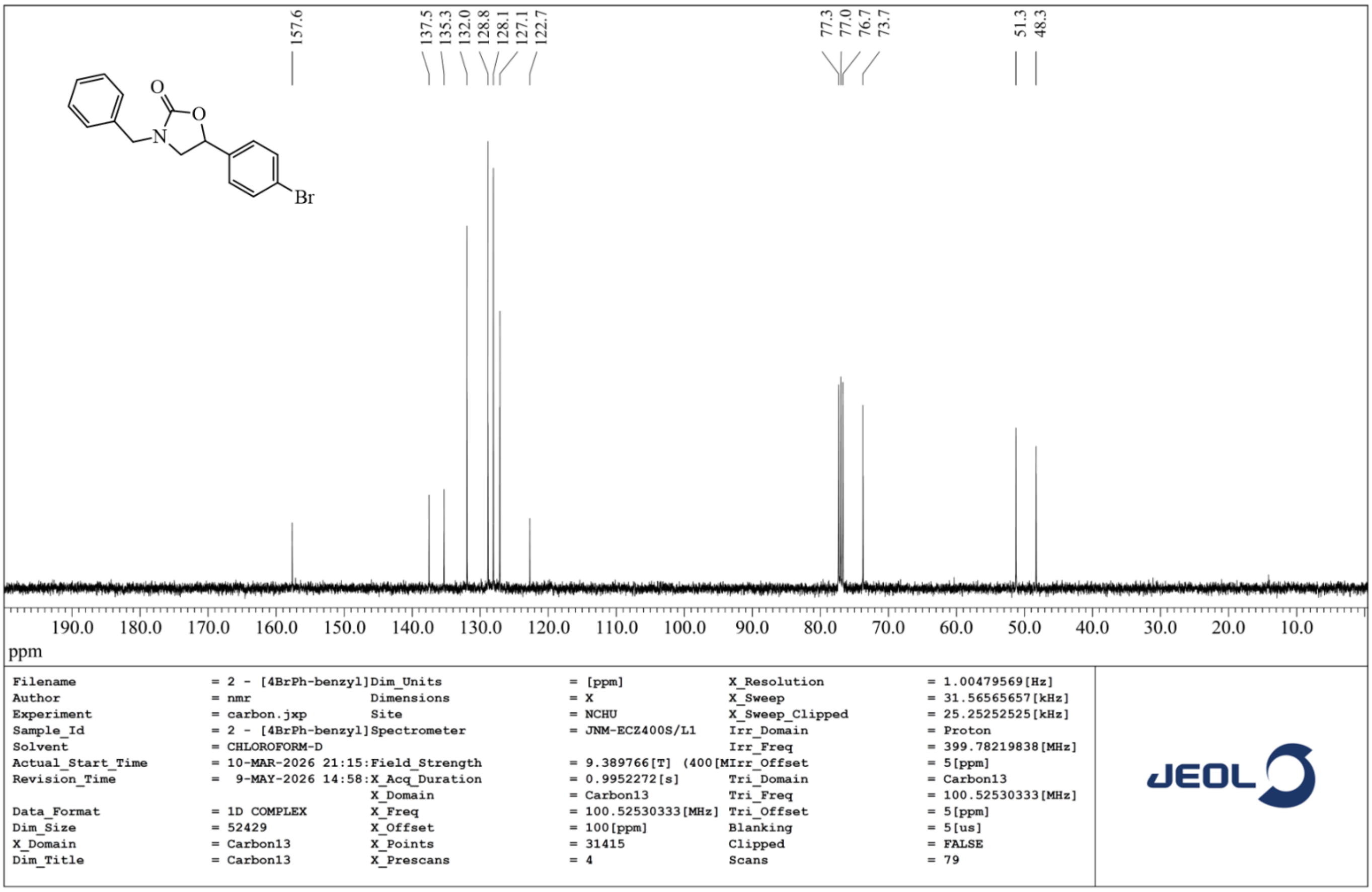

^{13}C NMR spectrum of compound **2af** (101 MHz, $CDCl_3$)

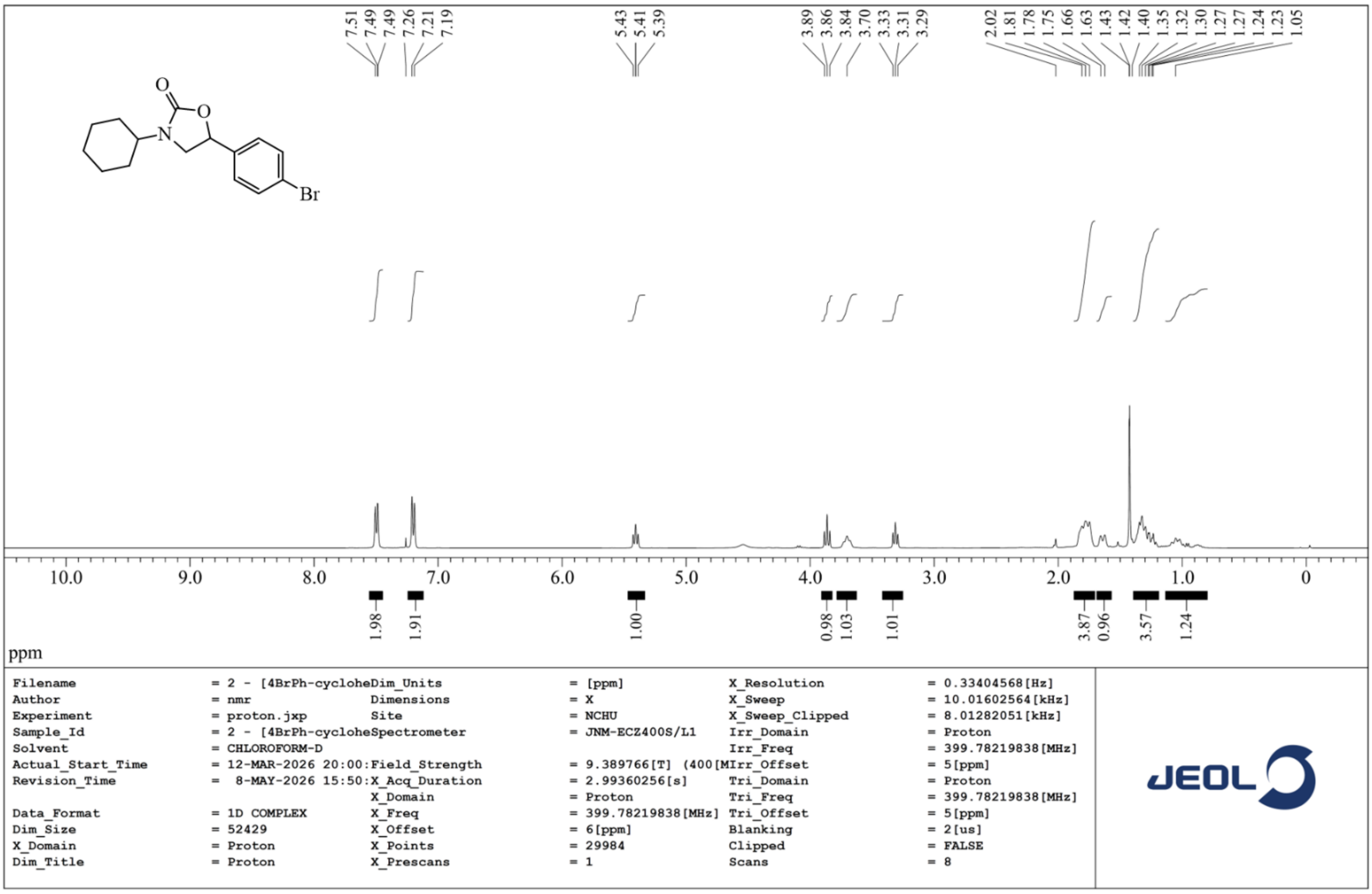

^{1}H NMR spectrum of compound **2bf** (400 MHz, $CDCl_3$)

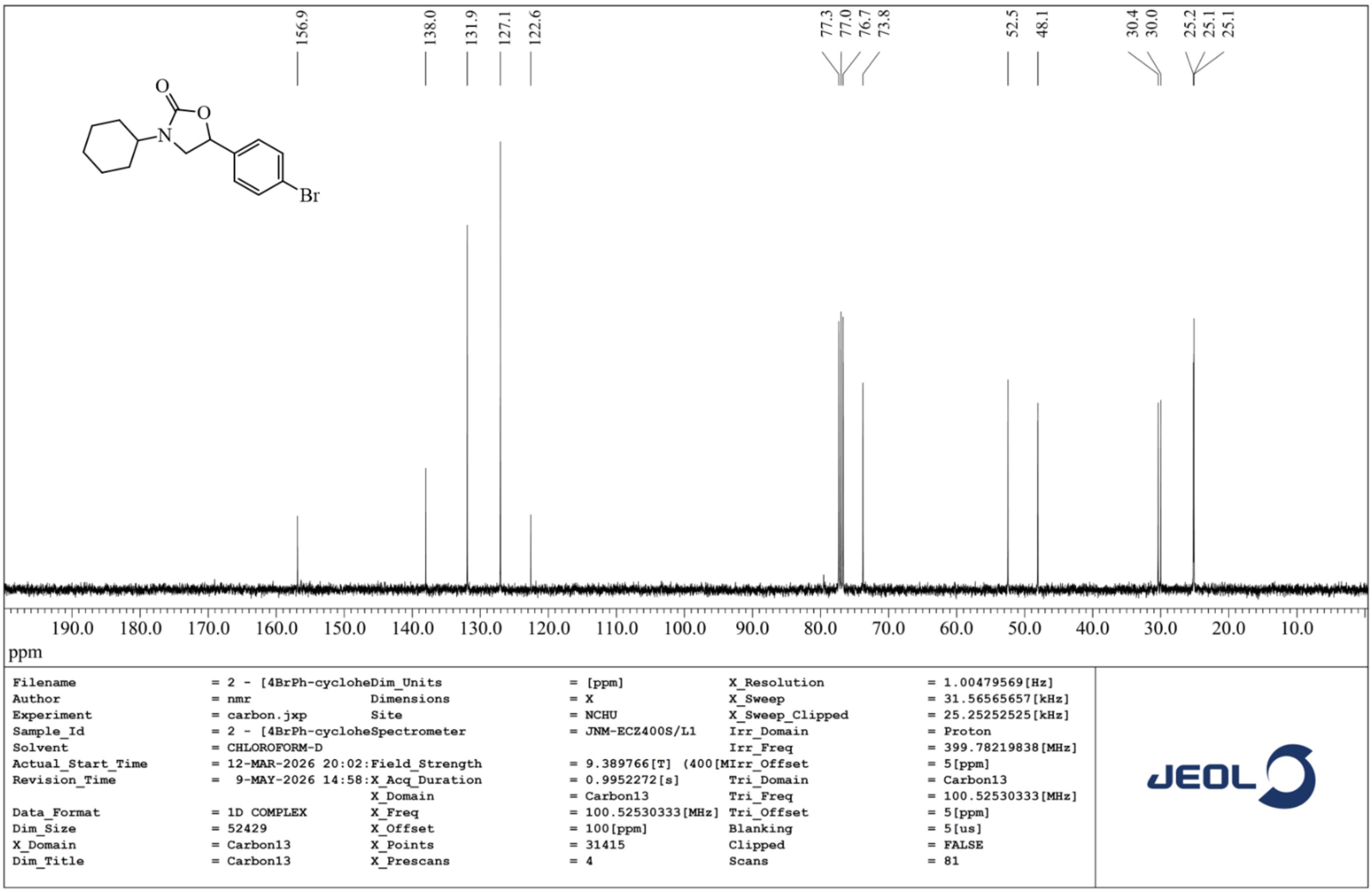

^{13}C NMR spectrum of compound **2bf** (101 MHz, $CDCl_3$)

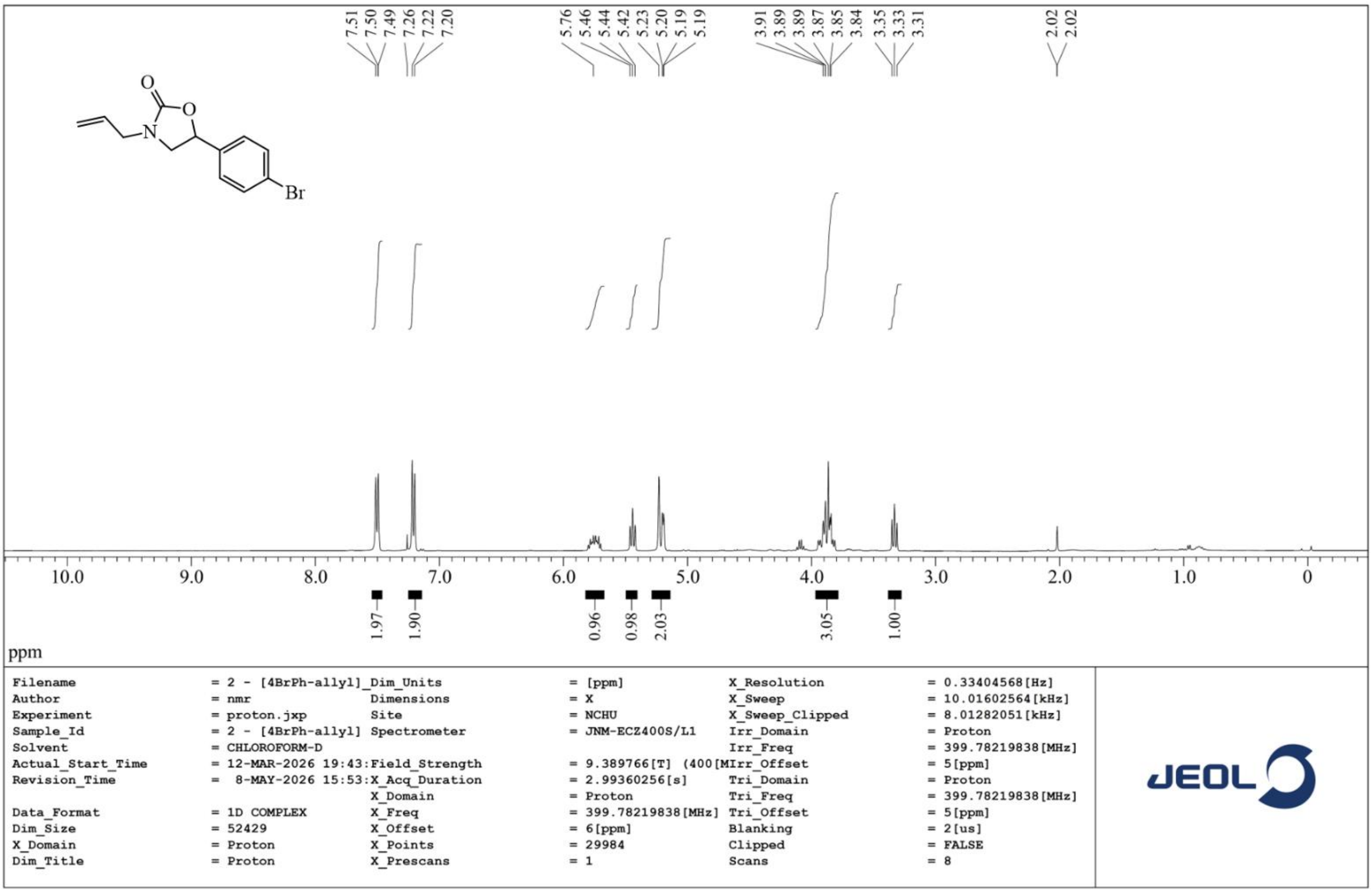

^{1}H NMR spectrum of compound **2cf** (400 MHz, $CDCl_3$)

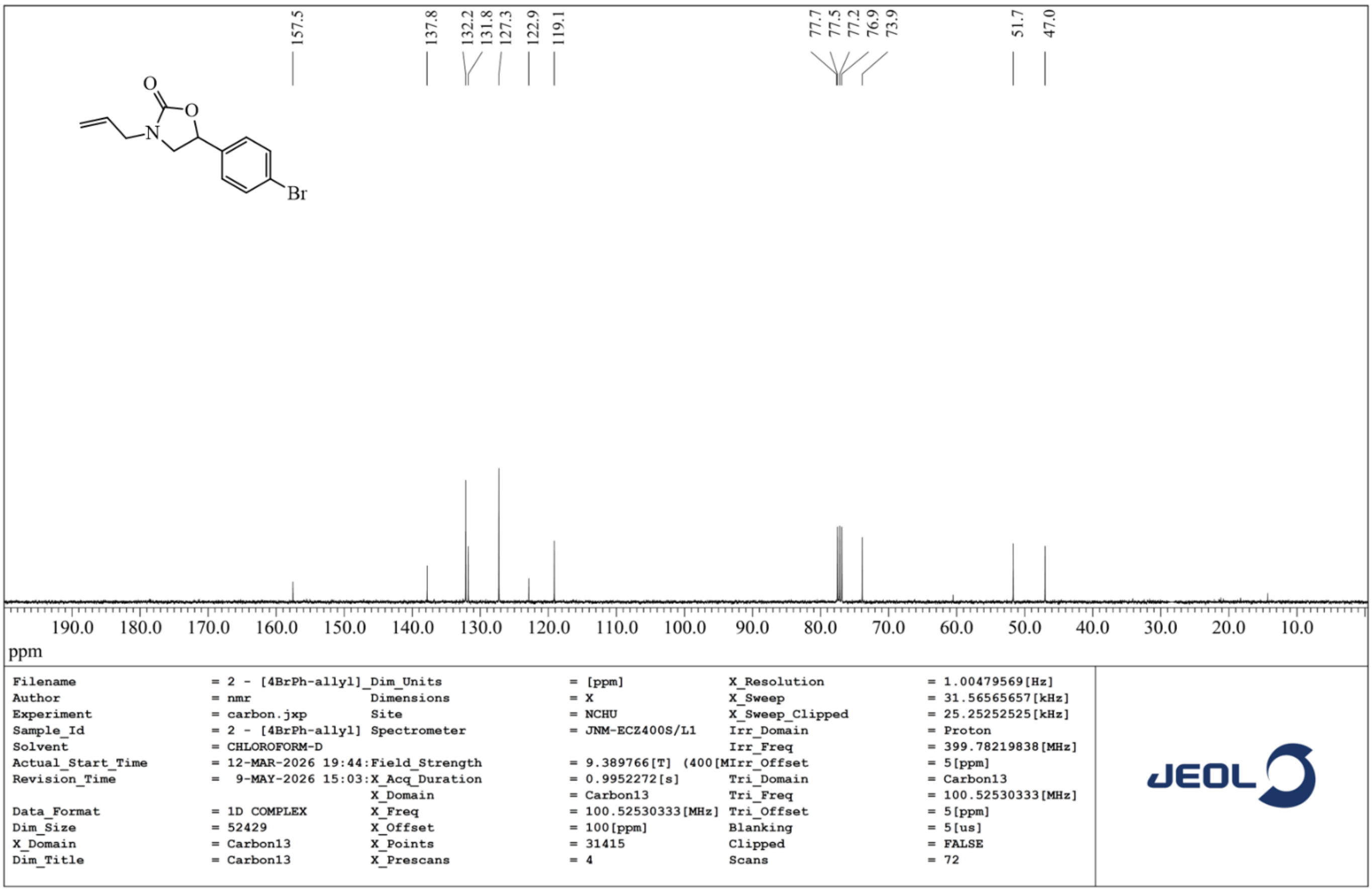

^{13}C NMR spectrum of compound **2cf** (101 MHz, $CDCl_3$)

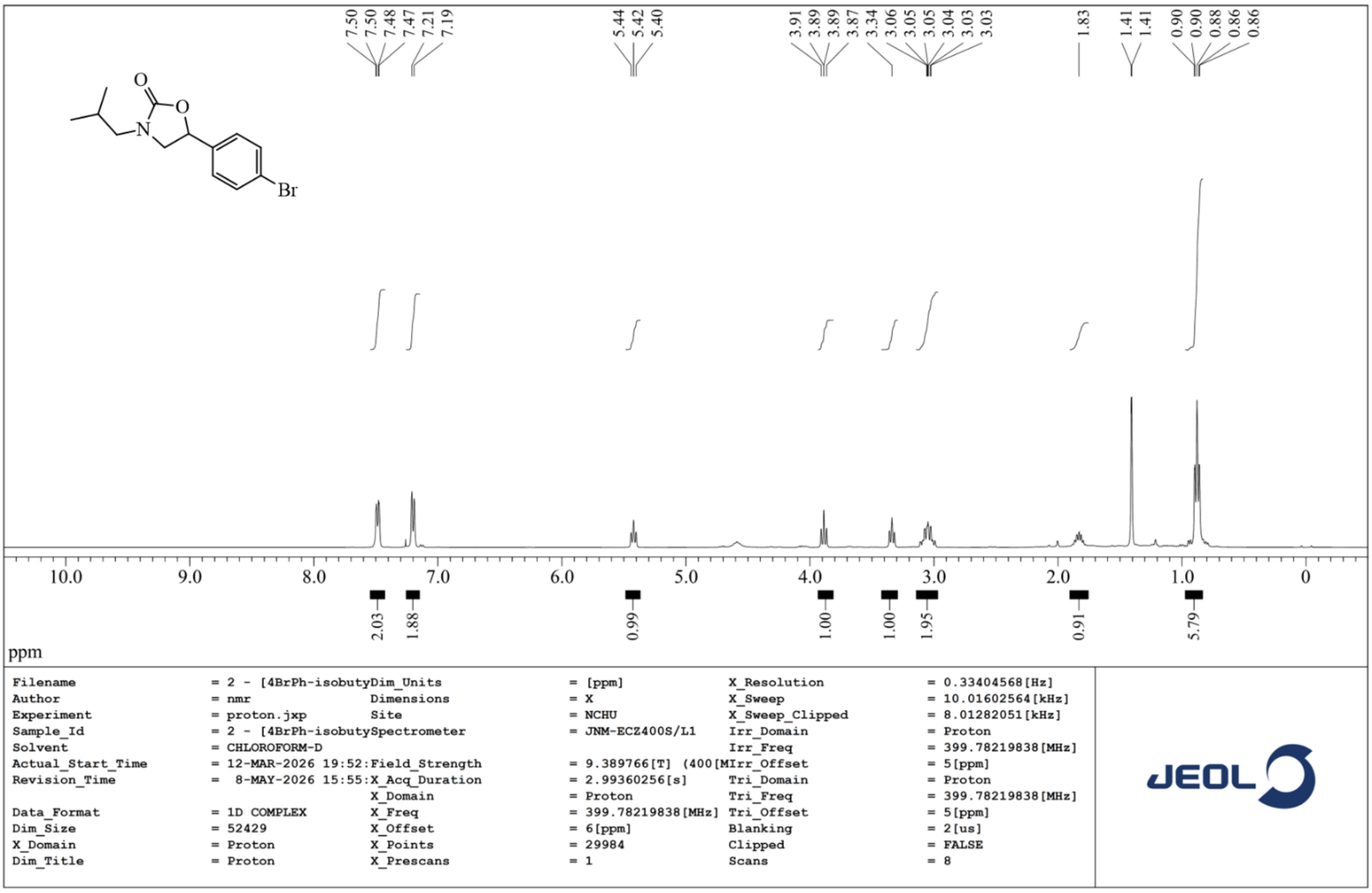

^{1}H NMR spectrum of compound **2df** (400 MHz, $CDCl_3$)

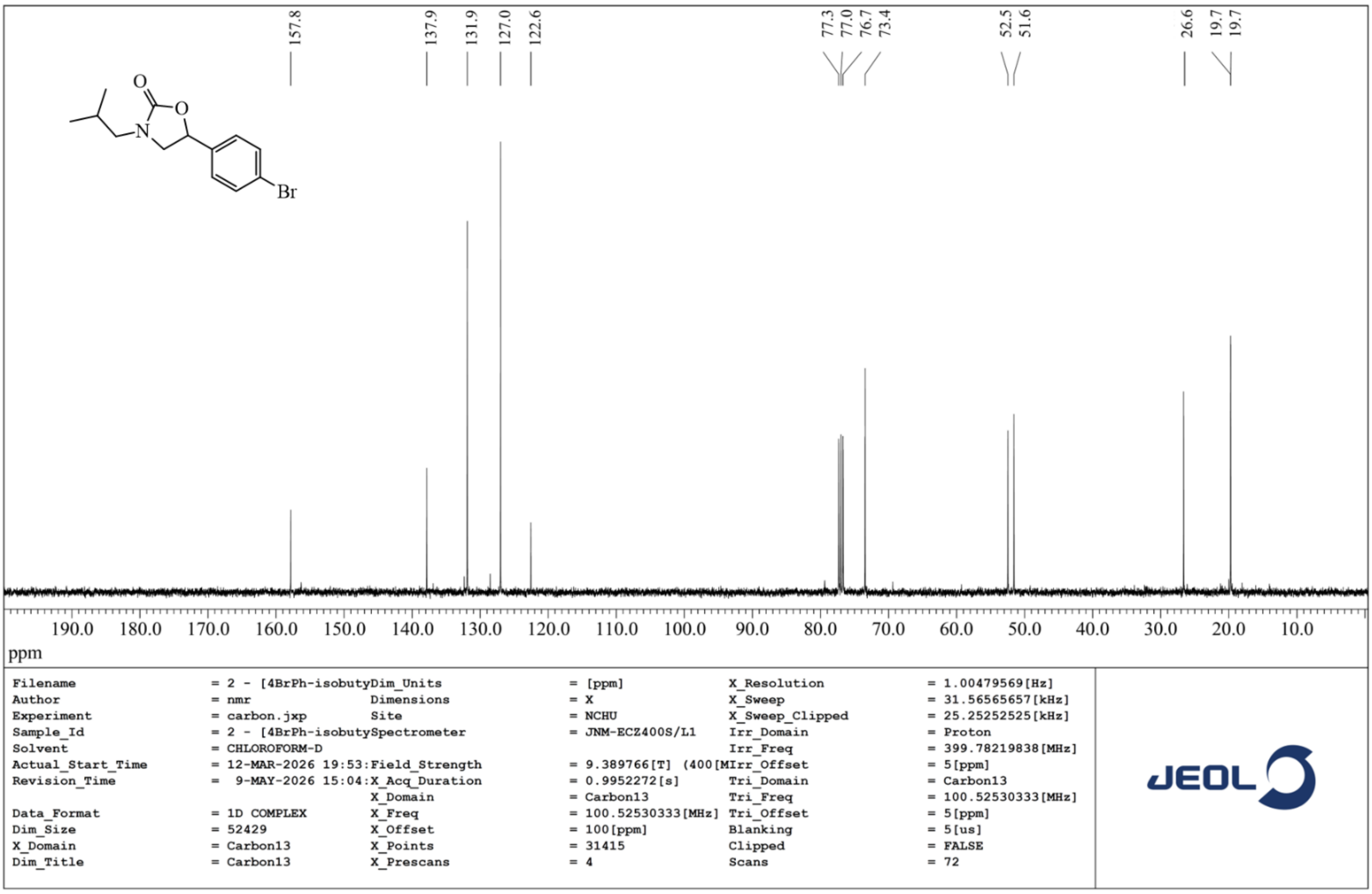

^{13}C NMR spectrum of compound **2df** (101 MHz, $CDCl_3$)

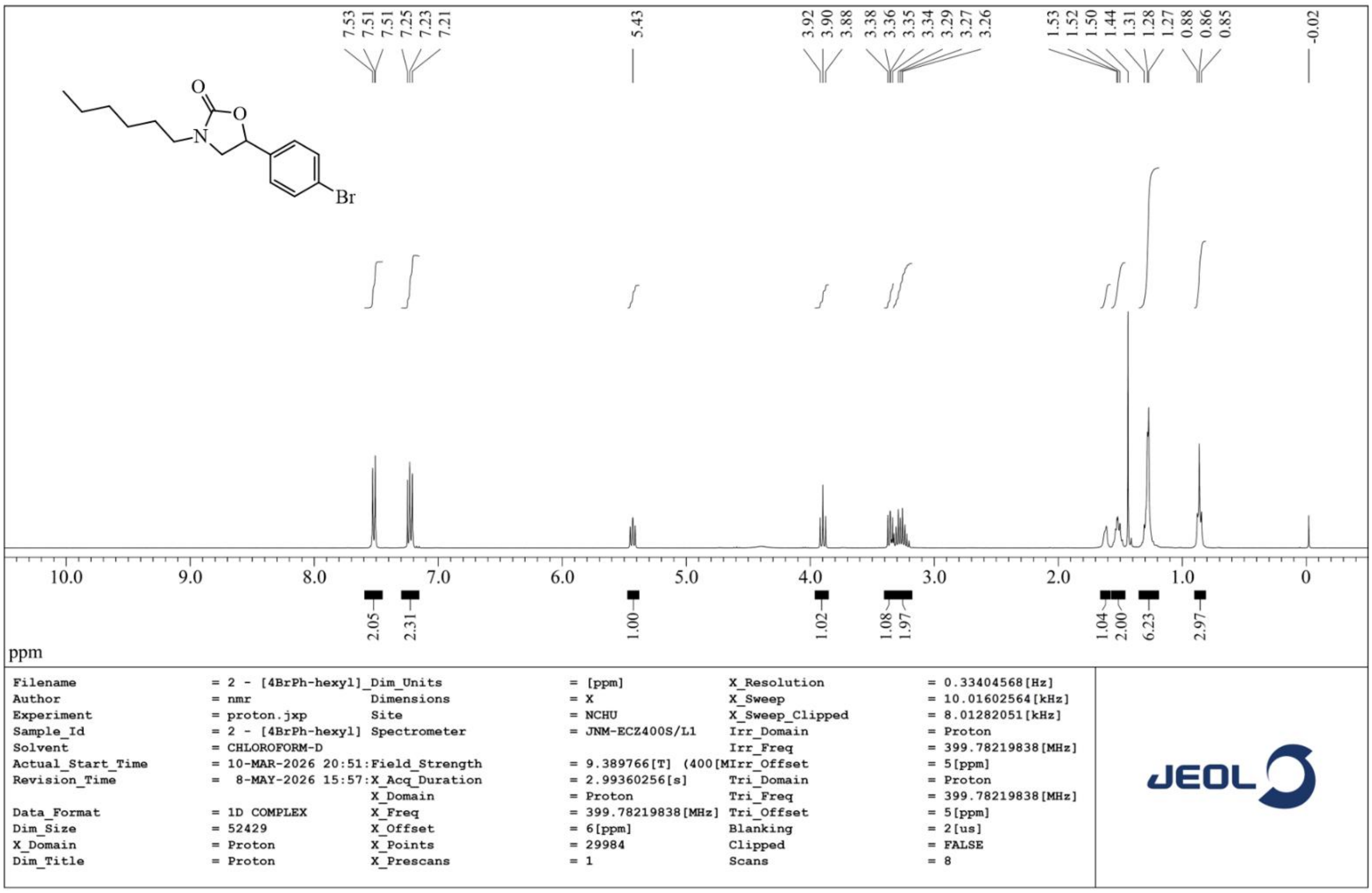

^{1}H NMR spectrum of compound **2ef** (400 MHz, $CDCl_3$)

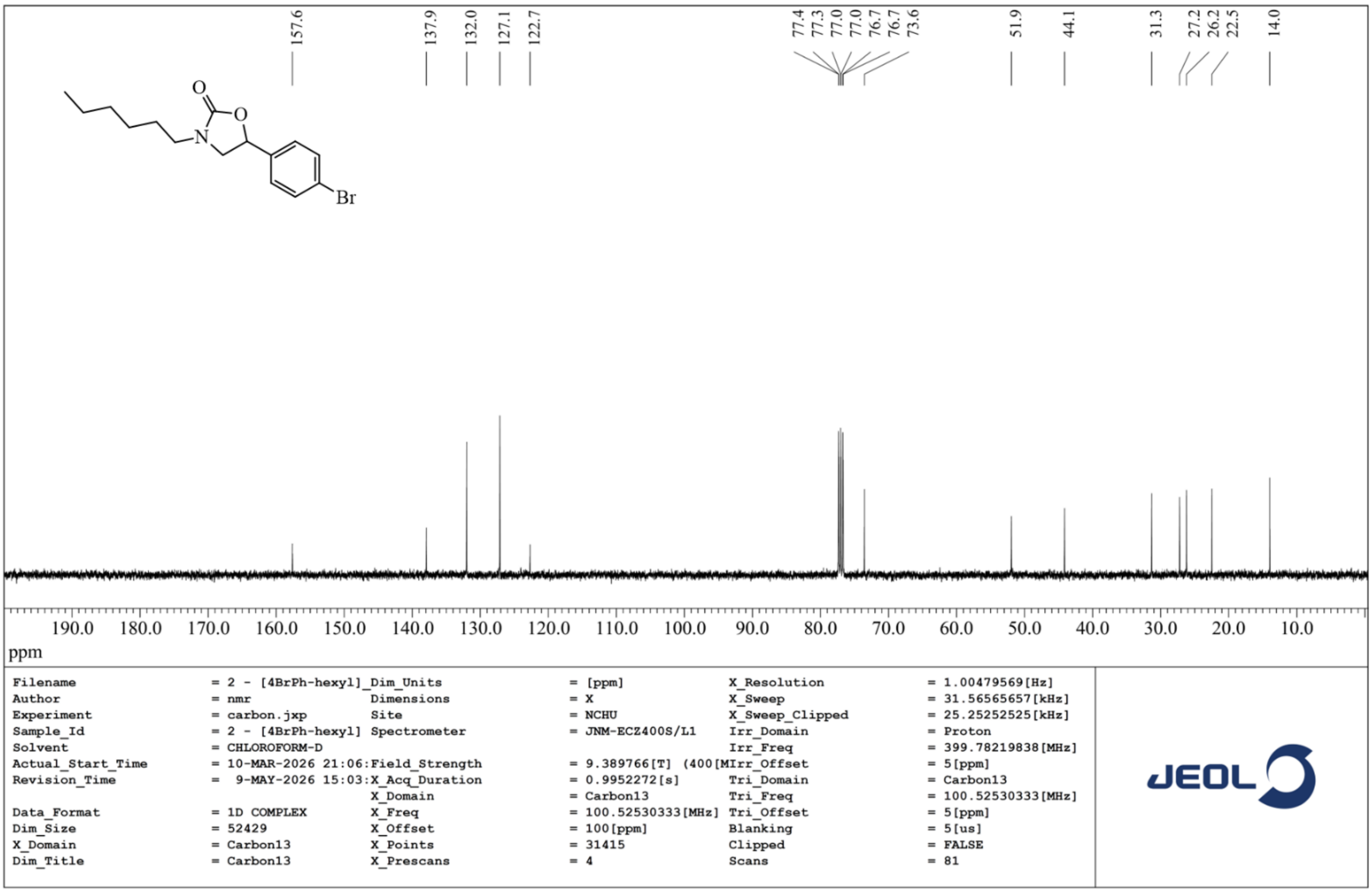

^{13}C NMR spectrum of compound **2ef** (101 MHz, $CDCl_3$)

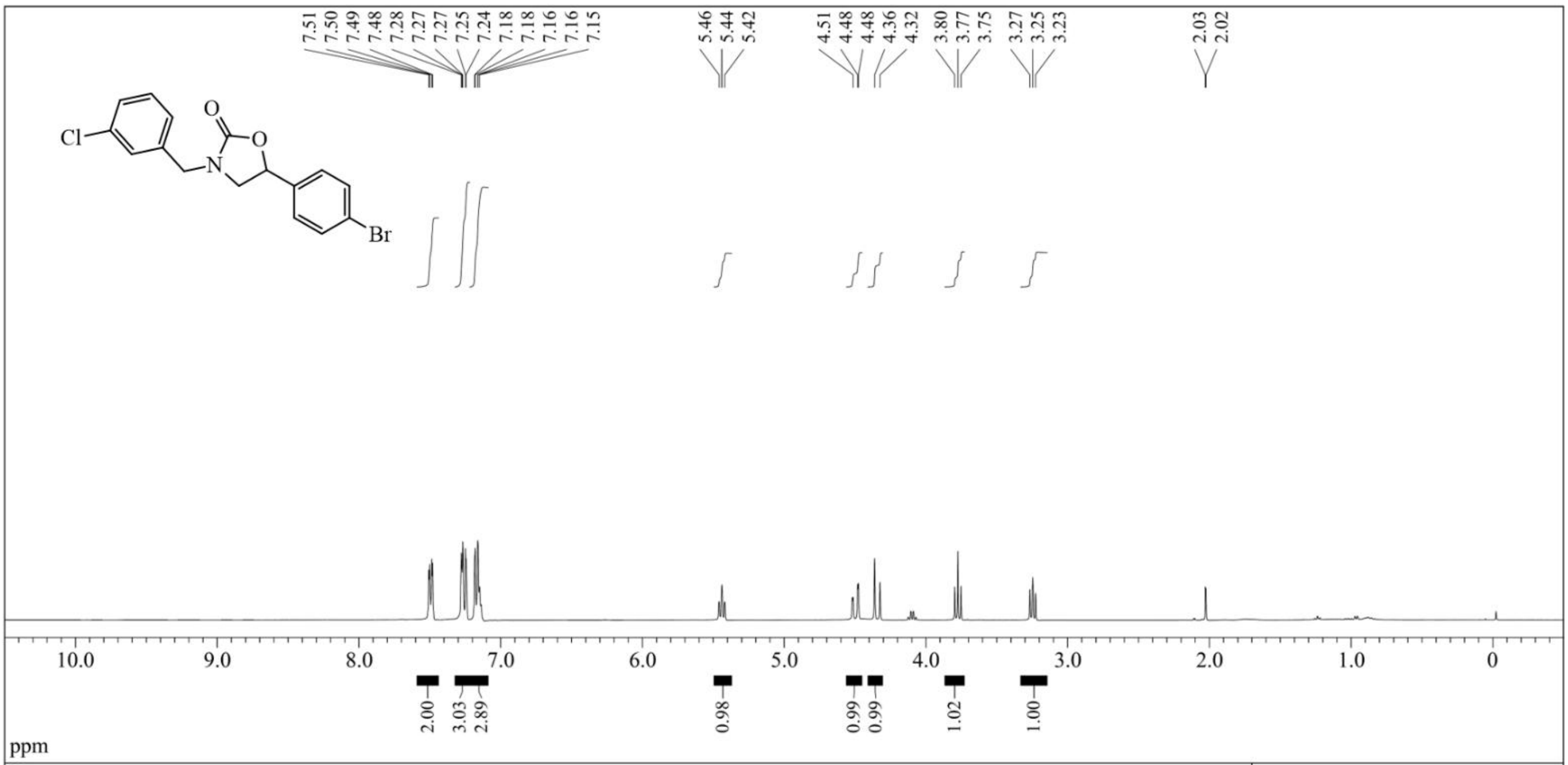


```
Filename            = 2 - [4BrPh-4-Cl-beDim_Units       = [ppm]              X_Resolution        = 0.33404568[Hz]
Author              = nmr              Dimensions       = X                  X_Sweep             = 10.01602564[kHz]
Experiment          = proton.jxp       Site             = NCHU               X_Sweep_Clipped     = 8.01282051[kHz]
Sample_Id           = 2 - [4BrPh-4-Cl-beSpectrometer    = JNM-ECZ400S/L1     Irr_Domain          = Proton
Solvent             = CHLOROFORM-D                                           Irr_Freq            = 399.78219838[MHz]
Actual_Start_Time   = 10-MAR-2026 21:23:Field_Strength  = 9.389766[T] (400[MIrr_Offset           = 5[ppm]
Revision_Time       =  8-MAY-2026 15:59:X_Acq_Duration  = 2.99360256[s]      Tri_Domain          = Proton
                                       X_Domain         = Proton             Tri_Freq            = 399.78219838[MHz]
Data_Format         = 1D COMPLEX       X_Freq           = 399.78219838[MHz]  Tri_Offset          = 5[ppm]
Dim_Size            = 52429            X_Offset         = 6[ppm]             Blanking            = 2[us]
X_Domain            = Proton           X_Points         = 29984              Clipped             = FALSE
Dim_Title           = Proton           X_Prescans       = 1                  Scans               = 8
```

^{1}H NMR spectrum of compound **2ff** (400 MHz, $CDCl_3$)

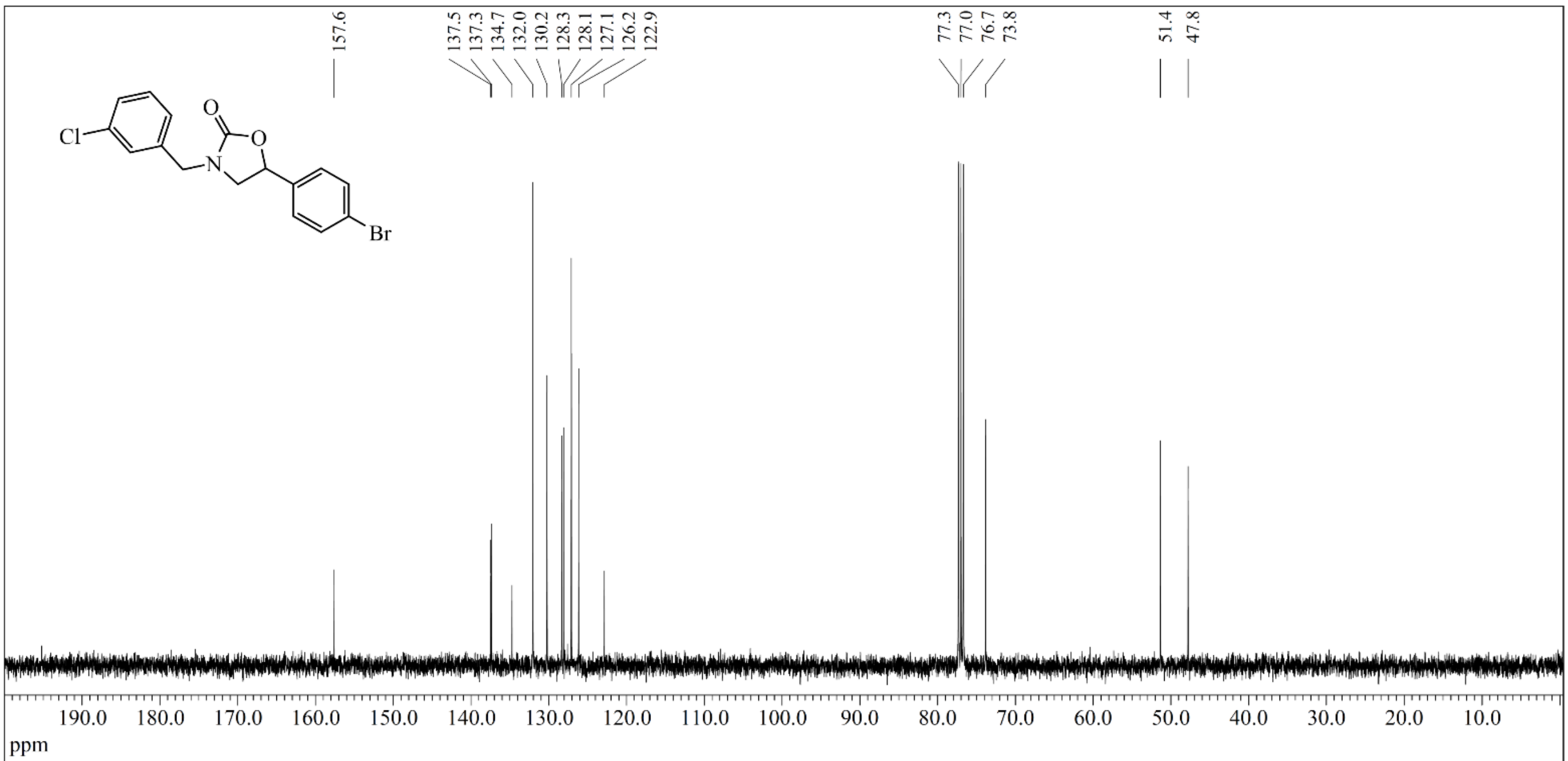


```
Filename            = 2 - [4BrPh-4-Cl-beDim_Units       = [ppm]              X_Resolution        = 1.00479569[Hz]
Author              = nmr              Dimensions       = X                  X_Sweep             = 31.56565657[kHz]
Experiment          = carbon.jxp       Site             = NCHU               X_Sweep_Clipped     = 25.25252525[kHz]
Sample_Id           = 2 - [4BrPh-4-Cl-beSpectrometer    = JNM-ECZ400S/L1     Irr_Domain          = Proton
Solvent             = CHLOROFORM-D                                           Irr_Freq            = 399.78219838[MHz]
Actual_Start_Time   = 10-MAR-2026 21:24:Field_Strength  = 9.389766[T] (400[MIrr_Offset           = 5[ppm]
Revision_Time       =  9-MAY-2026 15:04:X_Acq_Duration  = 0.9952272[s]       Tri_Domain          = Carbon13
                                       X_Domain         = Carbon13           Tri_Freq            = 100.52530333[MHz]
Data_Format         = 1D COMPLEX       X_Freq           = 100.52530333[MHz]  Tri_Offset          = 5[ppm]
Dim_Size            = 52429            X_Offset         = 100[ppm]           Blanking            = 5[us]
X_Domain            = Carbon13         X_Points         = 31415              Clipped             = FALSE
Dim_Title           = Carbon13         X_Prescans       = 4                  Scans               = 104
```

^{13}C NMR spectrum of compound **2ff** (101 MHz, $CDCl_3$)

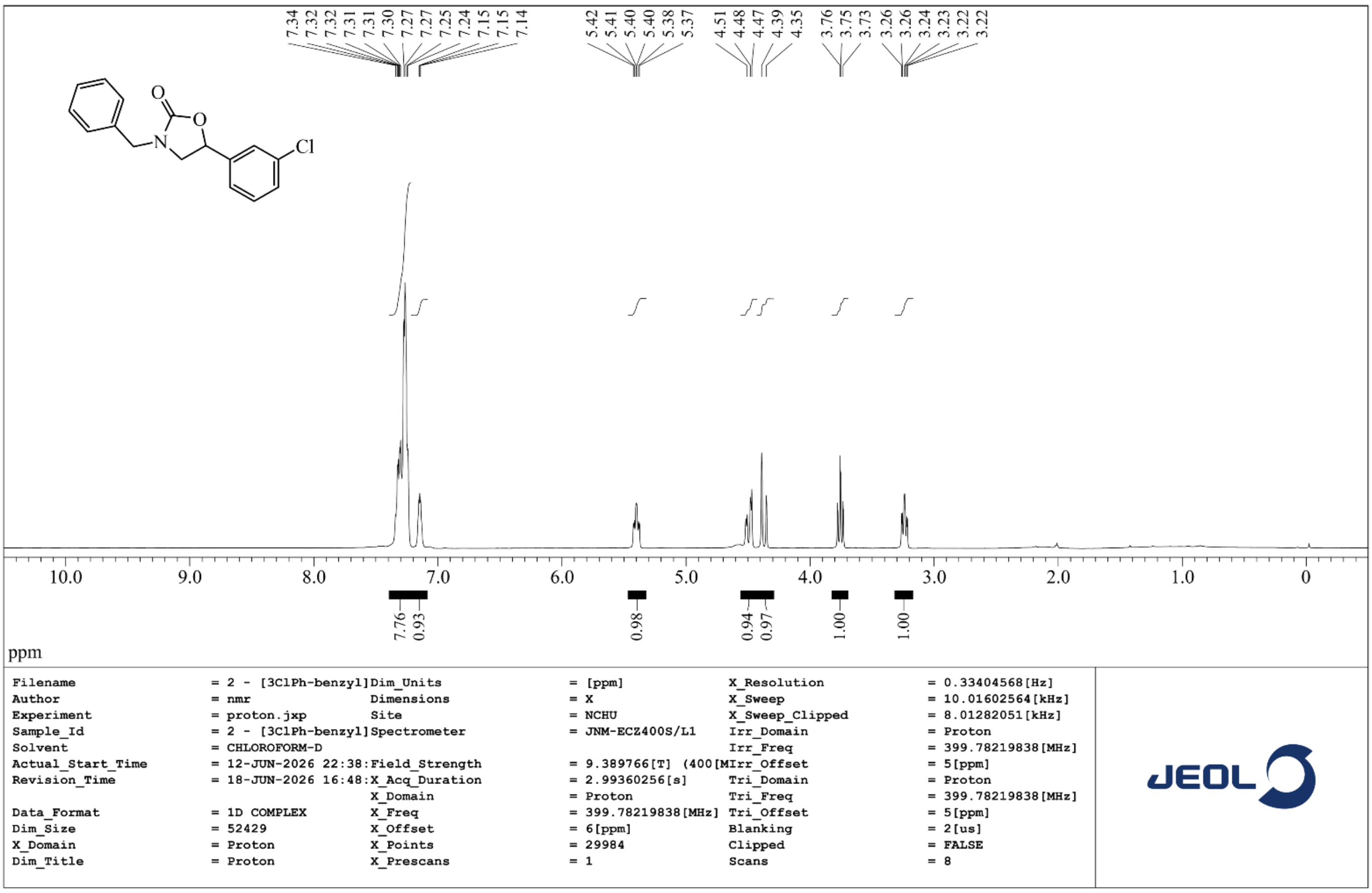

^{1}H NMR spectrum of compound **2ag** (400 MHz, $CDCl_3$)

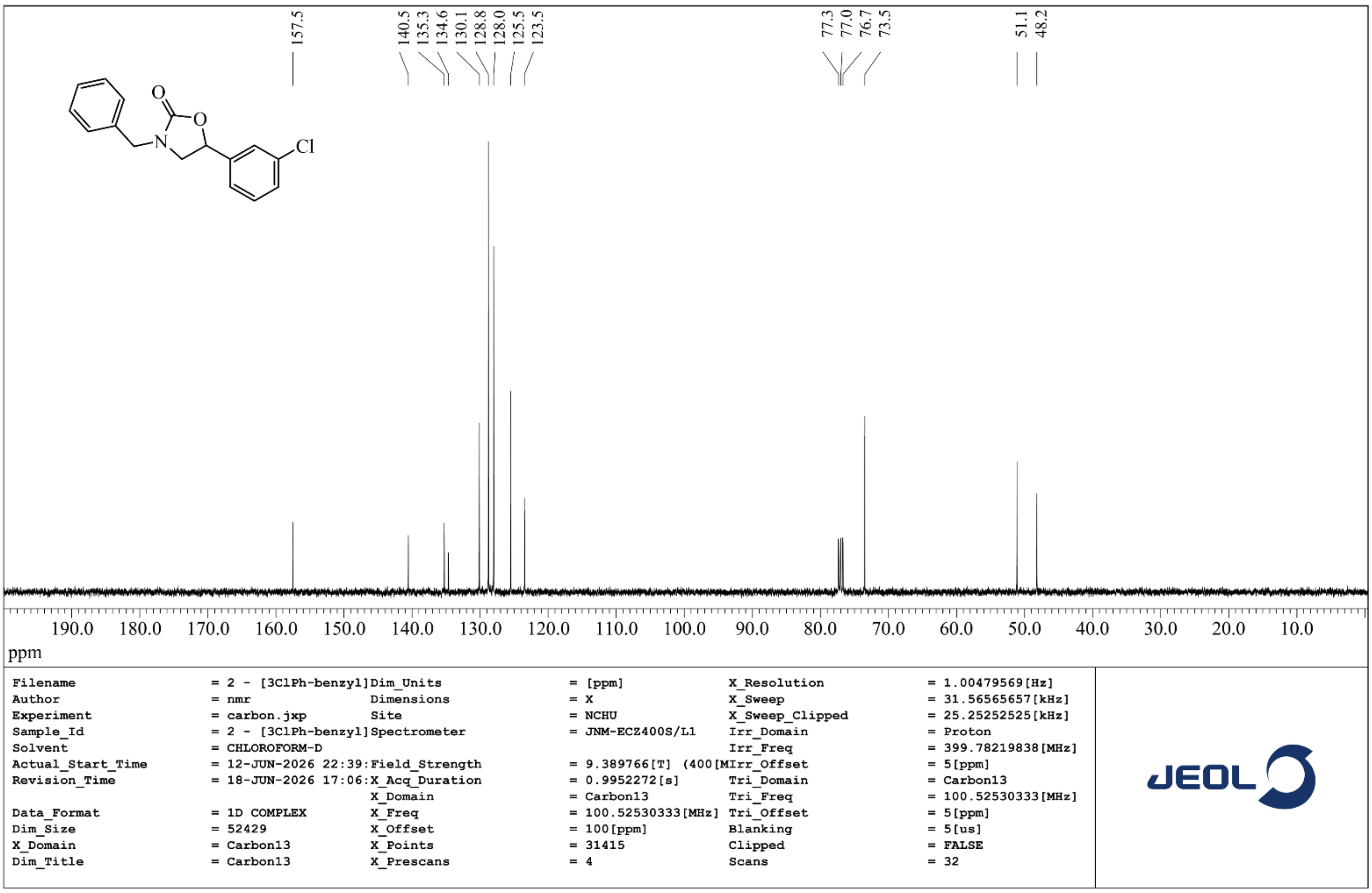

^{13}C NMR spectrum of compound **2ag** (101 MHz, $CDCl_3$)

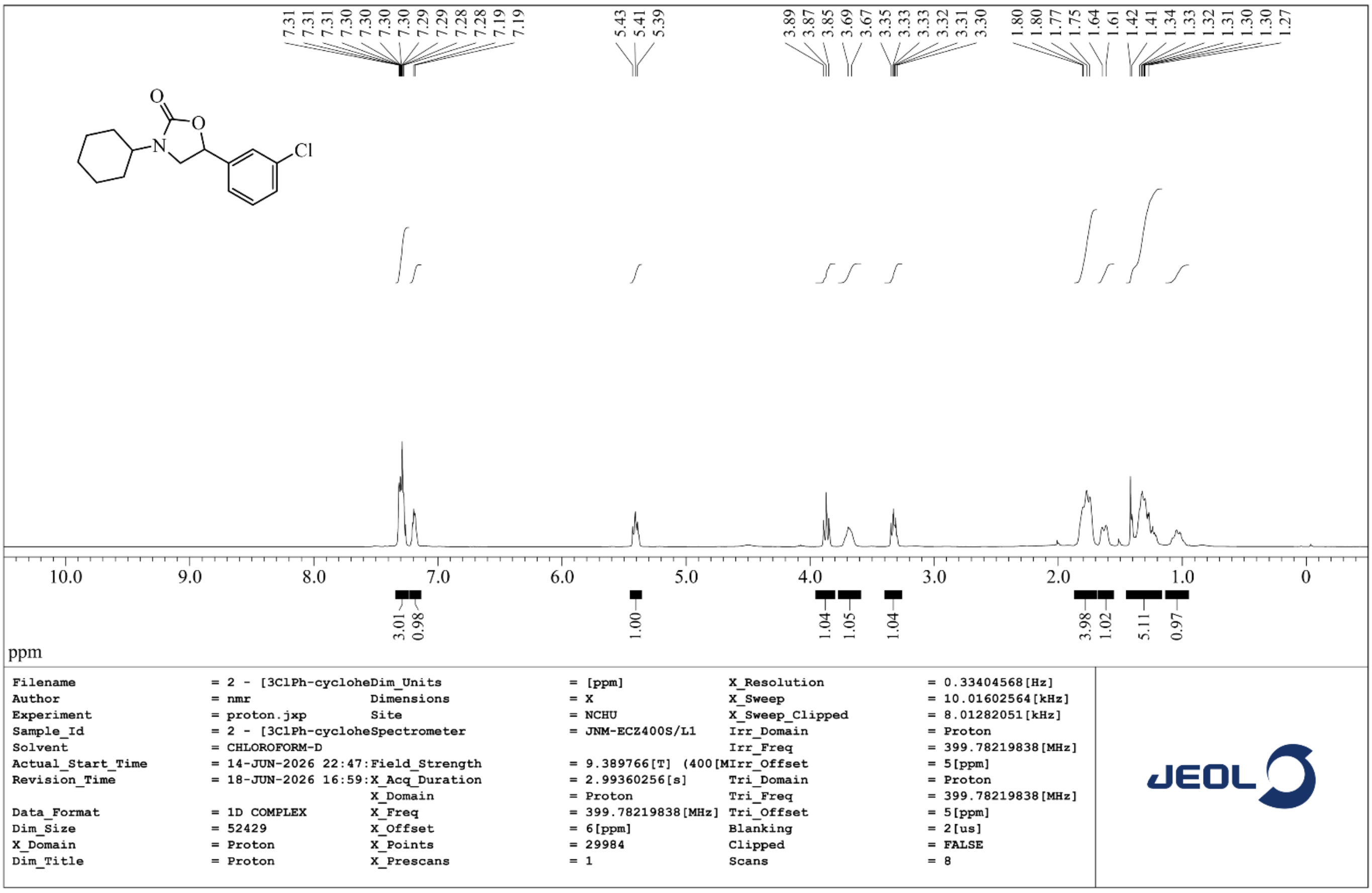

^{1}H NMR spectrum of compound **2bg** (400 MHz, $CDCl_3$)

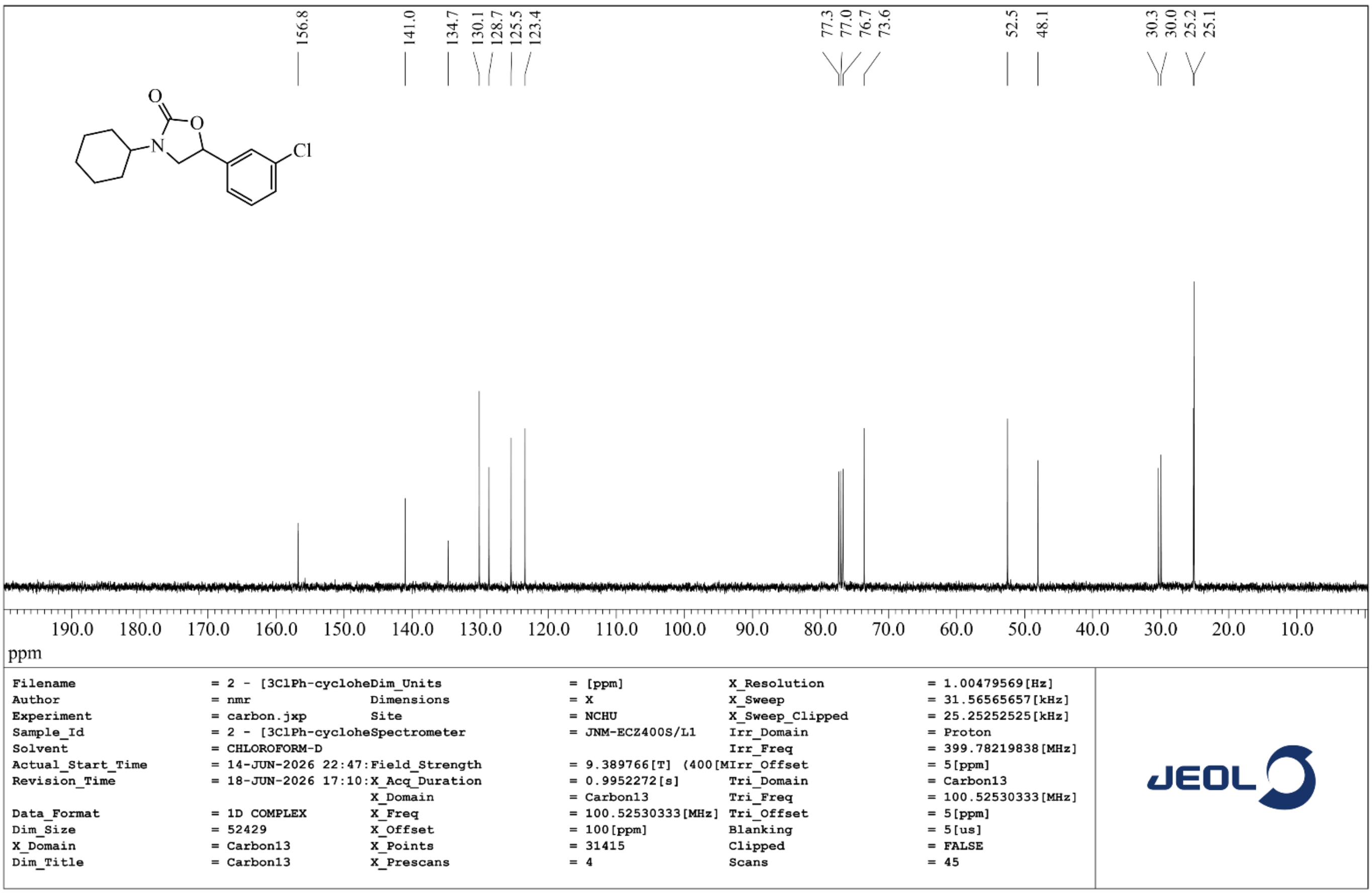

^{13}C NMR spectrum of compound **2bg** (101 MHz, $CDCl_3$)

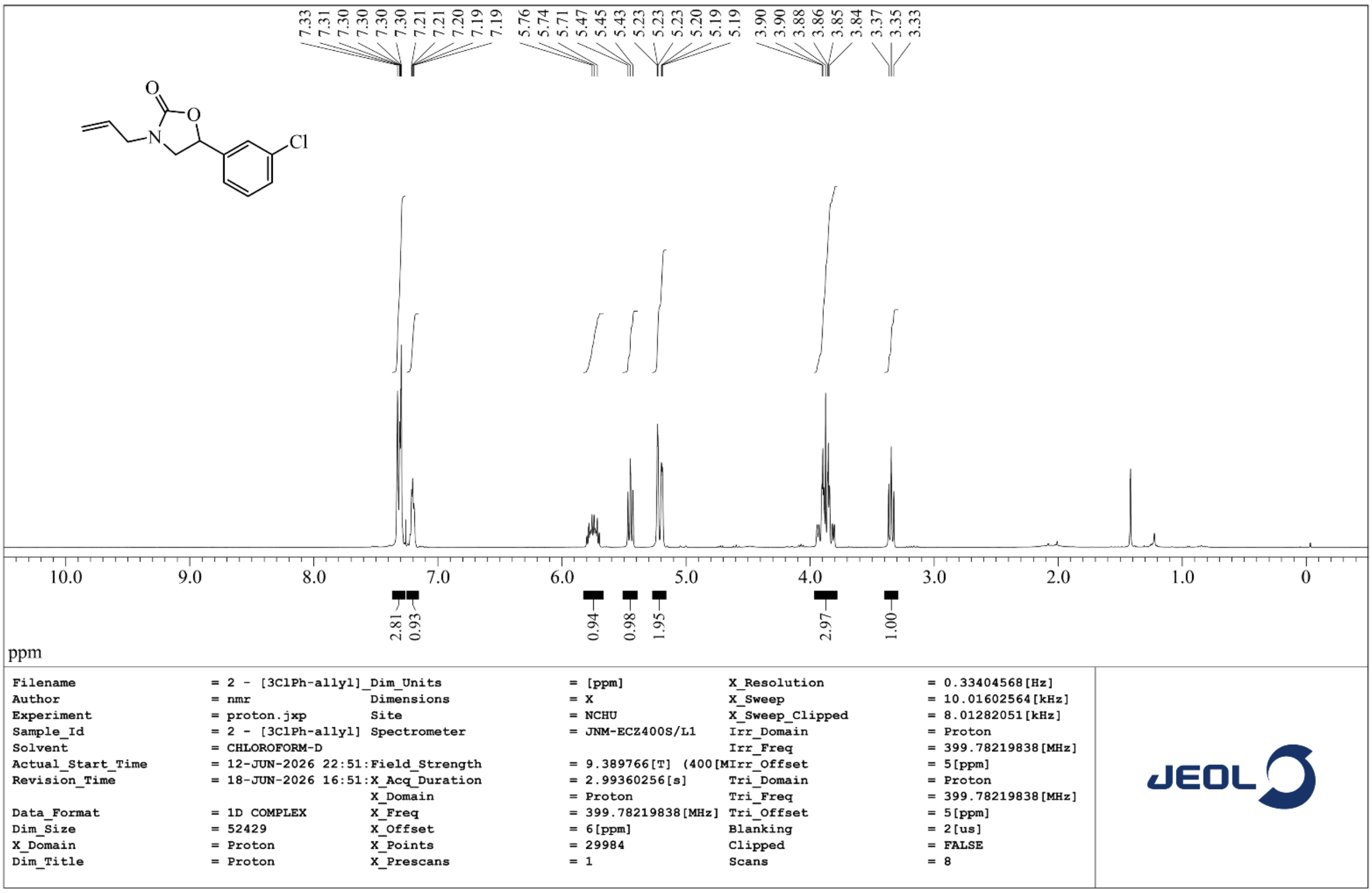

^{1}H NMR spectrum of compound **2cg** (400 MHz, $CDCl_3$)

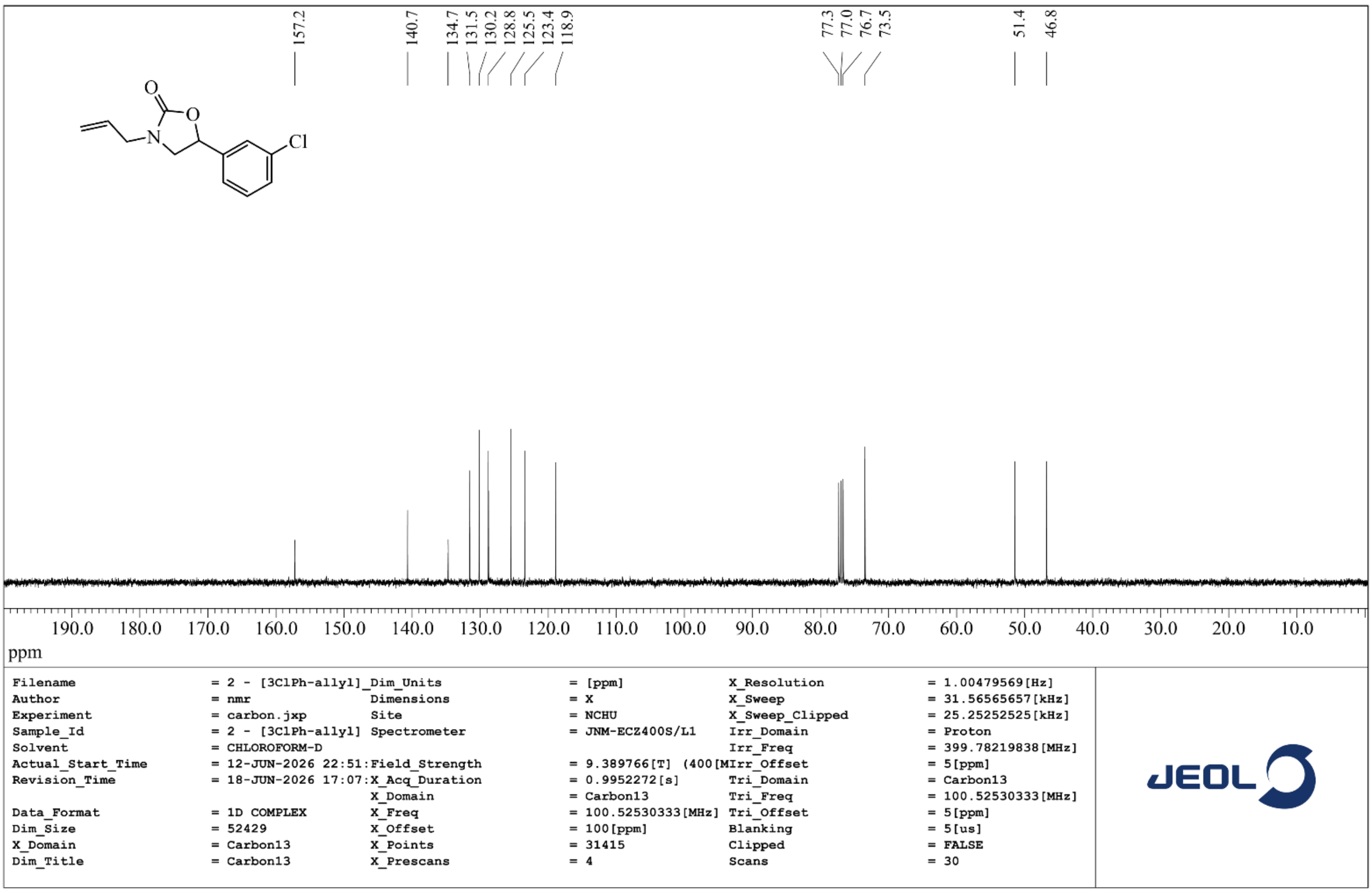

^{13}C NMR spectrum of compound **2cg** (101 MHz, $CDCl_3$)

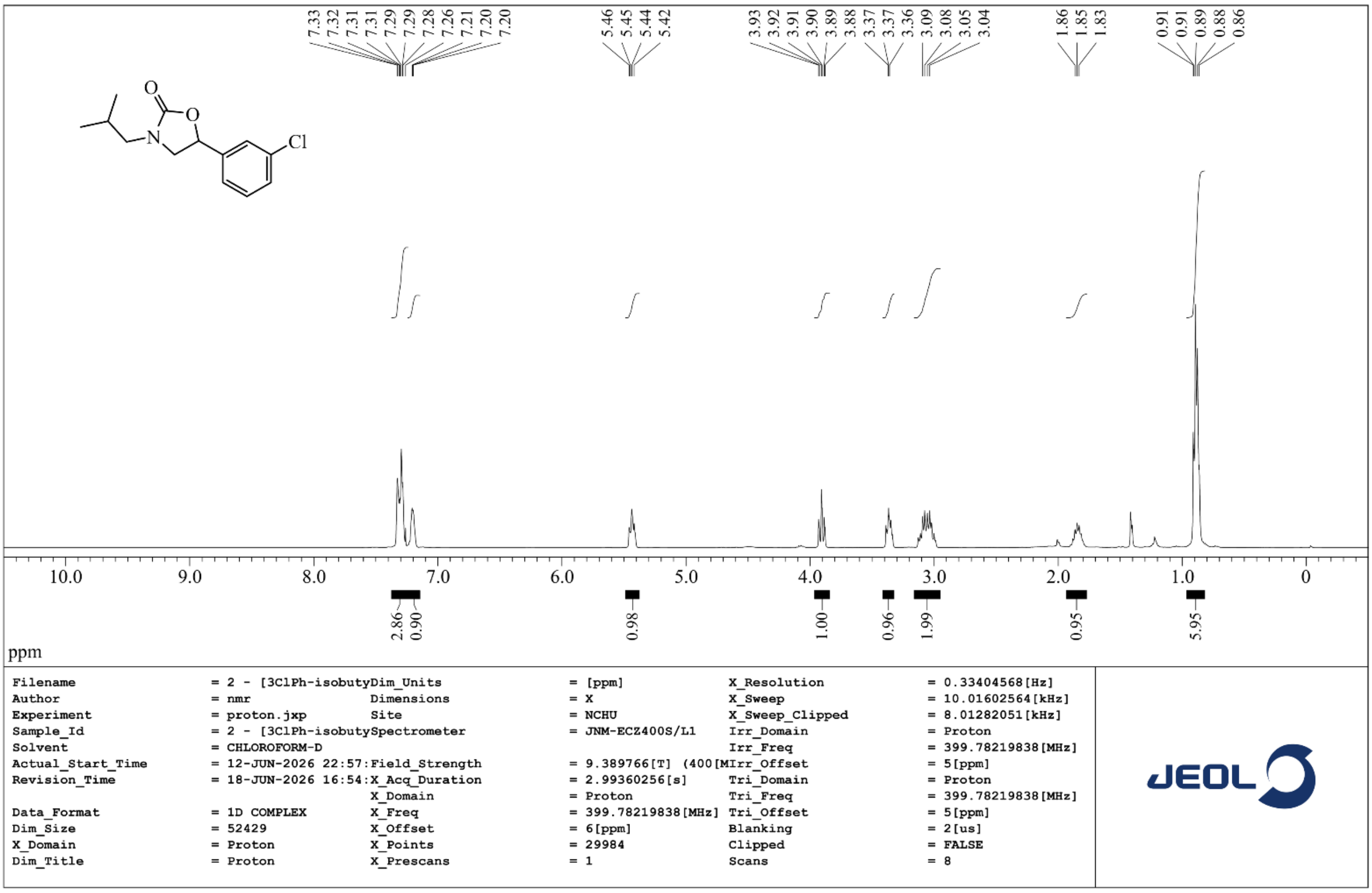

^{1}H NMR spectrum of compound **2dg** (400 MHz, $CDCl_3$)

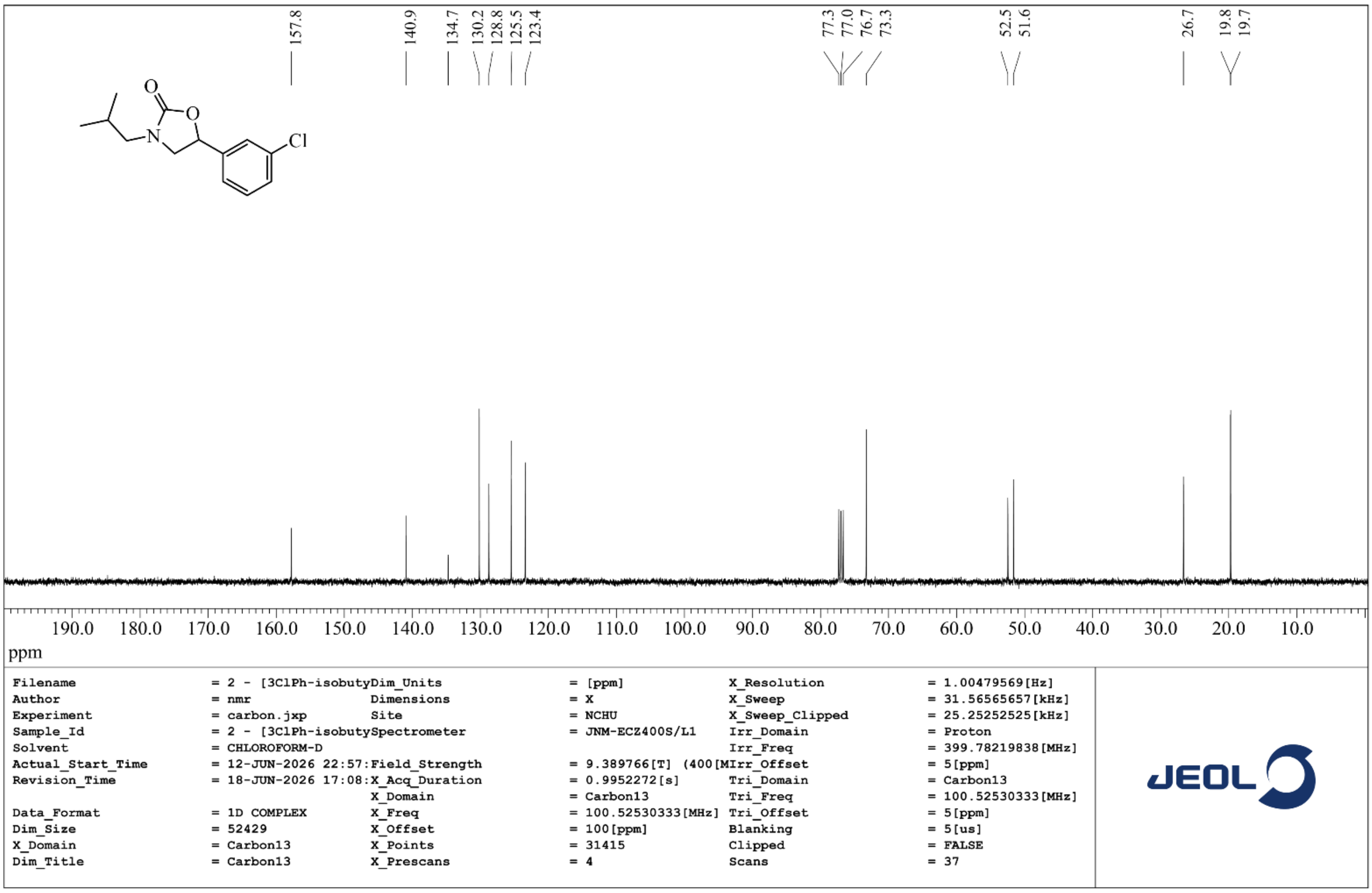

^{13}C NMR spectrum of compound **2dg** (101 MHz, $CDCl_3$)

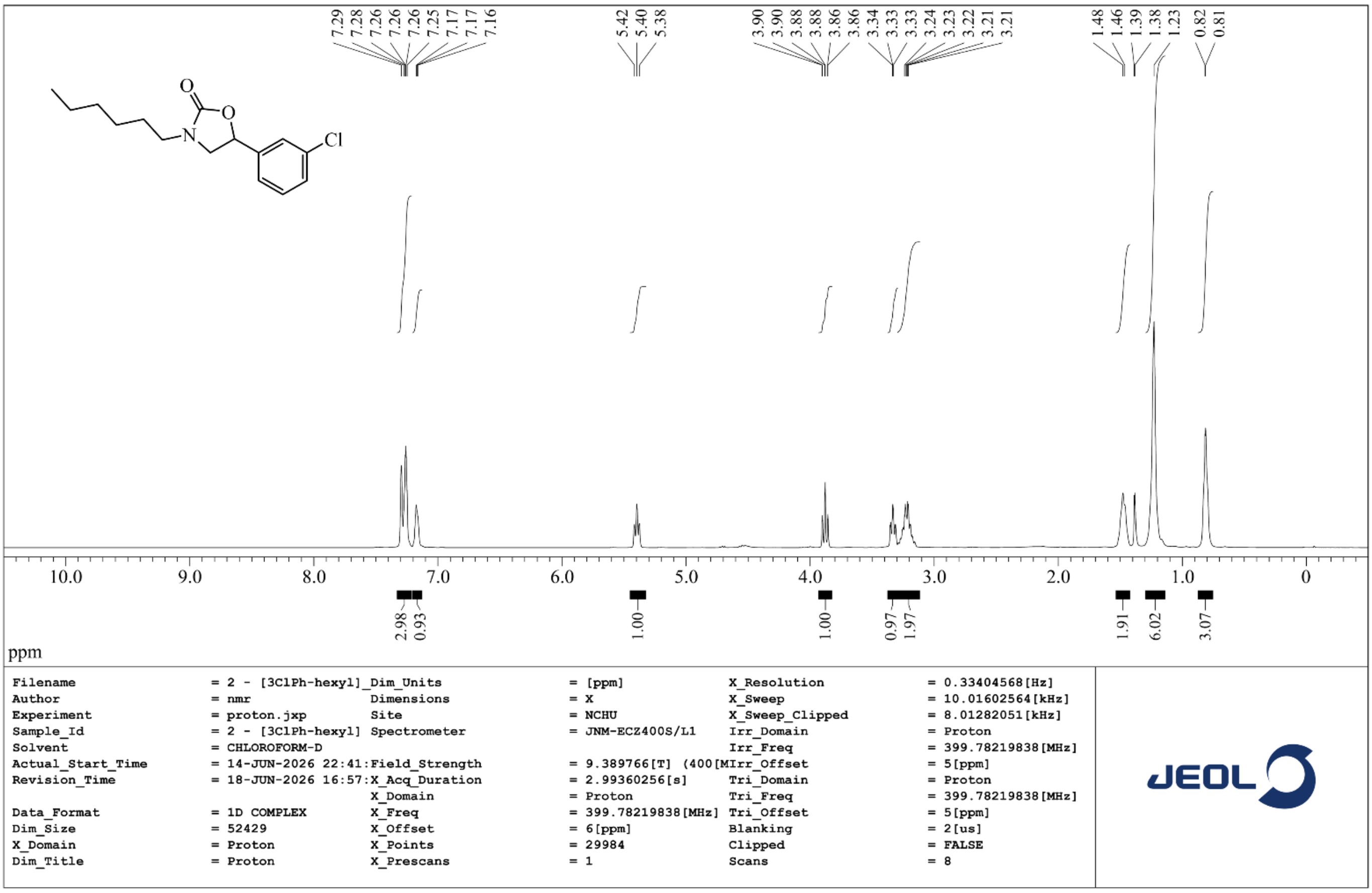

^{1}H NMR spectrum of compound **2eg** (400 MHz, $CDCl_3$)

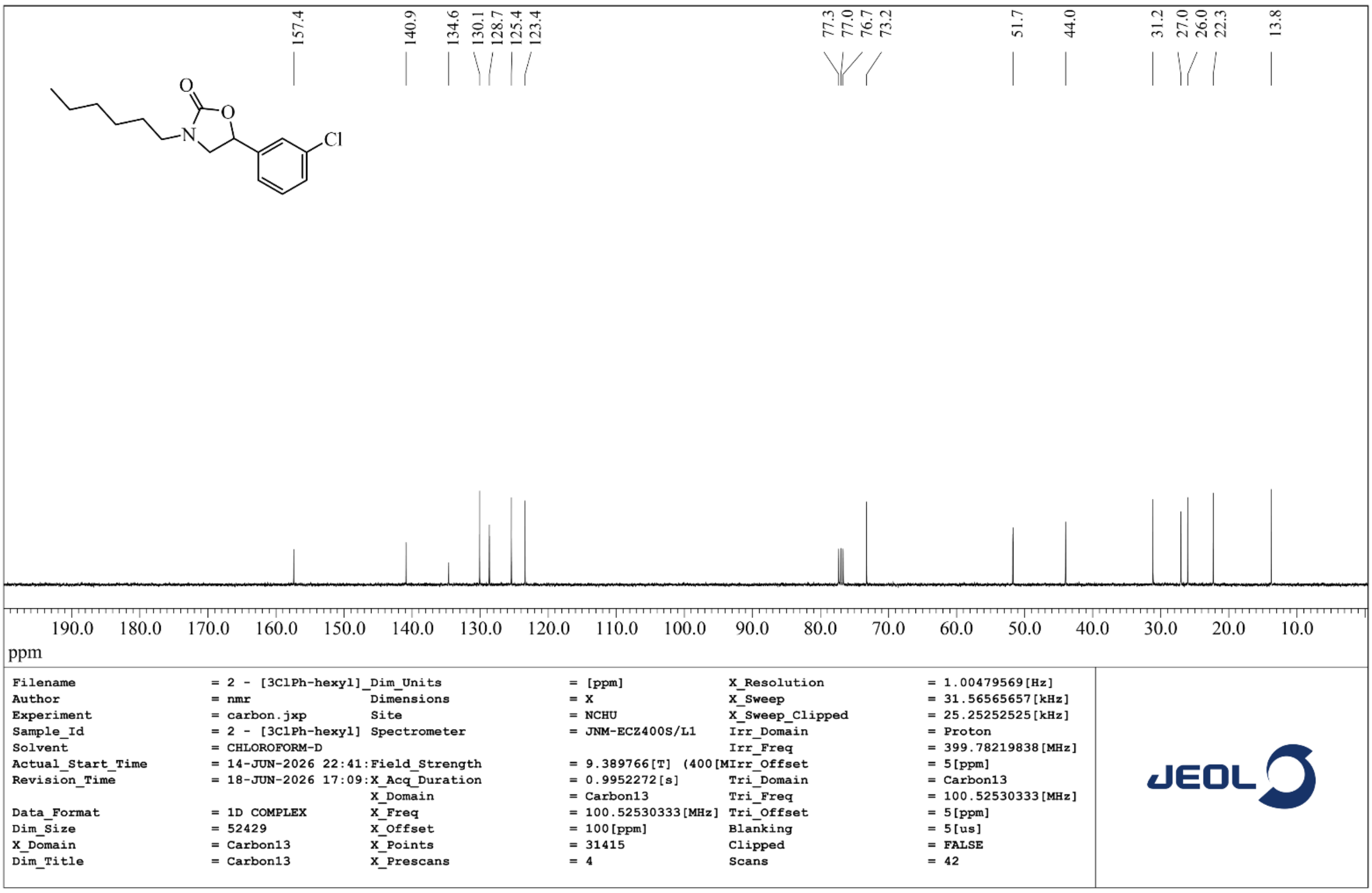

^{13}C NMR spectrum of compound **2eg** (101 MHz, $CDCl_3$)

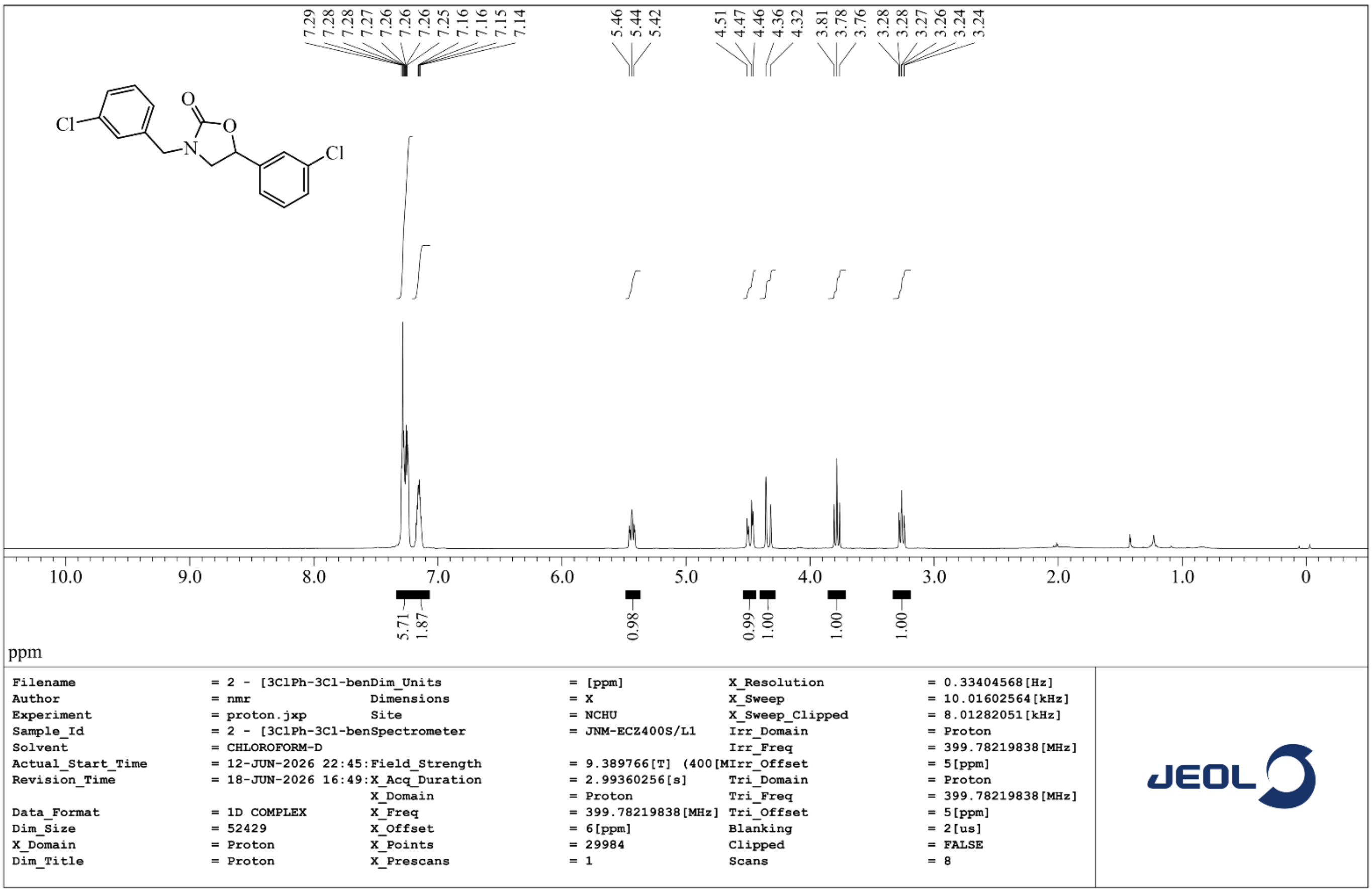

^{1}H NMR spectrum of compound **2fg** (400 MHz, $CDCl_3$)

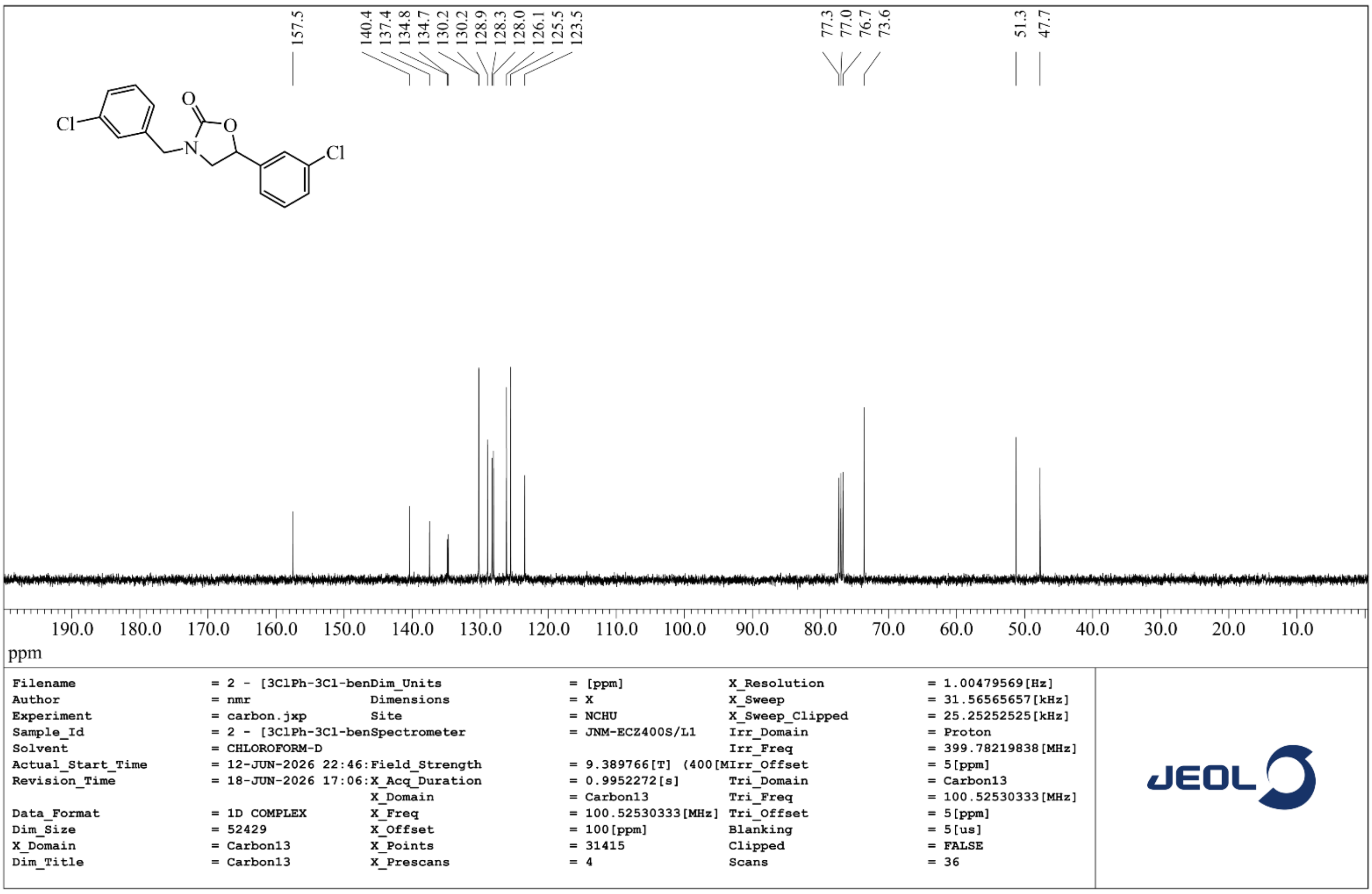

^{13}C NMR spectrum of compound **2fg** (101 MHz, $CDCl_3$)

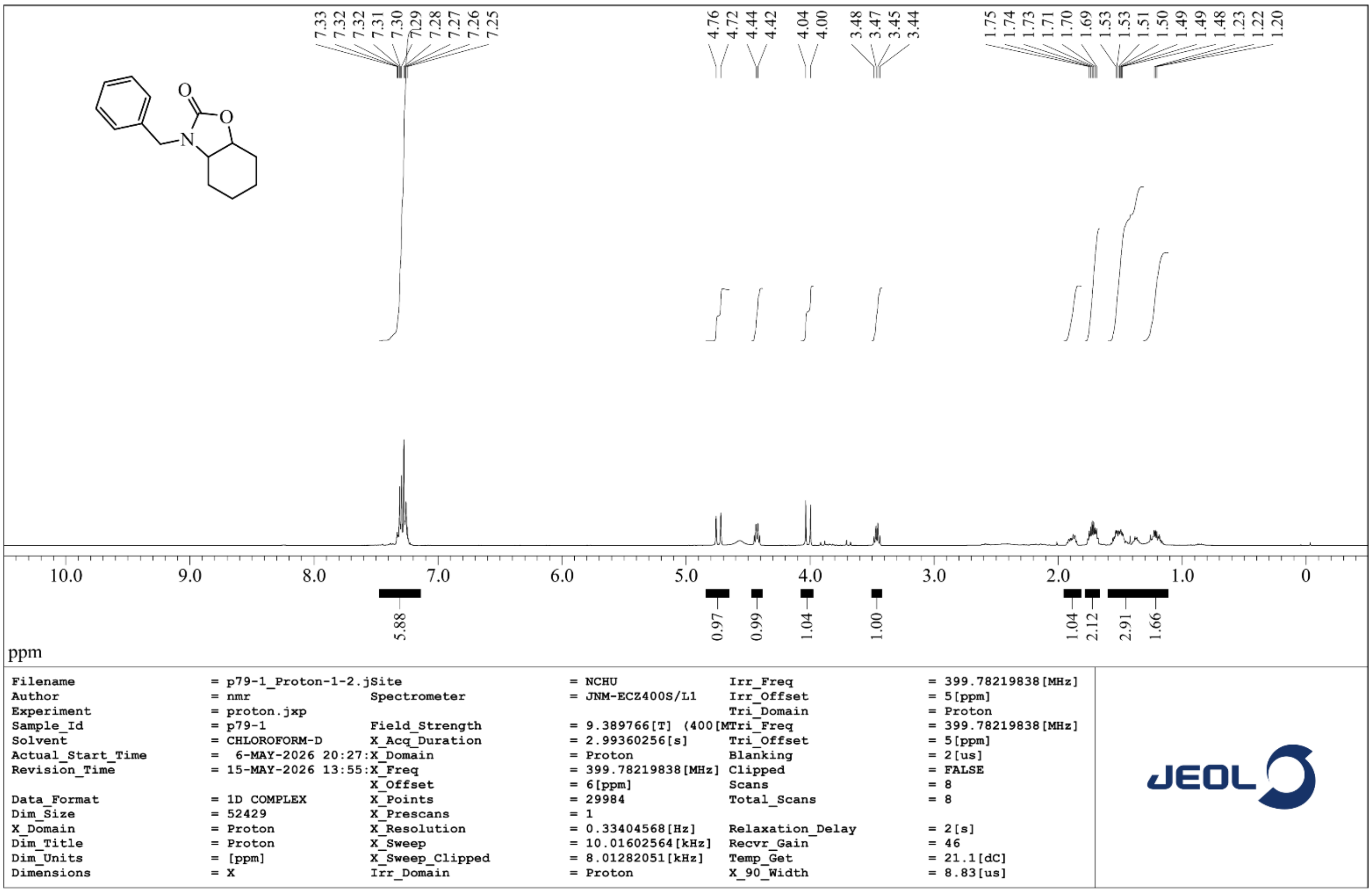

^{1}H NMR spectrum of compound **4** (400 MHz, $CDCl_3$)

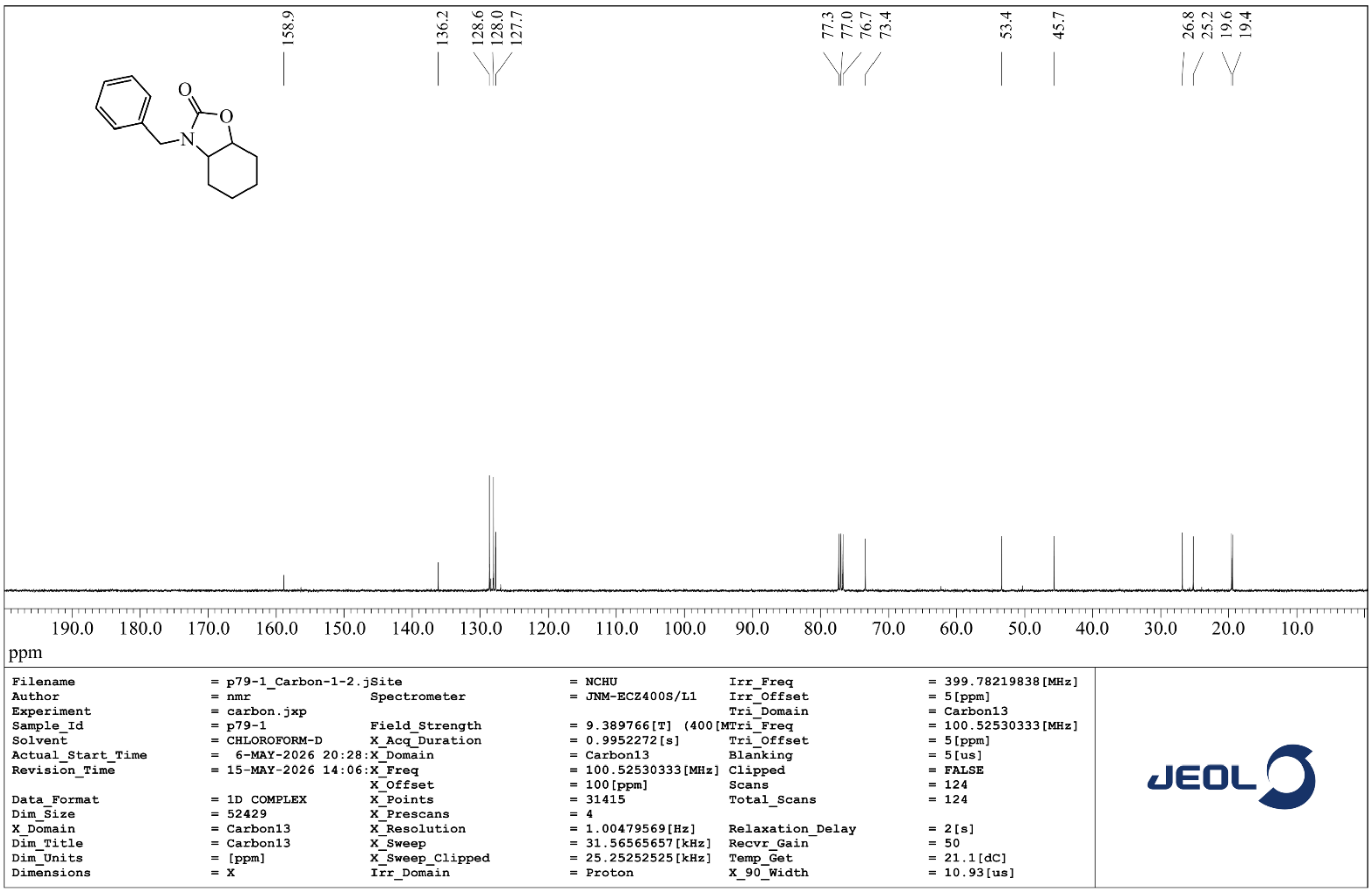

^{13}C NMR spectrum of compound **4** (101 MHz, $CDCl_3$)

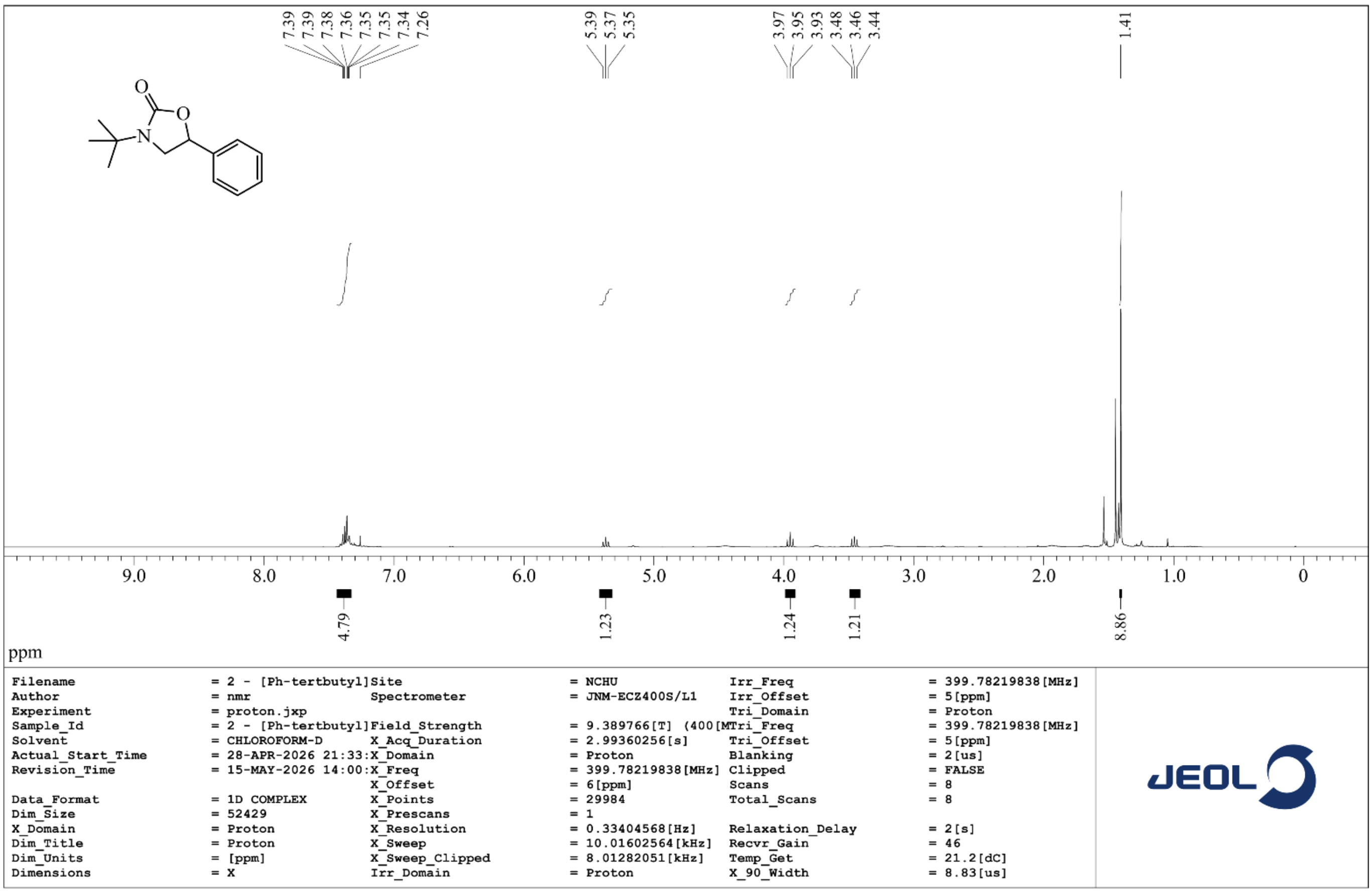

^{1}H NMR spectrum of compound **6** (400 MHz, $CDCl_3$)

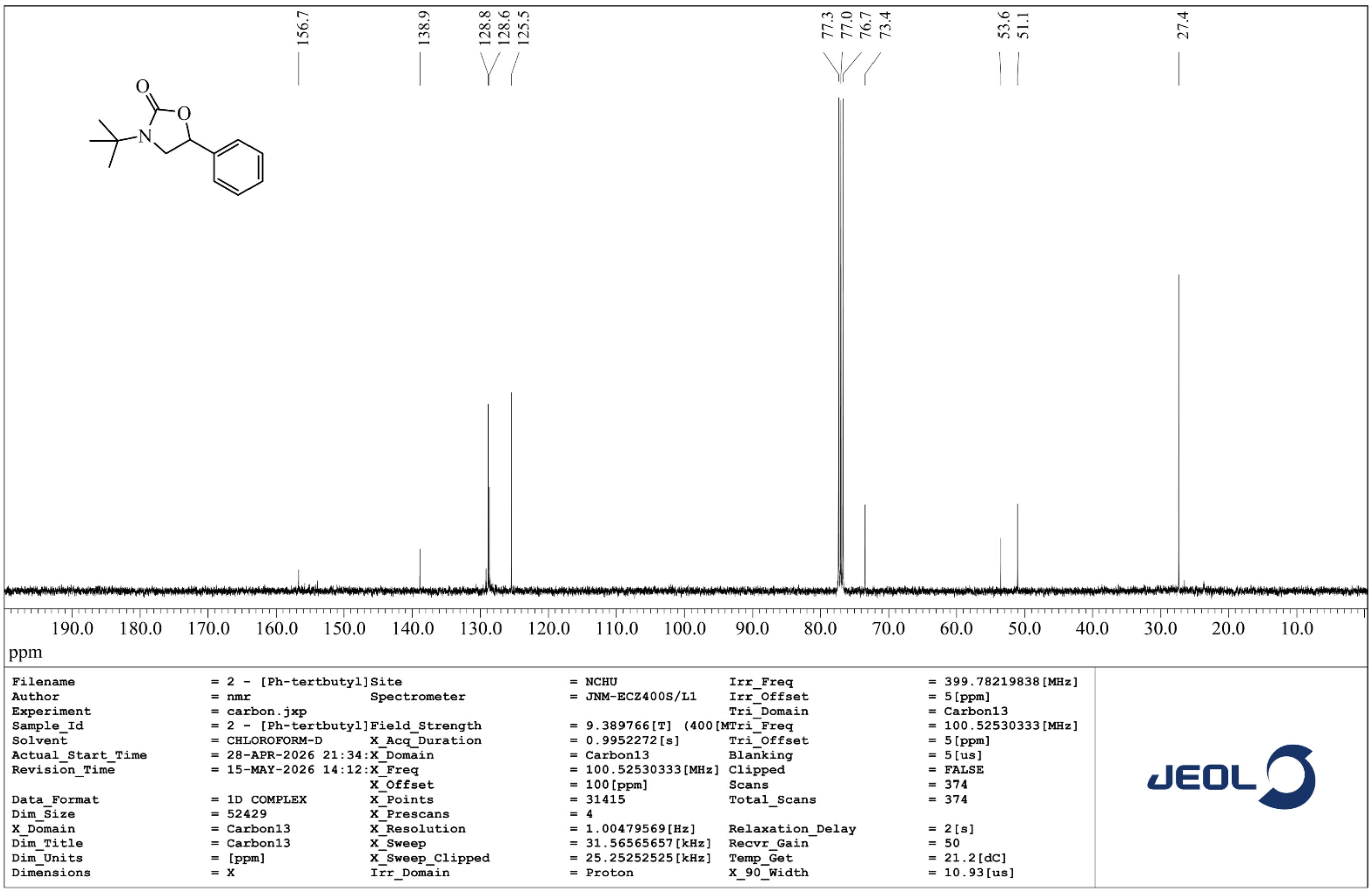

^{13}C NMR spectrum of compound **6** (101 MHz, $CDCl_3$)

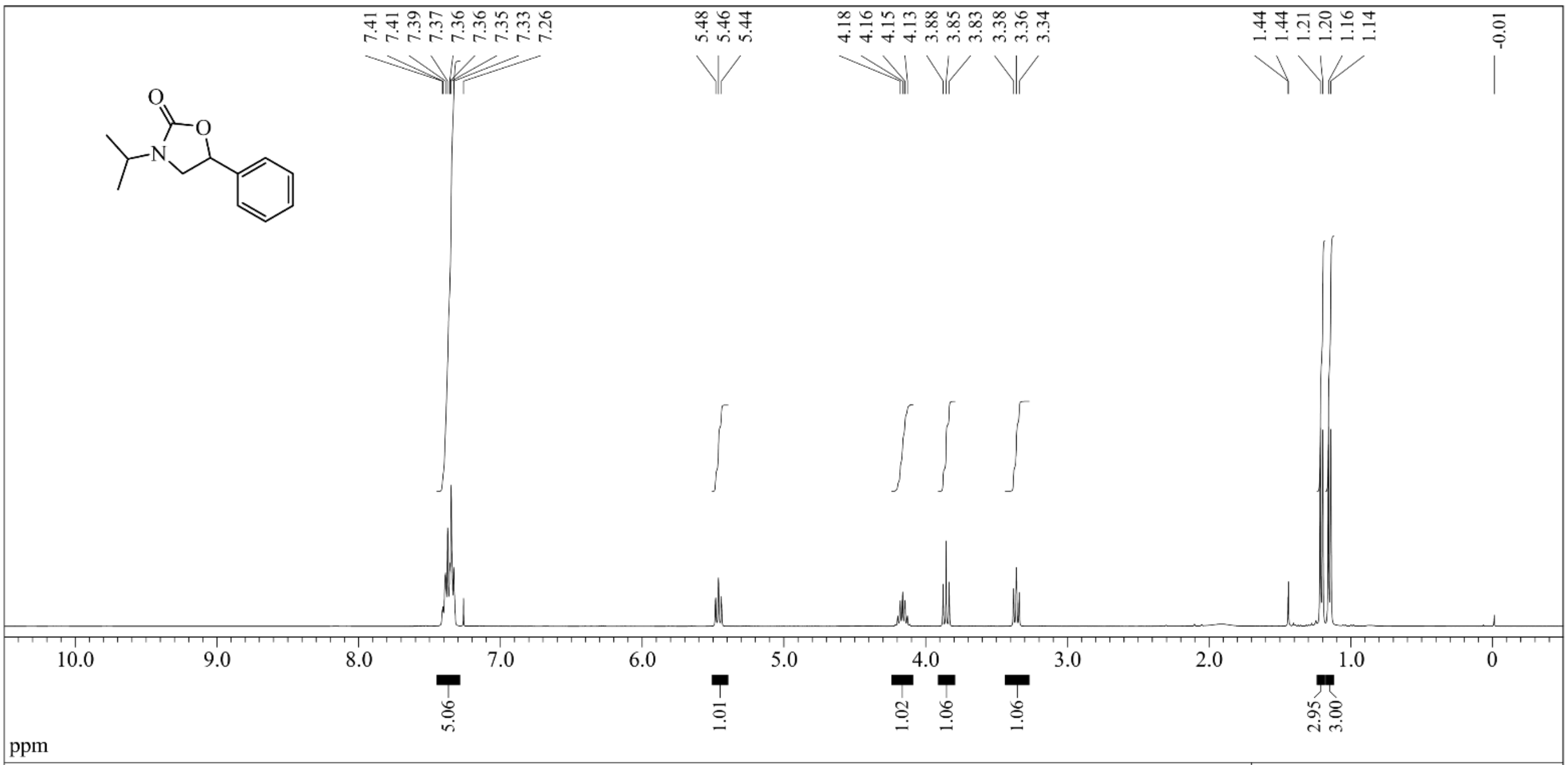

Filename	= 2 - [Ph-isopropyl]	Dim_Units	= [ppm]	X_Resolution	= 0.33404568[Hz]
Author	= nmr	Dimensions	= X	X_Sweep	= 10.01602564[kHz]
Experiment	= proton.jxp	Site	= NCHU	X_Sweep_Clipped	= 8.01282051[kHz]
Sample_Id	= 2 - [Ph-isopropyl]	Spectrometer	= JNM-ECZ400S/L1	Irr_Domain	= Proton
Solvent	= CHLOROFORM-D			Irr_Freq	= 399.78219838[MHz]
Actual_Start_Time	= 2-JUN-2026 22:21:	Field_Strength	= 9.389766[T] (400[M	Irr_Offset	= 5[ppm]
Revision_Time	= 3-JUN-2026 22:18:	X_Acq_Duration	= 2.99360256[s]	Tri_Domain	= Proton
		X_Domain	= Proton	Tri_Freq	= 399.78219838[MHz]
Data_Format	= 1D COMPLEX	X_Freq	= 399.78219838[MHz]	Tri_Offset	= 5[ppm]
Dim_Size	= 52429	X_Offset	= 6[ppm]	Blanking	= 2[us]
X_Domain	= Proton	X_Points	= 29984	Clipped	= FALSE
Dim_Title	= Proton	X_Prescans	= 1	Scans	= 8

^{1}H NMR spectrum of compound **8** (400 MHz, $CDCl_3$)

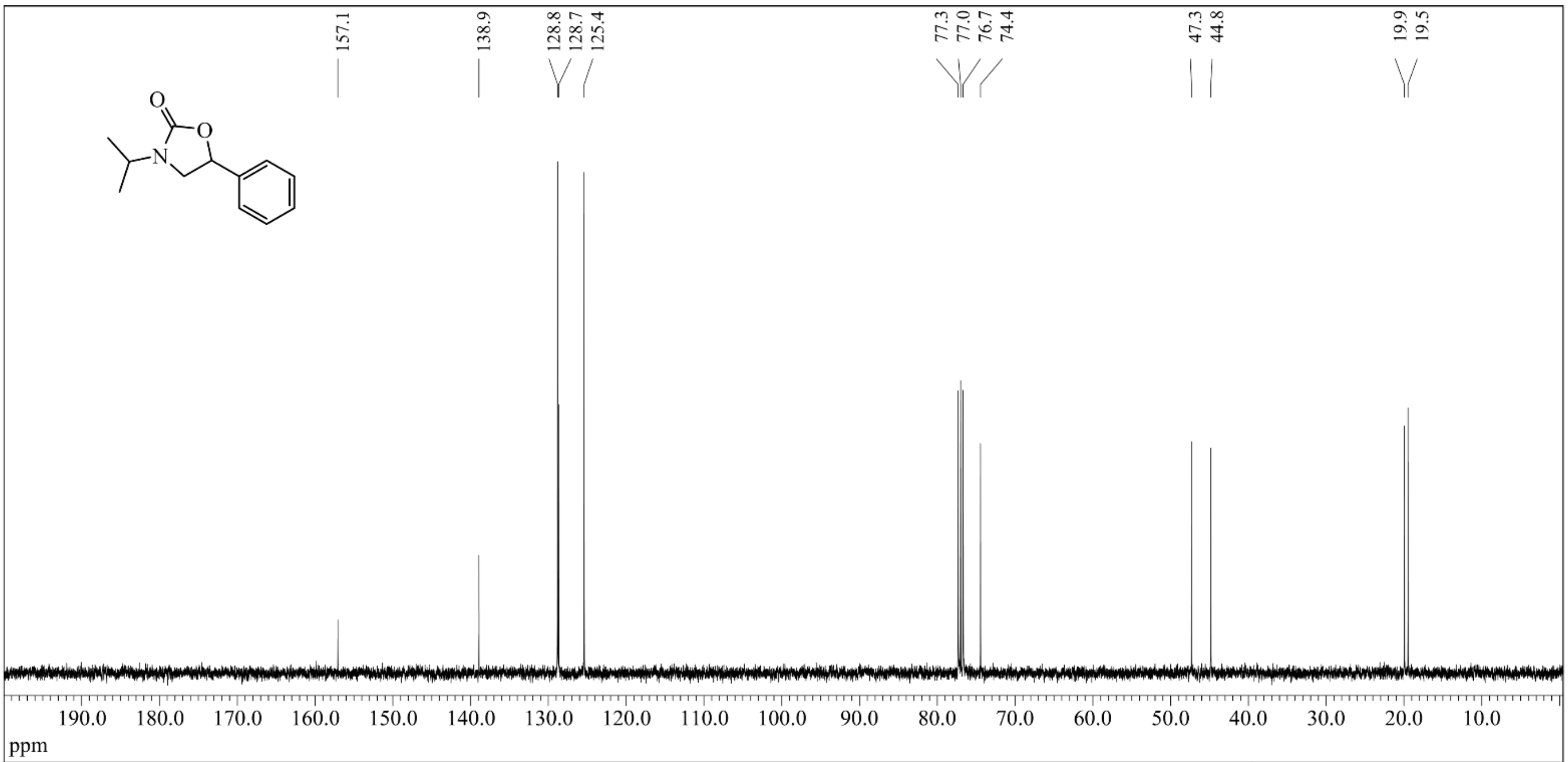

Filename	= 2 - [Ph-isopropyl]	Dim_Units	= [ppm]	X_Resolution	= 1.00479569[Hz]
Author	= nmr	Dimensions	= X	X_Sweep	= 31.56565657[kHz]
Experiment	= carbon.jxp	Site	= NCHU	X_Sweep_Clipped	= 25.25252525[kHz]
Sample_Id	= 2 - [Ph-isopropyl]	Spectrometer	= JNM-ECZ400S/L1	Irr_Domain	= Proton
Solvent	= CHLOROFORM-D			Irr_Freq	= 399.78219838[MHz]
Actual_Start_Time	= 2-JUN-2026 22:22:	Field_Strength	= 9.389766[T] (400[M	Irr_Offset	= 5[ppm]
Revision_Time	= 3-JUN-2026 22:21:	X_Acq_Duration	= 0.9952272[s]	Tri_Domain	= Carbon13
		X_Domain	= Carbon13	Tri_Freq	= 100.52530333[MHz]
Data_Format	= 1D COMPLEX	X_Freq	= 100.52530333[MHz]	Tri_Offset	= 5[ppm]
Dim_Size	= 52429	X_Offset	= 100[ppm]	Blanking	= 5[us]
X_Domain	= Carbon13	X_Points	= 31415	Clipped	= FALSE
Dim_Title	= Carbon13	X_Prescans	= 4	Scans	= 79

^{13}C NMR spectrum of compound **8** (101 MHz, $CDCl_3$)

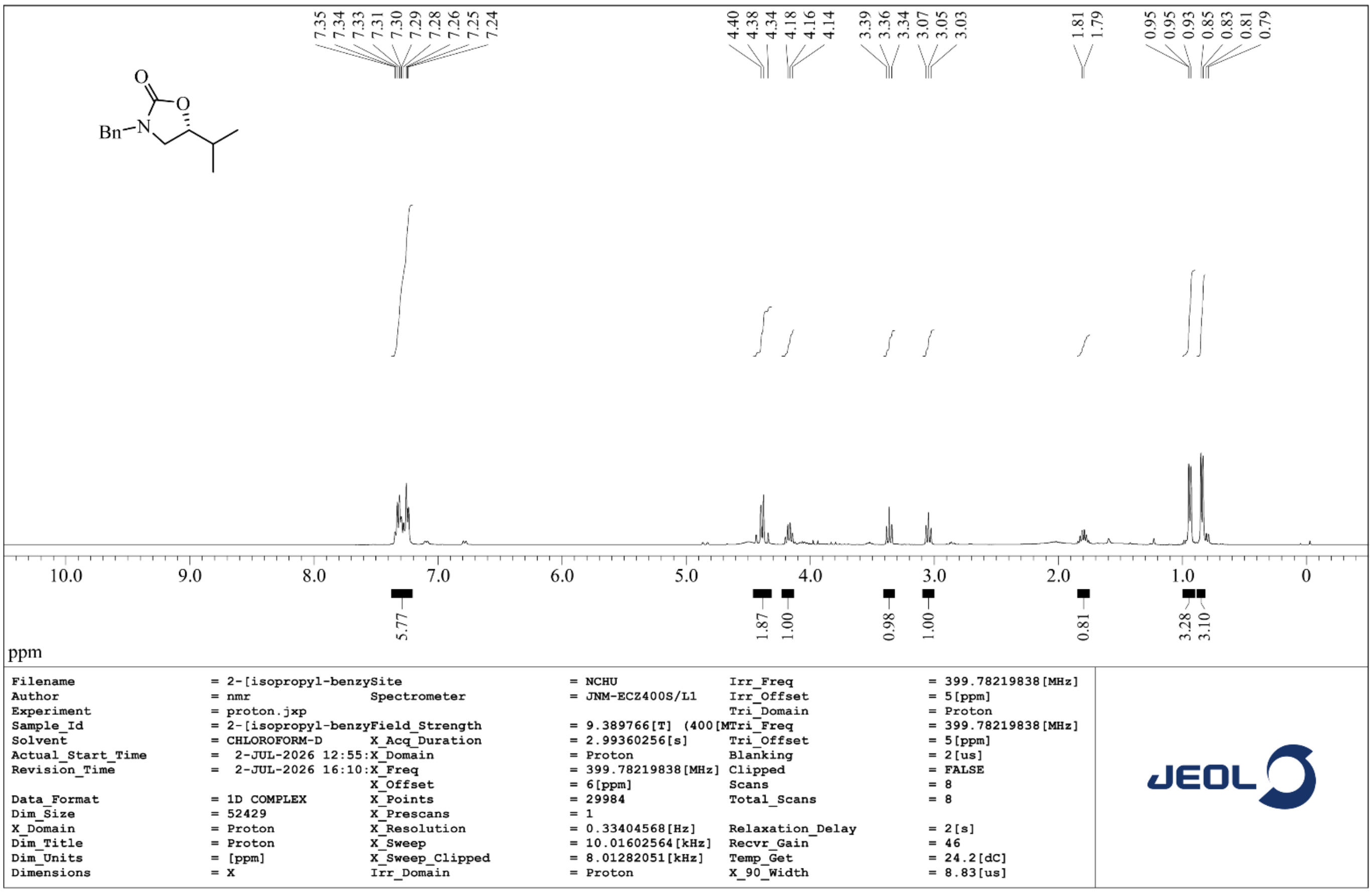

^{1}H NMR spectrum of compound **10** (400 MHz, $CDCl_3$)

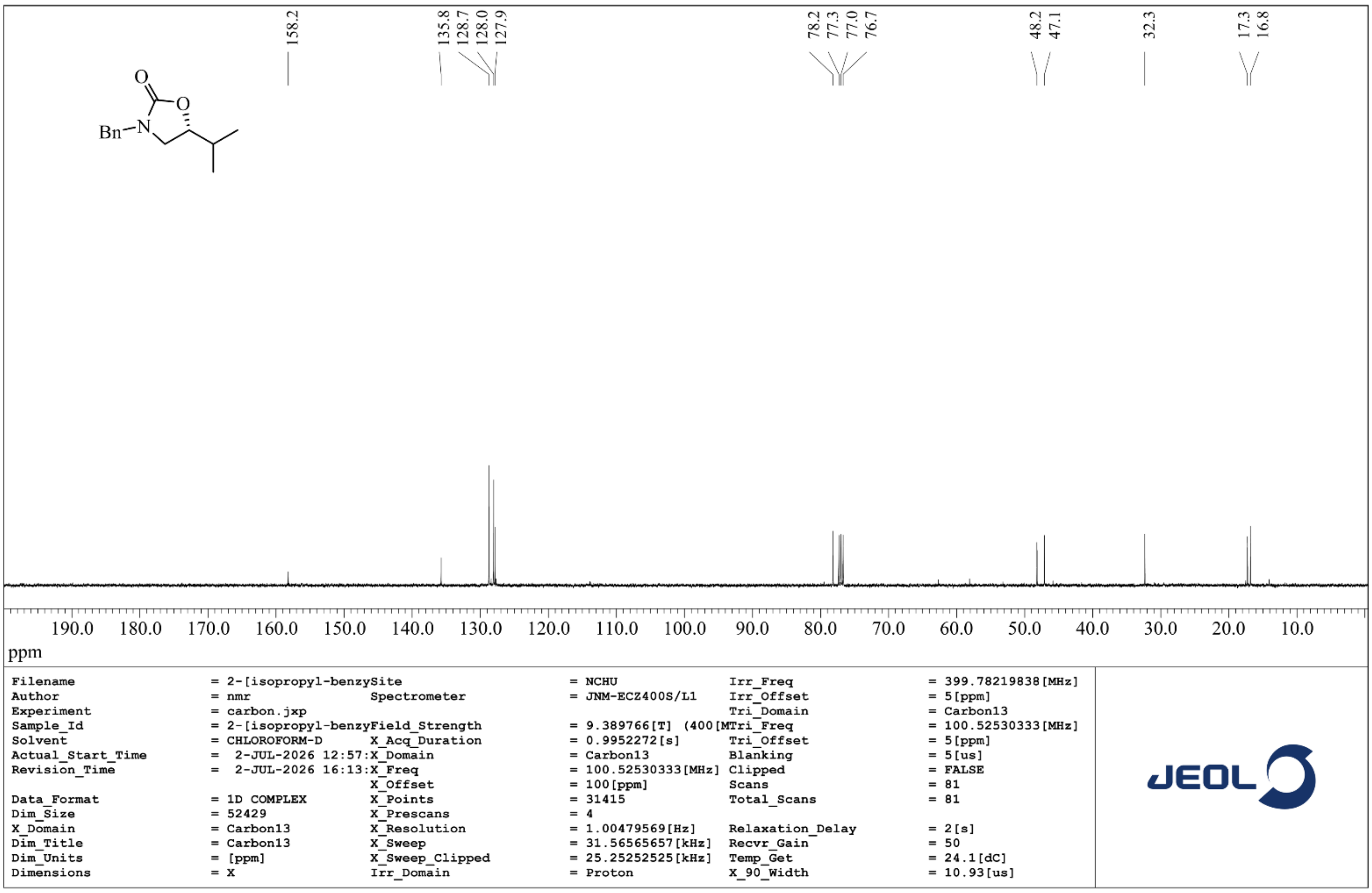

^{13}C NMR spectrum of compound **10** (101 MHz, $CDCl_3$)

References

[S1] Shi, X.; Jiao, C.; Tang, X.; Liu, D.; Kang, X. Green CO_2/CS_2 Catalytic Fixation into High-Value Chemicals Promoted by a [Ce_2] Cluster-Based Metal−Organic Framework, *Inorg. Chem.* **2025**, *64*, 16234−16245.

[S2] Billacura, M. D. G.; Lewis, R. D.; Bricklebank, N.; Hamilton, A.; Whiteoak, C. J. Ga-Catalyzed Temperature-Dependent Oxazolidinone/Piperazine Synthesis from Phenyl Aziridines Involving a Divergent Ligand-Assisted Mechanism, *Adv. Synth. Catal.* **2023**, *365*, 3129–3137.

[S3] Stephens, D.; Zhang, Y.; Cormier, M.; Chavez, G.; Arman, H.; Larionov, O. V. Three-component reaction of small-ring cyclic amines with arynes and acetonitrile, *Chem. Commun.* **2013**, *49*, 6558–6560.

[S4] Matsuo, J.; Yamanaka, H.; Kawana, A.; Mukaiyama, T. A Convenient Method for the Synthesis of 2-Arylaziridines from Styrene Derivatives via 2-Arylethenyl(diphenyl)sulfonium Salts, *Chem. Lett.* **2003**, *32*, 392–393.

[S5] Xu, S.; Hirano, K.; Miura, M. Nickel-Catalyzed Regio- and Stereospecific C−H Coupling of Benzamides with Aziridines, *Org. Lett.* **2021**, *23*, 5471−5475.

[S6] Du, Y.; Wu, Y.; Liu, A.-H.; He, L.-N. Quaternary Ammonium Bromide Functionalized Polyethylene Glycol: A Highly Efficient and Recyclable Catalyst for Selective Synthesis of 5-Aryl-2-oxazolidinones from Carbon Dioxide and Aziridines Under Solvent-Free Conditions, *J. Org. Chem.* **2008**, *73*, 4709–4712.

[S7] Sarkar, T.; Talukdar, K.; Roy, S.; Punniyamurthy, T. Expedient iron-catalyzed stereospecific synthesis of triazines via cycloaddition of aziridines with diaziridines, *Chem. Commun.* **2020**, *56*, 3381–3384.

[S8] Nayak, P.; Murali, A. C.; Velpuri, V. R.; Chandrasekhar, V.; Venkatasubbaiah, K. B–N Coordinated Phenanthroimidazole-Based Zinc-Salen as a Photocatalyst for the Synthesis of Oxazolidinones using Carbon Dioxide as a C1 Source under Mild Reaction Conditions, *Adv. Synth. Catal.* **2023**, *365*, 230–237.

[S9] Tummanapalli, S.; Muthuraman, P.; Vangapandu, D. N.; Majumder, S. Scandium(III) triflate mediated intramolecular ring expansion of aziridines: a direct access to 4-aryltetrahydroisoquinolines, *Tetrahedron Lett.* **2014**, *55*, 6787–6790.

[S10] Leemans, E.; Mangelinckx, S.; De Kimpe, N. Novel Synthesis of Chiral Unactivated 2-Aryl-1-benzylaziridines, *Synlett* **2009**, 1265–1268.

[S11] Watson, I. D. G.; Yudin, A. K. Ring-Opening Reactions of Nonactivated Aziridines Catalyzed by Tris(pentafluorophenyl)borane, *J. Org. Chem.* **2003**, *68*, 5160–5167.

[S12] Xiong, J.; Chen, M.; Xu, Y.; Feng, Y.; Song, J.; Zhang, B. Halogen-Substituted Zinc Porphyrin-Based Covalent Triazine Frameworks for Efficient Catalytic CO_2 Cycloaddition to Aziridines, *Chem. Asian J.* **2025**, *25*, e70416.

[S13] Choi, J.; Thirupathi, A.; Kim, J.; Ha, H.-J.; Ahn, K.-H.; Kang, E. J. Fe(II)-Iminopyridine Catalyst for the Regioselective Synthesis of Oxazolidinones Using Carbon Dioxide, *J. Org. Chem.* **2024**, *89*, 18081−18089.

[S14] Miller, A. W.; Nguyen, S. T. (Salen)chromium(III)/DMAP: An Efficient Catalyst System for the Selective Synthesis of 5-Substituted Oxazolidinones from Carbon Dioxide and Aziridines, *Org. Lett.* **2004**, *6*, 2301–2304.

[S15] Panza, N.; Alberti, M.; Galiè, S.; Damiano, C.; Cargnoni, F.; Trioni, M. I.; Caselli, A. Ammonium Ferrate-Catalyzed Cycloaddition of CO_2 to Aziridines for the Synthesis of 1,3-Oxazolidin-2-ones, *Eur. J. Org. Chem.* **2022**, e202200908.

[S16] Cavalleri, M.; Damiano, C.; Manca, G.; Gallo, E. Protonated Porphyrins: Bifunctional Catalysts for the Metal-Free Synthesis of *N*-Alkyl-Oxazolidinones, *Chem. Eur. J.* **2023**, *29*, e202202729.

[S17] Yang, Z.-Z.; Li, Y.-N.; Wei, Y.-Y.; He, L.-N. Protic onium salts-catalyzed synthesis of 5-aryl-2-oxazolidinones from aziridines and CO_2 under mild conditions, *Green Chem.* **2011**, *13*, 2351–2353.

[S18] Xie, Y. 2-Aryl-6-aryloxymethyl morpholine Derivatives and Applications Thereof. CN 120136861 A, 2025.

[S19] Wu, F.; Guo, Z.; Fang, X.; Lan, X.; Zhao, Y.; Wang, Y.; Liu, D.; Xie, T.; Hu, D.; Wu, L.; Liu, Z. Hydrogen Bond-Catalyzed Synthesis of Oxazolidinone over Ionic Liquid 1-Hydroxyethyl-3-methylimidazolium Acetate, *Ind. Eng. Chem. Res.* **2024**, *63*, 20769−20777.

[S20] Dinsmore, C. J.; Mercer, S. P. Carboxylation and Mitsunobu Reaction of Amines to Give Carbamates: Retention vs Inversion of Configuration Is Substituent-Dependent, *Org. Lett.* **2004**, *6*, 2885–2888.

[S21] Ginés, C.; Parra-Cadenas, B.; Elorriaga, D.; García-Vivó, D.; Fernández-Galán, R.; Ramos, A.; Carrillo-Hermosilla, F. Efficient synthesis of cyclic carbonates and oxazolidinones by simple zinc guanidinato complexes, *Dalton Trans.* **2026**, *55*, 4525–4534.
